# Scaling and Population Loss in Mexican Urban Centres

**DOI:** 10.1038/s41467-026-75780-5

**Published:** 2026-07-25

**Authors:** Gonzalo G. Peraza-Mues, Eugen Resendiz, Rodolfo Figueroa-Soriano, Rafael Prieto-Curiel, Roberto Ponce-Lopez

**Affiliations:** 1 Tecnologico de Monterrey, Center for the Future of Cities, Monterrey, Mexico; 2 Tecnologico de Monterrey, School of Government and Public Transformation, Monterrey, Mexico; 3 Tecnologico de Monterrey, School of Architecture, Art and Design, Mexico City, Mexico; 4 Complexity Science Hub, Vienna, Austria

**Keywords:** Geography, Social sciences, Statistical physics

## Abstract

Despite its pervasive implications, many cities worldwide continue to expand in a fragmented, horizontal manner. We analyse urban growth dynamics in 69 Mexican metropolitan areas from 1990 to 2020 using census data, developing a model of urban form change based on population size, density, and spatial configuration. We employ a radial probability density function and the urban expansion factor to create a framework for comparing urban expansion over time and across different regions. Over the past three decades, Mexico’s urban population has nearly doubled. However, populations have shifted outward, resulting in a decline of 2.5 million residents in central areas. Our analysis shows that distances from the city centre have increased by 28% on average, driven by population losses in central zones combined with growth in peripheral regions.

## Abstract (web summary)

We analysed urban growth dynamics in 69 Mexican metropolitan areas from 1990 to 2020 using radial scaling analysis. In 30 years, the urban population shifted outwards, with all central zones losing 2.5 million people, absorbed by the peripheries.

## Introduction

The global population is increasingly urban, with urban inhabitants nearly doubling between 1990 and 2020 and projected to grow by another 60% by 2050, while the rural population has already peaked and is expected to decline^1^. This demographic shift suggests that future population growth will occur primarily in cities. In this context, cities respond to population growth in two main ways: by increasing density within their existing built-up areas and by expanding outward into surrounding areas. These processes are intertwined. In recent decades, most urban population growth has been absorbed through outward expansion, often in a fragmented, unplanned manner^2^. This kind of fragmented growth has long-term implications for cities’ resilience, sustainability, and spatial equity^3–6^.

Urban sprawl places a significant burden on cities^7,8^. It increases travel distances and creates a fragmented labour market, particularly in areas with inadequate transit infrastructure^9^. Additionally, sprawl diminishes access to essential services such as water and sewage^10^, and increases the presence of informal settlements^11^. Despite extensive research on the characteristics and measurement of urban sprawl, a notable knowledge gap persists regarding the population dynamics that drive urban expansion. Traditionally, urban economics suggests that population densities should be highest in central areas where jobs and services are concentrated^12^. However, a counter-trend is emerging in many cities, with central regions experiencing population decline. In contrast, peripheral areas are growing even faster, resulting in higher population densities on the fringe than in the core. This pattern is a key driver of urban fragmentation^13^. The population’s retreat from the centre to the peripheries gives rise to numerous urban issues commonly attributed to sprawl in fast-growing urban areas. In multiple cities, the impact of this sprawl is exacerbated by weak institutional capacity, unplanned growth in peripheral areas, and the neglect of cities’ cores, leading to the proliferation of informal settlements, fragmented labour markets, insufficient and inadequate infrastructure, services, and longer travel distances^9,11,14^. The city’s core abandonment, rather than sprawl itself, is a critical yet under-examined force in urban dynamics. This phenomenon exacerbates spatial and social inequalities, ultimately reducing urban efficiency.

These demographic shifts have been associated with distinct spatial patterns and are often described within the traditional framework of urban sprawl. While expansion refers to the physical growth of cities, sprawl captures the low-density, car-dependent, and fragmented development that typically accompanies unplanned growth on cities’ peripheries^2^. Research has widely examined and measured sprawl through various methods, including geographic analysis^15^, cellular automata^16^, land-use models^17^, and spatial indicators of density and land-use mix^18^. Factors contributing to sprawl include the relocation of jobs and industries to the urban periphery^19,20^, and their consequences, such as the spatial mismatch between housing and employment^21^, as well as the development of car-oriented urban systems^22^. Additionally, although much research on changes in city size focuses on the impact of population growth, demographic trends in many countries (such as Hungary, Bulgaria, Japan, and Italy) suggest that both national and urban populations are likely to shrink^1^. It remains unclear, however, whether cities experiencing population decline tend to sprawl or become more compact. In terms of urban form, is population decline simply a reverse process of population increase?

The causes and consequences of urban core abandonment, although recognised as a significant issue, have been poorly captured and explained. Several functions have been proposed for modelling urban radial population distributions, including the negative exponential function^23,24^, the shifted normal^25^, inverse power law distributions^26^, and inverse-S functions^27^. For example, a density gradient based on a negative exponential function has been used to quantify population movement towards urban peripheries in cities worldwide^28,29^. However, no single model universally fits all cities, as different approaches best represent different contexts. These functions typically work best at coarse resolutions of 1 km or more and fit poorly near the centre, where we aim to model population decline^23^. Additionally, some decay parameters often yield conflicting results and a poor overall fit^30^. Similarly to population distribution, radial land density gradients have been recently modelled using either negative exponential^31^ or inverted-S functions^32,33^. While these models successfully capture several regularities in the radial distribution of land density (the ratio of built-up to available land), land density and urban population do not necessarily follow the same distribution^34^. This difference is particularly relevant for urban regions facing depopulation, where the built environment persists even as the population declines. Consequently, few existing models explicitly capture, forecast, or quantify one of the most harmful urban dynamics: the abandonment of city centres.

Distance to the city centre is one of the most critical features of urban form, influencing factors such as transportation, car ownership^35^, air pollution^36^, and even patterns of victimisation^37^. Population dispersion away from the centre has been documented in car-oriented cities in high-income countries such as the US and Canada, and in some cities in India^23,38,39^. However, this pattern is not universal, since cases of reoccupation of the city’s core have also been documented across Europe, North America, and Oceania^28,40,41^. Mexico offers a compelling setting in which to examine these pathways of urban expansion and population redistribution. Between 1990 and 2020, Mexico’s population increased from 81.7 million to 126 million, marking a 54% increase over 30 years^42^. In parallel, the country’s urbanisation rate increased from 71–81%, resulting in the urban population nearly doubling within three decades^42^. This accelerated population growth raises a critical question about the spatial distribution of the population within urban areas: Has growth in Mexican metropolitan areas been evenly distributed, remained concentrated in traditionally dense central zones, shifted toward peripheral regions, or significantly altered overall urban density patterns?

Here, we show that the populations of Mexican cities are retreating from their cores toward their peripheries, a shift towards suburbanisation that we capture through the evolution of their radial population density profiles. We analyse the radial patterns of urban growth at varying distances from the city centre, defined as the historical point of each city’s founding, across 69 metropolitan areas between 1990 and 2020^42^. We characterise this evolution through a scaling relation that does not assume a functional form for the radial profiles, thereby avoiding the fitting issues discussed above. To interpret it, we propose a model that splits population dispersion into two components: an extensive one, which depends on population size, and an intensive one, which captures processes independent of population size, such as housing policies. This model quantifies the shift to the urban periphery, separating the impact of urban agglomeration effects (the extensive component) from other processes (the intensive component) operating across the entire system, such as federal housing and infrastructure policies.

## Results

**Fig. 1:**
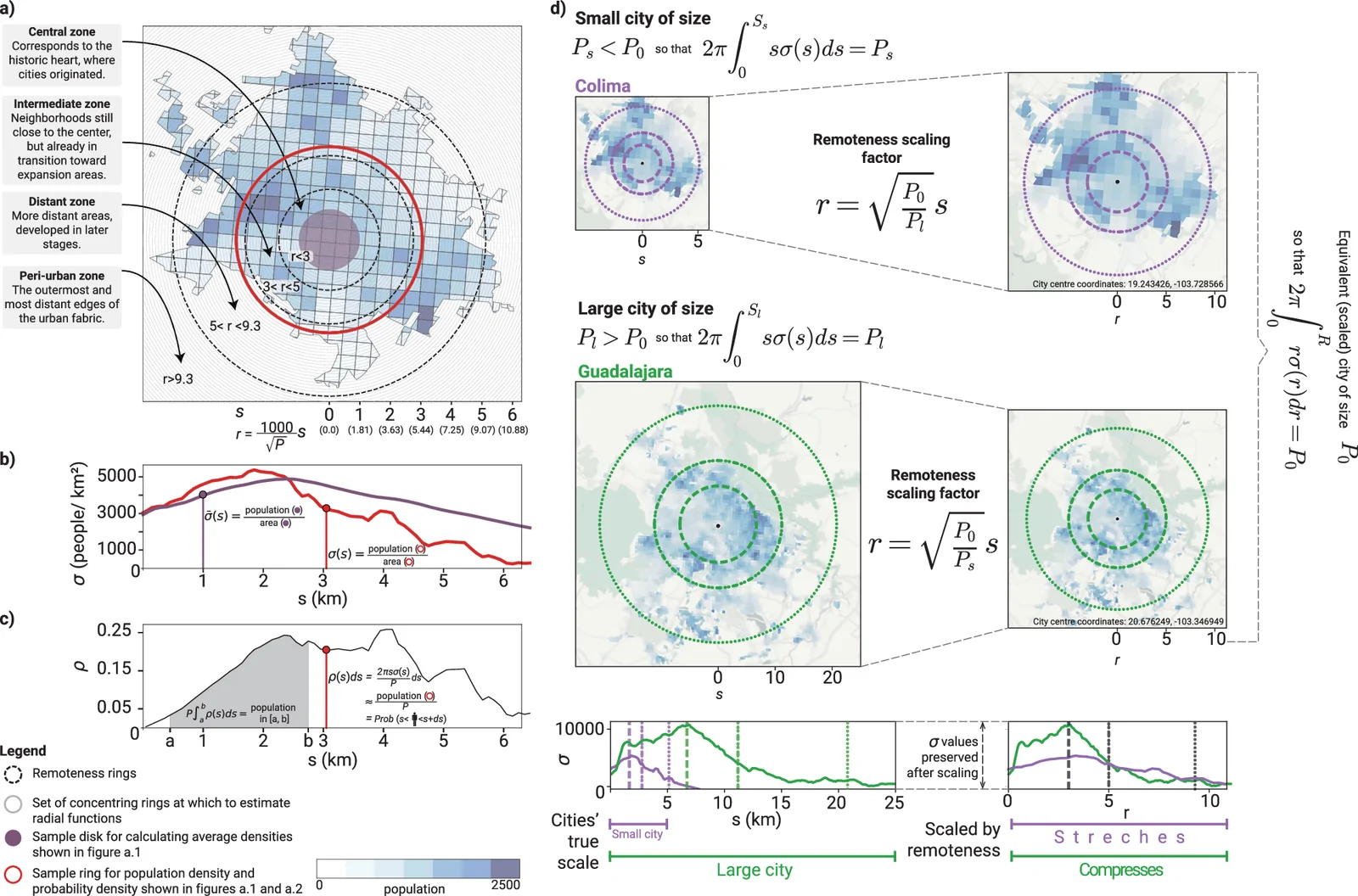
Radial analysis methodology and remoteness scaling. **a** Demonstration of the radial analysis methodology employed, with the city of Colima as an example. Grid cells with population counts (blue) are intersected with concentric rings of 100 m width (grey) at which radial functions are evaluated. We calculate the population within each ring as the sum of the population of grid cells intersecting the ring, weighted by the fraction of each cell’s area contained in the ring. Distance to the centre is labelled *s* and remoteness *r* (in parentheses). Urban regions comparable across cities are defined by their remoteness values and labelled as central, intermediate, distant, and peri-urban, with their boundaries shown as dashed black circles. **b** Population density *σ*(*s*) (red) and average population density $\bar{\sigma} (s)$ (purple) for Colima. Population density is evaluated as the population within a ring over the area of the ring. The red point corresponds to the value of the red ring in (**a**). Average population density is evaluated as the population within a disk of radius *s* over the area of the disk. The purple point corresponds to the average population density over the purple disk in (**a**). **c** Radial probability density function *ρ* and its relation to population density *σ*. The quantity *ρds* is the probability of finding a random person between distances *s* and *s* + *ds* from the city centre, and is approximately the population of the ring over the total population of the city *P* (see “Methods”). **d** Demonstration of remoteness scaling. Two cities, one with a small population (Colima), *P*_*s*_, and one with a large population (Guadalajara), *P*_*l*_, are not comparable in their true scale. Remoteness scales both cities to a comparable size corresponding to a city of population *P*_0_ = 1 × 10^6^, while maintaining the integral relation between density and population, 2*π*∫ *sσds* = *P* (see “Methods”). The scaling preserves the original population density values, allowing comparison between both cities. Further details on remoteness are provided in Supplementary Note 2.

Data from four census waves, spanning 1990 to 2020 at 10-year intervals, were used to measure changes in population density distribution and the effects on distances to the urban centre in 69 metropolitan areas of Mexico^42^. We used the 2020 definitions of Mexican metropolitan areas^43^ and considered the urban population within the central urban cluster’s 2020 limits. The urban population within the geographical limits was disaggregated onto a regular grid with a resolution of 470 × 470 m, allowing us to track population change within each cell over time (Fig. 1). More details in “Methods”.

**Fig. 2:**
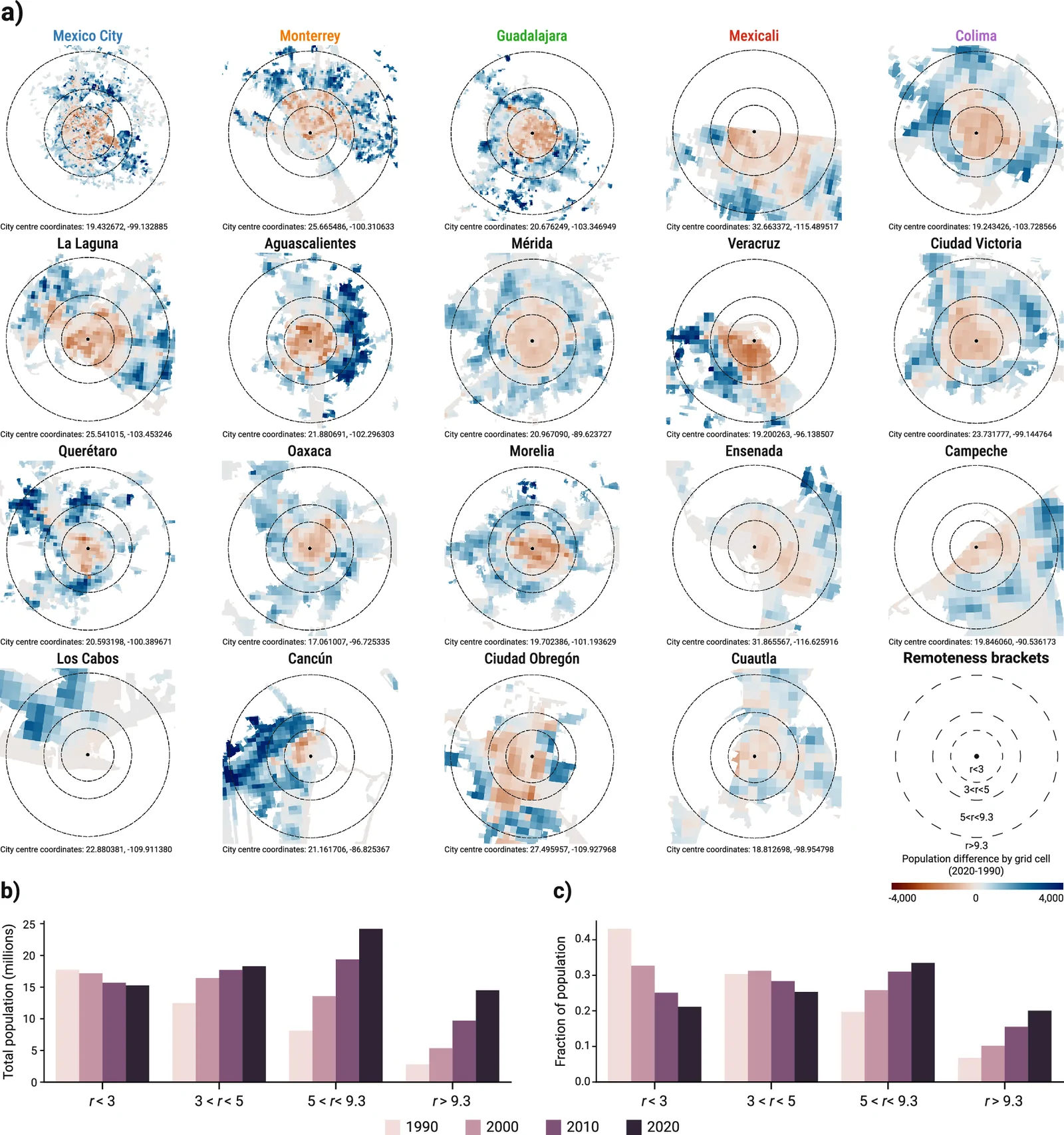
Population loss in the centres of Mexican metropolitan areas. **a** Population difference by grid cell (2020–1990) for 19 Mexican metropolitan zones. City centres are denoted as black dots. In all cases, cities experience population loss around the city centre (red) and population gains in the periphery (blue). Remoteness values that classify parts of a city into central, intermediate, distant and peri-urban are shown as concentric circles for each city (with cut-off values of *r* = 3, 5, and 9.3, obtained elsewhere with a classification tree over the constructed surface of a region^10^). Title colours refer to line colours in Figs. 3, 5. Larger versions of these maps are available in Supplementary Note 8 and online at CitiesMoving.com/UrbanDispersion. **b** Population aggregated over all metropolises at different remoteness brackets for all census years. **c** Fraction of total population at each remoteness bracket for the same years.

Aiming to capture the spatial dynamics of dispersion and expansion as these cities grow, we analysed any location in a city using concentric rings around the historical point of origin^44–46^ (Fig. 1). The city centre is typically defined as the historical location where the city was first established; although other points could be considered, this has little impact on a radial analysis of the city (see Supplementary Note 1). Results show that, despite population growth, all cities experienced a decline in population near their centre. In 1990, approximately 485,000 people lived within 5 km of Monterrey’s centre, but by 2020, this number had decreased to 377,000 (Fig. 2). A similar trend was observed in Guadalajara, where the population within 5 km of the centre dropped from 866,000 to 626,000, whilst the central part of Mexico City lost 166,000 people, during the same 30-year period. Despite the decline in the immediate urban centres, these cities, the three largest metropolitan areas in Mexico, experienced significant population growth during that period, with increases of 99%, 71%, and 38%, respectively.

### Population loss in urban centres

Comparing distances between cities of different sizes is problematic because larger cities naturally occupy larger areas. For example, the metropolitan area of Mexico City (with 21 million people) has nearly 100 times the population of other cities such as Chetumal or Guanajuato. Thus, we scaled distances within cities based on remoteness. Remoteness scales all cities to a comparable size while preserving their population density, avoiding distorting the differences in compactness and distribution inherent to each city^10^ (Fig. 1). Remoteness scales all distances in city *i* by

1 $$r=1000\frac{s}{\sqrt{{P}_{i}}},$$

where *s* is the radial distance to the centre of *i* and *P*_*i*_ is the population of the city. The area of the scaled city is that of an equivalent one (same population distribution and same average density) with a population of one million inhabitants. Remoteness is, then, the distance to the centre of an equivalent city of one million people, for which the scaling factor is one. While larger cities may have higher densities, alternative scaling models, such as those based on Urban Scaling Theory^47^ and homothetic scaling^24^, would change population density values, making direct comparison between cities less interpretable (Supplementary Note 2).

Because remoteness aims to compare different cities transversally (i.e., at the same time point), all cities are scaled using their 2020 populations, so that the same city at different points in time is always rescaled using the same factor. This preserves the relative distances in the temporal evolution of the cities’ population distributions (which we later show displays a different kind of scaling). Remoteness has been classified according to the constructed surface within walking distance, dividing the city into four regions^10^. Locations with *r* < 3 are considered part of the city’s central zone; those with *r* ∈ [3, 5) are the intermediate zone; *r* ∈ [5, 9.3) corresponds to the distant zone, and locations with *r* > 9.3 are classified as the peri-urban zone (Fig. 1). For each city, we classified locations into those four regions based on their remoteness *r* in 2020. This classification effectively identifies the central areas of cities experiencing population decline. All cities in Mexico lost population in the central zone (*r* < 3), while all other areas gained population between 1990 and 2020 (Fig. 2b). Nationwide, central regions lost 2.5 million people between 1990 and 2020. However, the intermediate, distant, and peri-urban zones gained 5.8, 16.1, and 11.7 million, respectively. The decline in the share of the population is even more pronounced. More than 40% of the urban population in 1990 lived in central areas, but this dropped to 22% by 2020 (Fig. 2c). Between 1990 and 2020, the share of the urban population living in a central zone decreased by nearly half.

The distribution of people in Mexican cities in 1990 is characterised by a peak in the central region, with densities decreasing farther from the city centre (Fig. 2b). However, by 2020, this distribution had changed. Initially, population density increases with distance from the centre, peaking in more distant regions, before tapering off in peri-urban regions.

**Fig. 3:**
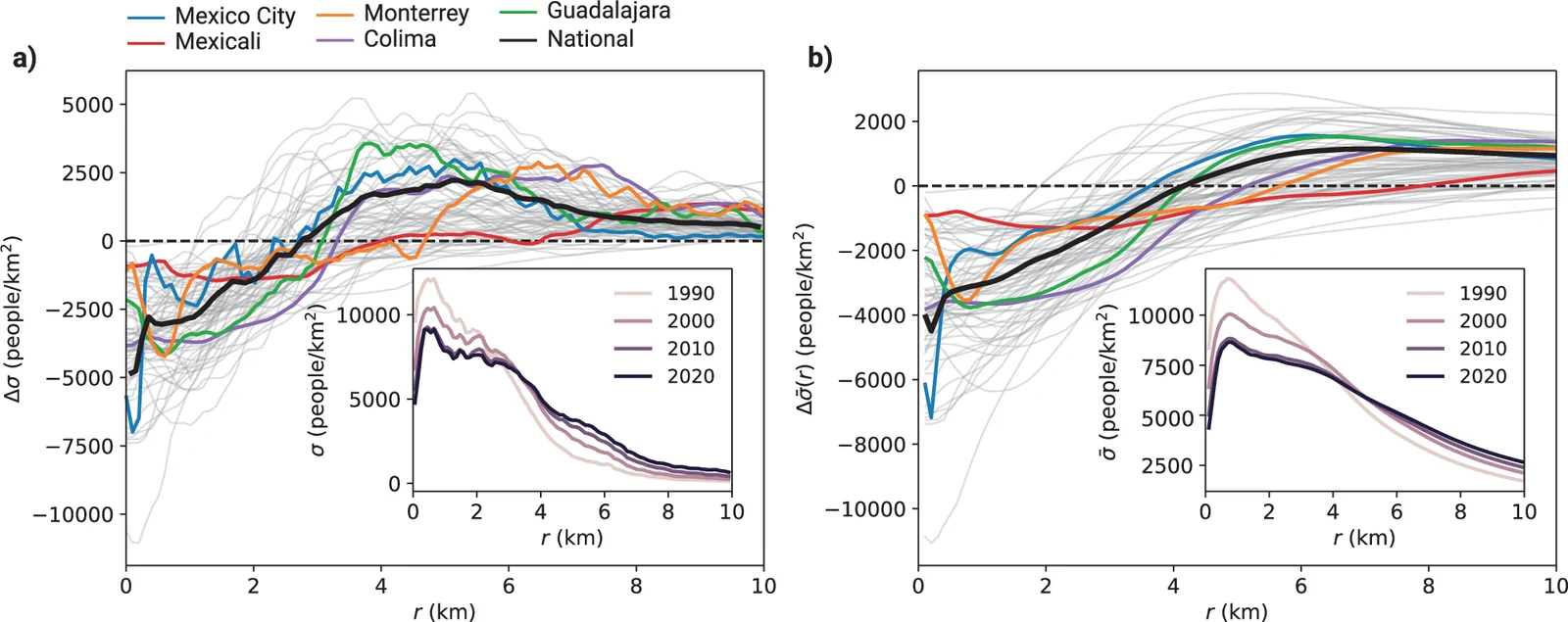
Change in radial population density, 1990–2020. **a** Change in population density (Δ*σ*) between 1990 and 2020 within concentric rings around each city’s centre at remoteness (*r*) measured at each ring’s mid-point in 2020. The inset shows population density *σ*(*r*) at remoteness *r* aggregated for all cities in each census year. **b** Change in population density ($\Delta \bar{\sigma }$) between 1990 and 2020 within discs of radius *r* around each city’s centre. The inset shows average population density $\bar{\sigma }(r)$ up to remoteness *r* aggregated for all cities in each census year. Aggregated values are calculated by treating the populations and areas of all cities at a given remoteness *r* as if they belonged to a single city and evaluating the corresponding density. This is equivalent to a weighted average, with each city’s total population as the weight.

To characterise the abandonment of a city’s core, we calculated two complementary population densities (people/km^2^, Fig. 1b): 1) the population density at distance *s* from the city centre *σ*(*s*, *t*), measured at each of a set of 100 m width concentric rings and 2) average population density up to distance *s*, $\bar{\sigma }(s,t)$, which are average densities within a disk of radius *s* (see “Methods”). Punctual densities *σ* show the actual radial density distribution as a distance from the city centre, while average densities $\bar{\sigma }$ show how density evolves as we consider increasingly larger portions of the city. The difference in these densities between different years, Δ*σ*(*s*) = *σ*(*s*, 2020) − *σ*(*s*, 1990), displays a strong dependence on the distance from the city centre *s* (Fig. 3). Despite rapid population growth between 1990 and 2020, all cities in Mexico became less dense around their centres and more dense away from them. Using remoteness instead of distance to the centre directly, the values of Δ*σ*(*r*) are comparable across cities (Fig. 1). For most cities, Δ*σ* < 0 below values of *r* ∈ (2, 4), and then the function becomes positive (Fig. 3a). Thus, all cities experienced a decline in central-area density between 1990 and 2020.

Urban centres, which usually concentrate jobs and urban infrastructure, have been systematically abandoned, with inner regions (*r *≤ 3) experiencing density losses of as much as 4000 people/km^2^ (Fig. 3a). In Mexico, the density in the central parts of the city decreased by around 1000 people/km^2^, on average, between 1990 and 2020 (bold line in Fig. 3b). The averaged radial densities $\Delta \bar{\sigma }(r)$ show that densification is not homogeneous across the entire urban region. Central regions lose density, but it recovers as larger discs include denser city regions, eventually increasing for large *r*, before decreasing again (Fig. 3b). The national average reveals that most of the loss happened before 2010 (Fig. 3, insets). This difference, as shown in the next section, can be attributed to differences in population growth rates before and after 2010, with growth rates between 2010 and 2020 much lower than before.

### Scaling of radial densities

**Fig. 4:**
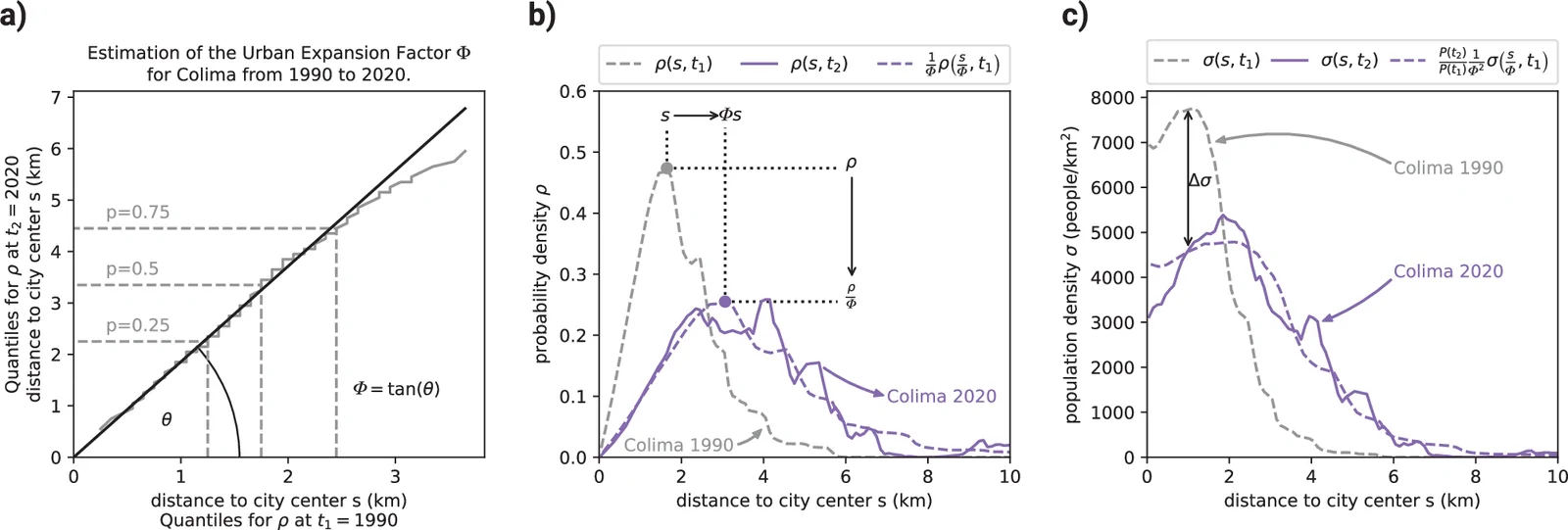
Scaling of the radial population distribution. **a** The Urban Expansion Factor *Φ*_*ij*_ is estimated as the slope ($\tan (\theta )$) of the black line, a Theil-Sen regression through the origin fitted to the Quantile-Quantile plot of the probability density *ρ* at different times *t*_*i*_ and *t*_*j*_ (mean *R*^2^ = 0.99; see “Methods”). The panel shows an example for the city of Colima from *t*_*i*_ = 1990 to *t*_*j*_ = 2020. Quartiles are shown as dashed lines. **b** Radial probability density functions *ρ* for Colima in 1990 (grey) and 2020 (purple). In dashed purple, we show the 1990 density scaled by *Φ*, which coincides with the 2020 density. Distances scale as *Φs* while density values scale as *ρ*/*Φ*, so that the normalisation of the probability density is maintained (see “Methods”). **c** The radial population density *σ* for Colima in 1990 (grey) and 2020 (purple). In dashed purple, we show the 1990 population density scaled according to Eq. (3), which coincides with the 2020 density. A graphical representation of the population density at each year is provided at the bottom.

The radial probability density *ρ*(*s*, *t*) specifies the distribution of people in the city at time *t* as the distribution of their radial distances *s*, so *ρ*(*s*, *t*) d*s* is the probability of finding a randomly chosen person between *s* and *s* + d*s* (Fig. 1c). As a city grows, the radial distribution *ρ*(*s*, *t*) of its population changes. These changes can result from four factors: births, deaths, migration, and housing relocation. But, as has been observed in cities from the US^38^, India^48^, across Africa^11^, and Germany (after the fall of the Berlin Wall)^49^, these changes often reflect feedback loops between policy interventions (e.g., new infrastructure) and their intended and unintended consequences. In the US, for example, car-oriented development led to higher car ownership, greater infrastructure, and increased parking demand^35^, resulting in suburban growth and further car dependency. These changes can lead to a new population distribution that depends on individual decisions and displays self-organising characteristics^50^. Thus, even if each household chooses its residential location individually in an open market, the overall redistribution of locations shows a general, organised pattern observed across many cities (see Supplementary Note 4). In all Mexican cities considered, the updated population distribution can be obtained from the old one by stretching along the radial distance, while scaling down the density values so that *ρ* remains normalised to one (Fig. 4b). By characterising this scaling, we can reevaluate the magnitude of population loss in urban central regions and its dynamics.

**Fig. 5:**
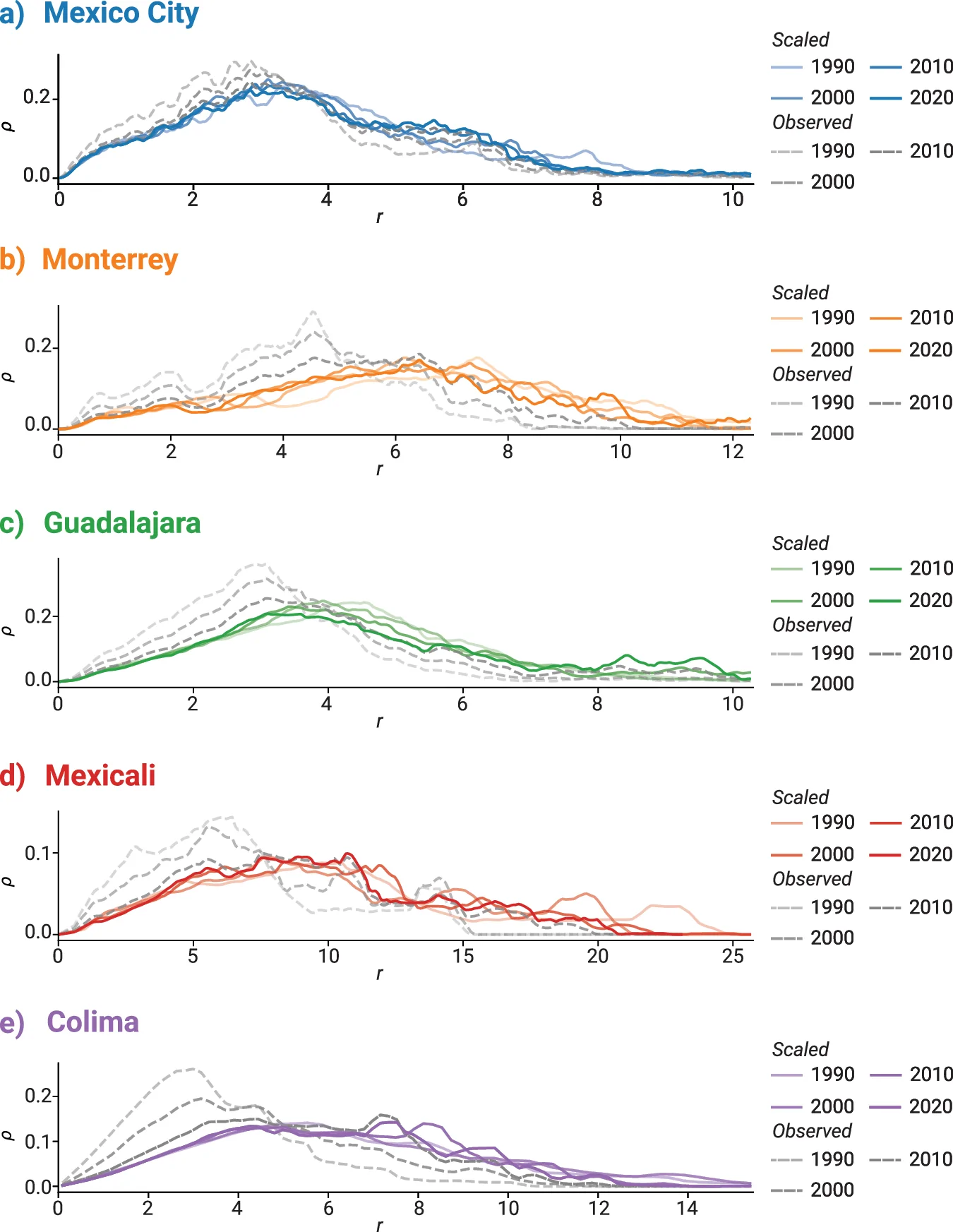
Rescaled radial probability densities for five cities. Scaling of radial probability density functions *ρ* for the five cities shown in Fig. 2: **a** Mexico City, **b** Monterrey, **c** Guadalajara, **d** Mexicali, and **e** Colima. Remoteness in 2020 is used on the horizontal axis for comparison purposes. The grey lines represent the density functions observed at 1990, 2000, 2010, and 2020. The light grey represents 1990, while the dark grey represents 2020. Coloured lines are the density functions rescaled up to 2020 using Eq. (2) with the empirically estimated scaling factors *Φ*_*ij*_. There is an agreement of rescaled functions around the centre, with deviations appearing in the tails of the distribution at large values of remoteness. In all cases, the density stretches along the horizontal axis and compresses along the vertical axis, reproducing the aggregated trend of Fig. 2b. Plots for all cities are shown in Supplementary Note 8.

We empirically find that a city’s radial probability density *ρ*, at some time *t*_*j*_, is a scaled version of the same function at a previous time *t*_*i*_ (Fig. 5). Expressed mathematically, scaling is described by the equation

2 $${\rho} ({s},{t}_{j})=\frac{1}{{{\varPhi}}_{ij}} {\rho} \left(\frac{s}{{{\varPhi}}_{ij}},{t}_{i}\right),$$

where *Φ*_*ij*_ is a scaling factor, which is different for each city and each period (*t*_*i*_ to *t*_*j*_). Expression (2) is the only possible scaling relation for a probability density function, given it preserves normalisation (i.e., the density must integrate to one) after scaling (see “Methods”). Given its relation to urban expansion, we call *Φ*_*ij*_ the urban expansion factor. Equation (2) induces a similar relation for *σ* through the relation *ρ* = 2*πsσ*/*P* (see “Methods”). Thus, population must scale as

3 $$\sigma (s,{t}_{j})=\frac{P({t}_{j})}{P({t}_{i})}\frac{1}{{{{{{\varPhi} }}}}_{ij}^{2}}\sigma \left(\frac{s}{{{{{{\varPhi} }}}}_{ij}},{t}_{i}\right),$$

where *P*(*t*) is the population of the city at time *t*. We estimate the scaling factors *Φ*_*ij*_ from the linear relation between the distribution quantiles *Q*(*p*, *t*_*j*_) = *Φ*_*ij*_*Q*(*p*, *t*_*i*_) induced by (2), where *Q*(*p*, *t*) is the quantile function at time *t* (Fig. 4a, details in “Methods”). When distribution functions for *t* < 2020 are rescaled (according to Eq. (2)), we recover the distribution at *t* = 2020 (Figs. 4, 5). Whenever *Φ*_*ij*_ > 1, the distribution *ρ* stretches out from the origin, increasing the city’s footprint. This is the case for all metropolitan areas except for Tlaxcala-Apizaco, a city composed of two separate cities that grew by infilling the space between them. For *Φ*_*ij*_ < 1, cities contract towards their centre. This expansion/contraction is proportional to the magnitude of *Φ*_*ij*_.

**Fig. 6:**
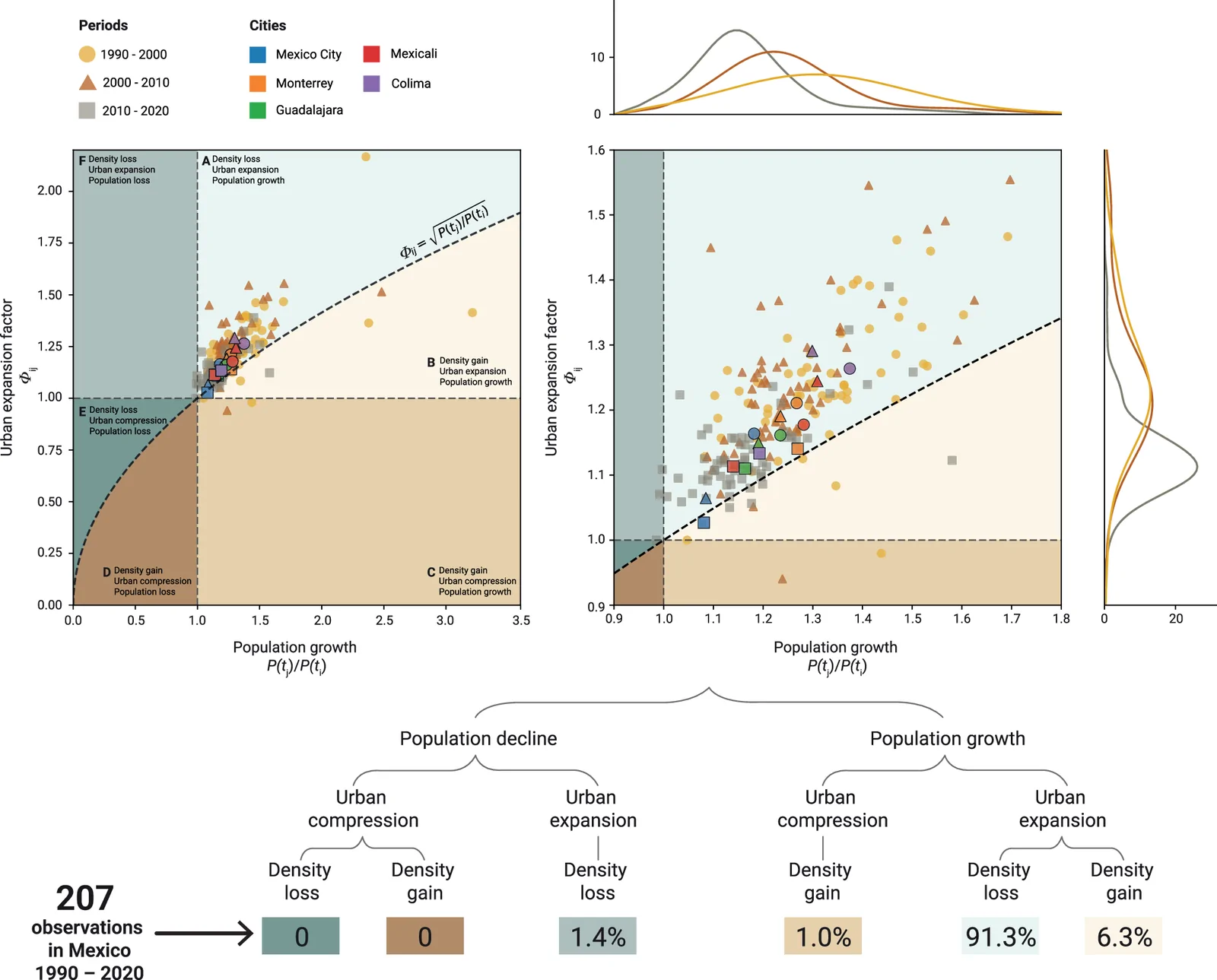
Phase space of population growth versus urban expansion. Growth factor *P*(*t*_*j*_)/*P*(*t*_*i*_) (horizontal axis) and empirical scaling factors *Φ*_*ij*_ (vertical axis) for all cities and all three intercensal periods. Both axes are on a logarithmic scale. The right plot zooms into the cluster of cities to distinguish individual points. Dotted lines divide regions with different population dynamics. Marginal distributions for *Φ*_*ij*_ and *P*(*t*_*j*_)/*P*(*t*_*i*_) are shown as kernel density estimates, coloured for each intercensal period. We present data only for the intercensal periods (Δ*t* = 10 years) for clarity. Data for all available values of Δ*t* are shown in Fig. 8.

The average radial population density $\bar{\sigma }$ follows the same scaling relation (3). Whether this average density increases or decreases after scaling is determined by the values *Φ*_*ij*_ and the population growth factor *P*(*t*_*j*_)/*P*(*t*_*i*_) as follows: Consider a disk of radius *s* at *t*_*i*_, whose density is given by $\bar{\sigma }(s,{t}_{i})$, and its scaled counterpart at *t*_*j*_, with radius *Φ*_*ij*_*s*. According to Eq. (3), the average density of the scaled disk decreases when

4 $${{{{{\varPhi}}}}}_{ij} > \sqrt{\frac{P({t}_{j})}{P({t}_{i})}}$$

and increases if the inequality is reversed. This result is independent of the city radius, so it applies to discs at different scales (e.g., a disk covering the city centre (*r* < 3)). When Eq. (4) is treated as an equality, the city scales while preserving its average density and distribution, i.e., remoteness scaling. In such cases, the density function *σ* is just stretched away from the origin, preserving density values (Fig. 1d). This boundary divides the regions of density loss and gain in the space of variables *P*(*t*_*j*_)/*P*(*t*_*i*_) - *Φ*_*ij*_ (Fig. 6).

Between two consecutive periods, a city belongs to one of six possible regions, depending on its population growth *P*(*t*_*j*_)/*P*(*t*_*i*_) and its urban expansion factor *Φ*_*ij*_. (Fig. 6):

A. **Density loss, and urban expansion, with population growth.** Redistribution of existing population away from the centre, combined with preferential allocation of new residents in the periphery, results in cities that grow while losing average density. The vast majority of Mexican metropolitan areas are in this region. Most cities (67 of 69) appear at least once, accounting for 189 of 207 observations (91%).
B. **Density gain and urban expansion, with population growth.** Cities in this region expand (*Φ*_*ij*_ > 1), yet the fraction of new people allocated to the footprint is sufficient to increase average density. Perhaps part of this density gain is due to vertical expansion^51^. Eleven cities were present in this region at least once, including Mexico City (for the period 2010–2020).
C. **Density gain and urban compression with population growth.** In this region, the surface of the city compacts (*Φ*_*ij*_ < 1) even as their populations increase. This combination always results in an increased average density. Only one city (Tlaxcala-Apizaco, composed of two large urban centres with growth between them) appears in this region for the periods 1990–2000 and 2000–2010.
D. **Density gain and urban compression, with population loss.** Although no cities were observed in this region, they would appear if they shrank faster than they lose population.
E. **Density loss and urban compression, with population loss.** No cities were observed in this region; however, they would appear if they lost density despite contracting due to a faster population decline.
F. **Density loss and urban expansion, with population loss.** Cities that lose population (*P*(*t*_*j*_)/*P*(*t*_*i*_) < 1) but expand nonetheless (*Φ*_*ij*_ > 1). This combination always leads to a loss of density. The remaining population of cities in this region is redistributed away from the urban centre. Only three cities were observed in this region (Acapulco, Minatitlán, and Poza Rica), all for the period 2010–2020.

The case of cities experiencing density loss and urban expansion amid population growth is the most frequently observed scenario in Mexican cities over the past three decades. These trends were exacerbated by reforms to Mexico’s housing finance agencies in the early 1990s^52^. These reforms enabled private companies to build housing, often labelled as more affordable, in peripheral areas where land was cheaper. The result is that, although the city has a larger population, it also encourages residents of the city’s central zones to move to the peripheries^38^. Yet, these housing policies were not matched with the provision of essential services (e.g., transport, water, sewage, and schools)^10^. The other scenarios are discussed in the Supplementary Note 5.

**Fig. 7:**
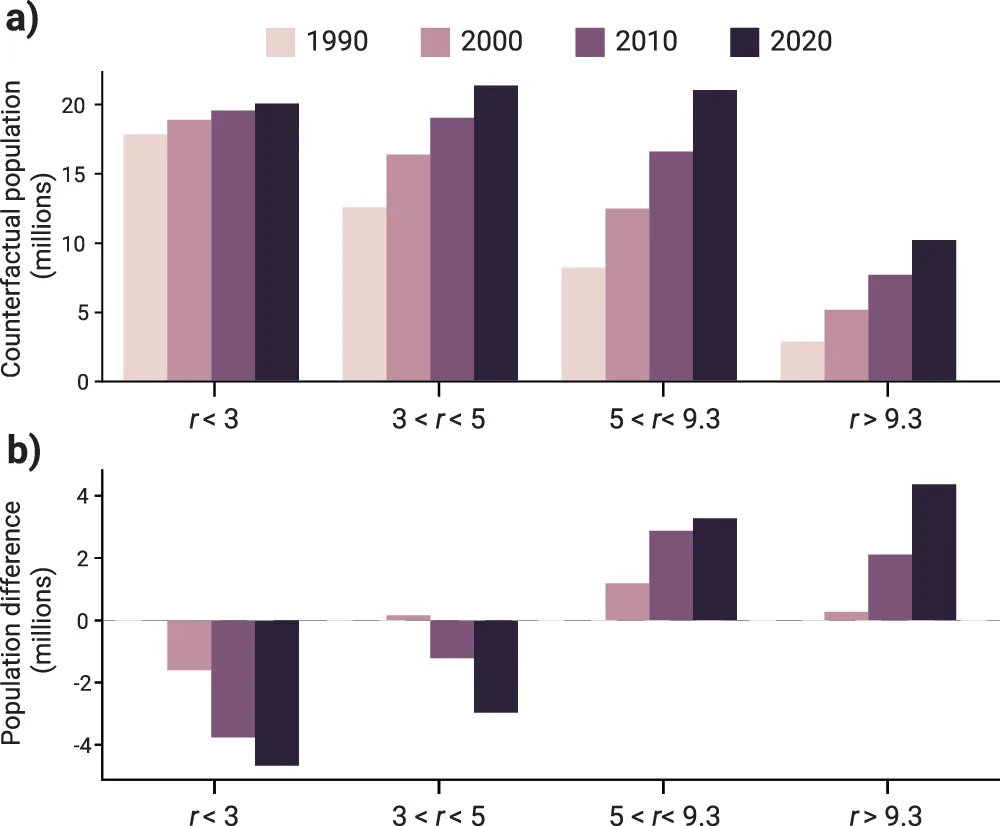
Observed versus density-preserving counterfactual populations. **a** Population aggregated over all metropolises at different remoteness brackets for all census years corresponding to the counterfactual scaling at constant average density. **b** Difference between the observed population and the counterfactual population at each remoteness bracket for the same years.

What would a city look like when it grows while preserving its average density? Given the empirical evidence of scaling and density loss in Mexican cities, it is relevant to compare the observed density with the counterfactual case in which the city’s average population density is preserved. The scaling factors for Mexican cities are usually larger than those of density-preserving scaling. The extent of density loss relative to the counterfactual indicates that many more individuals have been displaced than would have been necessary if average residential densities had remained constant. To compute this comparison, we take the observed density *σ*(*s*, 1990) as the base density, and scale it to the *t*_*j*_ = 2000, 2010, 2020 values, using the observed population counts with ${{{{{\varPhi} }}}}_{ij}=\sqrt{P({t}_{j})/P({t}_{i})}$. We then use these scaled versions to compare the corresponding observed and counterfactual population counts and fractions at each year (Fig. 7). Between 1990 and 2020, 4.7 million people were displaced from the city centre, nearly double the number in a counterfactual scenario that preserved density. These people have relocated to the peripheral zone of the city.

### Modelling the urban expansion factor

**Fig. 8:**
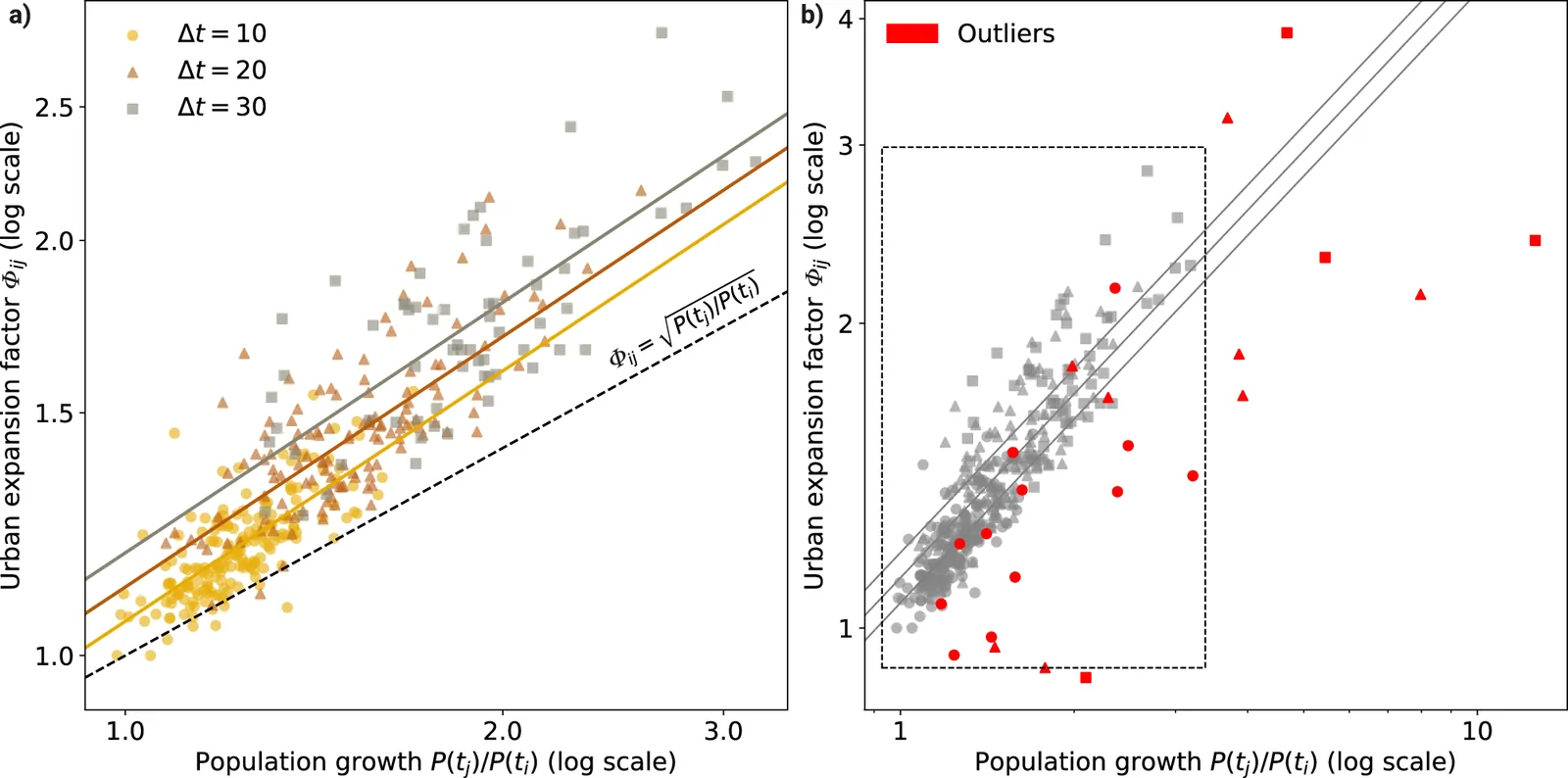
Log-linear model of the urban expansion factor. Empirical scaling factors *Φ*_*ij*_ plotted against the growth factor *P*(*t*_*j*_)/*P*(*t*_*i*_) for all cities and all possible combinations of *t*_*i*_, *t*_*j*_ for *t*_*j*_ > *t*_*i*_, with *t*_*i*_, *t*_*j*_ in {1990, 2000, 2010, 2020}. Both axes are on a logarithmic scale in both panels. **a** The model was fitted to 65 of the 69 cities. Coloured lines correspond to the fitted model (6) for the observed values of Δ*t*, and the dashed line of constant density is shown for comparison; the fit lies in the region of density loss. The model is fitted by ordinary least squares, giving *β* = 0.60 and *α* = 0.0057 with 95% bootstrapped confidence intervals of (0.54, 0.67) and (0.0043, 0.0071), respectively, and *R*^2^ = 0.82 (see “Methods”). **b** The same data shown over the full range for all 69 cities, with the four cities excluded from the fit highlighted in red: a bicentric city experiencing infill growth (Tlaxcala-Apizaco) and three ports with abnormally rapid, tourism-fuelled growth (Cancún, Los Cabos, and Puerto Vallarta). The dotted rectangle marks the extent of panel (**a**).

The urban expansion factors *Φ*_*ij*_ have similar distributions for the periods of 1990–2000 and 2000–2010, while the period of 2010–2020 is characterised by smaller *Φ*_*ij*_ values (Fig. 6). These smaller *Φ*_*ij*_ values correlate with a decline in population growth. The trend in Mexico is for *Φ*_*ij*_ to increase with the population growth factor *P*(*t*_*j*_)/*P*(*t*_*i*_), attributing the slowing expansion rates of recent periods mainly to a decrease in population growth. Including data for the 20-year intervals 1990–2010 and 2000–2010, and the 30-year interval 1990–2020, shows that *Φ*_*ij*_ is log-linearly related to the growth factor *P*(*t*_*j*_)/*P*(*t*_*i*_) (Fig. 8). Therefore, we fit the following model:

5 $${{{{{\varPhi} }}}}_{ij}={\left(\frac{P({t}_{j})}{P({t}_{i})}\right)}^{\beta }{e}^{\alpha ({t}_{j}-{t}_{i})},$$

which is linear in logarithmic variables

6 $$\ln {{{{{\varPhi} }}}}_{ij}=\beta \ln \left(\frac{P({t}_{j})}{P({t}_{i})}\right)+\alpha ({t}_{j}-{t}_{i}),$$

where *α* and *β* are parameters, and Δ*t* = *t*_*j*_ − *t*_*i*_ is the length of the interval over which *Φ*_*ij*_ is evaluated, with observed values of 10, 20, and 30 years. We obtain (using ordinary least squares) that *β* = 0.60(0.54, 0.67) and *α* = 0.0057 (0.0043, 0.0071), with 95% bootstrapped confidence intervals. The coefficient of determination is *R*^2^ = 0.82. The statistically-significant time-dependent *α*Δ*t* term allows for the different intercepts observed for *Φ*_*ij*_ corresponding to different Δ*t* (Fig. 8). When Δ*t* = 0 (*t*_*j*_ = *t*_*i*_), Eq. (6) correctly predicts *Φ*_*ii*_ = 1 (density distributions should remain invariant).

Equation (5) contributes to understanding urban expansion in two ways. Firstly, an exponential term that depends only on the elapsed time *e*^*α*Δ*t*^, and secondly, a power law term that depends on population growth ${(P({t}_{j})/P({t}_{i}))}^{\beta }$, but not on time. The exponential term captures the city’s expansion driven by intensive factors, i.e., factors unrelated to population growth, such as housing relocation choices responding to job location dynamics. The power law term captures expansion driven by population growth, i.e., extensive processes. Expanding at constant average density implies *β* = 0.5 and *α* = 0. Yet, the fitted values for *β* and *α* are both larger than these density-preserving values. Therefore, the national trend is to expand while losing density. This contrasts with a recent reported scaling of *β* = 1/3 (and *α* = 0) obtained from a global sample of cities studied at a single point in time^24^, implying that larger cities tend to be denser, an observation that has been made before, though with possibly different exponents^47,53^. Given that our analysis is longitudinal rather than transversal, this suggests key differences between temporal and transversal urban scaling^54^. A more in-depth comparison among different scaling alternatives is given in Supplementary Note 2.

A positive and statistically significant *α* exponent means that distances in a city increase over time, even in the absence of population growth. The estimated value of *α* corresponds to a displacement of 6 m outside the city centre per year and per km. This means that a location in the population distribution of some city that is 10 km from the centre in 1990 will be 11.8 km from the centre by 2020 in the absence of population growth. We tested the possibility of *α* = 0, (a model without an intercept). However, this is rejected (using an F-test, *F*(1, 388) = 91.52, *P* = 1.3 × 10^−19^), with a reduced *R*^2^ = 0.78.

Model (5) captures different dynamics with varying values of *α* and *β*. Since different sets of cities (e.g., in other countries) may exhibit different exponents, a discussion of these dynamics is warranted and presented in Supplementary Note 6, along with a possible connection to diffusion in physical systems.

#### Impact of scaling on travel distances

Increased distances result in longer commutes, fewer provisions of public transport infrastructure^55^, increased vehicle emissions, reduced access to essential services^10^, and negatively impact overall physical and mental health^56^. To estimate the burden of density loss in the city centre, we consider how the mean and total commuting distances change under the model (5).

Let *γ* be the fraction of commuting trips to work in the city centre. If the city is monocentric, then *γ* ≈ 1; otherwise, it is generally less than one. We express the total commuting distance travelled to the city centre *D* as a function of the mean distance to the centre $\left\langle s\right\rangle=\int\,s\rho ds$, as $D\approx \gamma P\left\langle s\right\rangle$.

We can evaluate the ratio of the observed total distance *D* to the total distance expected for a city that scales at a constant average density (the counterfactual). For a time interval from *t*_*i*_ to *t*_*j*_, this is just the ratio of the mean distance at *t*_*j*_, $\left\langle s\right\rangle ({t}_{j})$, to the counterfactual mean distance ${\left.\left\langle s\right\rangle \right\vert }_{{{{\rm{cf}}}}}({t}_{j})$. Given that $\left\langle s\right\rangle$ scales as $\left\langle s\right\rangle ({t}_{j})={{{{{\varPhi} }}}}_{ij}\left\langle s\right\rangle ({t}_{i})$ (see “Methods”), this ratio is

7 $$\frac{\left\langle s\right\rangle ({t}_{j})}{{\left.\left\langle s\right\rangle \right\vert }_{{{{\rm{cf}}}}}({t}_{j})}=\frac{{{{{{\varPhi} }}}}_{ij}}{{\left.{{{{{\varPhi} }}}}_{ij}\right\vert }_{{{{\rm{cf}}}}}}={\left(\frac{P({t}_{j})}{P({t}_{i})}\right)}^{\beta -0.5}{e}^{\alpha ({t}_{j}-{t}_{i})}.$$

The further *α* and *β* are from their density-preserving values (*β* = 0.5 and *α* = 0), the faster distances grow as the average density decreases. For the 30-year period between 1990 and 2020, the average growth factor for Mexican cities was 2.16. This growth factor, together with the estimated values for *α* and *β*, yields a ratio of 1.28. Thus, on average, distances are 28% longer, due to density loss in Mexican cities.

## Discussion

Over the past three decades, Mexico’s urban population nearly doubled, but the central areas of its cities experienced rapid population decline. Despite overall demographic growth and urbanisation, these areas have lost approximately 2.5 million residents and nearly 5 million compared to the density-preserving counterfactual scenario. These trends were exacerbated by demographic and lifestyle transitions and by housing policies in the 1990s that encouraged new development in peripheral, lower-cost areas^52,57^. However, this shift placed a significant burden on the population, reducing access to services and increasing commuting distances, among other pervasive consequences. Here, we developed a model to quantify population loss in urban centres by examining population dynamics, demonstrating that population distribution scales over time and that this scaling depends on population growth. The scaling of the radial probability density functions enabled us to identify six distinct population dynamics associated with density changes induced by urban expansion. Our analysis revealed that throughout the 30-year study period, most urban centres experienced urban expansion and population growth, accompanied by density loss. In Mexican cities, distances are, on average, 28% larger due to density losses. Urban expansion is an inevitable process that poses challenges for population health, sustainability, equity, and the economy^58^. However, with adequate planning, it can create opportunities to address population and economic needs by fostering compact, diverse urban areas without encouraging uncontrolled urban sprawl or compromising sustainability^59,60^.

Historically, population density peaked in urban cores, but it now peaks several km away from them. The abandonment of city centres across the country suggests a self-organising dynamic in growing cities, where housing redistribution has pushed people to more remote areas^50^. While some basic diffusion models have been shown to lead to scaling, they are limited to reproducing exponential density functions^29^. Yet, here we observe density shifts that such basic models cannot capture. Thus, there is a need for a comprehensive theory that incorporates factors such as housing, transportation, and employment location, along with principles of urban economics and urban scaling, to explain how cities evolve and how their shapes change^12,61^.

Migration, urban growth, and changes in land use have shaped the distribution of people across Latin American cities^58^. In Mexico, this process followed a distinct timeline. After rapid growth in the mid-1900s, the country reached a turning point as birth rates began to decline after the 1960s^62–64^. Lower fertility and smaller households started to slow population growth in central districts before 2010. At the same time, economic changes in the 1990s, including the North American Free Trade Agreement (NAFTA) between Mexico, the USA, and Canada in 1994, and more remittances^65^, increased household buying power and led to a sharp rise in car ownership. The number of cars grew from under 5 million in 1990 to over 40 million today^66^ (see Supplementary Note 3 for a fuller account of these demographic and economic transitions). This made it more common for people to live farther from the city centre and to commute longer distances, accelerating the decline in urban core populations. Our findings show that both the movement of households out of city centres and the growth of new and established residential areas on the outskirts contributed to these trends^67^. This outward migration and the resulting urban sprawl can be better understood by examining the specific supply-and-demand factors that reshaped the national housing landscape.

The population loss in central urban areas and the simultaneous expansion of urban peripheries in Mexico are closely linked to the interaction between supply and demand. On the demand side, rising incomes and a demographic boom over the past three decades led to the rapid formation of new households willing to exercise housing credits accumulated through a Central Provident Fund (CPF)-like system^68,69^. A large pool of households was therefore ready to enter the housing market simultaneously. On the supply side, two structural shifts were decisive. First, the 1992 constitutional reform of land tenure enabled the sale of communal land, sharply expanding the supply of developable land on the urban fringe^70^. Second, beginning in 2001, the national housing agency transitioned from direct housing provision to a financial intermediary role, thereby dramatically expanding credit origination^71,72^. The meeting of credit-ready demand with abundant peripheral land triggered a housing boom, with annual housing production rising from roughly 50,000 units to more than 1.4 million between 2001 and 2013^71^. Together, these dynamics in Mexico fuelled large-scale peripheral housing development, reinforcing the depopulation of central areas and growth in the periphery^68^.

Results for these dynamics can be modelled by analysing the distribution of distances from the city centre, in which contributions from extensive and intensive urban processes have been linked to the magnitudes of the exponents *β* and *α*. Fitted values for these exponents (*α* = 0.0057, *β* = 0.6) place Mexican metropolises in the regime of cities that expand while losing density. Given that the extensive growth of a city is coupled with intensive growth, the abandonment of central urban areas results from various economic, territorial, and demographic factors, as well as the suburbanisation process driven by dysfunctional markets^38^. The observed decline in population in central areas can also be attributed to a lack of affordable housing in these regions, coupled with a shift in consumer preferences toward the periphery, where affordable housing options are more readily available^73,74^.

The magnitude of the *β* exponent captures abandonment driven by population growth. It includes elements such as increased demand for non-residential land uses at the centre, disruptions to the housing market driven by a rising demand for limited available housing, and the establishment of consolidated urban centres in peripheral regions, contributing to the ongoing suburbanisation process^73^. These new locations often emerge around employment, shopping, or business hubs, where modern infrastructure, services, and facilities have been developed, rendering city centres less appealing to new families^75^. The magnitude of the *α* exponent captures the centre decline and the suburbanisation process due to non-growth-related factors. Within our model, these exponents are constant in the 30-year period studied. Changes in the magnitude of urban expansion are mainly explained by corresponding changes in population growth rates (Eq. (5)), rather than in the mechanisms and drivers of urban expansion in this period.

These processes are fuelled by the need for more space for urban development, the emergence of new urban centres in peripheral locations, real estate market speculation, the financialisation of housing, changes in housing regulations^73^, and shifts in consumer preferences. The effects of these urban processes can be addressed through thoughtful urban planning that seeks to contain growth-related scaling by implementing comprehensive housing, transportation, and economic policy strategies to maintain existing population densities.

By using a radial probability density function and the urban expansion factor, we provide a framework for understanding and comparing urban expansion across space and time in growing metropolitan areas. This approach allows the exploration of urban forms experiencing population loss and shrinkage. This framework, applied in urban and transport modelling and planning, helps anticipate the spatial consequences of population shifts and urban sprawl and design evidence-based strategies and interventions that promote more equitable and sustainable urban development.

## Methods

### Urban population mesh

Population data for this study are processed from census data by Mexico’s National Institute of Statistics and Geography (INEGI) for 1990, 2000, 2010, and 2020^42^. We considered other globally available datasets (such as the Global Human Settlement Population (GHS-POP)^76^, and WorldPop^77^), but rejected them, given discrepancies with official statistics^34,78–81^ that may bias analyses of intra-urban population dynamics in Mexican metropolitan areas (Supplementary Note 7). To address these limitations, we built a high-resolution gridded population dataset for the metropolitan areas in Mexico, using official census data for 1990, 2000, 2010, and 2020.

INEGI defines a hierarchical system of administrative units in Mexico, consisting of states, municipalities, localities (urban and rural), and basic geostatistical areas (AGEBs), which are equivalent to the U.S. census tracts. Localities with more than 2,500 inhabitants are classified as urban and subdivided into urban AGEBs, each containing between 1 and 50 city blocks. Only the urban population is spatially disaggregated onto our proposed grid. Population counts at the AGEB level are obtained from the Population and Housing Censuses^42^. AGEB geometries were sourced from the National Geostatistical Framework ^82^ (years 2000, 2010, 2020) and from the System for Census Information Consultation (SCINCE) (year 1990), provided directly by INEGI upon request.

Geographic grids divide space into uniform cells, enabling urban planning, epidemiology, and climate analysis at multiple spatial scales. Compared to traditional administrative boundaries, they provide more granular and spatially consistent insights^83–85^. For this study, the urban grid required spatial alignment of AGEB geometries from the 1990 and 2000 censuses, as these datasets exhibited substantial positional shifts and geometric distortions relative to the accurately georeferenced boundaries of 2020. This issue has been previously reported, but existing correction methods have achieved limited success^86^. The alignment is non-trivial, as AGEB's unique identifiers, required for direct matching across census years, are available for only a subset of units, since they frequently change from one census to another. Identifiers change for several reasons, including the subdivision of existing geometries, reclassification into higher-level administrative units, and the incorporation of newly urbanised areas to reflect population change, among others. As a solution, we developed a custom alignment algorithm that automatically identifies control points between AGEB geometries from consecutive census years. First, the algorithm detects intersection points shared by three or more AGEBs whose identifiers remain unchanged and designates them as control points. These points typically correspond to intersections of arterial streets, which are unlikely to change between census years. The control points are then manually inspected, and any erroneous matches are removed. Finally, the geometries are aligned using a thin-plate spline transformation^87^.

In some cases, we perform manual alignment by constructing a replacement mapping list, in which AGEB identifiers from the year being aligned are matched to a corresponding set of identifiers from the subsequent census year. This mapping can be one-to-one, one-to-many, or many-to-one. Once the mapping list is established, we replace the misaligned geometries with their corrected counterparts. After all AGEBs are correctly aligned, their population counts are disaggregated onto INEGI’s National Geostatistical Grid^88^ level 9, with a spatial resolution of approximately 470 m × 470 m. Population within each cell is the sum of the populations of all-intersecting AGEBs, weighted by the fractional area of each AGEB within the cell.

### Defining radial distributions

The spatial distribution of the residential population in a city is represented by the set of position vectors {**r**_*i*_∣*i* = 1, …, *P*}, where *P* denotes the total population, and *i* indexes every person in the city. In practice, it is not possible to perfectly define the vectors **r**_*i*_, so we assign to each person *i* a probability density function *ρ*_*i*_(**r**), such that *ρ*_*i*_(**r**)*dA* is the probability of finding person *i* within the element area *dA*. Probability densities *ρ*_*i*_ are expected to be peaked around each person’s home location and are often defined as constant within some area element (such as a census block or a grid cell) and zero everywhere else. The expected number of people within element area *dA* around the position **r** is

8 $$\mathop{\sum }_{i=1}^{P}{\rho }_{i}({{{\bf{r}}}})dA=\sigma ({{{\bf{r}}}})dA,$$

where we have defined the two-dimensional population density

9 $$\sigma ({{{\bf{r}}}})=\mathop{\sum }_{i=1}^{P}{\rho }_{i}({{{\bf{r}}}}),$$

with units of people over unit area (here taken as people/km^2^). The integral of the population density over the entire urban area equals the total population of the city, ∫_city_*σ*(**r**)*dA* = *P*. By normalising *σ*(**r**) by the total population of the city, we obtain the probability density function that describes the residential distribution of people within the city,

10 $$\rho ({{{\bf{r}}}})dA=\frac{1}{P}\sigma ({{{\bf{r}}}})dA.$$

The function *ρ*(**r**) can be interpreted as the probability of finding a randomly selected person within the element area *dA*. As a probability density, *ρ*(**r**) is independent of the total population of the city, but it depends on its spatial-physical extent. Therefore, it is not directly comparable across cities of different sizes without appropriate rescaling.

We select the city centre as a reference point and analyse the radial distribution of people (details in Supplementary Note 1). In polar coordinates, we can write *ρ*(*s*, *θ*) = *sσ*(*s*, *θ*)/*P*, where *s* is used for the radial distance to avoid confusion with remoteness *r*. Marginalising over the angular variable *θ*, we obtain the one-dimensional radial probability density

11 $$\rho (s)=\frac{2\pi }{P}s\sigma (s),$$

where *σ*(*s*) is the mean population density at radial distance *s* obtained by applying the mean-value theorem. The function *ρ*(*s*) represents the radial distribution of distances within the city and corresponds to the probability that a randomly selected person is located between distances *s* and *s* + *ds* from the city centre.

### Estimating radial distributions

To estimate the radial population density *σ*(*s*) and the radial probability density *ρ*(*s*), we built concentric rings of width 100 m around each city’s centre (Fig. 1). We calculated the population within each ring as the weighted sum of the population of all grid cells intersecting the ring, where the weights correspond to the fraction of each cell’s area contained within the ring. The value of the point population density *σ*(*s*) is then estimated as the population of the ring whose midpoint is located at a distance *s*, divided by the area of that ring. The radial probability density *ρ*(*s*) is subsequently obtained from Eq. (11). The average population density $\bar{\sigma }(s)$ is estimated as the population within a disk of radius *s* over the area of that disk. Each disk of radius *s* is aggregated from all rings contained within *s*, with the population of the disk being the sum of the populations of the rings.

### Delineating the urban area

Mexican metropolitan areas correspond to the most recent official definition from 2023 (based on the 2020 census)^43^, which distinguishes metropolitan areas, metropolitan municipalities, and conurbated areas. For this analysis, we selected 47 metropolitan areas (out of 48, excluding the metropolitan area of Playa del Carmen, which did not exist in 1990) and all 22 metropolitan municipalities (see Supplementary Note 1 for the complete list). The 2023 definitions apply to 2020; for earlier years, we use the same spatial extent as defined for 2020. Although this approach may include areas that were not yet integrated into metropolitan structure in earlier periods, it allows us to study the temporal evolution of each metropolis using a consistent unit of analysis.

The 2023 definition conceptualises a metropolis as a functional urban unit by explicitly incorporating commuting zones. This represents a major methodological shift, as previous definitions did not account for commuting patterns; for the first time, systematic information on daily travel and commuting was collected in the 2020 census and incorporated into the delineation of metropolitan areas. As a result, the entire area of municipalities containing urban populations is classified as part of the metropolis, even when urbanisation occupies only a small fraction of the municipal territory. This leads to the inclusion of contiguous rural zones that are largely uninhabited, over which radial functions are zero. Since scaling is observed near the urban core and always within the main urban cluster, we truncate all radial functions at their first zero and renormalise the corresponding probability densities to 1. This truncation facilitates the comparison of radial profiles across cities and removes the influence of distant commuting zones, which are more heavily constrained by their historical development and the area’s natural geography. After truncation, we retain approximately 98% of the 2020 population.

### Empirical scaling of *ρ*

The radial probability density function at year *t*_*i*_, expressed as *ρ*(*s*, *t*_*i*_), changes by year *t*_*j*_, according to the equation

12 $$\rho (s,{t}_{j})=\frac{1}{{{{{{\varPhi} }}}}_{ij}}\rho \left(\frac{s}{{{{{{\varPhi} }}}}_{ij}},{t}_{i}\right),$$

where Δ*t* = *t*_*j*_ − *t*_*i*_ can be one of {10, 20, 30}, corresponds to one tuple in the set (*t*_*i*_, *t*_*j*_) ∈ {(1990, 2000), (2000, 2010), (2010, 2020), (1990, 2010), (1990, 2020), (2000, 2020)}, and where *Φ*_*ij*_ is a scaling factor that stretches or compresses *ρ* along the horizontal axis by scaling the radial distances *s*. Equation (12) is the only valid scaling transformation for *ρ*, since it preserves the normalisation of the probability density. This is, for an already normalised *ρ*(*s*, *t*_*i*_), *ρ*(*s*, *t*_*j*_) given by Eq. (12) remains normalised for any *Φ*_*ij*_.

Given that population density *σ* is related to *ρ* by Eq. (11), *σ*(*s*, *t*) must scale as

13 $$\sigma (s,{t}_{j})=\frac{P({t}_{j})}{P({t}_{i})}\frac{1}{{{{{{\varPhi} }}}}_{ij}^{2}}\sigma \left(\frac{s}{{{{{{\varPhi} }}}}_{ij}},{t}_{i}\right).$$

This scaling equation ensures that the radial integral of *σ*(*s*, *t*) equals the population at time *t*. If 2*π*∫ *sσ*(*s*, *t*_*i*_)*ds* = *P*(*t*_*i*_), then 2*π*∫ *sσ*(*s*, *t*_*j*_)*ds* = *P*(*t*_*j*_), with *σ*(*s*, *t*_*j*_) given by Eq. (13).

When the radial probability function scales according to Eq. (12), all central distances scale as *s* → *Φ*_*ij*_*s*. This applies to quantiles and moments of *ρ* as well. More formally, if we define the n-th moment at *t*_*i*_ as

14 $$\left\langle {s}^{n}\right\rangle ({t}_{i})=\int_{0}^{{S}_{i}}{s}^{n}\rho (s,{t}_{i})ds,$$

then, at *t*_*j*_

15 $$\left\langle {s}^{n}\right\rangle ({t}_{j})=	\int_{0}^{{S}_{j}}{s}^{n}\rho (s,{t}_{j})ds\hfill\\=	\frac{1}{{{{{{\varPhi} }}}}_{ij}}\int_{0}^{{S}_{j}}{s}^{n}\rho \left(\frac{s}{{{{{{\varPhi} }}}}_{ij}},{t}_{i}\right)ds\hfill\\=	{{{{{\varPhi} }}}}_{ij}^{n}\int_{0}^{{S}_{i}}{{s^\prime} }^{n}\rho \left(s^{\prime},{t}_{i}\right)ds^{\prime} \hfill\\=	{{{{{\varPhi} }}}}_{ij}^{n}\left\langle {s}^{n}\right\rangle ({t}_{i}),\hfill$$

where we have used the change of variables $s^{\prime}=s/{{{{{\varPhi} }}}}_{ij}$ and *S*_*i*_ and *S*_*j*_ are the maximum radii of the city at *t*_*i*_ and *t*_*j*_, with *S*_*j*_ = *Φ*_*ij*_*S*_*i*_.

We can perform a similar exercise for the quantiles. The quantiles function *Q*_*i*_(*p*) is the radial distance below which we find the fraction *p* of the total population at *t*_*i*_, and is defined through the equation

16 $$\int_{0}^{{Q}_{i}(p)}\rho (s,{t}_{i})ds=p.$$

At *t*_*j*_,

17 $$p=	\int_{0}^{{Q}_{j}(p)}\rho (s,{t}_{j})ds\hfill\\=	\frac{1}{{{{{{\varPhi} }}}}_{ij}}\int_{0}^{{Q}_{j}(p)}\rho \left(\frac{s}{{{{{{\varPhi} }}}}_{ij}},{t}_{i}\right)ds\\=	\int_{0}^{{Q}_{j}(p)/{{{{{\varPhi} }}}}_{ij}}\rho (s^{\prime},{t}_{i})ds^{\prime},$$

where we have used the same change of variables as in (15). By equating equation (16) and (17) we arrive at the linear relationship for quantiles

18 $${Q}_{j}(p)={{{{{\varPhi} }}}}_{ij}{Q}_{i}(p).$$

Relation (18) would appear as a straight line in a quantile-quantile plot (Q-Q plot), with the slope of the line being *Φ*_*ij*_ (Fig. 4a). This enabled us to estimate the scaling factors *Φ*_*ij*_ through a linear fit to (18). We computed quantiles at Δ*p* = 0.01 intervals from *p* = 0.01 to *p* = 1.00 and performed a Theil-Sen regression without an intercept for every city-period combination in our dataset. In practice, scaling is observed in a region around *s* = 0, where the line fits the data well. A Theil-Sen regression is chosen for its robustness to such deviations from scaling at quantiles far from the origin. These deviations cause standard regression to fail to fit the scaling relation where it is stronger (more linear), i.e., around the centre. Furthermore, we restrict the range of *p*-values that enter the regression to *p* = 0.38. This value is chosen as it covers all regions of density loss for all cities in all periods, though in most cases the estimated line fits well beyond this quantile (see Fig. 4a for an example for Colima). The Theil-Sen estimator computes the slope of all points to the origin and takes the median as the estimate. The interquartile range of the slopes is a usual measure of uncertainty, similar to confidence intervals in linear regression. The mean *R*^2^ across the regressions is 0.99, indicating a good fit. Q-Q plots for all cities, along with the regression estimates, are provided in Supplementary Note 8.

### Reporting summary

Further information on research design is available in the Nature Portfolio Reporting Summary linked to this article.

## Supplementary information

Supplementary Information

Reporting Summary

Transparent Peer Review file

## Data Availability

The processed population data generated in this study—the high-resolution gridded population dataset for the 69 Mexican metropolitan areas across the 1990, 2000, 2010, and 2020 censuses, together with the derived radial density functions—have been deposited in the Zenodo repository^89^ under accession code 10.5281/zenodo.20630381, and are also available at the project repository (https://github.com/CentroFuturoCiudades/scaling_depopulation_mex). The raw census data used in this study are third-party data owned by Mexico’s National Institute of Statistics and Geography (INEGI). The 2000, 2010, and 2020 census population counts and AGEB geometries are available in the INEGI database (https://www.inegi.org.mx/). The 1990 AGEB geometries are available under restricted access, because INEGI distributes them under terms of use that do not permit redistribution by the authors; access can be obtained by requesting them directly from INEGI through its System for Census Information Consultation (SCINCE) (https://www.inegi.org.mx/). The processed 1990 population grid derived from these geometries is included in the deposited dataset above, so no restricted-access data are required to reproduce the results reported here.

## Code availability

All code used to build the dataset and perform the analyses (developed in Python) is publicly available at https://github.com/CentroFuturoCiudades/scaling_depopulation_mex and archived on Zenodo at 10.5281/zenodo.20630381.
