# Supplementary material for: Scaling and Population Loss in Mexican Urban Centres
Source: Nat Commun. 2026 Jul 25;17:9039. doi: 10.1038/s41467-026-75780-5 (PMC13507054; doi:10.1038/s41467-026-75780-5)
Supplement: Supplementary file 1 [file 41467_2026_75780_MOESM1_ESM.pdf]

Supplementary Information: Scaling and Population Loss in Mexican Urban Centres

Gonzalo G. Peraza-Mues[1,2], Eugen Resendiz[1,3], Rodolfo Figueroa-Soriano[1], Rafael Prieto-Curiel[4], and Roberto Ponce-Lopez[1,2]

[1]Tecnologico de Monterrey, Center for the Future of Cities, Monterrey, Mexico
[2]Tecnologico de Monterrey, School of Government and Public Transformation, Monterrey, Mexico
[3]Tecnologico de Monterrey, School of Architecture, Art and Design, Mexico City, Mexico
[4]Complexity Science Hub, Vienna, Austria

List of Supplementary items

Supplementary Note 1: Identifying the city centre and the central parts **2**

Supplementary Note 2: Distance to the city centre, remoteness, and alternative scaling exponents **3**

Supplementary Note 3: A country in evolution **6**

Supplementary Note 4: Cities as a self-organising object **7**

Supplementary Note 5: Different scenarios and implications at the city level **7**

Supplementary Note 6: Dynamical regimes of the scaling model **8**

Supplementary Note 7: Error estimation for global population grids **9**

Supplementary Note 8: Data for all metropolitan zones **11**

Supplementary References **81**

Supplementary Note 1: Identifying the city centre and the central parts

We identify the city centre as the historical point where it initially developed. While multiple locations could serve as the city's centre, the main objective is to identify remote areas, for which the exact position of the centre is less important[1]. Studies have shown that defining the city centre as the city hall, for example, and then shifting this point to other locations, has a minor effect on radial analyses of the city[2]. Thus, while the exact definition of the city centre may be ambiguous, it has little influence on which areas are classified as remote or peri-urban[3].

A technique for evaluating the relevance of a city centre definition is to quantify the overlap among different central areas[3]. In other words, the process is to measure the extent to which the rings with $r \leq 3$ intersect. A high degree of overlap indicates that, regardless of the definition used, the delineation of central, inter-urban, distant, or peri-urban areas remains broadly consistent. Across many cities, it was observed that the overlap of the central region is roughly 90 to 95%[3], meaning that although the precise location of the centre of a city might be ambiguous, it has a negligible impact on the overall radial analysis of a metropolitan area.

Centres identified for the 69 metropolitan areas in Mexico are listed in Supplementary Table 1.

Supplementary Table 1: MA: Metropolitan Area. MM: Metropolitan Municipality. Locations are based on historical documentation for each city (e.g., government websites, books, etc.)

Code	Name	ID Type	longitude	Center latitude
01.1.01	Aguascalientes	MA	-102.296304	21.880691
02.1.01	Tijuana	MA	-117.040313	32.535727
02.2.02	Ensenada	MM	-116.625916	31.865567
02.2.03	Mexicali	MM	-115.489518	32.663373
03.2.01	La Paz	MM	-110.313517	24.162093
03.2.02	Los Cabos	MM	-109.911380	22.880381
04.2.01	Campeche	MM	-90.536174	19.846060
05.1.01	La Laguna	MA	-103.453247	25.541015
05.1.02	Monclova-Frontera	MA	-101.416584	26.900959
05.1.03	Piedras Negras	MA	-100.514117	28.705423
05.1.04	Saltillo	MA	-101.000370	25.421838
06.1.01	Colima-Villa de Álvarez	MA	-103.728567	19.243427
07.1.01	Tapachula	MA	-92.264866	14.910261
07.1.02	Tuxtla Gutiérrez	MA	-93.115647	16.754261
08.1.01	Chihuahua	MA	-106.076753	28.636442
08.1.02	Delicias	MA	-105.475929	28.189491
08.2.03	Juárez	MM	-106.486651	31.738583
09.1.01	Ciudad de México	MA	-99.132886	19.432673
10.2.01	Durango	MM	-104.670217	24.023943
11.1.01	Celaya	MA	-100.814069	20.521931
11.1.02	León	MA	-101.682519	21.121933
11.2.03	Guanajuato	MM	-101.253960	21.016606
11.2.04	Irapuato	MM	-101.347182	20.672689
12.1.01	Chilpancingo	MA	-99.501418	17.552148
12.2.02	Acapulco	MM	-99.908527	16.848769
13.1.01	Pachuca	MA	-98.731923	20.127609
13.1.02	Tulancingo	MA	-98.369053	20.080679
14.1.01	Guadalajara	MA	-103.346949	20.676249
14.1.02	Puerto Vallarta	MA	-105.235562	20.608492
15.1.01	Toluca	MA	-99.656914	19.292524
16.1.01	La Piedad-Pénjamo	MA	-102.021884	20.341015

Continued on next page

Code	Name	ID Type	Center longitude	latitude
16.1.02	Morelia	MA	-101.193629	19.702387
16.1.03	Zamora	MA	-102.285794	19.984167
16.2.04	Uruapan	MM	-102.063074	19.420952
17.1.01	Cuautla	MA	-98.954799	18.812698
17.1.02	Cuernavaca	MA	-99.234373	18.921887
18.1.01	Tepic	MA	-104.892515	21.512221
19.1.01	Monterrey	MA	-100.310633	25.665487
20.1.01	Oaxaca	MA	-96.725335	17.061007
21.1.01	Puebla-Tlaxcala	MA	-98.198242	19.043965
21.1.02	San Martín Texmelucan	MA	-98.435504	19.282997
21.1.03	Tehuacán	MA	-97.392613	18.462401
22.1.01	Querétaro	MA	-100.389671	20.593198
23.1.01	Cancún	MA	-86.825367	21.161707
23.2.02	Chetumal	MM	-88.297794	18.493826
24.1.01	San Luis Potosí	MA	-100.976419	22.151694
25.2.01	Culiacán	MM	-107.393899	24.808840
25.2.02	Los Mochis	MM	-108.998336	25.790603
25.2.03	Mazatlán	MM	-106.425022	23.199808
26.1.01	Guaymas	MA	-110.888916	27.924353
26.2.02	Ciudad Obregón	MM	-109.927969	27.495958
26.2.03	Hermosillo	MM	-110.958971	29.075178
26.2.04	Nogales	MM	-110.943636	31.331695
27.1.01	Villahermosa	MA	-92.919519	17.987428
28.1.01	Reynosa	MA	-98.277901	26.092897
28.1.02	Tampico	MA	-97.857636	22.216049
28.2.03	Ciudad Victoria	MM	-99.144765	23.731777
28.2.04	Matamoros	MM	-97.503900	25.880135
28.2.05	Nuevo Laredo	MM	-99.507047	27.493525
29.1.01	Tlaxcala-Apizaco	MA	-98.140509	19.415234
30.1.01	Coatzacoalcos	MA	-94.411101	18.146450
30.1.02	Córdoba	MA	-96.934795	18.894092
30.1.03	Minatitlán	MA	-94.542226	17.982831
30.1.04	Orizaba	MA	-97.104943	18.848813
30.1.05	Poza Rica	MA	-97.444009	20.553802
30.1.06	Veracruz	MA	-96.138507	19.200264
30.1.07	Xalapa	MA	-96.923825	19.526760
31.1.01	Mérida	MA	-89.623728	20.967091
32.1.01	Zacatecas-Guadalupe	MA	-102.572129	22.776133

Supplementary Note 2: Distance to the city centre, remoteness, and alternative scaling exponents

Cities vary considerably in size, ranging from a few thousand to many millions of inhabitants. Thus, when comparing cities, it is crucial to consider their size. For example, more populous cities naturally occupy a larger surface, so distances tend to be longer. When comparing different cities, we scale distances based on remoteness. That metric has been used to obtain a more comparable unit for distances in cities[3]. Formally, the remoteness for some location in city i is defined as

$$r = 1000\frac{s}{\sqrt{P_i}}, \tag{1}$$

where s is the radial distance to the centre of i and P_i is its population. The preceding 1000 was added to obtain one- or two-digit numbers (with the corresponding decimals), and is interpreted (below) as the square root of the population of the scaled city.

The rationale for defining remoteness as the distance to the city centre divided by the square root of the population is as follows. Imagine a city with P inhabitants. If each person occupies a fixed surface in that city (say μ m^2), then the whole urban area occupies a surface of μP m^2. If the population is arranged in a circular and compact manner (like a honeycomb), then they would be placed in a circle with radius $r = \sqrt{\mu P/\pi} = \alpha\sqrt{P}$, for some value of $\alpha = \sqrt{\mu/\pi}$, which does not depend on the population. In that idealised city, we can express its radius as a function of the square root of the urban population. To compare distances to the city centre across idealised cities with different populations, we normalise by the city radius, or, equivalently, divide the distance by a factor proportional to the square root of the population. The idealised city enables us to compare more urban indicators, for example, the distance between two randomly selected locations in each town[4].

This idealised city is not necessarily observed. Larger cities could get denser as they grow. In that case, instead of observing that each person occupies μ m^2 across all cities, the number may be smaller for larger populations. As a result, the distance-based remoteness, adjusted by the square root of population, could remain incomparable across cities of different sizes. However, it is unclear if large cities are denser. For example, in Spain, Germany, and Italy, larger cities have a similar density to small ones[5]. Outside Europe, there is also evidence that large cities are not denser than small ones. For example, by examining nearly 6,000 urban areas in Africa, it was found that people experience longer distances than the model with idealised cities (round and compact)[4]. Crucially, even if the constant-μ assumption does not hold exactly across all city sizes, $\beta = 0.5$ remains the uniquely appropriate choice for the cross-sectional density comparison performed here since it is the only one for which the rescaling factor $(P_o/P_i)^{1-2\beta}$ is one, leaving population density values completely unchanged. Any $\beta \neq 0.5$ would introduce a size-dependent distortion into density values, making cities of different populations incomparable in terms of their actual densities (see equation (3) and discussion below).

We have used the square root of the population to scale distances in cities, but we could also consider an alternative scaling model based on area. Consider the area of the city as $A = \alpha P^\gamma$, for some value of γ. If large cities had a higher density, then $\gamma < 1$, but if large cities are equally dense, then we would observe that $\gamma \approx 1$. Then, instead of scaling distances by the square root of the population, we could consider distances divided by P^β with $\beta = \gamma/2$. For example, if we used a value of $\gamma = 5/6$, as predicted by Urban Scaling Theory[6] and observed in France[5] and the US[7], we would obtain the following. Consider, for example, the cases of Mexico City (with approximately 22 million people) and Merida (with nearly 1 million people). A remoteness of $r = 3$, identifying the central part of the city, is 14 km away from the city centre in Mexico City and only 3 km away from the centre of Merida. Those locations are considered equally remote based on the square root of their populations. If we used $\gamma = 5/6$, then the central part of Mexico City would correspond to a disc with a radius of 3.8 km in Merida (Figure 1). Therefore, using this value for the scaling surface coefficient would only slightly compress the cumulative curves.

While it would not alter the loss of urban population near the centre of all cities in Mexico, density values would scale as $1 - 2\beta = 1/6$ (equation (3)), distorting our comparison of population densities and making them less interpretable. Moreover, this exponent was obtained for the scaling of the actual urbanised area within metropolitan Areas, not the total extent of the city, taken as the disk that inscribes the city, used when performing radial analysis. Additionally, the temporal analysis of urban expansion (meaning the estimation of the urban expansion factor Φ_{ij} and finding that all cities expanded their radial distribution while losing central density) is conducted independently for each city using actual physical distances s. Remoteness is used only for cross-city comparisons of density profiles and for defining zone boundaries. The observation that $\Delta\sigma < 0$ near the centre of every city follows directly from each city's raw radial data and is entirely independent of the cross-sectional scaling assumption.

It is possible to utilise remoteness and alternative scaling exponents within our scaling framework. By dropping time dependence, we can apply the scaling described by equations (2) and (3) of the main text to a transversal comparison of cities. These results in the following equations for the urban expansion factor

$$\Phi_{io} = \left(\frac{P_o}{P_i}\right)^{\beta}, \tag{2}$$

and population density

$$\sigma_o(s) = \left(\frac{P_o}{P_i}\right)^{1-2\beta} \sigma_i\left(\frac{s}{\Phi_{io}}\right), \tag{3}$$

where o refers to the scaled version of city i, with population P_o. Remoteness is obtained by setting $\beta = 0.5$ and $P_o = 1 \times 10^6$. Note that for $\beta = 0.5$, the factor $\left(\frac{P_o}{P_i}\right)^{1-2\beta} = 1$, and population density values are preserved after scaling. By using remoteness, we scale urban areas to a similar city with a

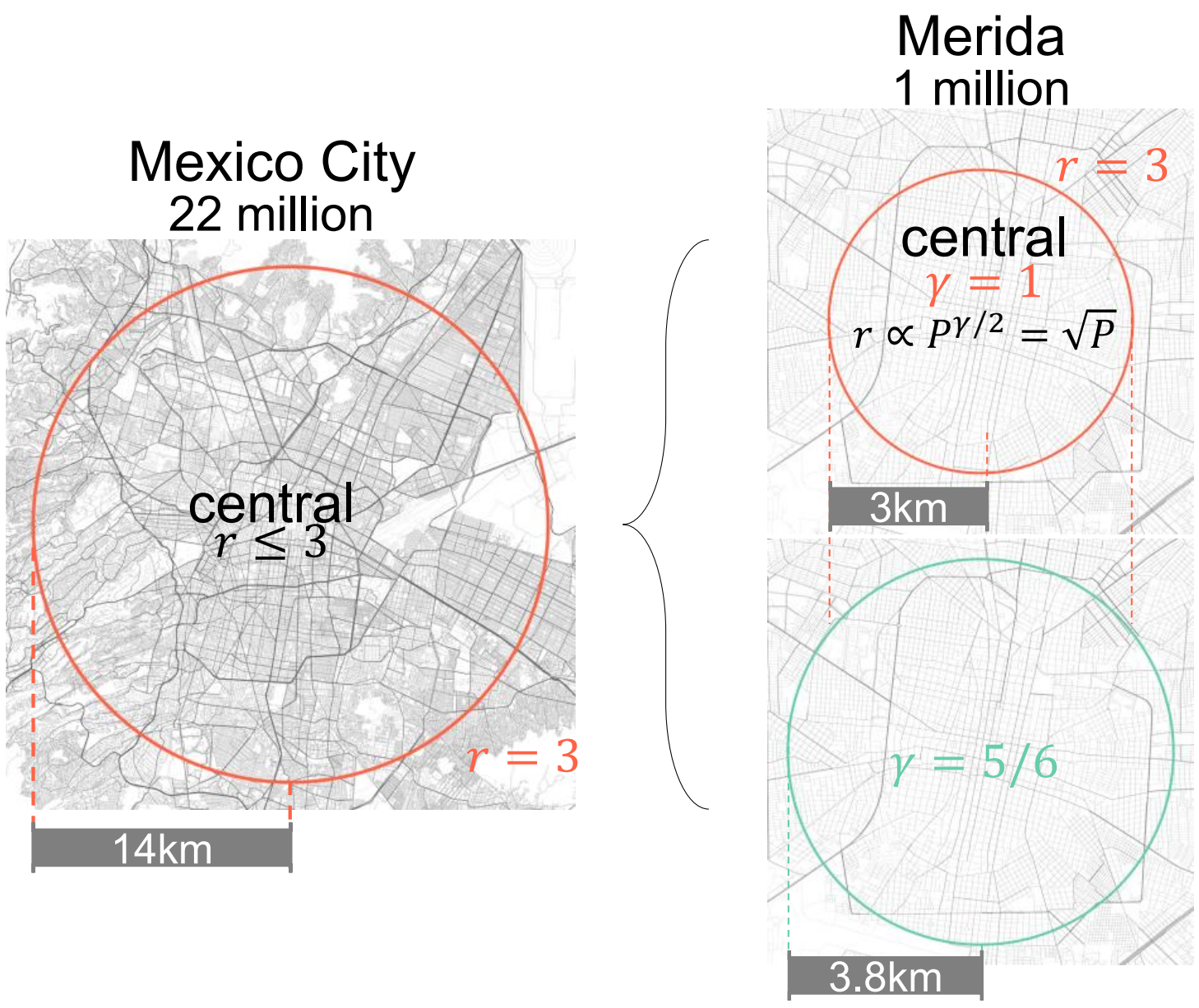

Supplementary Figure 1: Area-based remoteness scaling: Mexico City versus Merida. The central part of Mexico City, identified as the region with a remoteness smaller than three units (left), corresponds to a smaller disc in Merida (right). The core difference between the two cities is that Mexico City has over 22 million inhabitants, while Merida has roughly 1 million. Applying a different definition of remoteness (based on area, with the corresponding pre-factor to obtain the same region in Mexico City) has a minor impact on the definition of Merida's central part.

population of 1 million and an equivalent population density. Since population density is preserved, we can compare population densities of the rescaled cities (as we do in Results of the main text). Recently, a scaling exponent of $\beta = 1/3$ was proposed in a study using radial analysis on a global sample of cities[8]. There, a special case of equation (3) was suggested, deemed homothetic scaling, of the form

$$\sigma_o(s) = \left(\frac{P_o}{P_i}\right)^{\beta} \sigma_i \left(\left(\frac{P_i}{P_o}\right)^{\beta} s \right), \tag{4}$$

where the exponent that best fitted the sample of cities was $\beta = 1/3$. A possible explanation for this result is that the obtained $\beta = 1/3$ is the only value for β that is compatible with equation (3) (a solution to $1 - 2\beta = \beta$). Any other exponent would result in a transformation that is not a scaling, as radial probability densities ρ of transformed cities would not be normalised (see Methods in main text). Therefore, it is unclear if using the more general model (3) would yield a different exponent. However, using $\beta = 1/3$ would also alter population density values and impede comparisons between cities.

Population redistribution under alternative scaling exponents

While remoteness is adequate for density comparisons under scaling, it is informative to explore how the observed redistribution changes under the alternative scaling exponents. We perform a sensitivity analysis re-classifying each city's population into the four remoteness zones under two alternative values of β: the Urban Scaling Theory prediction $\beta = 0.425$ ($\gamma = 5/6$)[6,7], and the homothetic scaling exponent $\beta = 1/3$ ($\gamma = 2/3$)[8]. For each alternative value, we recompute the total population in the central zone ($r < 3$) and the aggregate loss between 1990 and 2020 (Supplementary Figure 2).

Across all tested exponents, Mexican metropolitan zones continue to show a net population decline in their central zone, confirming that the finding is robust to the choice of parameter values (Supplementary Table 2).

The aggregate loss estimate changes because a smaller β compresses zone boundaries inward for larger cities, shrinking the physical extent of what is classified as central. Yet, even in those different scenarios, the central parts of cities in Mexico lost, at least, 2.2 million inhabitants.

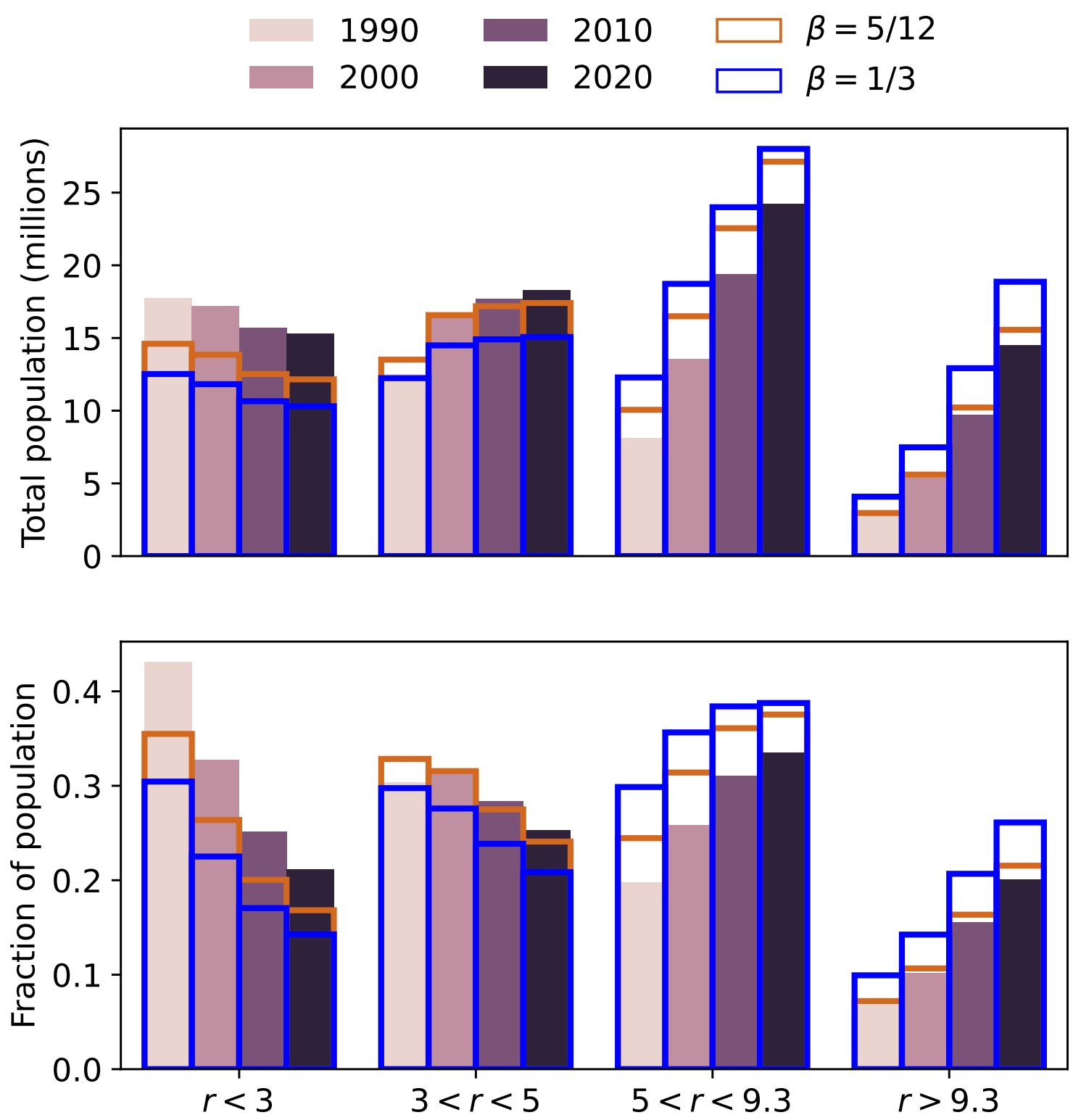

Supplementary Figure 2: Population redistribution under alternative scaling exponents. Population and population fraction aggregated over all metropolises at different remoteness brackets for all census years. Coloured bars correspond to remoteness scaling ($\beta = 0.5$), hollowed bars to UST ($\beta = 5/12$, brown) and homothetic scaling ($\beta = 1/3$, blue).

Supplementary Table 2: Sensitivity of central population loss to the choice of scaling exponent β. "Central loss" is the aggregate population decline in zones classified as central ($r < 3$) between 1990 and 2020 across all 69 metropolitan areas.

Scaling	β	Central loss (millions)
Standard remoteness	0.5	2.5
Urban Scaling Theory	0.425	2.4
Homothetic	1/3	2.2

Supplementary Note 3: A country in evolution

Demographic changes

Over the past three decades, Latin America has undergone major demographic, lifestyle, and epidemiological transitions as the region rapidly urbanised, with these changes contributing to social, health, and spatial inequities[9]. In the latter half of the 20th century, Latin America experienced rapid population growth, characterised by declining mortality rates, high fertility, and a nine-year increase in life expectancy[10]. Concurrently, migration from rural to urban areas led to urbanisation and significant lifestyle changes[11].

Mexico, like other countries in Latin America, has experienced a rapid demographic shift over the past few decades. By 1970, there were 6.5 births per woman, declining to 4.7 by 1980, 3.4 by 1990, and to fewer than two births by 2023[12,13]. This demographic shift implies that the largest cohort in Mexico was born around 1990, and then each successive cohort is smaller than the previous one[13]. Life expectancy at birth has increased from less than 60 years in 1970 to nearly 75 years in 2023[14]. As a result of those two shifts, the country's population continues to expand, but its demographic composition has changed.

In 1990, an average of five people lived in each private housing unit, but by 2020, this figure had fallen to less than 3.6. Smaller households, some formed more frequently by single individuals, and an ageing population, are increasingly prevalent in the country.

These rapid demographic changes also affect the demand for different resources. The country has fewer children and fewer elementary school students, but more people commuting to work. Due to this shift, there is reduced demand for obstetricians and paediatricians but greater demand for caregivers. A similar pattern has emerged regarding mobility, housing, and demand for other goods and services.

Economic changes

Due to many reasons, including the formation of the North American Free Trade Agreement (NAFTA) between Mexico, the USA, and Canada in 1994, an industrialisation process in the North of Mexico, and the flow of remittances (resulting from nearly one million migrants moving from Mexico to the US each year around 2000), the country became wealthier between 1990 and 2020. Using 1990 as the baseline, GDP per capita (adjusted for purchasing power) increased by 36% by 2000, 85% by 2010, and 126% by 2020[15]. Although inequality in the country increased slightly between 1990 and 2000, it has declined since then[16]. Thus, households now have a much higher budget than they did decades ago.

With more money (on average), Mexican families can afford more goods and satisfy their needs, including housing and transport. For example, in Mexico, the number of private vehicles increased from less than 5 million in 1990 to more than 40 million today[17]. Part of that increase in car ownership is due to higher household income.

Supplementary Note 4: Cities as a self-organising object

A self-organising object can autonomously arrange its internal structure or behaviour without external control. It uses local interactions among its parts to create an overall organised pattern or function, often adapting dynamically to changing conditions. Cities can also be regarded as self-organising systems[18]. Through countless decisions (for example, where people build, work, trade, and gather), they develop complex patterns, infrastructure, and social networks without a central planner controlling every detail.

For example, segregation has been observed to emerge as a complex process, driven often by housing relocation within a city[19]. Although segregation is often perceived as a poverty-related phenomenon, the rich may voluntarily segregate themselves to avoid interactions with the rest of the population[20].

In the case of the abandonment of the central parts of cities in Mexico, it should also be considered a process resulting from self-organisation. At some point before 1990, people created a city with some layout (including density and sprawl). For some reason, after 1990, people are moving to more peripheral locations. But this results from millions of collective housing relocations (uncoordinated and asynchronous), rather than from a single policy or intervention in the country.

One of those elements is the rapid shift towards car mobility. In Mexico, there were fewer than 5 million private vehicles in 1990, but there are over 40 million cars today[17]. Thus, cities in the country have faced pressure to create space not only for a large number of people but also for an even larger number of cars.

Supplementary Note 5: Different scenarios and implications at the city level

Between two consecutive periods, a city belongs to one of six regions. The scenario depends on two elements: its population growth $P(t_j)/P(t_i)$ and its urban expansion factor Φ_{ij}. Each scenario and its implications are analysed below.

A. Density loss, and urban expansion, with population growth. Most cities in Mexico fall into this scenario (189 of 207 observations). This scenario signals poor housing policies that promote suburbanisation. There may also be informal developments to satisfy the rapidly increasing housing needs. Many factors combine to reduce urban density, including housing and transport policies. For example, car dependency requires vast amounts of infrastructure (for moving and parking), which in turn has promoted sprawl[21]. This type of urban growth further contributes to poor public transport infrastructure, which, in the long run, further encourages car dependency[22].

B. Density gain, and urban expansion, with population growth. This is, perhaps, an expected scenario for a city with an expanding population. As the city's population grows, it must increase its housing capacity, but the process is accompanied by densification, perhaps through infill or vertical development[23].

C. Density gain, and urban compression, with population growth. In this region, cities become smaller despite increasing their population. This scenario is rarely observed, but it would suggest major urban redesigns (to reduce its footprint) accompanied by infill or vertical expansion[23].

D. Density gain, and urban compression, with population loss. Although no city was in this region, they would be observed if they increase their density by contracting faster than they lose population. This is perhaps a desirable scenario for cities undergoing demographic contraction. Bulgaria, for example, went from nearly nine million inhabitants in 1990 to less than seven million, accompanied by rapid population ageing[24]. Cities in regions with rapid ageing and demographic contraction will host fewer inhabitants with fewer transport needs. Thus, urban compression will help reduce travel distance and energy use.

E. Density loss, and urban compression, with population loss. This happens if a city loses density despite contracting because of a faster population decline.

F. Density loss, and urban expansion, with population loss. This is one of the most pervasive scenarios for a city. In Mexico, from 2010 to 2020, Acapulco, Minatitlan, and Poza Rica were in this region. This may be due to a combination of two elements. One, linked to a security crisis. In Mexico, a yearly average of 330,000 Mexicans leave their homes due to the fear of crime[25]. This situation has been particularly intense in cities like Acapulco, usually ranked among the most violent cities worldwide. However, the second element is that urban growth continues, often driven by wealthier families seeking to avoid interactions with others[20]. In the case of Acapulco, for example, Acapulco Diamante is the upscale, modern section, known for its luxury hotels and high-rise towers. That part of the city mainly developed after the 1990s, and far from the city centre, partly as a strategy to divide luxury tourism from the rest. Thus, despite population loss, the city can still have urban expansion and density loss.

Supplementary Note 6: Dynamical regimes of the scaling model

Our model for the urban expansion factor (equation (5) of the main text) captures different dynamics with varying values of α and β. Since different sets of cities (e.g., in other countries) may exhibit different exponents, a discussion of these dynamics is warranted. To simplify the discussion, we assume positive β values (i.e., population growth that drives expansion). There are three different cases to consider depending on the value of β (Supplementary Figure 3).

1. For $\beta = 0.5$, population growth does not affect the scaling of σ, and the model remains parallel to the boundary between density gain and loss regions. If $\alpha > 0$, it is impossible to increase density for any value of population growth. If $\alpha < 0$ (so that the contribution of the temporal term corresponds to a compression, opposite to a stretching), density always increases, but the city enters the region of urban expansion ($\Phi_{ij} > 1$) when growth is large enough (point A), resulting in a combination of expansion and densification. If $\alpha = 0$ and $\beta = 0.5$, the model coincides with the boundary, and we recover remoteness scaling, so density is preserved for any value of population growth.
2. If $\beta < 0.5$, the power law term favours concentration of population into the existing footprint, and density increases if population growth is large enough for this effect to dominate over the temporal expansion associated with α (point C). Despite the growth in central density, it is also true that $\Phi_{ij} > 1$, resulting again in expansion with densification beyond point C.
3. If $\beta > 0.5$, the density only increases if the city loses population below point B, resulting in densification and compression. For any value of population growth, the outcome is expansion with a loss of density.

In all cases, for $\beta > 0$, population growth always leads to expansion since in all three cases we end up in the first quadrant with density loss and population loss (Figure 3). However, moderate population loss may also result in urban expansion (second quadrant) if $\alpha > 0$.

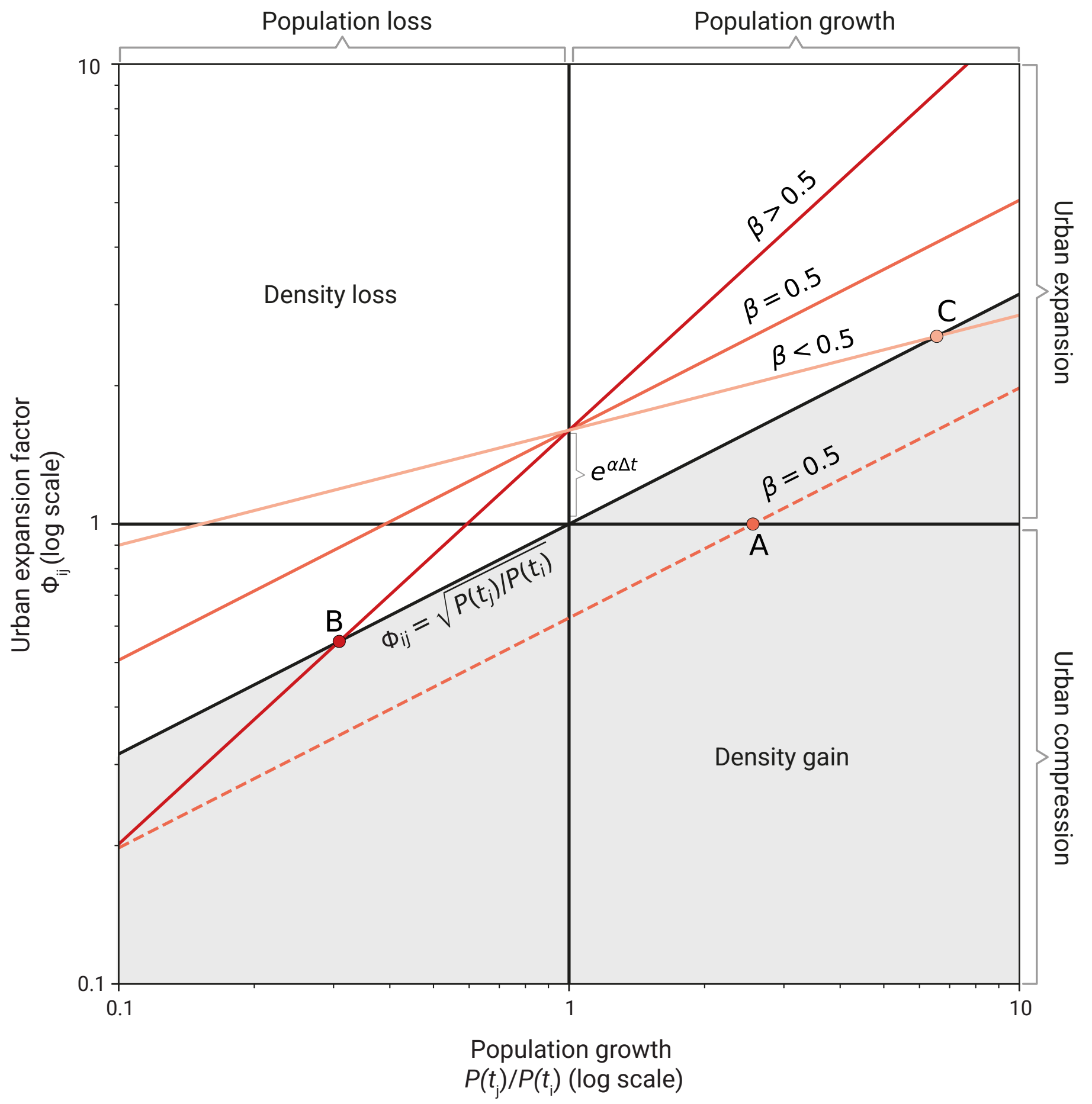

Supplementary Figure 3: Dynamical regimes of the urban-expansion-factor model. Possible model behaviour for Φ_{ij} (equation (5)), main text) for different values of exponent β. Different cases enter and leave the density-gain region at different points. The boundary between regions of density loss and gain is given by $\Phi_{ij} = \sqrt{P(t_j)/P(t_i)}$. The complete set of coloured lines refers to models with $\alpha > 0$, and the dashed lines refer to models with $\alpha < 0$.

The regimes discussed resemble those of sub-diffusion and super-diffusion in physical systems[26]. In this case, $\beta < 0.5$ corresponds to sub-diffusion of the population from the centre of the city (so the density is allowed to increase). In contrast, $\beta > 0.5$ corresponds to super-diffusion (where density at the centre is depleted). Mexican cities have followed super-diffusive dynamics. In the sub-diffusive regime characterised by densification, existing density valleys at time t_i will expand by time t_j as the density function stretches outward from the centre. This process causes the valley's edge to shift, yet its average density increases. In the case of remoteness, where $\beta = 0.5, \alpha = 0$, it aligns with standard diffusion, which preserves the average density within the city.

Supplementary Note 7: Error estimation for global population grids

To assess the feasibility of utilising global population products for analysing depopulation trends in the central areas of Mexican metropolitan zones, we performed a validation exercise for two widely cited global population grids: WorldPop[27]) and Global Human Settlement Population (GHS-POP)[28], both with a resolution of 100 m. Both use a dasymetric mapping approach to disaggregate coarse population counts onto a finer grid using auxiliary variables. WorldPop employs a random forest model with a large set of covariates[29], while GHS-POP disaggregates population counts proportionally to built-up volume[30]. Of these two datasets, only GHS-POP covers the 30-year period of our study, and was, thus, the only

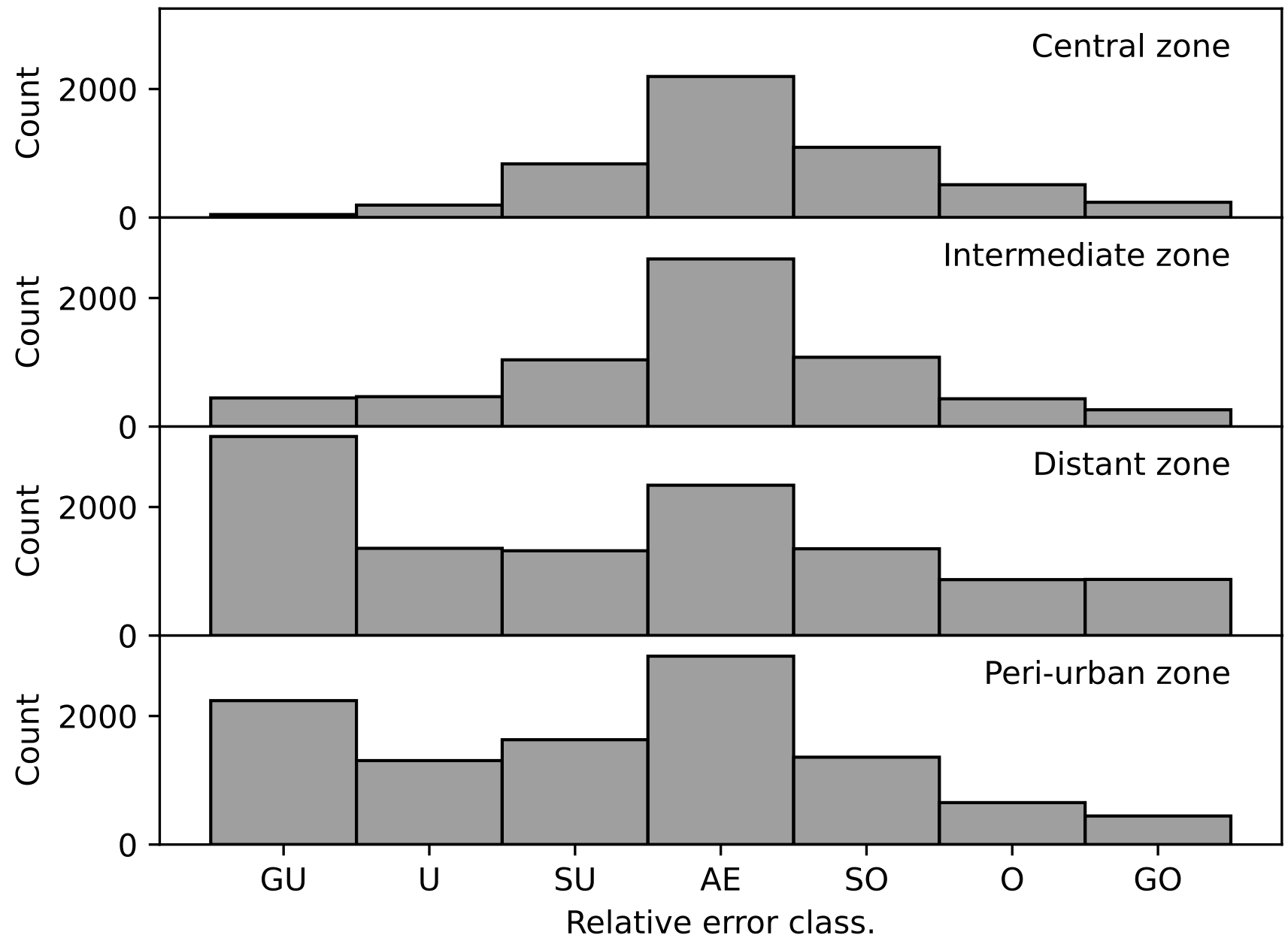

Supplementary Figure 4: GHS-POP relative error by remoteness bracket. Distributions for the relative error η for census tracts at each remoteness bracket. Each tract is classified according to the scheme in Supplementary Table 3.

one considered as an alternative to our custom population grid.

While global grids offer spatially disaggregated data that facilitates the analysis of arbitrary administrative units, it is imperative to establish their accuracy relative to ground-truth official statistics before applying them to granular intra-urban analyses. For this analysis, we utilised census tracts (AGEBs) as the fundamental unit of comparison. To quantify the discrepancy between the grid estimates and the official census counts at this granular level, we aggregated GHS-POP counts into census track geometries and calculated the error $\epsilon = P_{\text{GHS}} - P_{\text{census}}$ and the relative error $\eta = \epsilon / P_{\text{census}}$ for each census track in the 69 Mexican Metropolitan Zones. The mean error is $\langle \epsilon \rangle = -171.85$, showing that GHS-POP tends to underestimate the population. The mean absolute error and the mean relative absolute error are $\langle |\epsilon| \rangle = 472.31$ and $\langle |\eta| \rangle = 0.54$. These values are relatively high, with differences of about 50% in average across census tracts.

Supplementary Table 3: The classification scheme for relative error η. Originally defined in[31].

Value range	Category	Abbreviation
$-\infty$ to -0.5	Greatly underestimated	GU
-0.5 to -0.25	Underestimated	U
-0.25 to -0.1	Slightly underestimated	SU
-0.1 to 0.1	Accurately estimated	AE
0.1 to 0.25	Slightly overestimated	SO
0.25 to 0.5	Overestimated	O
0.5 to ∞	Greatly overestimated	GO

To further understand the limitations of GHS-POP, we investigated whether deviations depend on the distance from the metropolitan centre. Intra-urban analysis requires not only aggregate accuracy but also spatial consistency; therefore, it is critical to determine if grid errors are uniformly distributed or structurally biased towards specific urban morphologies. Each tract was classified into one of the four previously used remoteness brackets (central, intermediate, distant, or peri-urban) based on its centroid. We computed the relative error distribution for each remoteness bracket based on a classification scheme defined in a recent study[31] (Supplementary Table 3). The distribution of relative absolute errors

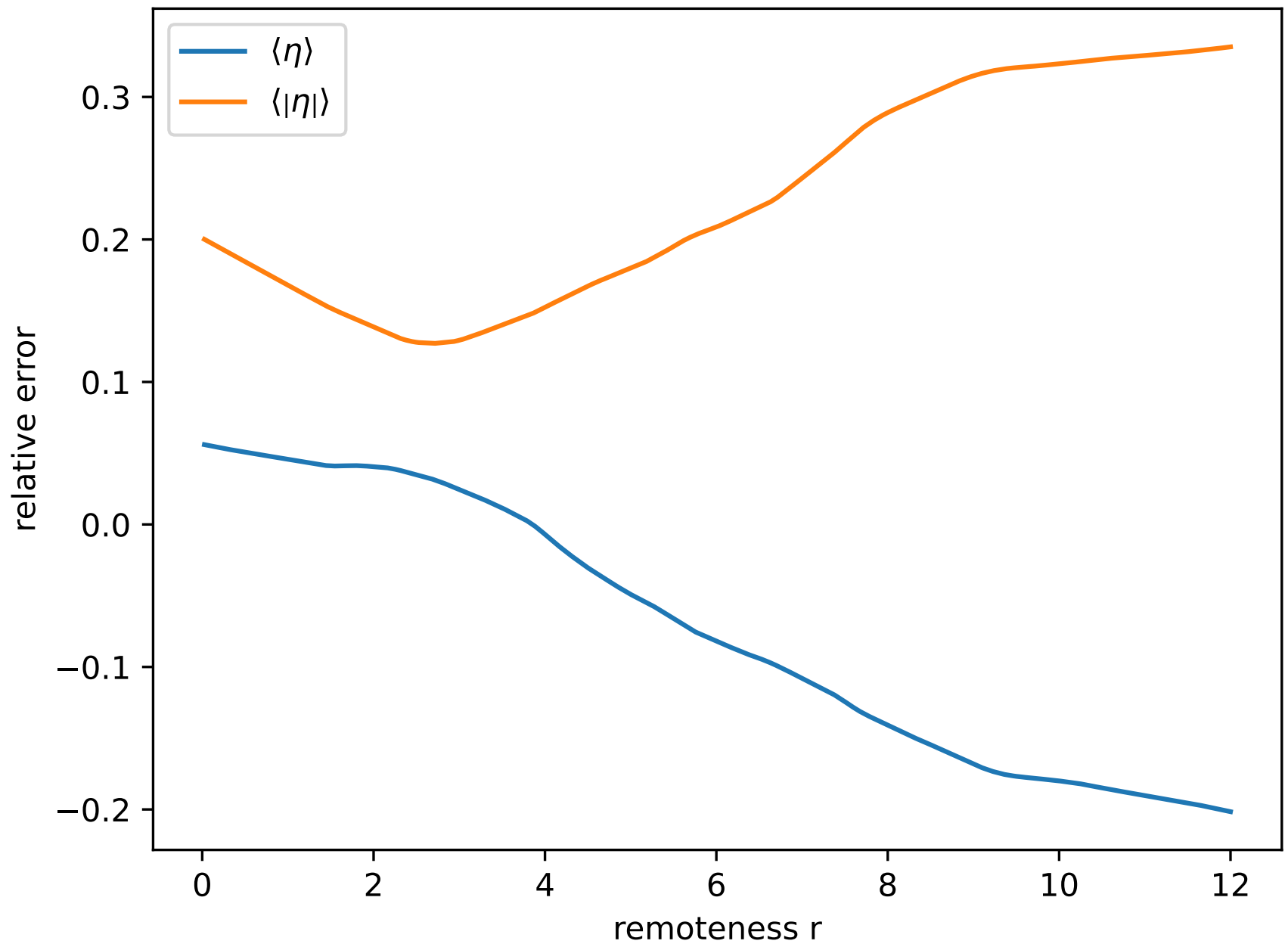

Supplementary Figure 5: GHS-POP error versus remoteness. LOWESS smoothed average for the relative error η and the absolute relative error $|\eta|$ versus remoteness r.

is different for each remoteness bracket (Supplementary Figure 4). GHS-POP tends to overestimate population in central zones, while clearly underestimating population in the distant and peri-urban areas, where errors are also larger. In particular, the large occurrence of the "Greatly underestimated" class in distant and peri-urban zones is worrisome. This dependence of the error on the distance to the centre is confirmed using a LOWESS (Locally Weighted Scatterplot Smoothing)[32] smoothing average for η and $|\eta|$ against remoteness (Supplementary Figure 5). GHS-POP exhibits its lowest errors in central tracts and its highest errors in distant tracts. Absolute relative error $|\eta|$ takes its minimum around $r = 3$, but almost doubles the value of the centre at $r = 10$. Relative error η is positive in central areas (overestimation) and becomes negative as we move away from the centre (underestimation). This suggests that global downscaling algorithms struggle to accurately capture population distributions in peri-urban and low-density transition zones, likely due to the difficulty of distinguishing complex settlement patterns in satellite imagery. This systematic issue has also been identified for detection across rural areas[33].

In summary, population count errors for GHS-POP in Mexican metropolitan areas are substantial and display spatial patterns, contradicting the assumption of random error. These systematic variations may bias analyses of population dynamics and reinforce the conclusion that global grids lack the necessary spatial stability to model nuanced demographic shifts.

Supplementary Note 8: Data for all metropolitan zones

We include here visualisations for all 69 metropolitan zones we studied. In a page per city:

- Map of population difference between 1990 and 2020.
- Quantile-quantile plot with the three censal intervals and the fitted slope, which is equal to the urban expansion factor Φ_{ij}. The region over which scaling is observed appears linear in the Q-Q plot.
- The radial population distribution $\rho(r)$ as a function of remoteness in 2020. Observed (grey lines) and scaled (blue lines).
- The radial population density $\sigma(r)$ as a function of remoteness in 2020. Observed (grey lines) and scaled (blue lines).

- The average population density $\bar{\sigma}(r)$ as a function of remoteness in 2020. Observed (grey lines) and scaled (blue lines).
- Table with population growth $P(t_j)/P(t_i)$, estimated urban expansion factors Φ_{ij}, and the interquartile range from the Theil-Sen estimation for Φ_{ij} for all six observed intervals between 1990 and 2020. The interquartile range of the Theil-Sen slopes is a usual measure of uncertainty for the slope estimate, similar to confidence intervals in linear regression.

Aguascalientes, 01.1.01

a)

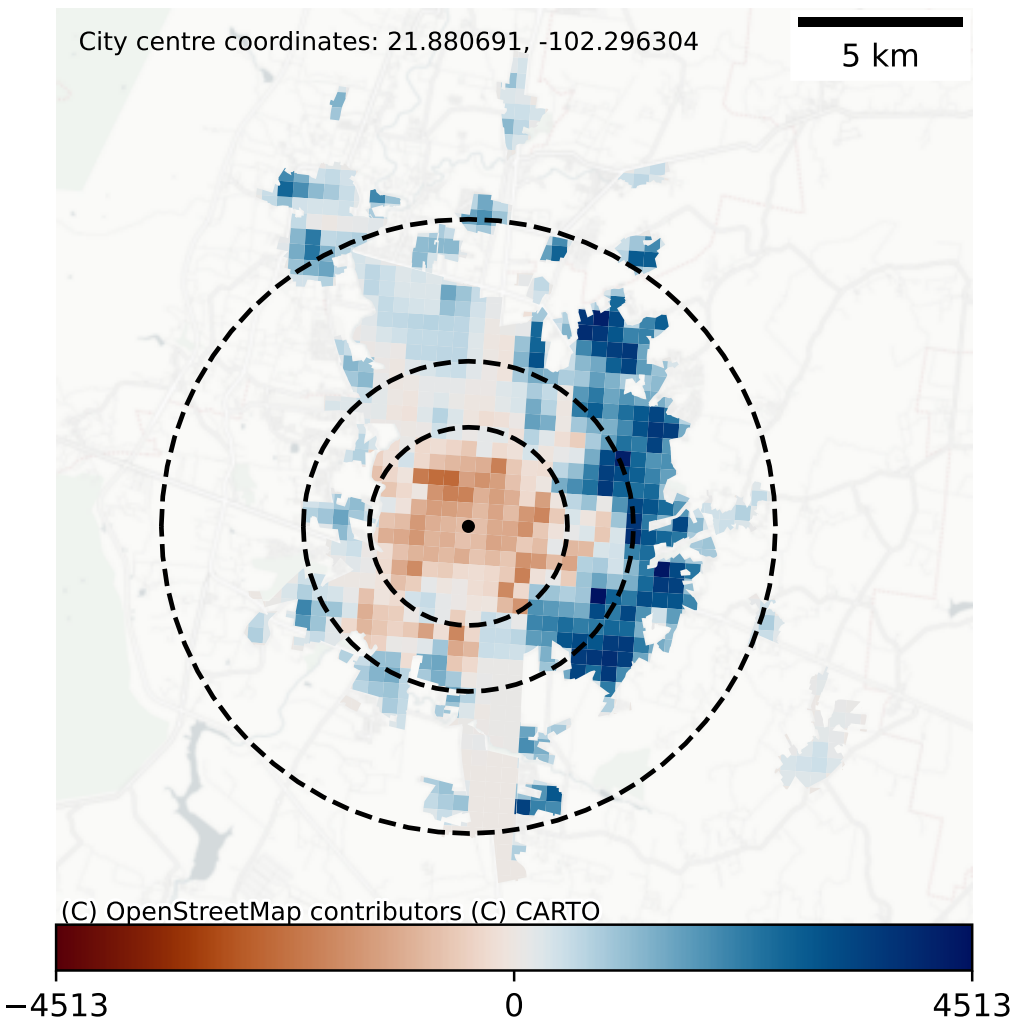

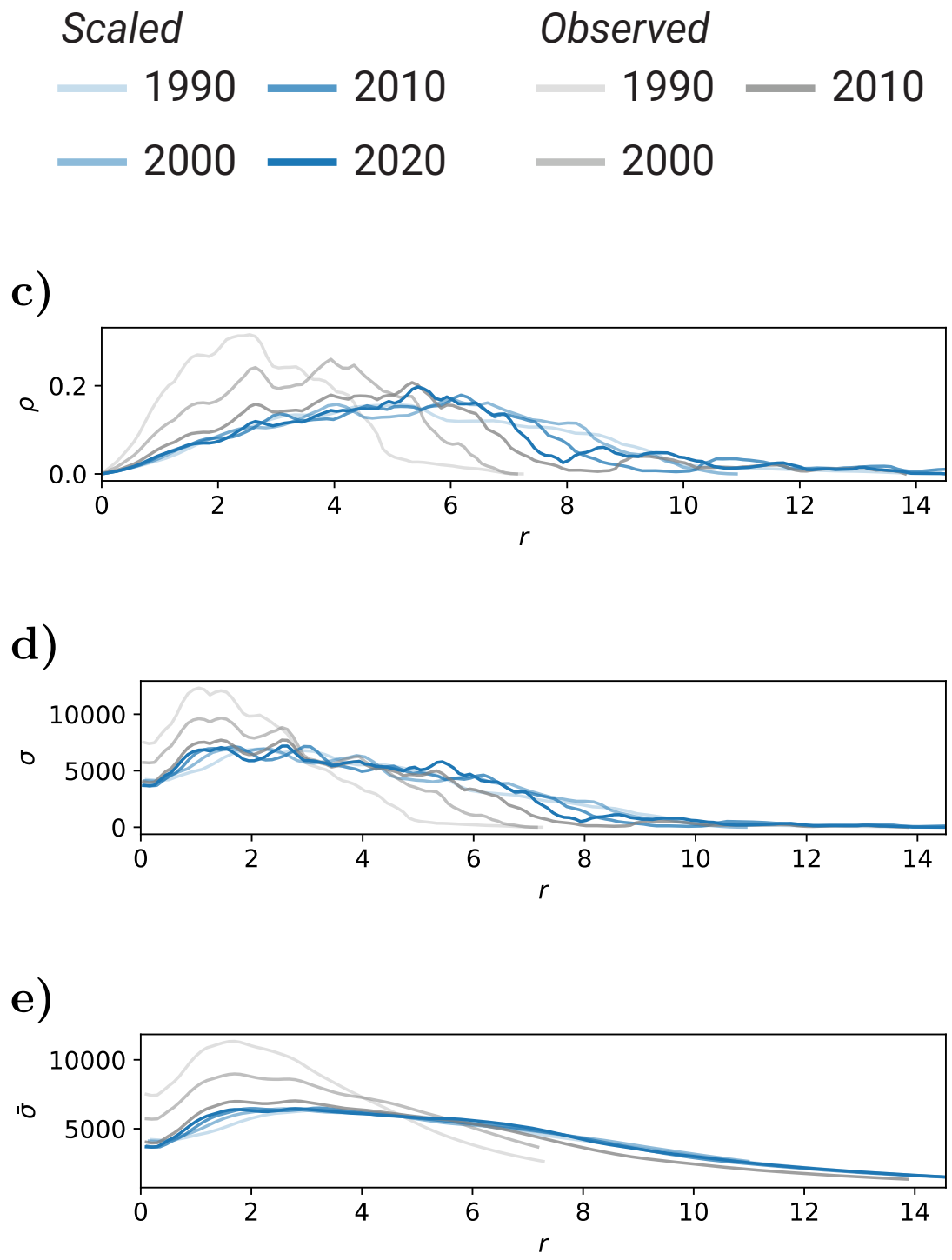

b)

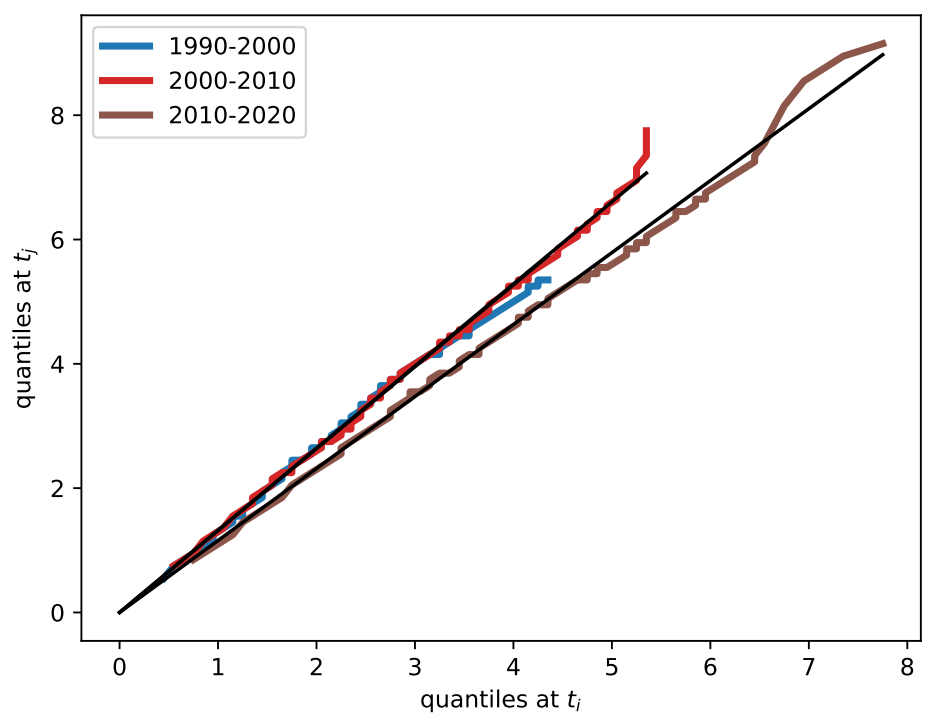

f)

Period (t_i-t_j)	$\frac{P(t_j)}{P(t_i)}$	Φ_{ij}	IQR
1990-2000	1.36	1.32	(1.29, 1.34)
2000-2010	1.35	1.32	(1.30, 1.34)
2010-2020	1.24	1.16	(1.15, 1.17)
1990-2010	1.84	1.74	(1.70, 1.77)
2000-2020	1.68	1.53	(1.51, 1.55)
1990-2020	2.28	2.02	(1.96, 2.04)

Supplementary Figure 6: Supplementary data for the metropolitan zone of Aguascalientes with code 01.1.01. Remoteness values are those of 2020. a) Population difference by grid cell (2020–1990); city centres are denoted as black dots. b) Quantile-quantile plots for the radial population distributions $\rho(s,t_i)$ and $\rho(s,t_j)$ (coloured curves); urban expansion factors Φ_{ij} from t_i to t_j are the estimated slopes (black lines). c) Radial population distribution $\rho(r)$ at remoteness distance r from the city centre. d) Radial population density $\sigma(r)$ at remoteness distance r from the city centre. e) Average population density $\bar{\sigma}(r)$ within disks of remoteness r with the same centre as the city. f) Urban expansion factors and their inter quartile range from the Theil-Sen estimation.

Tijuana, 02.1.01

a)

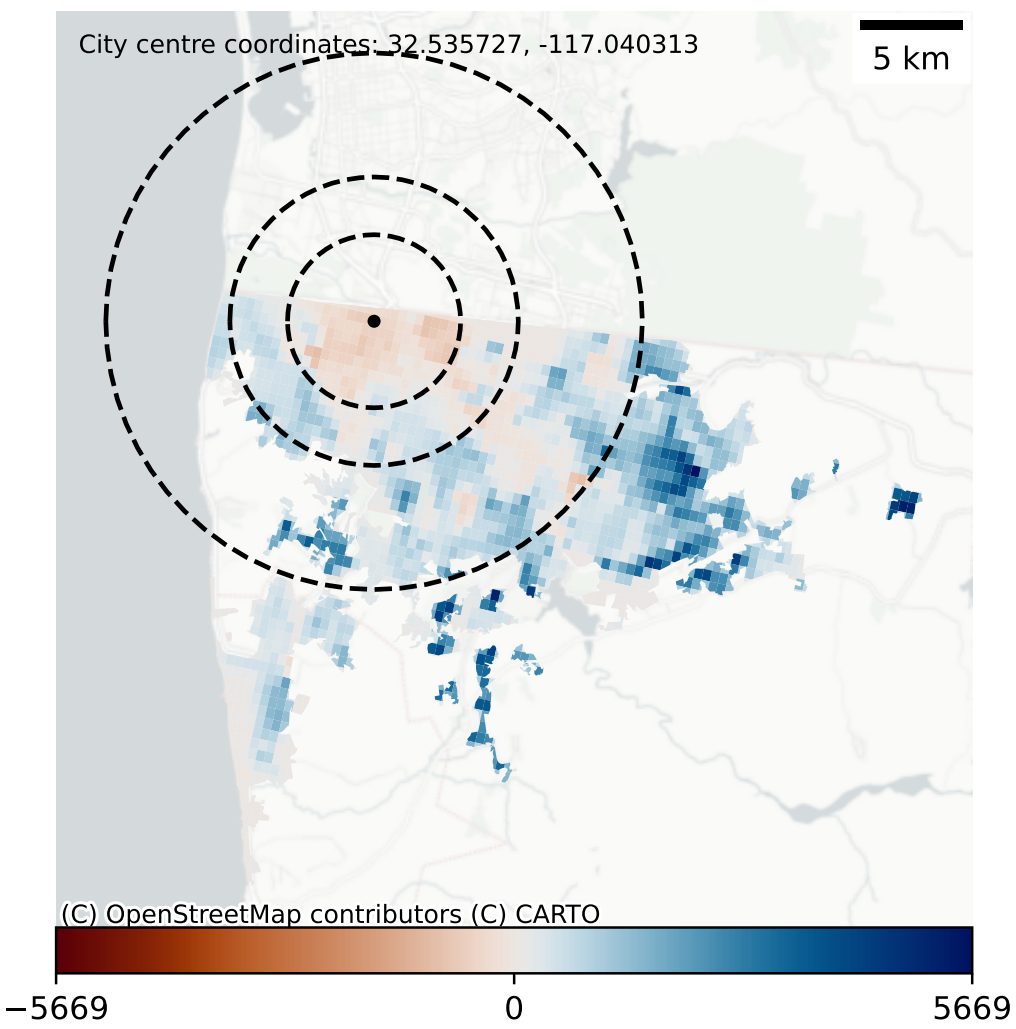

b)

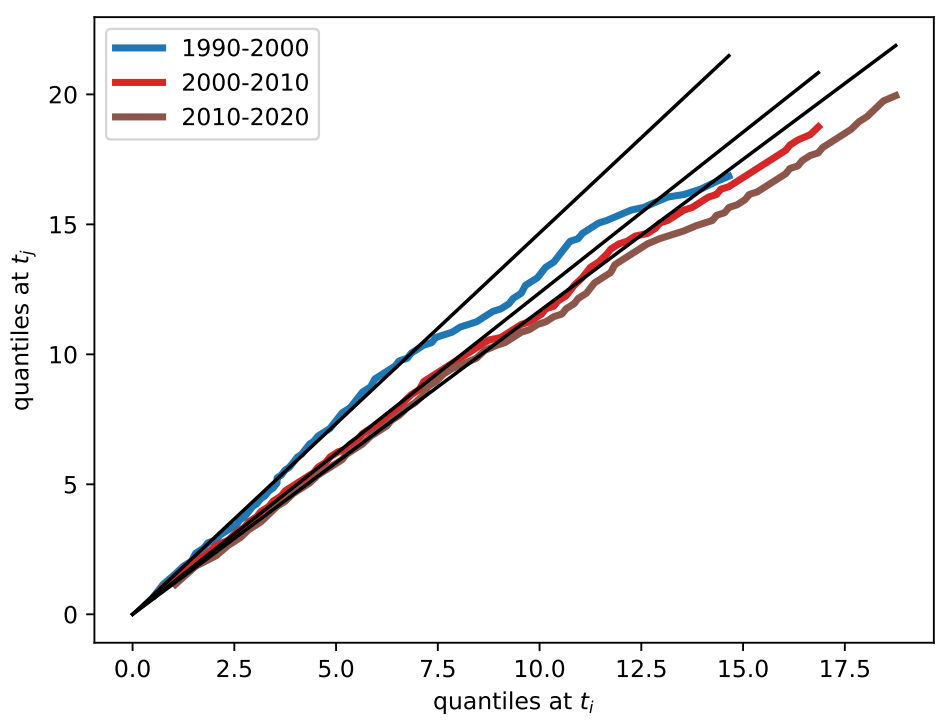

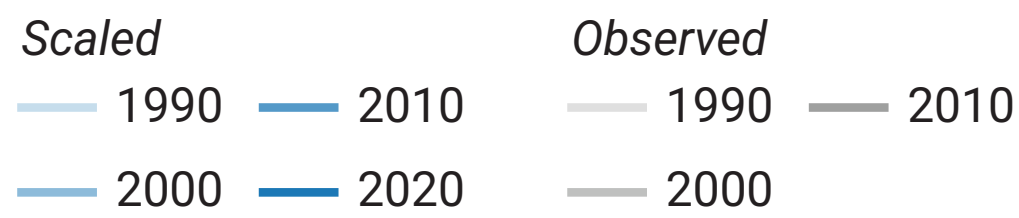

c)

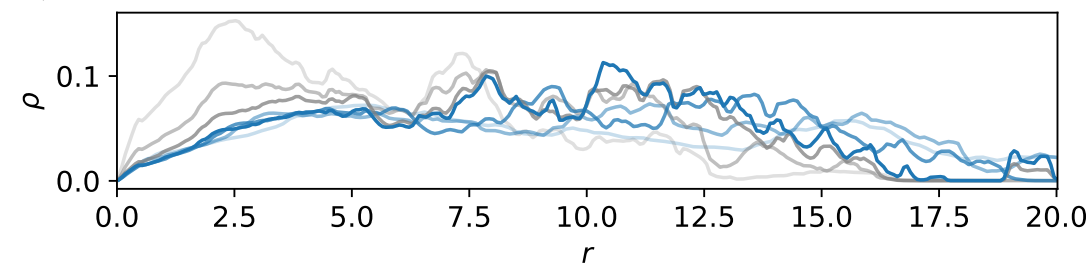

d)

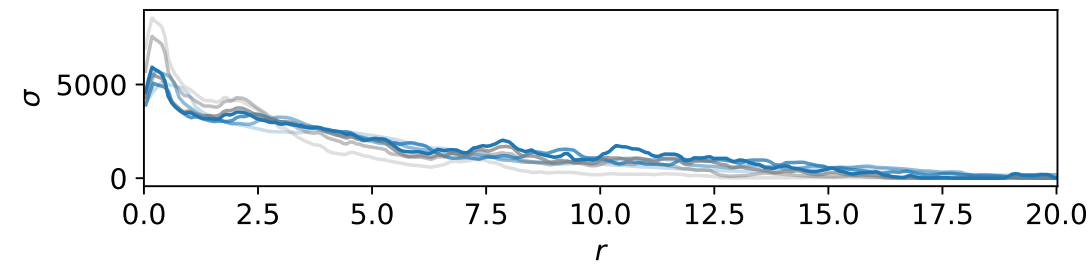

e)

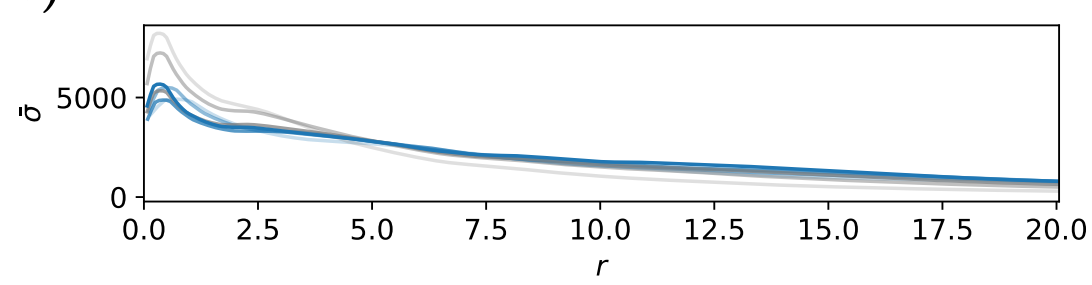

f)

Period (t_i-t_j)	$\frac{P(t_j)}{P(t_i)}$	Φ_{ij}	IQR
1990-2000	1.69	1.47	(1.40, 1.48)
2000-2010	1.27	1.24	(1.22, 1.25)
2010-2020	1.24	1.17	(1.16, 1.18)
1990-2010	2.15	1.80	(1.74, 1.83)
2000-2020	1.58	1.44	(1.43, 1.45)
1990-2020	2.67	2.09	(2.02, 2.14)

Supplementary Figure 7: Supplementary data for the metropolitan zone of Tijuana with code 02.1.01. Remoteness values are those of 2020. a) Population difference by grid cell (2020–1990); city centres are denoted as black dots. b) Quantile-quantile plots for the radial population distributions $\rho(s, t_i)$ and $\rho(s, t_j)$ (coloured curves); urban expansion factors Φ_{ij} from t_i to t_j are the estimated slopes (black lines). c) Radial population distribution $\rho(r)$ at remoteness distance r from the city centre. d) Radial population density $\sigma(r)$ at remoteness distance r from the city centre. e) Average population density $\bar{\sigma}(r)$ within disks of remoteness r with the same centre as the city. f) Urban expansion factors and their inter quartile range from the Theil-Sen estimation.

Ensenada, 02.2.02

a)

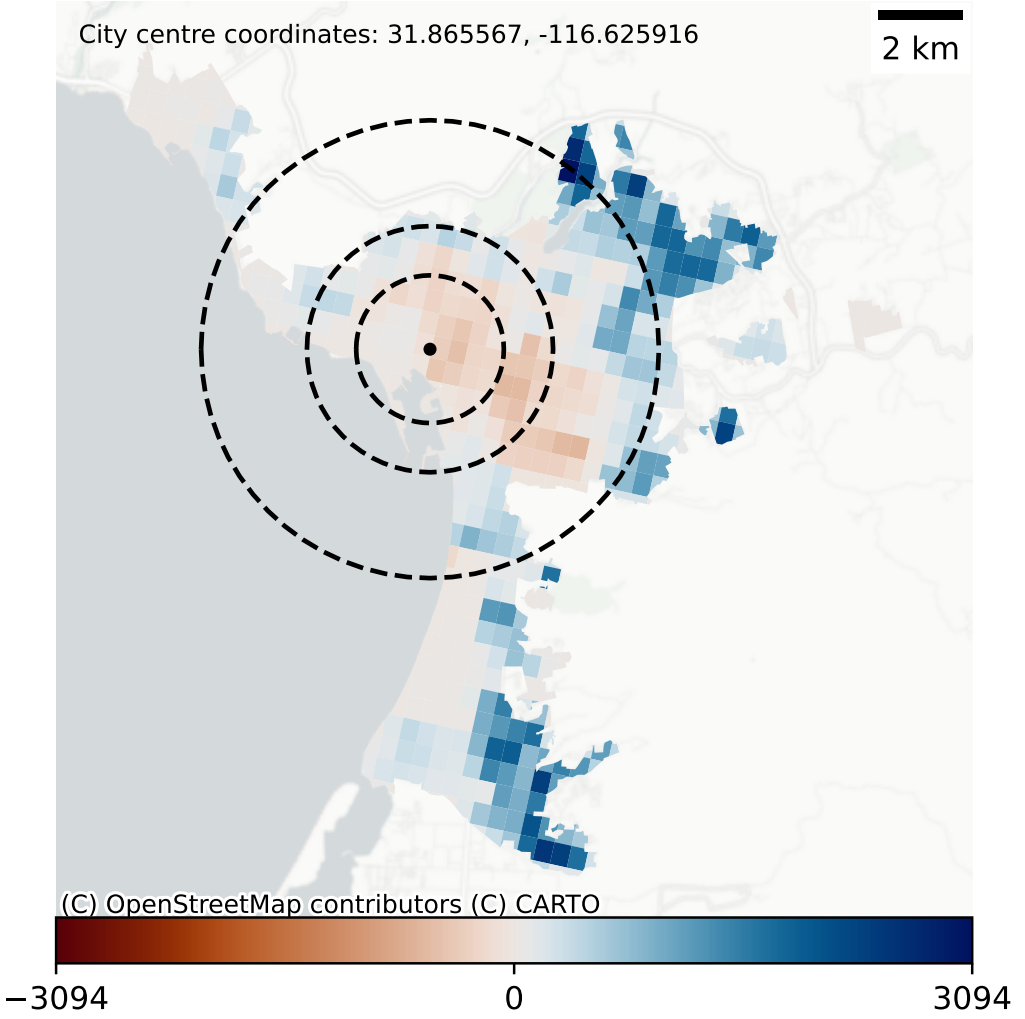

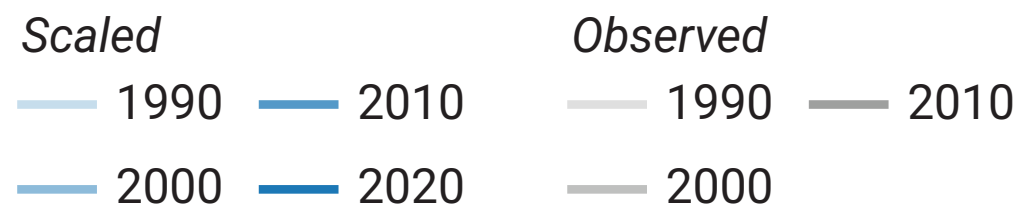

c)

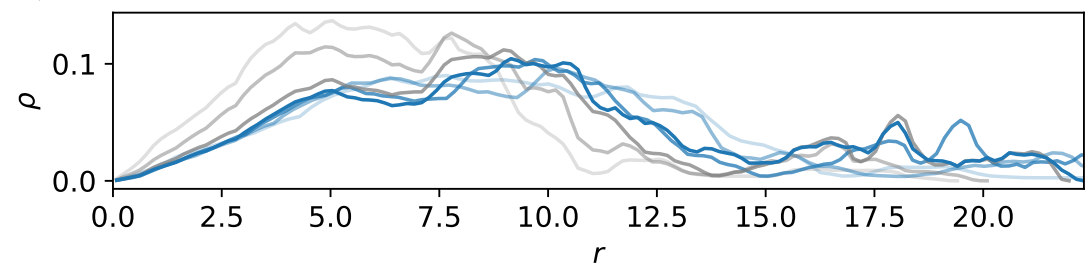

d)

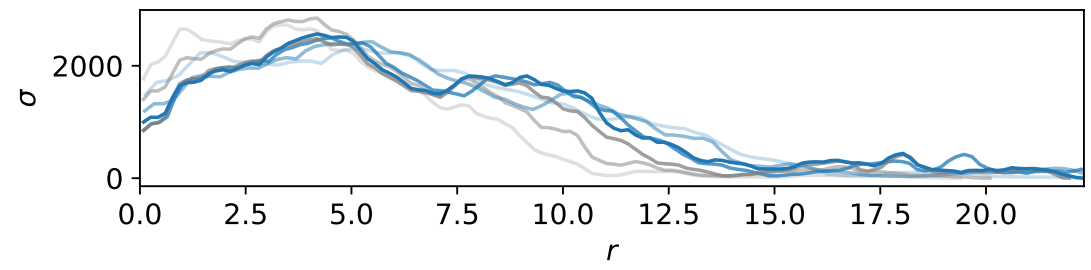

e)

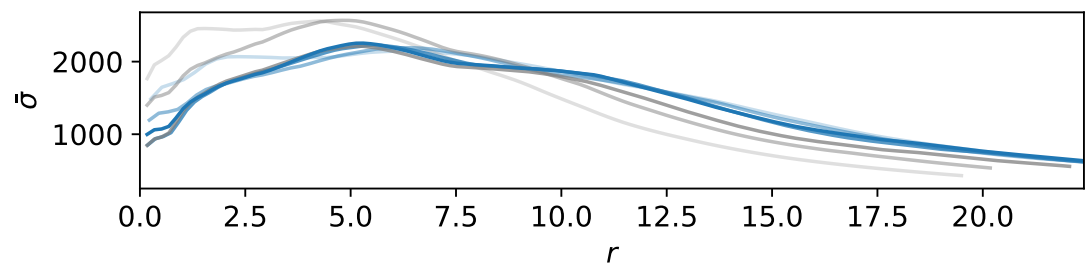

b)

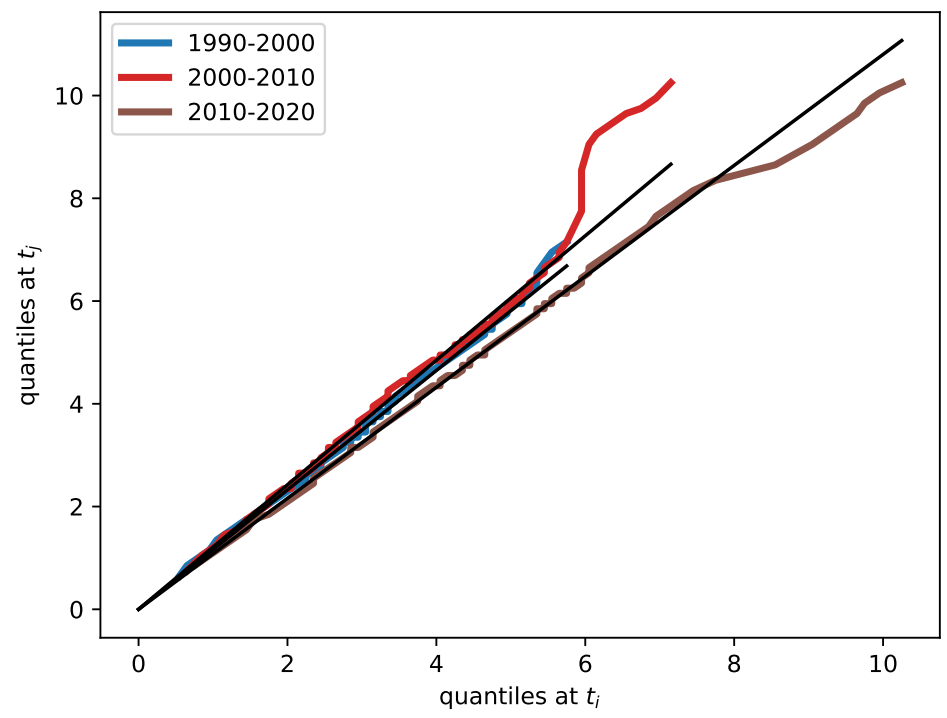

f)

Period (t_i-t_j)	$\frac{P(t_j)}{P(t_i)}$	Φ_{ij}	IQR
1990-2000	1.33	1.16	(1.15, 1.18)
2000-2010	1.24	1.21	(1.19, 1.23)
2010-2020	1.17	1.08	(1.07, 1.09)
1990-2010	1.66	1.41	(1.38, 1.44)
2000-2020	1.46	1.30	(1.28, 1.34)
1990-2020	1.95	1.53	(1.49, 1.56)

Supplementary Figure 8: Supplementary data for the metropolitan zone of Ensenada with code 02.2.02. Remoteness values are those of 2020. a) Population difference by grid cell (2020–1990); city centres are denoted as black dots. b) Quantile-quantile plots for the radial population distributions $\rho(s,t_i)$ and $\rho(s,t_j)$ (coloured curves); urban expansion factors Φ_{ij} from t_i to t_j are the estimated slopes (black lines). c) Radial population distribution $\rho(r)$ at remoteness distance r from the city centre. d) Radial population density $\sigma(r)$ at remoteness distance r from the city centre. e) Average population density $\bar{\sigma}(r)$ within disks of remoteness r with the same centre as the city. f) Urban expansion factors and their inter quartile range from the Theil-Sen estimation.

Mexicali, 02.2.03

a)

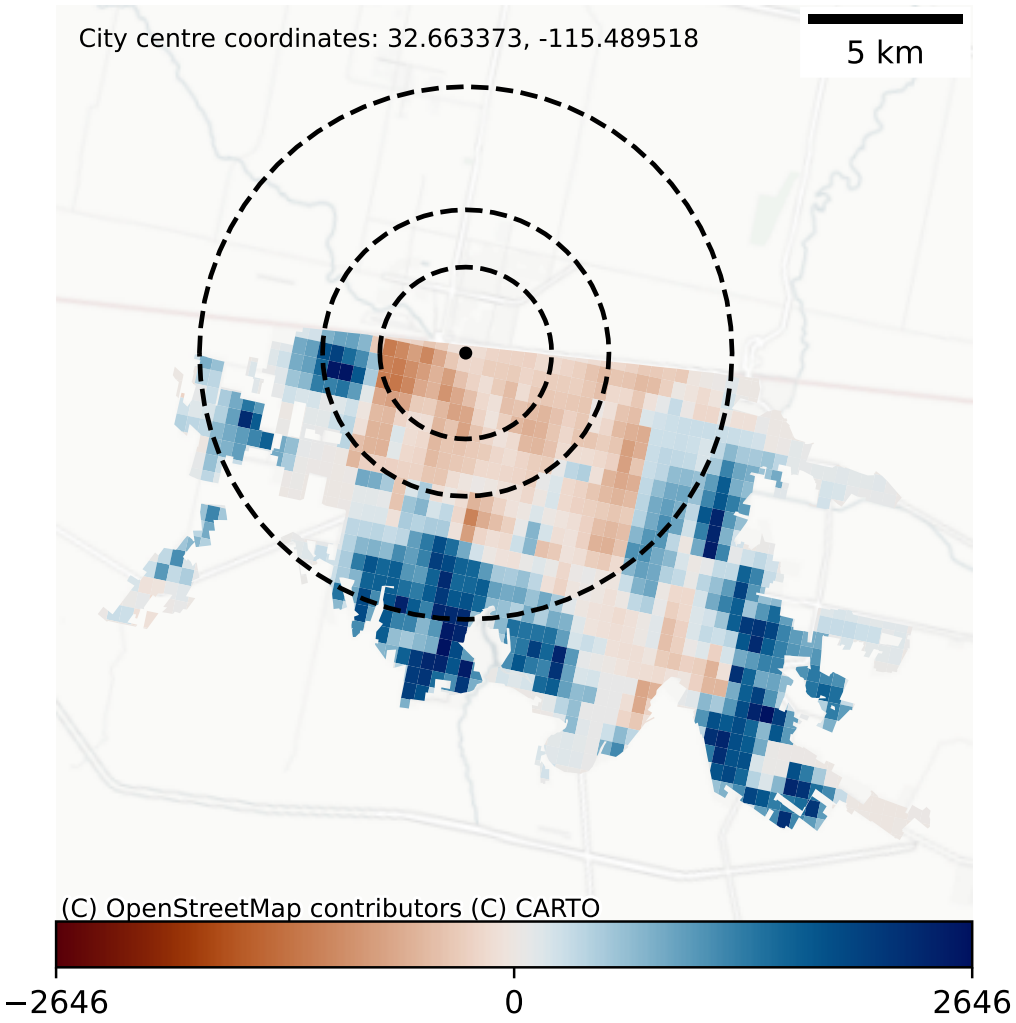

b)

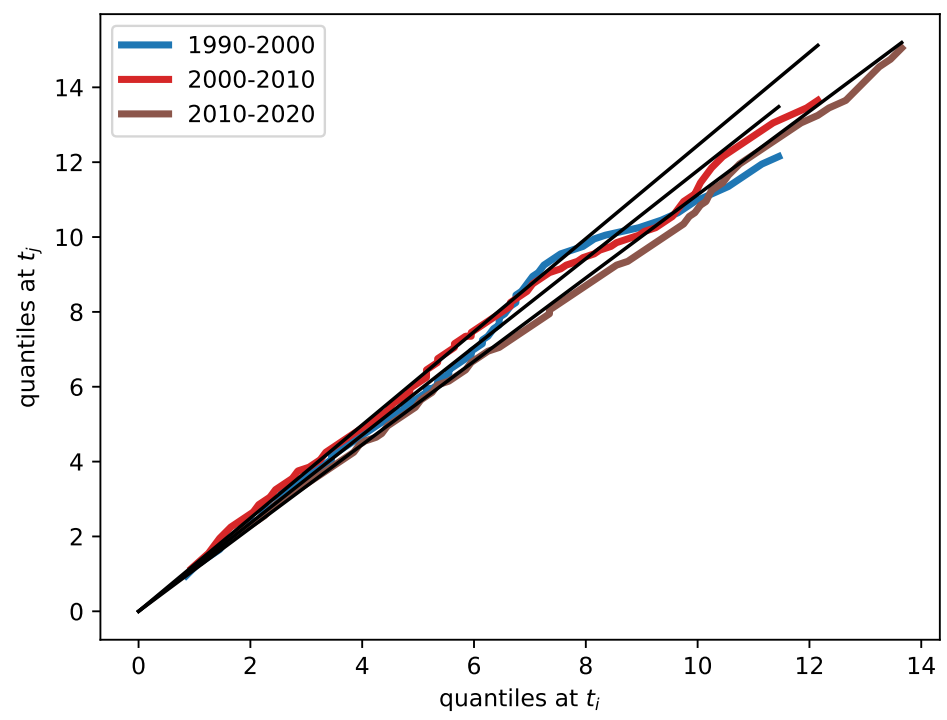

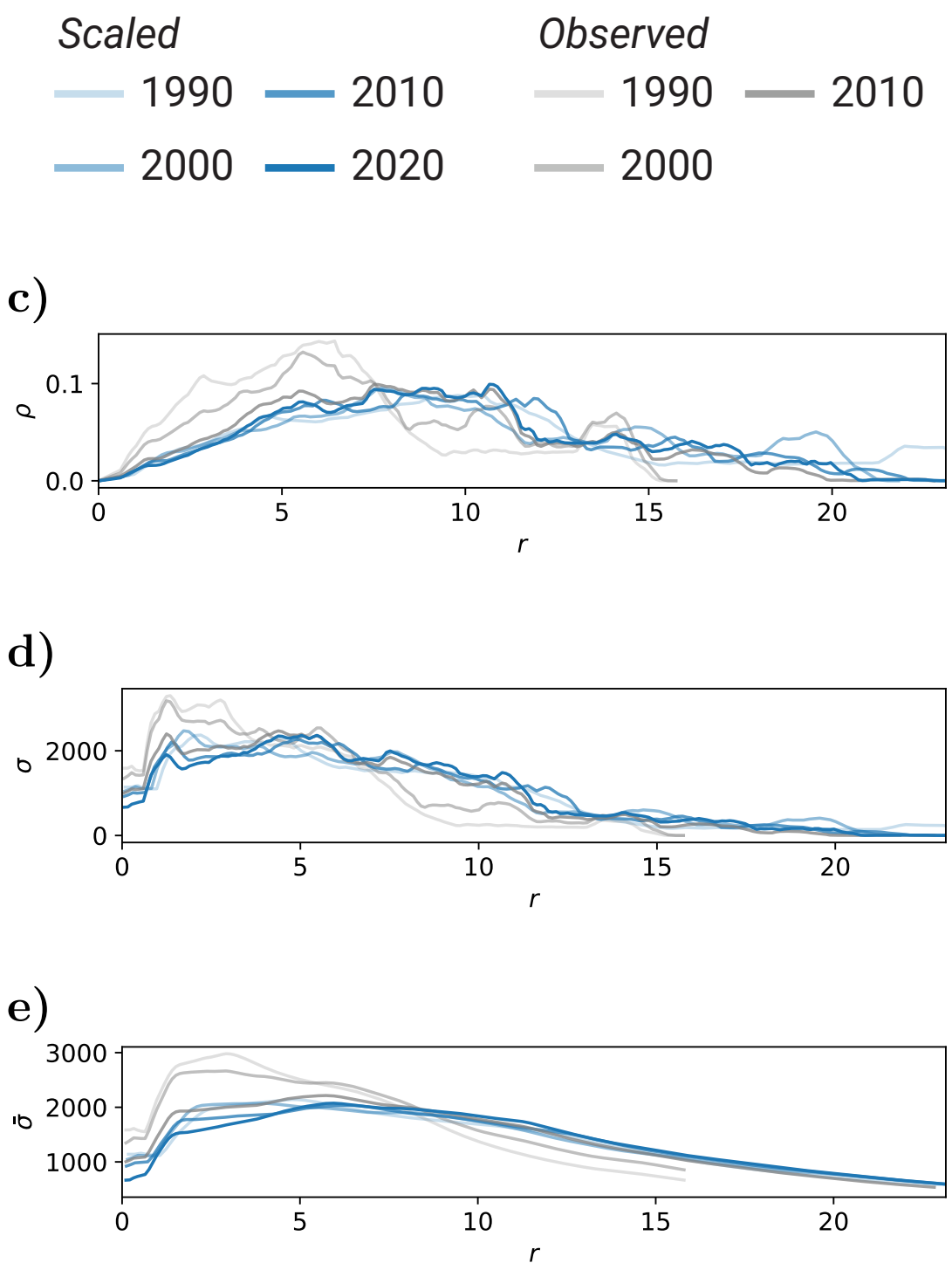

f)

Period (t_i-t_j)	$\frac{P(t_j)}{P(t_i)}$	Φ_{ij}	IQR
1990-2000	1.28	1.18	(1.16, 1.20)
2000-2010	1.31	1.24	(1.22, 1.29)
2010-2020	1.14	1.11	(1.10, 1.13)
1990-2010	1.68	1.47	(1.43, 1.53)
2000-2020	1.49	1.38	(1.36, 1.45)
1990-2020	1.91	1.64	(1.59, 1.71)

Supplementary Figure 9: Supplementary data for the metropolitan zone of Mexicali with code 02.2.03. Remoteness values are those of 2020. a) Population difference by grid cell (2020–1990); city centres are denoted as black dots. b) Quantile-quantile plots for the radial population distributions $\rho(s, t_i)$ and $\rho(s, t_j)$ (coloured curves); urban expansion factors Φ_{ij} from t_i to t_j are the estimated slopes (black lines). c) Radial population distribution $\rho(r)$ at remoteness distance r from the city centre. d) Radial population density $\sigma(r)$ at remoteness distance r from the city centre. e) Average population density $\bar{\sigma}(r)$ within disks of remoteness r with the same centre as the city. f) Urban expansion factors and their inter quartile range from the Theil-Sen estimation.

La Paz, 03.2.01

a)

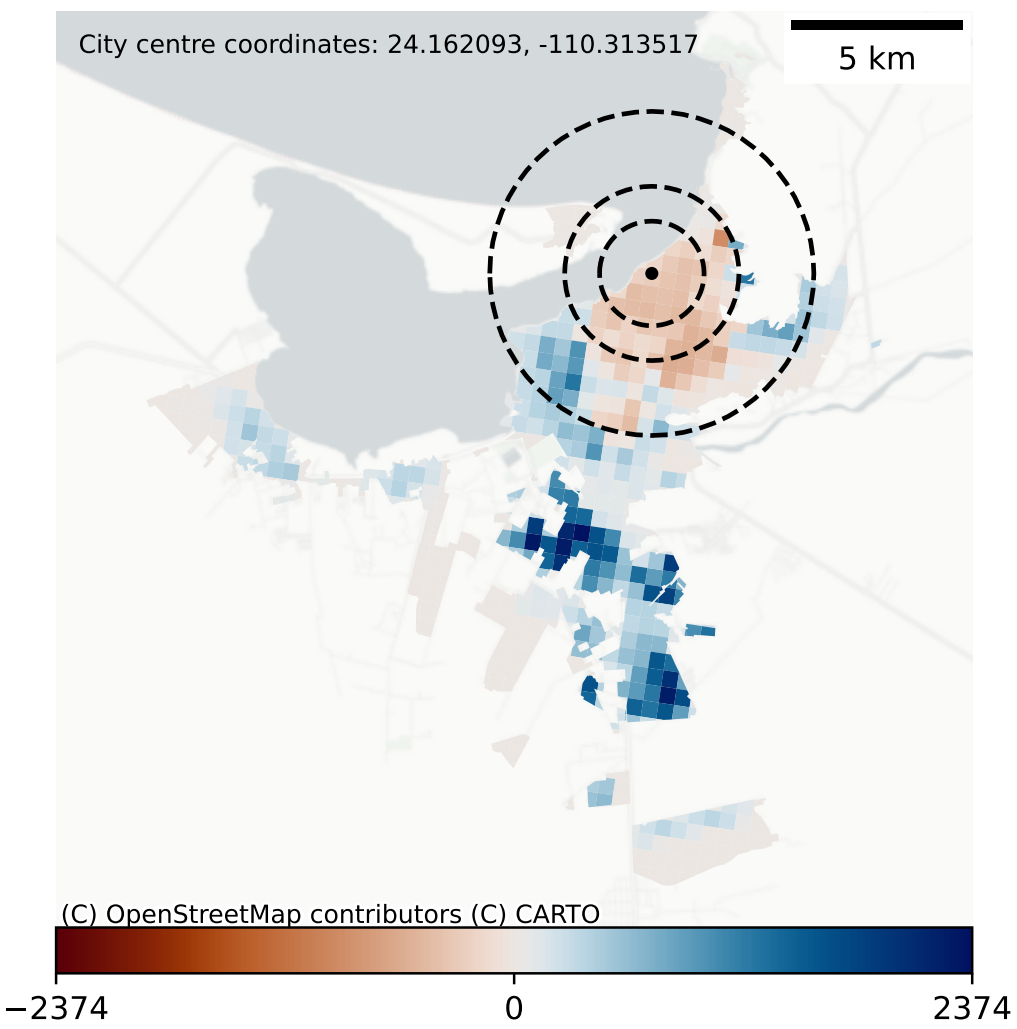

b)

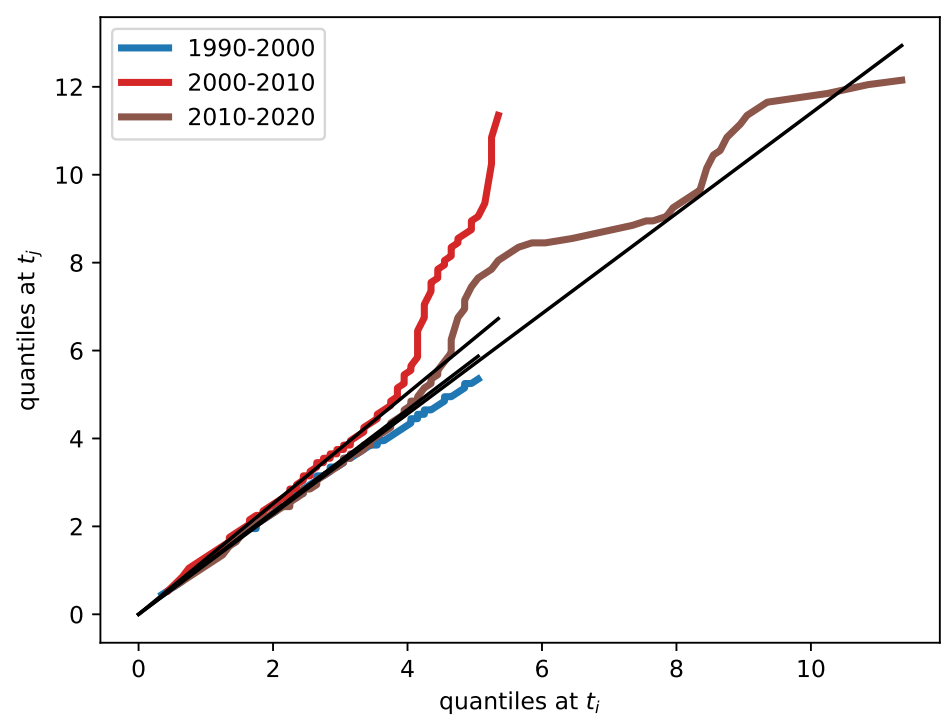

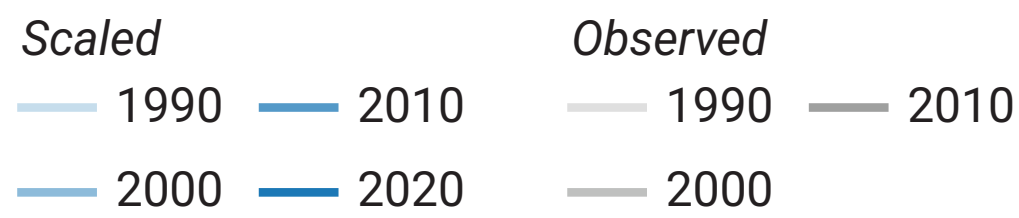

c)

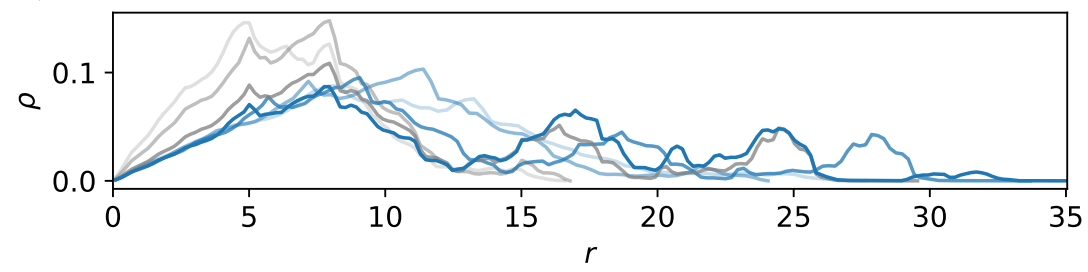

d)

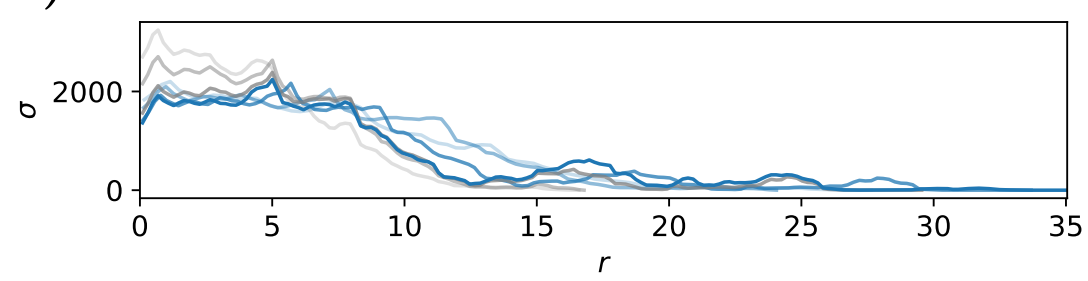

e)

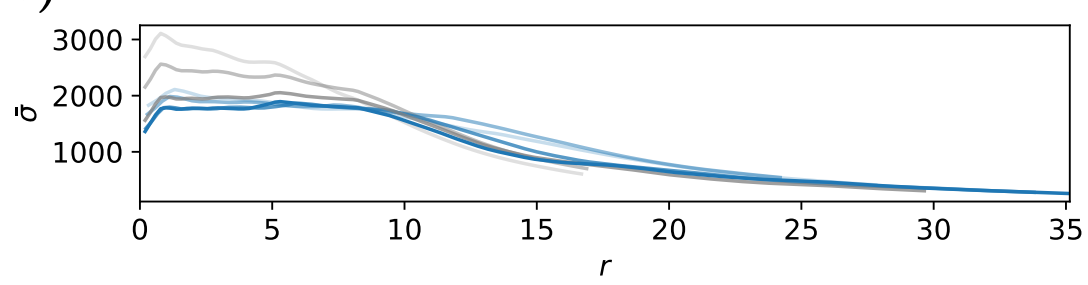

f)

Period (t_i-t_j)	$\frac{P(t_j)}{P(t_i)}$	Φ_{ij}	IQR
1990-2000	1.18	1.16	(1.15, 1.19)
2000-2010	1.35	1.26	(1.24, 1.28)
2010-2020	1.18	1.14	(1.12, 1.15)
1990-2010	1.60	1.47	(1.44, 1.51)
2000-2020	1.59	1.44	(1.42, 1.46)
1990-2020	1.88	1.67	(1.64, 1.72)

Supplementary Figure 10: Supplementary data for the metropolitan zone of La Paz with code 03.2.01. Remoteness values are those of 2020. a) Population difference by grid cell (2020–1990); city centres are denoted as black dots. b) Quantile-quantile plots for the radial population distributions $\rho(s, t_i)$ and $\rho(s, t_j)$ (coloured curves); urban expansion factors Φ_{ij} from t_i to t_j are the estimated slopes (black lines). c) Radial population distribution $\rho(r)$ at remoteness distance r from the city centre. d) Radial population density $\sigma(r)$ at remoteness distance r from the city centre. e) Average population density $\bar{\sigma}(r)$ within disks of remoteness r with the same centre as the city. f) Urban expansion factors and their inter quartile range from the Theil-Sen estimation.

Los Cabos, 03.2.02

a)

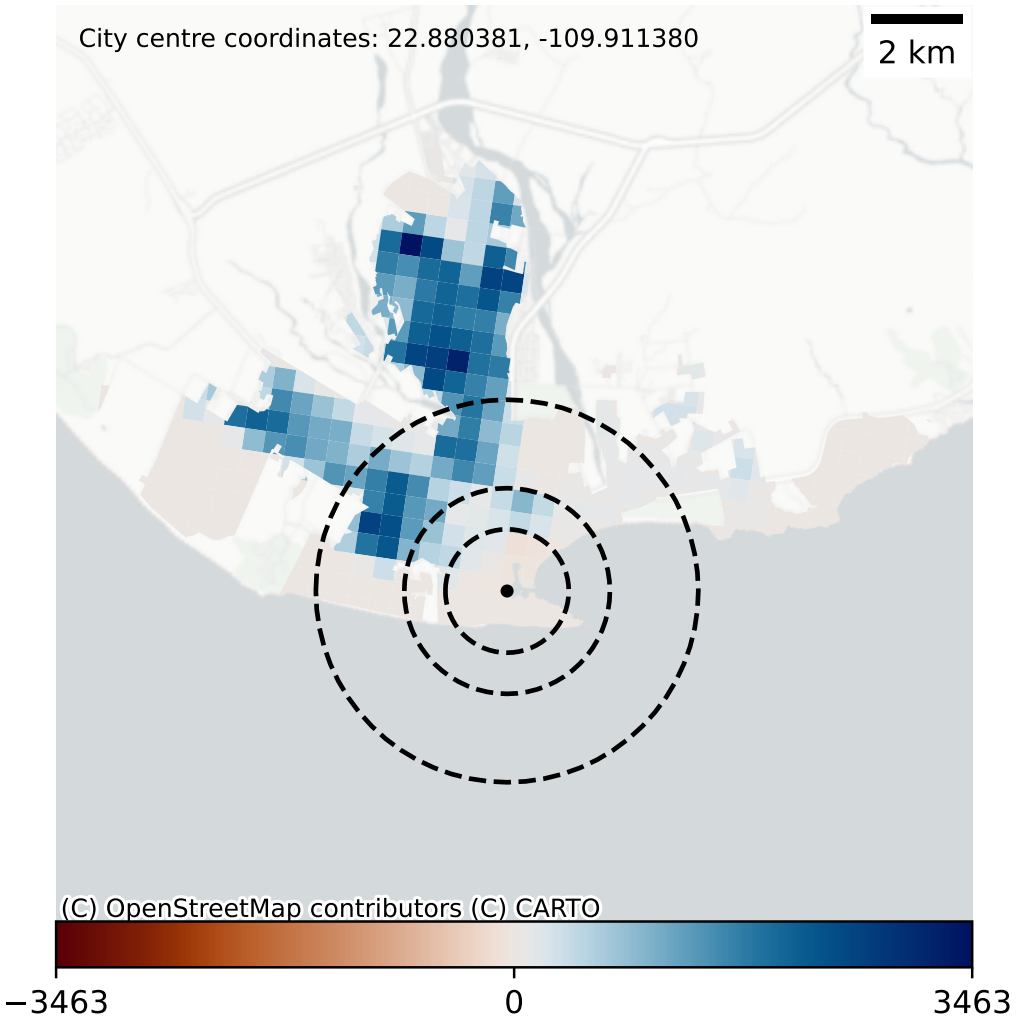

b)

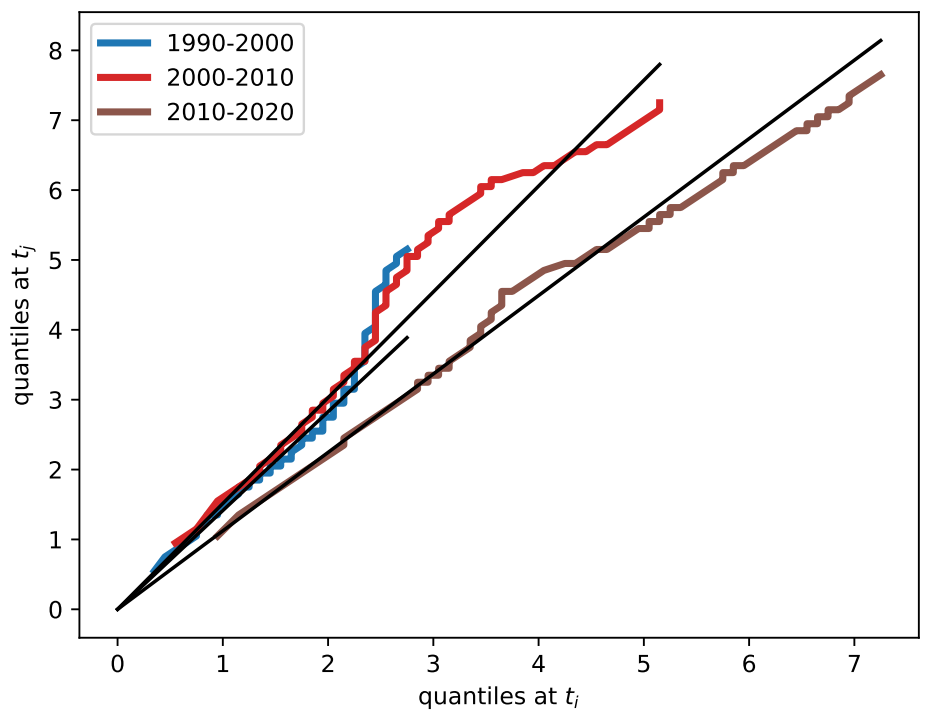

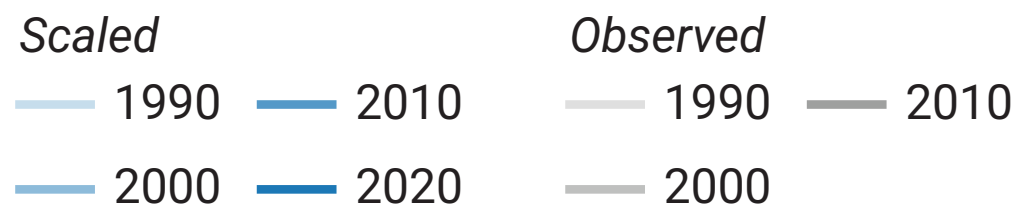

c)

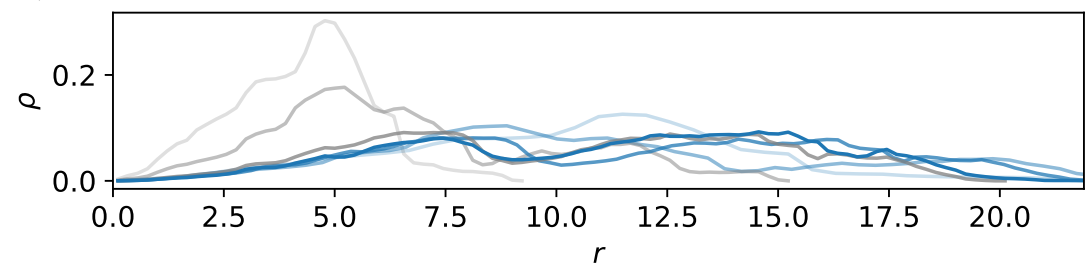

d)

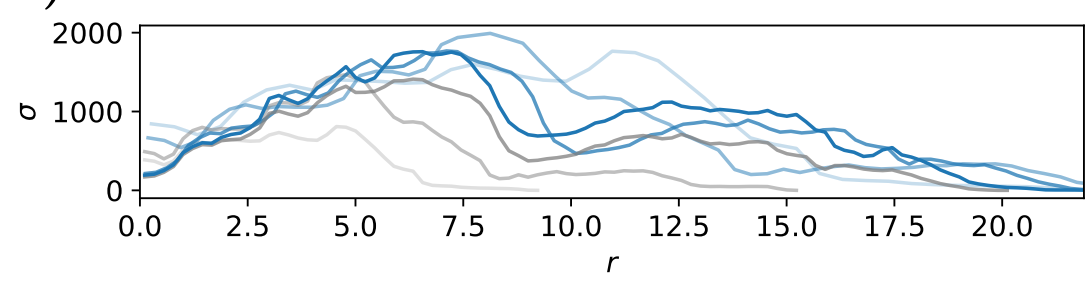

e)

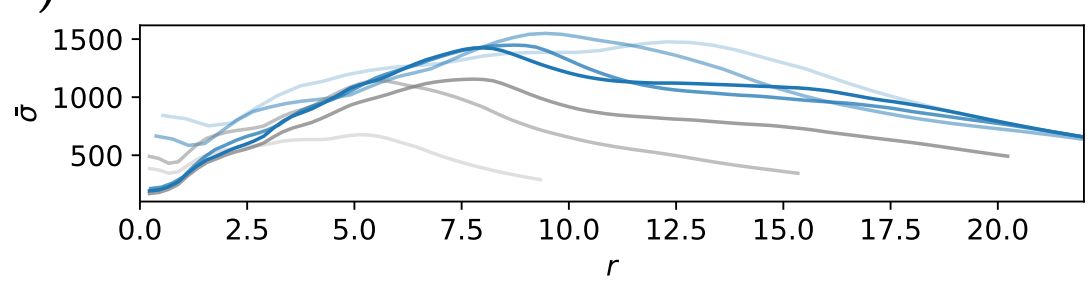

f)

Period (t_i-t_j)	$\frac{P(t_j)}{P(t_i)}$	Φ_{ij}	IQR
1990-2000	3.21	1.41	(1.37, 1.48)
2000-2010	2.48	1.51	(1.49, 1.54)
2010-2020	1.58	1.12	(1.11, 1.14)
1990-2010	7.97	2.14	(2.10, 2.21)
2000-2020	3.92	1.70	(1.67, 1.78)
1990-2020	12.60	2.42	(2.36, 2.51)

Supplementary Figure 11: Supplementary data for the metropolitan zone of Los Cabos with code 03.2.02. Remoteness values are those of 2020. a) Population difference by grid cell (2020–1990); city centres are denoted as black dots. b) Quantile-quantile plots for the radial population distributions $\rho(s, t_i)$ and $\rho(s, t_j)$ (coloured curves); urban expansion factors Φ_{ij} from t_i to t_j are the estimated slopes (black lines). c) Radial population distribution $\rho(r)$ at remoteness distance r from the city centre. d) Radial population density $\sigma(r)$ at remoteness distance r from the city centre. e) Average population density $\bar{\sigma}(r)$ within disks of remoteness r with the same centre as the city. f) Urban expansion factors and their inter quartile range from the Theil-Sen estimation.

Campeche, 04.2.01

a)

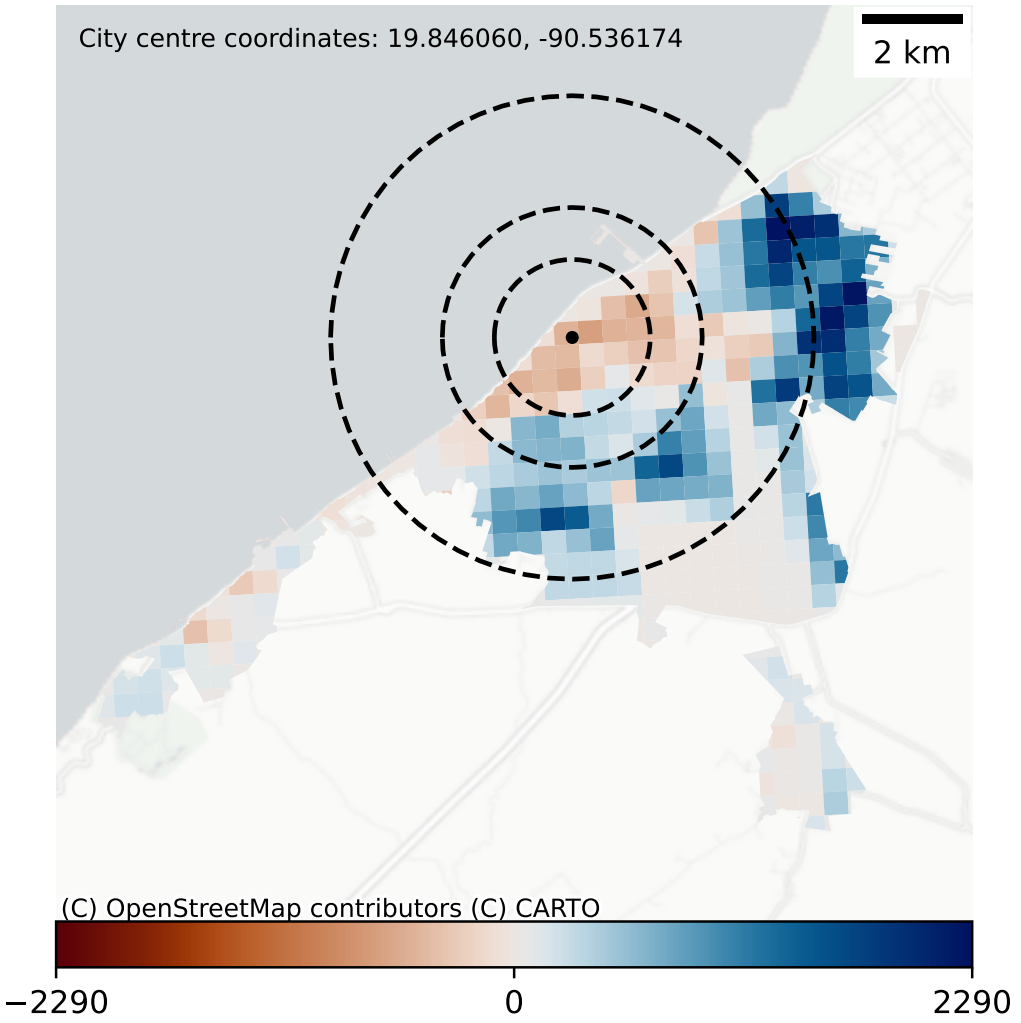

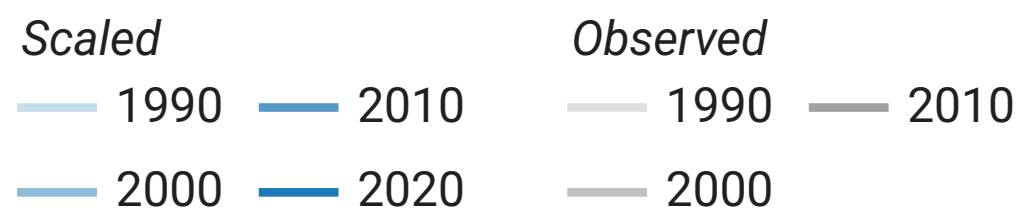

c)

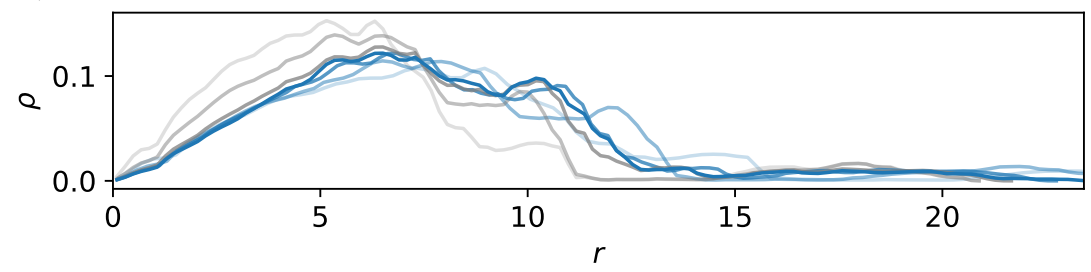

d)

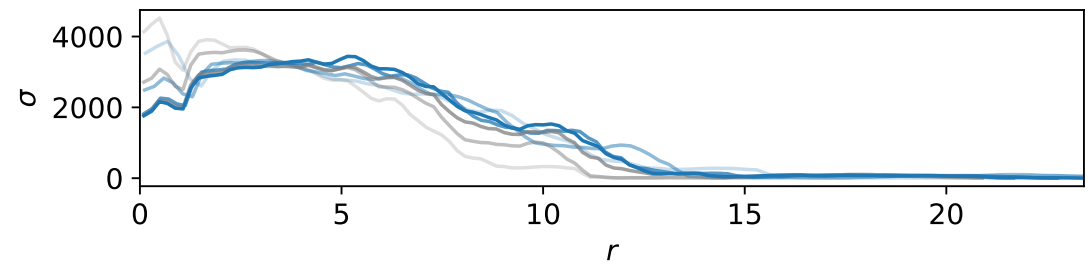

e)

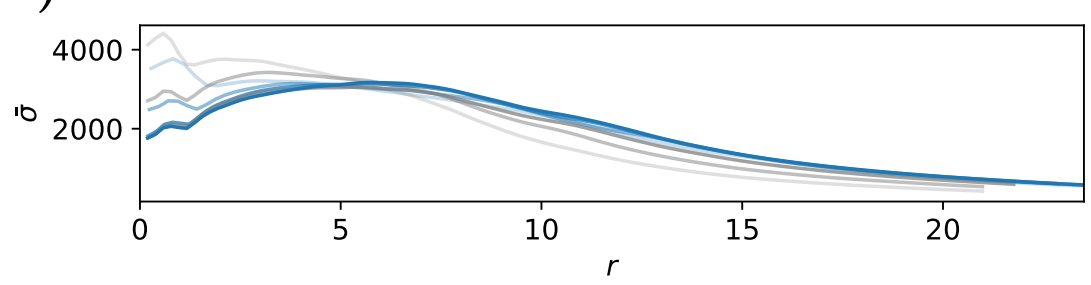

b)

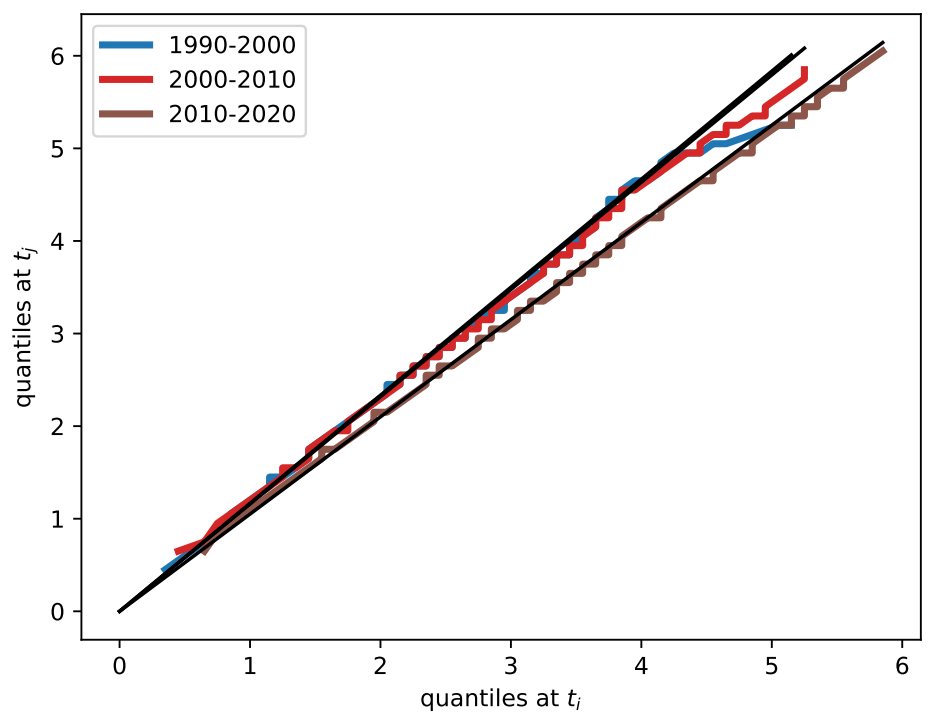

f)

Period (t_i-t_j)	$\frac{P(t_j)}{P(t_i)}$	Φ_{ij}	IQR
1990-2000	1.27	1.17	(1.15, 1.19)
2000-2010	1.20	1.16	(1.14, 1.18)
2010-2020	1.13	1.05	(1.04, 1.07)
1990-2010	1.52	1.36	(1.33, 1.39)
2000-2020	1.36	1.23	(1.20, 1.26)
1990-2020	1.72	1.44	(1.39, 1.48)

Supplementary Figure 12: Supplementary data for the metropolitan zone of Campeche with code 04.2.01. Remoteness values are those of 2020. a) Population difference by grid cell (2020–1990); city centres are denoted as black dots. b) Quantile-quantile plots for the radial population distributions $\rho(s, t_i)$ and $\rho(s, t_j)$ (coloured curves); urban expansion factors Φ_{ij} from t_i to t_j are the estimated slopes (black lines). c) Radial population distribution $\rho(r)$ at remoteness distance r from the city centre. d) Radial population density $\sigma(r)$ at remoteness distance r from the city centre. e) Average population density $\bar{\sigma}(r)$ within disks of remoteness r with the same centre as the city. f) Urban expansion factors and their inter quartile range from the Theil-Sen estimation.

La Laguna, 05.1.01

a)

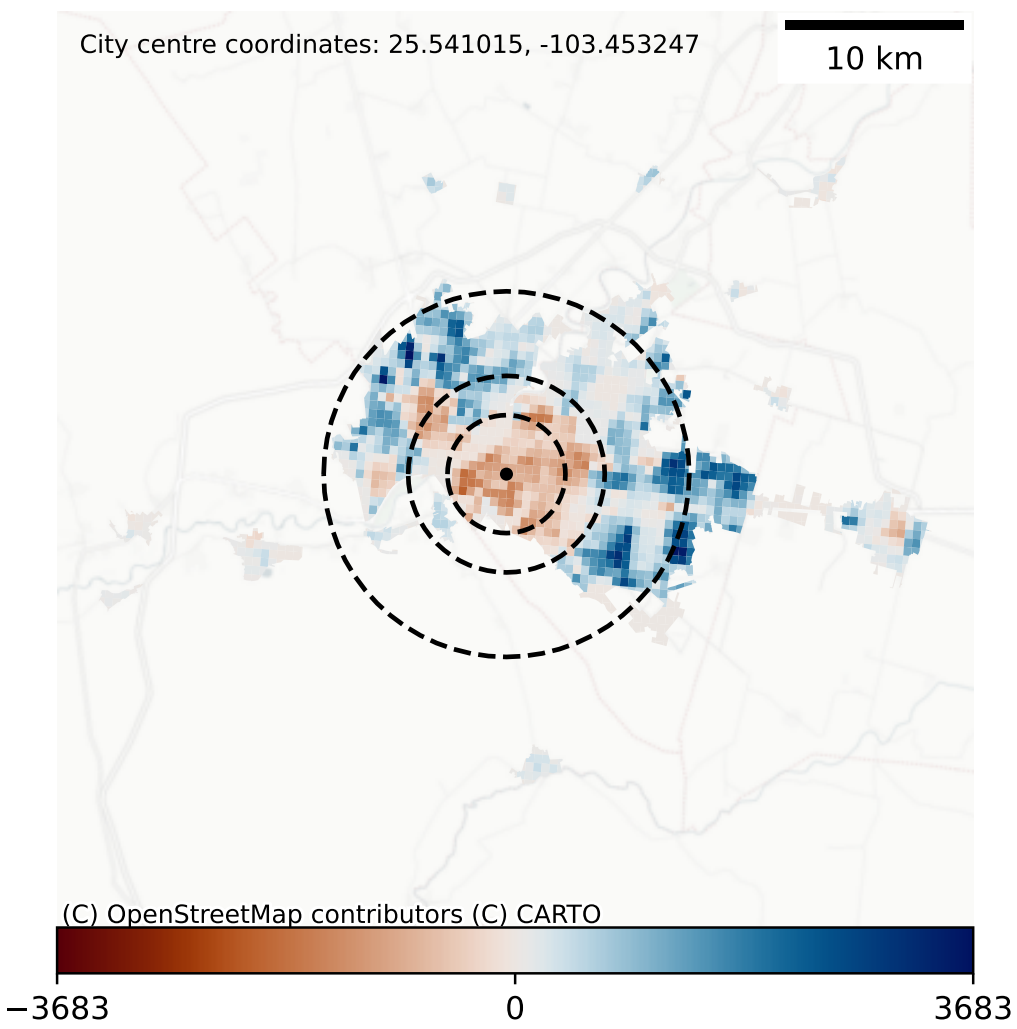

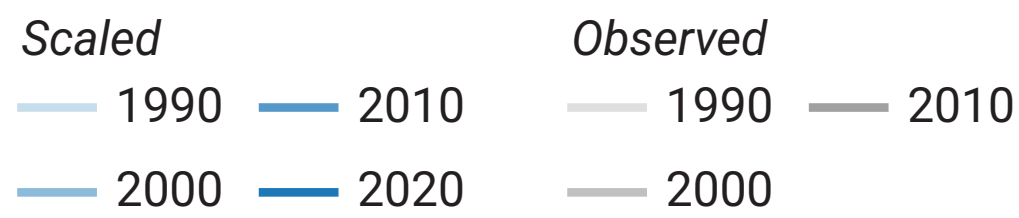

c)

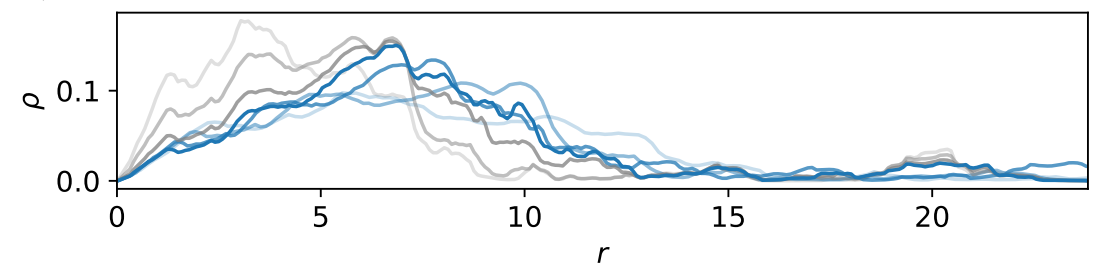

d)

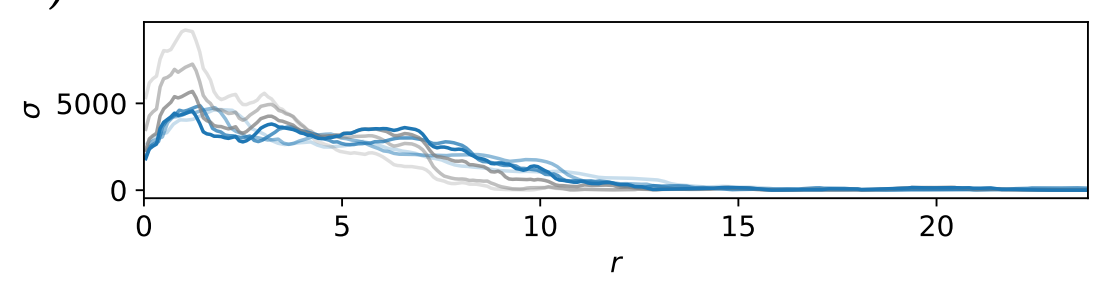

e)

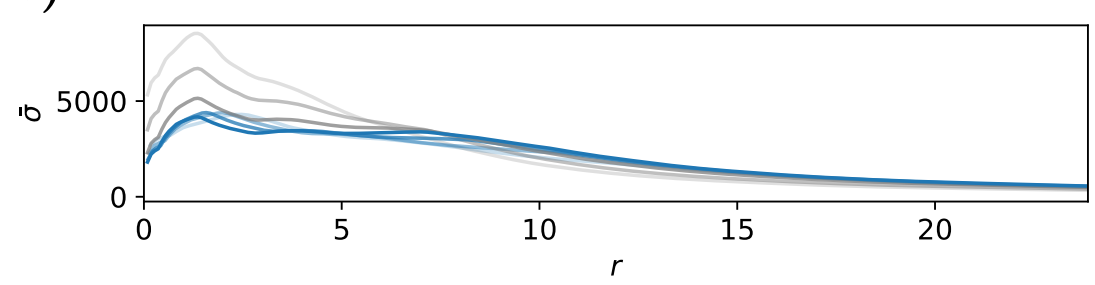

b)

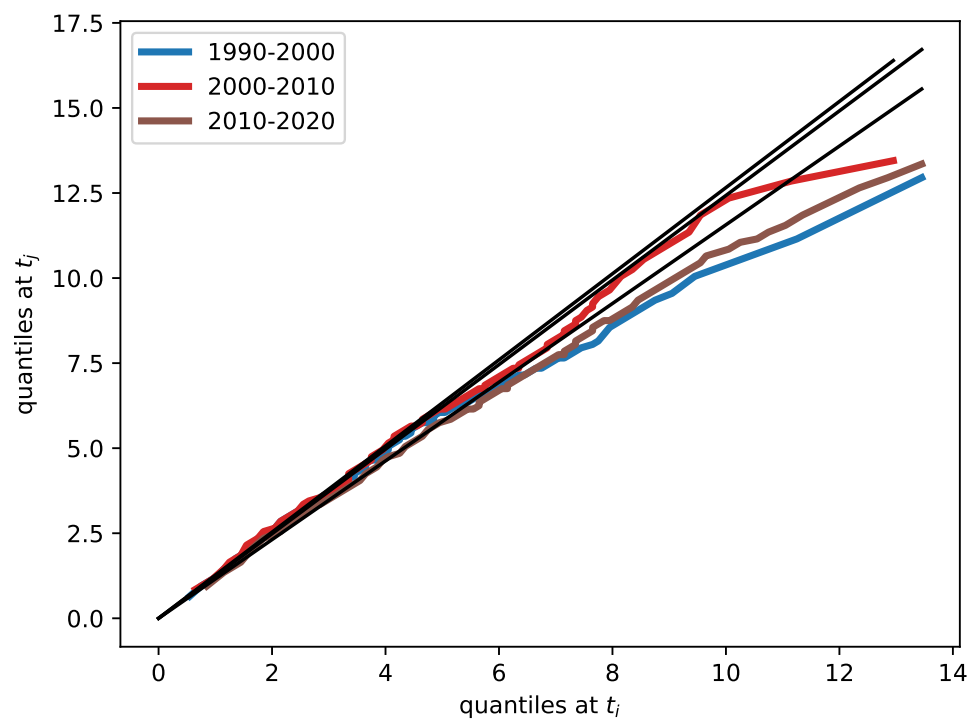

f)

Period $(t_i$-$t_j)$	$\frac{P(t_j)}{P(t_i)}$	Φ_{ij}	IQR
1990-2000	1.19	1.24	(1.22, 1.29)
2000-2010	1.23	1.27	(1.25, 1.29)
2010-2020	1.14	1.16	(1.13, 1.17)
1990-2010	1.46	1.56	(1.54, 1.64)
2000-2020	1.40	1.45	(1.42, 1.49)
1990-2020	1.67	1.80	(1.75, 1.95)

Supplementary Figure 13: Supplementary data for the metropolitan zone of La Laguna with code 05.1.01. Remoteness values are those of 2020. a) Population difference by grid cell (2020–1990); city centres are denoted as black dots. b) Quantile-quantile plots for the radial population distributions $\rho(s,t_i)$ and $\rho(s,t_j)$ (coloured curves); urban expansion factors Φ_{ij} from t_i to t_j are the estimated slopes (black lines). c) Radial population distribution $\rho(r)$ at remoteness distance r from the city centre. d) Radial population density $\sigma(r)$ at remoteness distance r from the city centre. e) Average population density $\bar{\sigma}(r)$ within disks of remoteness r with the same centre as the city. f) Urban expansion factors and their inter quartile range from the Theil-Sen estimation.

Monclova-Frontera, 05.1.02

a)

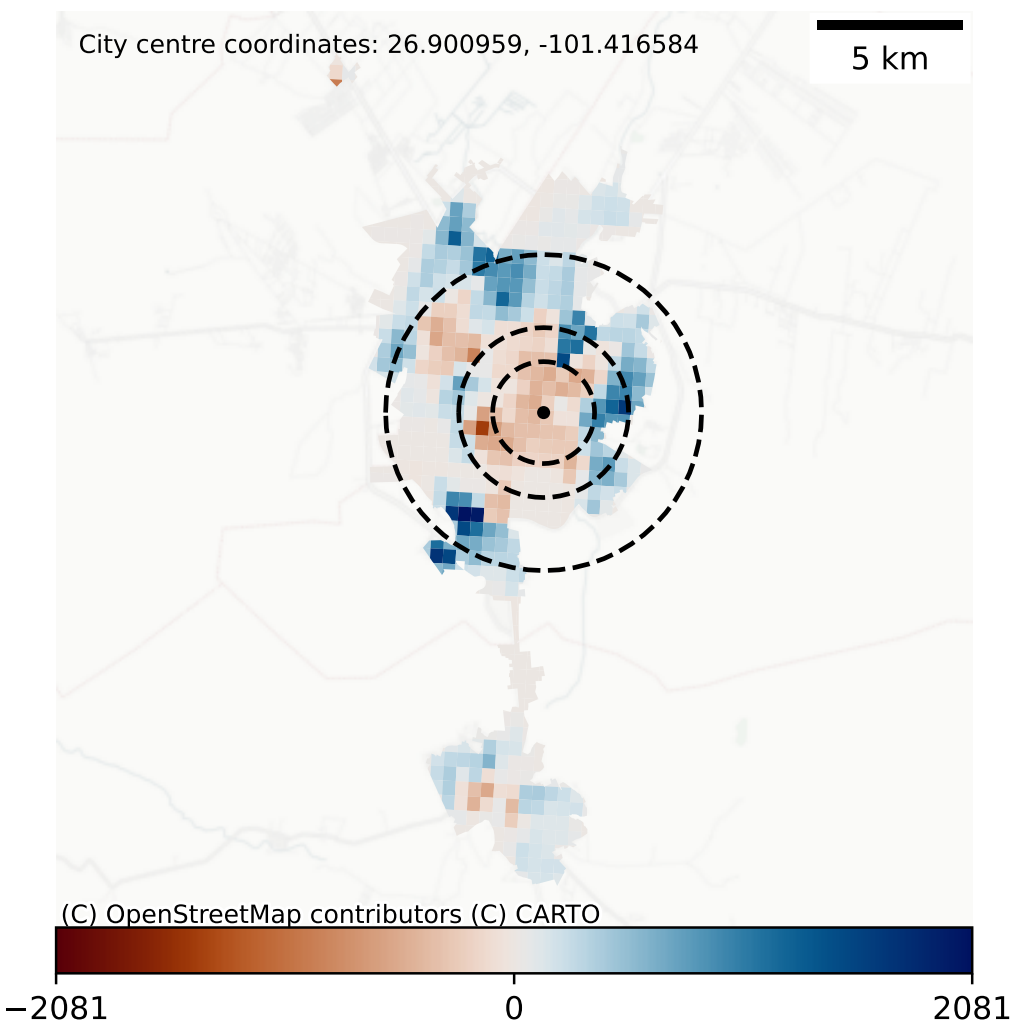

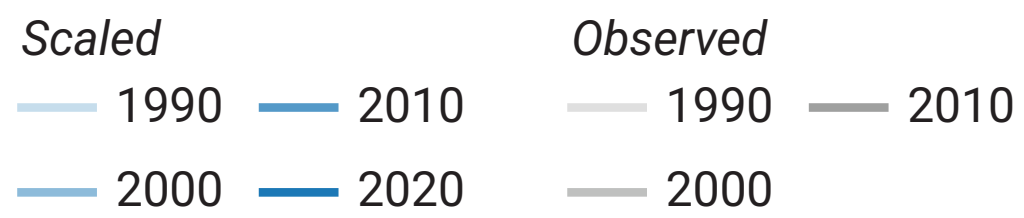

c)

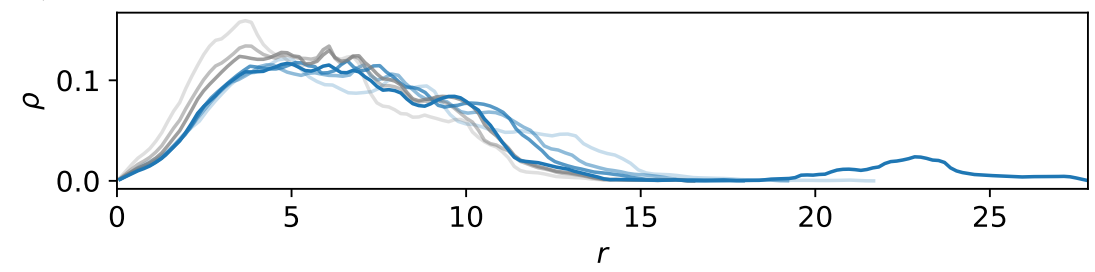

d)

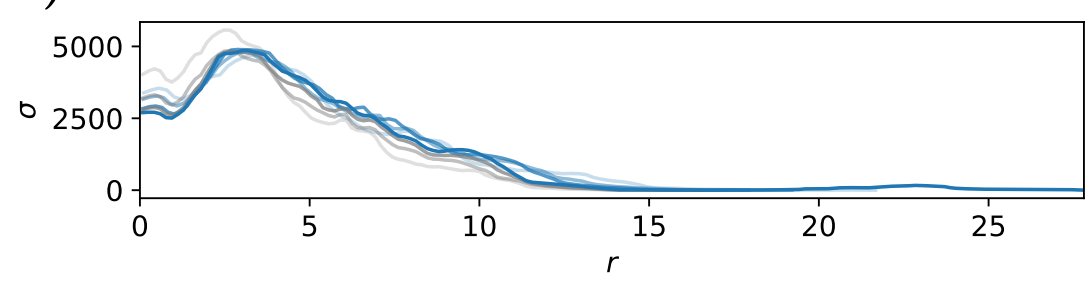

e)

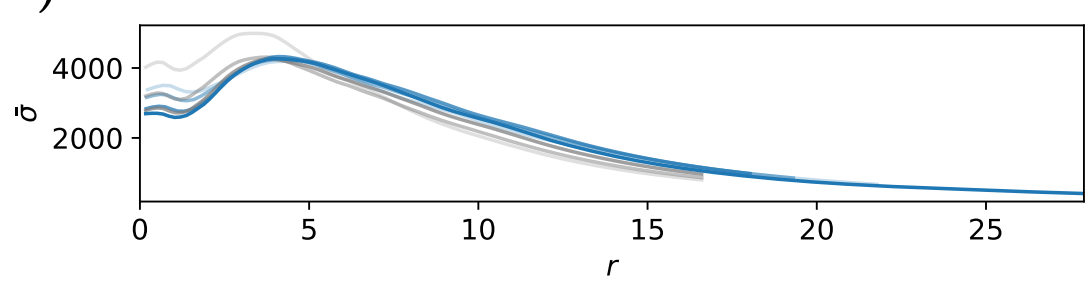

b)

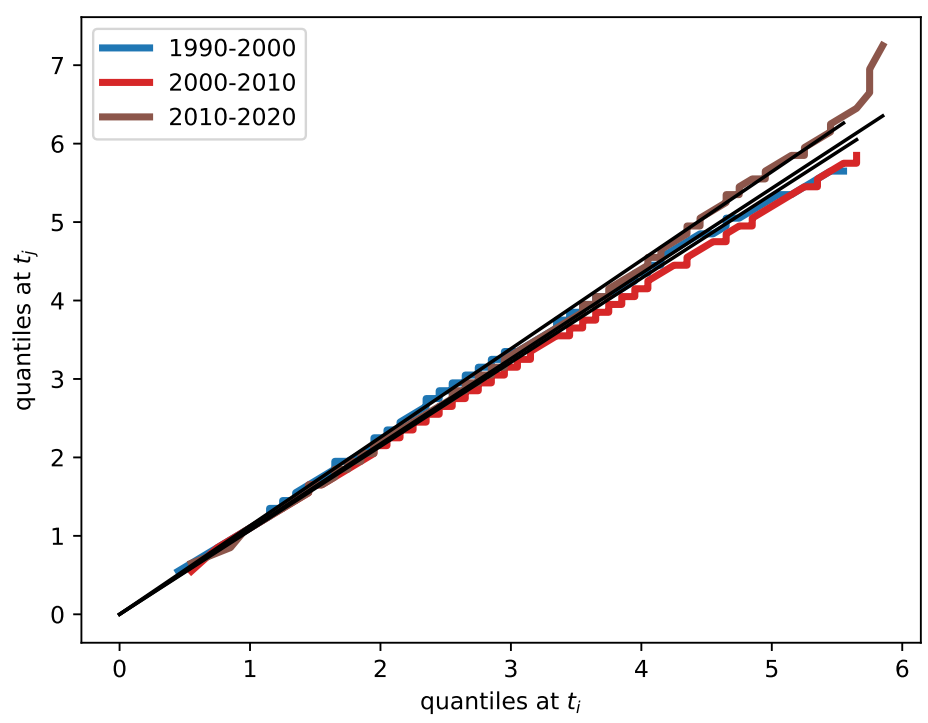

f)

Period (t_i-t_j)	$\frac{P(t_j)}{P(t_i)}$	Φ_{ij}	IQR
1990-2000	1.08	1.13	(1.11, 1.14)
2000-2010	1.11	1.07	(1.05, 1.09)
2010-2020	1.20	1.09	(1.07, 1.10)
1990-2010	1.21	1.21	(1.19, 1.22)
2000-2020	1.34	1.16	(1.15, 1.18)
1990-2020	1.45	1.31	(1.29, 1.34)

Supplementary Figure 14: Supplementary data for the metropolitan zone of Monclova-Frontera with code 05.1.02. Remoteness values are those of 2020. a) Population difference by grid cell (2020–1990); city centres are denoted as black dots. b) Quantile-quantile plots for the radial population distributions $\rho(s,t_i)$ and $\rho(s,t_j)$ (coloured curves); urban expansion factors Φ_{ij} from t_i to t_j are the estimated slopes (black lines). c) Radial population distribution $\rho(r)$ at remoteness distance r from the city centre. d) Radial population density $\sigma(r)$ at remoteness distance r from the city centre. e) Average population density $\bar{\sigma}(r)$ within disks of remoteness r with the same centre as the city. f) Urban expansion factors and their inter quartile range from the Theil-Sen estimation.

Piedras Negras, 05.1.03

a)

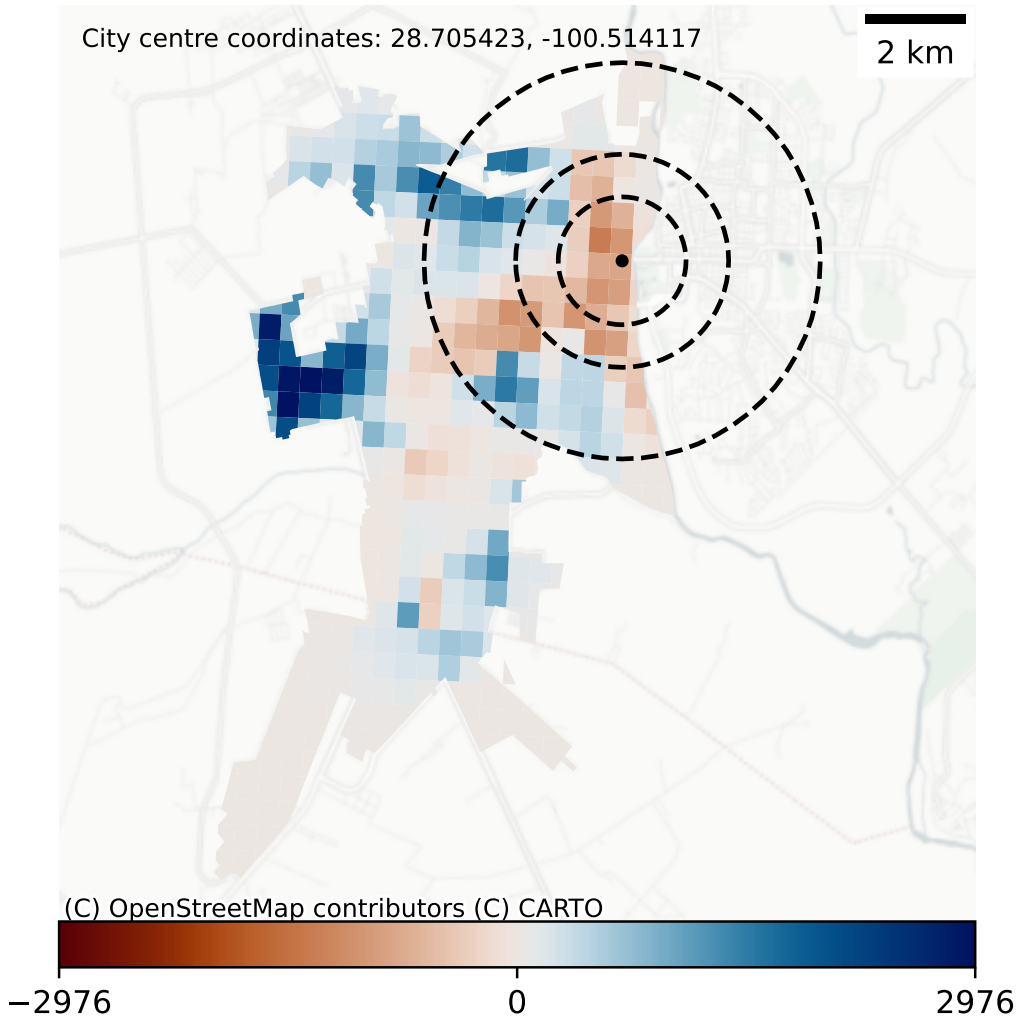

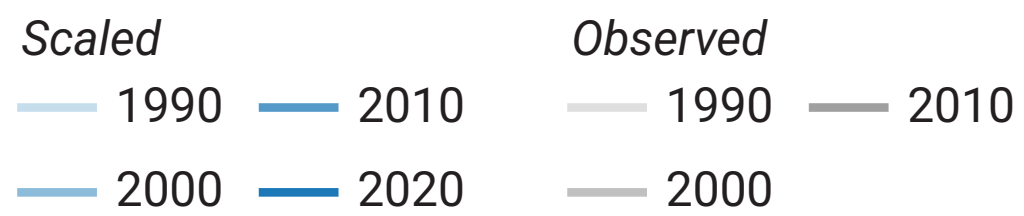

c)

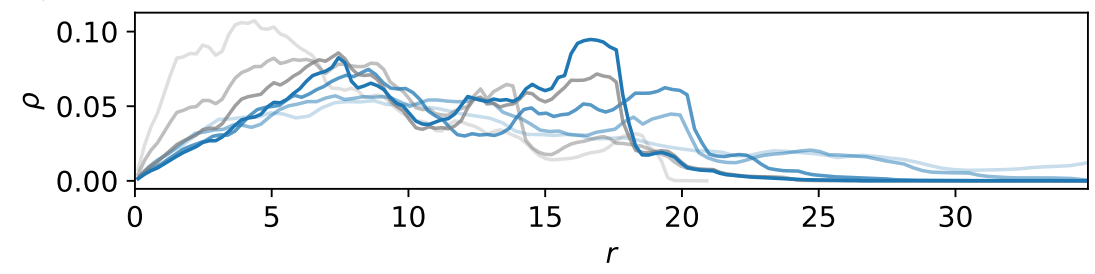

d)

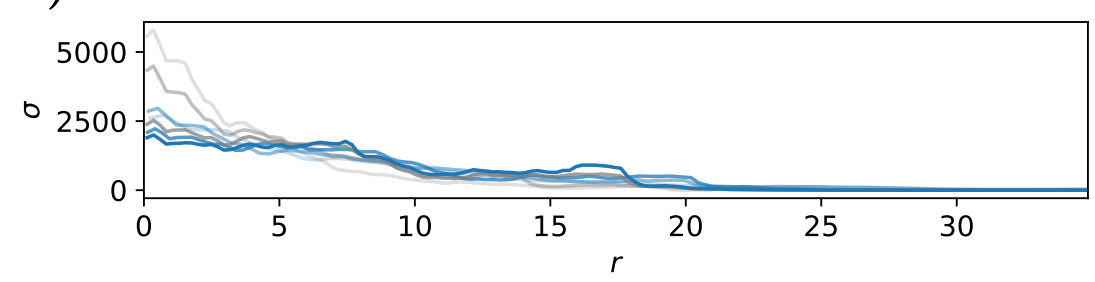

b)

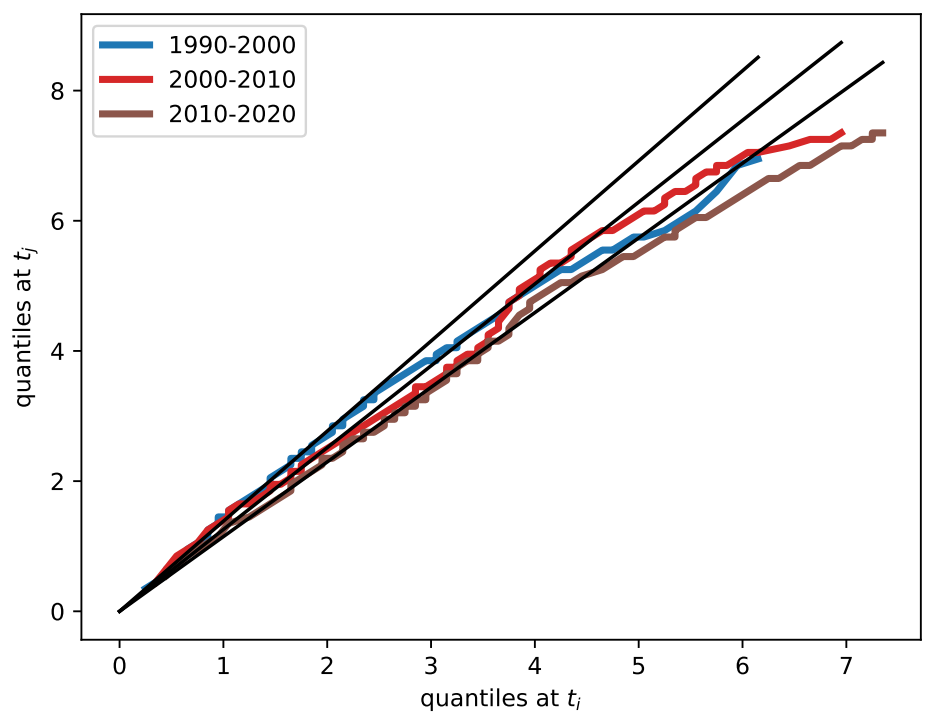

e)

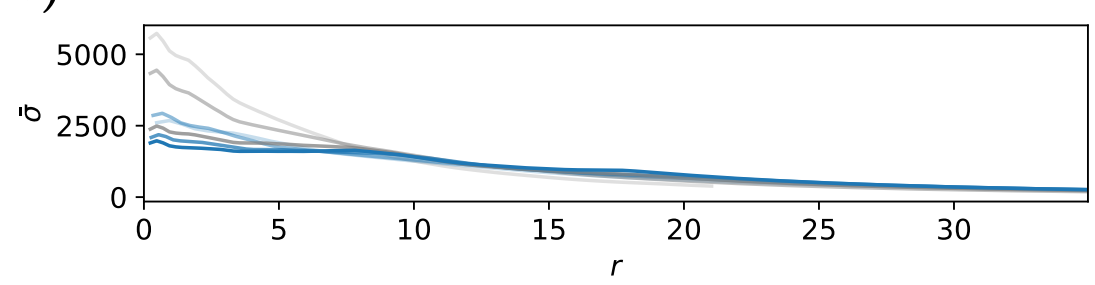

f)

Period (t_i-t_j)	$\frac{P(t_j)}{P(t_i)}$	Φ_{ij}	IQR
1990-2000	1.36	1.38	(1.36, 1.41)
2000-2010	1.19	1.26	(1.22, 1.34)
2010-2020	1.16	1.15	(1.13, 1.17)
1990-2010	1.61	1.76	(1.67, 1.88)
2000-2020	1.37	1.45	(1.37, 1.57)
1990-2020	1.86	2.04	(1.88, 2.22)

Supplementary Figure 15: Supplementary data for the metropolitan zone of Piedras Negras with code 05.1.03. Remoteness values are those of 2020. a) Population difference by grid cell (2020–1990); city centres are denoted as black dots. b) Quantile-quantile plots for the radial population distributions $\rho(s,t_i)$ and $\rho(s,t_j)$ (coloured curves); urban expansion factors Φ_{ij} from t_i to t_j are the estimated slopes (black lines). c) Radial population distribution $\rho(r)$ at remoteness distance r from the city centre. d) Radial population density $\sigma(r)$ at remoteness distance r from the city centre. e) Average population density $\bar{\sigma}(r)$ within disks of remoteness r with the same centre as the city. f) Urban expansion factors and their inter quartile range from the Theil-Sen estimation.

Saltillo, 05.1.04

a)

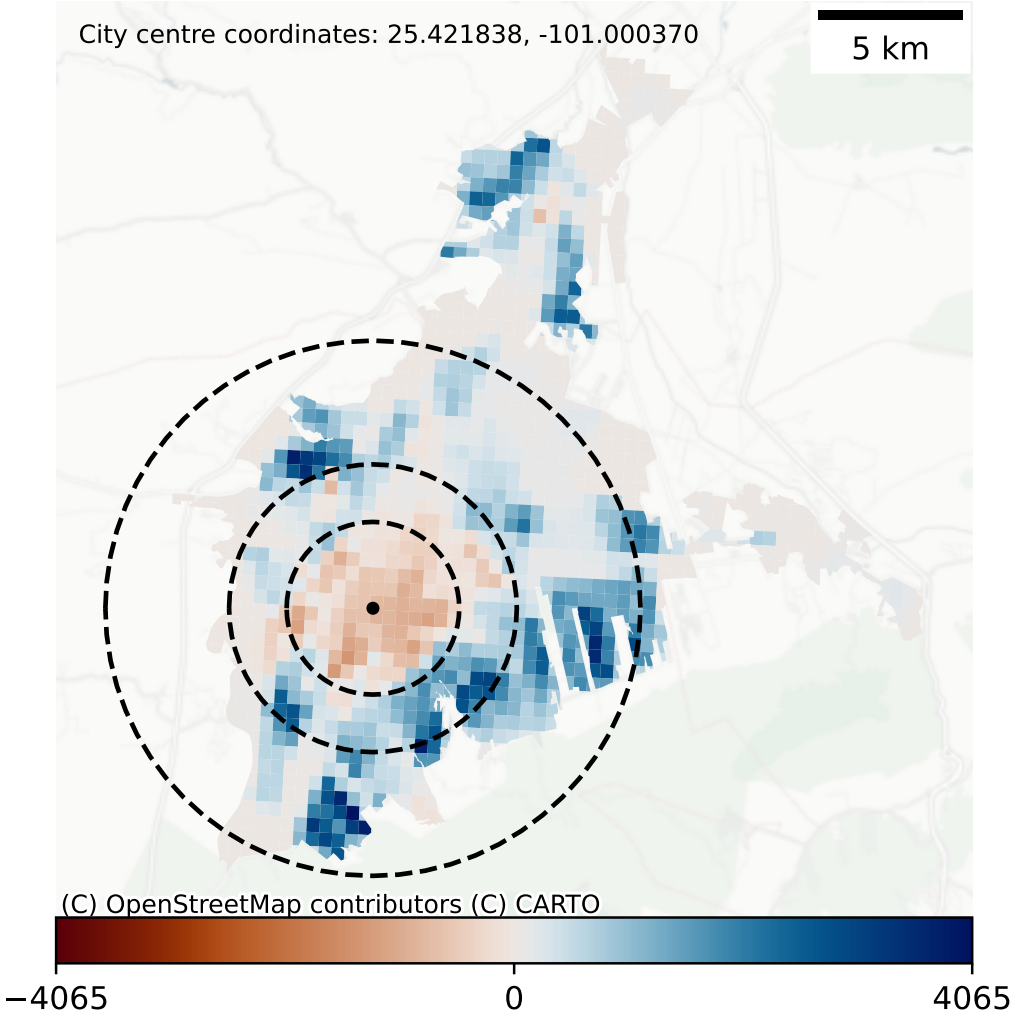

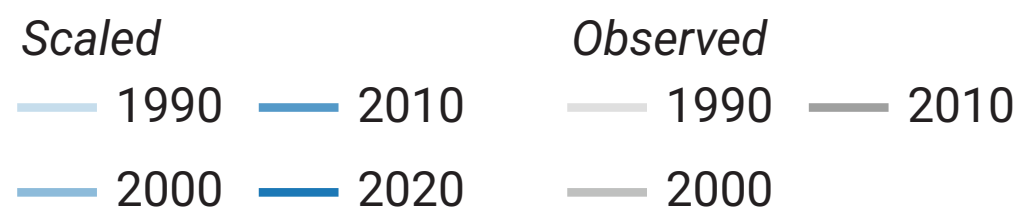

c)

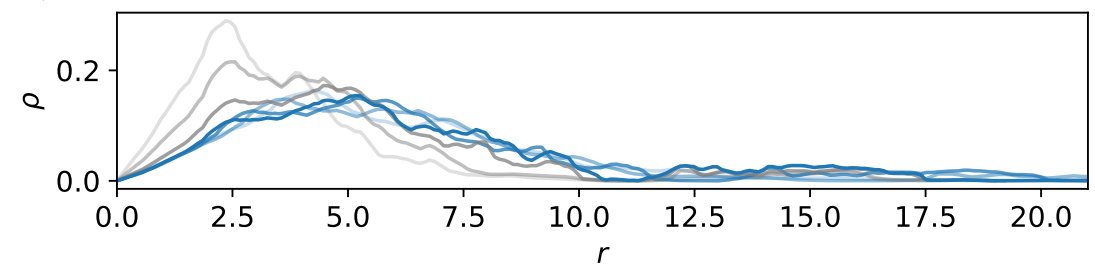

d)

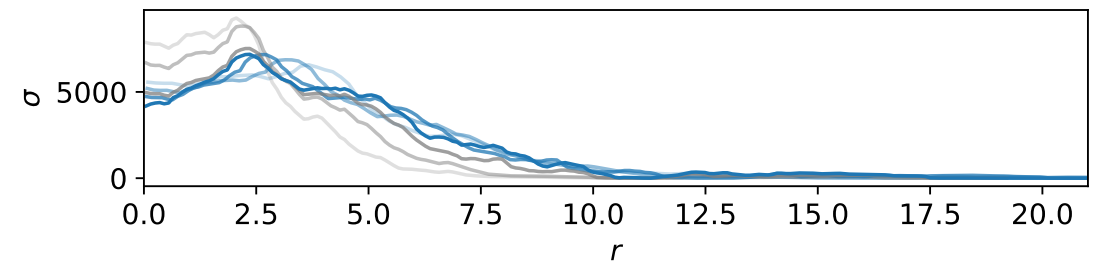

e)

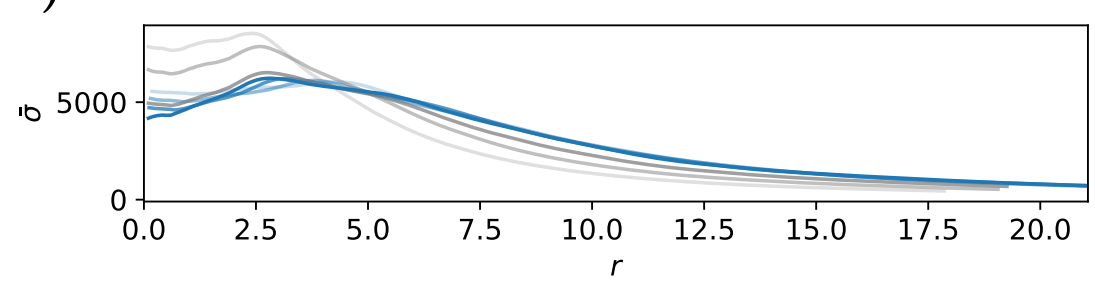

b)

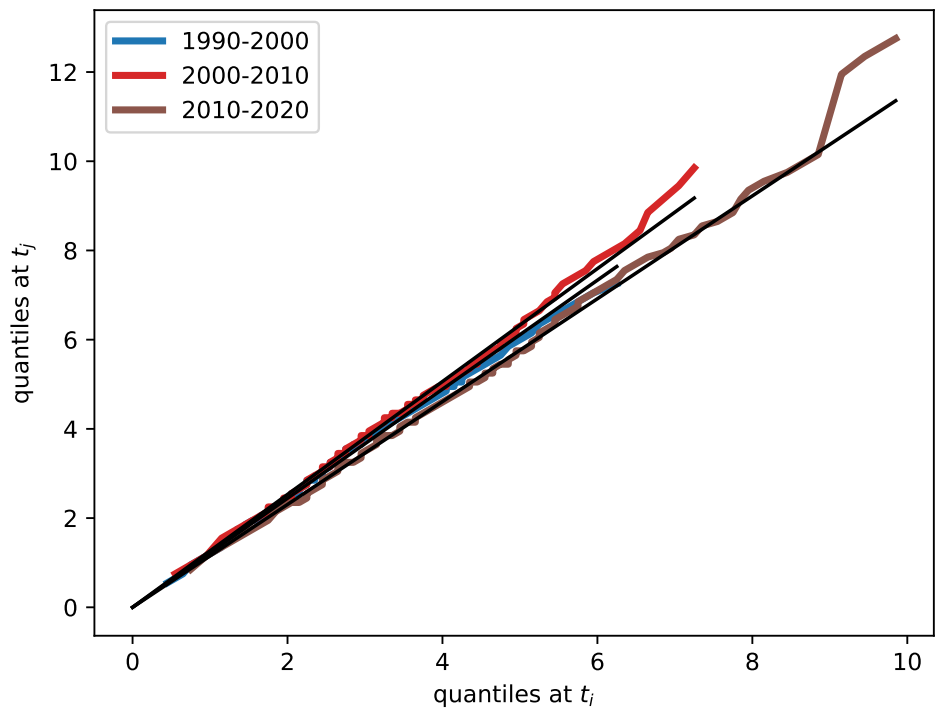

f)

Period $(t_i\text{-}t_j)$	$\frac{P(t_j)}{P(t_i)}$	Φ_{ij}	IQR
1990-2000	1.36	1.22	(1.20, 1.24)
2000-2010	1.31	1.27	(1.24, 1.29)
2010-2020	1.27	1.15	(1.14, 1.16)
1990-2010	1.78	1.55	(1.51, 1.59)
2000-2020	1.66	1.46	(1.43, 1.49)
1990-2020	2.25	1.79	(1.74, 1.82)

Supplementary Figure 16: Supplementary data for the metropolitan zone of Saltillo with code 05.1.04. Remoteness values are those of 2020. a) Population difference by grid cell (2020–1990); city centres are denoted as black dots. b) Quantile-quantile plots for the radial population distributions $\rho(s, t_i)$ and $\rho(s, t_j)$ (coloured curves); urban expansion factors Φ_{ij} from t_i to t_j are the estimated slopes (black lines). c) Radial population distribution $\rho(r)$ at remoteness distance r from the city centre. d) Radial population density $\sigma(r)$ at remoteness distance r from the city centre. e) Average population density $\bar{\sigma}(r)$ within disks of remoteness r with the same centre as the city. f) Urban expansion factors and their inter quartile range from the Theil-Sen estimation.

Colima-Villa de Álvarez, 06.1.01

a)

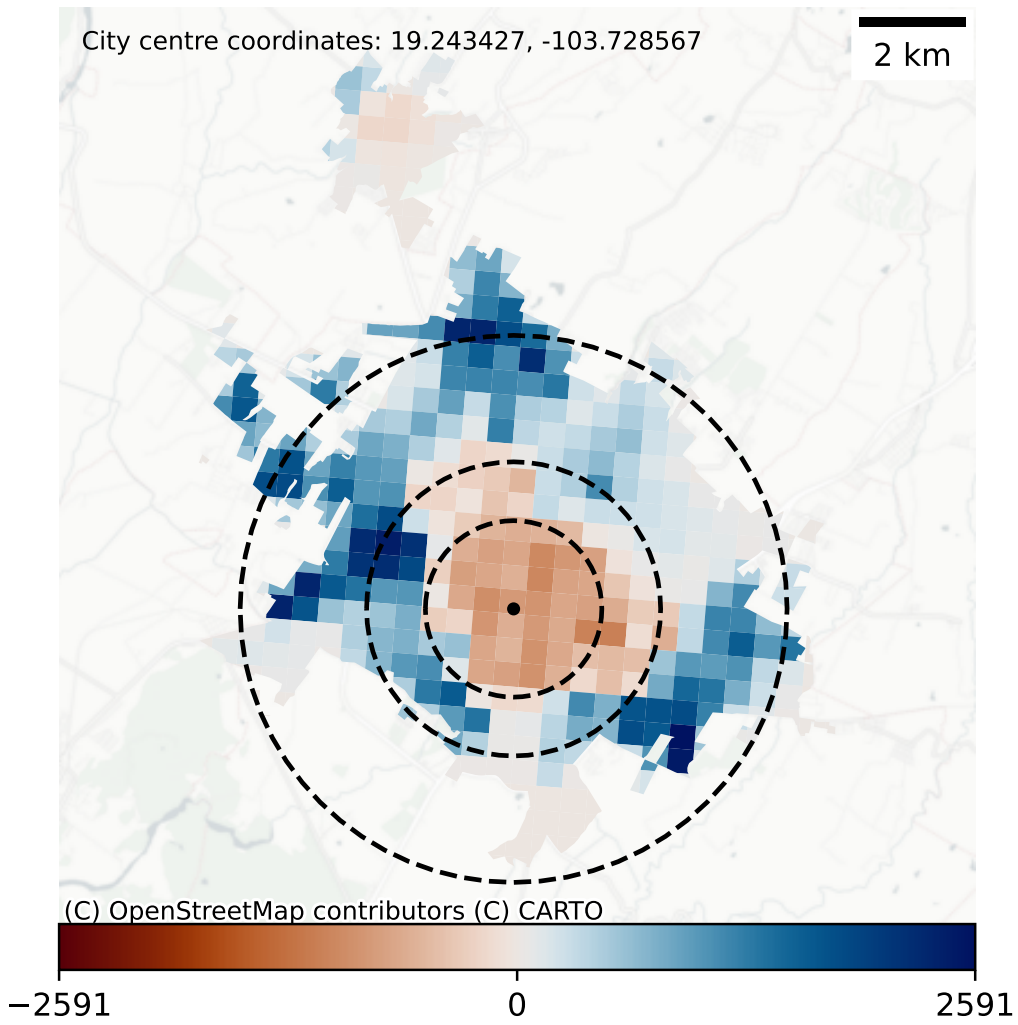

b)

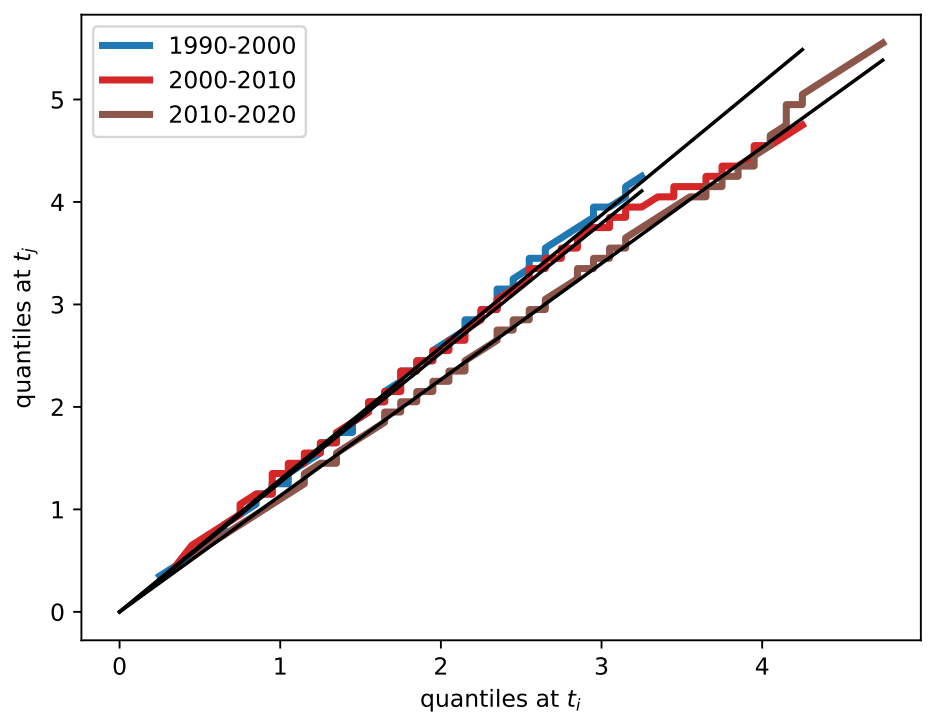

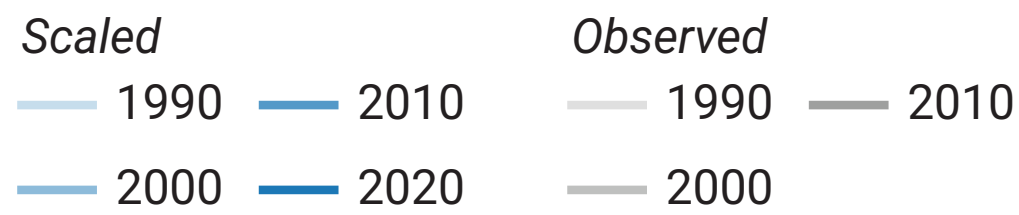

c)

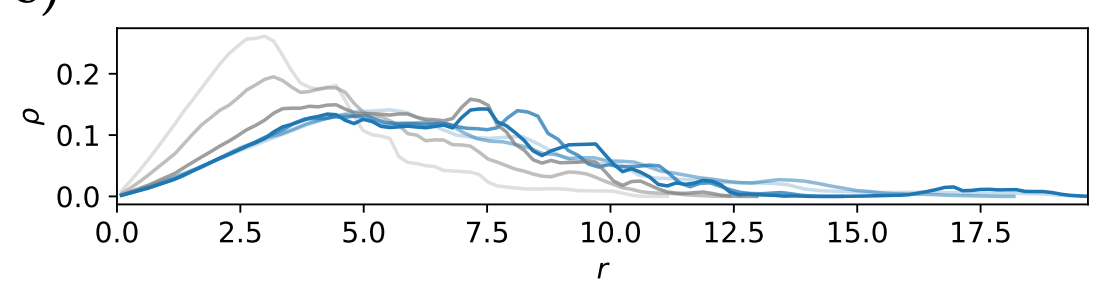

d)

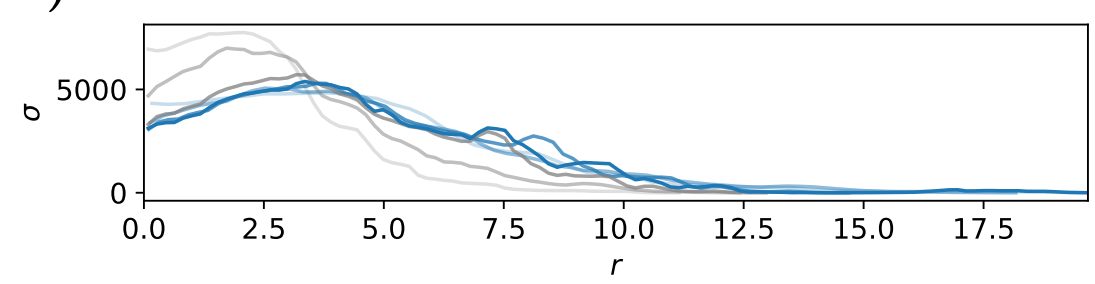

e)

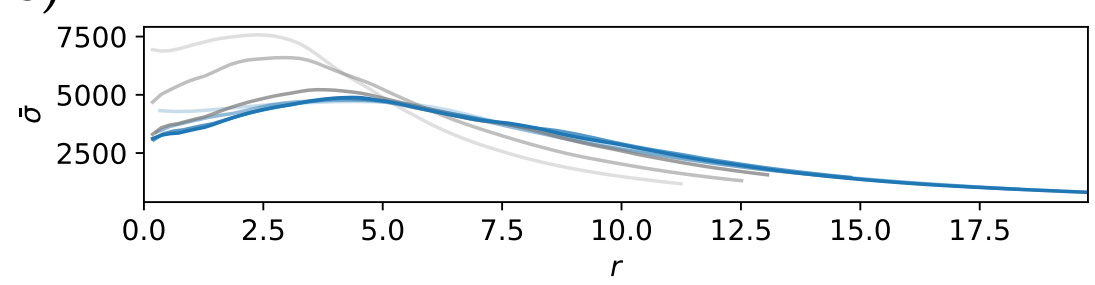

f)

Period (t_i-t_j)	$\frac{P(t_j)}{P(t_i)}$	Φ_{ij}	IQR
1990-2000	1.37	1.26	(1.24, 1.29)
2000-2010	1.30	1.29	(1.27, 1.32)
2010-2020	1.19	1.13	(1.12, 1.15)
1990-2010	1.79	1.63	(1.59, 1.67)
2000-2020	1.55	1.47	(1.43, 1.52)
1990-2020	2.13	1.86	(1.80, 1.91)

Supplementary Figure 17: Supplementary data for the metropolitan zone of Colima-Villa de Álvarez with code 06.1.01. Remoteness values are those of 2020. a) Population difference by grid cell (2020–1990); city centres are denoted as black dots. b) Quantile-quantile plots for the radial population distributions $\rho(s, t_i)$ and $\rho(s, t_j)$ (coloured curves); urban expansion factors Φ_{ij} from t_i to t_j are the estimated slopes (black lines). c) Radial population distribution $\rho(r)$ at remoteness distance r from the city centre. d) Radial population density $\sigma(r)$ at remoteness distance r from the city centre. e) Average population density $\bar{\sigma}(r)$ within disks of remoteness r with the same centre as the city. f) Urban expansion factors and their inter quartile range from the Theil-Sen estimation.

Tapachula, 07.1.01

a)

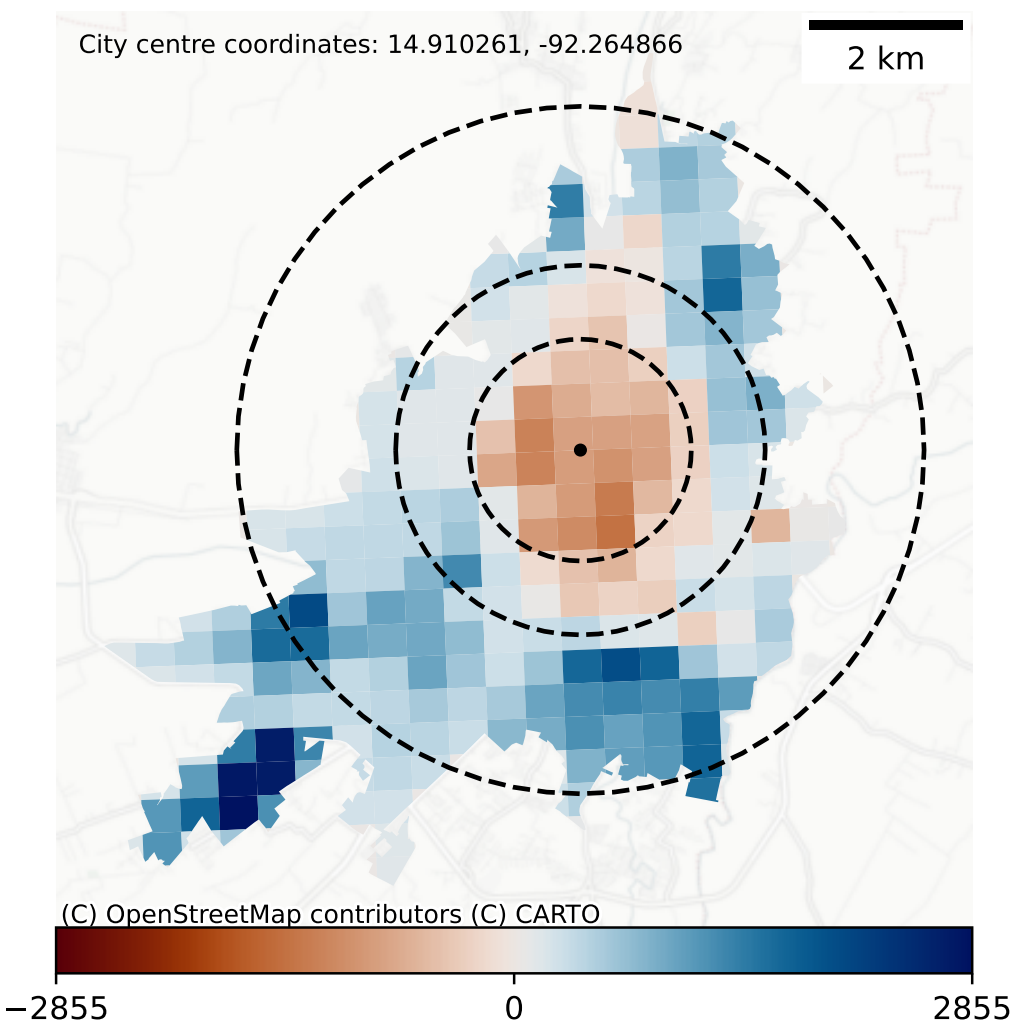

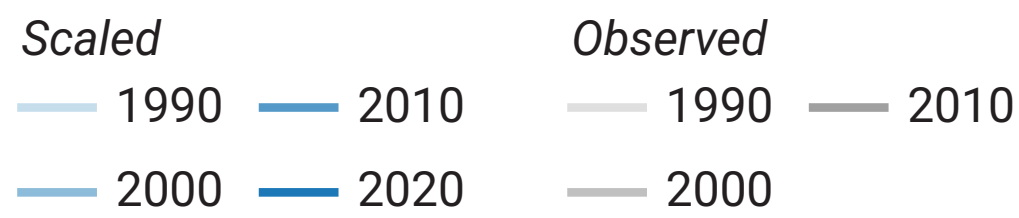

c)

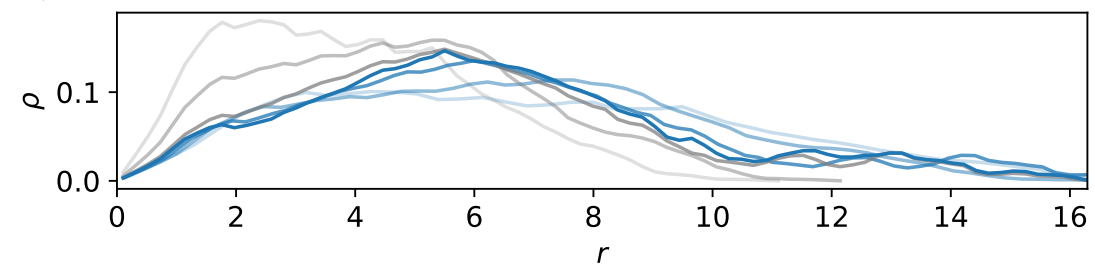

d)

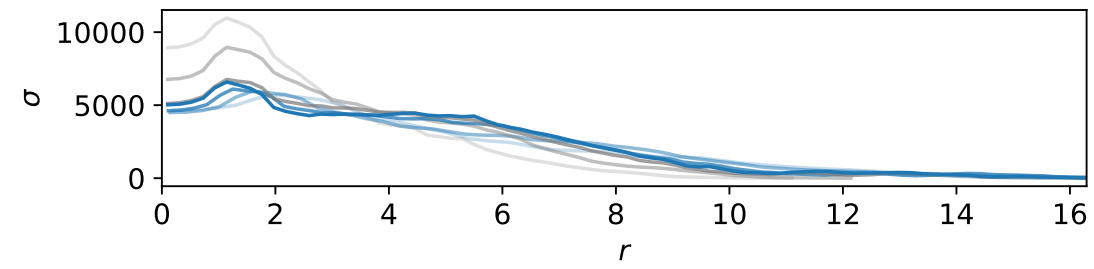

e)

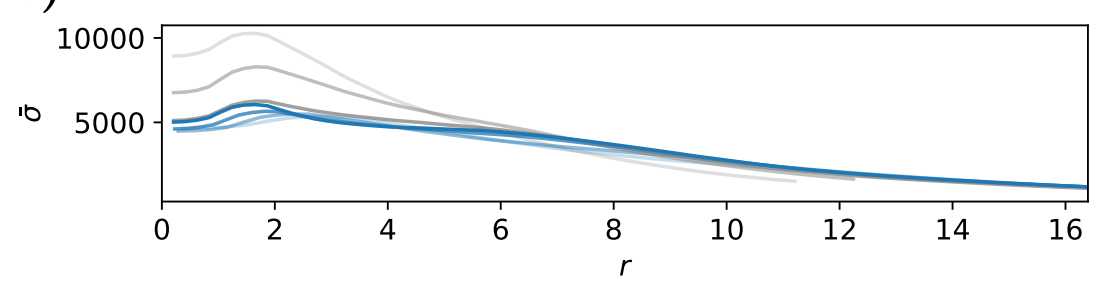

b)

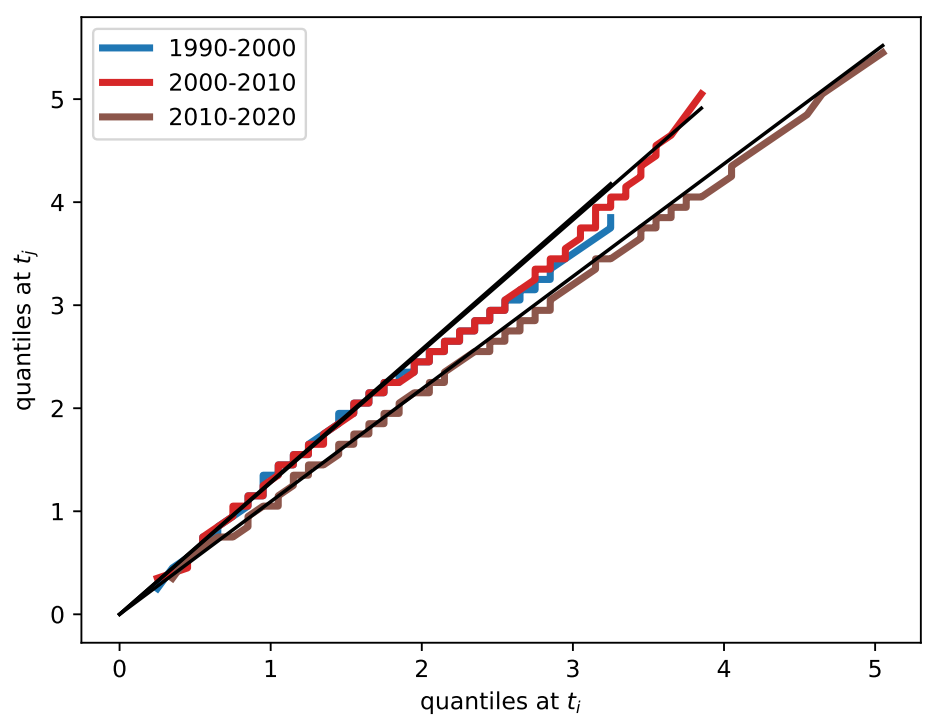

f)

Period (t_i-t_j)	$\frac{P(t_j)}{P(t_i)}$	Φ_{ij}	IQR
1990-2000	1.30	1.29	(1.25, 1.32)
2000-2010	1.20	1.28	(1.24, 1.31)
2010-2020	1.08	1.09	(1.07, 1.11)
1990-2010	1.55	1.64	(1.59, 1.70)
2000-2020	1.29	1.39	(1.36, 1.42)
1990-2020	1.67	1.80	(1.72, 1.85)

Supplementary Figure 18: Supplementary data for the metropolitan zone of Tapachula with code 07.1.01. Remoteness values are those of 2020. a) Population difference by grid cell (2020–1990); city centres are denoted as black dots. b) Quantile-quantile plots for the radial population distributions $\rho(s,t_i)$ and $\rho(s,t_j)$ (coloured curves); urban expansion factors Φ_{ij} from t_i to t_j are the estimated slopes (black lines). c) Radial population distribution $\rho(r)$ at remoteness distance r from the city centre. d) Radial population density $\sigma(r)$ at remoteness distance r from the city centre. e) Average population density $\bar{\sigma}(r)$ within disks of remoteness r with the same centre as the city. f) Urban expansion factors and their inter quartile range from the Theil-Sen estimation.

Tuxtla Gutiérrez, 07.1.02

a)

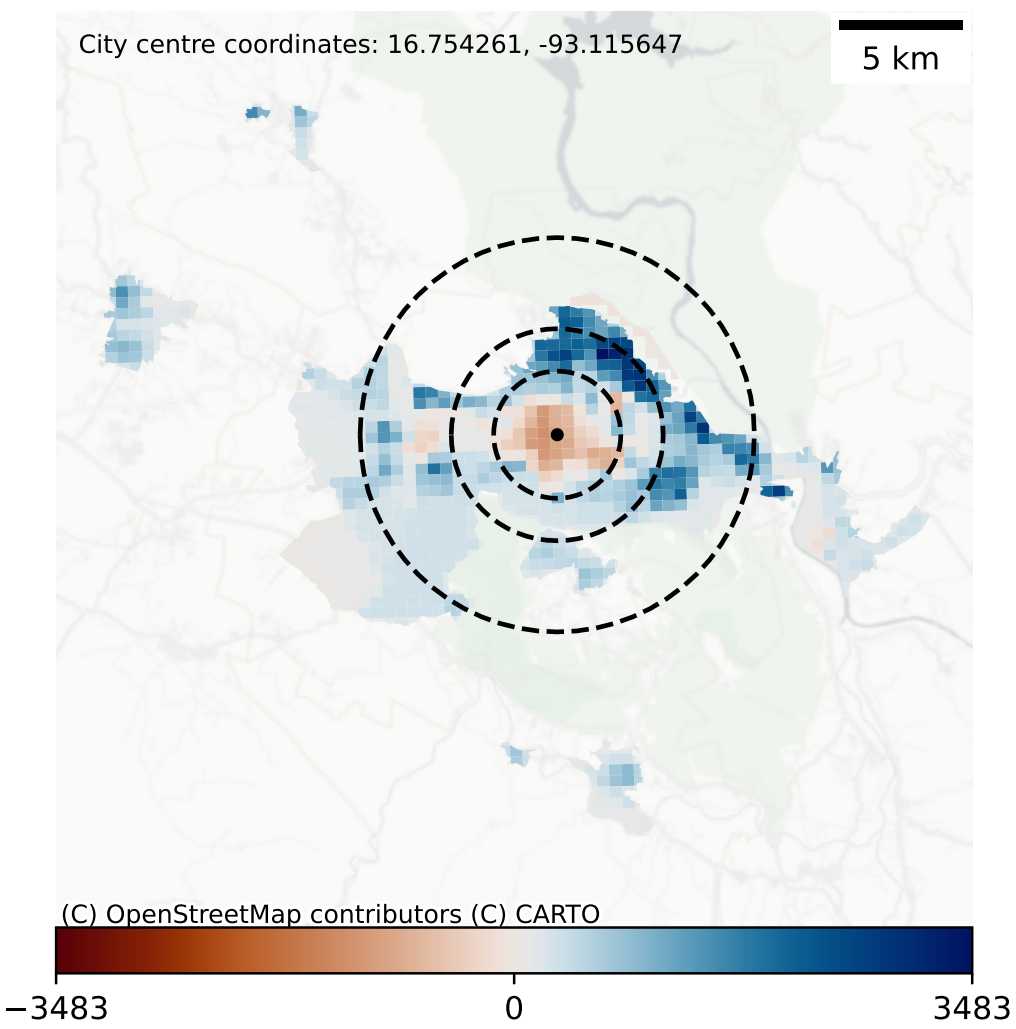

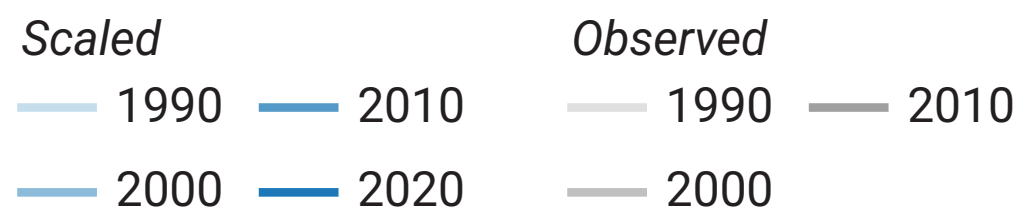

c)

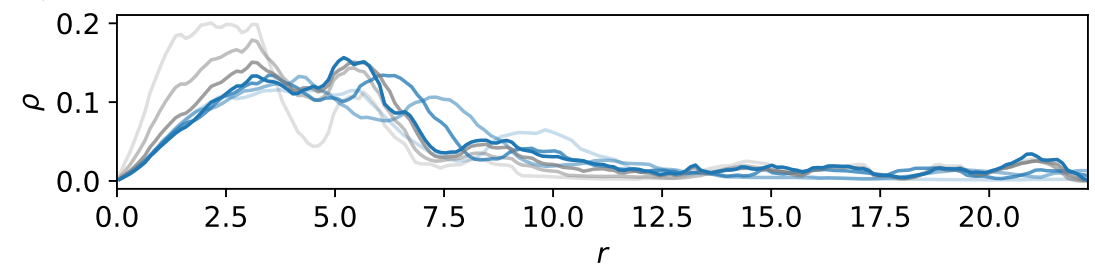

d)

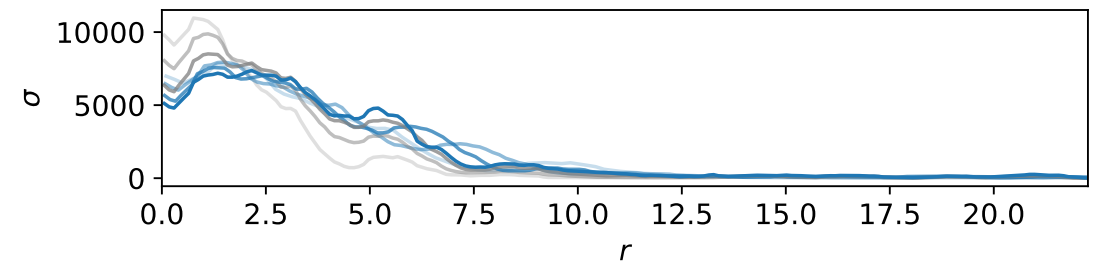

e)

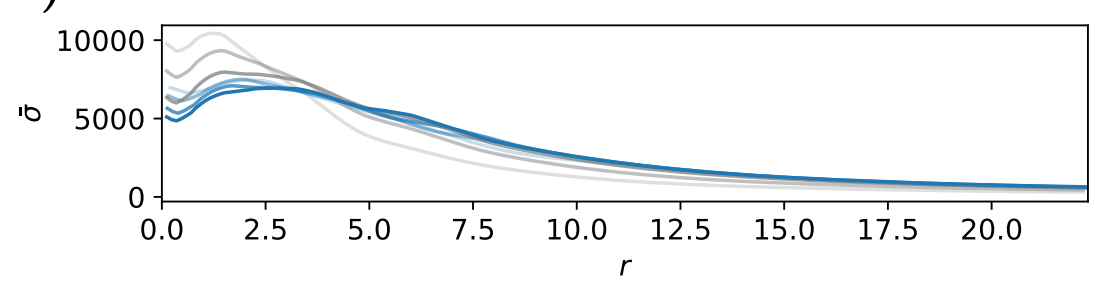

b)

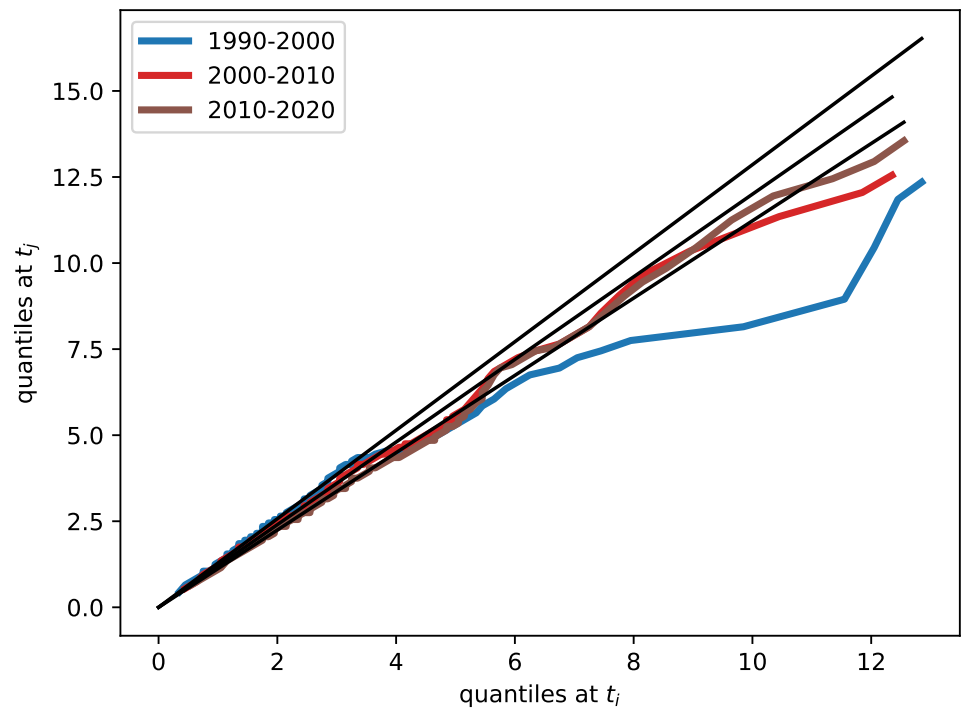

f)

Period (t_i-t_j)	$\frac{P(t_j)}{P(t_i)}$	Φ_{ij}	IQR
1990-2000	1.47	1.29	(1.26, 1.32)
2000-2010	1.30	1.20	(1.18, 1.23)
2010-2020	1.12	1.12	(1.11, 1.14)
1990-2010	1.91	1.55	(1.51, 1.59)
2000-2020	1.46	1.35	(1.32, 1.38)
1990-2020	2.14	1.74	(1.70, 1.80)

Supplementary Figure 19: Supplementary data for the metropolitan zone of Tuxtla Gutiérrez with code 07.1.02. Remoteness values are those of 2020. a) Population difference by grid cell (2020–1990); city centres are denoted as black dots. b) Quantile-quantile plots for the radial population distributions $\rho(s,t_i)$ and $\rho(s,t_j)$ (coloured curves); urban expansion factors Φ_{ij} from t_i to t_j are the estimated slopes (black lines). c) Radial population distribution $\rho(r)$ at remoteness distance r from the city centre. d) Radial population density $\sigma(r)$ at remoteness distance r from the city centre. e) Average population density $\bar{\sigma}(r)$ within disks of remoteness r with the same centre as the city. f) Urban expansion factors and their inter quartile range from the Theil-Sen estimation.

Chihuahua, 08.1.01

a)

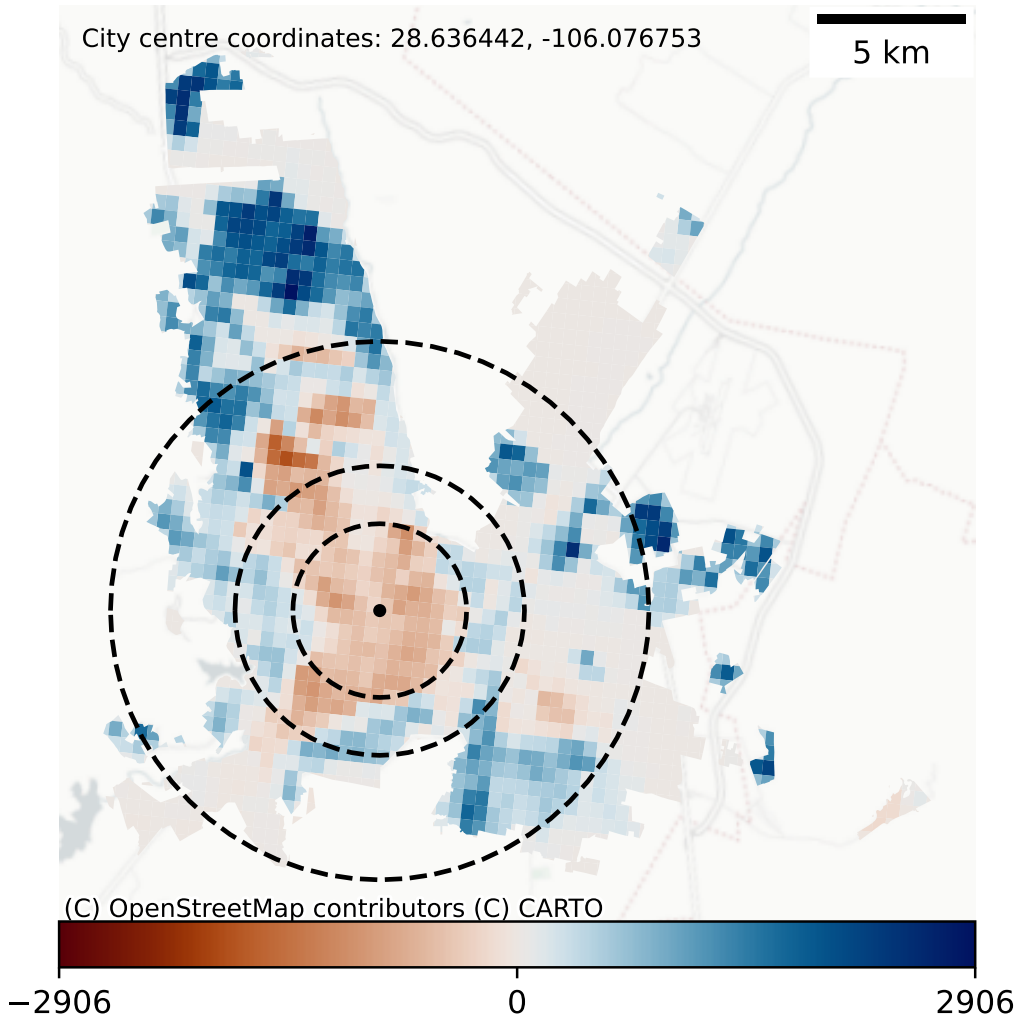

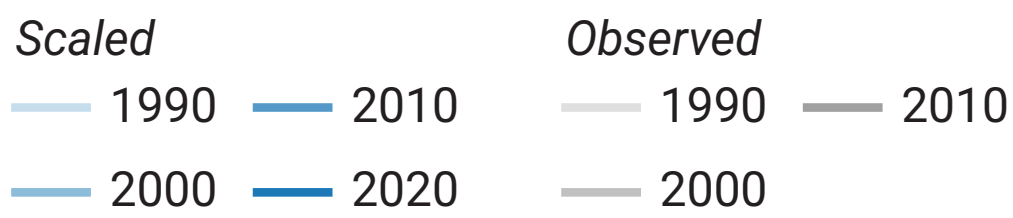

c)

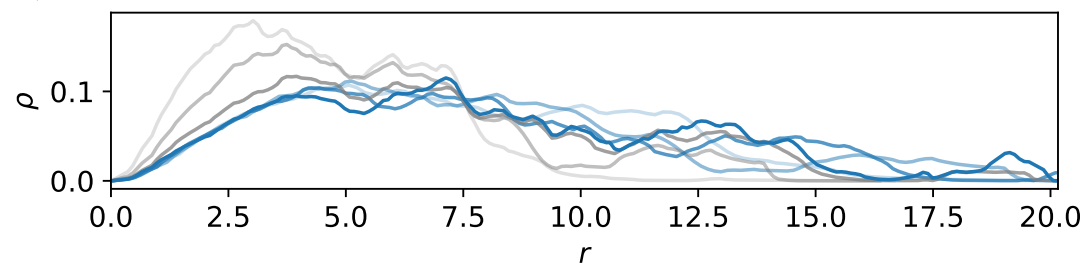

d)

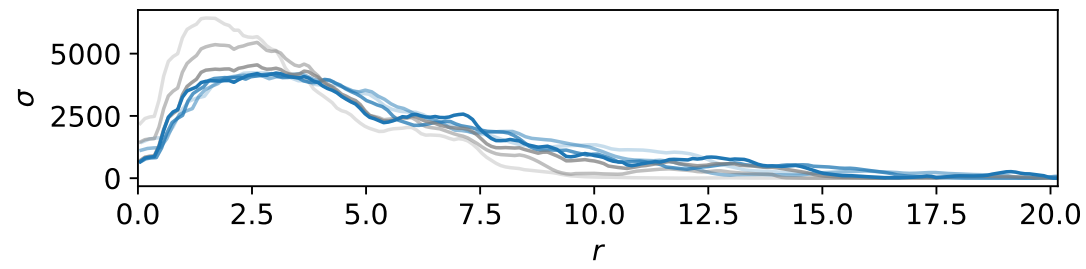

e)

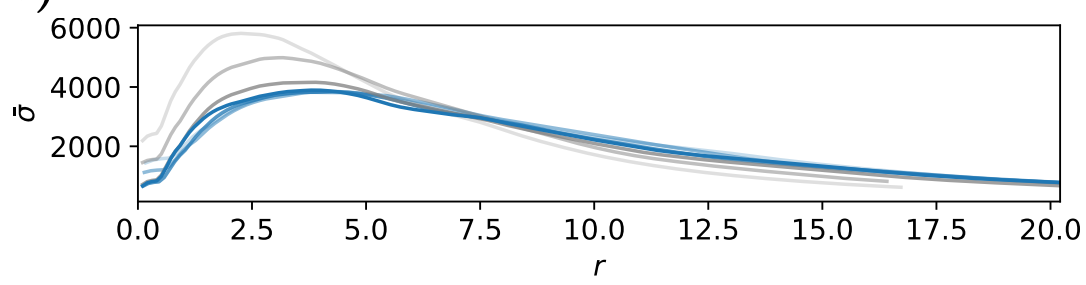

b)

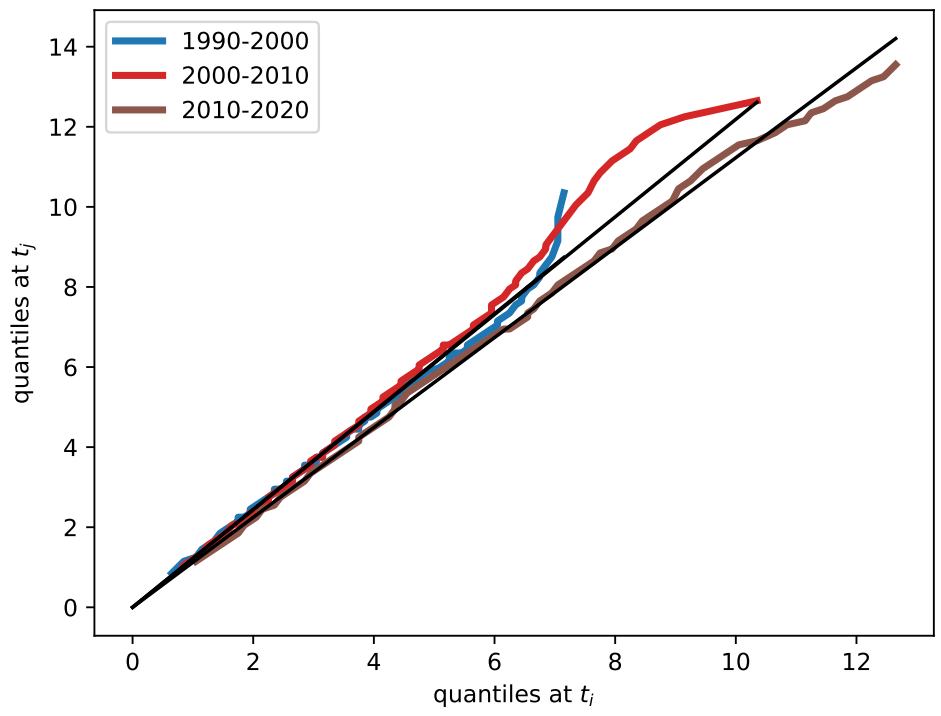

f)

Period (t_i-t_j)	$\frac{P(t_j)}{P(t_i)}$	Φ_{ij}	IQR
1990-2000	1.27	1.22	(1.21, 1.24)
2000-2010	1.24	1.22	(1.20, 1.23)
2010-2020	1.16	1.12	(1.11, 1.14)
1990-2010	1.58	1.49	(1.47, 1.51)
2000-2020	1.44	1.36	(1.34, 1.39)
1990-2020	1.84	1.68	(1.65, 1.72)

Supplementary Figure 20: Supplementary data for the metropolitan zone of Chihuahua with code 08.1.01. Remoteness values are those of 2020. a) Population difference by grid cell (2020–1990); city centres are denoted as black dots. b) Quantile-quantile plots for the radial population distributions $\rho(s, t_i)$ and $\rho(s, t_j)$ (coloured curves); urban expansion factors Φ_{ij} from t_i to t_j are the estimated slopes (black lines). c) Radial population distribution $\rho(r)$ at remoteness distance r from the city centre. d) Radial population density $\sigma(r)$ at remoteness distance r from the city centre. e) Average population density $\bar{\sigma}(r)$ within disks of remoteness r with the same centre as the city. f) Urban expansion factors and their inter quartile range from the Theil-Sen estimation.

Delicias, 08.1.02

a)

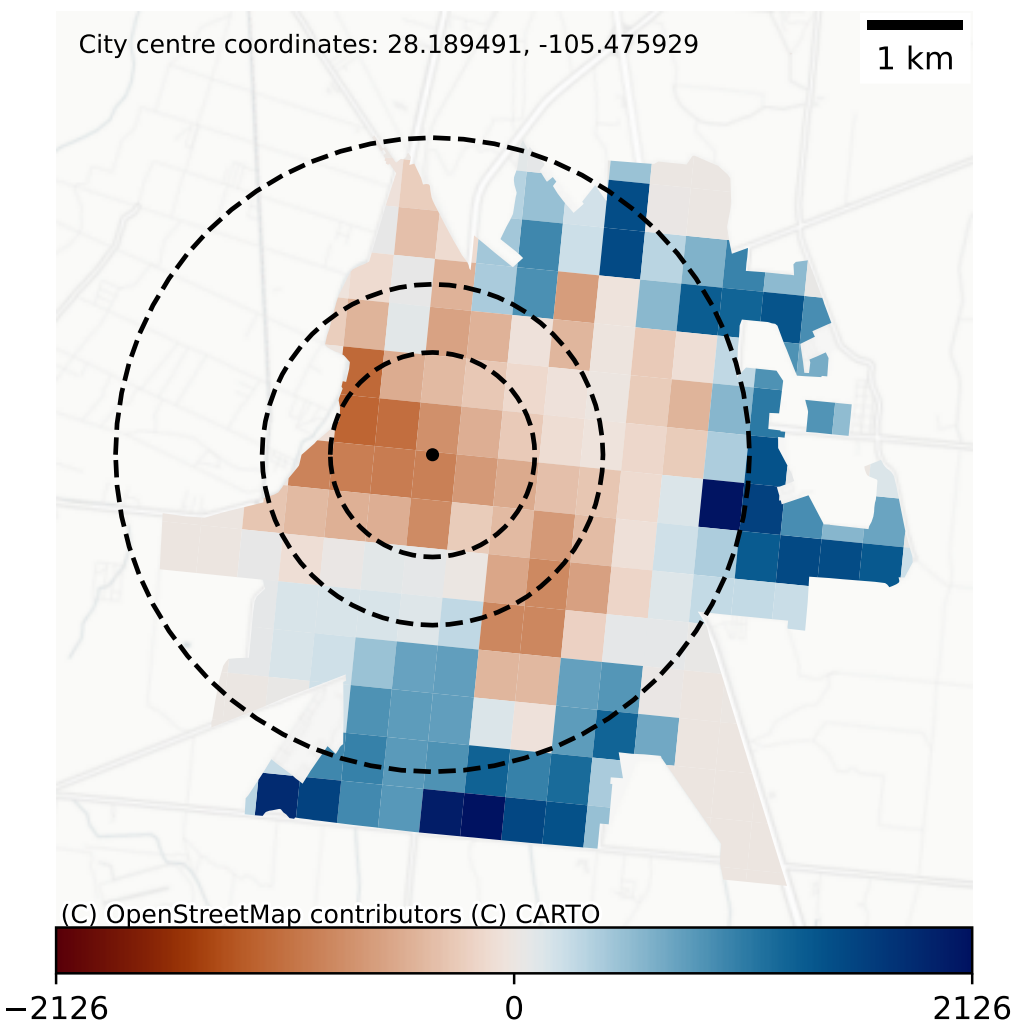

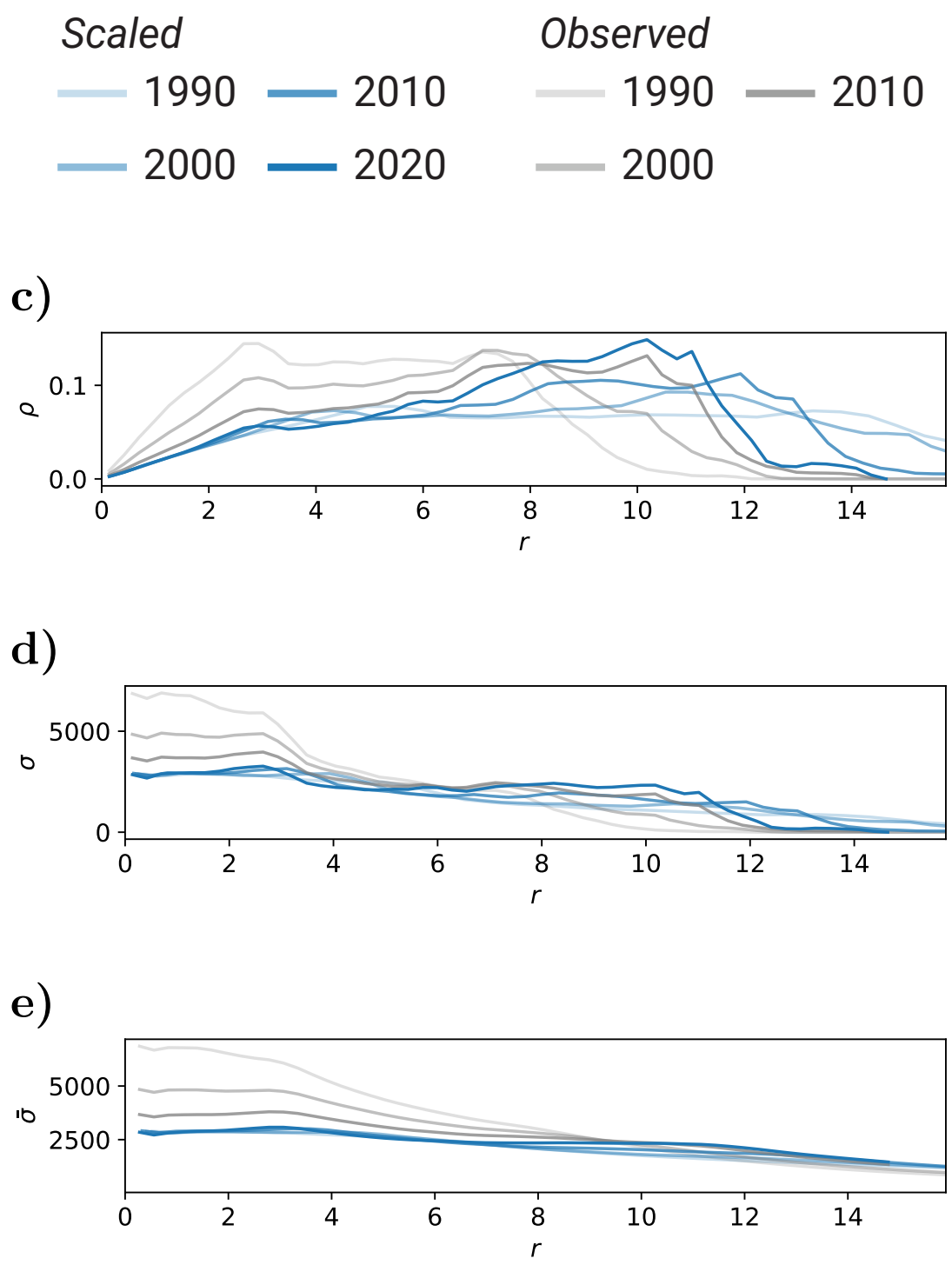

b)

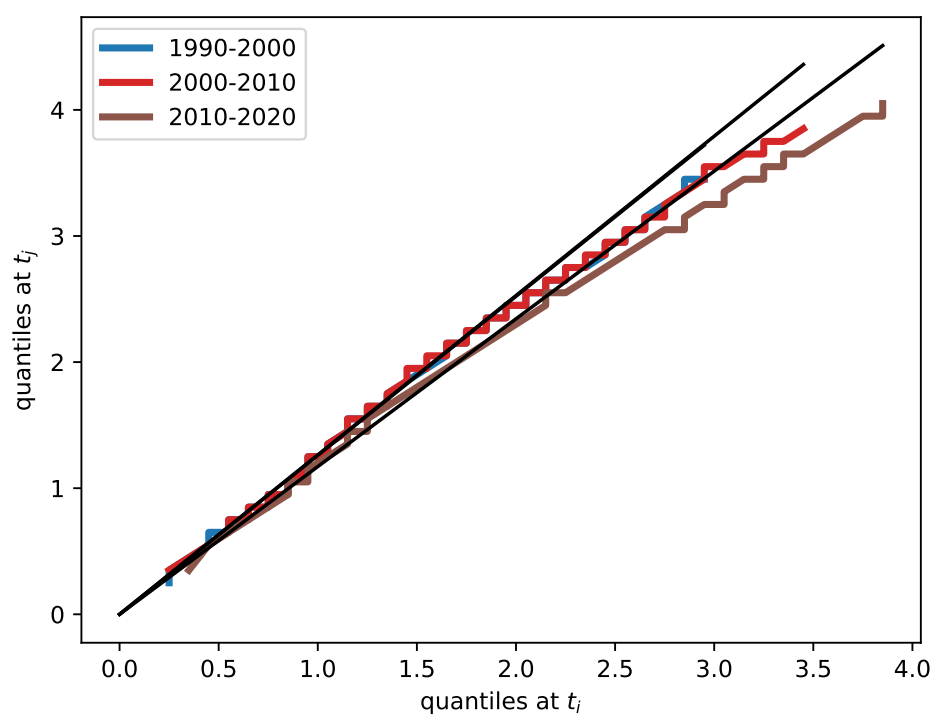

f)

Period (t_i-t_j)	$\frac{P(t_j)}{P(t_i)}$	Φ_{ij}	IQR
1990-2000	1.13	1.26	(1.22, 1.29)
2000-2010	1.20	1.26	(1.22, 1.30)
2010-2020	1.09	1.17	(1.15, 1.19)
1990-2010	1.35	1.59	(1.53, 1.64)
2000-2020	1.30	1.48	(1.44, 1.52)
1990-2020	1.47	1.87	(1.80, 1.93)

Supplementary Figure 21: Supplementary data for the metropolitan zone of Delicias with code 08.1.02. Remoteness values are those of 2020. a) Population difference by grid cell (2020–1990); city centres are denoted as black dots. b) Quantile-quantile plots for the radial population distributions $\rho(s, t_i)$ and $\rho(s, t_j)$ (coloured curves); urban expansion factors Φ_{ij} from t_i to t_j are the estimated slopes (black lines). c) Radial population distribution $\rho(r)$ at remoteness distance r from the city centre. d) Radial population density $\sigma(r)$ at remoteness distance r from the city centre. e) Average population density $\bar{\sigma}(r)$ within disks of remoteness r with the same centre as the city. f) Urban expansion factors and their inter quartile range from the Theil-Sen estimation.

Juárez, 08.2.03

a)

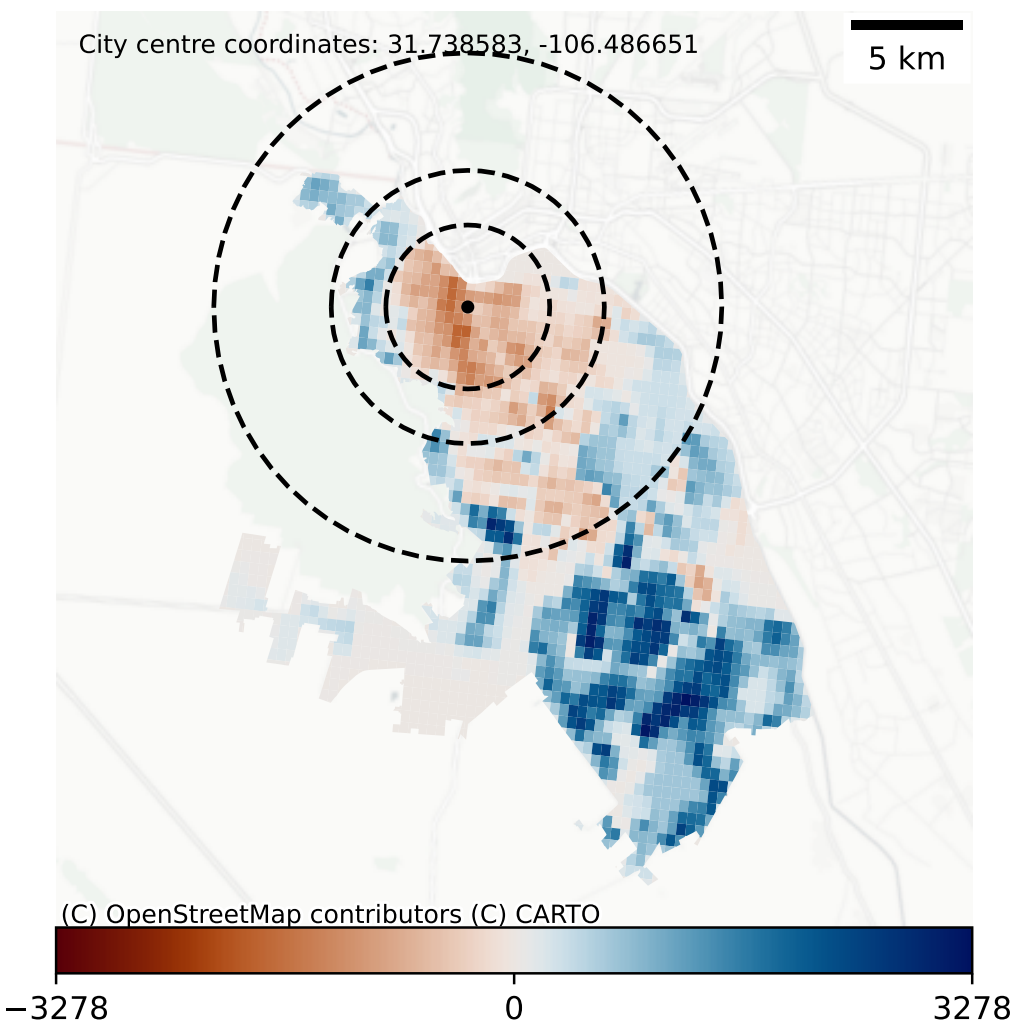

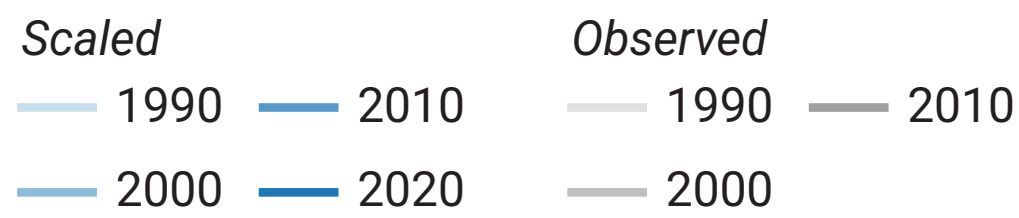

c)

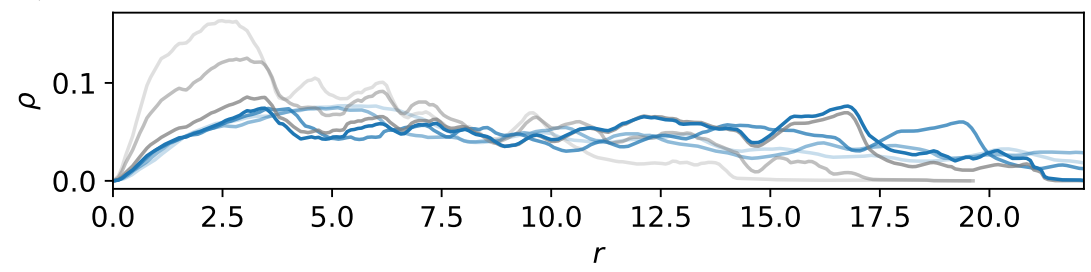

d)

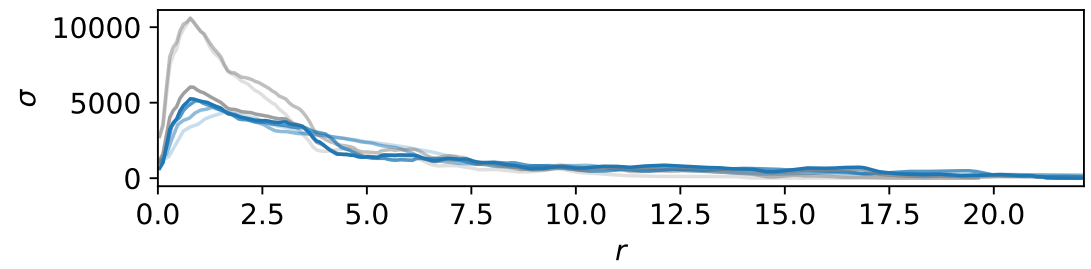

e)

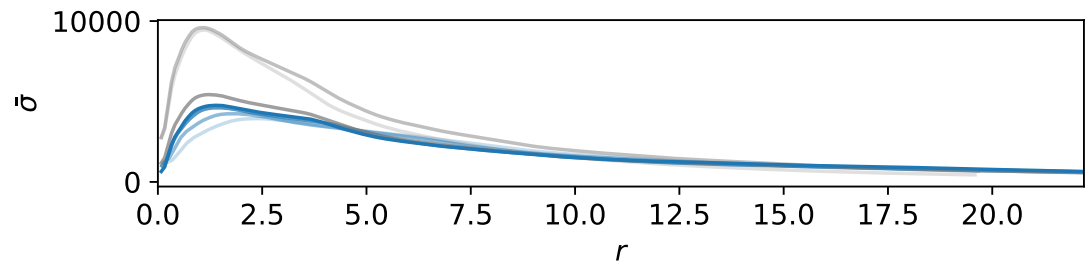

b)

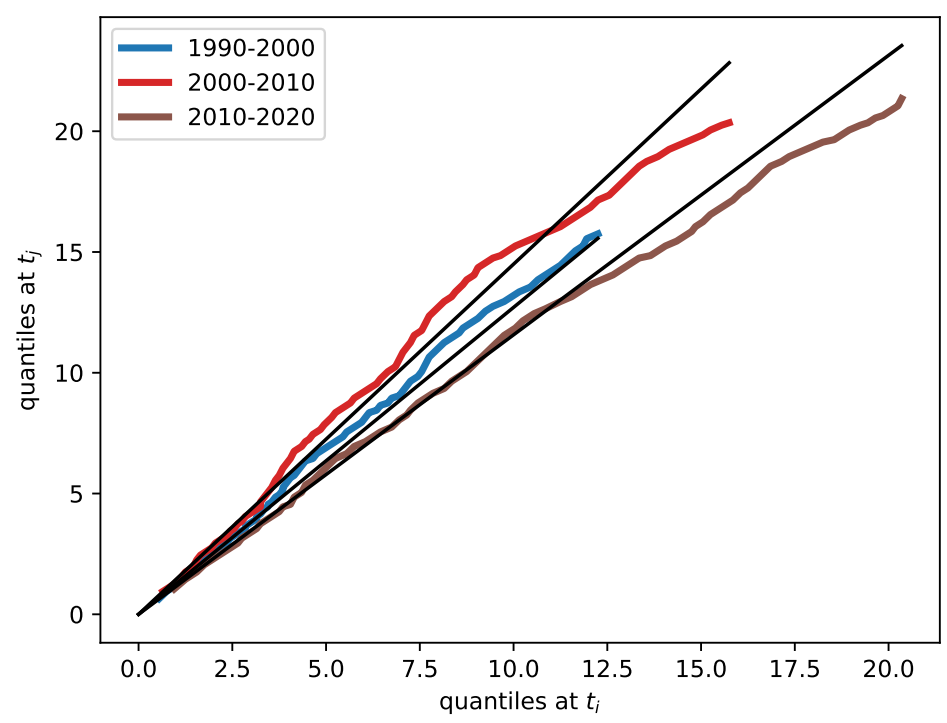

f)

Period (t_i-t_j)	$\frac{P(t_j)}{P(t_i)}$	Φ_{ij}	IQR
1990-2000	1.52	1.27	(1.25, 1.29)
2000-2010	1.09	1.45	(1.40, 1.58)
2010-2020	1.14	1.16	(1.14, 1.17)
1990-2010	1.67	1.83	(1.77, 2.03)
2000-2020	1.24	1.66	(1.61, 1.85)
1990-2020	1.89	2.09	(2.03, 2.38)

Supplementary Figure 22: Supplementary data for the metropolitan zone of Juárez with code 08.2.03. Remoteness values are those of 2020. a) Population difference by grid cell (2020–1990); city centres are denoted as black dots. b) Quantile-quantile plots for the radial population distributions $\rho(s,t_i)$ and $\rho(s,t_j)$ (coloured curves); urban expansion factors Φ_{ij} from t_i to t_j are the estimated slopes (black lines). c) Radial population distribution $\rho(r)$ at remoteness distance r from the city centre. d) Radial population density $\sigma(r)$ at remoteness distance r from the city centre. e) Average population density $\bar{\sigma}(r)$ within disks of remoteness r with the same centre as the city. f) Urban expansion factors and their inter quartile range from the Theil-Sen estimation.

Ciudad de México, 09.1.01

a)

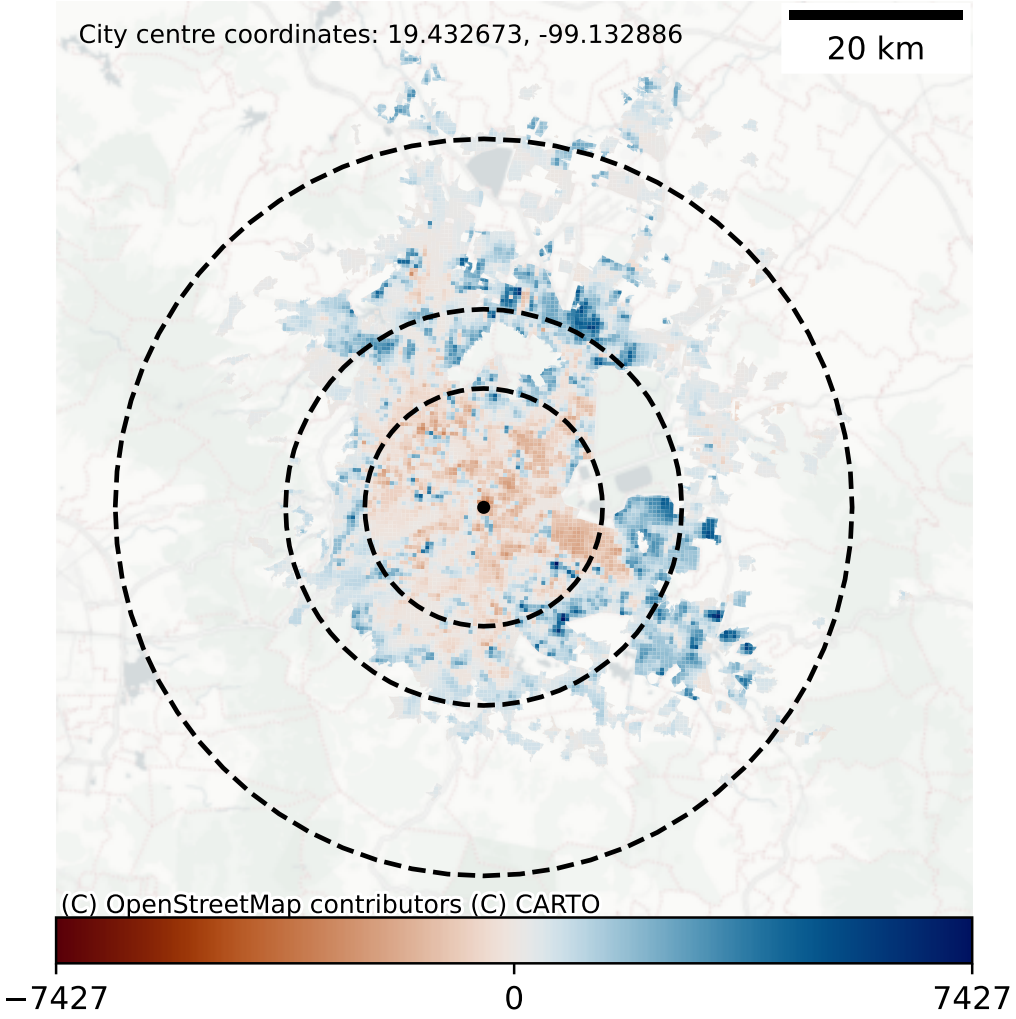

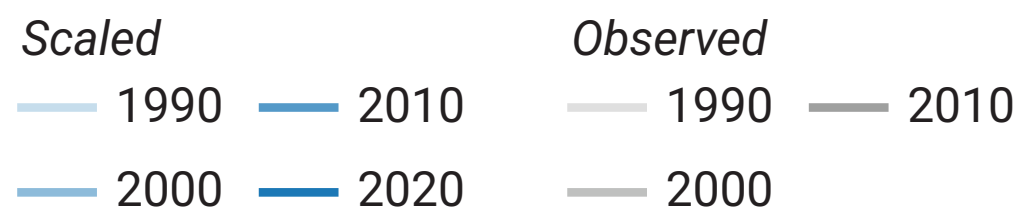

c)

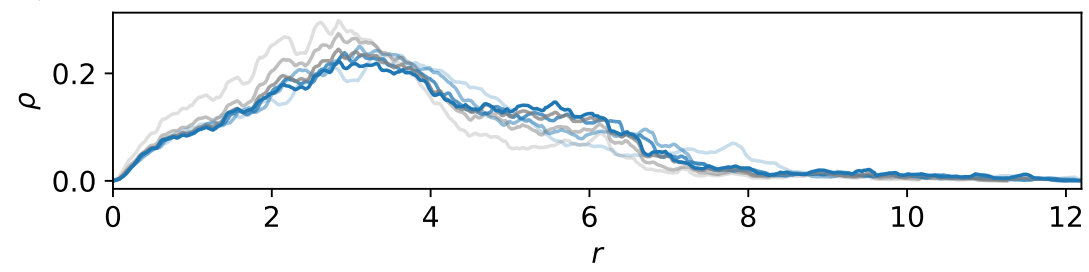

d)

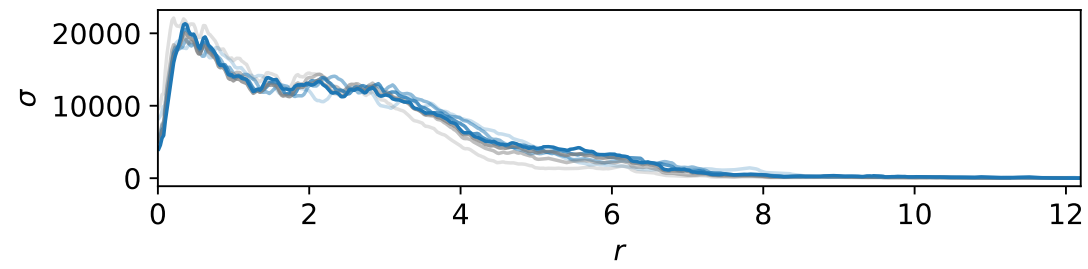

e)

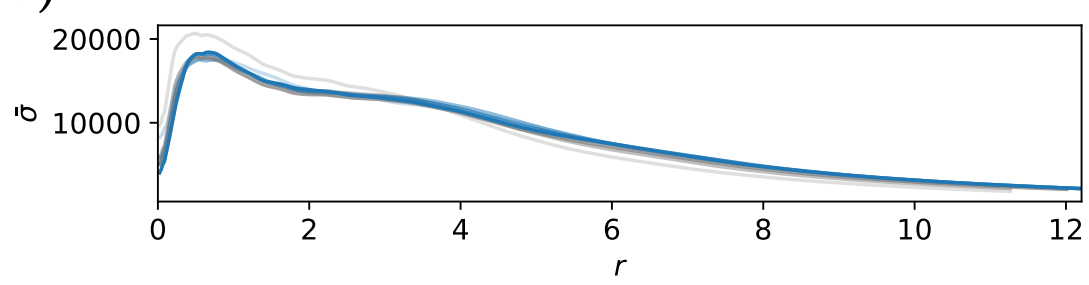

b)

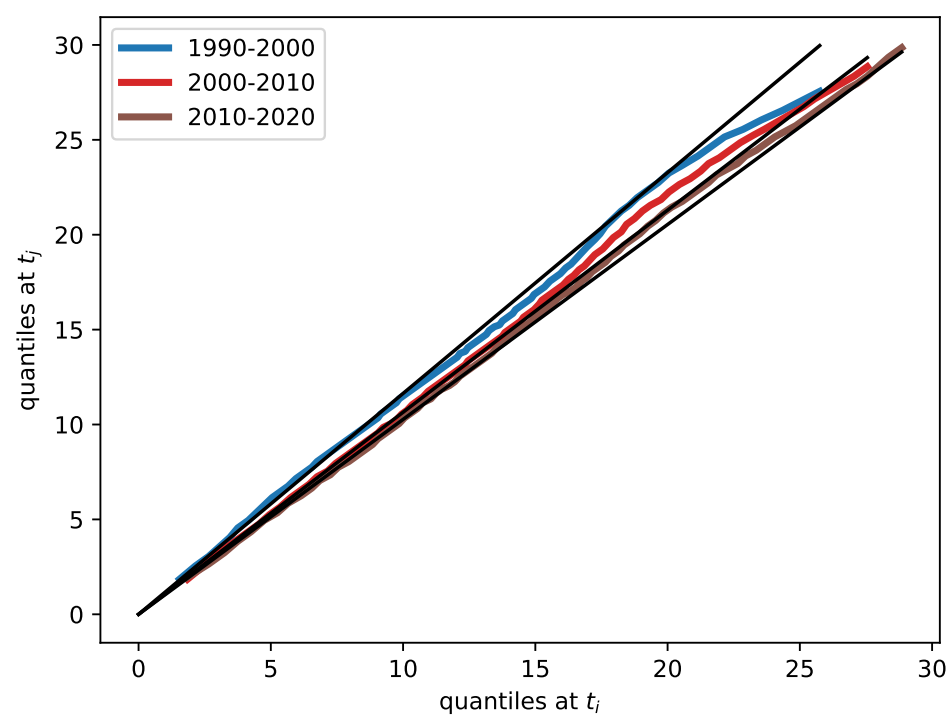

f)

Period (t_i-t_j)	$\frac{P(t_j)}{P(t_i)}$	Φ_{ij}	IQR
1990-2000	1.18	1.16	(1.15, 1.19)
2000-2010	1.08	1.06	(1.06, 1.07)
2010-2020	1.08	1.03	(1.02, 1.03)
1990-2010	1.28	1.23	(1.22, 1.26)
2000-2020	1.17	1.09	(1.08, 1.10)
1990-2020	1.39	1.26	(1.26, 1.28)

Supplementary Figure 23: Supplementary data for the metropolitan zone of Ciudad de México with code 09.1.01. Remoteness values are those of 2020. a) Population difference by grid cell (2020–1990); city centres are denoted as black dots. b) Quantile-quantile plots for the radial population distributions $\rho(s, t_i)$ and $\rho(s, t_j)$ (coloured curves); urban expansion factors Φ_{ij} from t_i to t_j are the estimated slopes (black lines). c) Radial population distribution $\rho(r)$ at remoteness distance r from the city centre. d) Radial population density $\sigma(r)$ at remoteness distance r from the city centre. e) Average population density $\bar{\sigma}(r)$ within disks of remoteness r with the same centre as the city. f) Urban expansion factors and their inter quartile range from the Theil-Sen estimation.

Durango, 10.2.01

a)

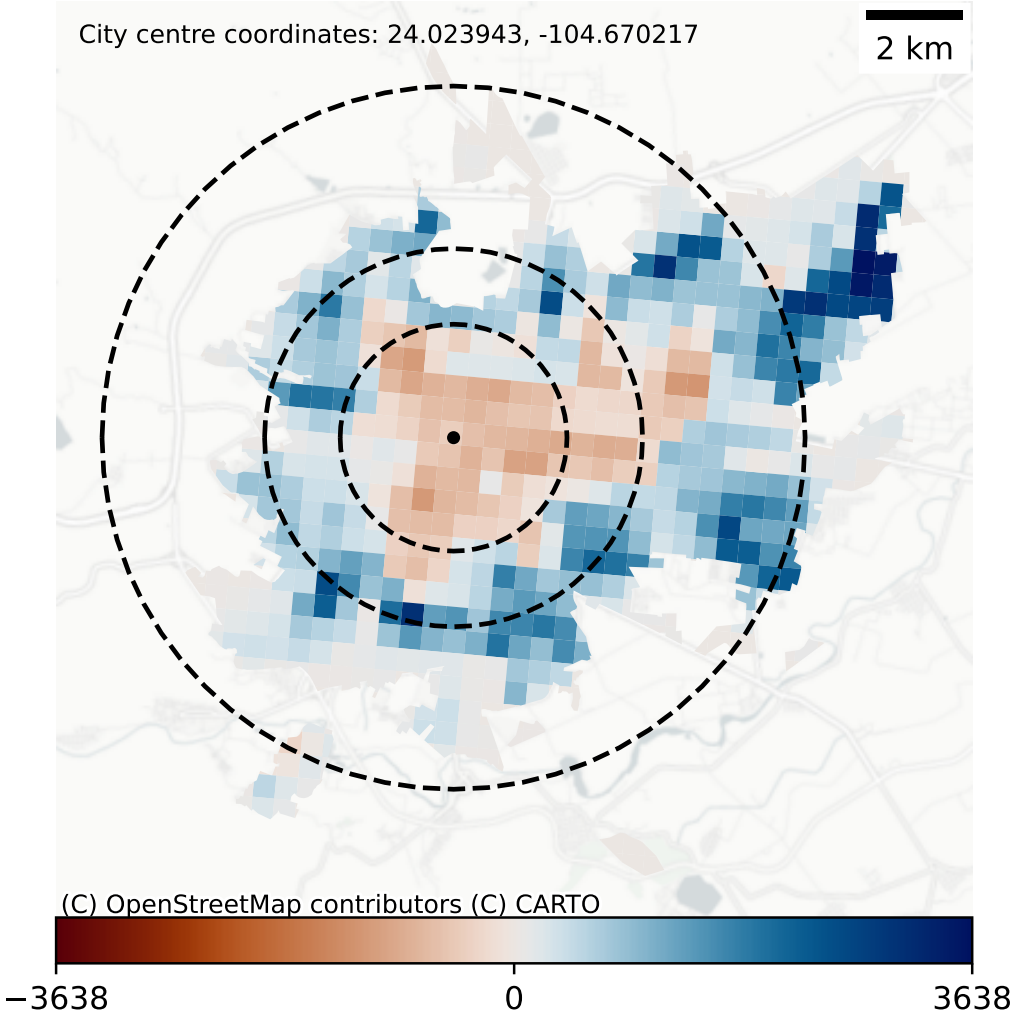

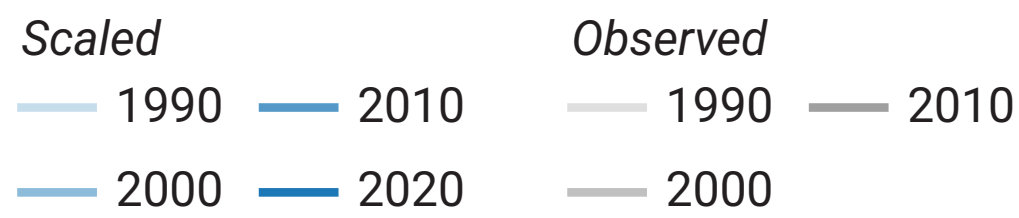

c)

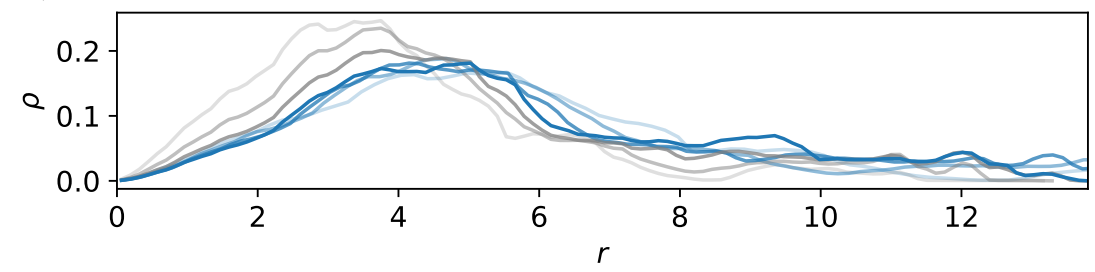

d)

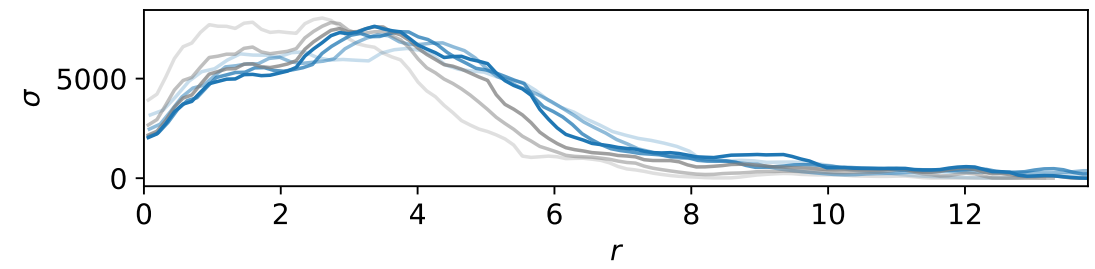

e)

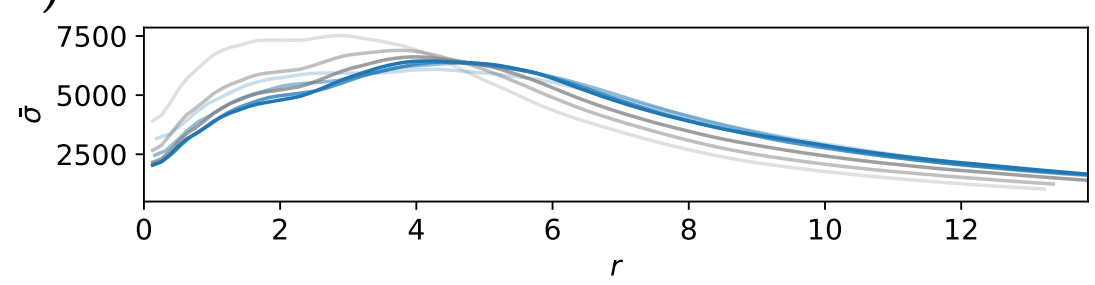

b)

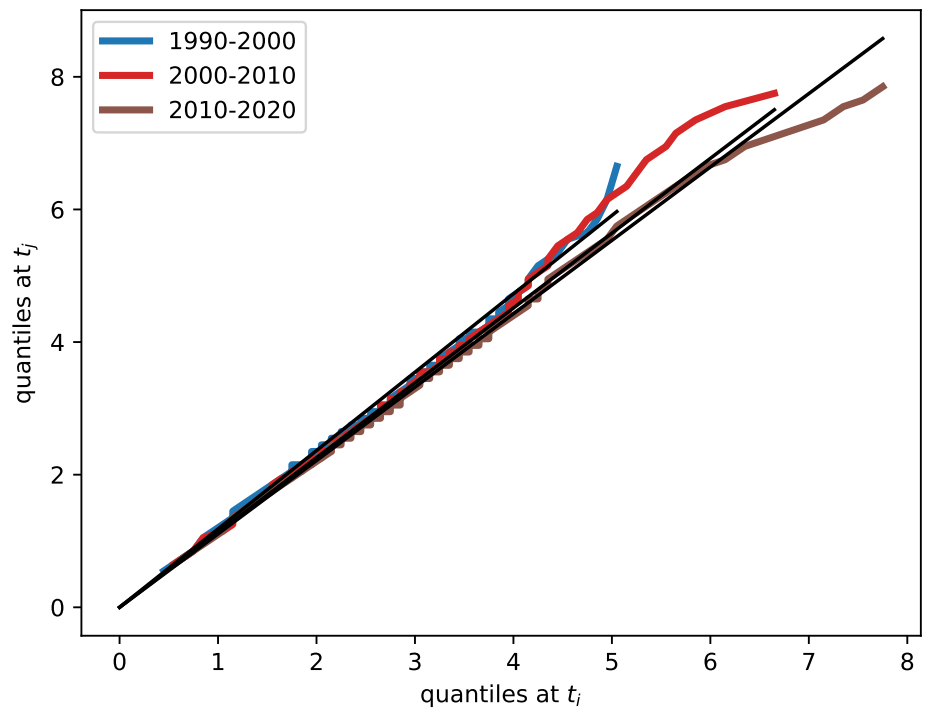

f)

Period (t_i-t_j)	$\frac{P(t_j)}{P(t_i)}$	Φ_{ij}	IQR
1990-2000	1.23	1.18	(1.16, 1.21)
2000-2010	1.21	1.13	(1.11, 1.15)
2010-2020	1.18	1.11	(1.10, 1.13)
1990-2010	1.49	1.33	(1.31, 1.38)
2000-2020	1.44	1.26	(1.23, 1.28)
1990-2020	1.76	1.47	(1.44, 1.53)

Supplementary Figure 24: Supplementary data for the metropolitan zone of Durango with code 10.2.01. Remoteness values are those of 2020. a) Population difference by grid cell (2020–1990); city centres are denoted as black dots. b) Quantile-quantile plots for the radial population distributions $\rho(s, t_i)$ and $\rho(s, t_j)$ (coloured curves); urban expansion factors Φ_{ij} from t_i to t_j are the estimated slopes (black lines). c) Radial population distribution $\rho(r)$ at remoteness distance r from the city centre. d) Radial population density $\sigma(r)$ at remoteness distance r from the city centre. e) Average population density $\bar{\sigma}(r)$ within disks of remoteness r with the same centre as the city. f) Urban expansion factors and their inter quartile range from the Theil-Sen estimation.

Celaya, 11.1.01

a)

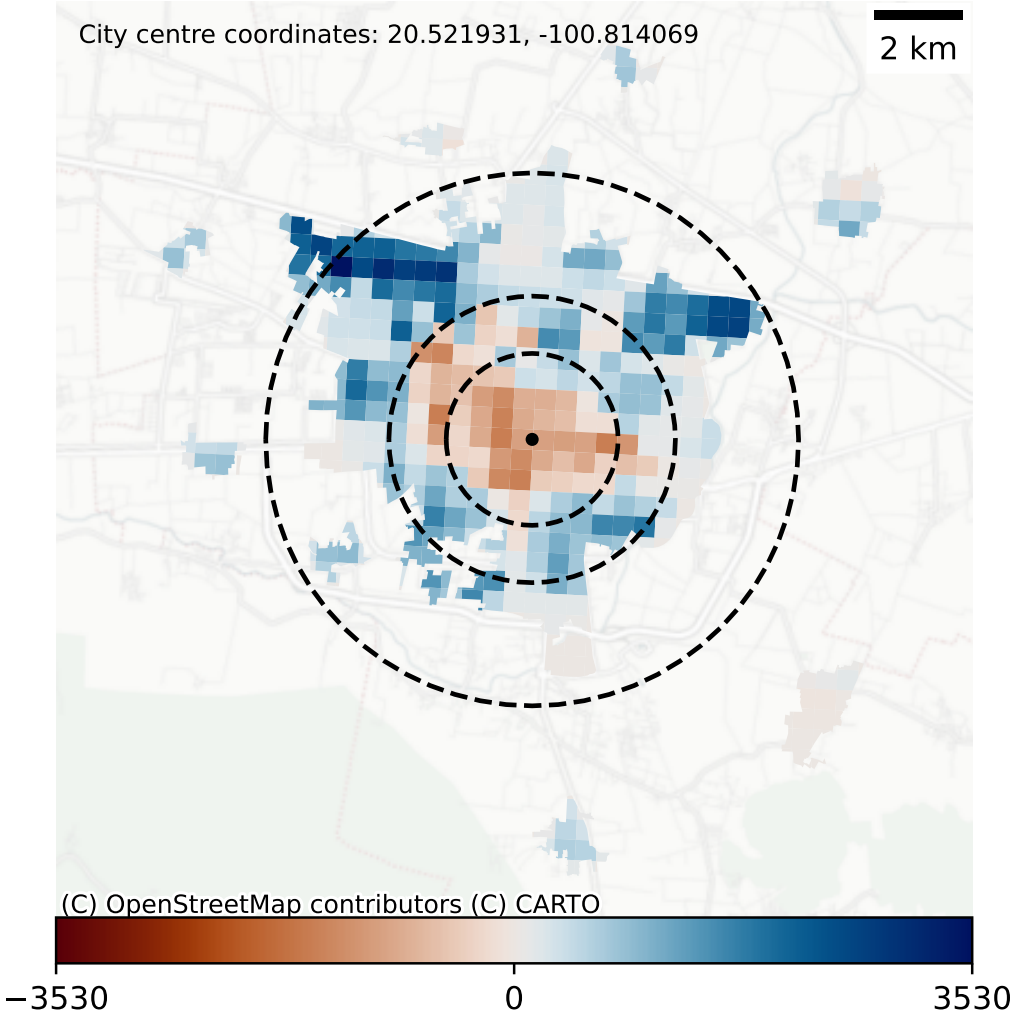

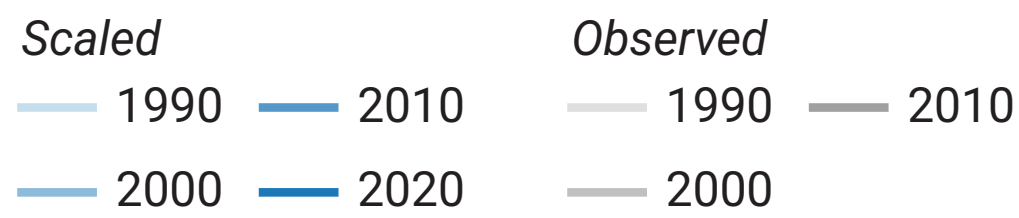

c)

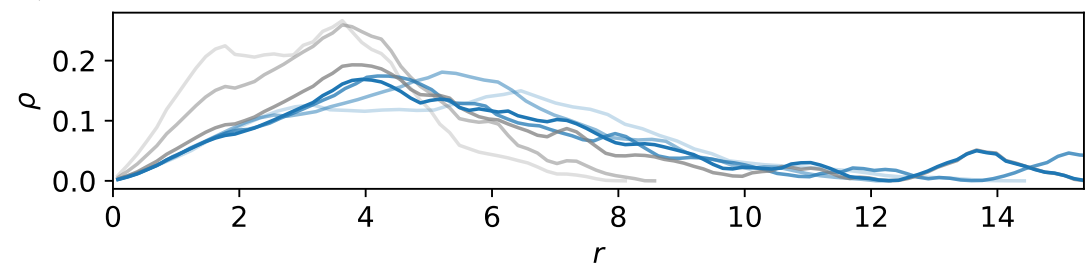

d)

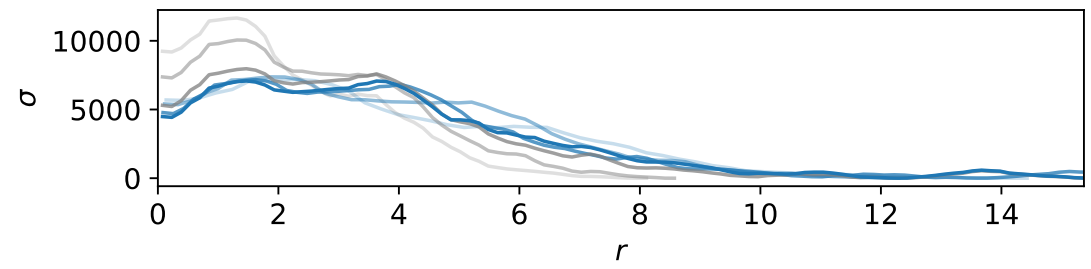

e)

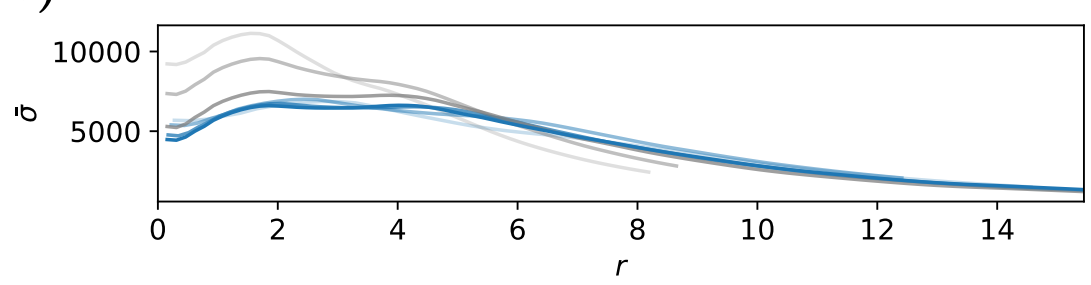

b)

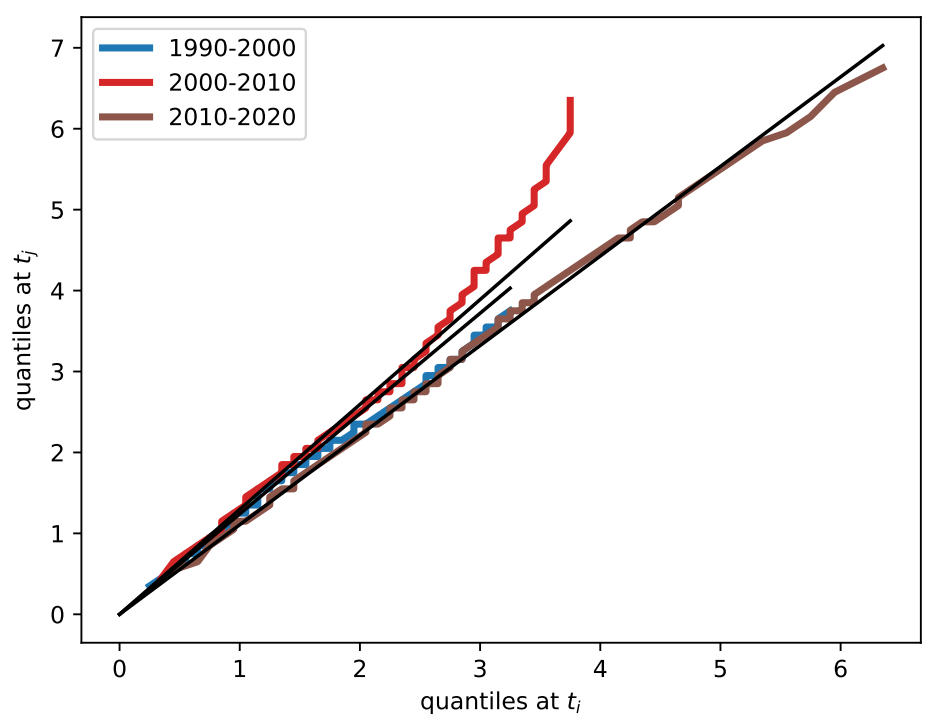

f)

Period (t_i-t_j)	$\frac{P(t_j)}{P(t_i)}$	Φ_{ij}	IQR
1990-2000	1.29	1.24	(1.21, 1.28)
2000-2010	1.37	1.30	(1.27, 1.32)
2010-2020	1.10	1.11	(1.09, 1.13)
1990-2010	1.77	1.63	(1.57, 1.67)
2000-2020	1.51	1.44	(1.40, 1.48)
1990-2020	1.95	1.80	(1.74, 1.86)

Supplementary Figure 25: Supplementary data for the metropolitan zone of Celaya with code 11.1.01. Remoteness values are those of 2020. a) Population difference by grid cell (2020–1990); city centres are denoted as black dots. b) Quantile-quantile plots for the radial population distributions $\rho(s,t_i)$ and $\rho(s,t_j)$ (coloured curves); urban expansion factors Φ_{ij} from t_i to t_j are the estimated slopes (black lines). c) Radial population distribution $\rho(r)$ at remoteness distance r from the city centre. d) Radial population density $\sigma(r)$ at remoteness distance r from the city centre. e) Average population density $\bar{\sigma}(r)$ within disks of remoteness r with the same centre as the city. f) Urban expansion factors and their inter quartile range from the Theil-Sen estimation.

León, 11.1.02

a)

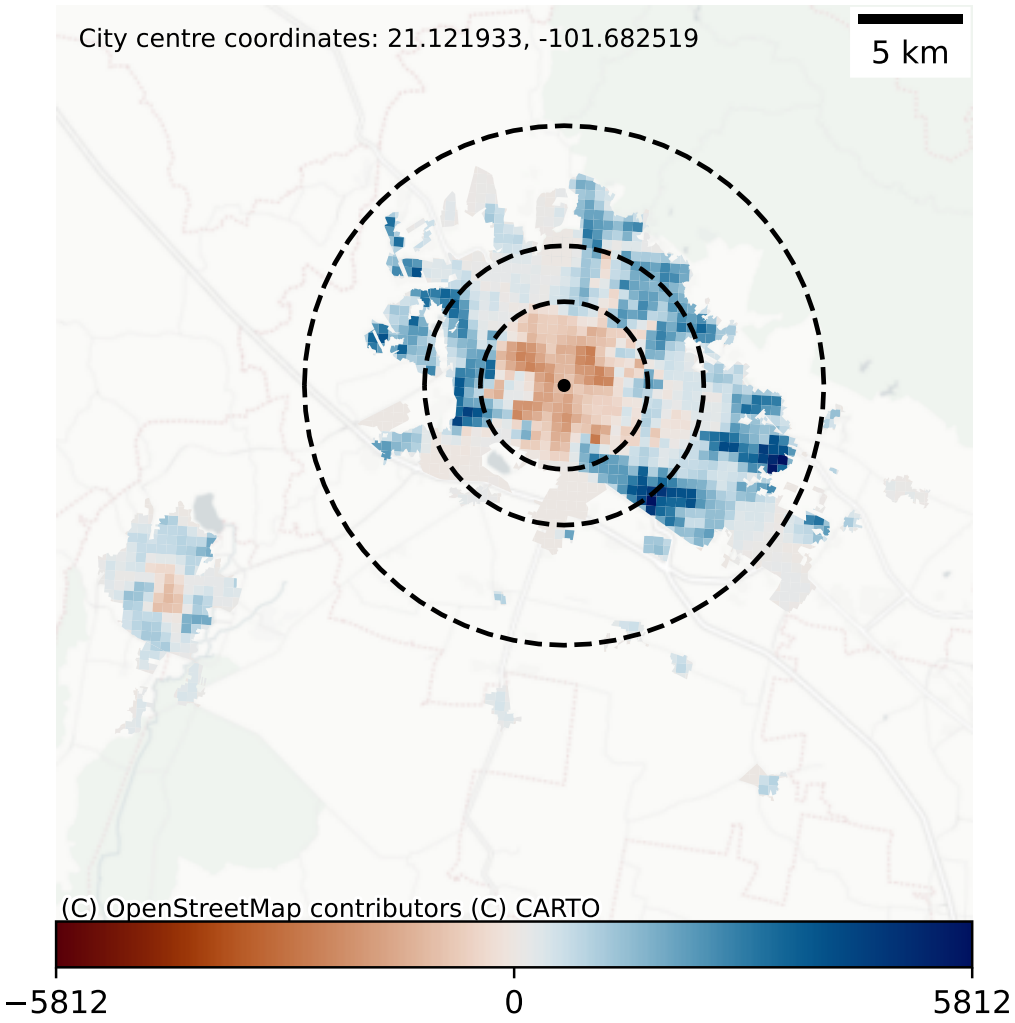

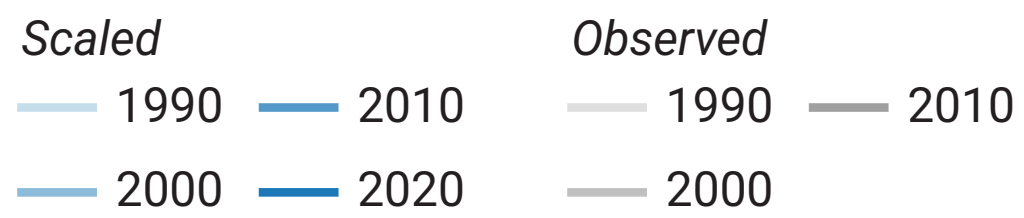

c)

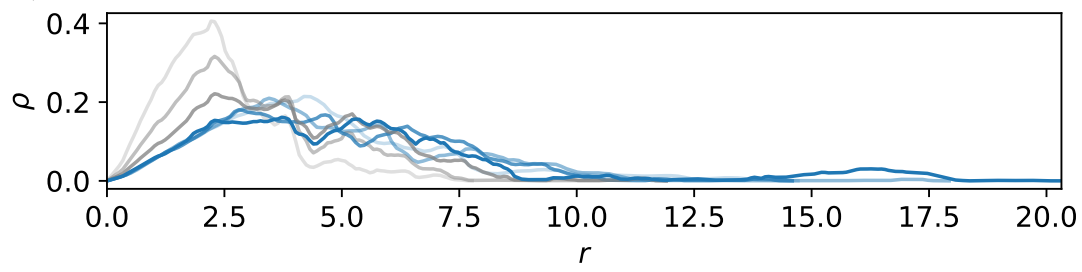

d)

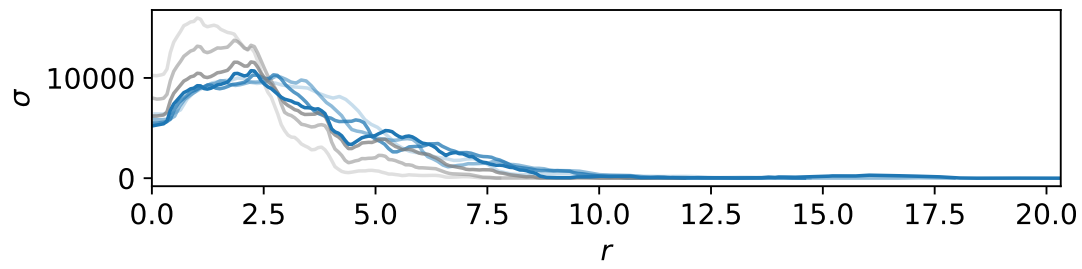

e)

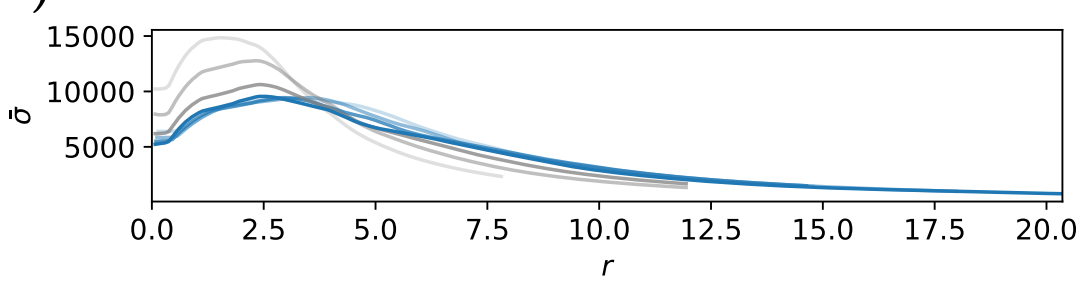

b)

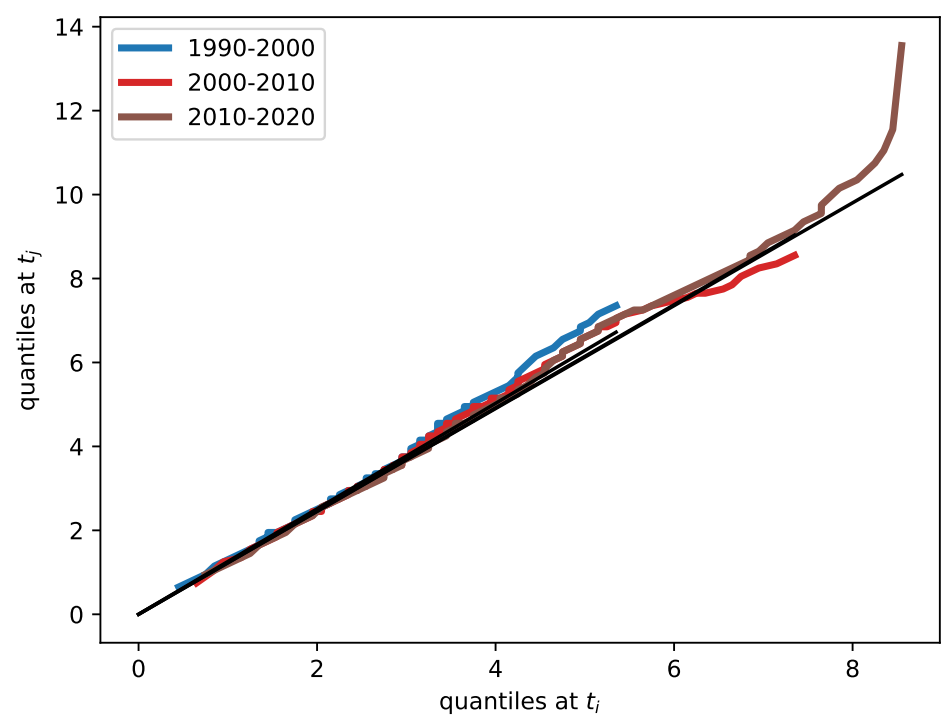

f)

Period (t_i-t_j)	$\frac{P(t_j)}{P(t_i)}$	Φ_{ij}	IQR
1990-2000	1.34	1.26	(1.24, 1.28)
2000-2010	1.26	1.23	(1.21, 1.25)
2010-2020	1.33	1.23	(1.20, 1.25)
1990-2010	1.68	1.55	(1.52, 1.58)
2000-2020	1.68	1.51	(1.47, 1.54)
1990-2020	2.24	1.91	(1.87, 1.94)

Supplementary Figure 26: Supplementary data for the metropolitan zone of León with code 11.1.02. Remoteness values are those of 2020. a) Population difference by grid cell (2020–1990); city centres are denoted as black dots. b) Quantile-quantile plots for the radial population distributions $\rho(s, t_i)$ and $\rho(s, t_j)$ (coloured curves); urban expansion factors Φ_{ij} from t_i to t_j are the estimated slopes (black lines). c) Radial population distribution $\rho(r)$ at remoteness distance r from the city centre. d) Radial population density $\sigma(r)$ at remoteness distance r from the city centre. e) Average population density $\bar{\sigma}(r)$ within disks of remoteness r with the same centre as the city. f) Urban expansion factors and their inter quartile range from the Theil-Sen estimation.

Guanajuato, 11.2.03

a)

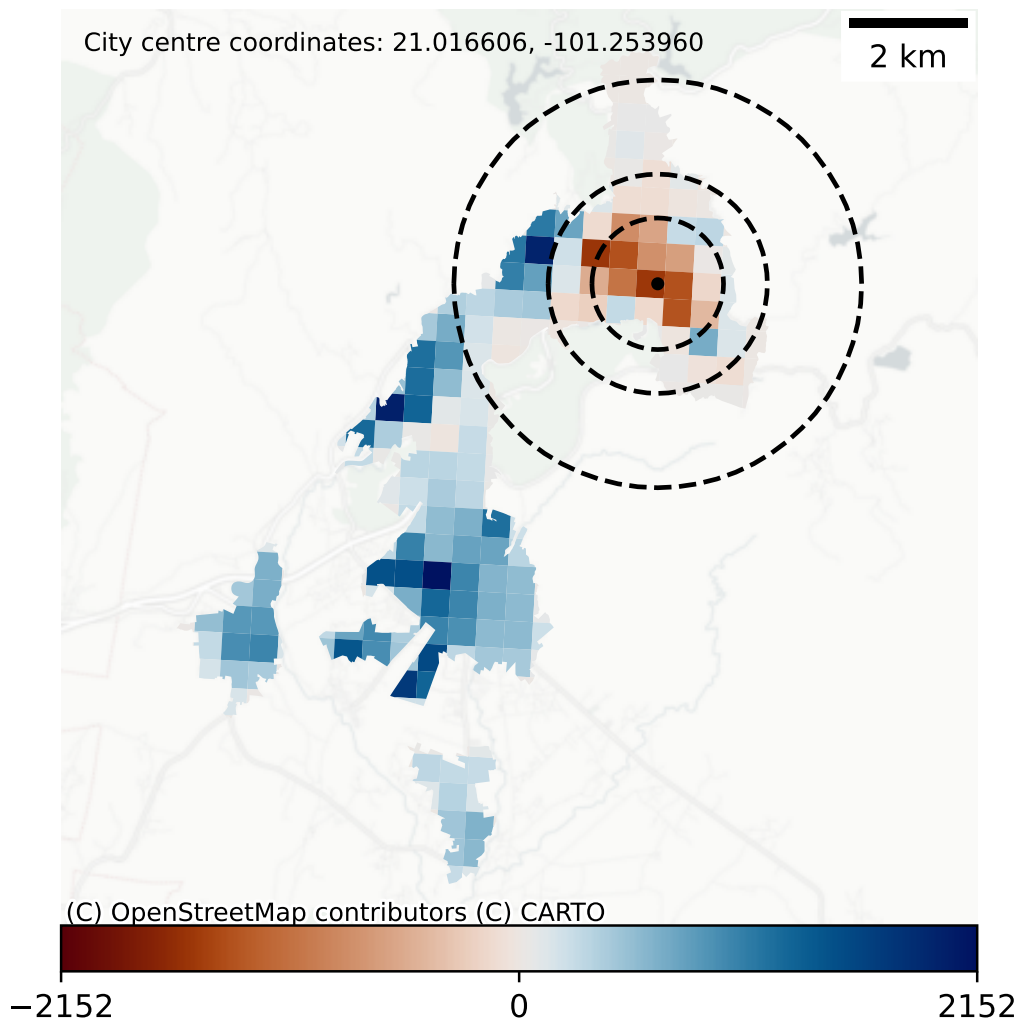

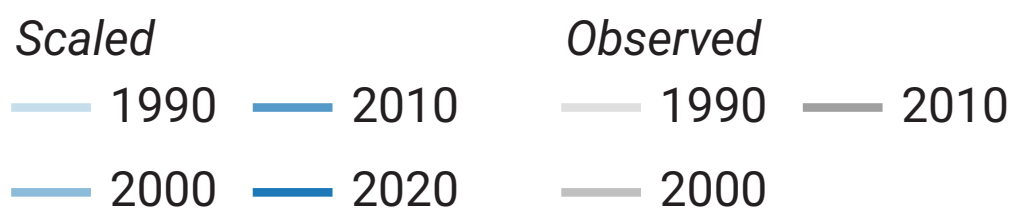

c)

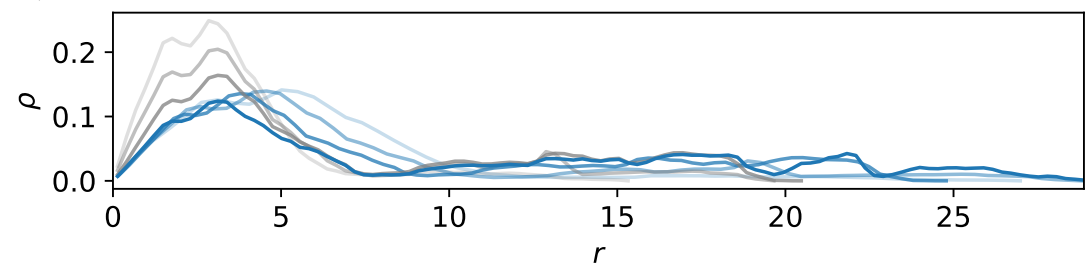

d)

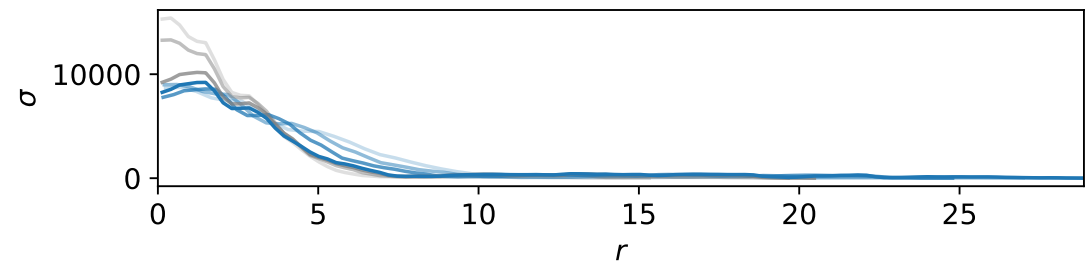

e)

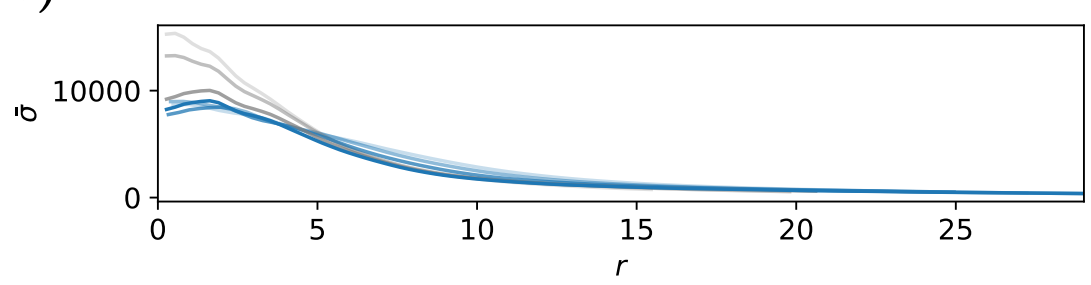

b)

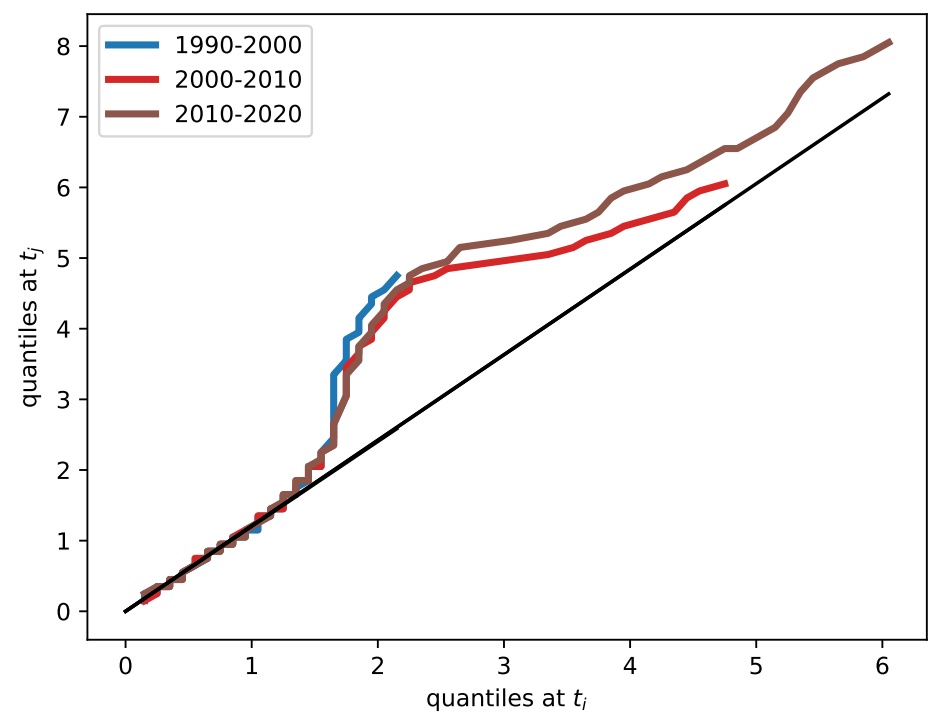

f)

Period (t_i-t_j)	$\frac{P(t_j)}{P(t_i)}$	Φ_{ij}	IQR
1990-2000	1.21	1.20	(1.14, 1.27)
2000-2010	1.18	1.21	(1.18, 1.26)
2010-2020	1.24	1.21	(1.17, 1.24)
1990-2010	1.42	1.45	(1.37, 1.53)
2000-2020	1.45	1.46	(1.40, 1.53)
1990-2020	1.76	1.75	(1.67, 1.82)

Supplementary Figure 27: Supplementary data for the metropolitan zone of Guanajuato with code 11.2.03. Remoteness values are those of 2020. a) Population difference by grid cell (2020–1990); city centres are denoted as black dots. b) Quantile-quantile plots for the radial population distributions $\rho(s, t_i)$ and $\rho(s, t_j)$ (coloured curves); urban expansion factors Φ_{ij} from t_i to t_j are the estimated slopes (black lines). c) Radial population distribution $\rho(r)$ at remoteness distance r from the city centre. d) Radial population density $\sigma(r)$ at remoteness distance r from the city centre. e) Average population density $\bar{\sigma}(r)$ within disks of remoteness r with the same centre as the city. f) Urban expansion factors and their inter quartile range from the Theil-Sen estimation.

Irapuato, 11.2.04

a)

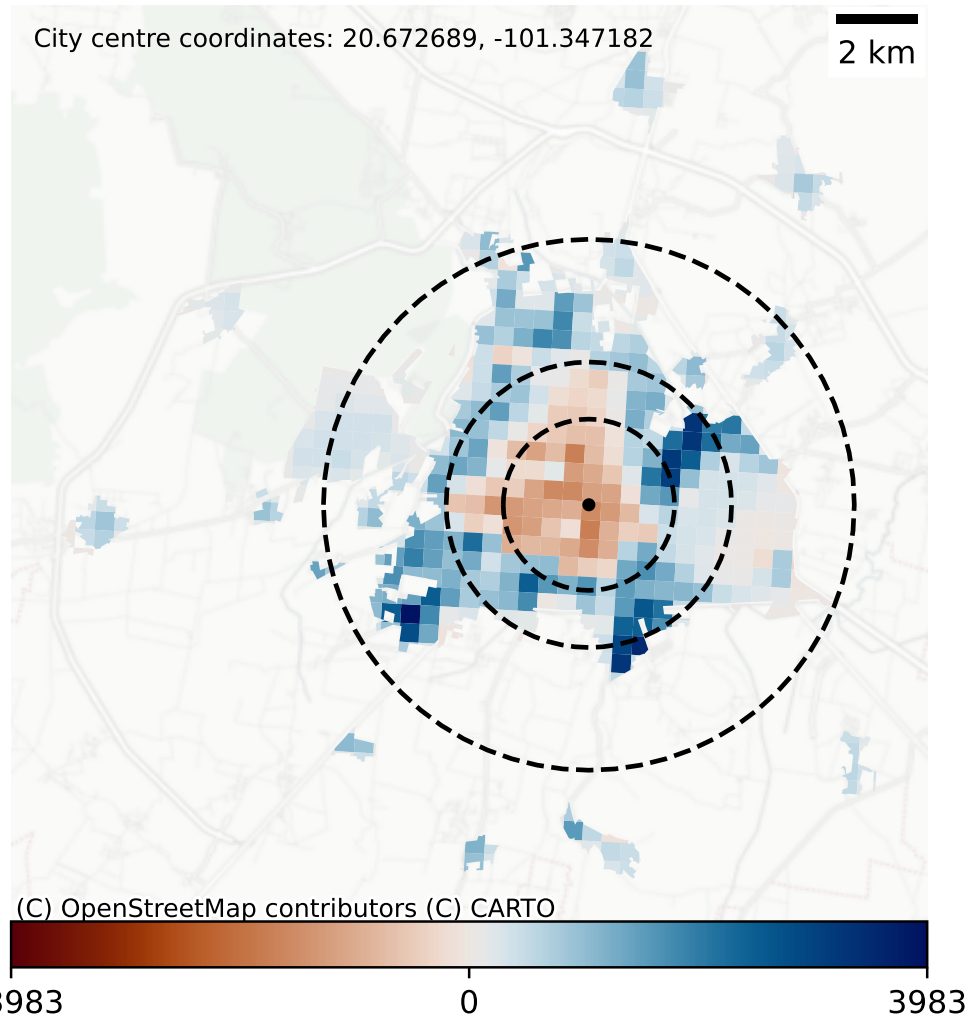

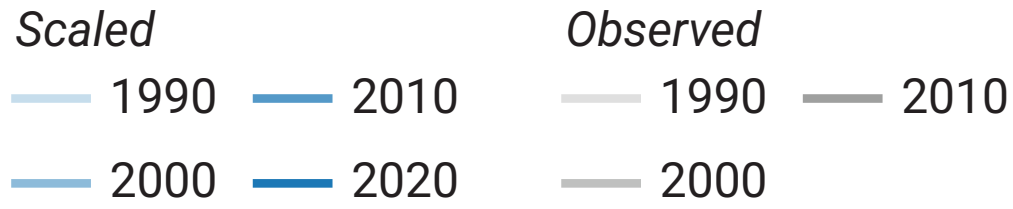

c)

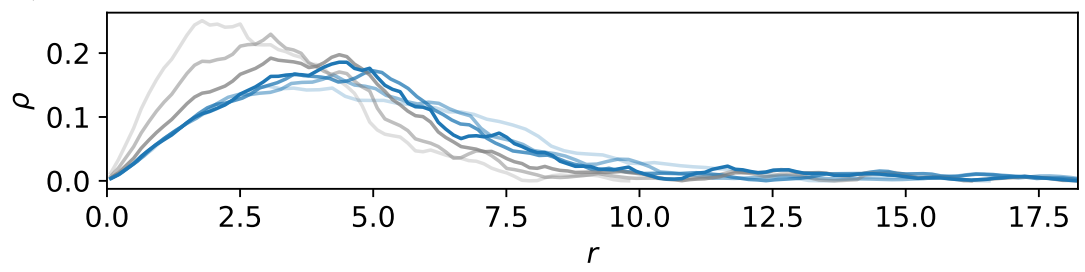

d)

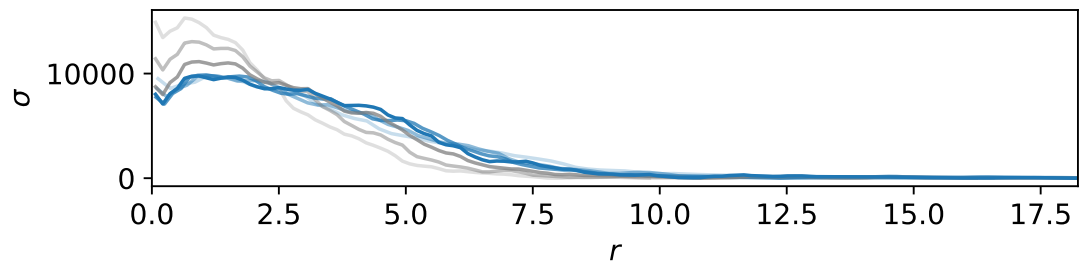

e)

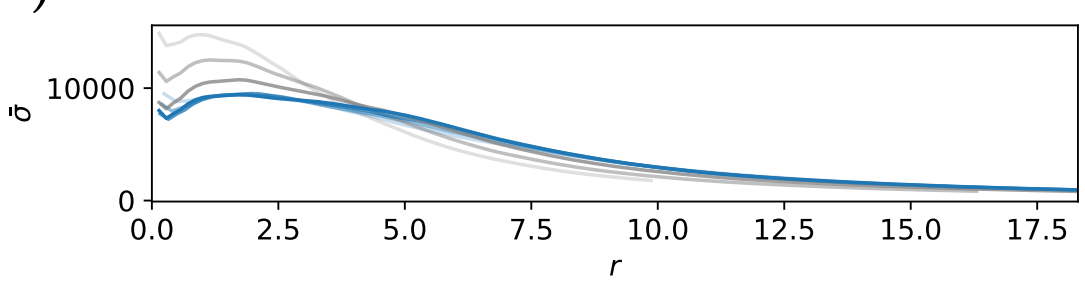

b)

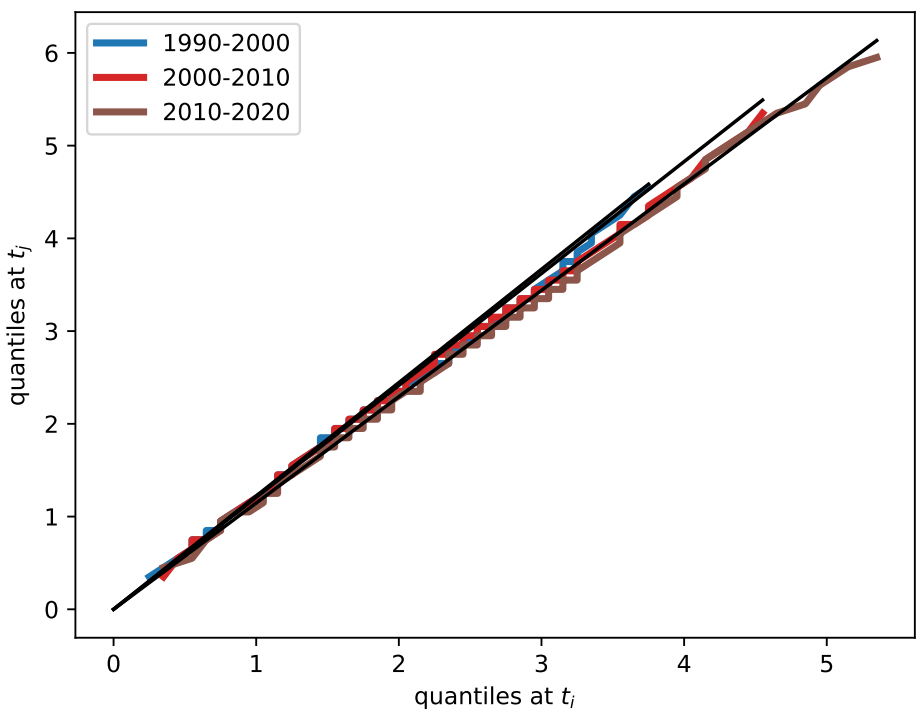

f)

Period (t_i-t_j)	$\frac{P(t_j)}{P(t_i)}$	Φ_{ij}	IQR
1990-2000	1.27	1.22	(1.19, 1.25)
2000-2010	1.24	1.21	(1.18, 1.23)
2010-2020	1.16	1.15	(1.13, 1.17)
1990-2010	1.57	1.47	(1.43, 1.52)
2000-2020	1.44	1.38	(1.35, 1.41)
1990-2020	1.83	1.67	(1.64, 1.73)

Supplementary Figure 28: Supplementary data for the metropolitan zone of Irapuato with code 11.2.04. Remoteness values are those of 2020. a) Population difference by grid cell (2020–1990); city centres are denoted as black dots. b) Quantile-quantile plots for the radial population distributions $\rho(s, t_i)$ and $\rho(s, t_j)$ (coloured curves); urban expansion factors Φ_{ij} from t_i to t_j are the estimated slopes (black lines). c) Radial population distribution $\rho(r)$ at remoteness distance r from the city centre. d) Radial population density $\sigma(r)$ at remoteness distance r from the city centre. e) Average population density $\bar{\sigma}(r)$ within disks of remoteness r with the same centre as the city. f) Urban expansion factors and their inter quartile range from the Theil-Sen estimation.

Chilpancingo, 12.1.01

a)

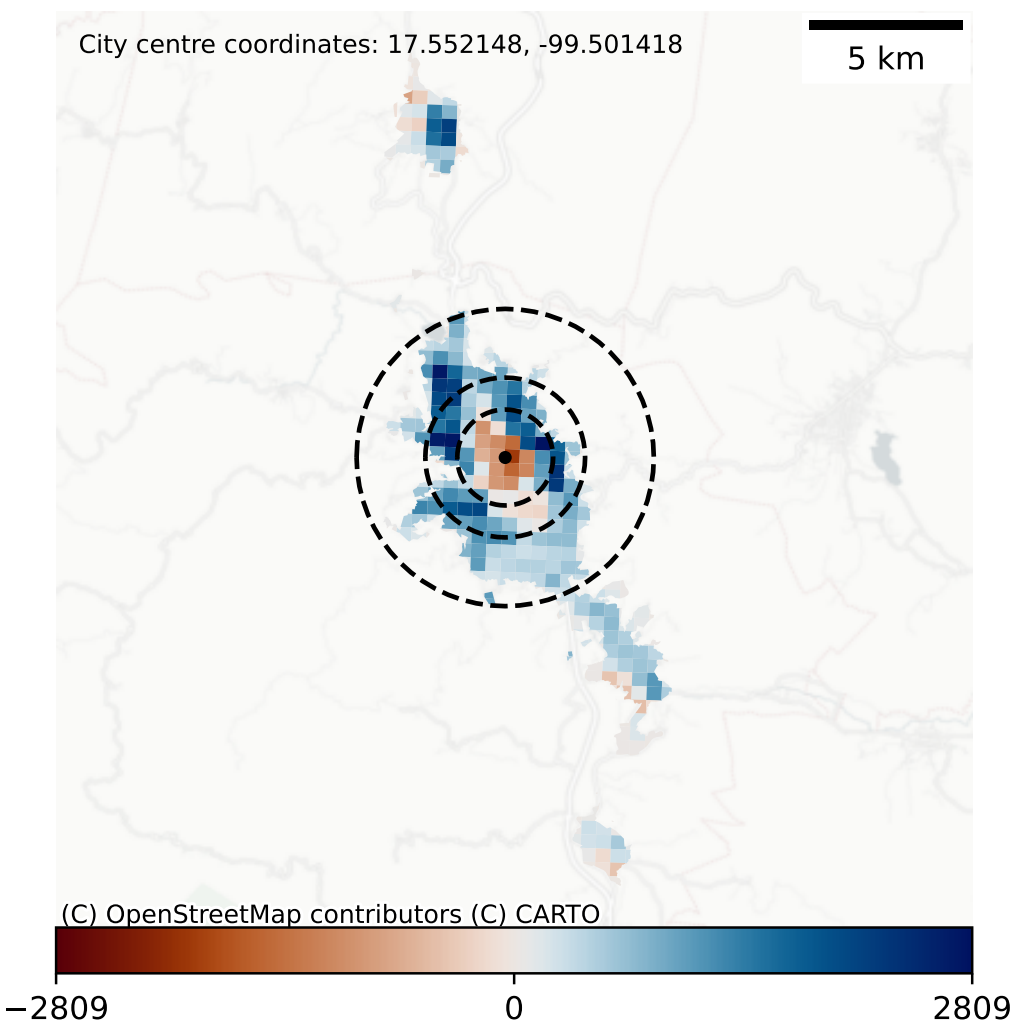

b)

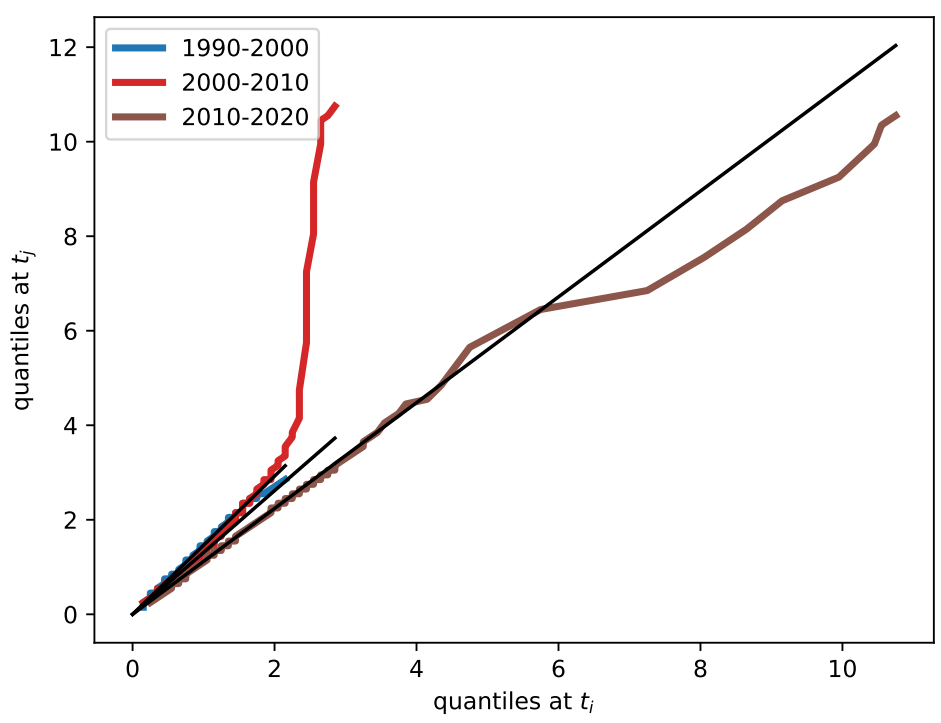

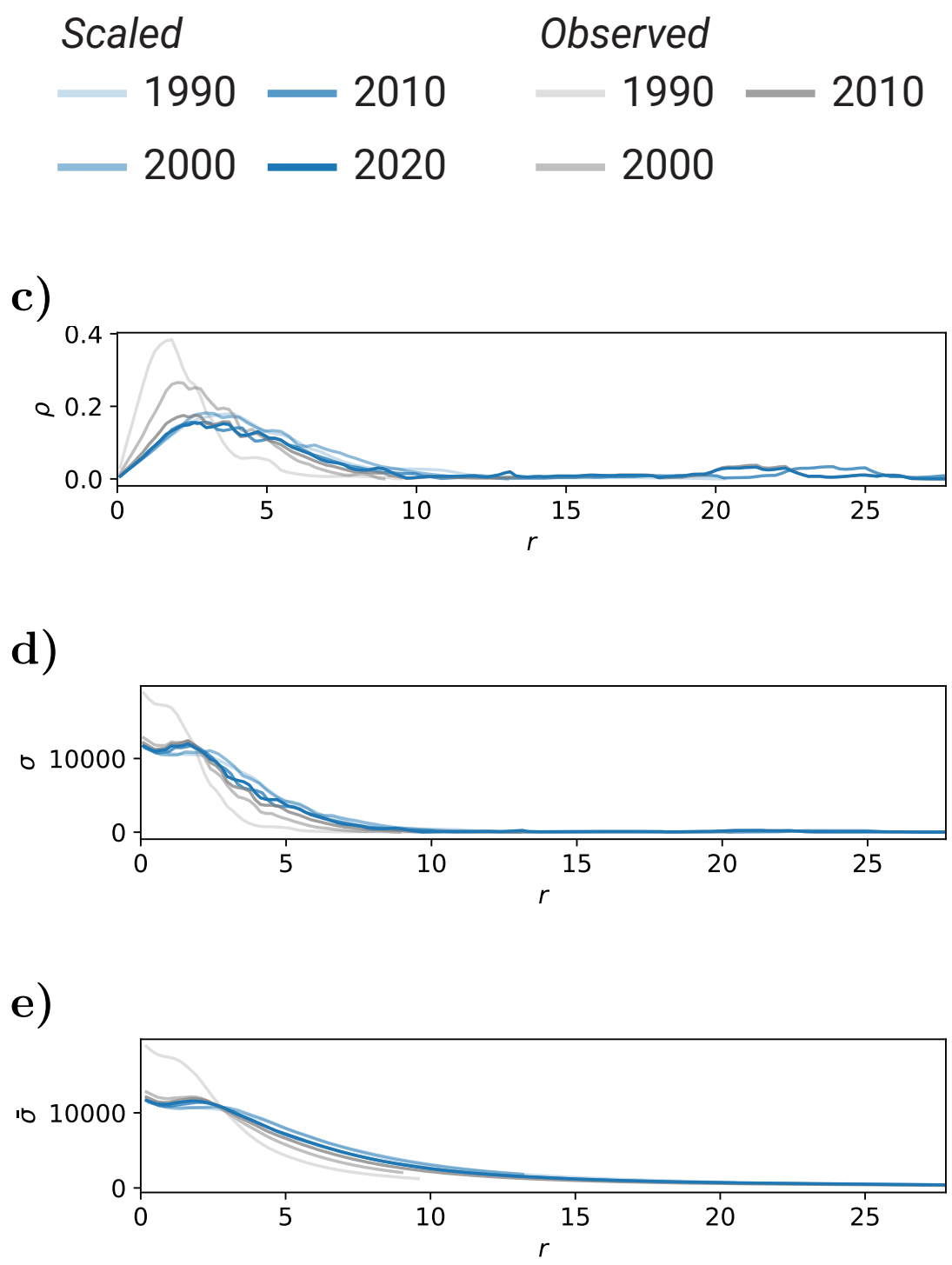

f)

Period (t_i-t_j)	$\frac{P(t_j)}{P(t_i)}$	Φ_{ij}	IQR
1990-2000	1.47	1.46	(1.40, 1.55)
2000-2010	1.59	1.31	(1.26, 1.35)
2010-2020	1.20	1.12	(1.09, 1.14)
1990-2010	2.34	1.91	(1.80, 1.94)
2000-2020	1.91	1.45	(1.40, 1.48)
1990-2020	2.80	2.11	(2.06, 2.20)

Supplementary Figure 29: Supplementary data for the metropolitan zone of Chilpancingo with code 12.1.01. Remoteness values are those of 2020. a) Population difference by grid cell (2020–1990); city centres are denoted as black dots. b) Quantile-quantile plots for the radial population distributions $\rho(s, t_i)$ and $\rho(s, t_j)$ (coloured curves); urban expansion factors Φ_{ij} from t_i to t_j are the estimated slopes (black lines). c) Radial population distribution $\rho(r)$ at remoteness distance r from the city centre. d) Radial population density $\sigma(r)$ at remoteness distance r from the city centre. e) Average population density $\bar{\sigma}(r)$ within disks of remoteness r with the same centre as the city. f) Urban expansion factors and their inter quartile range from the Theil-Sen estimation.

Acapulco, 12.2.02

a)

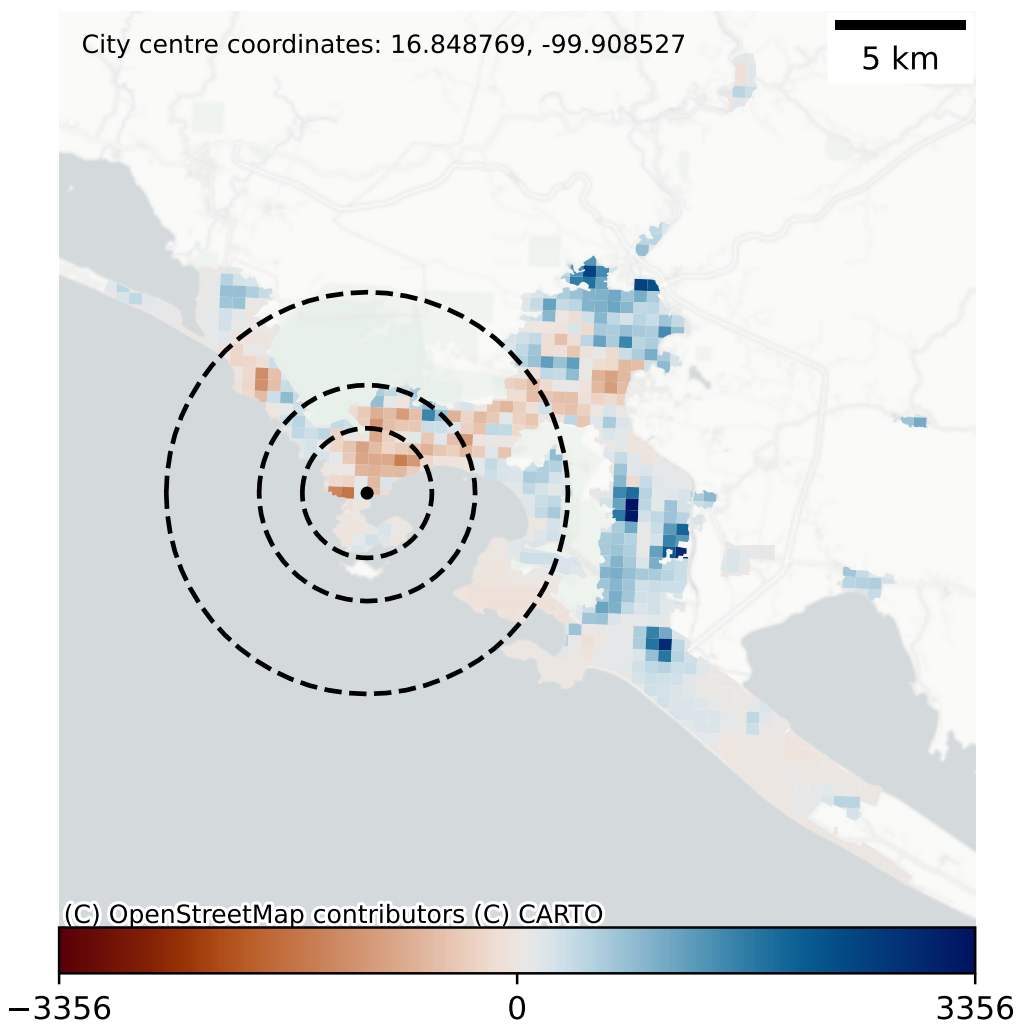

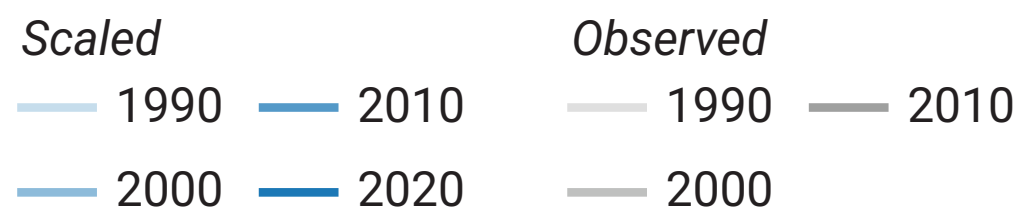

c)

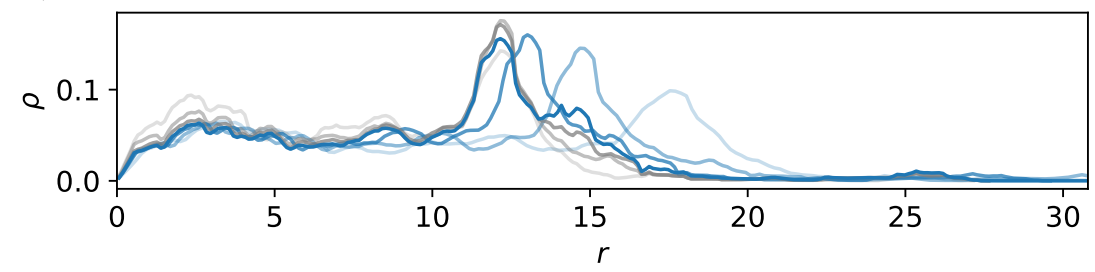

d)

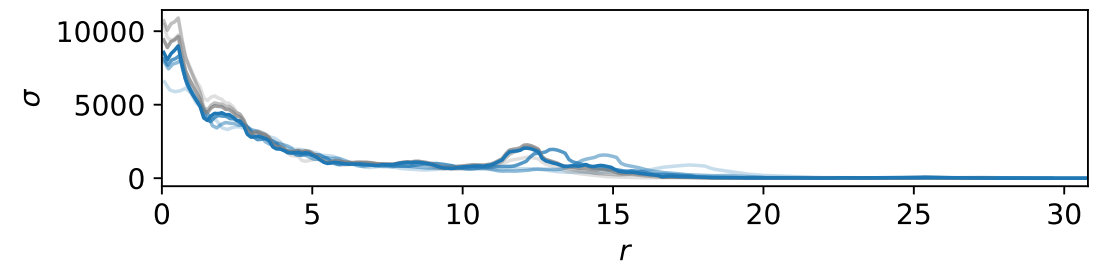

b)

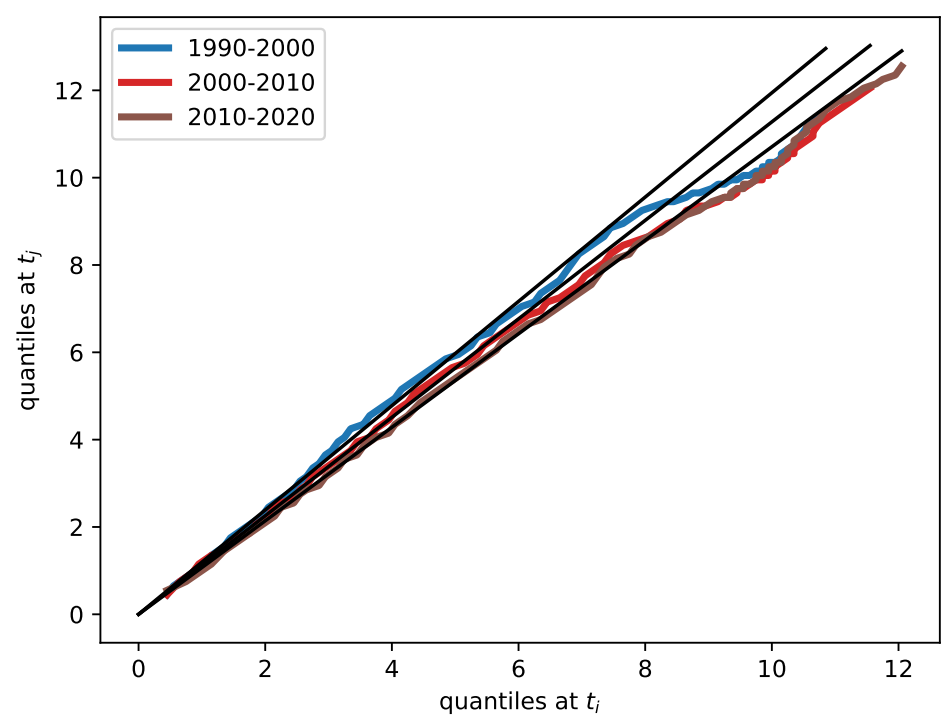

e)

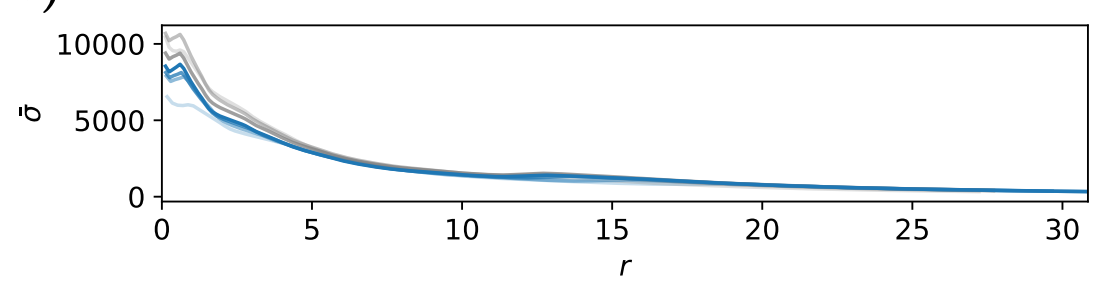

f)

Period (t_i-t_j)	$\frac{P(t_j)}{P(t_i)}$	Φ_{ij}	IQR
1990-2000	1.21	1.19	(1.17, 1.23)
2000-2010	1.09	1.13	(1.11, 1.14)
2010-2020	0.99	1.07	(1.06, 1.08)
1990-2010	1.31	1.35	(1.31, 1.38)
2000-2020	1.08	1.21	(1.19, 1.22)
1990-2020	1.30	1.43	(1.38, 1.49)

Supplementary Figure 30: Supplementary data for the metropolitan zone of Acapulco with code 12.2.02. Remoteness values are those of 2020. a) Population difference by grid cell (2020–1990); city centres are denoted as black dots. b) Quantile-quantile plots for the radial population distributions $\rho(s, t_i)$ and $\rho(s, t_j)$ (coloured curves); urban expansion factors Φ_{ij} from t_i to t_j are the estimated slopes (black lines). c) Radial population distribution $\rho(r)$ at remoteness distance r from the city centre. d) Radial population density $\sigma(r)$ at remoteness distance r from the city centre. e) Average population density $\bar{\sigma}(r)$ within disks of remoteness r with the same centre as the city. f) Urban expansion factors and their inter quartile range from the Theil-Sen estimation.

Pachuca, 13.1.01

a)

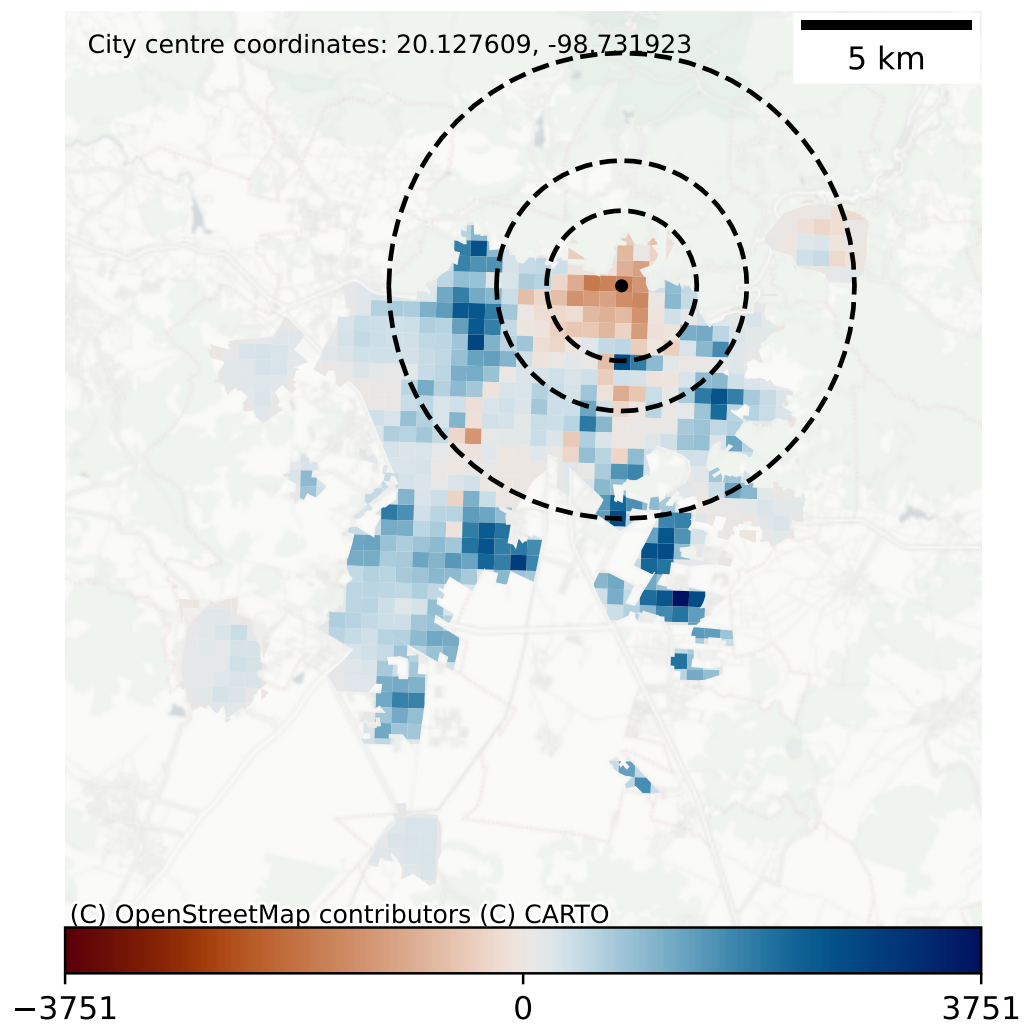

b)

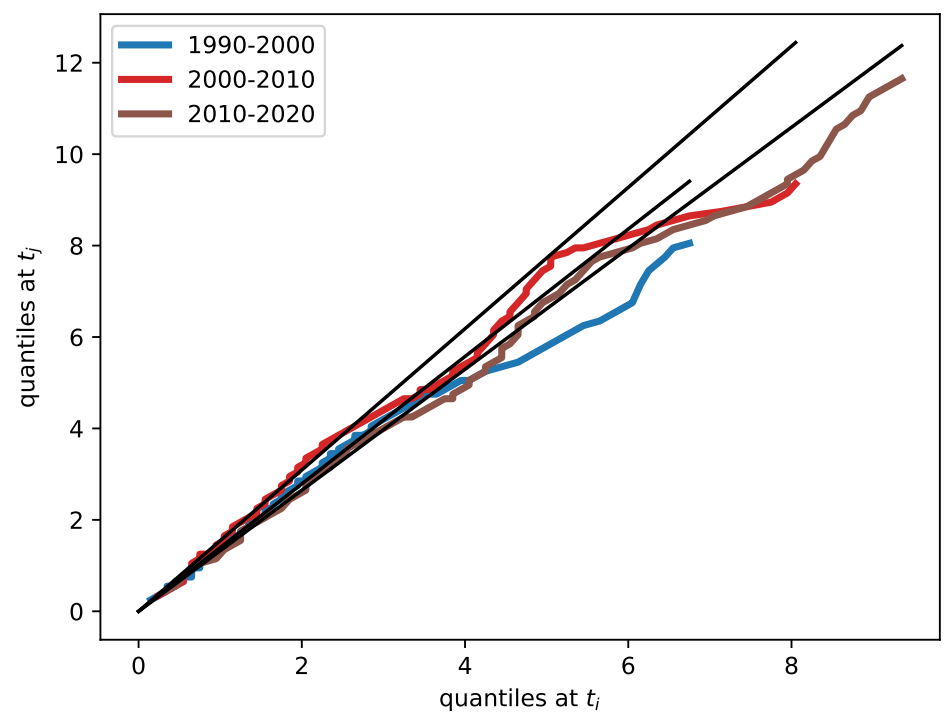

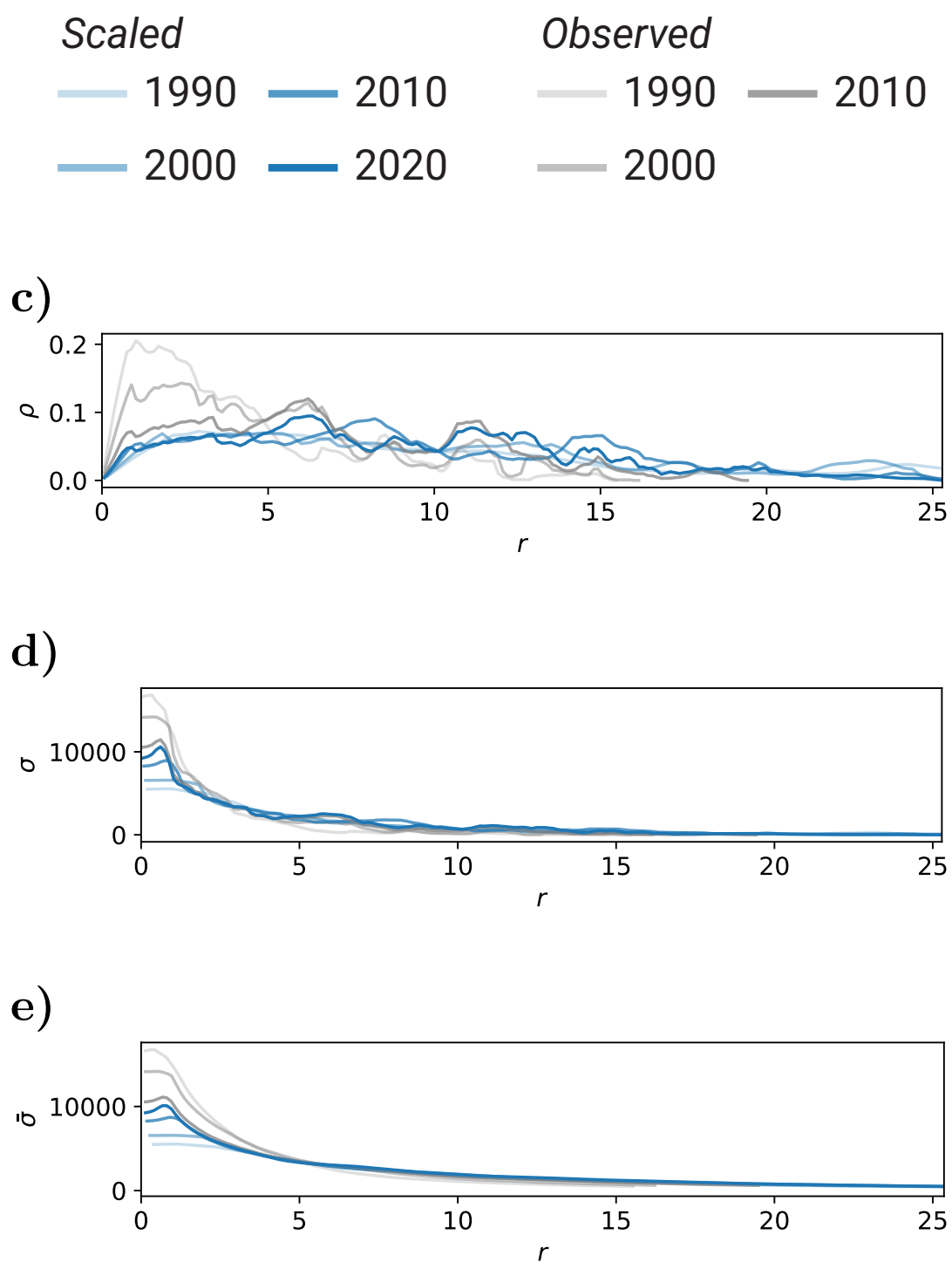

f)

Period (t_i-t_j)	$\frac{P(t_j)}{P(t_i)}$	Φ_{ij}	IQR
1990-2000	1.38	1.39	(1.35, 1.42)
2000-2010	1.41	1.55	(1.49, 1.58)
2010-2020	1.37	1.32	(1.27, 1.36)
1990-2010	1.95	2.15	(2.05, 2.21)
2000-2020	1.94	2.04	(1.95, 2.08)
1990-2020	2.68	2.83	(2.67, 2.91)

Supplementary Figure 31: Supplementary data for the metropolitan zone of Pachuca with code 13.1.01. Remoteness values are those of 2020. a) Population difference by grid cell (2020–1990); city centres are denoted as black dots. b) Quantile-quantile plots for the radial population distributions $\rho(s, t_i)$ and $\rho(s, t_j)$ (coloured curves); urban expansion factors Φ_{ij} from t_i to t_j are the estimated slopes (black lines). c) Radial population distribution $\rho(r)$ at remoteness distance r from the city centre. d) Radial population density $\sigma(r)$ at remoteness distance r from the city centre. e) Average population density $\bar{\sigma}(r)$ within disks of remoteness r with the same centre as the city. f) Urban expansion factors and their inter quartile range from the Theil-Sen estimation.

Tulancingo, 13.1.02

a)

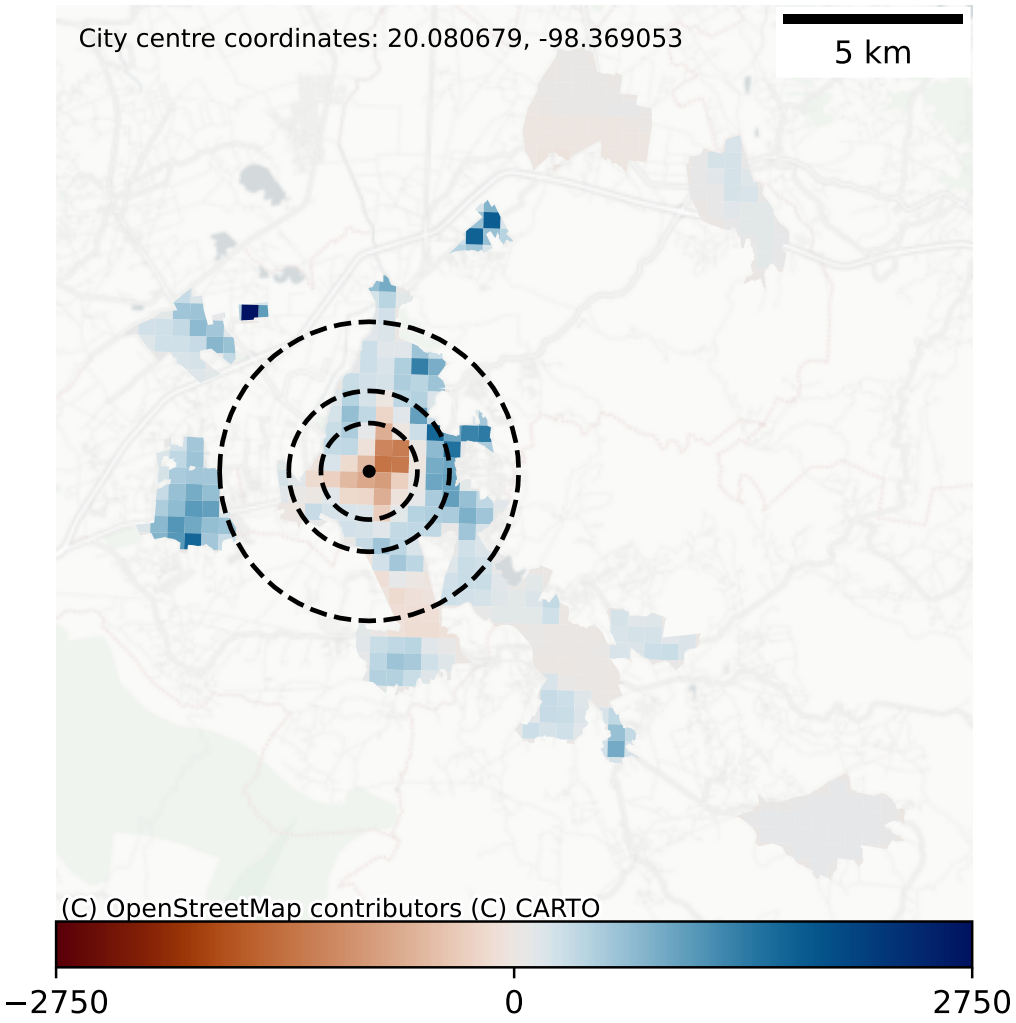

b)

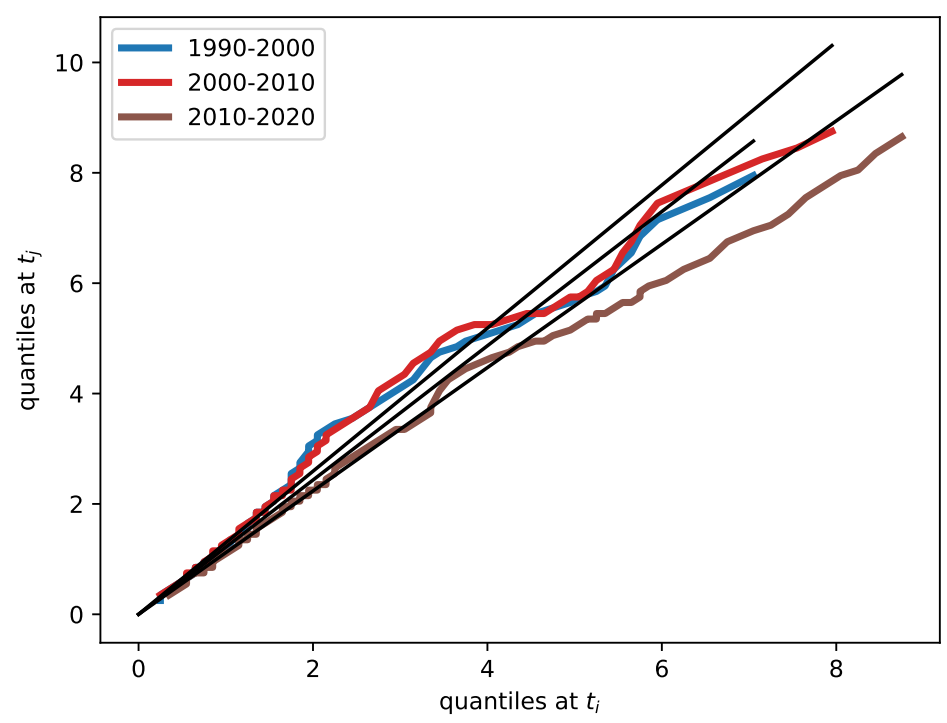

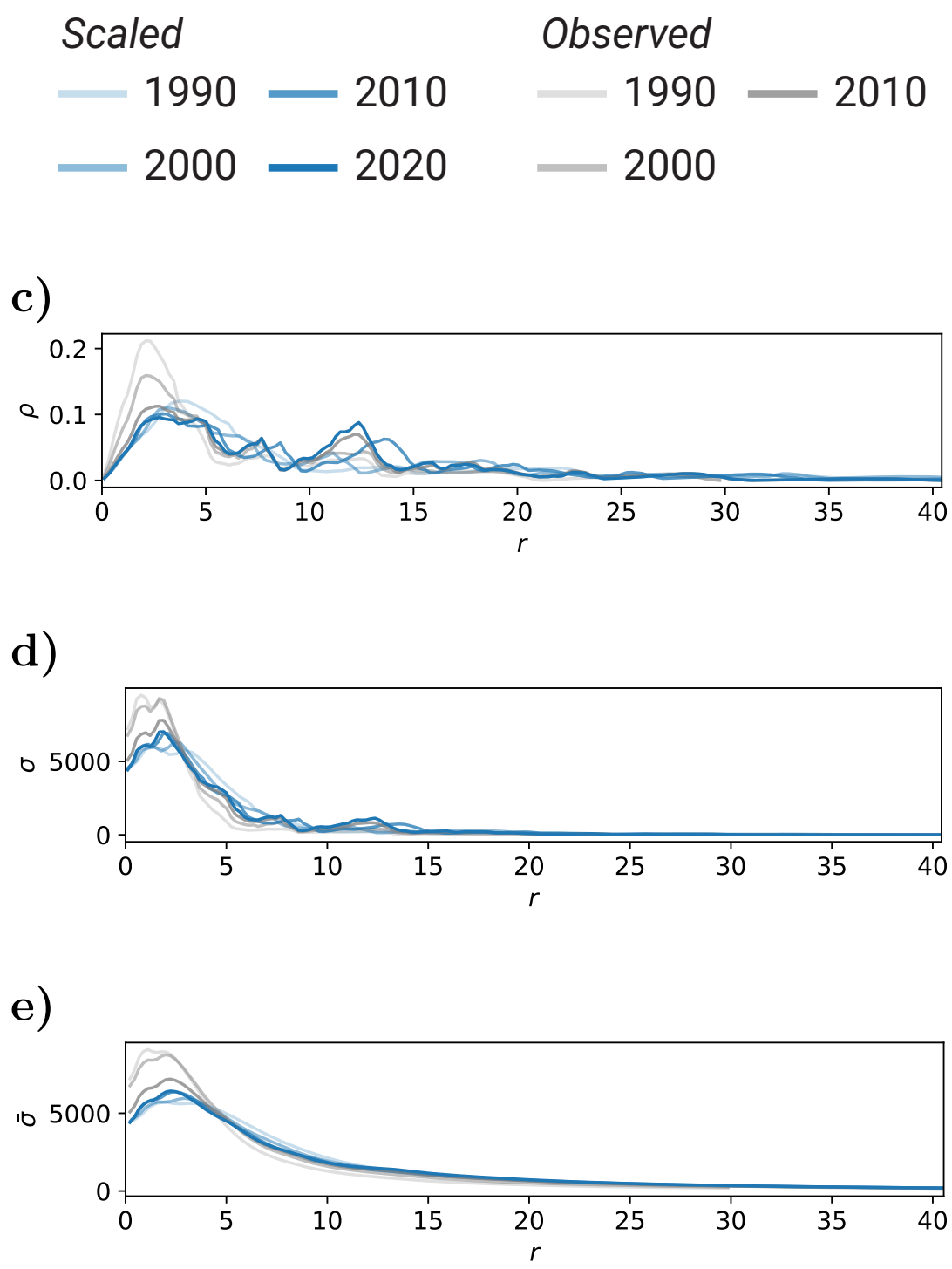

f)

Period (t_i-t_j)	$\frac{P(t_j)}{P(t_i)}$	Φ_{ij}	IQR
1990-2000	1.37	1.22	(1.18, 1.26)
2000-2010	1.29	1.30	(1.26, 1.32)
2010-2020	1.11	1.12	(1.09, 1.15)
1990-2010	1.76	1.57	(1.53, 1.63)
2000-2020	1.43	1.44	(1.37, 1.48)
1990-2020	1.95	1.76	(1.67, 1.84)

Supplementary Figure 32: Supplementary data for the metropolitan zone of Tulancingo with code 13.1.02. Remoteness values are those of 2020. a) Population difference by grid cell (2020–1990); city centres are denoted as black dots. b) Quantile-quantile plots for the radial population distributions $\rho(s, t_i)$ and $\rho(s, t_j)$ (coloured curves); urban expansion factors Φ_{ij} from t_i to t_j are the estimated slopes (black lines). c) Radial population distribution $\rho(r)$ at remoteness distance r from the city centre. d) Radial population density $\sigma(r)$ at remoteness distance r from the city centre. e) Average population density $\bar{\sigma}(r)$ within disks of remoteness r with the same centre as the city. f) Urban expansion factors and their inter quartile range from the Theil-Sen estimation.

Guadalajara, 14.1.01

a)

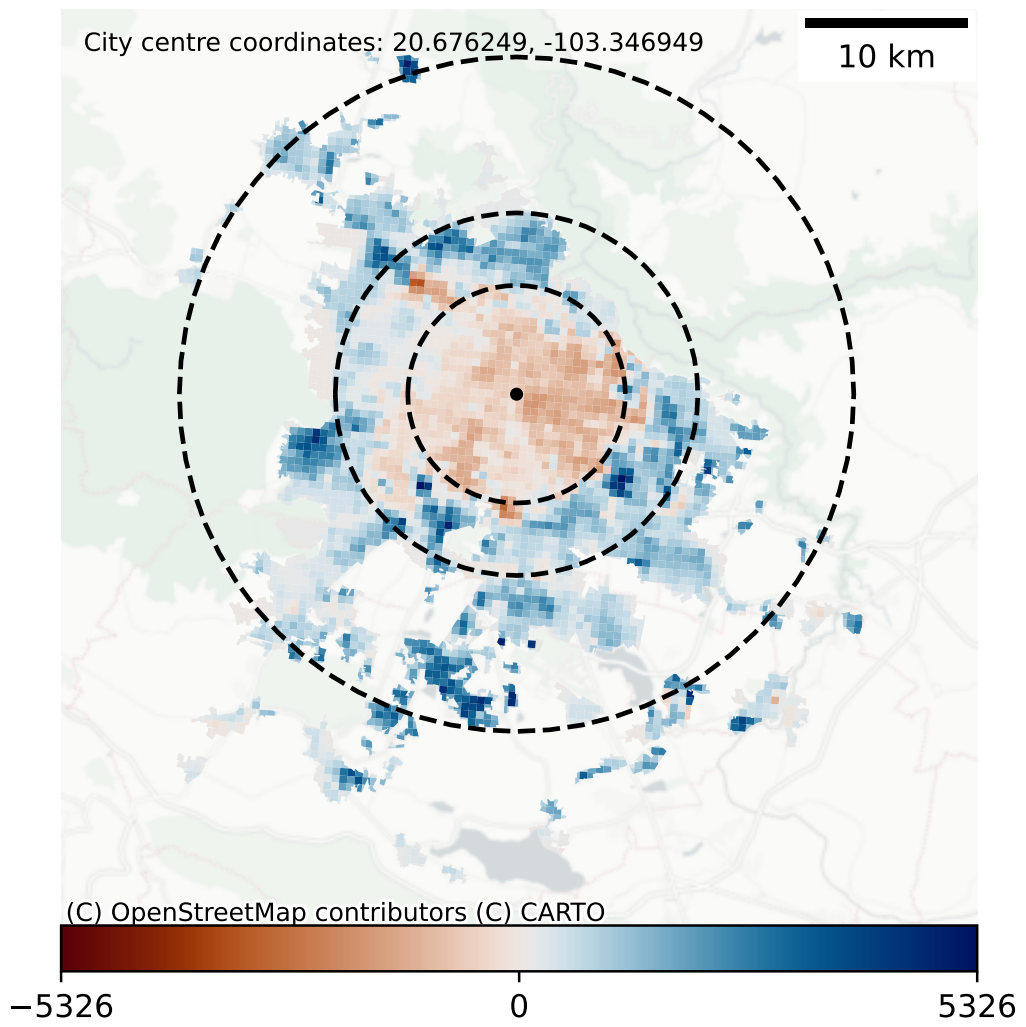

b)

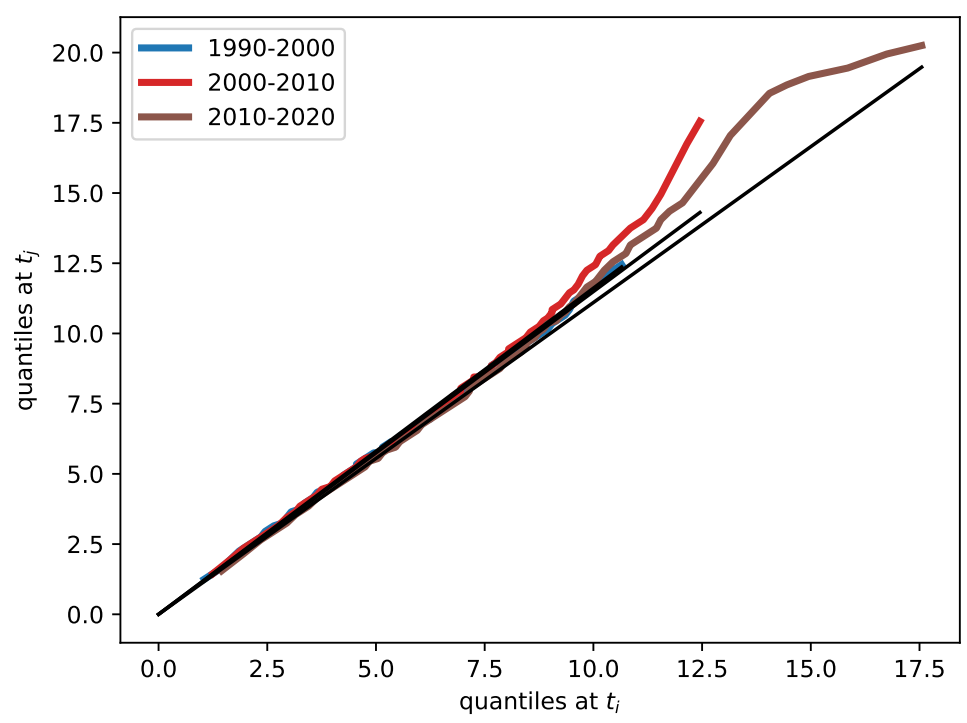

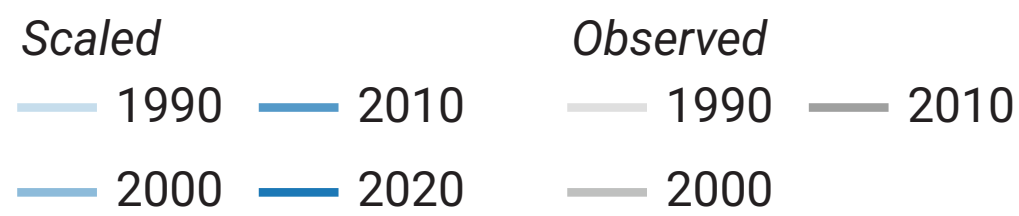

c)

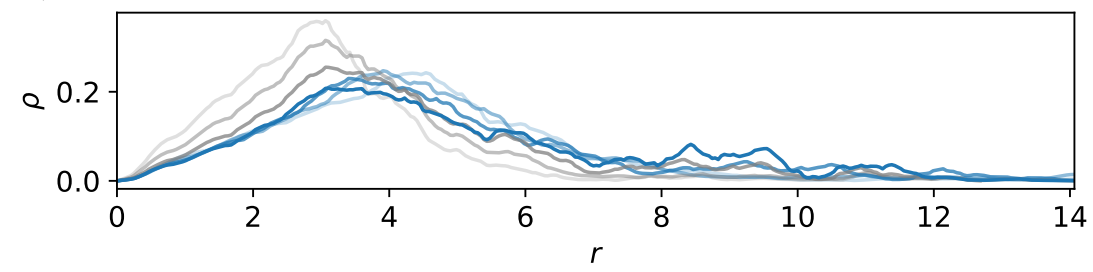

d)

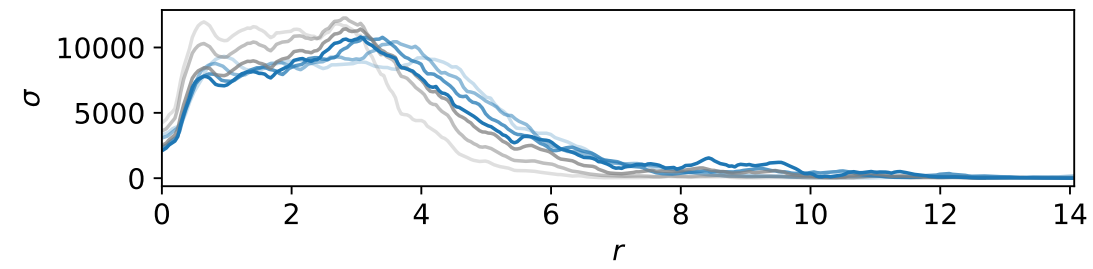

e)

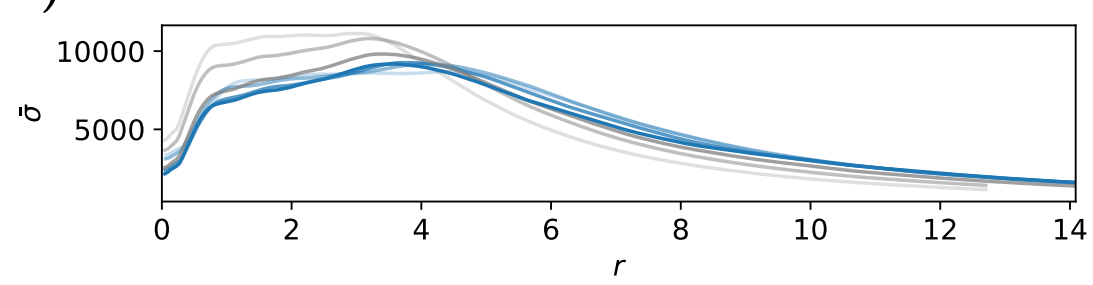

f)

Period (t_i-t_j)	$\frac{P(t_j)}{P(t_i)}$	Φ_{ij}	IQR
1990-2000	1.23	1.16	(1.15, 1.18)
2000-2010	1.19	1.15	(1.13, 1.17)
2010-2020	1.16	1.11	(1.10, 1.12)
1990-2010	1.47	1.33	(1.31, 1.37)
2000-2020	1.39	1.27	(1.26, 1.30)
1990-2020	1.71	1.48	(1.45, 1.52)

Supplementary Figure 33: Supplementary data for the metropolitan zone of Guadalajara with code 14.1.01. Remoteness values are those of 2020. a) Population difference by grid cell (2020–1990); city centres are denoted as black dots. b) Quantile-quantile plots for the radial population distributions $\rho(s, t_i)$ and $\rho(s, t_j)$ (coloured curves); urban expansion factors Φ_{ij} from t_i to t_j are the estimated slopes (black lines). c) Radial population distribution $\rho(r)$ at remoteness distance r from the city centre. d) Radial population density $\sigma(r)$ at remoteness distance r from the city centre. e) Average population density $\bar{\sigma}(r)$ within disks of remoteness r with the same centre as the city. f) Urban expansion factors and their inter quartile range from the Theil-Sen estimation.

Puerto Vallarta, 14.1.02

a)

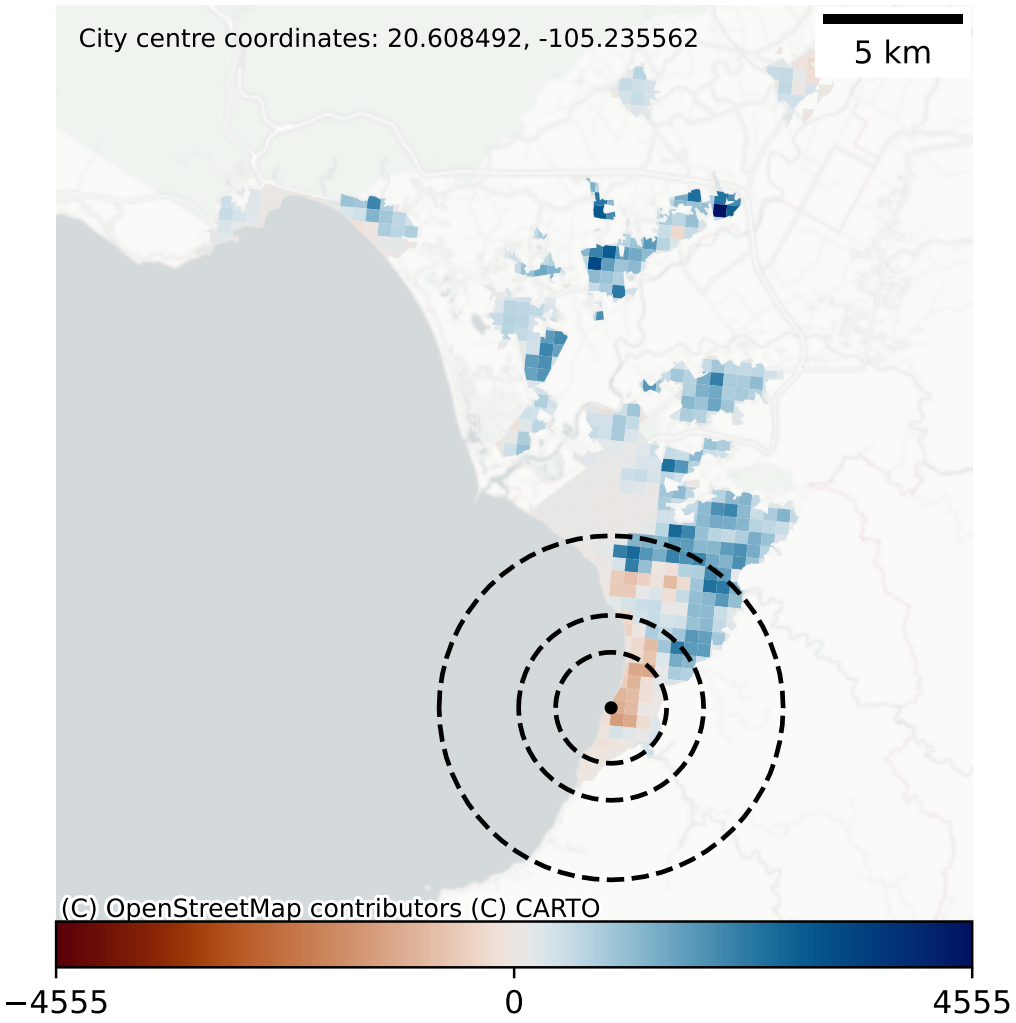

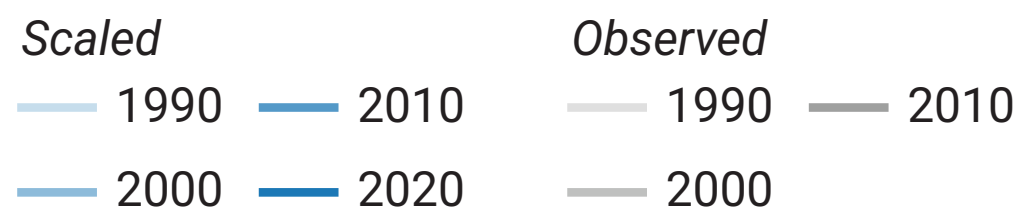

c)

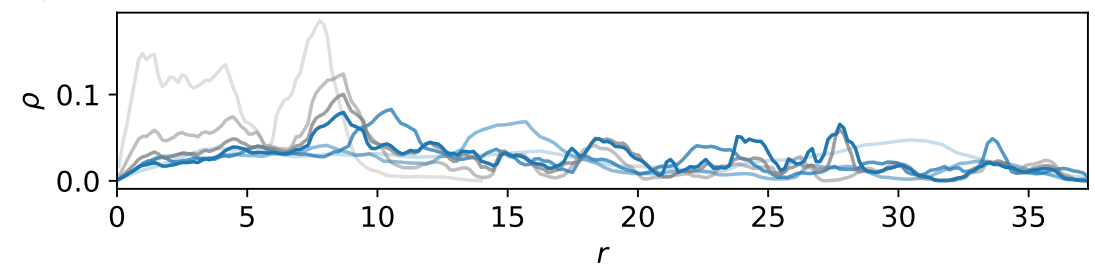

d)

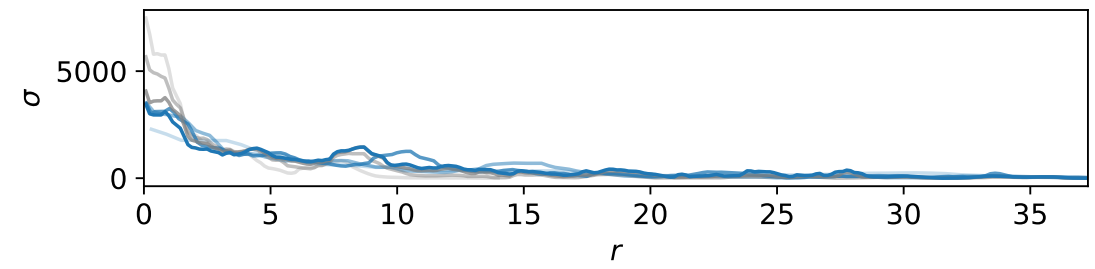

e)

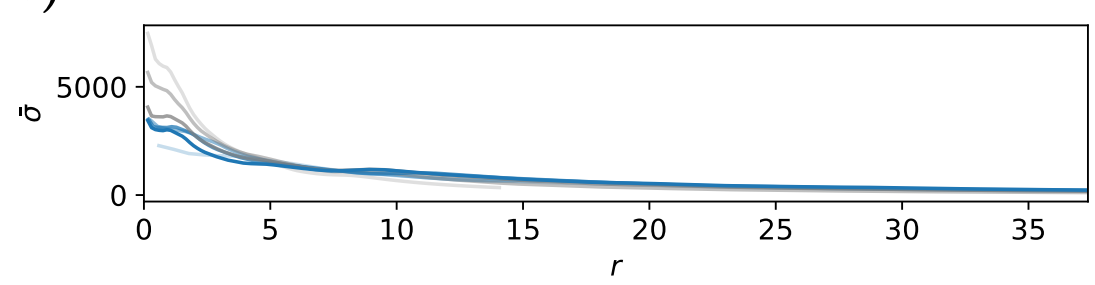

b)

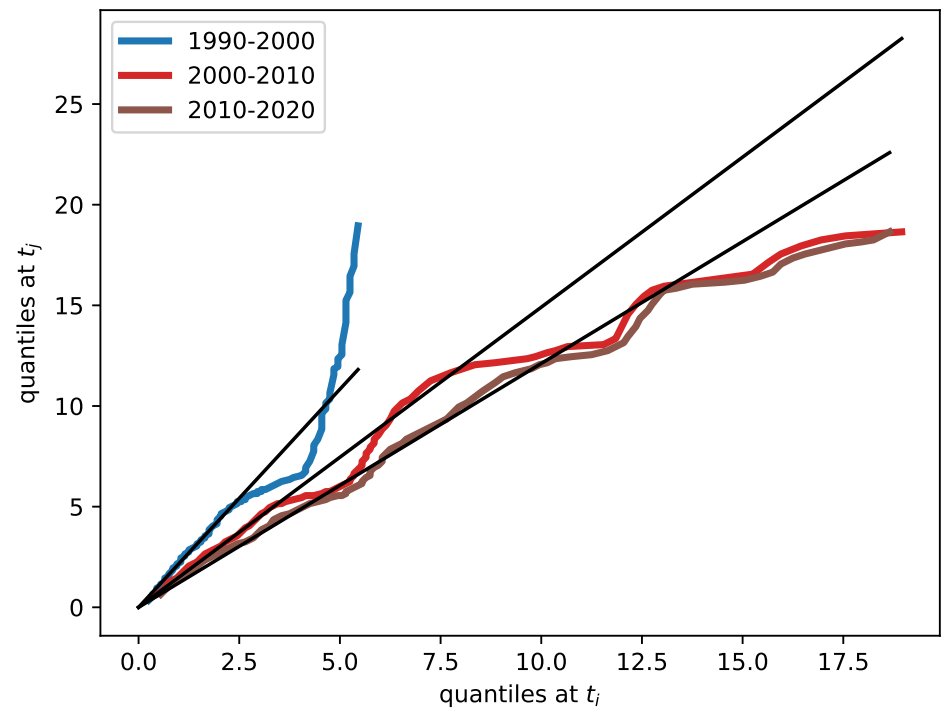

f)

Period (t_i-t_j)	$\frac{P(t_j)}{P(t_i)}$	Φ_{ij}	IQR
1990-2000	2.36	2.17	(2.09, 2.22)
2000-2010	1.57	1.49	(1.40, 1.53)
2010-2020	1.27	1.21	(1.14, 1.28)
1990-2010	3.69	3.19	(2.85, 3.29)
2000-2020	1.99	1.82	(1.61, 1.93)
1990-2020	4.68	3.87	(3.29, 4.21)

Supplementary Figure 34: Supplementary data for the metropolitan zone of Puerto Vallarta with code 14.1.02. Remoteness values are those of 2020. a) Population difference by grid cell (2020–1990); city centres are denoted as black dots. b) Quantile-quantile plots for the radial population distributions $\rho(s,t_i)$ and $\rho(s,t_j)$ (coloured curves); urban expansion factors Φ_{ij} from t_i to t_j are the estimated slopes (black lines). c) Radial population distribution $\rho(r)$ at remoteness distance r from the city centre. d) Radial population density $\sigma(r)$ at remoteness distance r from the city centre. e) Average population density $\bar{\sigma}(r)$ within disks of remoteness r with the same centre as the city. f) Urban expansion factors and their inter quartile range from the Theil-Sen estimation.

Toluca, 15.1.01

a)

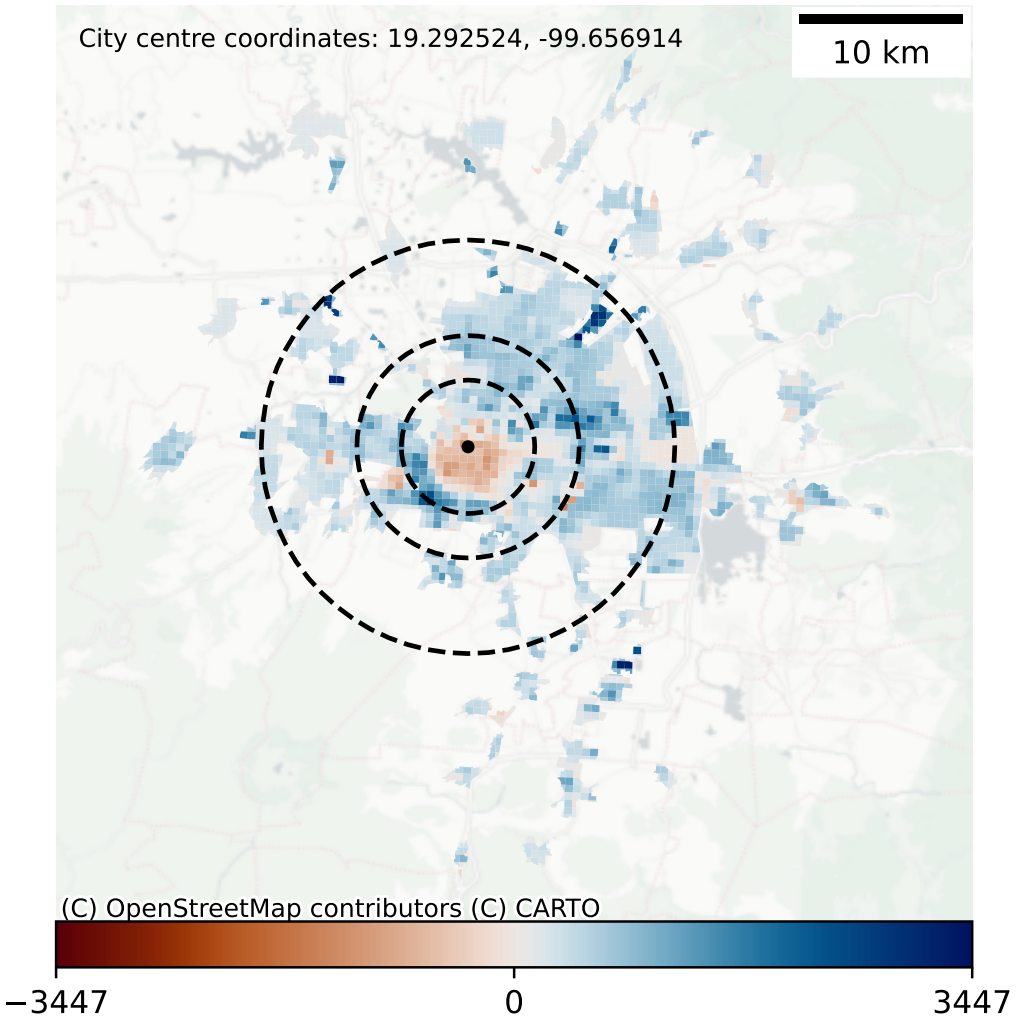

b)

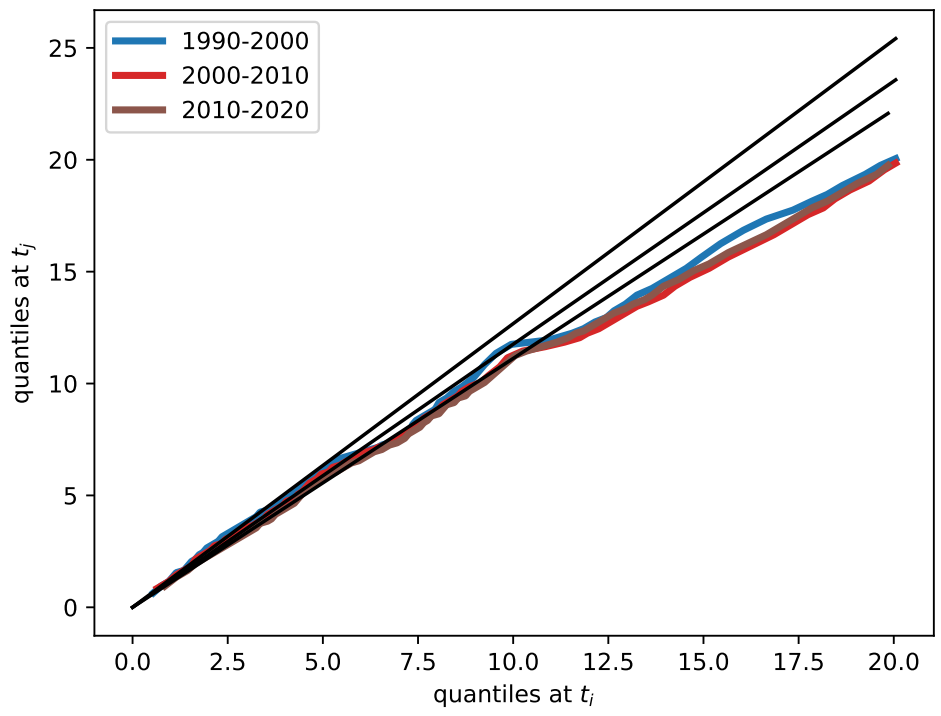

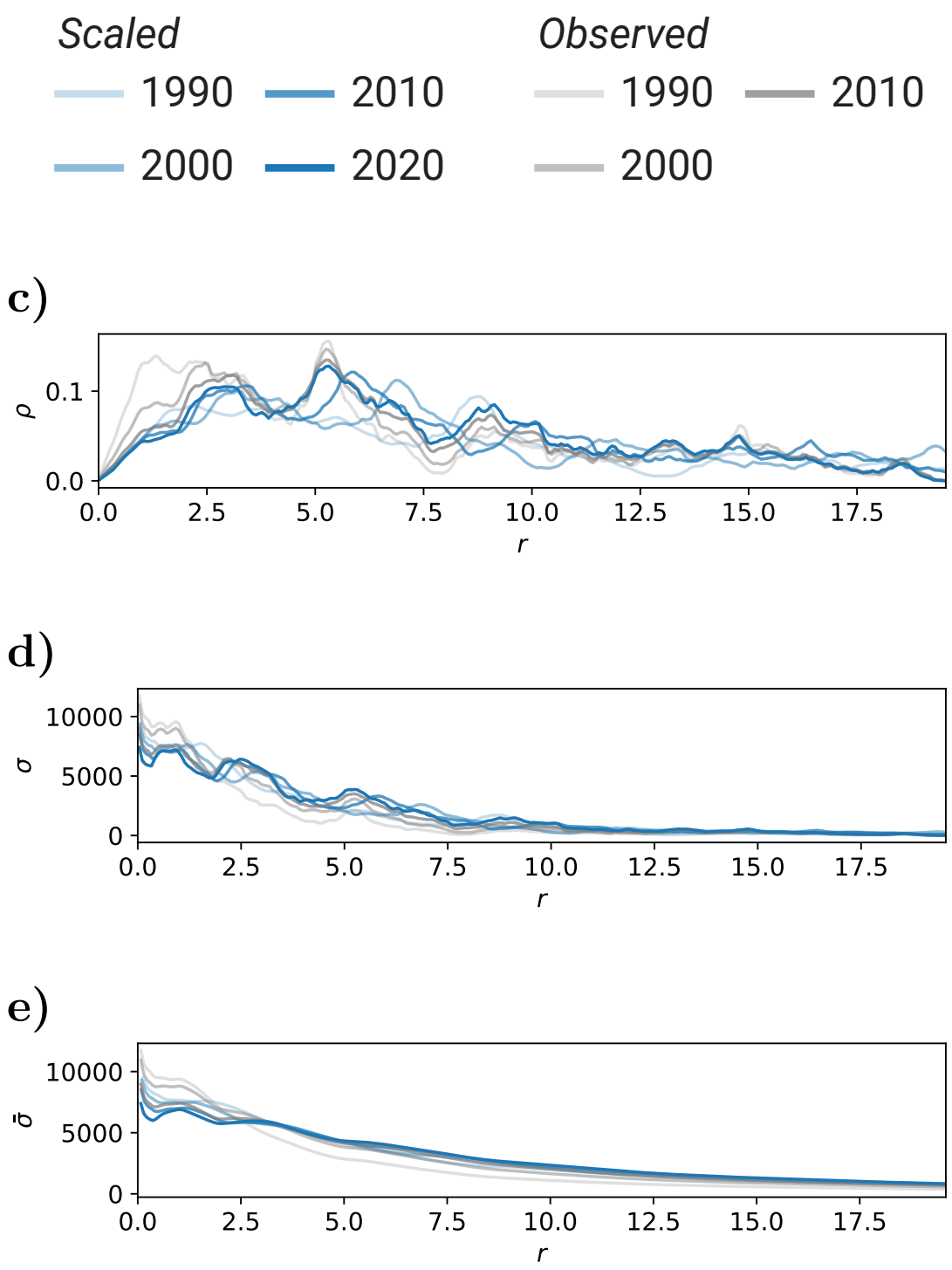

f)

Period (t_i-t_j)	$\frac{P(t_j)}{P(t_i)}$	Φ_{ij}	IQR
1990-2000	1.53	1.27	(1.22, 1.31)
2000-2010	1.24	1.18	(1.16, 1.21)
2010-2020	1.17	1.11	(1.10, 1.14)
1990-2010	1.90	1.50	(1.44, 1.58)
2000-2020	1.45	1.32	(1.28, 1.36)
1990-2020	2.22	1.67	(1.63, 1.76)

Supplementary Figure 35: Supplementary data for the metropolitan zone of Toluca with code 15.1.01. Remoteness values are those of 2020. a) Population difference by grid cell (2020–1990); city centres are denoted as black dots. b) Quantile-quantile plots for the radial population distributions $\rho(s, t_i)$ and $\rho(s, t_j)$ (coloured curves); urban expansion factors Φ_{ij} from t_i to t_j are the estimated slopes (black lines). c) Radial population distribution $\rho(r)$ at remoteness distance r from the city centre. d) Radial population density $\sigma(r)$ at remoteness distance r from the city centre. e) Average population density $\bar{\sigma}(r)$ within disks of remoteness r with the same centre as the city. f) Urban expansion factors and their inter quartile range from the Theil-Sen estimation.

La Piedad-Pénjamo, 16.1.01

a)

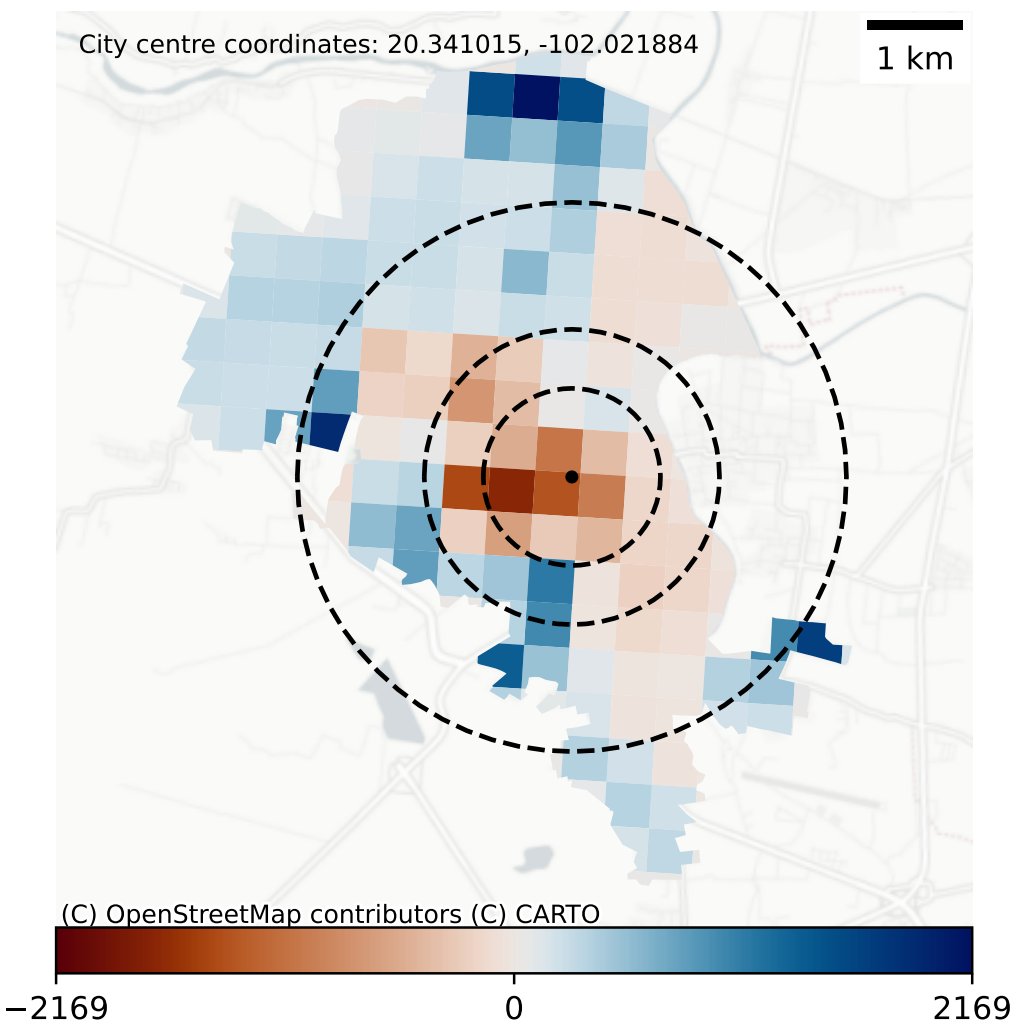

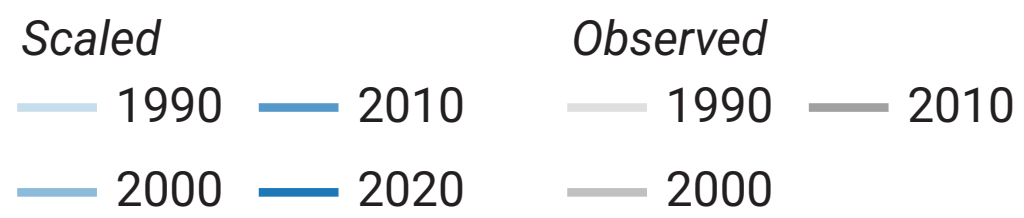

c)

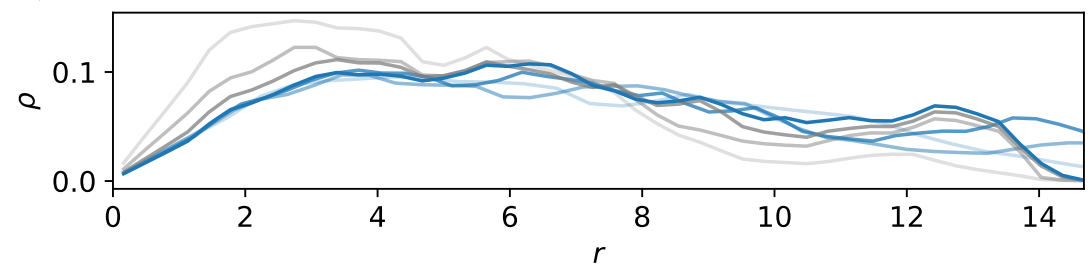

d)

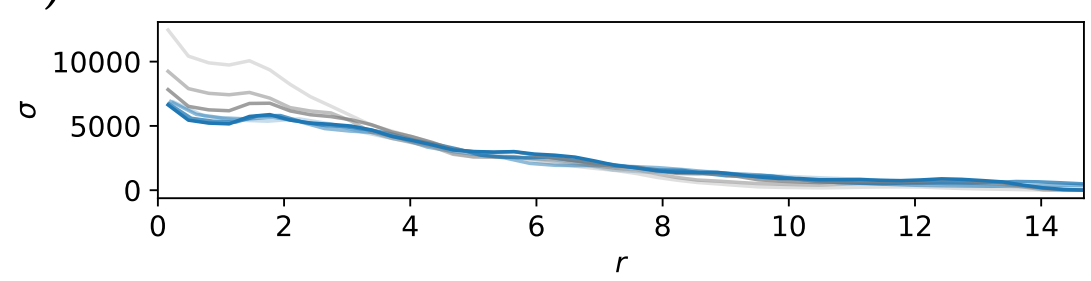

b)

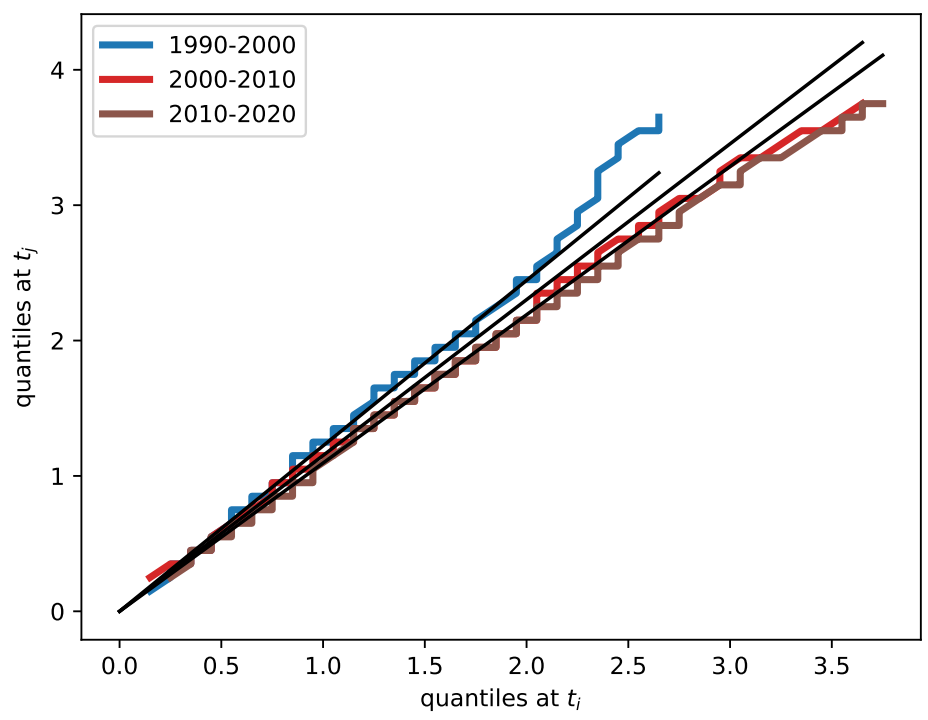

e)

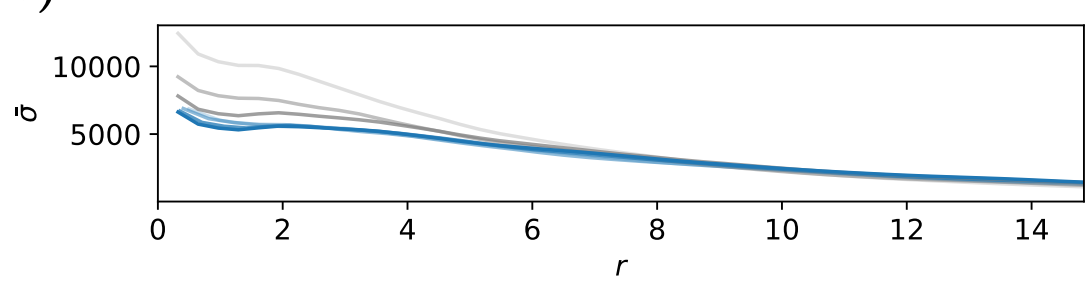

f)

Period (t_i-t_j)	$\frac{P(t_j)}{P(t_i)}$	Φ_{ij}	IQR
1990-2000	1.10	1.22	(1.18, 1.29)
2000-2010	1.15	1.15	(1.10, 1.19)
2010-2020	1.03	1.10	(1.07, 1.14)
1990-2010	1.27	1.40	(1.36, 1.46)
2000-2020	1.19	1.26	(1.22, 1.30)
1990-2020	1.31	1.54	(1.47, 1.59)

Supplementary Figure 36: Supplementary data for the metropolitan zone of La Piedad-Pénjamo with code 16.1.01. Remoteness values are those of 2020. a) Population difference by grid cell (2020–1990); city centres are denoted as black dots. b) Quantile-quantile plots for the radial population distributions $\rho(s,t_i)$ and $\rho(s,t_j)$ (coloured curves); urban expansion factors Φ_{ij} from t_i to t_j are the estimated slopes (black lines). c) Radial population distribution $\rho(r)$ at remoteness distance r from the city centre. d) Radial population density $\sigma(r)$ at remoteness distance r from the city centre. e) Average population density $\bar{\sigma}(r)$ within disks of remoteness r with the same centre as the city. f) Urban expansion factors and their inter quartile range from the Theil-Sen estimation.

Morelia, 16.1.02

a)

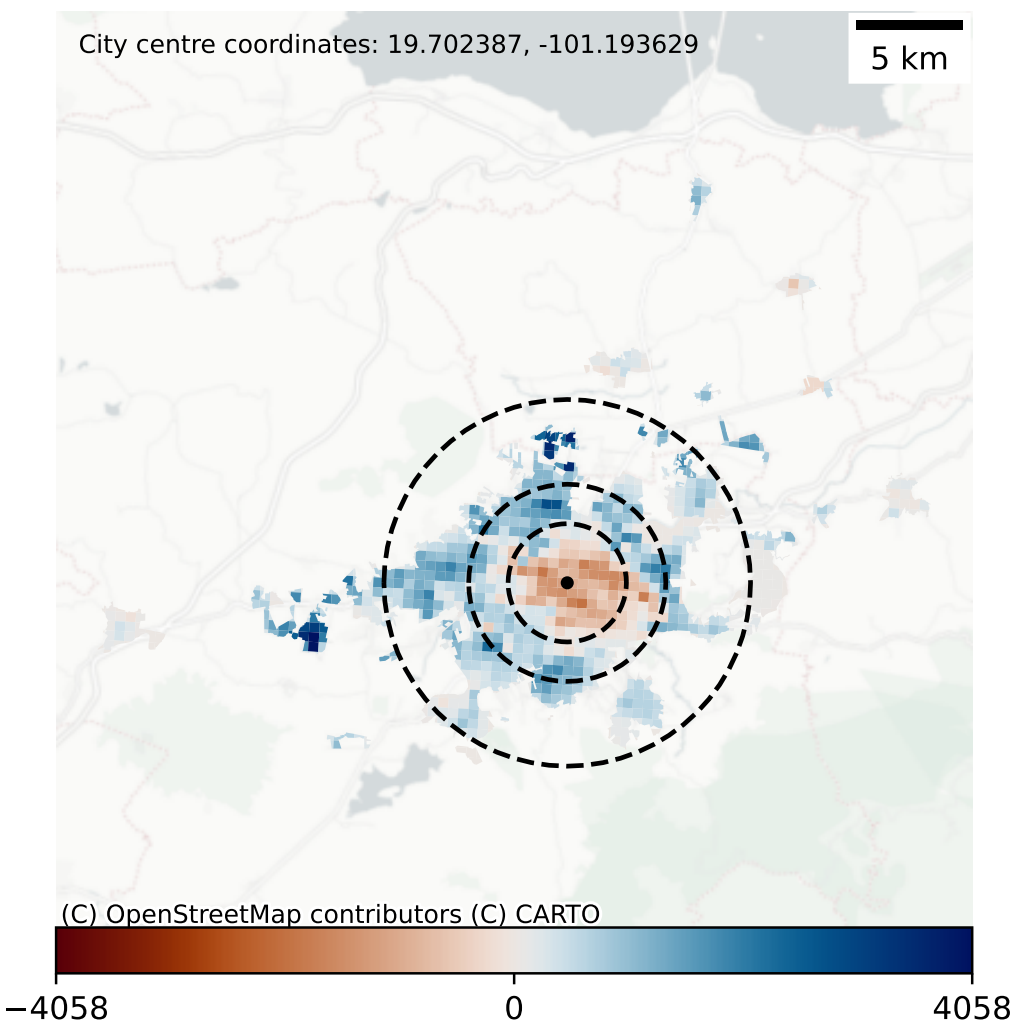

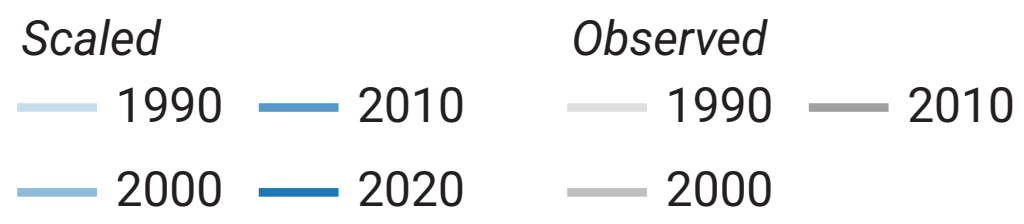

c)

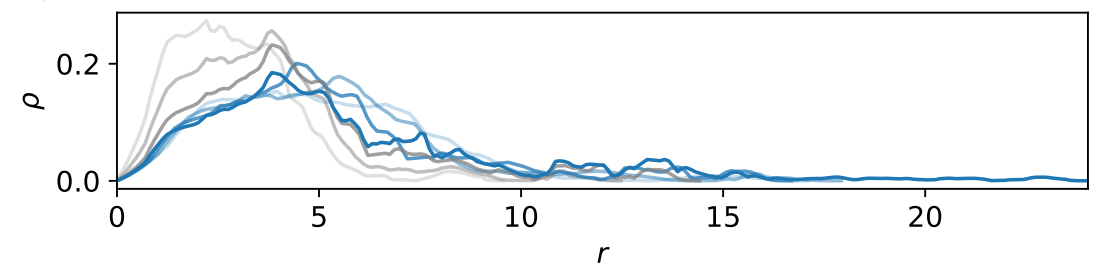

d)

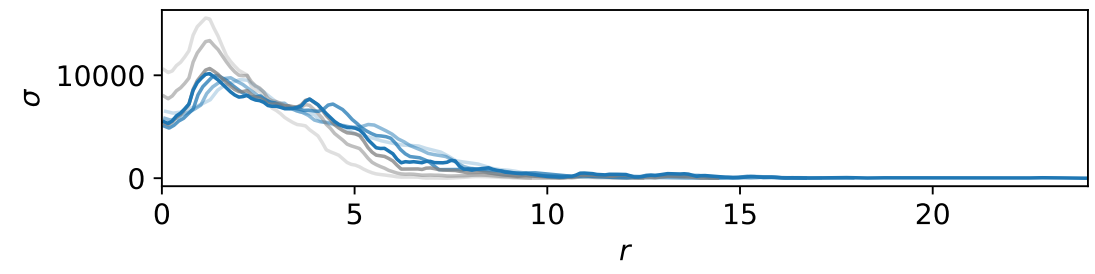

e)

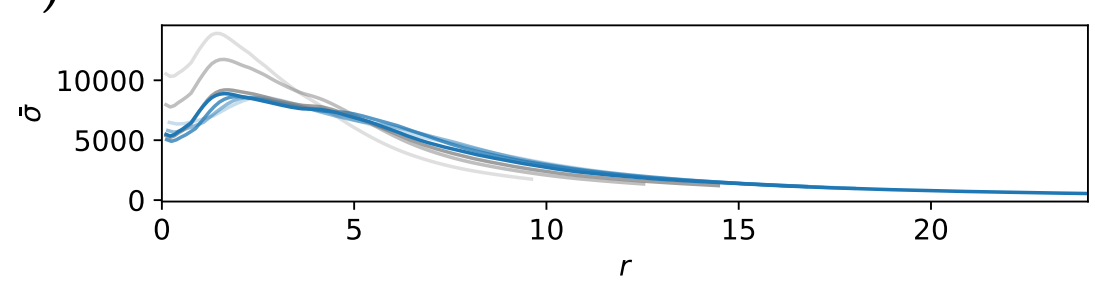

b)

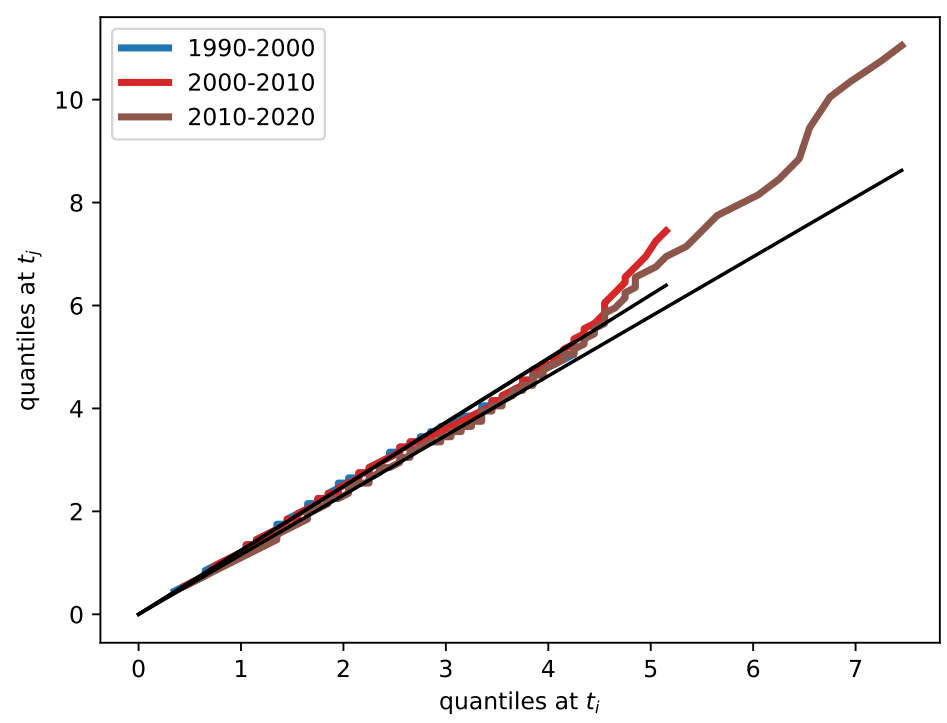

f)

Period (t_i-t_j)	$\frac{P(t_j)}{P(t_i)}$	Φ_{ij}	IQR
1990-2000	1.31	1.24	(1.22, 1.27)
2000-2010	1.20	1.24	(1.22, 1.26)
2010-2020	1.26	1.16	(1.13, 1.18)
1990-2010	1.56	1.55	(1.52, 1.58)
2000-2020	1.51	1.44	(1.40, 1.46)
1990-2020	1.97	1.80	(1.75, 1.84)

Supplementary Figure 37: Supplementary data for the metropolitan zone of Morelia with code 16.1.02. Remoteness values are those of 2020. a) Population difference by grid cell (2020–1990); city centres are denoted as black dots. b) Quantile-quantile plots for the radial population distributions $\rho(s, t_i)$ and $\rho(s, t_j)$ (coloured curves); urban expansion factors Φ_{ij} from t_i to t_j are the estimated slopes (black lines). c) Radial population distribution $\rho(r)$ at remoteness distance r from the city centre. d) Radial population density $\sigma(r)$ at remoteness distance r from the city centre. e) Average population density $\bar{\sigma}(r)$ within disks of remoteness r with the same centre as the city. f) Urban expansion factors and their inter quartile range from the Theil-Sen estimation.

Zamora, 16.1.03

a)

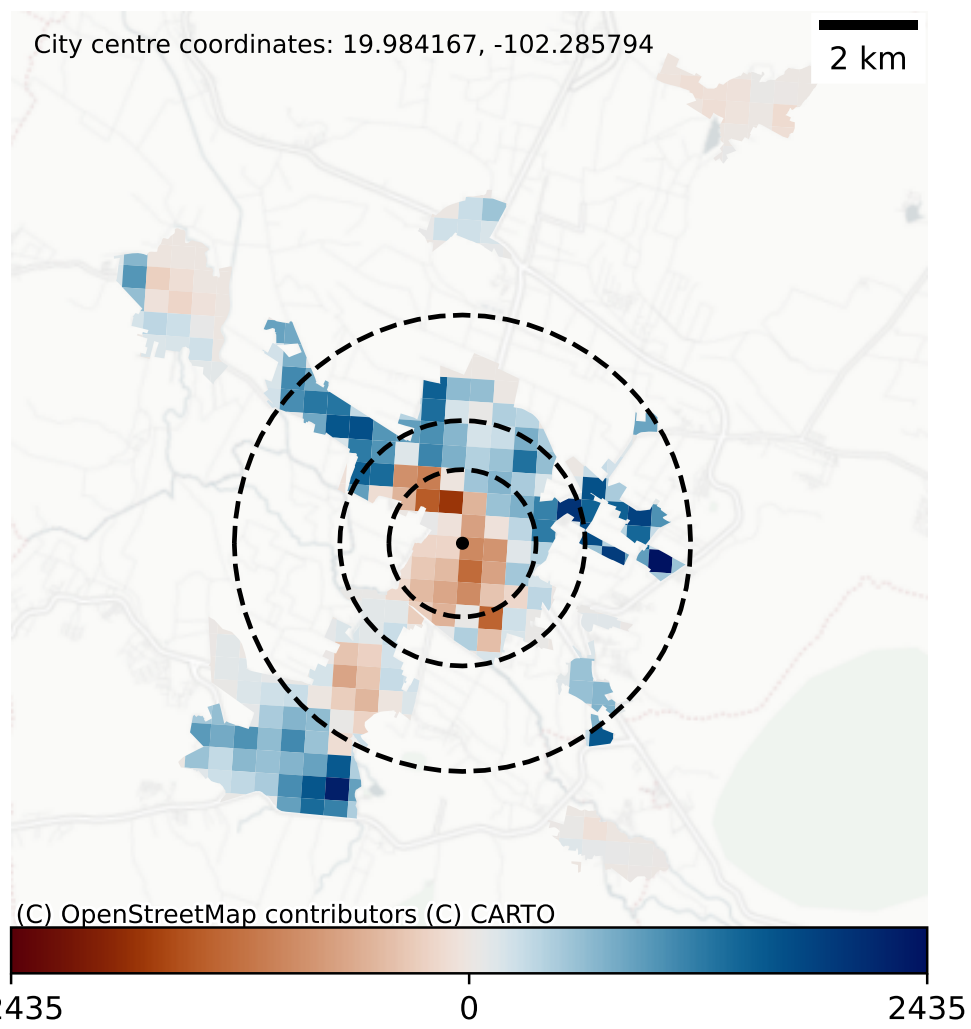

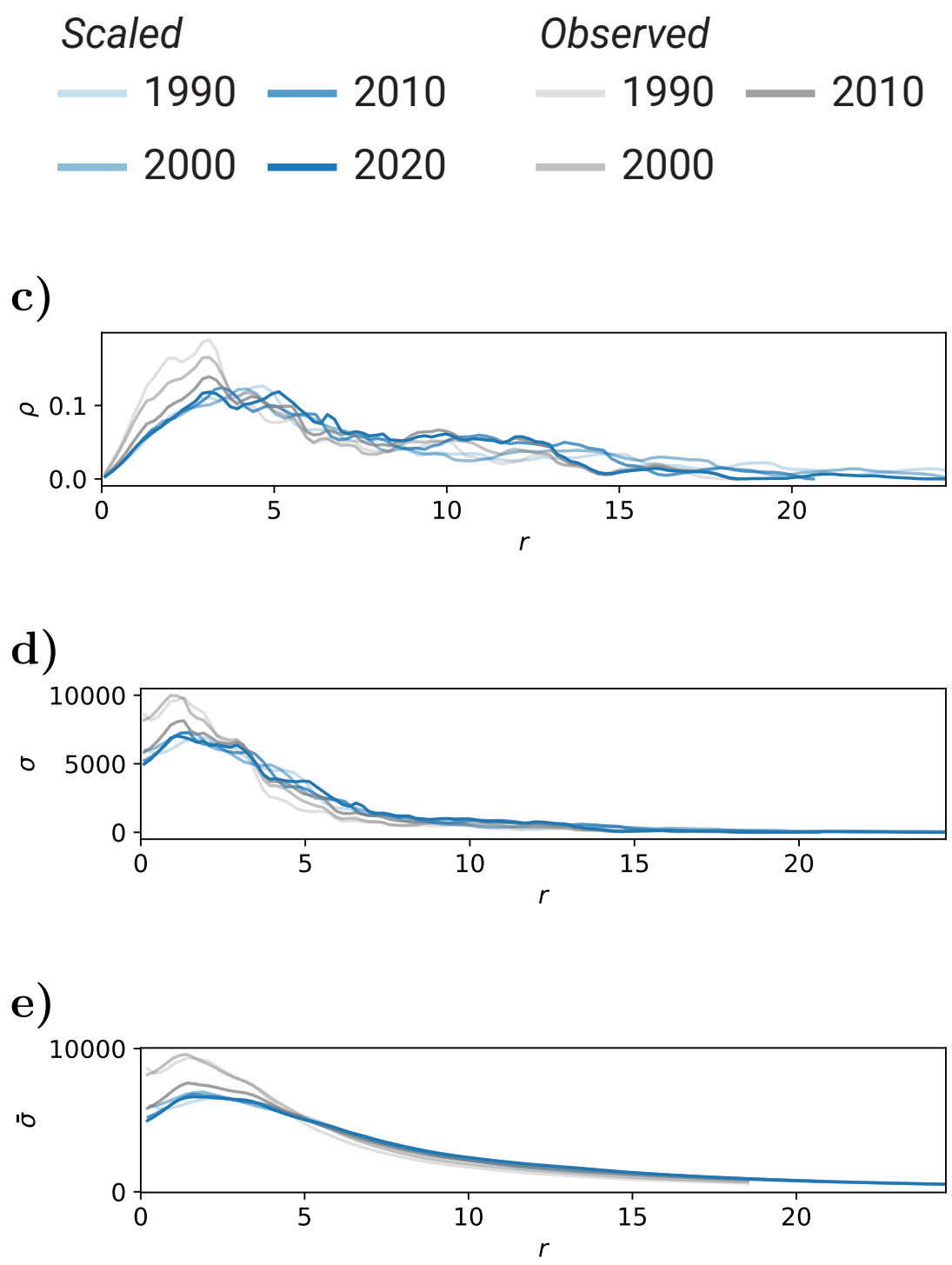

b)

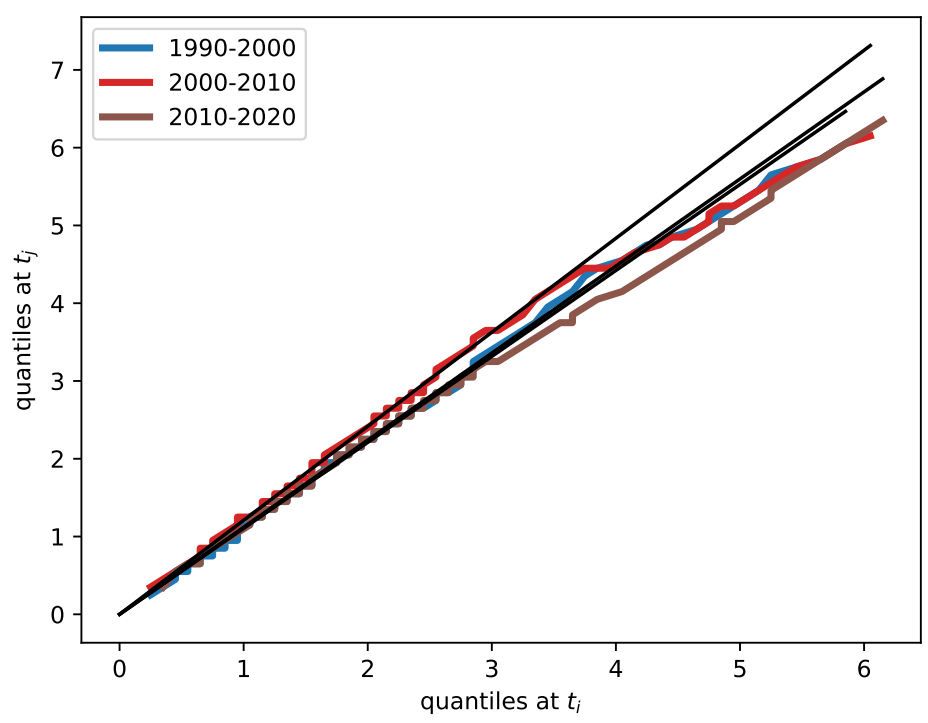

f)

Period (t_i-t_j)	$\frac{P(t_j)}{P(t_i)}$	Φ_{ij}	IQR
1990-2000	1.18	1.11	(1.07, 1.15)
2000-2010	1.18	1.21	(1.18, 1.24)
2010-2020	1.12	1.12	(1.10, 1.15)
1990-2010	1.39	1.33	(1.30, 1.38)
2000-2020	1.33	1.35	(1.32, 1.40)
1990-2020	1.57	1.48	(1.44, 1.53)

Supplementary Figure 38: Supplementary data for the metropolitan zone of Zamora with code 16.1.03. Remoteness values are those of 2020. a) Population difference by grid cell (2020–1990); city centres are denoted as black dots. b) Quantile-quantile plots for the radial population distributions $\rho(s, t_i)$ and $\rho(s, t_j)$ (coloured curves); urban expansion factors Φ_{ij} from t_i to t_j are the estimated slopes (black lines). c) Radial population distribution $\rho(r)$ at remoteness distance r from the city centre. d) Radial population density $\sigma(r)$ at remoteness distance r from the city centre. e) Average population density $\bar{\sigma}(r)$ within disks of remoteness r with the same centre as the city. f) Urban expansion factors and their inter quartile range from the Theil-Sen estimation.

Uruapan, 16.2.04

a)

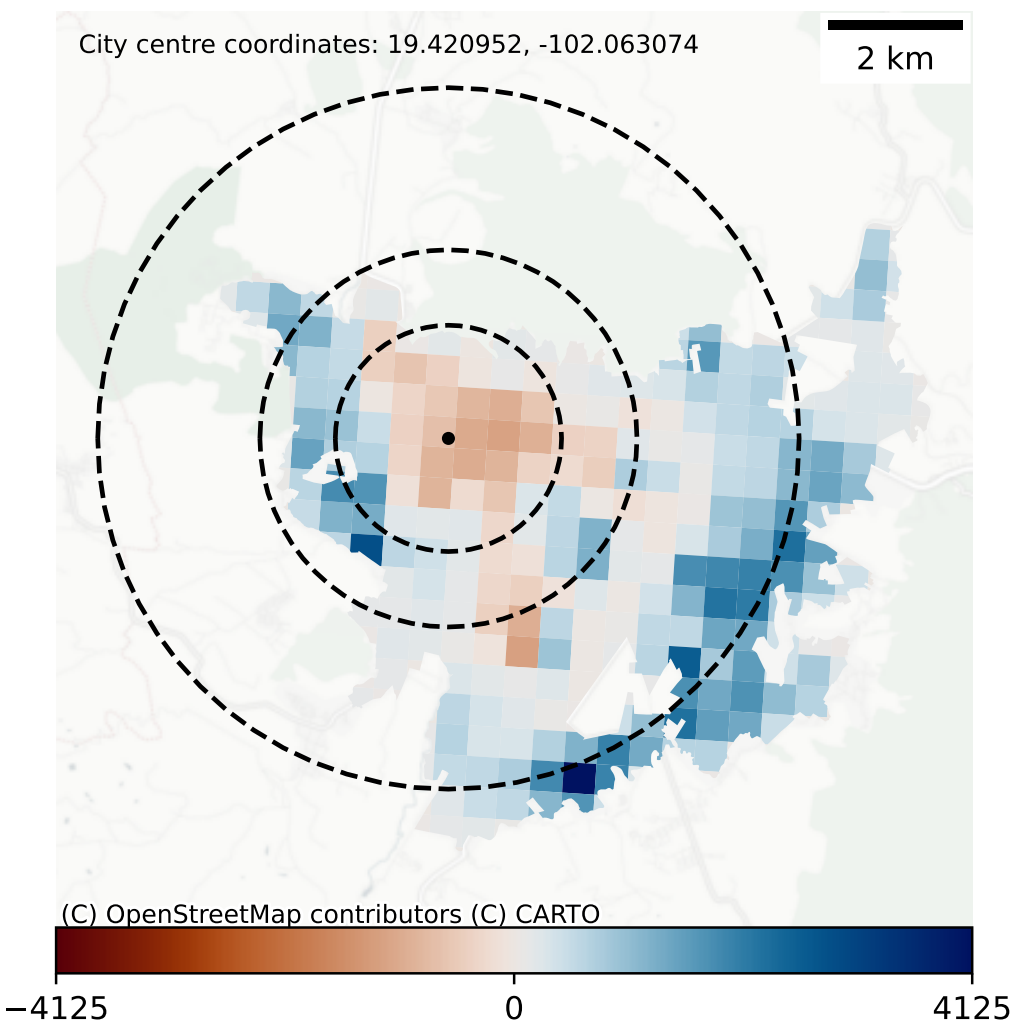

c)

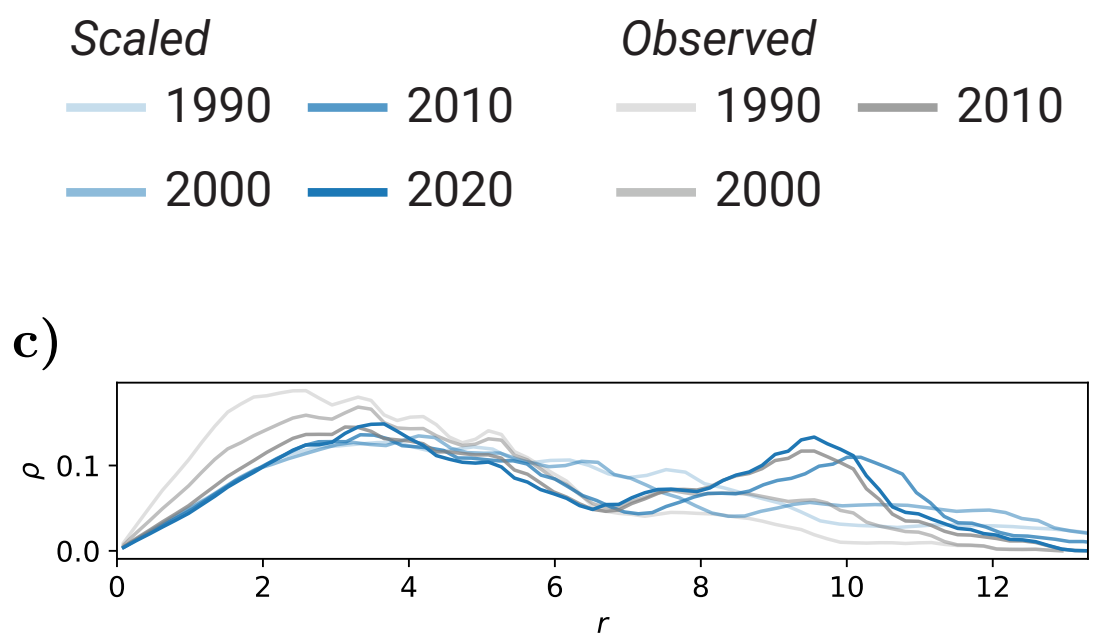

d)

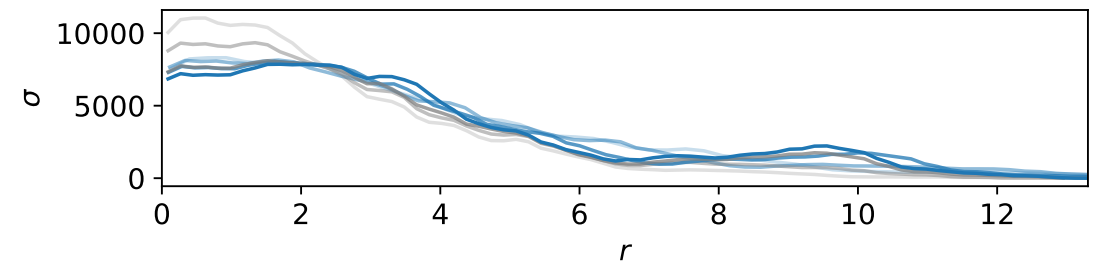

e)

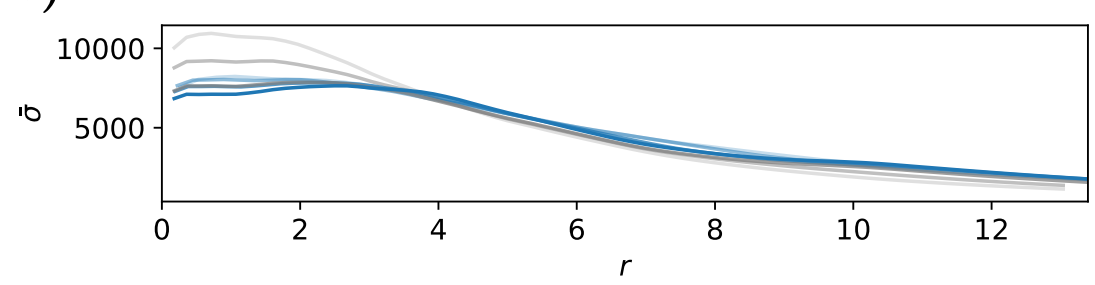

b)

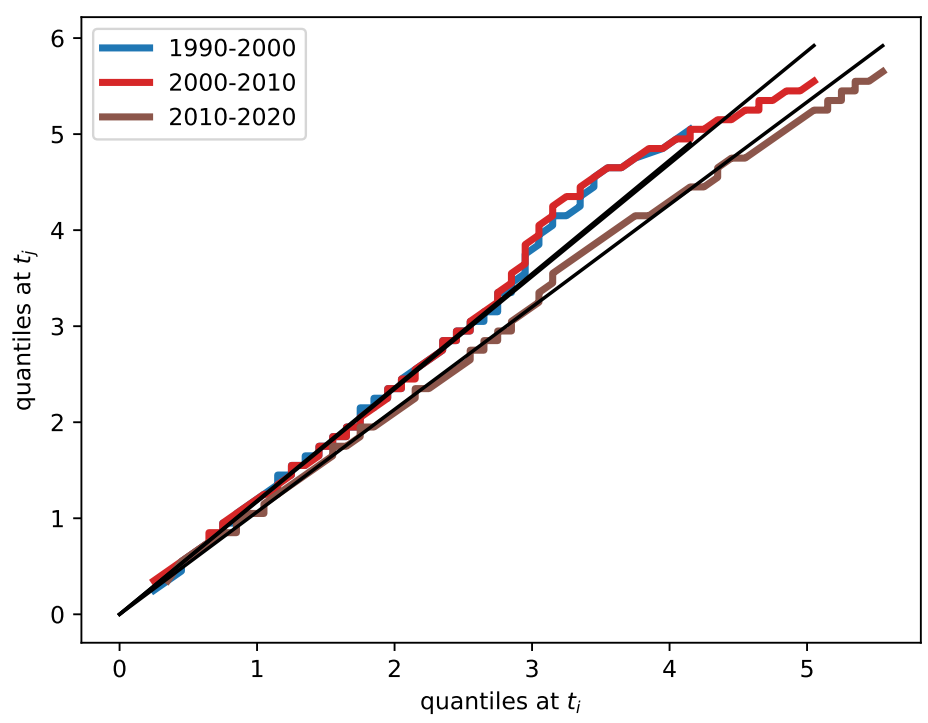

f)

Period (t_i-t_j)	$\frac{P(t_j)}{P(t_i)}$	Φ_{ij}	IQR
1990-2000	1.20	1.18	(1.15, 1.21)
2000-2010	1.20	1.17	(1.15, 1.21)
2010-2020	1.13	1.07	(1.05, 1.09)
1990-2010	1.45	1.38	(1.35, 1.42)
2000-2020	1.36	1.26	(1.22, 1.31)
1990-2020	1.64	1.48	(1.44, 1.53)

Supplementary Figure 39: Supplementary data for the metropolitan zone of Uruapan with code 16.2.04. Remoteness values are those of 2020. a) Population difference by grid cell (2020–1990); city centres are denoted as black dots. b) Quantile-quantile plots for the radial population distributions $\rho(s, t_i)$ and $\rho(s, t_j)$ (coloured curves); urban expansion factors Φ_{ij} from t_i to t_j are the estimated slopes (black lines). c) Radial population distribution $\rho(r)$ at remoteness distance r from the city centre. d) Radial population density $\sigma(r)$ at remoteness distance r from the city centre. e) Average population density $\bar{\sigma}(r)$ within disks of remoteness r with the same centre as the city. f) Urban expansion factors and their inter quartile range from the Theil-Sen estimation.

Cuautla, 17.1.01

a)

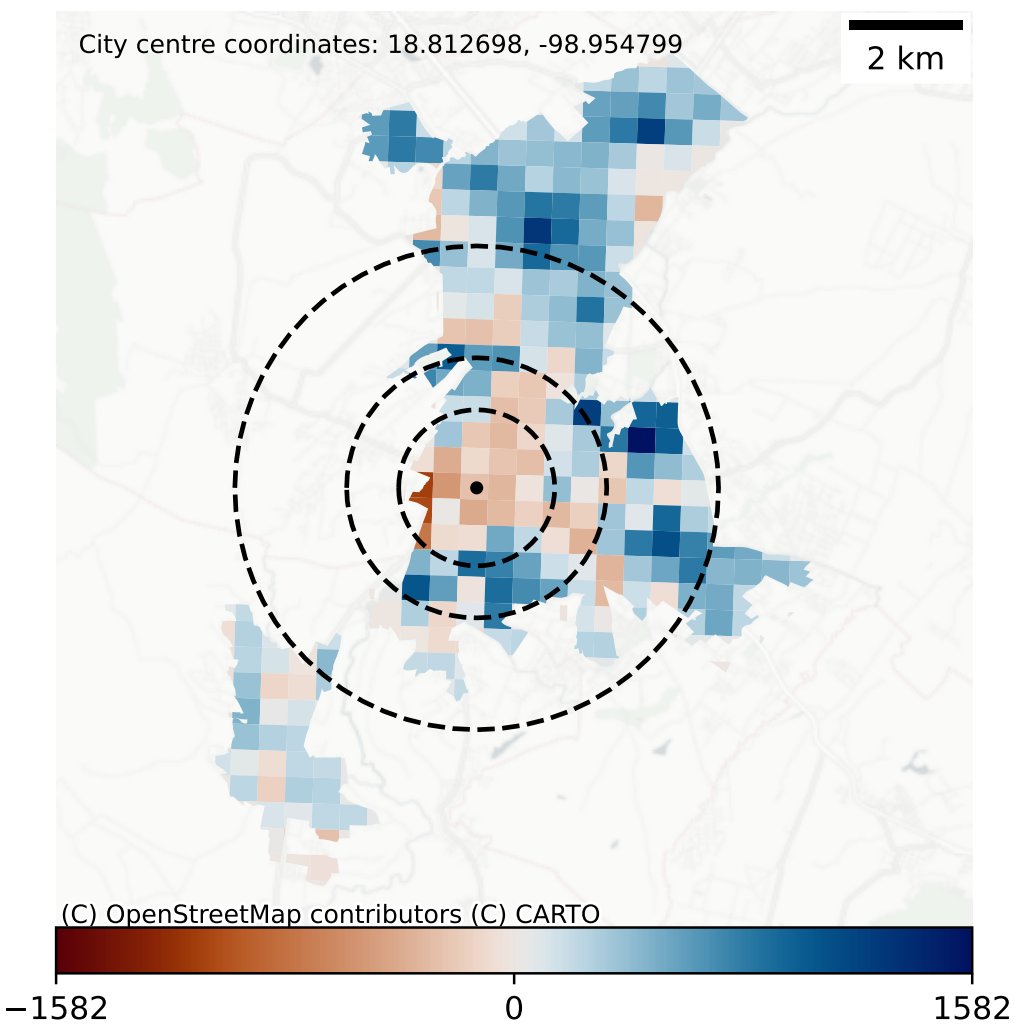

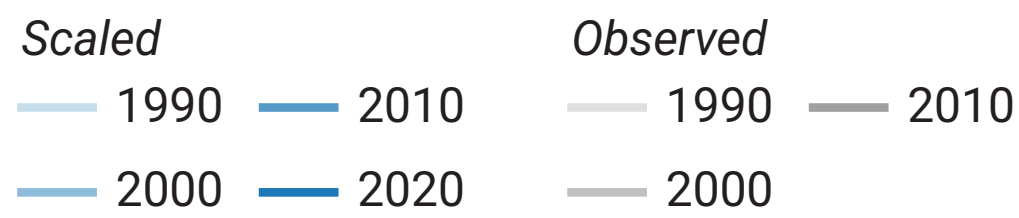

c)

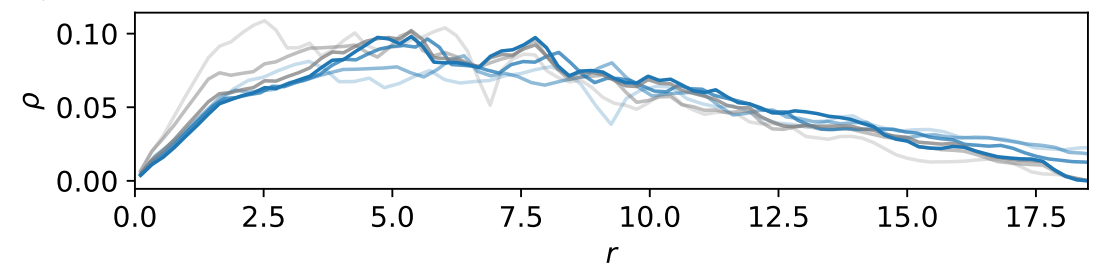

d)

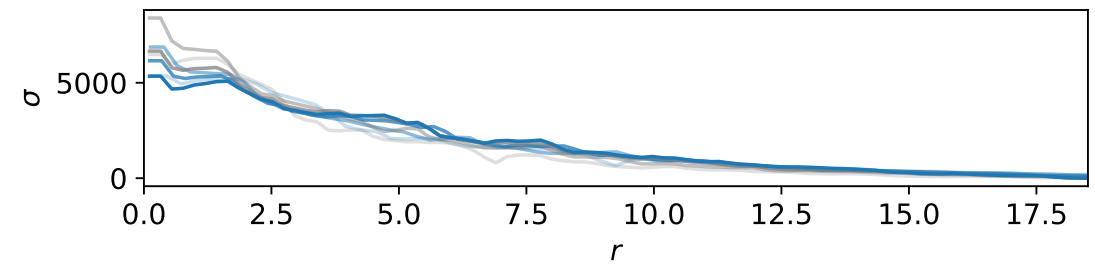

e)

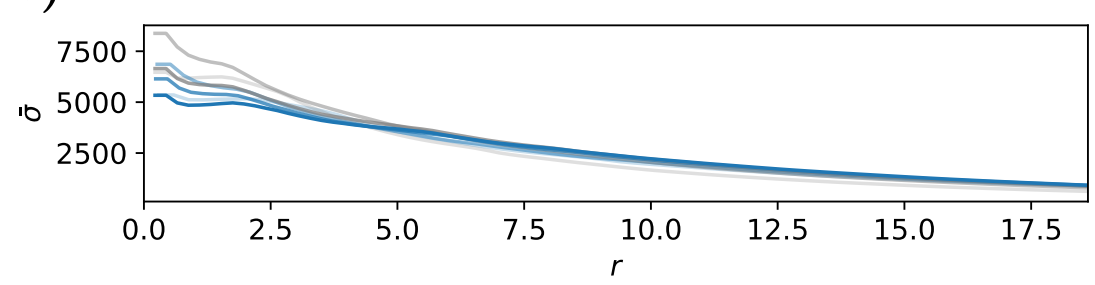

b)

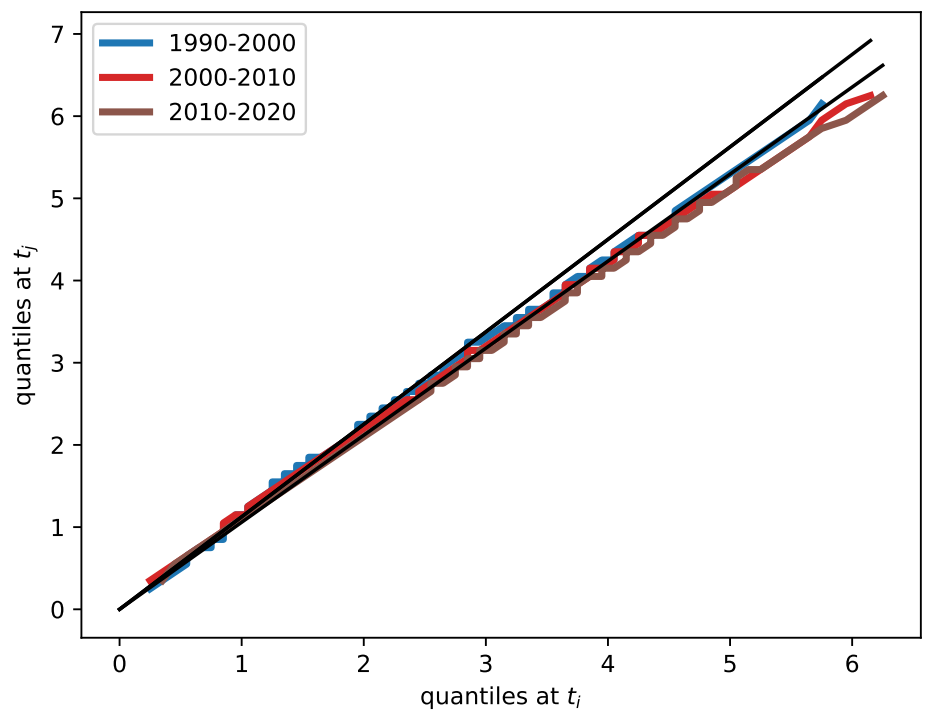

f)

Period (t_i-t_j)	$\frac{P(t_j)}{P(t_i)}$	Φ_{ij}	IQR
1990-2000	1.28	1.13	(1.10, 1.16)
2000-2010	1.12	1.13	(1.10, 1.16)
2010-2020	1.04	1.06	(1.05, 1.09)
1990-2010	1.44	1.27	(1.22, 1.34)
2000-2020	1.16	1.19	(1.15, 1.27)
1990-2020	1.49	1.38	(1.29, 1.43)

Supplementary Figure 40: Supplementary data for the metropolitan zone of Cuautla with code 17.1.01. Remoteness values are those of 2020. a) Population difference by grid cell (2020–1990); city centres are denoted as black dots. b) Quantile-quantile plots for the radial population distributions $\rho(s, t_i)$ and $\rho(s, t_j)$ (coloured curves); urban expansion factors Φ_{ij} from t_i to t_j are the estimated slopes (black lines). c) Radial population distribution $\rho(r)$ at remoteness distance r from the city centre. d) Radial population density $\sigma(r)$ at remoteness distance r from the city centre. e) Average population density $\bar{\sigma}(r)$ within disks of remoteness r with the same centre as the city. f) Urban expansion factors and their inter quartile range from the Theil-Sen estimation.

Cuernavaca, 17.1.02

a)

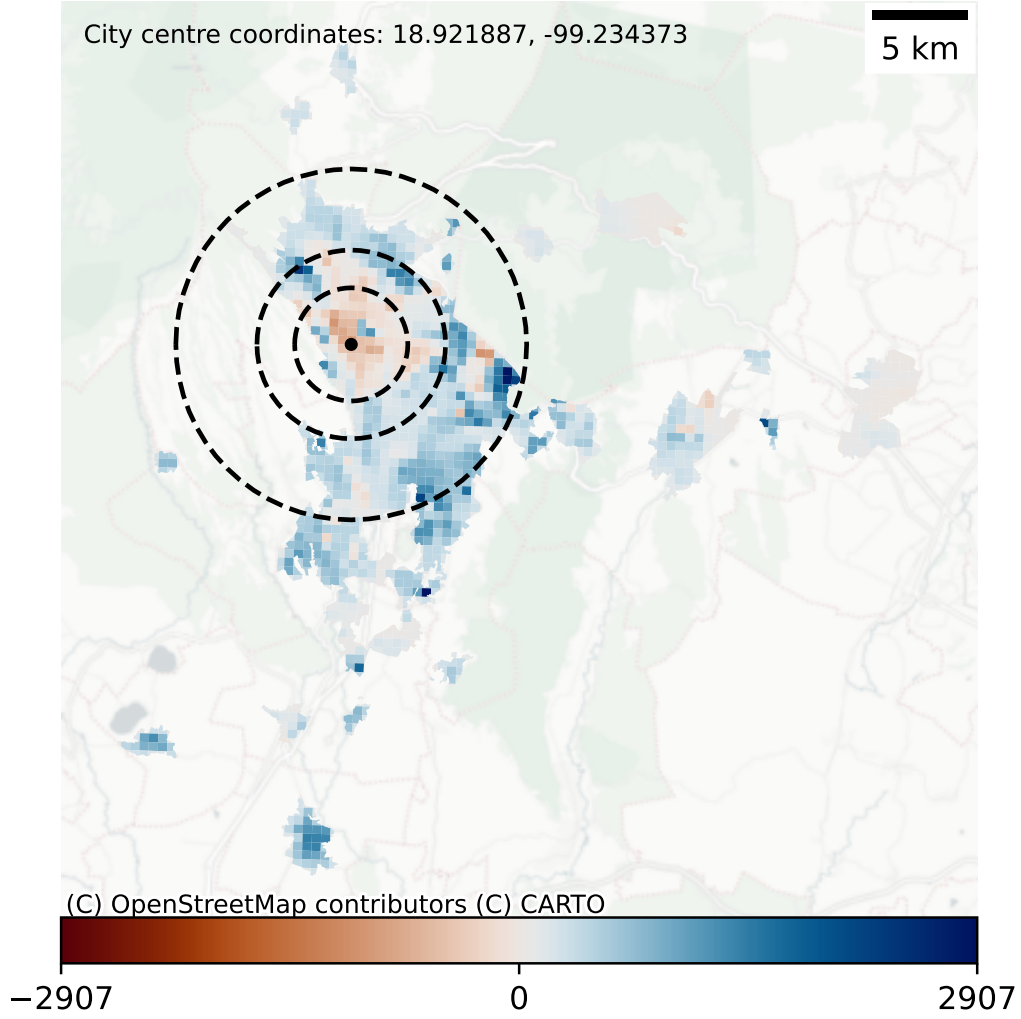

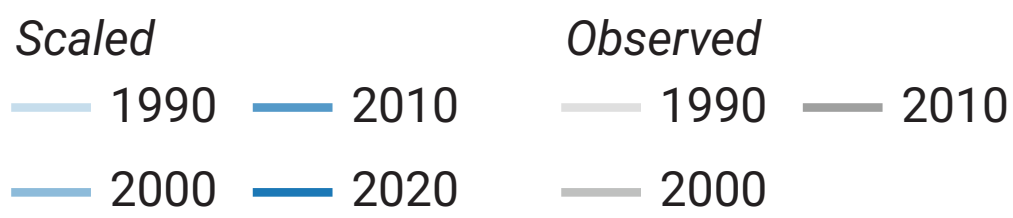

c)

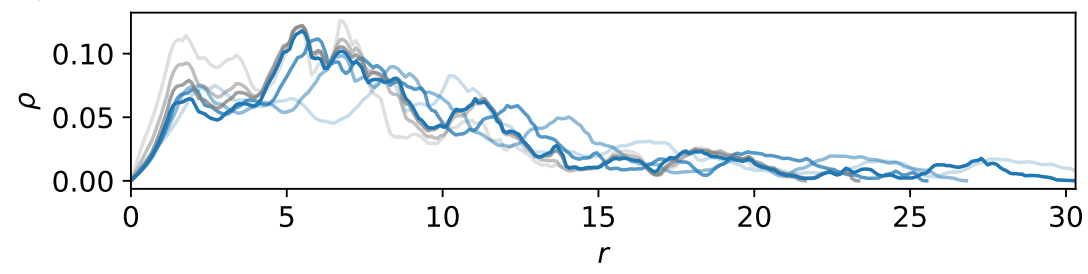

d)

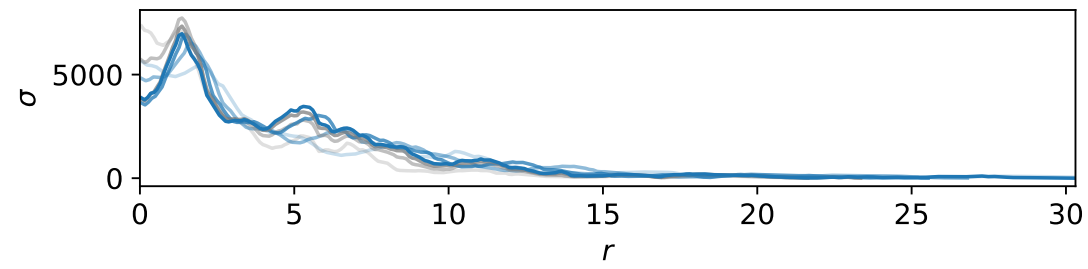

e)

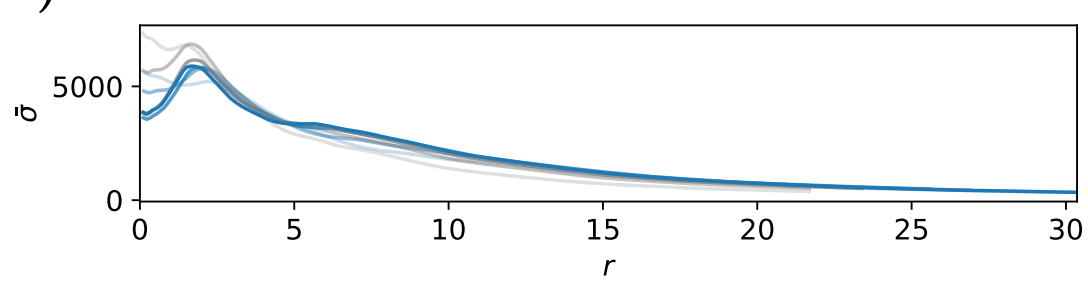

b)

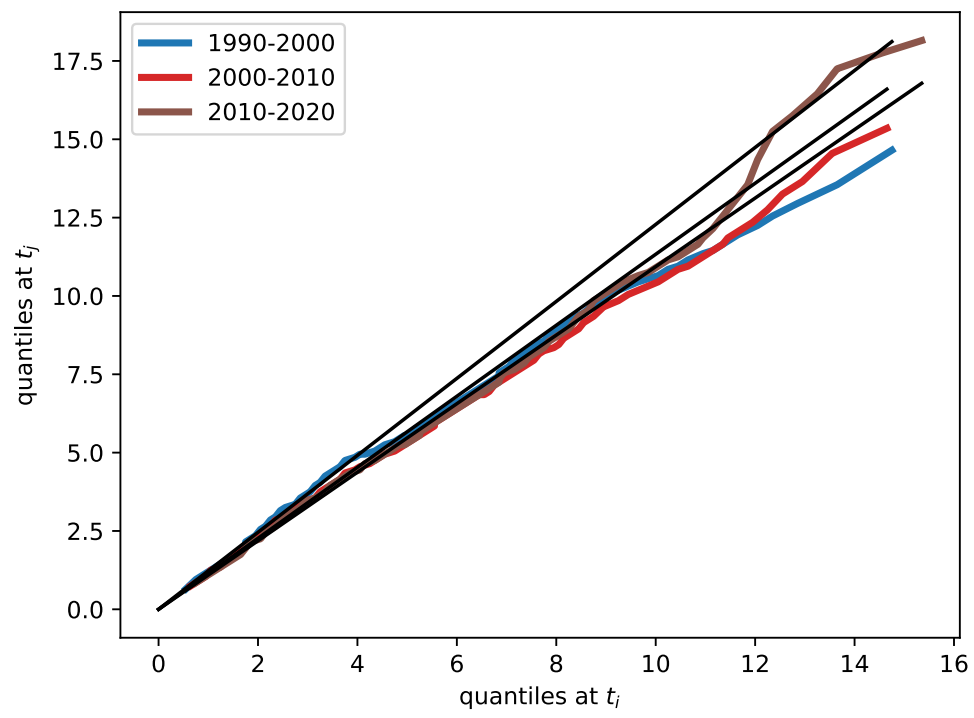

f)

Period (t_i-t_j)	$\frac{P(t_j)}{P(t_i)}$	Φ_{ij}	IQR
1990-2000	1.37	1.23	(1.17, 1.25)
2000-2010	1.14	1.13	(1.09, 1.16)
2010-2020	1.13	1.09	(1.07, 1.13)
1990-2010	1.56	1.37	(1.30, 1.42)
2000-2020	1.29	1.26	(1.16, 1.31)
1990-2020	1.77	1.51	(1.43, 1.59)

Supplementary Figure 41: Supplementary data for the metropolitan zone of Cuernavaca with code 17.1.02. Remoteness values are those of 2020. a) Population difference by grid cell (2020–1990); city centres are denoted as black dots. b) Quantile-quantile plots for the radial population distributions $\rho(s,t_i)$ and $\rho(s,t_j)$ (coloured curves); urban expansion factors Φ_{ij} from t_i to t_j are the estimated slopes (black lines). c) Radial population distribution $\rho(r)$ at remoteness distance r from the city centre. d) Radial population density $\sigma(r)$ at remoteness distance r from the city centre. e) Average population density $\bar{\sigma}(r)$ within disks of remoteness r with the same centre as the city. f) Urban expansion factors and their inter quartile range from the Theil-Sen estimation.

Tepic, 18.1.01

a)

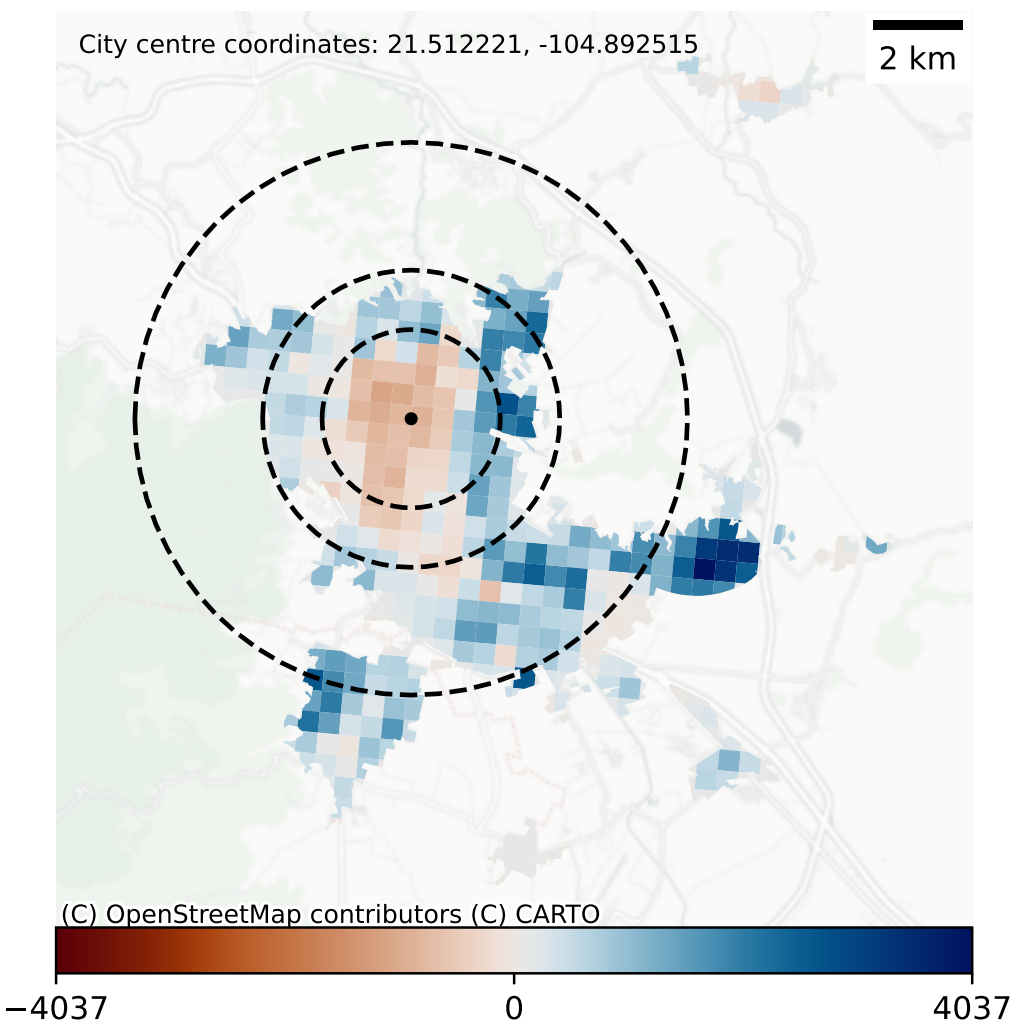

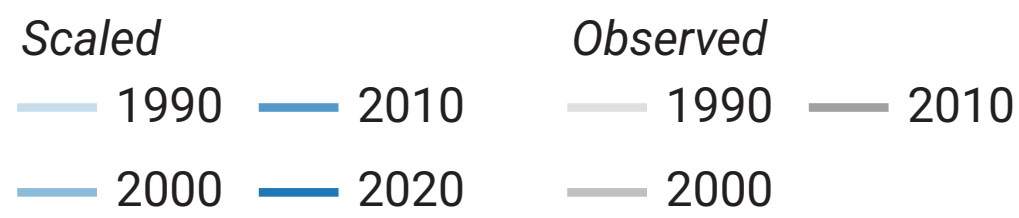

c)

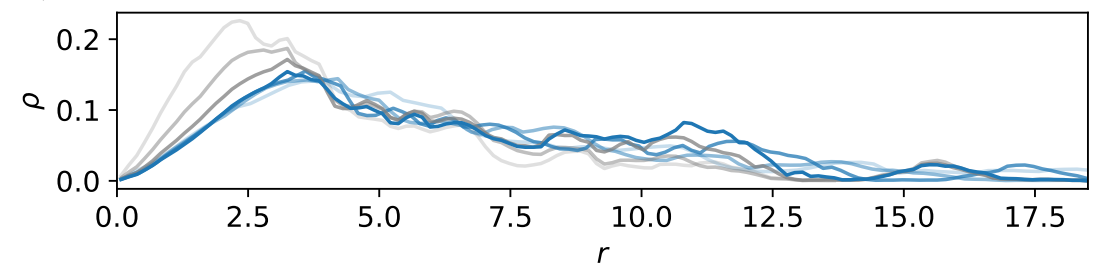

d)

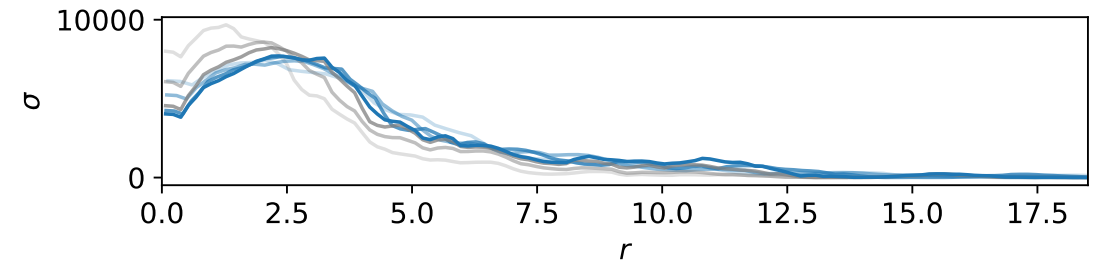

e)

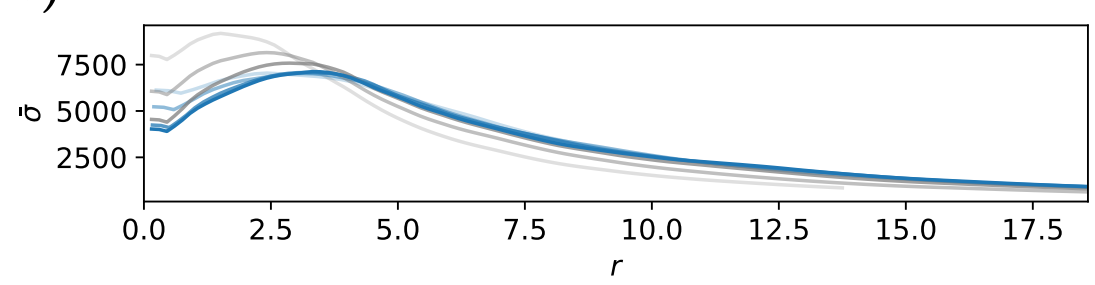

b)

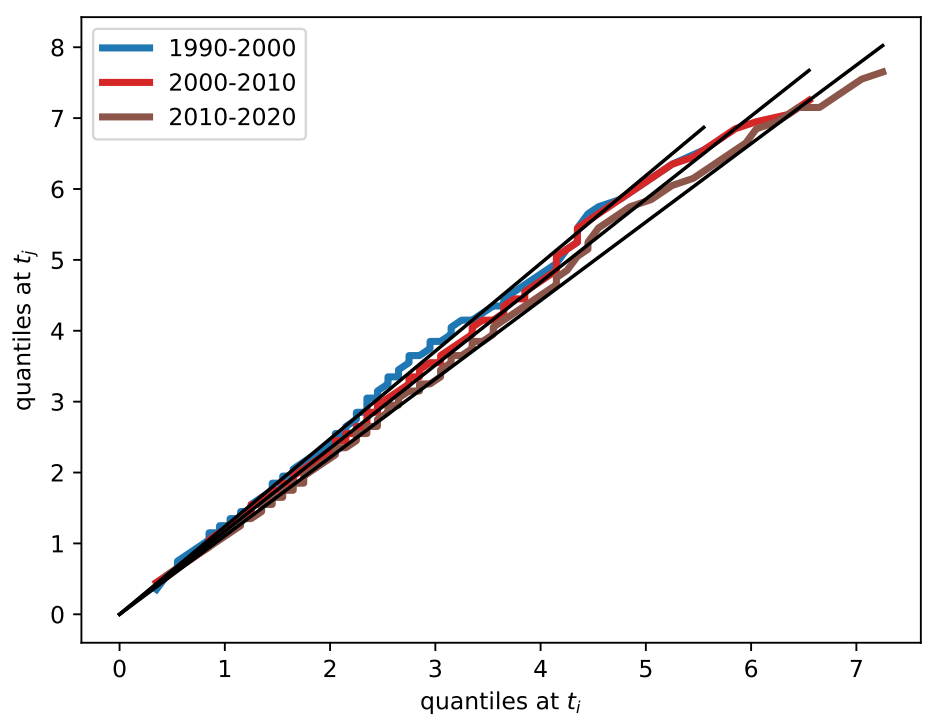

f)

Period (t_i-t_j)	$\frac{P(t_j)}{P(t_i)}$	Φ_{ij}	IQR
1990-2000	1.36	1.24	(1.21, 1.27)
2000-2010	1.27	1.17	(1.15, 1.19)
2010-2020	1.14	1.11	(1.09, 1.13)
1990-2010	1.73	1.44	(1.41, 1.48)
2000-2020	1.45	1.29	(1.27, 1.32)
1990-2020	1.97	1.60	(1.56, 1.63)

Supplementary Figure 42: Supplementary data for the metropolitan zone of Tepic with code 18.1.01. Remoteness values are those of 2020. a) Population difference by grid cell (2020–1990); city centres are denoted as black dots. b) Quantile-quantile plots for the radial population distributions $\rho(s, t_i)$ and $\rho(s, t_j)$ (coloured curves); urban expansion factors Φ_{ij} from t_i to t_j are the estimated slopes (black lines). c) Radial population distribution $\rho(r)$ at remoteness distance r from the city centre. d) Radial population density $\sigma(r)$ at remoteness distance r from the city centre. e) Average population density $\bar{\sigma}(r)$ within disks of remoteness r with the same centre as the city. f) Urban expansion factors and their inter quartile range from the Theil-Sen estimation.

Monterrey, 19.1.01

a)

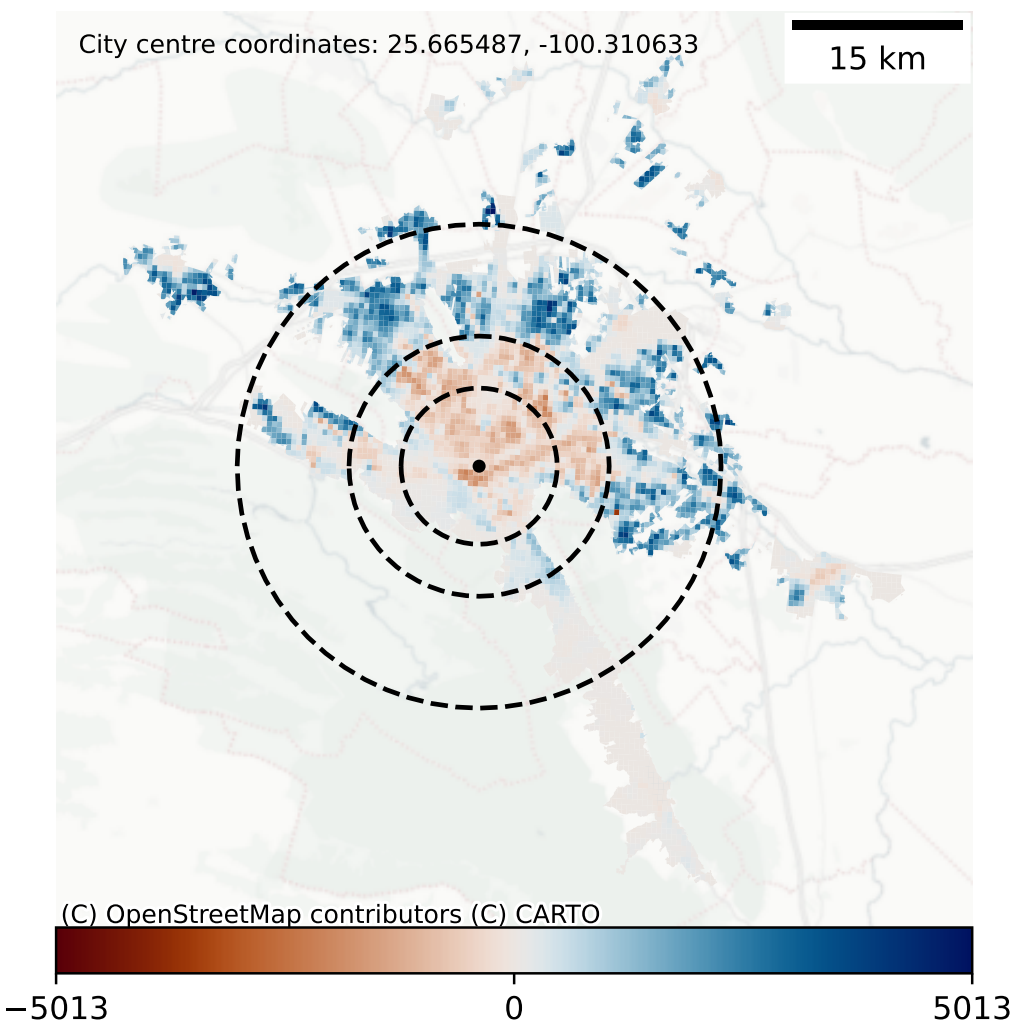

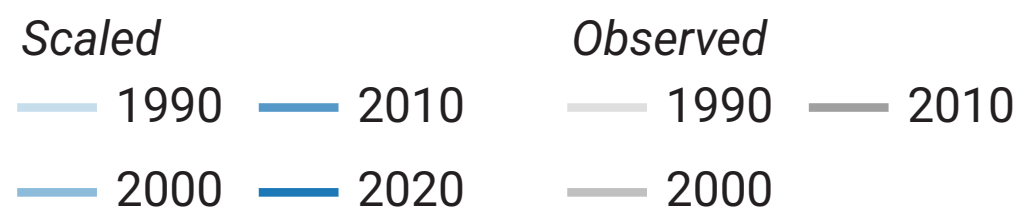

b)

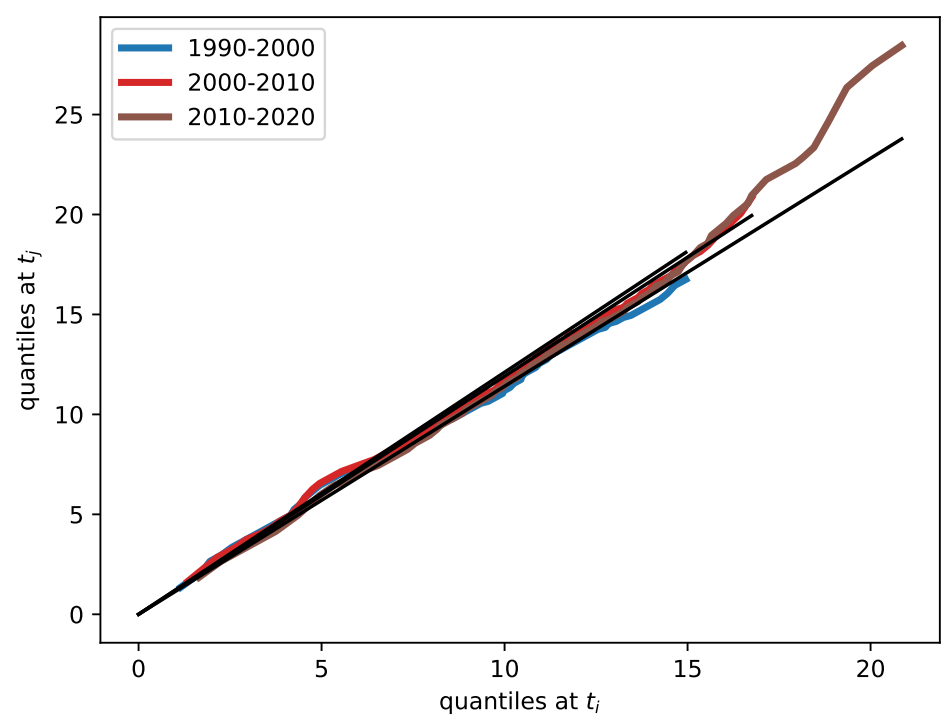

c)

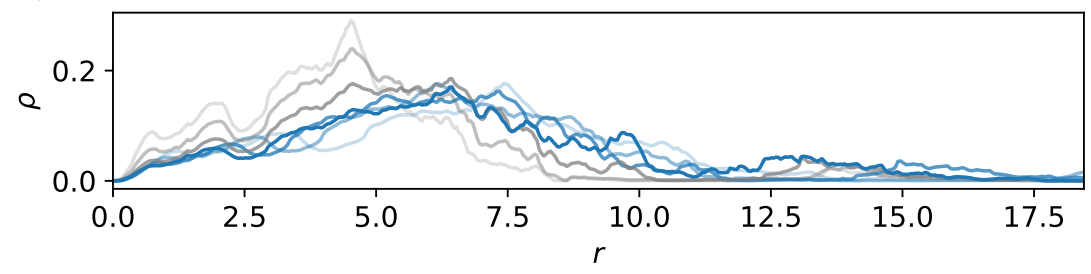

d)

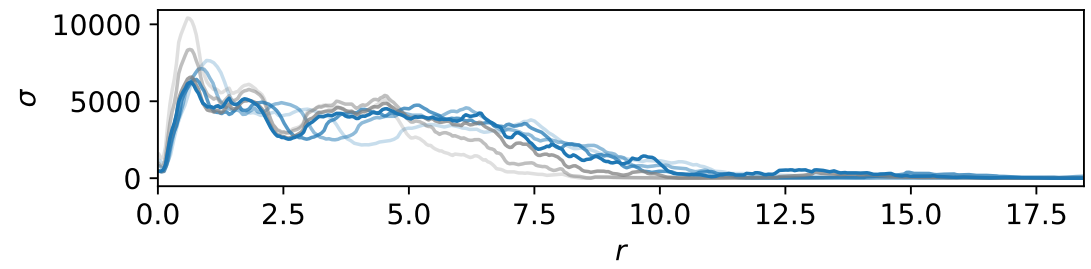

e)

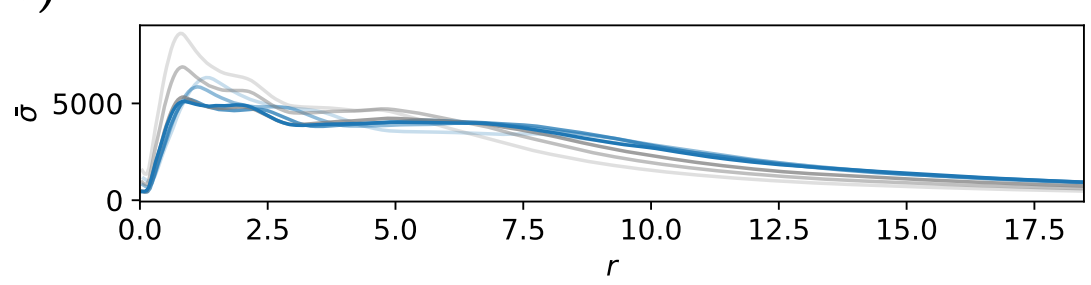

f)

Period (t_i-t_j)	$\frac{P(t_j)}{P(t_i)}$	Φ_{ij}	IQR
1990-2000	1.27	1.21	(1.16, 1.27)
2000-2010	1.24	1.19	(1.17, 1.25)
2010-2020	1.27	1.14	(1.13, 1.15)
1990-2010	1.57	1.48	(1.36, 1.58)
2000-2020	1.57	1.35	(1.34, 1.42)
1990-2020	1.99	1.72	(1.55, 1.81)

Supplementary Figure 43: Supplementary data for the metropolitan zone of Monterrey with code 19.1.01. Remoteness values are those of 2020. a) Population difference by grid cell (2020–1990); city centres are denoted as black dots. b) Quantile-quantile plots for the radial population distributions $\rho(s, t_i)$ and $\rho(s, t_j)$ (coloured curves); urban expansion factors Φ_{ij} from t_i to t_j are the estimated slopes (black lines). c) Radial population distribution $\rho(r)$ at remoteness distance r from the city centre. d) Radial population density $\sigma(r)$ at remoteness distance r from the city centre. e) Average population density $\bar{\sigma}(r)$ within disks of remoteness r with the same centre as the city. f) Urban expansion factors and their inter quartile range from the Theil-Sen estimation.

Oaxaca, 20.1.01

a)

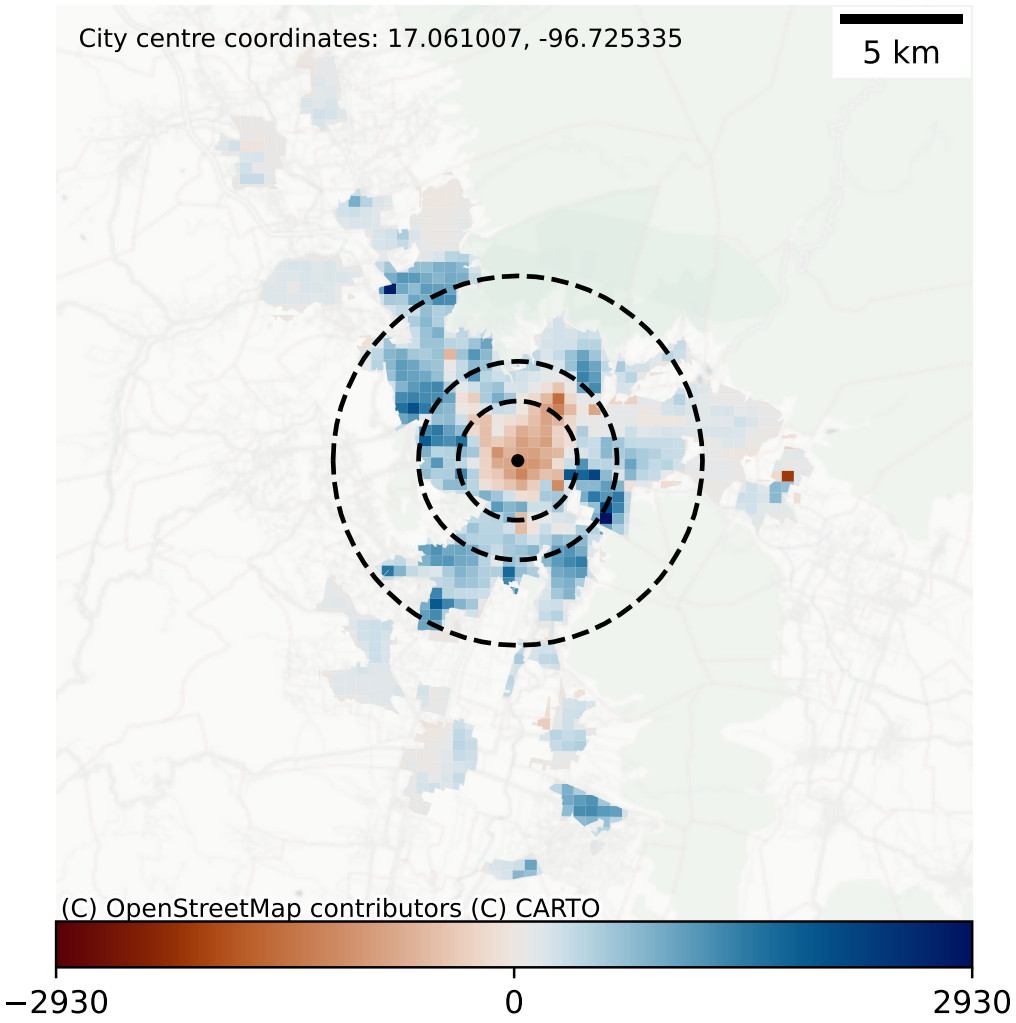

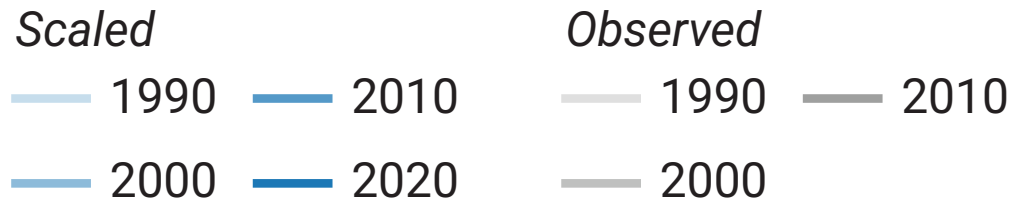

c)

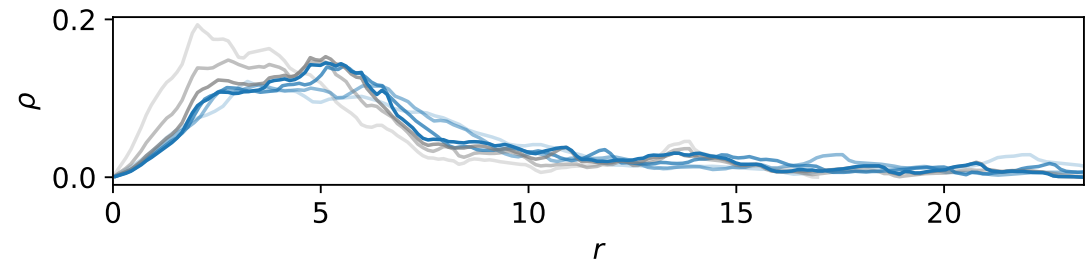

d)

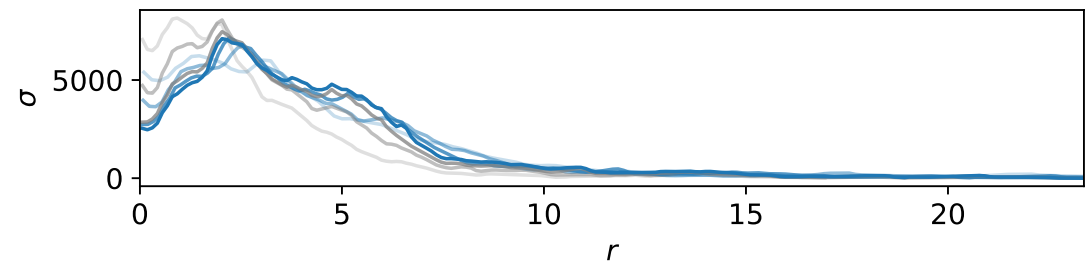

e)

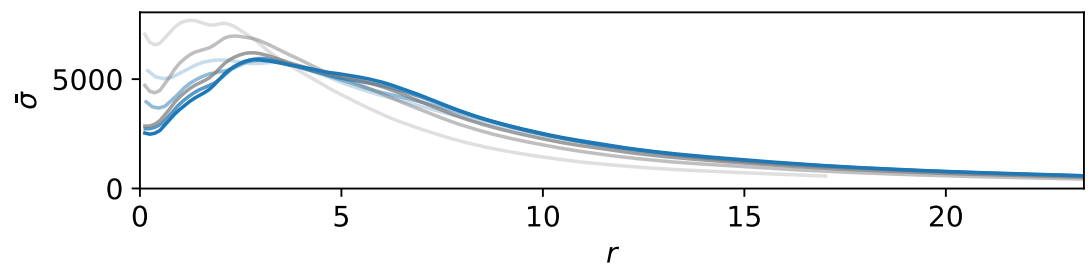

b)

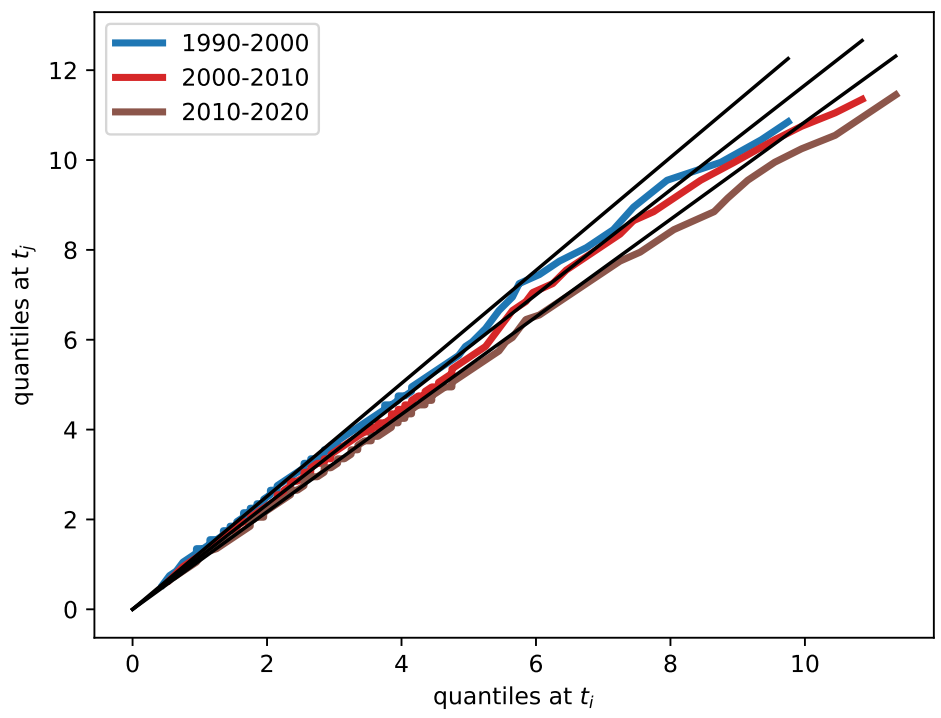

f)

Period (t_i-t_j)	$\frac{P(t_j)}{P(t_i)}$	Φ_{ij}	IQR
1990-2000	1.44	1.26	(1.24, 1.29)
2000-2010	1.19	1.17	(1.15, 1.18)
2010-2020	1.13	1.08	(1.07, 1.10)
1990-2010	1.72	1.46	(1.44, 1.51)
2000-2020	1.35	1.26	(1.24, 1.29)
1990-2020	1.94	1.60	(1.57, 1.63)

Supplementary Figure 44: Supplementary data for the metropolitan zone of Oaxaca with code 20.1.01. Remoteness values are those of 2020. a) Population difference by grid cell (2020–1990); city centres are denoted as black dots. b) Quantile-quantile plots for the radial population distributions $\rho(s,t_i)$ and $\rho(s,t_j)$ (coloured curves); urban expansion factors Φ_{ij} from t_i to t_j are the estimated slopes (black lines). c) Radial population distribution $\rho(r)$ at remoteness distance r from the city centre. d) Radial population density $\sigma(r)$ at remoteness distance r from the city centre. e) Average population density $\bar{\sigma}(r)$ within disks of remoteness r with the same centre as the city. f) Urban expansion factors and their inter quartile range from the Theil-Sen estimation.

Puebla-Tlaxcala, 21.1.01

a)

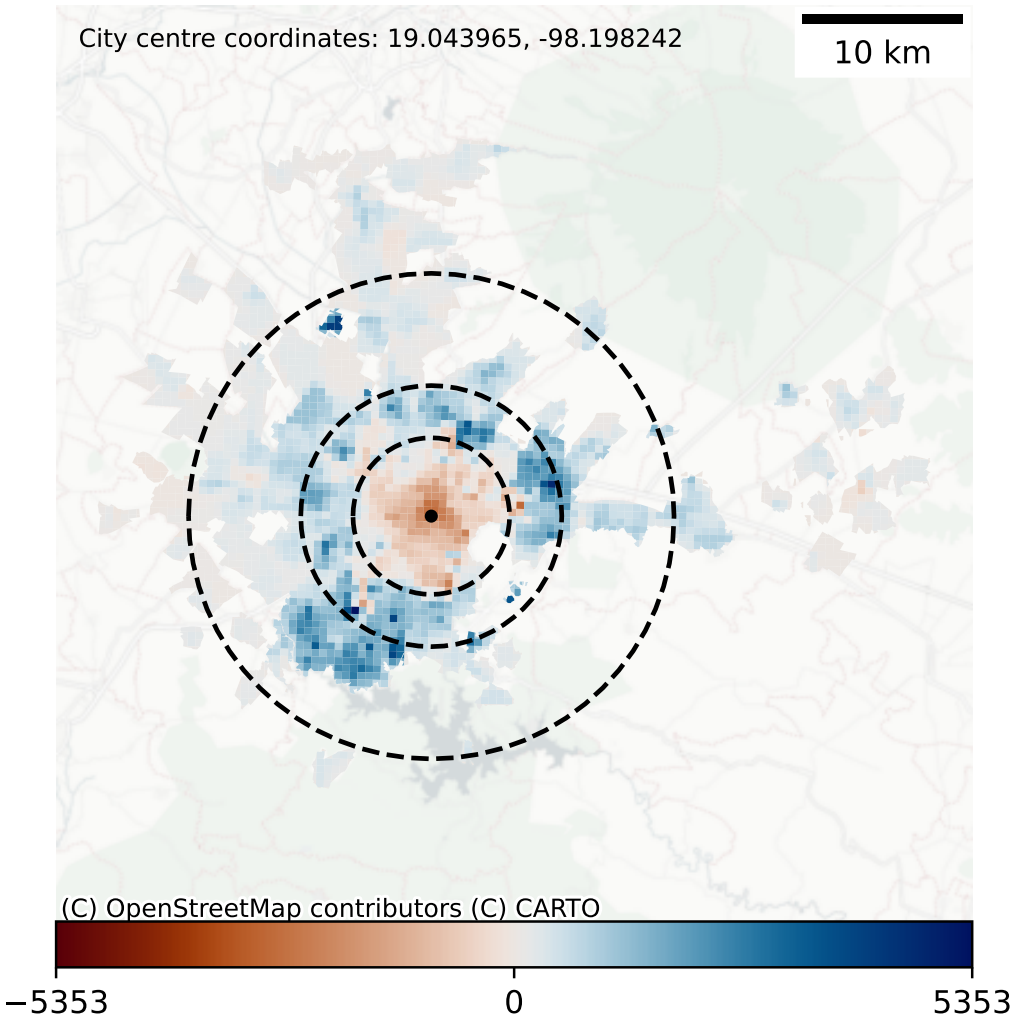

b)

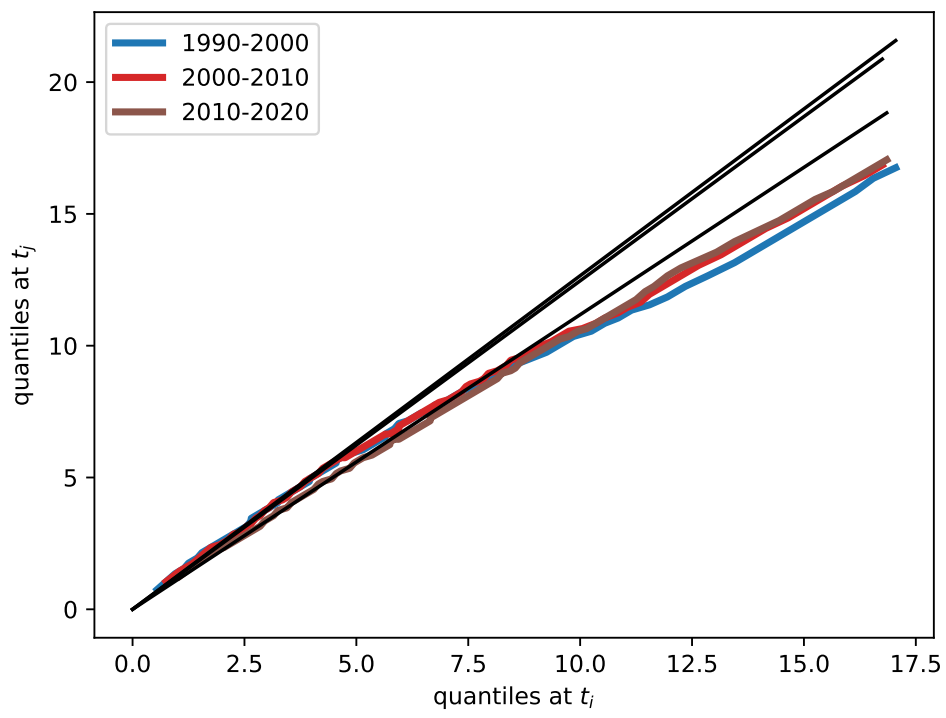

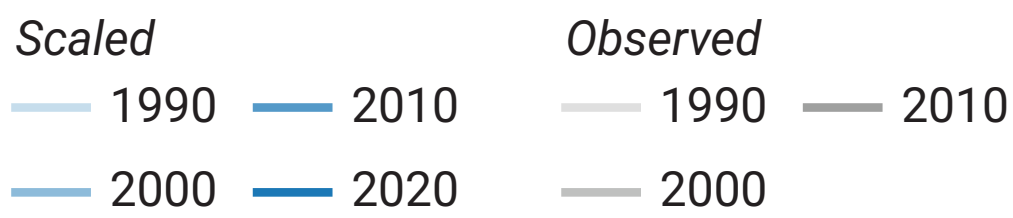

c)

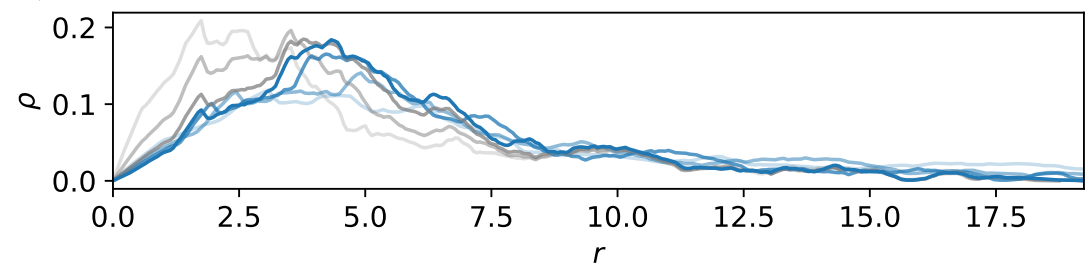

d)

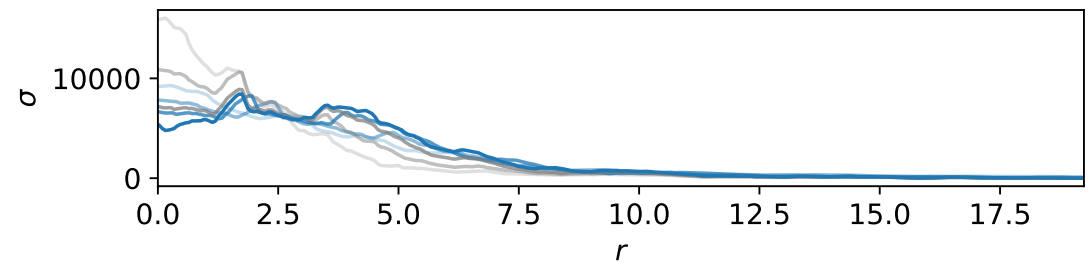

e)

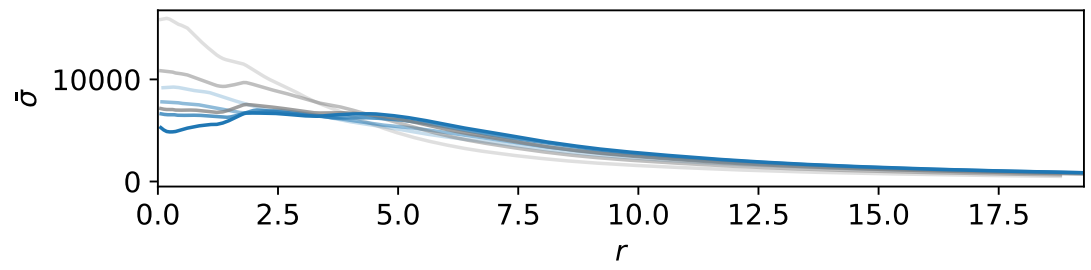

f)

Period (t_i-t_j)	$\frac{P(t_j)}{P(t_i)}$	Φ_{ij}	IQR
1990-2000	1.29	1.27	(1.25, 1.31)
2000-2010	1.20	1.25	(1.23, 1.26)
2010-2020	1.16	1.12	(1.09, 1.13)
1990-2010	1.55	1.58	(1.55, 1.61)
2000-2020	1.40	1.39	(1.35, 1.42)
1990-2020	1.80	1.78	(1.69, 1.83)

Supplementary Figure 45: Supplementary data for the metropolitan zone of Puebla-Tlaxcala with code 21.1.01. Remoteness values are those of 2020. a) Population difference by grid cell (2020–1990); city centres are denoted as black dots. b) Quantile-quantile plots for the radial population distributions $\rho(s,t_i)$ and $\rho(s,t_j)$ (coloured curves); urban expansion factors Φ_{ij} from t_i to t_j are the estimated slopes (black lines). c) Radial population distribution $\rho(r)$ at remoteness distance r from the city centre. d) Radial population density $\sigma(r)$ at remoteness distance r from the city centre. e) Average population density $\bar{\sigma}(r)$ within disks of remoteness r with the same centre as the city. f) Urban expansion factors and their inter quartile range from the Theil-Sen estimation.

San Martín Texmelucan, 21.1.02

a)

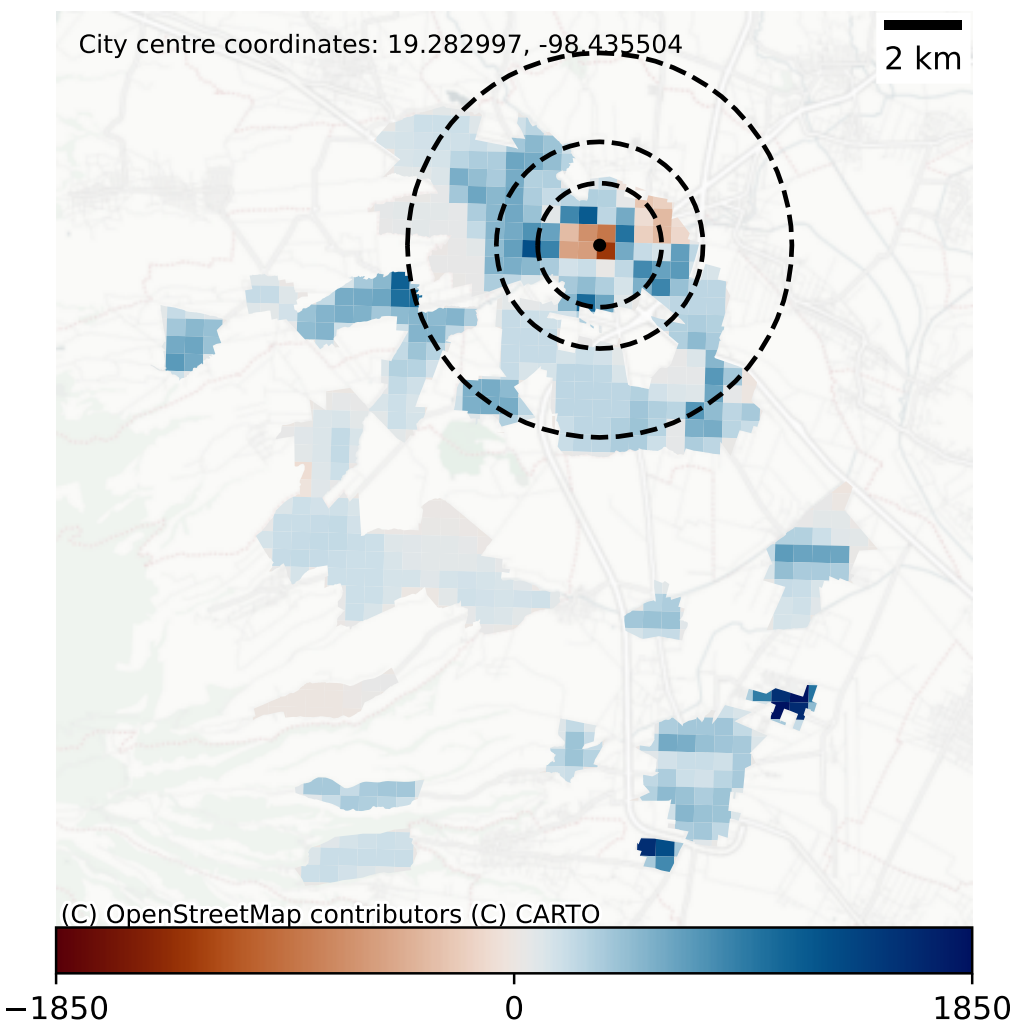

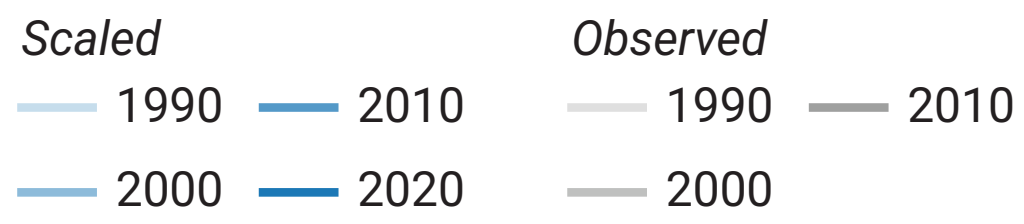

c)

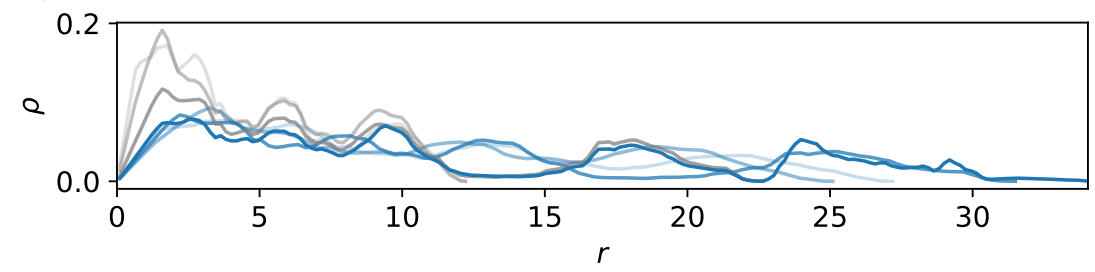

d)

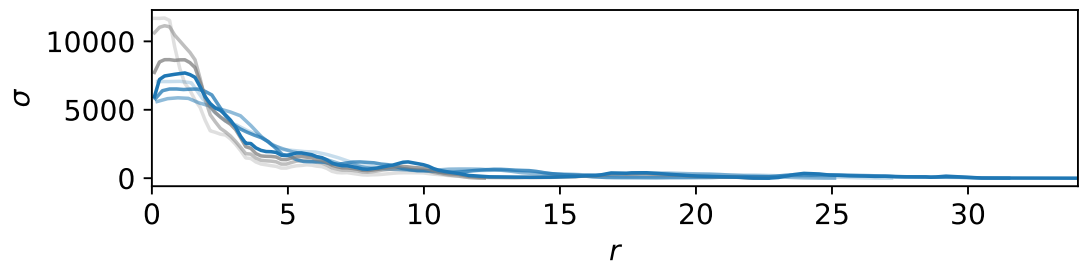

e)

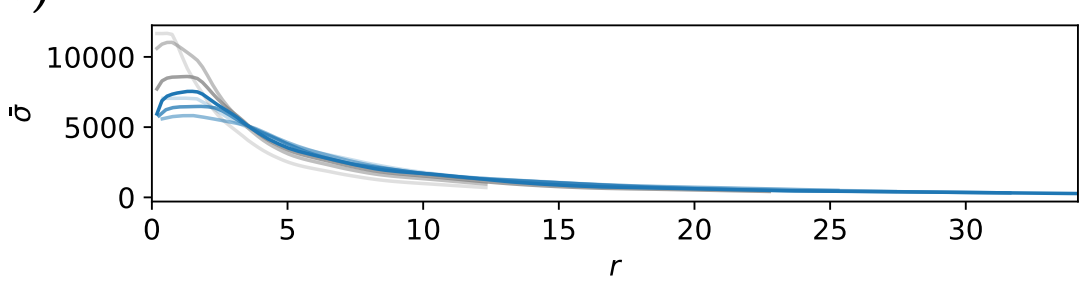

b)

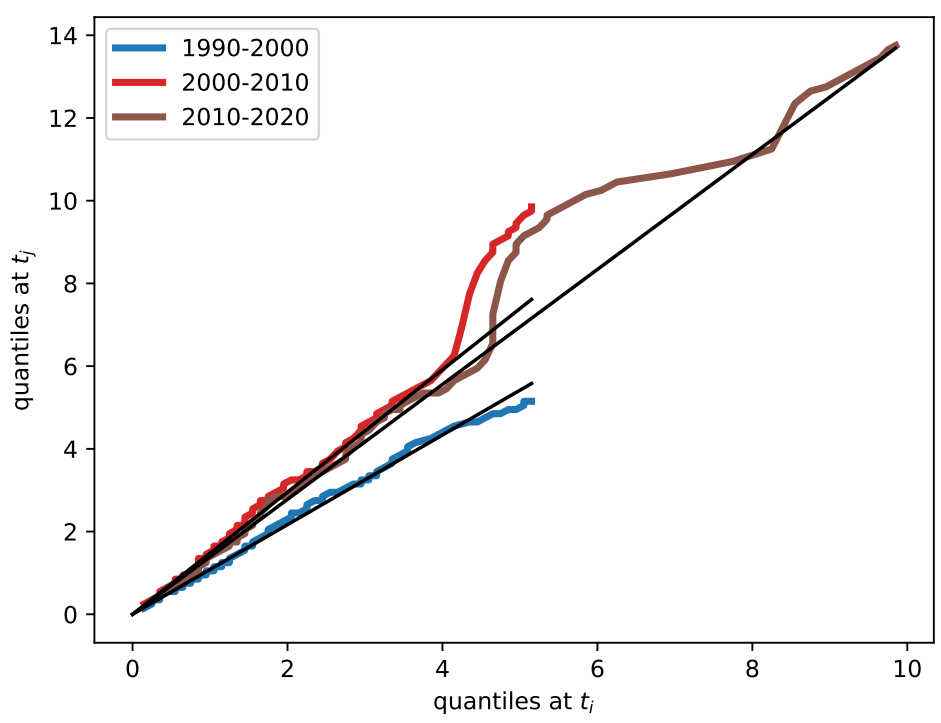

f)

Period (t_i-t_j)	$\frac{P(t_j)}{P(t_i)}$	Φ_{ij}	IQR
1990-2000	1.35	1.08	(1.00, 1.14)
2000-2010	1.53	1.48	(1.42, 1.56)
2010-2020	1.45	1.39	(1.32, 1.44)
1990-2010	2.06	1.62	(1.55, 1.68)
2000-2020	2.22	2.06	(1.89, 2.24)
1990-2020	2.99	2.27	(2.10, 2.39)

Supplementary Figure 46: Supplementary data for the metropolitan zone of San Martín Texmelucan with code 21.1.02. Remoteness values are those of 2020. a) Population difference by grid cell (2020–1990); city centres are denoted as black dots. b) Quantile-quantile plots for the radial population distributions $\rho(s, t_i)$ and $\rho(s, t_j)$ (coloured curves); urban expansion factors Φ_{ij} from t_i to t_j are the estimated slopes (black lines). c) Radial population distribution $\rho(r)$ at remoteness distance r from the city centre. d) Radial population density $\sigma(r)$ at remoteness distance r from the city centre. e) Average population density $\bar{\sigma}(r)$ within disks of remoteness r with the same centre as the city. f) Urban expansion factors and their inter quartile range from the Theil-Sen estimation.

Tehuacán, 21.1.03

a)

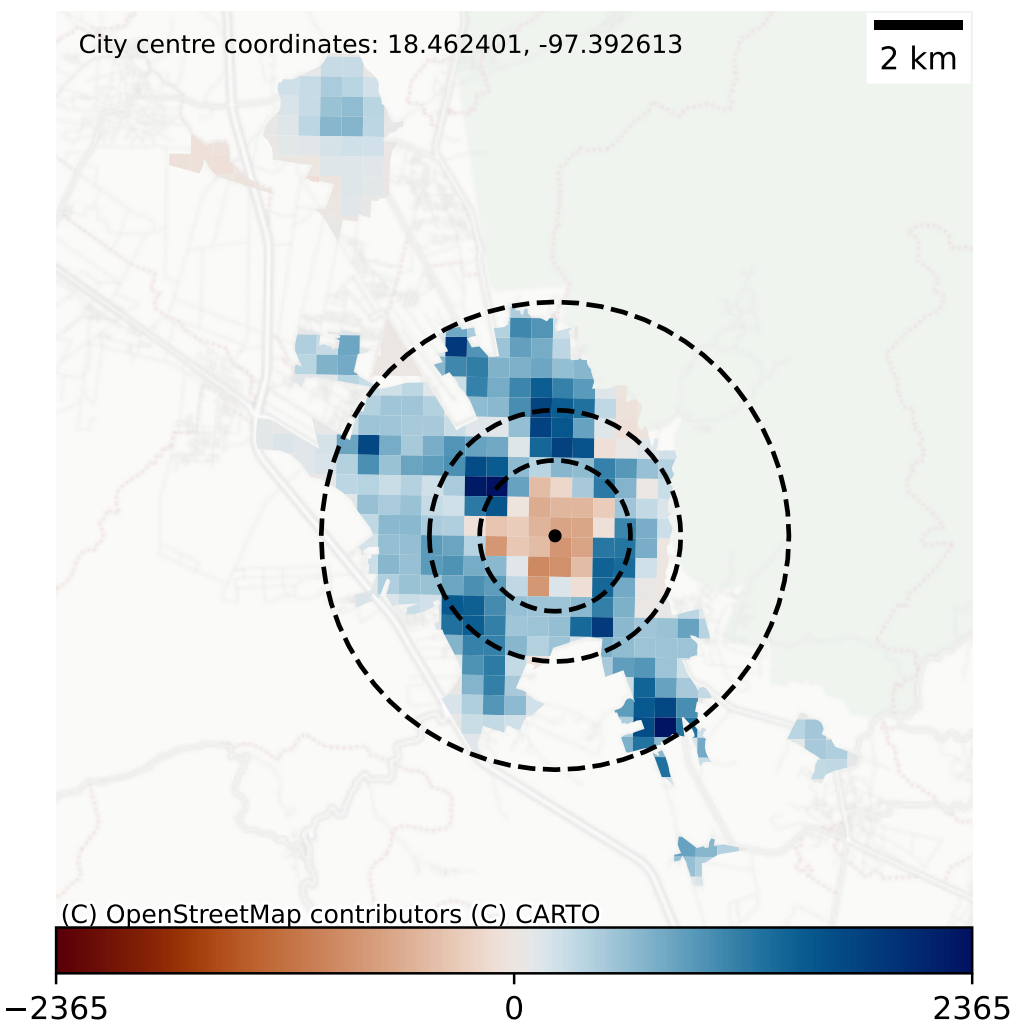

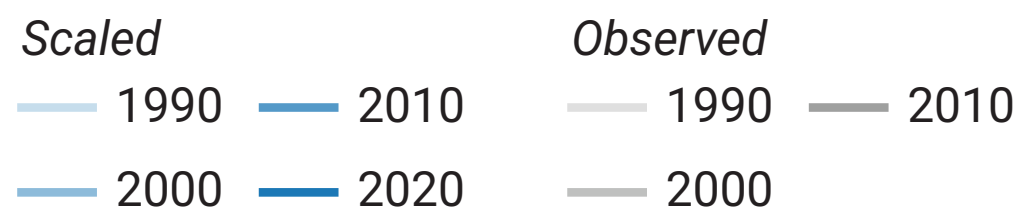

c)

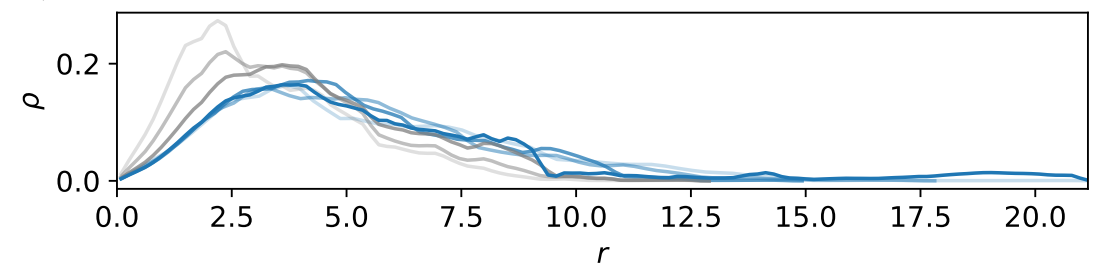

d)

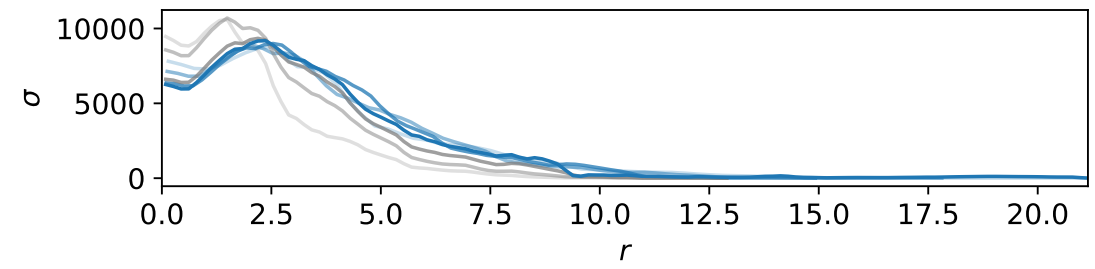

e)

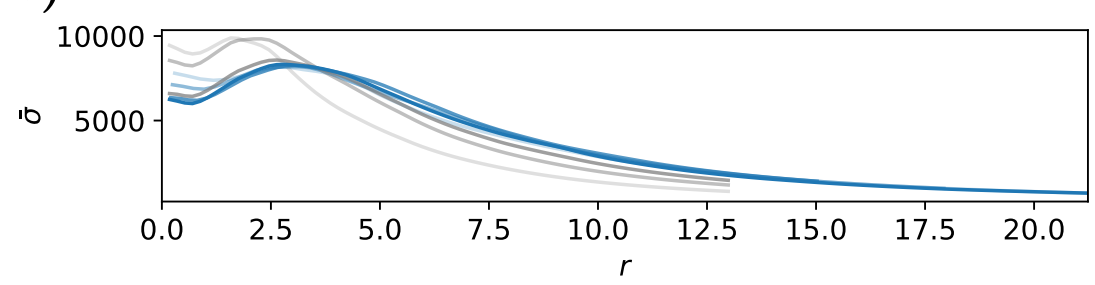

b)

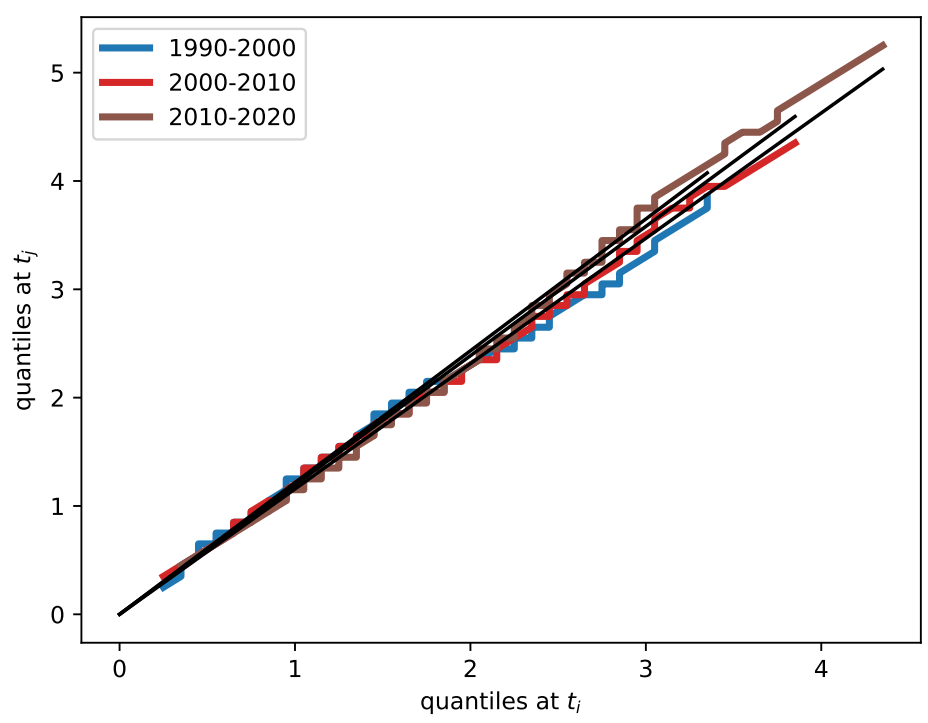

f)

Period (t_i-t_j)	$\frac{P(t_j)}{P(t_i)}$	Φ_{ij}	IQR
1990-2000	1.47	1.22	(1.17, 1.26)
2000-2010	1.23	1.19	(1.16, 1.23)
2010-2020	1.29	1.16	(1.12, 1.18)
1990-2010	1.81	1.45	(1.40, 1.51)
2000-2020	1.59	1.38	(1.34, 1.41)
1990-2020	2.33	1.67	(1.63, 1.73)

Supplementary Figure 47: Supplementary data for the metropolitan zone of Tehuacán with code 21.1.03. Remoteness values are those of 2020. a) Population difference by grid cell (2020–1990); city centres are denoted as black dots. b) Quantile-quantile plots for the radial population distributions $\rho(s, t_i)$ and $\rho(s, t_j)$ (coloured curves); urban expansion factors Φ_{ij} from t_i to t_j are the estimated slopes (black lines). c) Radial population distribution $\rho(r)$ at remoteness distance r from the city centre. d) Radial population density $\sigma(r)$ at remoteness distance r from the city centre. e) Average population density $\bar{\sigma}(r)$ within disks of remoteness r with the same centre as the city. f) Urban expansion factors and their inter quartile range from the Theil-Sen estimation.

Querétaro, 22.1.01

a)

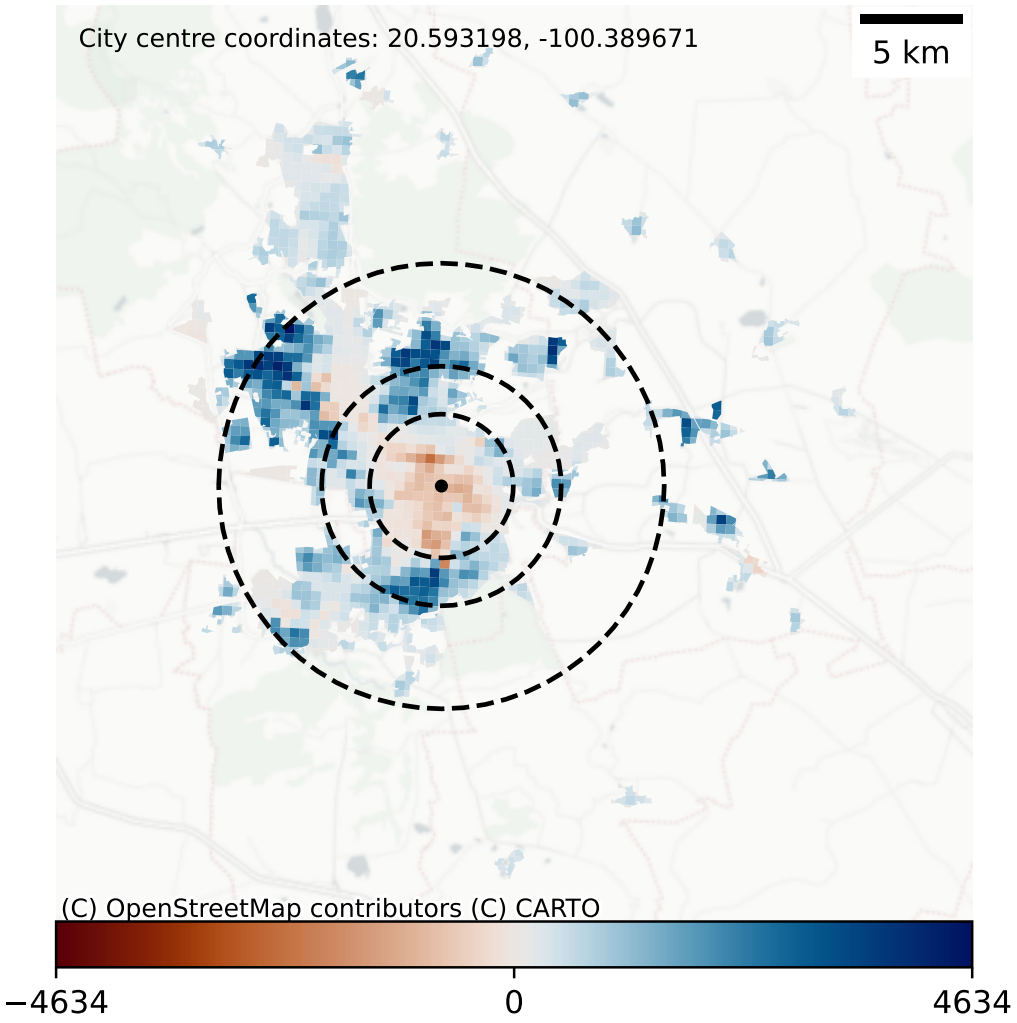

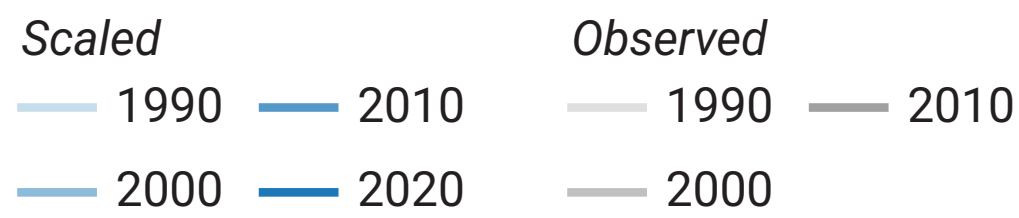

c)

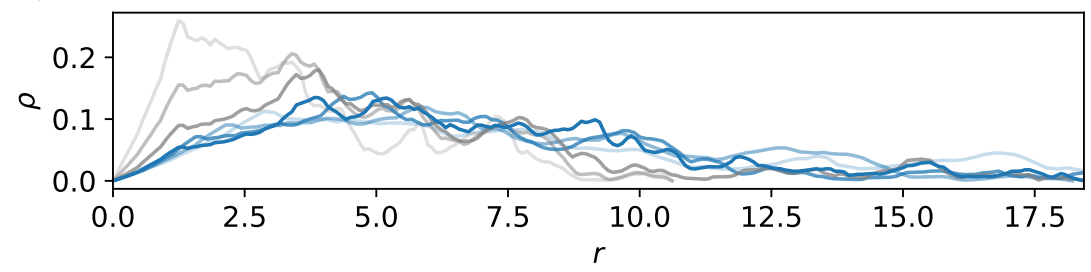

d)

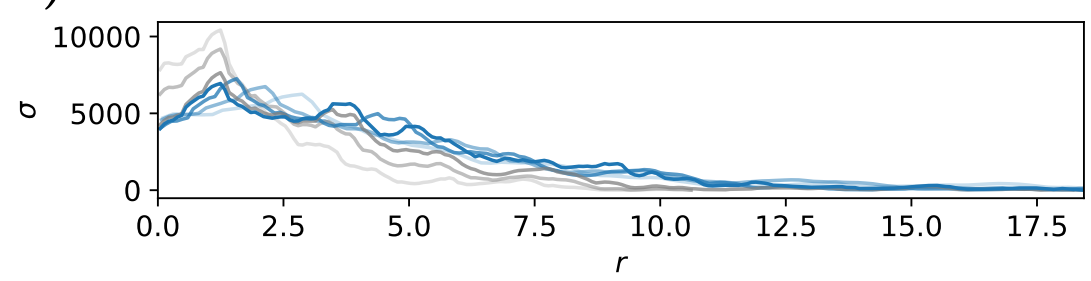

e)

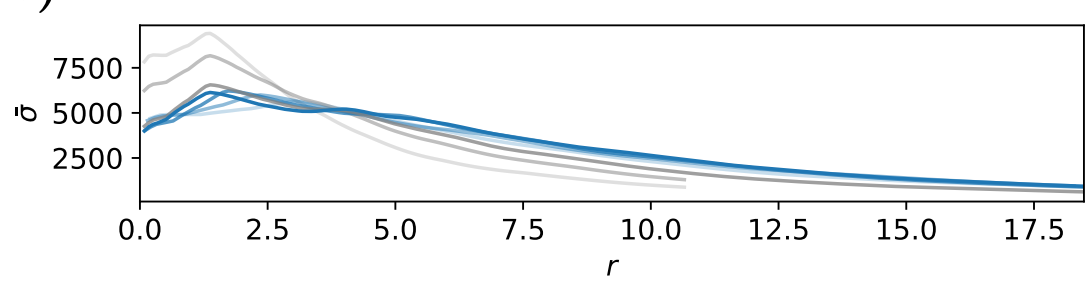

b)

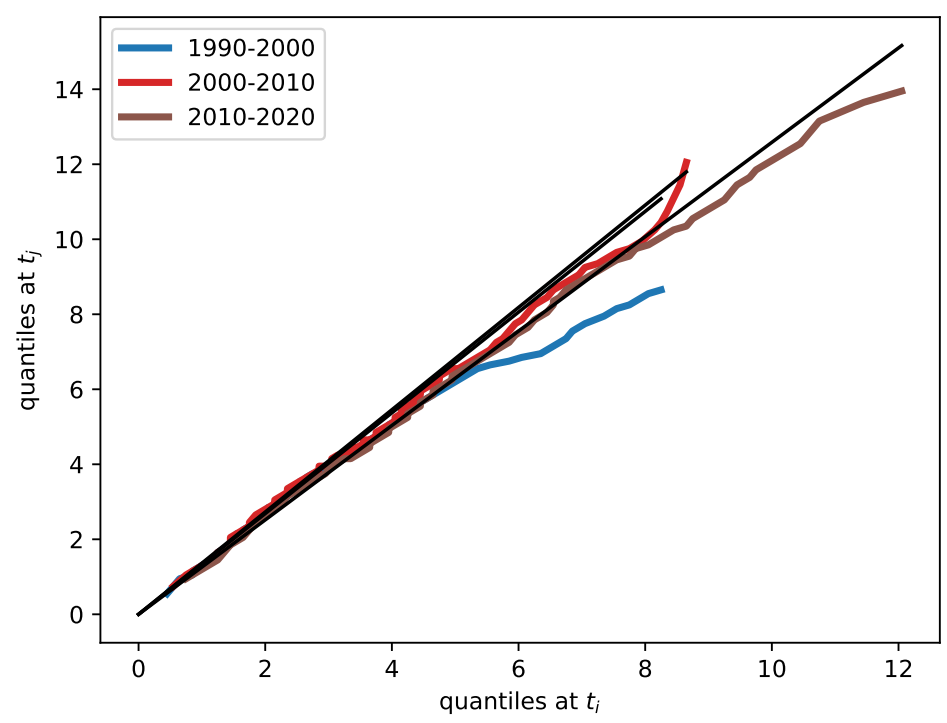

f)

Period (t_i-t_j)	$\frac{P(t_j)}{P(t_i)}$	Φ_{ij}	IQR
1990-2000	1.47	1.34	(1.31, 1.36)
2000-2010	1.44	1.36	(1.32, 1.39)
2010-2020	1.50	1.26	(1.24, 1.29)
1990-2010	2.12	1.82	(1.76, 1.87)
2000-2020	2.16	1.69	(1.66, 1.78)
1990-2020	3.18	2.28	(2.23, 2.35)

Supplementary Figure 48: Supplementary data for the metropolitan zone of Querétaro with code 22.1.01. Remoteness values are those of 2020. a) Population difference by grid cell (2020–1990); city centres are denoted as black dots. b) Quantile-quantile plots for the radial population distributions $\rho(s, t_i)$ and $\rho(s, t_j)$ (coloured curves); urban expansion factors Φ_{ij} from t_i to t_j are the estimated slopes (black lines). c) Radial population distribution $\rho(r)$ at remoteness distance r from the city centre. d) Radial population density $\sigma(r)$ at remoteness distance r from the city centre. e) Average population density $\bar{\sigma}(r)$ within disks of remoteness r with the same centre as the city. f) Urban expansion factors and their inter quartile range from the Theil-Sen estimation.

Cancún, 23.1.01

a)

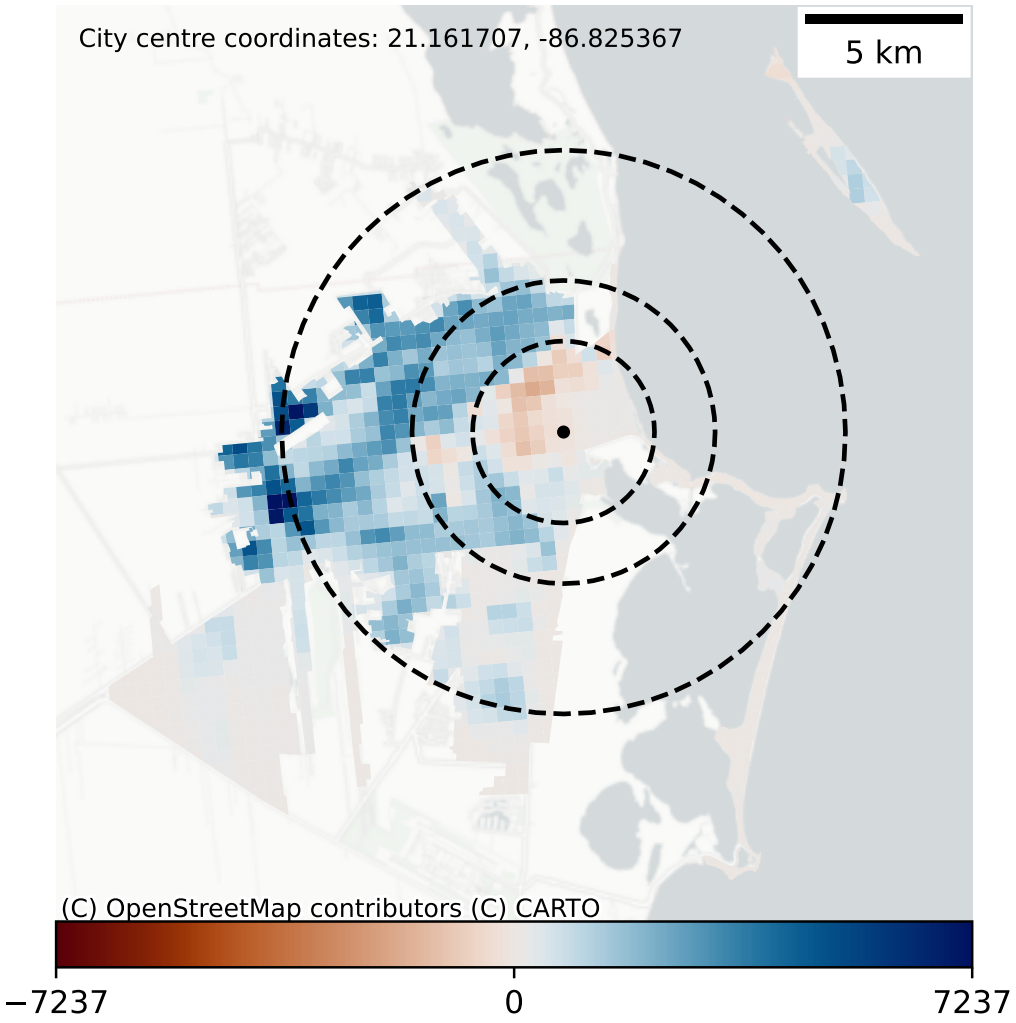

b)

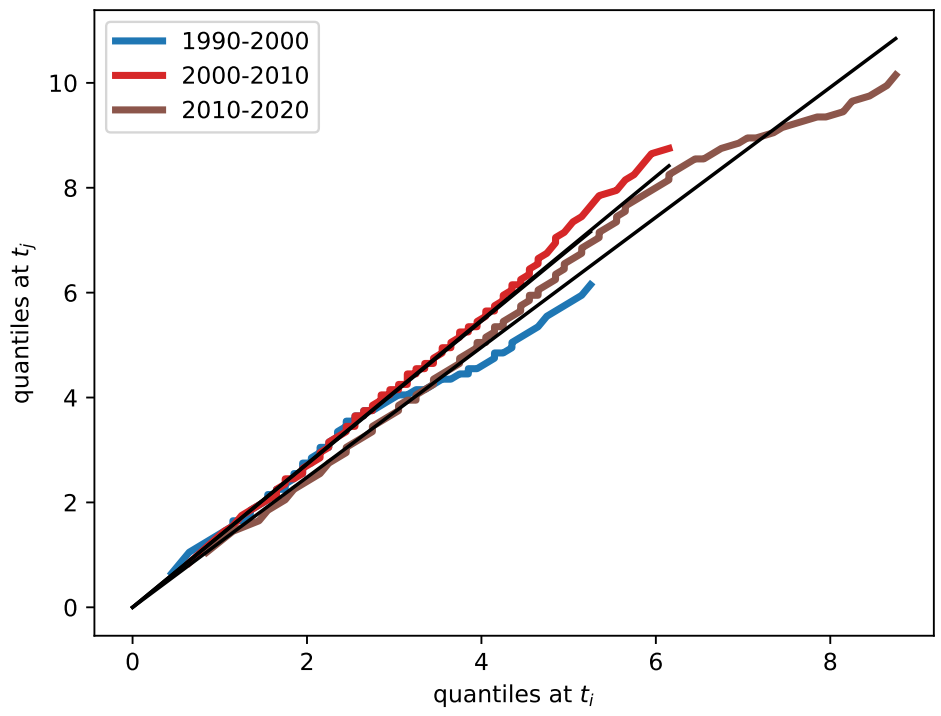

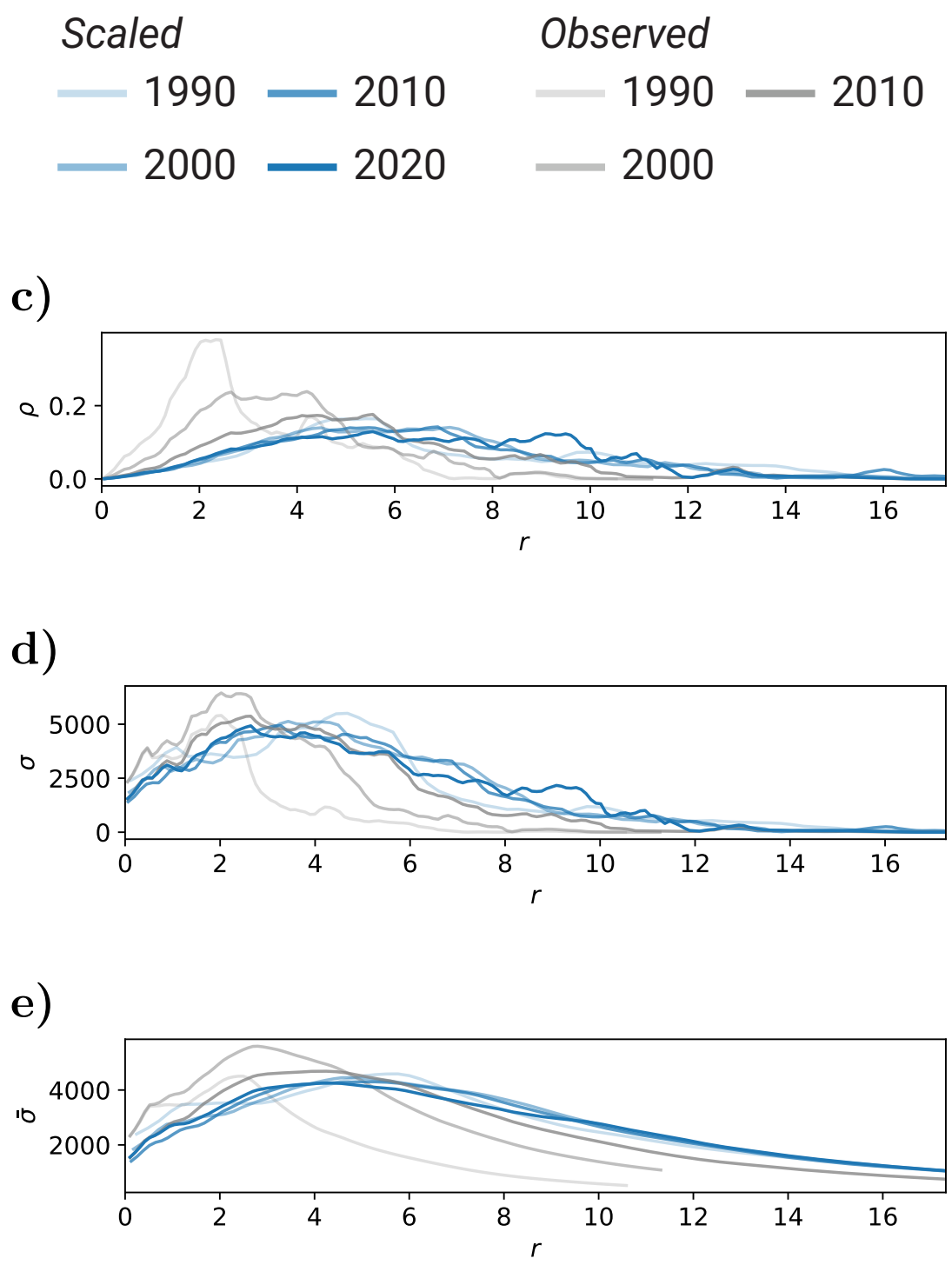

f)

Period (t_i-t_j)	$\frac{P(t_j)}{P(t_i)}$	Φ_{ij}	IQR
1990-2000	2.38	1.36	(1.33, 1.39)
2000-2010	1.63	1.37	(1.35, 1.39)
2010-2020	1.41	1.24	(1.22, 1.25)
1990-2010	3.86	1.86	(1.81, 1.92)
2000-2020	2.29	1.69	(1.64, 1.74)
1990-2020	5.45	2.32	(2.24, 2.40)

Supplementary Figure 49: Supplementary data for the metropolitan zone of Cancún with code 23.1.01. Remoteness values are those of 2020. a) Population difference by grid cell (2020–1990); city centres are denoted as black dots. b) Quantile-quantile plots for the radial population distributions $\rho(s, t_i)$ and $\rho(s, t_j)$ (coloured curves); urban expansion factors Φ_{ij} from t_i to t_j are the estimated slopes (black lines). c) Radial population distribution $\rho(r)$ at remoteness distance r from the city centre. d) Radial population density $\sigma(r)$ at remoteness distance r from the city centre. e) Average population density $\bar{\sigma}(r)$ within disks of remoteness r with the same centre as the city. f) Urban expansion factors and their inter quartile range from the Theil-Sen estimation.

Chetumal, 23.2.02

a)

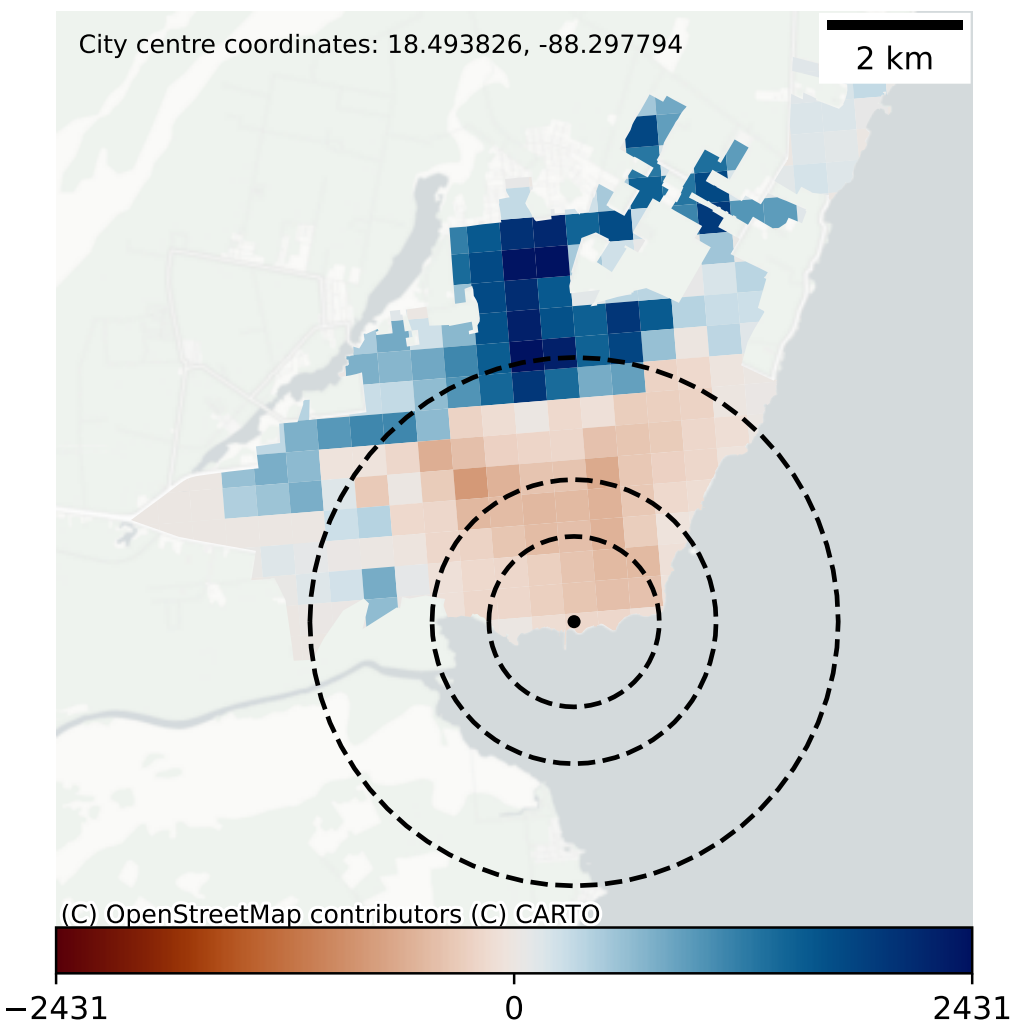

b)

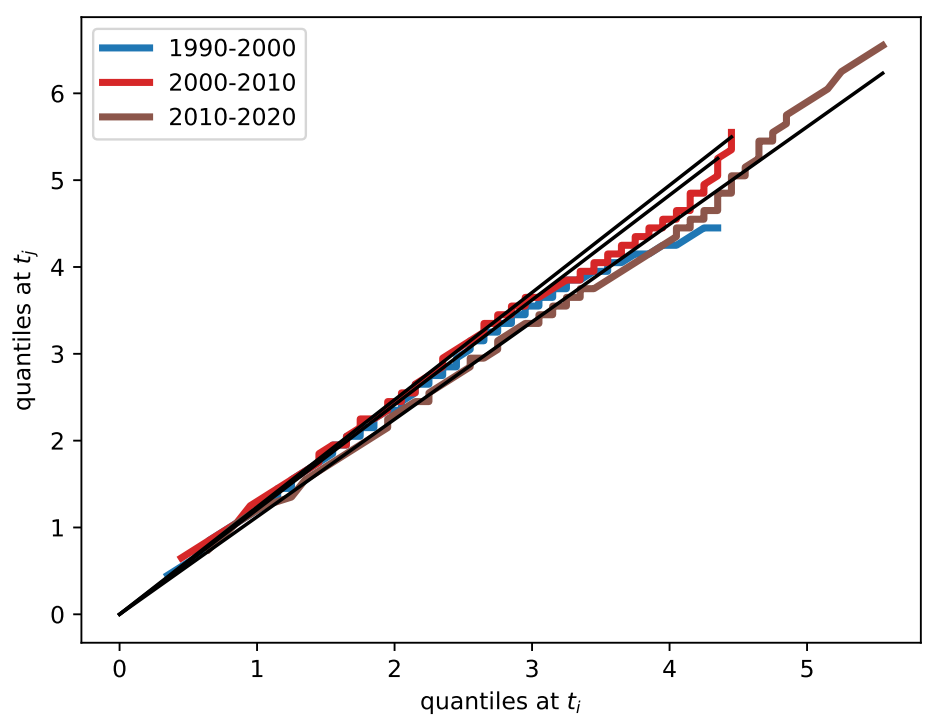

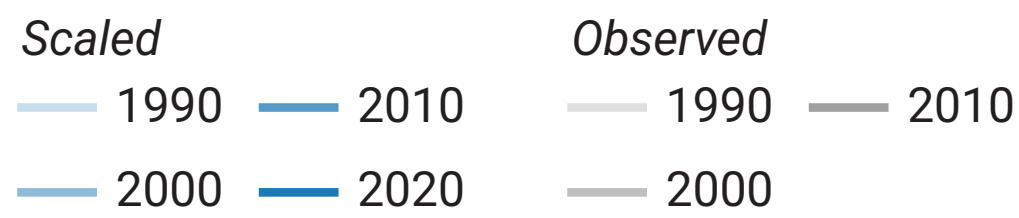

c)

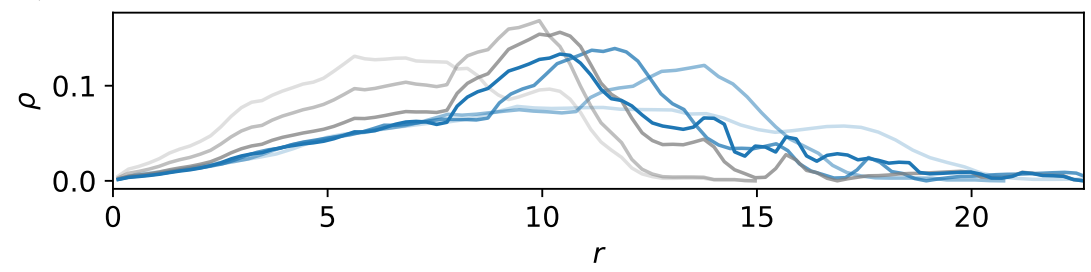

d)

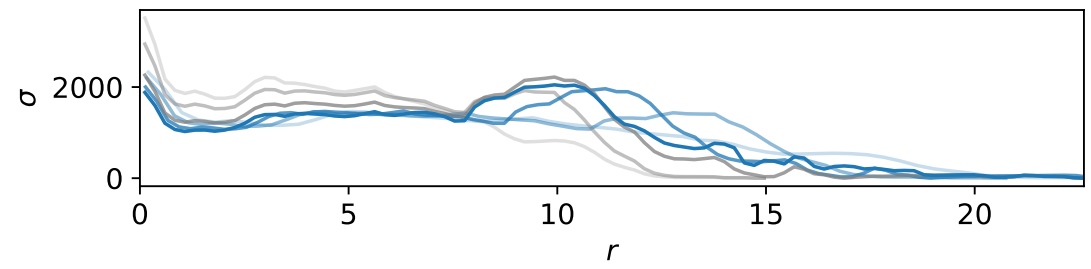

e)

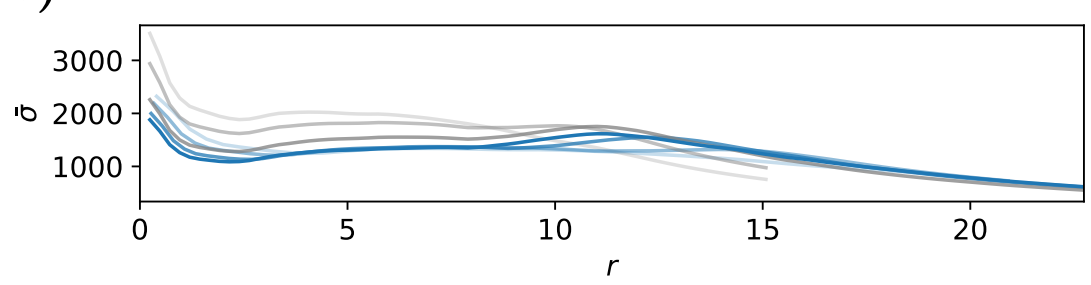

f)

Period (t_i-t_j)	$\frac{P(t_j)}{P(t_i)}$	Φ_{ij}	IQR
1990-2000	1.29	1.21	(1.18, 1.23)
2000-2010	1.29	1.24	(1.22, 1.26)
2010-2020	1.12	1.12	(1.10, 1.14)
1990-2010	1.66	1.49	(1.46, 1.52)
2000-2020	1.44	1.38	(1.36, 1.41)
1990-2020	1.85	1.67	(1.64, 1.70)

Supplementary Figure 50: Supplementary data for the metropolitan zone of Chetumal with code 23.2.02. Remoteness values are those of 2020. a) Population difference by grid cell (2020–1990); city centres are denoted as black dots. b) Quantile-quantile plots for the radial population distributions $\rho(s, t_i)$ and $\rho(s, t_j)$ (coloured curves); urban expansion factors Φ_{ij} from t_i to t_j are the estimated slopes (black lines). c) Radial population distribution $\rho(r)$ at remoteness distance r from the city centre. d) Radial population density $\sigma(r)$ at remoteness distance r from the city centre. e) Average population density $\bar{\sigma}(r)$ within disks of remoteness r with the same centre as the city. f) Urban expansion factors and their inter quartile range from the Theil-Sen estimation.

San Luis Potosí, 24.1.01

a)

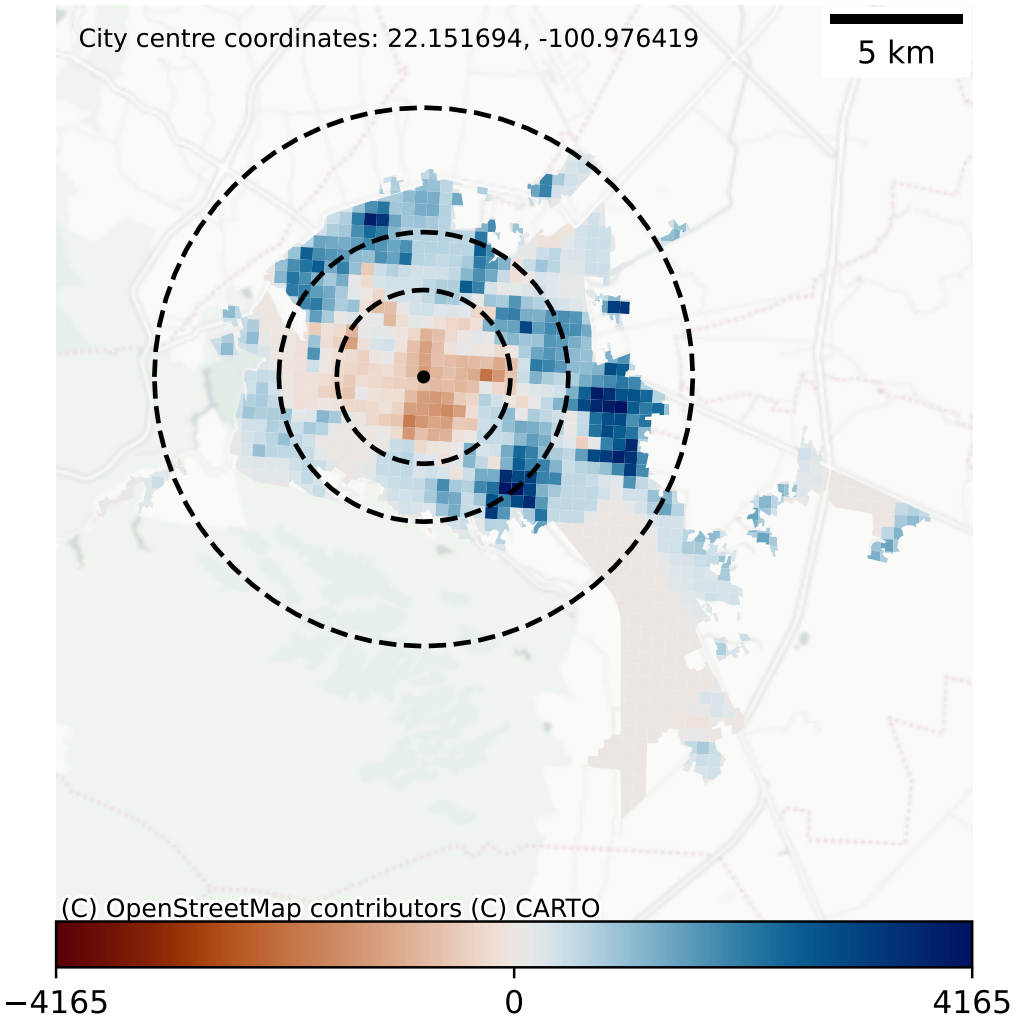

b)

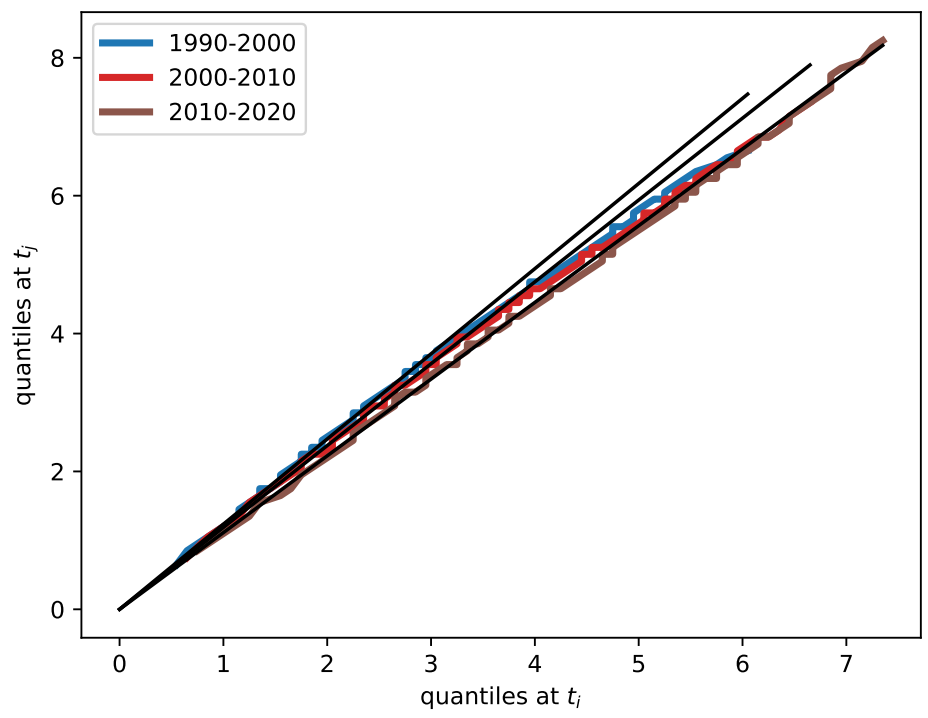

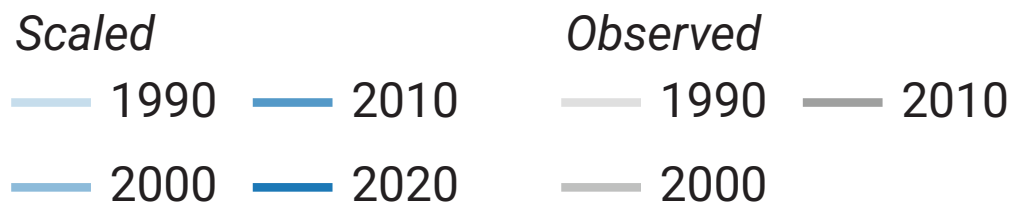

c)

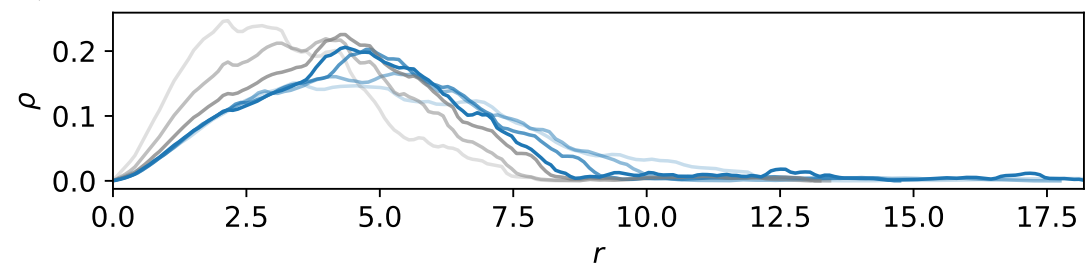

d)

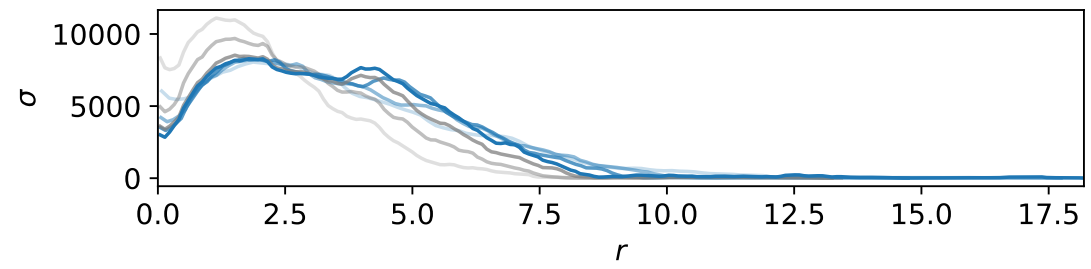

e)

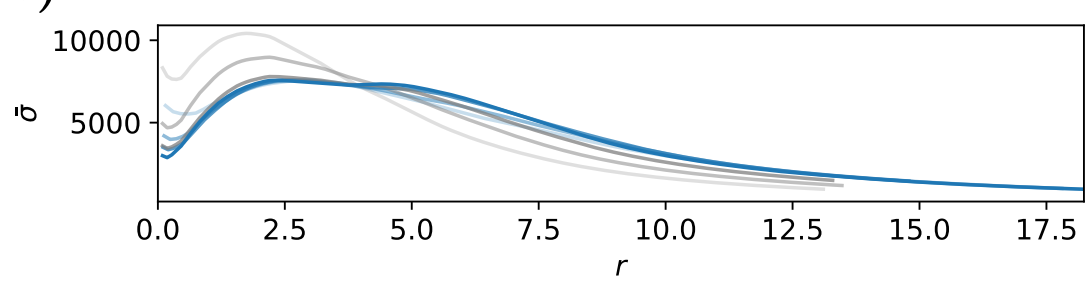

f)

Period (t_i-t_j)	$\frac{P(t_j)}{P(t_i)}$	Φ_{ij}	IQR
1990-2000	1.30	1.24	(1.22, 1.26)
2000-2010	1.23	1.19	(1.17, 1.20)
2010-2020	1.21	1.11	(1.10, 1.13)
1990-2010	1.60	1.47	(1.45, 1.49)
2000-2020	1.48	1.33	(1.31, 1.34)
1990-2020	1.93	1.64	(1.62, 1.67)

Supplementary Figure 51: Supplementary data for the metropolitan zone of San Luis Potosí with code 24.1.01. Remoteness values are those of 2020. a) Population difference by grid cell (2020–1990); city centres are denoted as black dots. b) Quantile-quantile plots for the radial population distributions $\rho(s, t_i)$ and $\rho(s, t_j)$ (coloured curves); urban expansion factors Φ_{ij} from t_i to t_j are the estimated slopes (black lines). c) Radial population distribution $\rho(r)$ at remoteness distance r from the city centre. d) Radial population density $\sigma(r)$ at remoteness distance r from the city centre. e) Average population density $\bar{\sigma}(r)$ within disks of remoteness r with the same centre as the city. f) Urban expansion factors and their inter quartile range from the Theil-Sen estimation.

Culiacán, 25.2.01

a)

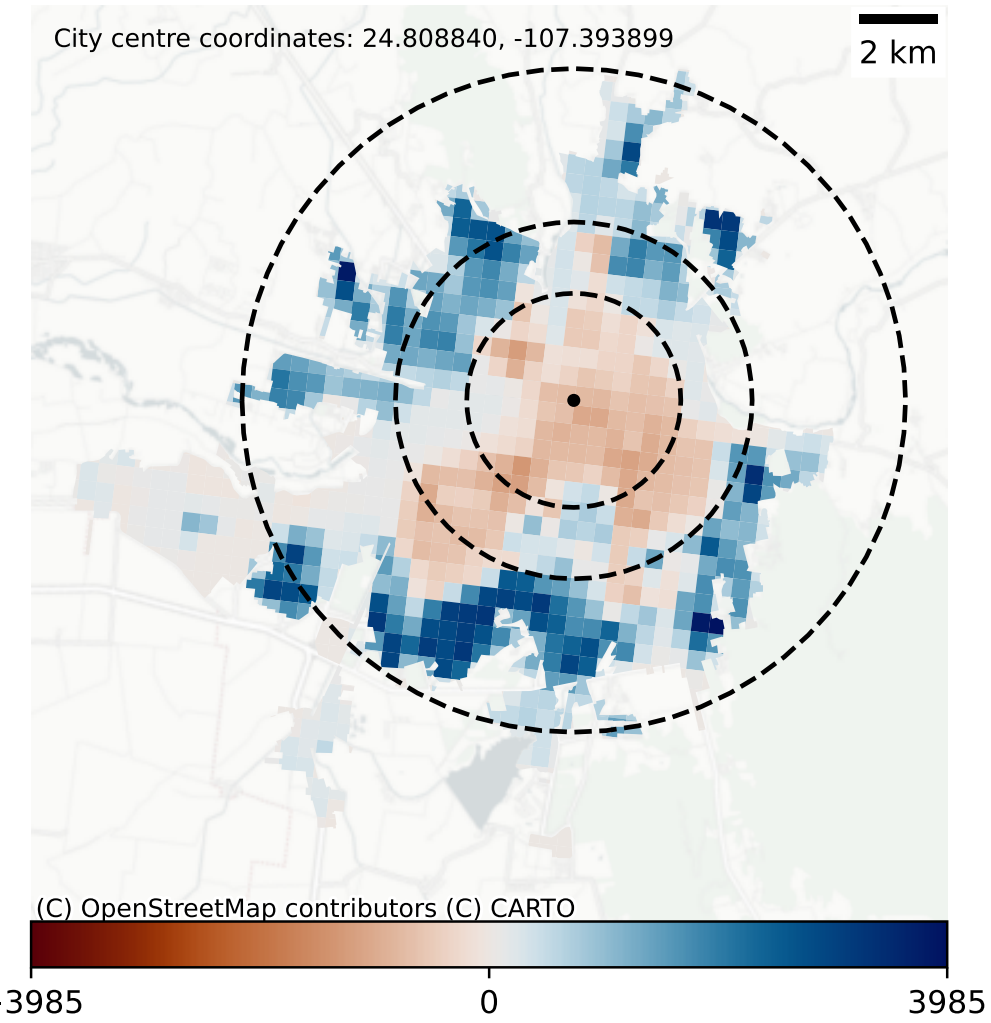

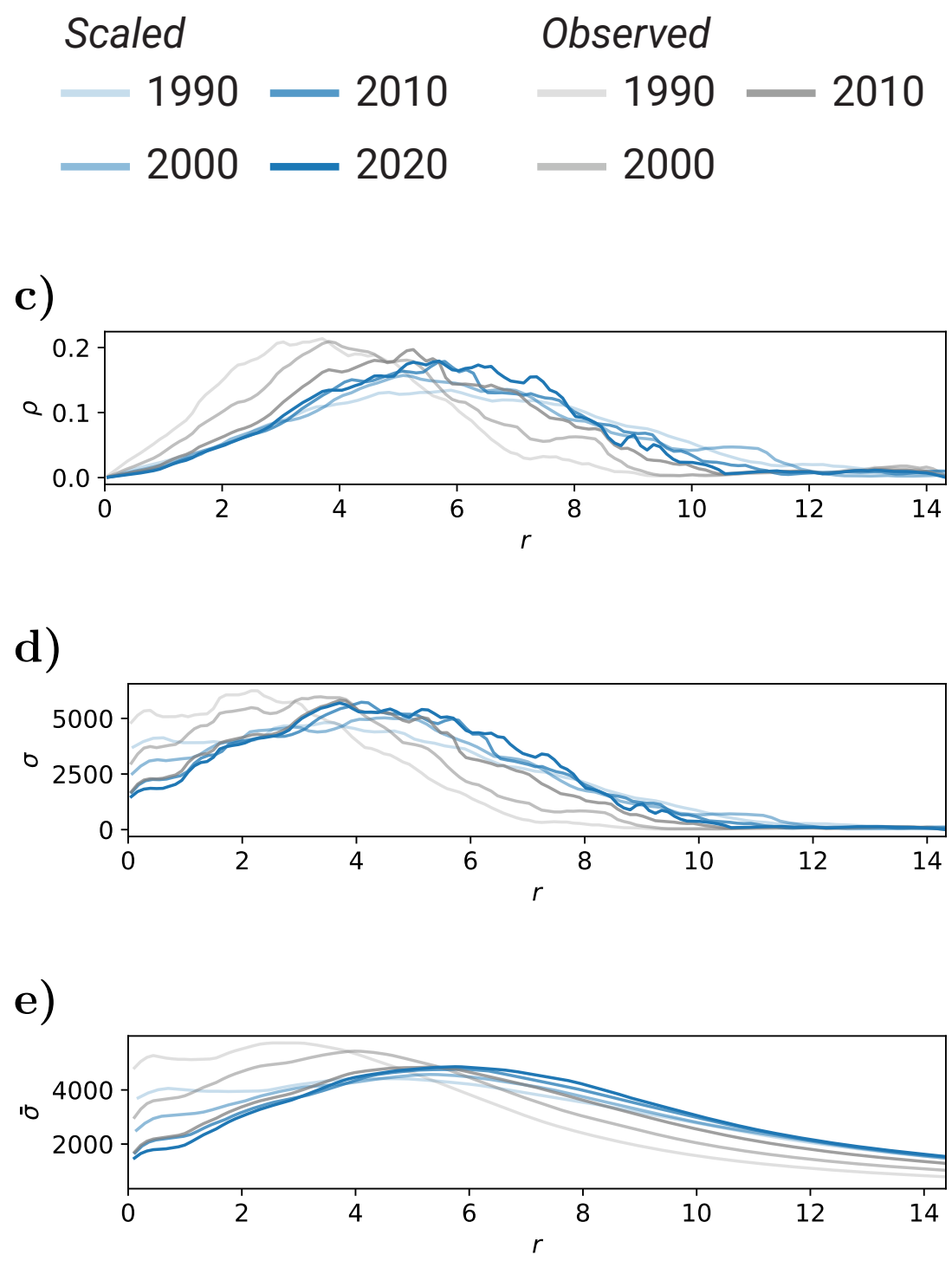

b)

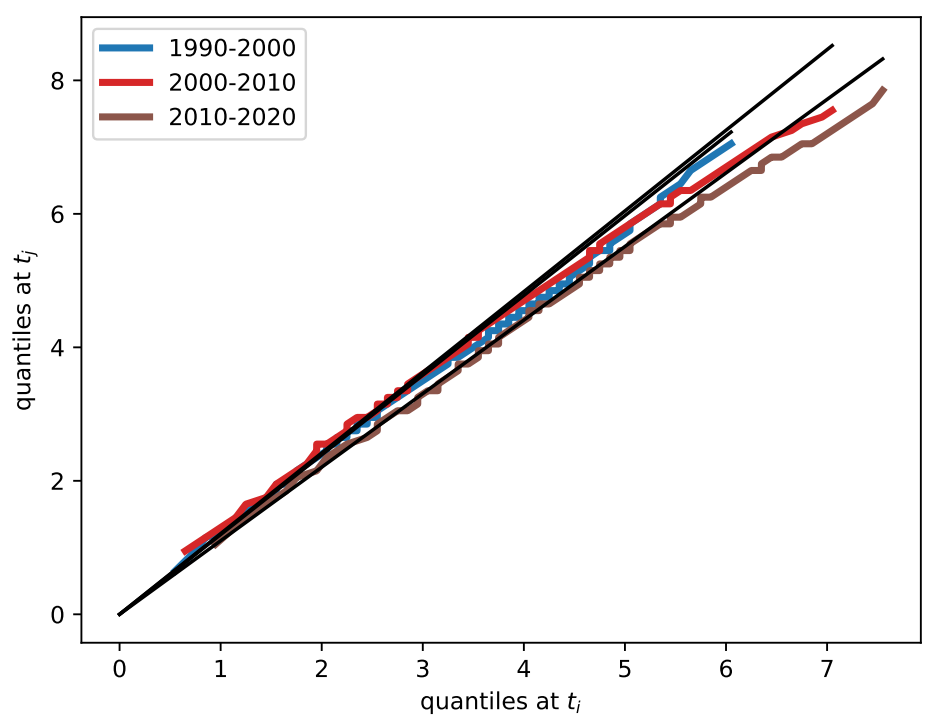

f)

Period (t_i-t_j)	$\frac{P(t_j)}{P(t_i)}$	Φ_{ij}	IQR
1990-2000	1.31	1.19	(1.18, 1.22)
2000-2010	1.25	1.21	(1.19, 1.24)
2010-2020	1.20	1.10	(1.09, 1.12)
1990-2010	1.63	1.45	(1.42, 1.52)
2000-2020	1.49	1.33	(1.31, 1.38)
1990-2020	1.95	1.59	(1.55, 1.68)

Supplementary Figure 52: Supplementary data for the metropolitan zone of Culiacán with code 25.2.01. Remoteness values are those of 2020. a) Population difference by grid cell (2020–1990); city centres are denoted as black dots. b) Quantile-quantile plots for the radial population distributions $\rho(s, t_i)$ and $\rho(s, t_j)$ (coloured curves); urban expansion factors Φ_{ij} from t_i to t_j are the estimated slopes (black lines). c) Radial population distribution $\rho(r)$ at remoteness distance r from the city centre. d) Radial population density $\sigma(r)$ at remoteness distance r from the city centre. e) Average population density $\bar{\sigma}(r)$ within disks of remoteness r with the same centre as the city. f) Urban expansion factors and their inter quartile range from the Theil-Sen estimation.

Los Mochis, 25.2.02

a)

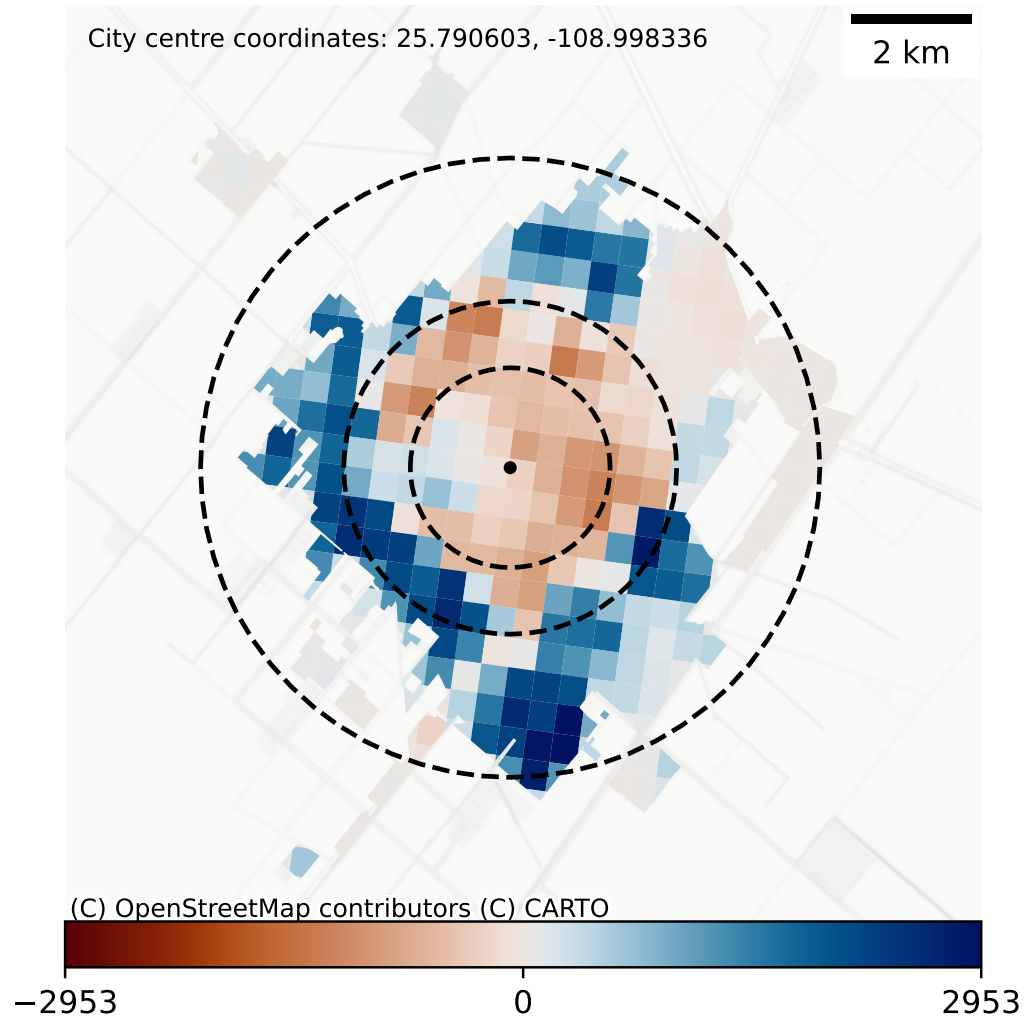

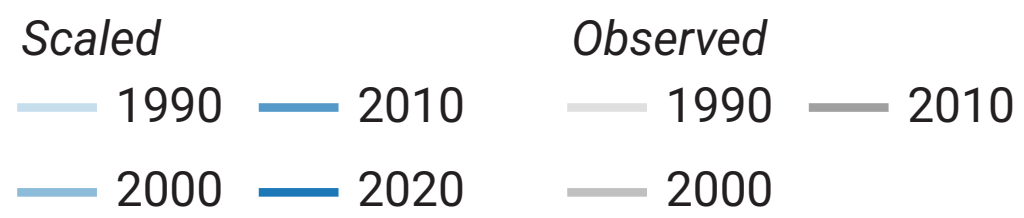

c)

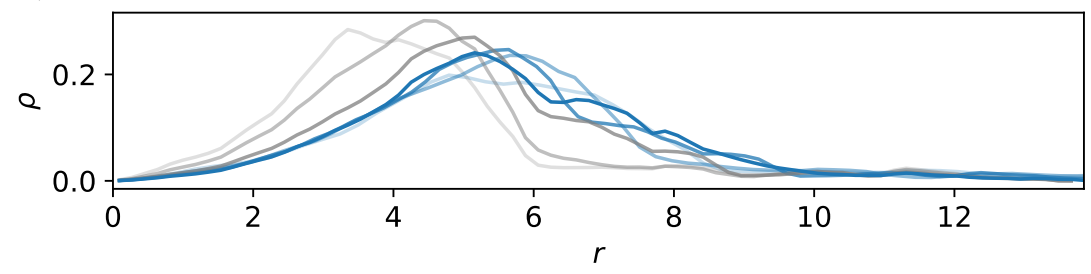

d)

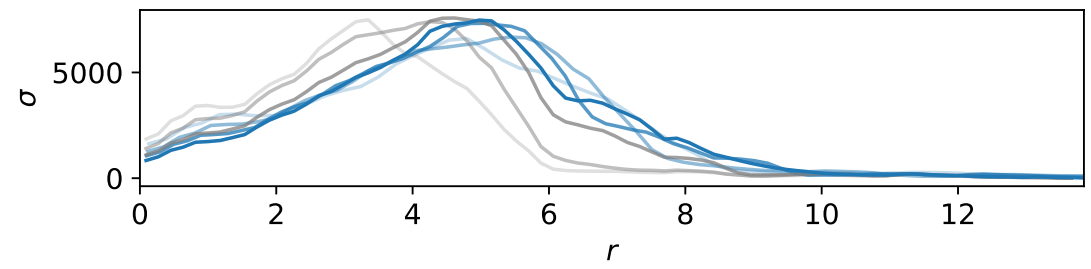

e)

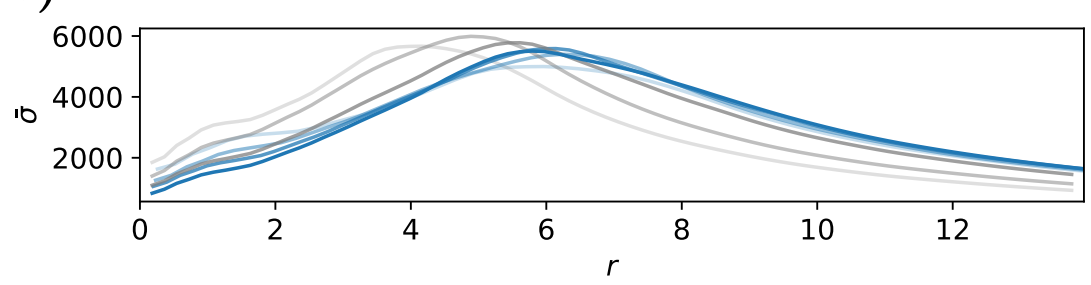

b)

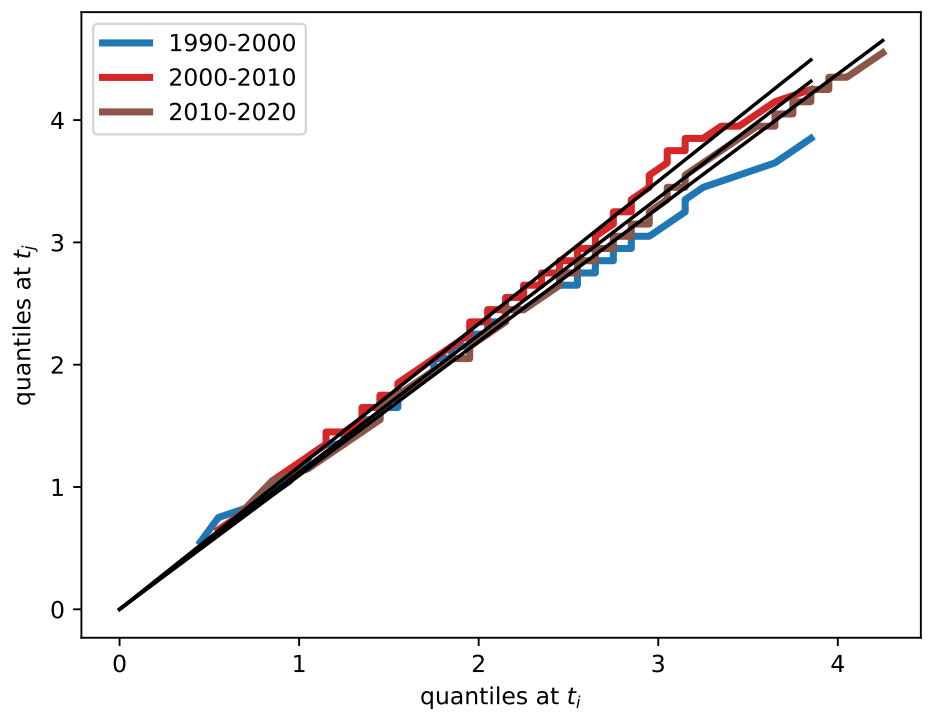

f)

Period (t_i-t_j)	$\frac{P(t_j)}{P(t_i)}$	Φ_{ij}	IQR
1990-2000	1.23	1.12	(1.11, 1.15)
2000-2010	1.27	1.17	(1.15, 1.19)
2010-2020	1.16	1.09	(1.09, 1.11)
1990-2010	1.56	1.32	(1.30, 1.35)
2000-2020	1.47	1.29	(1.26, 1.32)
1990-2020	1.81	1.45	(1.42, 1.48)

Supplementary Figure 53: Supplementary data for the metropolitan zone of Los Mochis with code 25.2.02. Remoteness values are those of 2020. a) Population difference by grid cell (2020–1990); city centres are denoted as black dots. b) Quantile-quantile plots for the radial population distributions $\rho(s, t_i)$ and $\rho(s, t_j)$ (coloured curves); urban expansion factors Φ_{ij} from t_i to t_j are the estimated slopes (black lines). c) Radial population distribution $\rho(r)$ at remoteness distance r from the city centre. d) Radial population density $\sigma(r)$ at remoteness distance r from the city centre. e) Average population density $\bar{\sigma}(r)$ within disks of remoteness r with the same centre as the city. f) Urban expansion factors and their inter quartile range from the Theil-Sen estimation.

Mazatlán, 25.2.03

a)

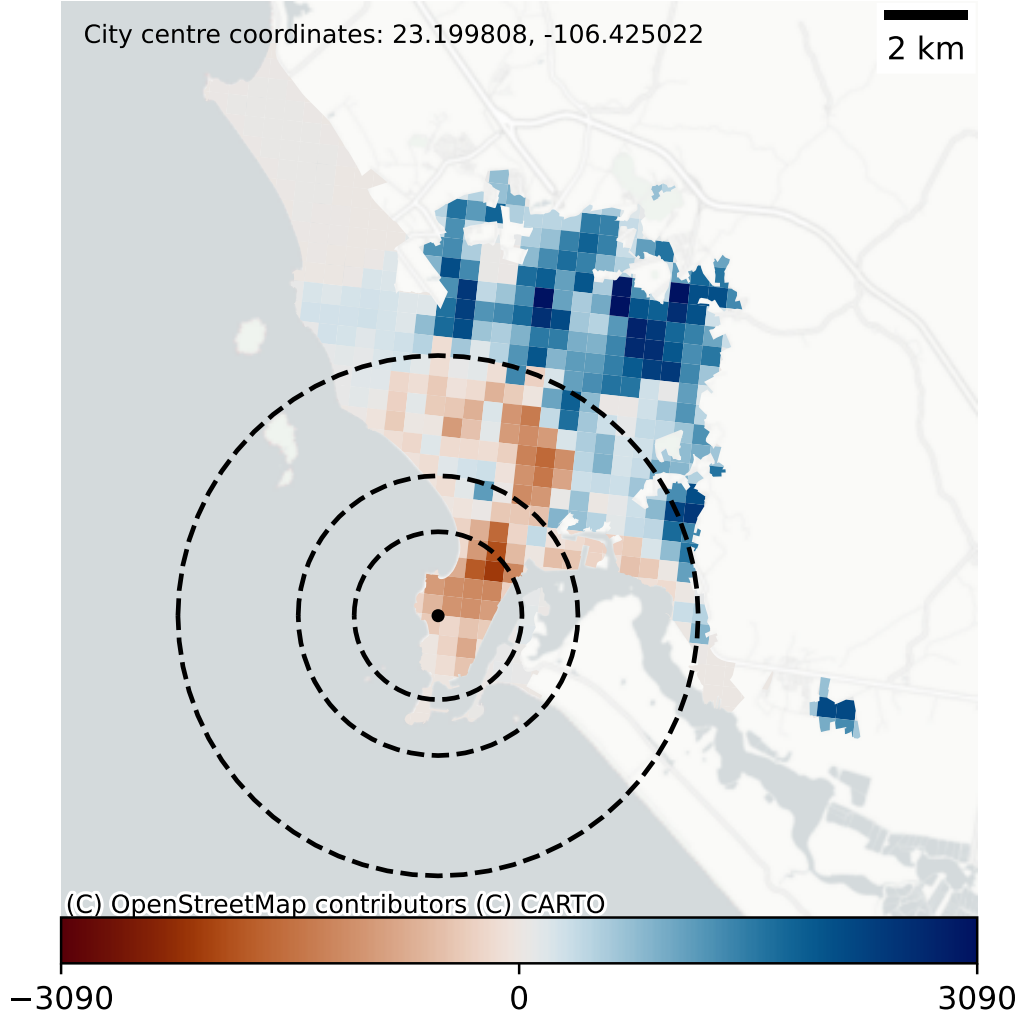

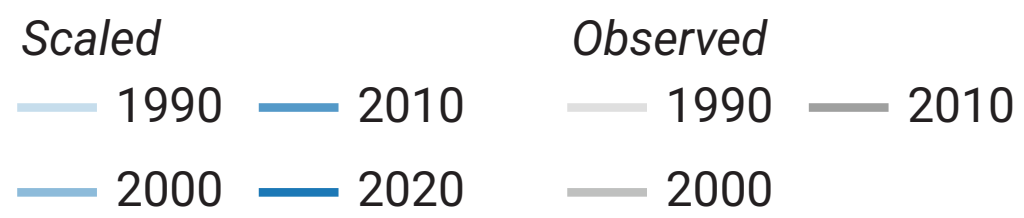

c)

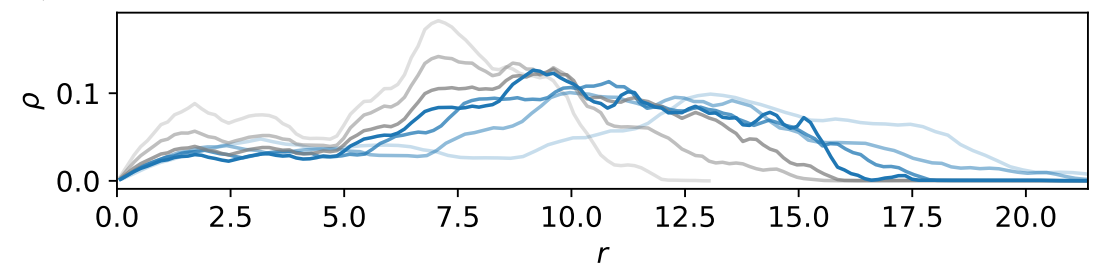

d)

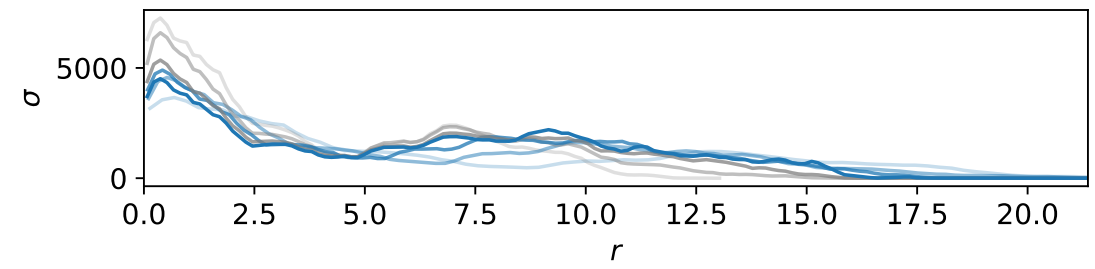

e)

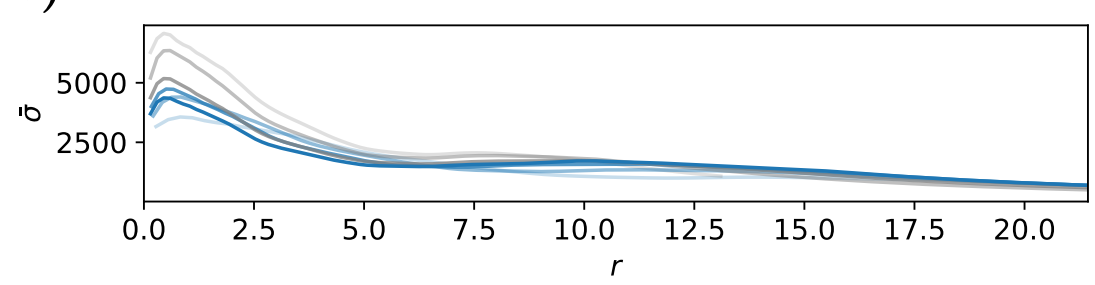

b)

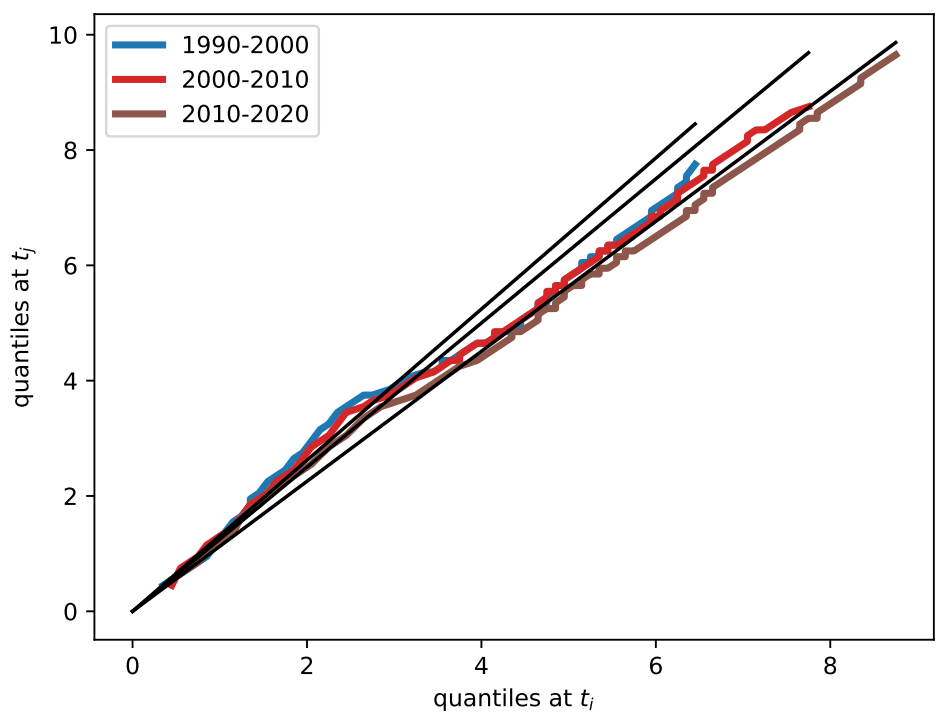

f)

Period (t_i-t_j)	$\frac{P(t_j)}{P(t_i)}$	Φ_{ij}	IQR
1990-2000	1.25	1.31	(1.20, 1.42)
2000-2010	1.18	1.25	(1.15, 1.34)
2010-2020	1.16	1.13	(1.10, 1.22)
1990-2010	1.48	1.65	(1.43, 1.81)
2000-2020	1.38	1.38	(1.27, 1.61)
1990-2020	1.72	1.89	(1.59, 2.21)

Supplementary Figure 54: Supplementary data for the metropolitan zone of Mazatlán with code 25.2.03. Remoteness values are those of 2020. a) Population difference by grid cell (2020–1990); city centres are denoted as black dots. b) Quantile-quantile plots for the radial population distributions $\rho(s, t_i)$ and $\rho(s, t_j)$ (coloured curves); urban expansion factors Φ_{ij} from t_i to t_j are the estimated slopes (black lines). c) Radial population distribution $\rho(r)$ at remoteness distance r from the city centre. d) Radial population density $\sigma(r)$ at remoteness distance r from the city centre. e) Average population density $\bar{\sigma}(r)$ within disks of remoteness r with the same centre as the city. f) Urban expansion factors and their inter quartile range from the Theil-Sen estimation.

Guaymas, 26.1.01

a)

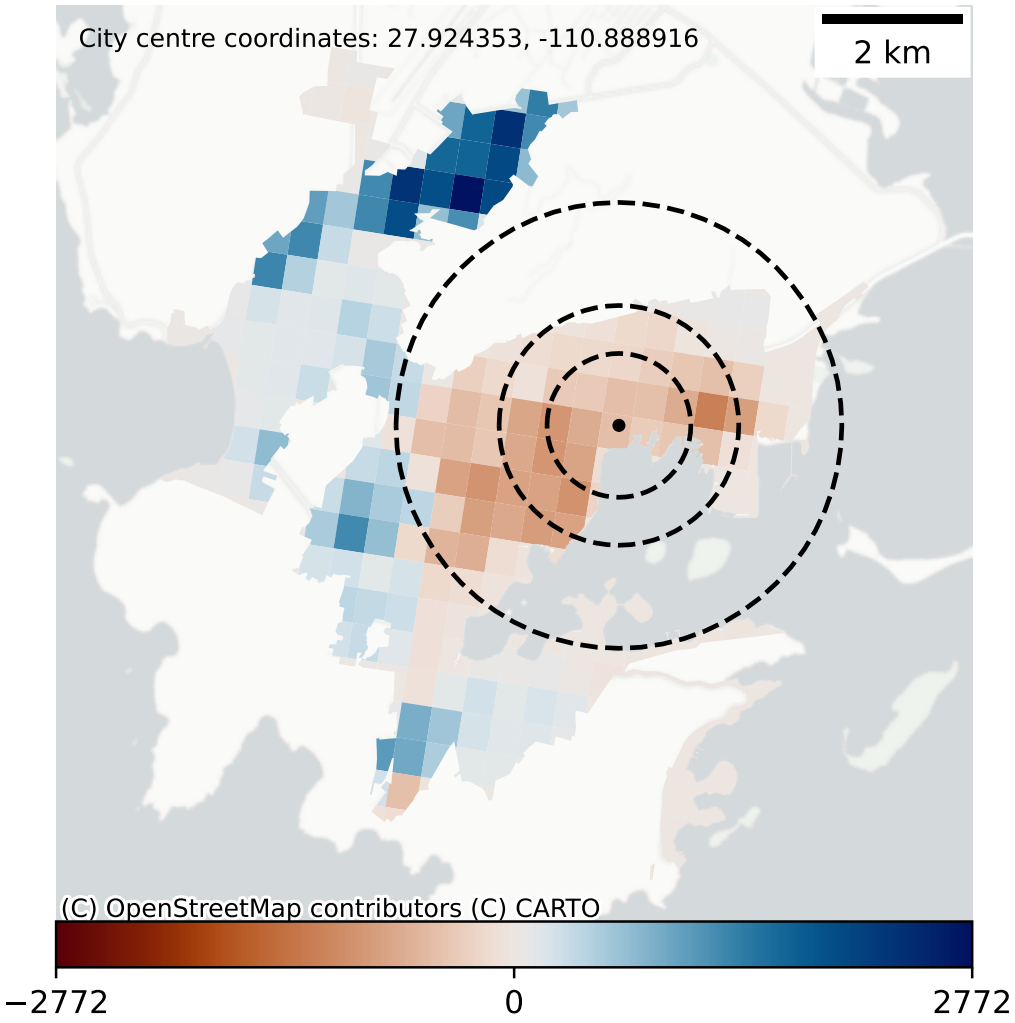

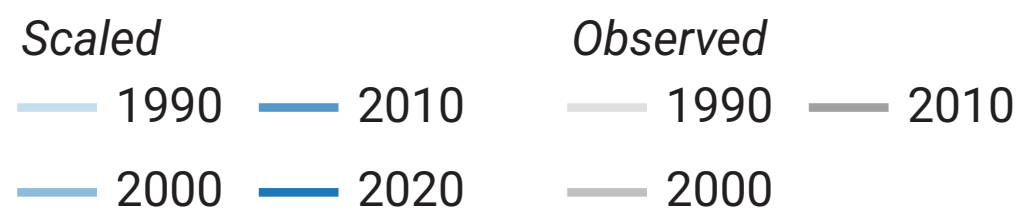

c)

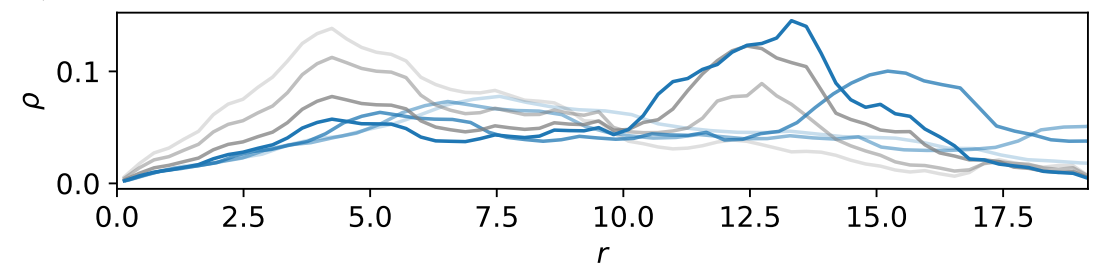

d)

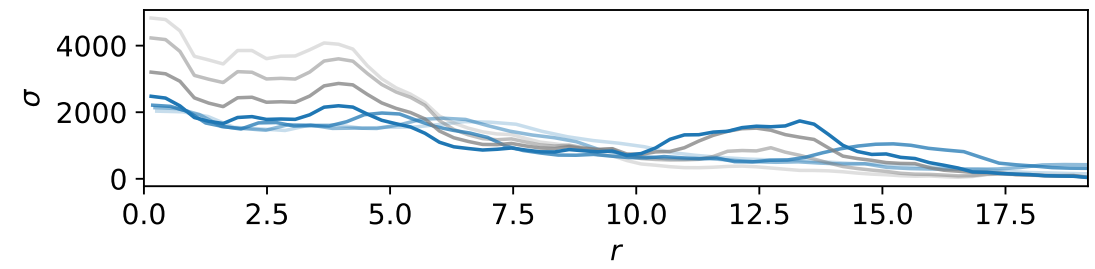

e)

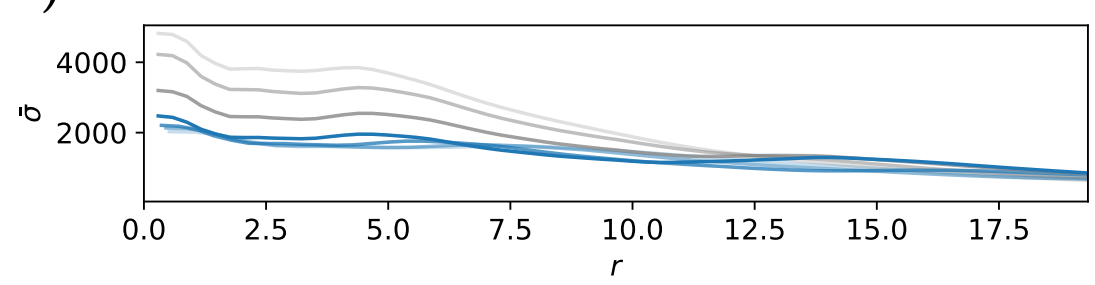

b)

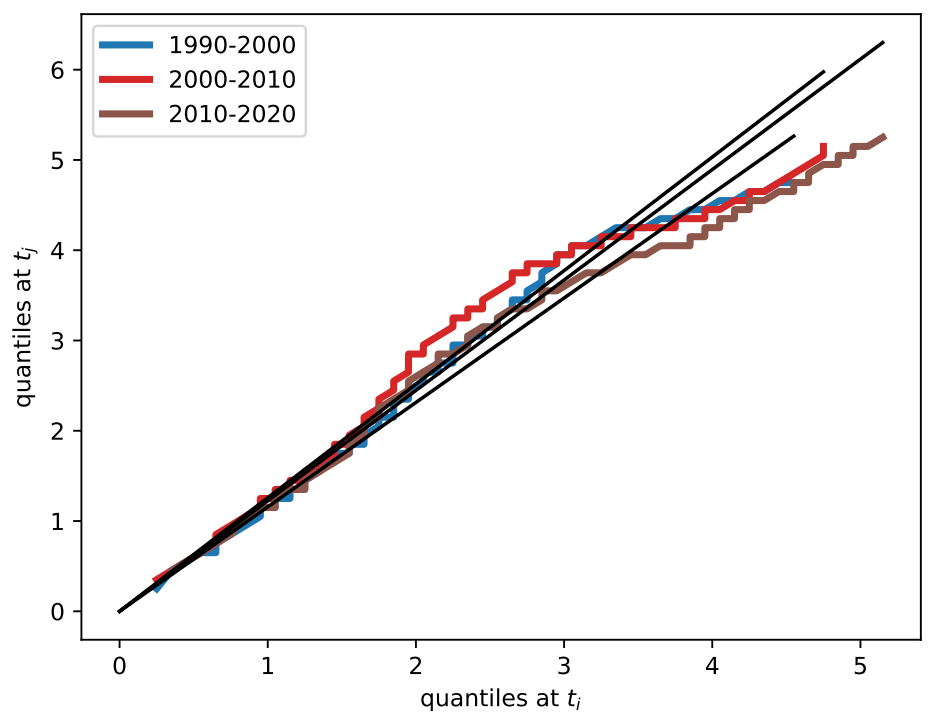

f)

Period (t_i-t_j)	$\frac{P(t_j)}{P(t_i)}$	Φ_{ij}	IQR
1990-2000	1.12	1.16	(1.12, 1.19)
2000-2010	1.16	1.26	(1.21, 1.31)
2010-2020	1.03	1.22	(1.16, 1.27)
1990-2010	1.29	1.44	(1.39, 1.52)
2000-2020	1.20	1.53	(1.42, 1.68)
1990-2020	1.33	1.75	(1.62, 1.96)

Supplementary Figure 55: Supplementary data for the metropolitan zone of Guaymas with code 26.1.01. Remoteness values are those of 2020. a) Population difference by grid cell (2020–1990); city centres are denoted as black dots. b) Quantile-quantile plots for the radial population distributions $\rho(s, t_i)$ and $\rho(s, t_j)$ (coloured curves); urban expansion factors Φ_{ij} from t_i to t_j are the estimated slopes (black lines). c) Radial population distribution $\rho(r)$ at remoteness distance r from the city centre. d) Radial population density $\sigma(r)$ at remoteness distance r from the city centre. e) Average population density $\bar{\sigma}(r)$ within disks of remoteness r with the same centre as the city. f) Urban expansion factors and their inter quartile range from the Theil-Sen estimation.

Ciudad Obregón, 26.2.02

a)

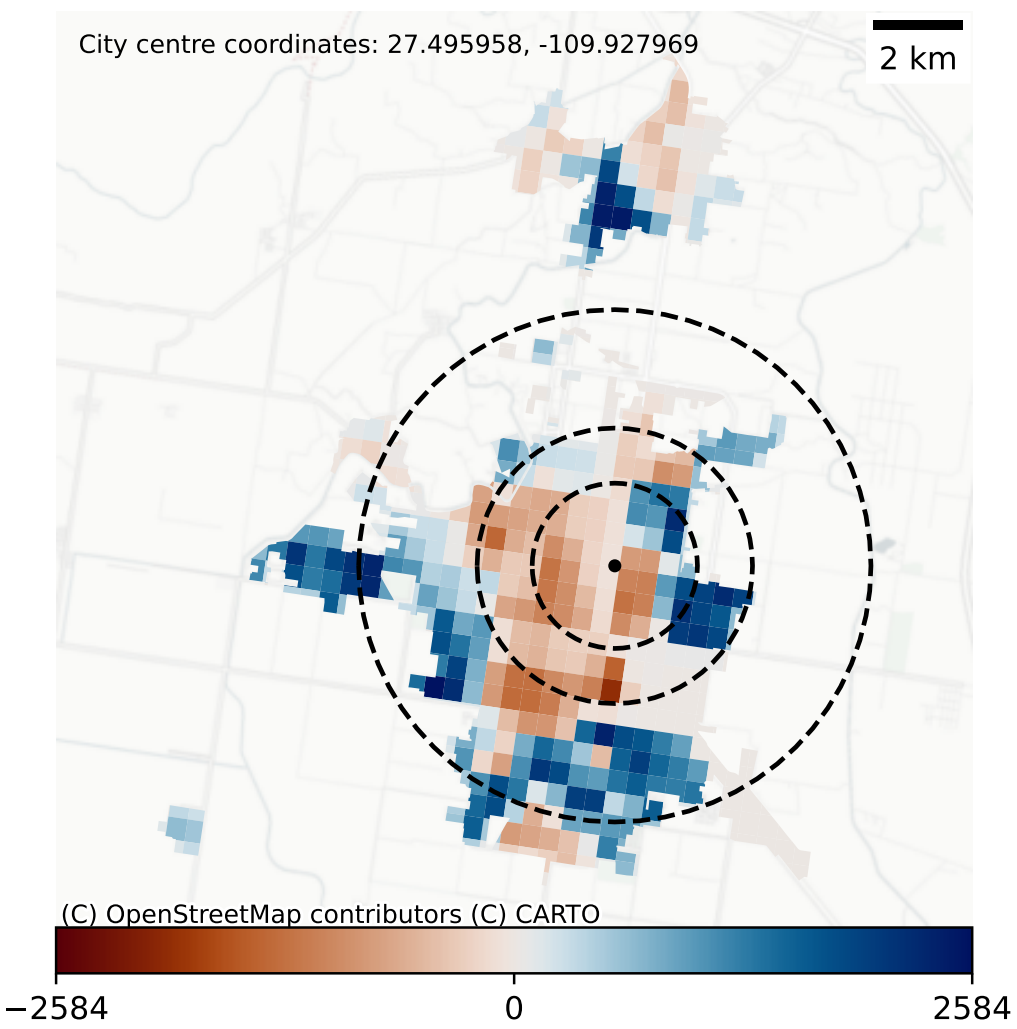

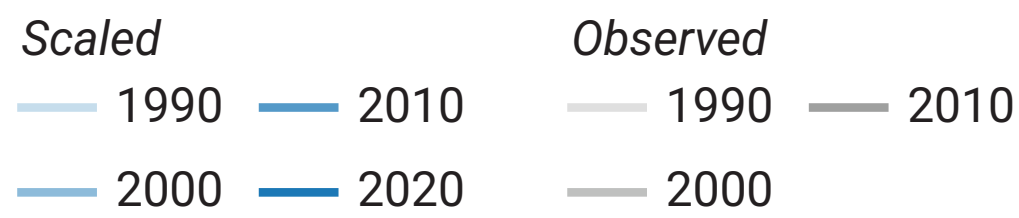

c)

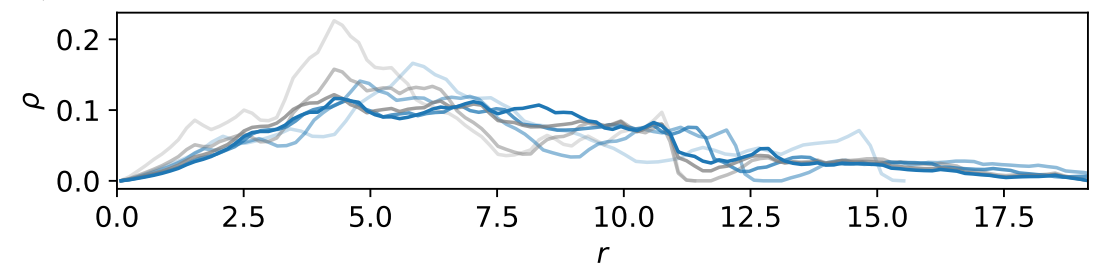

d)

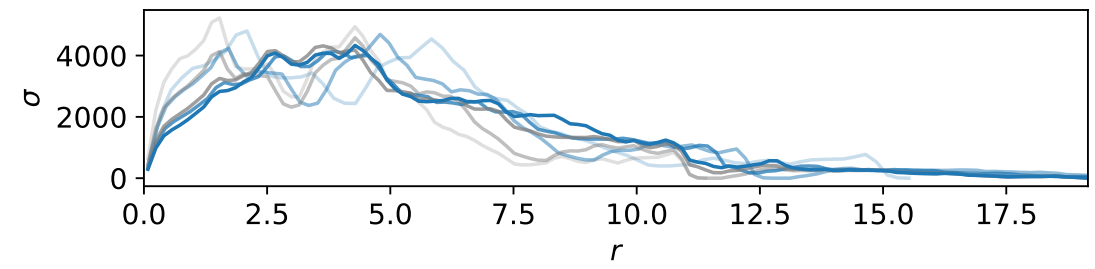

e)

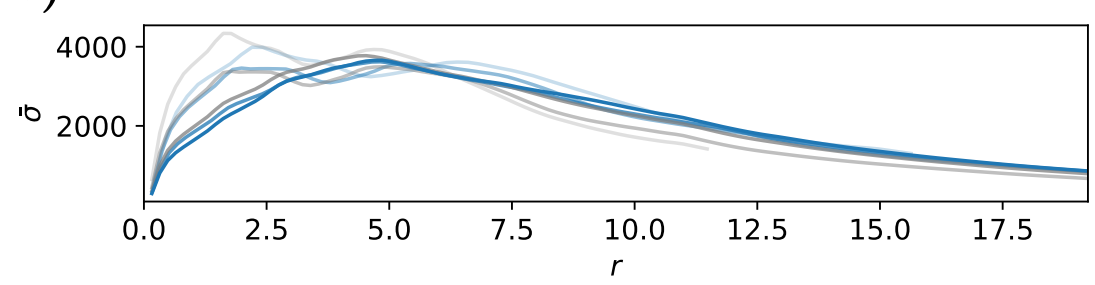

b)

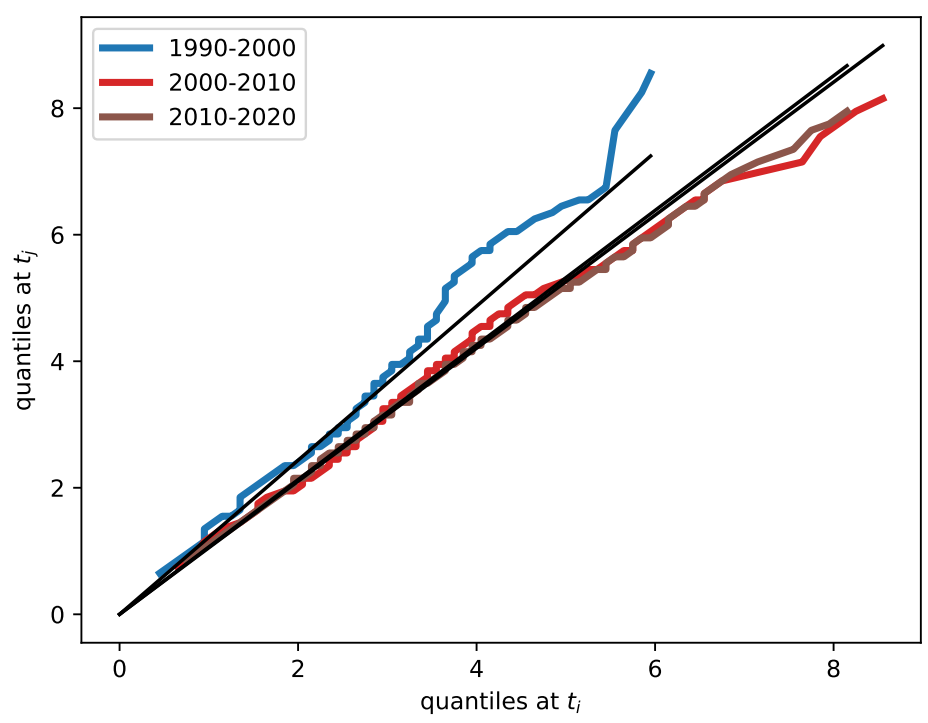

f)

Period (t_i-t_j)	$\frac{P(t_j)}{P(t_i)}$	Φ_{ij}	IQR
1990-2000	1.33	1.22	(1.19, 1.30)
2000-2010	1.18	1.05	(1.03, 1.09)
2010-2020	1.09	1.06	(1.05, 1.08)
1990-2010	1.57	1.29	(1.24, 1.40)
2000-2020	1.28	1.11	(1.09, 1.16)
1990-2020	1.70	1.38	(1.31, 1.48)

Supplementary Figure 56: Supplementary data for the metropolitan zone of Ciudad Obregón with code 26.2.02. Remoteness values are those of 2020. a) Population difference by grid cell (2020–1990); city centres are denoted as black dots. b) Quantile-quantile plots for the radial population distributions $\rho(s, t_i)$ and $\rho(s, t_j)$ (coloured curves); urban expansion factors Φ_{ij} from t_i to t_j are the estimated slopes (black lines). c) Radial population distribution $\rho(r)$ at remoteness distance r from the city centre. d) Radial population density $\sigma(r)$ at remoteness distance r from the city centre. e) Average population density $\bar{\sigma}(r)$ within disks of remoteness r with the same centre as the city. f) Urban expansion factors and their inter quartile range from the Theil-Sen estimation.

Hermosillo, 26.2.03

a)

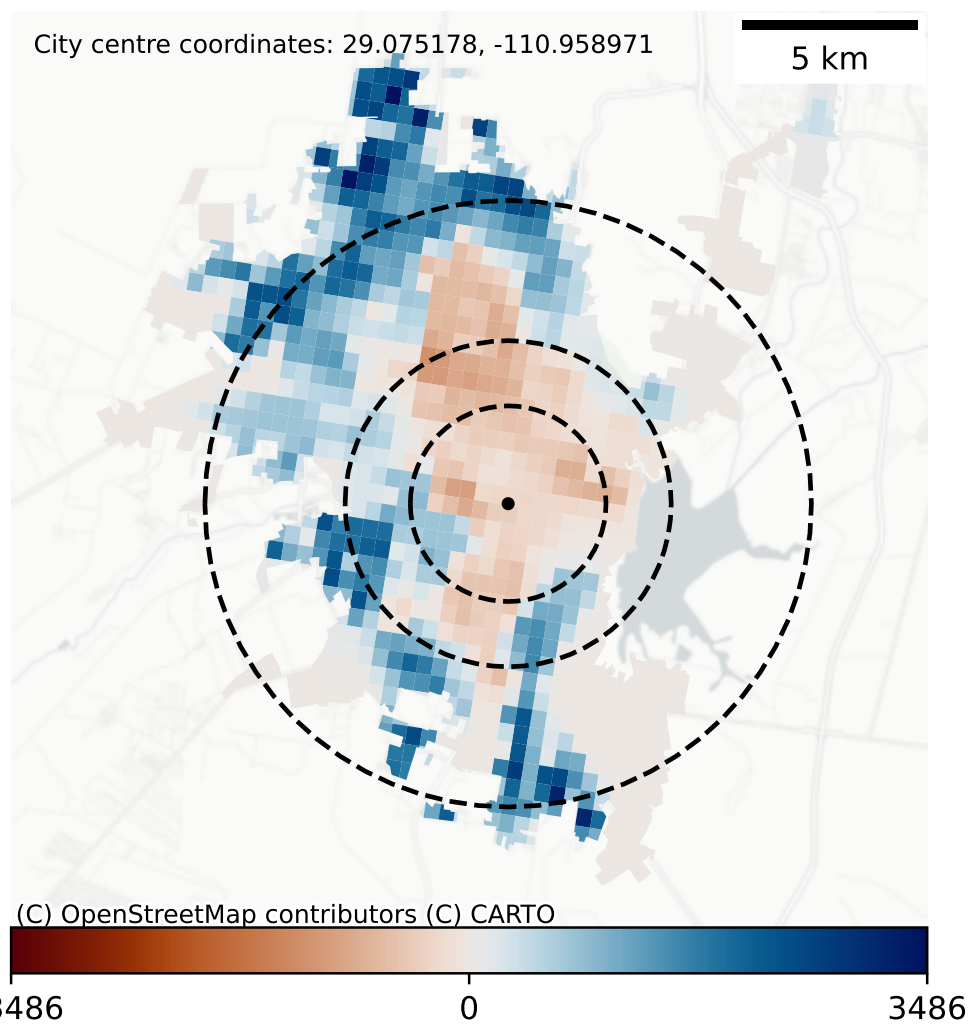

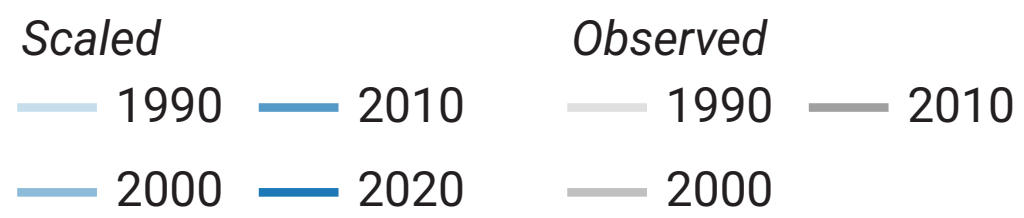

c)

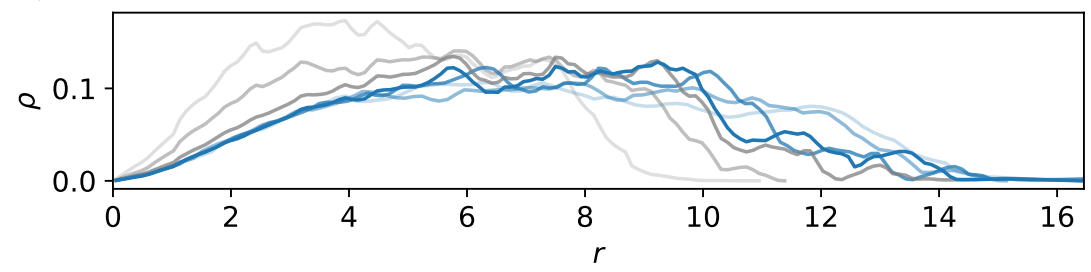

d)

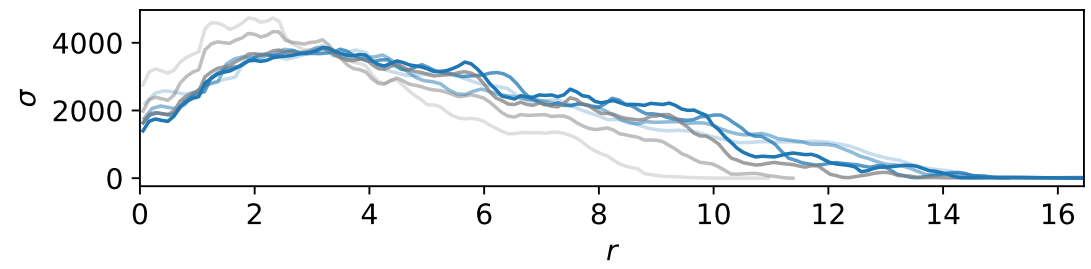

e)

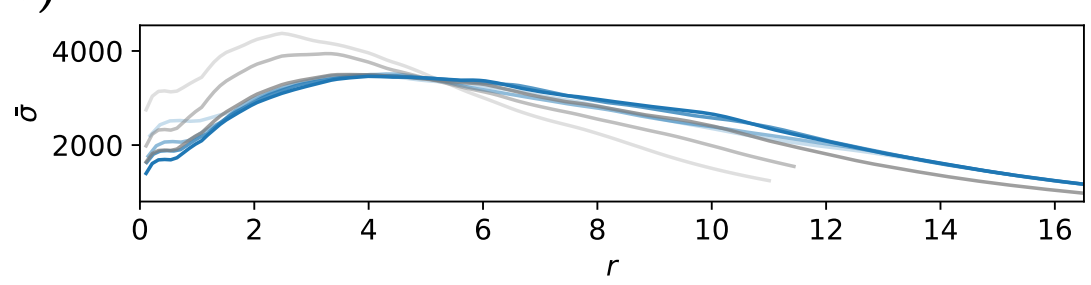

b)

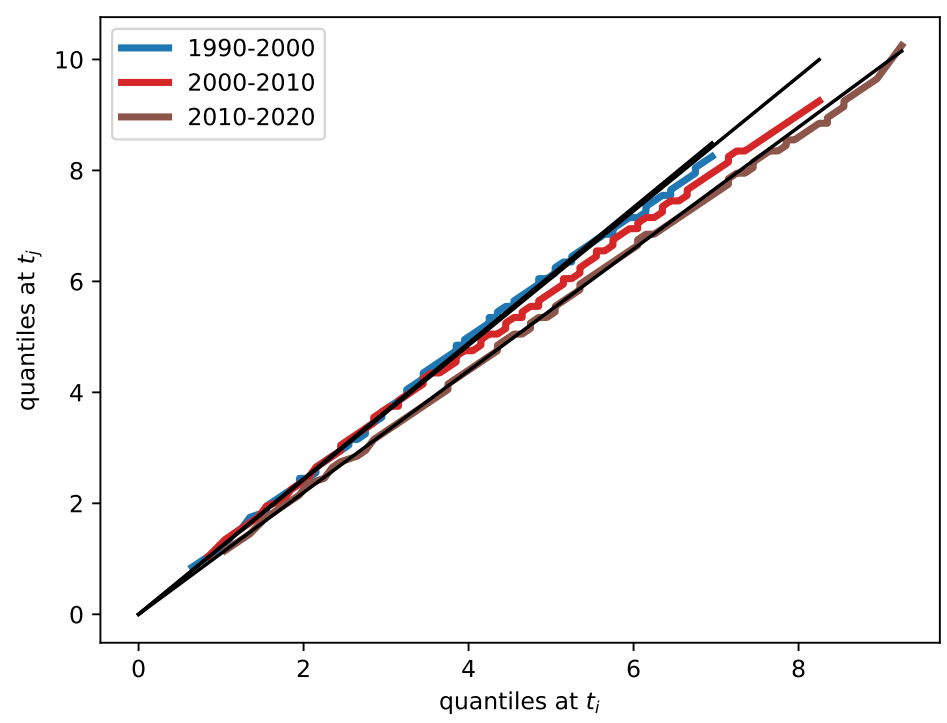

f)

Period (t_i-t_j)	$\frac{P(t_j)}{P(t_i)}$	Φ_{ij}	IQR
1990-2000	1.34	1.22	(1.21, 1.24)
2000-2010	1.32	1.21	(1.19, 1.23)
2010-2020	1.20	1.10	(1.09, 1.11)
1990-2010	1.77	1.48	(1.46, 1.51)
2000-2020	1.57	1.33	(1.32, 1.35)
1990-2020	2.11	1.62	(1.60, 1.65)

Supplementary Figure 57: Supplementary data for the metropolitan zone of Hermosillo with code 26.2.03. Remoteness values are those of 2020. a) Population difference by grid cell (2020–1990); city centres are denoted as black dots. b) Quantile-quantile plots for the radial population distributions $\rho(s, t_i)$ and $\rho(s, t_j)$ (coloured curves); urban expansion factors Φ_{ij} from t_i to t_j are the estimated slopes (black lines). c) Radial population distribution $\rho(r)$ at remoteness distance r from the city centre. d) Radial population density $\sigma(r)$ at remoteness distance r from the city centre. e) Average population density $\bar{\sigma}(r)$ within disks of remoteness r with the same centre as the city. f) Urban expansion factors and their inter quartile range from the Theil-Sen estimation.

Nogales, 26.2.04

a)

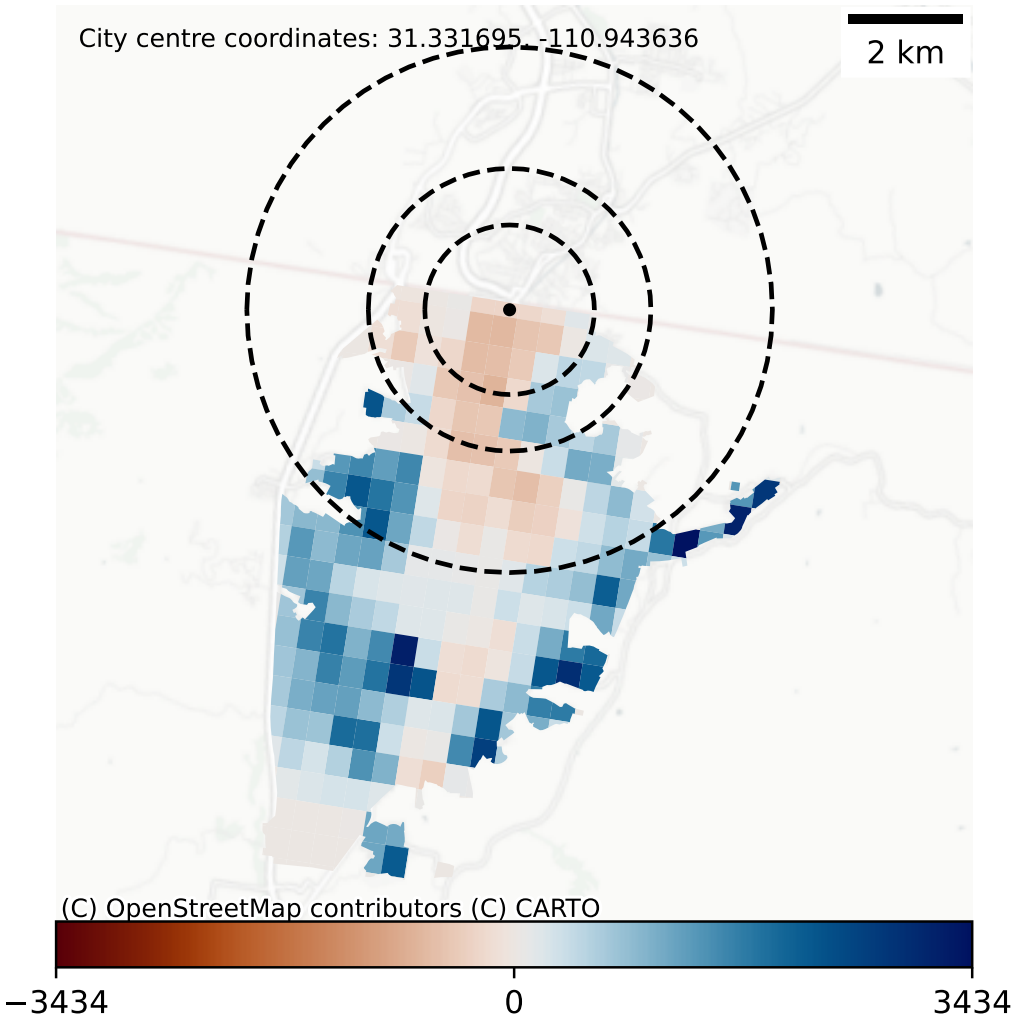

b)

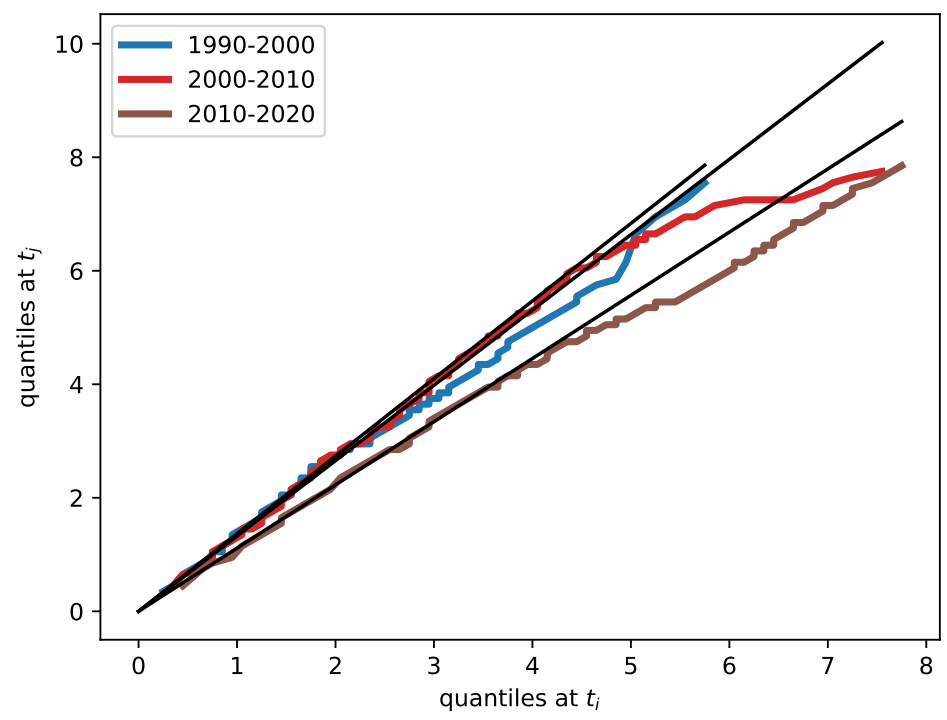

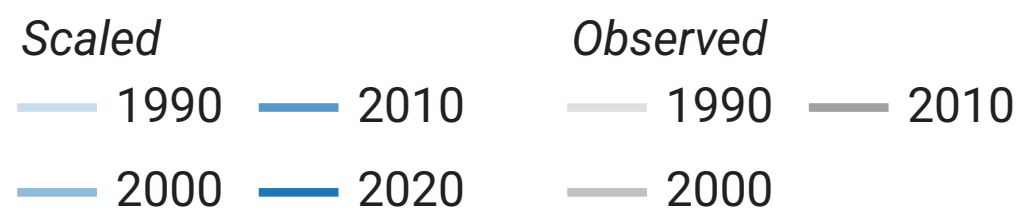

c)

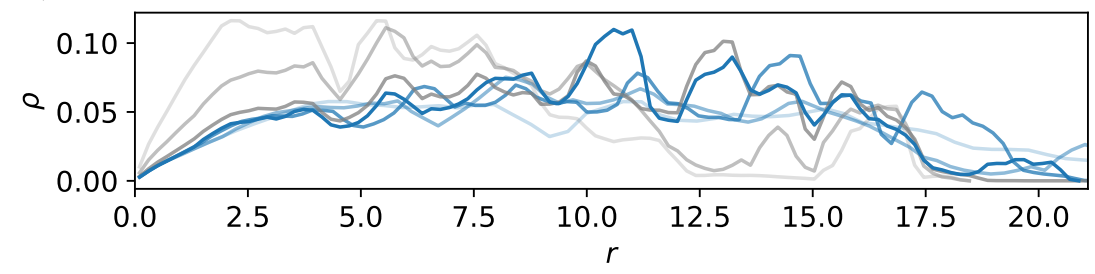

d)

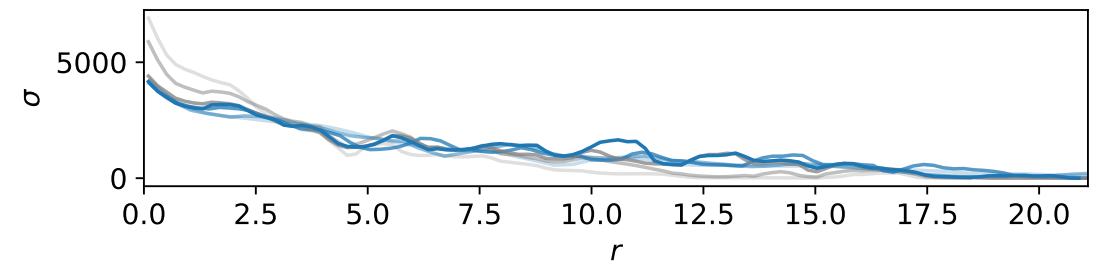

e)

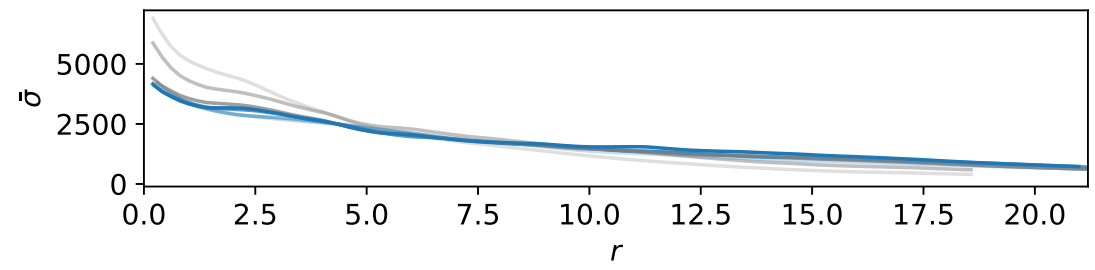

f)

Period (t_i-t_j)	$\frac{P(t_j)}{P(t_i)}$	Φ_{ij}	IQR
1990-2000	1.48	1.37	(1.34, 1.40)
2000-2010	1.35	1.33	(1.30, 1.37)
2010-2020	1.16	1.11	(1.09, 1.13)
1990-2010	2.01	1.82	(1.78, 1.87)
2000-2020	1.57	1.48	(1.44, 1.51)
1990-2020	2.32	2.03	(1.94, 2.09)

Supplementary Figure 58: Supplementary data for the metropolitan zone of Nogales with code 26.2.04. Remoteness values are those of 2020. a) Population difference by grid cell (2020–1990); city centres are denoted as black dots. b) Quantile-quantile plots for the radial population distributions $\rho(s, t_i)$ and $\rho(s, t_j)$ (coloured curves); urban expansion factors Φ_{ij} from t_i to t_j are the estimated slopes (black lines). c) Radial population distribution $\rho(r)$ at remoteness distance r from the city centre. d) Radial population density $\sigma(r)$ at remoteness distance r from the city centre. e) Average population density $\bar{\sigma}(r)$ within disks of remoteness r with the same centre as the city. f) Urban expansion factors and their inter quartile range from the Theil-Sen estimation.

Villahermosa, 27.1.01

a)

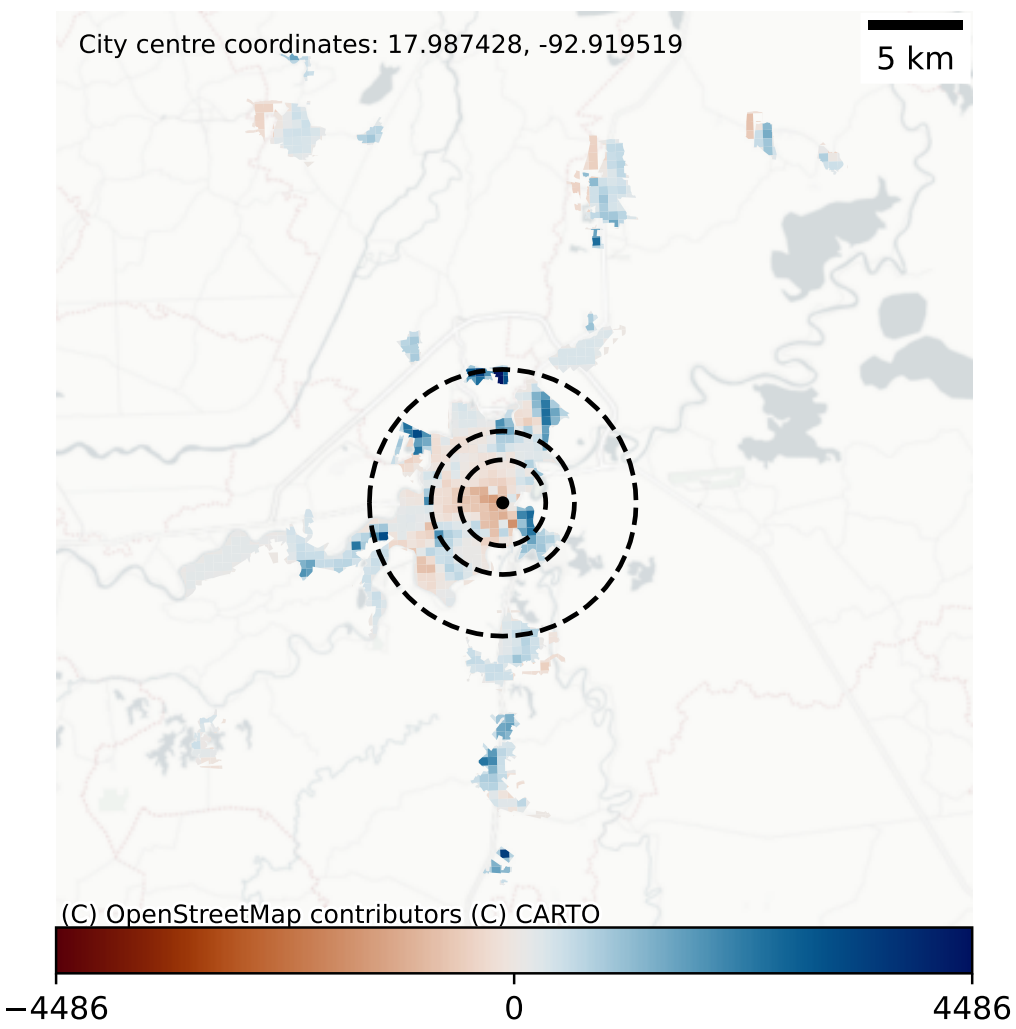

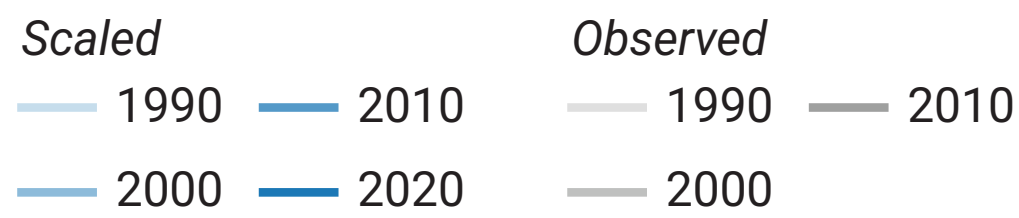

c)

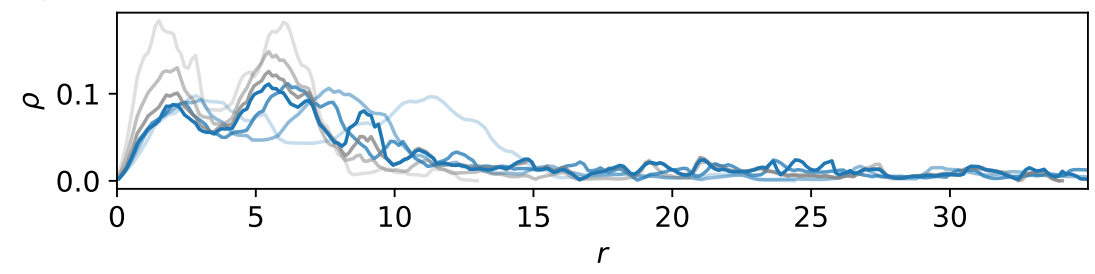

d)

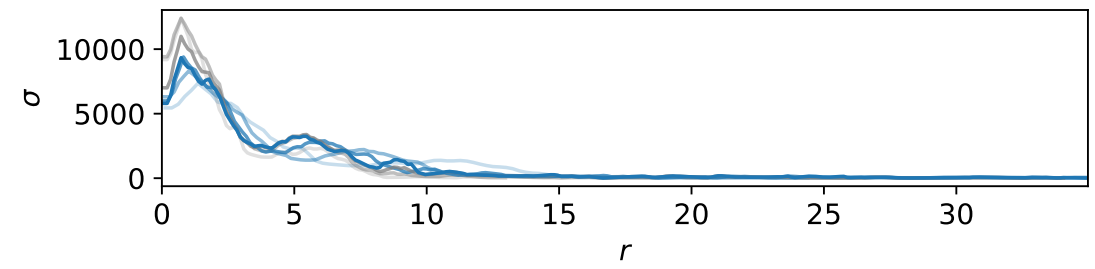

e)

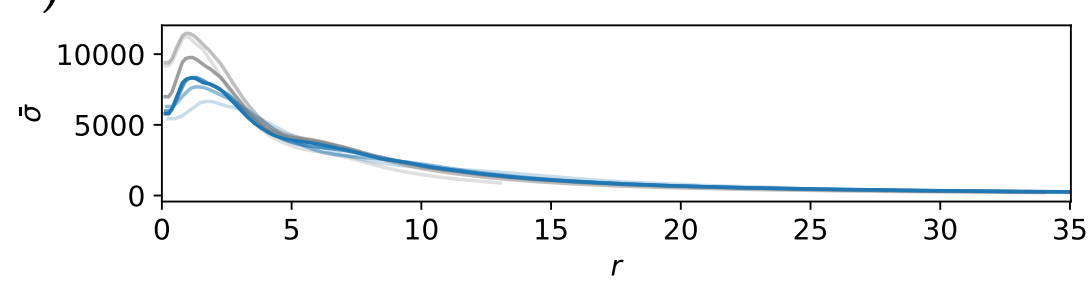

b)

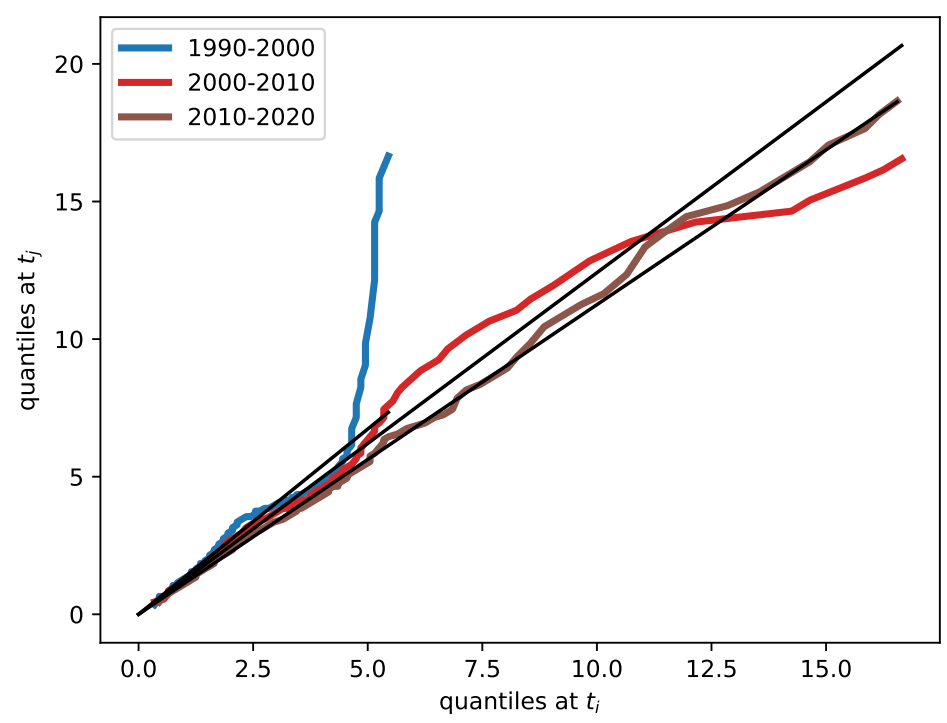

f)

Period (t_i-t_j)	$\frac{P(t_j)}{P(t_i)}$	Φ_{ij}	IQR
1990-2000	1.60	1.35	(1.29, 1.44)
2000-2010	1.21	1.24	(1.20, 1.30)
2010-2020	1.08	1.13	(1.09, 1.15)
1990-2010	1.94	1.68	(1.57, 1.83)
2000-2020	1.31	1.40	(1.32, 1.46)
1990-2020	2.09	1.93	(1.79, 2.03)

Supplementary Figure 59: Supplementary data for the metropolitan zone of Villahermosa with code 27.1.01. Remoteness values are those of 2020. a) Population difference by grid cell (2020–1990); city centres are denoted as black dots. b) Quantile-quantile plots for the radial population distributions $\rho(s, t_i)$ and $\rho(s, t_j)$ (coloured curves); urban expansion factors Φ_{ij} from t_i to t_j are the estimated slopes (black lines). c) Radial population distribution $\rho(r)$ at remoteness distance r from the city centre. d) Radial population density $\sigma(r)$ at remoteness distance r from the city centre. e) Average population density $\bar{\sigma}(r)$ within disks of remoteness r with the same centre as the city. f) Urban expansion factors and their inter quartile range from the Theil-Sen estimation.

Reynosa, 28.1.01

a)

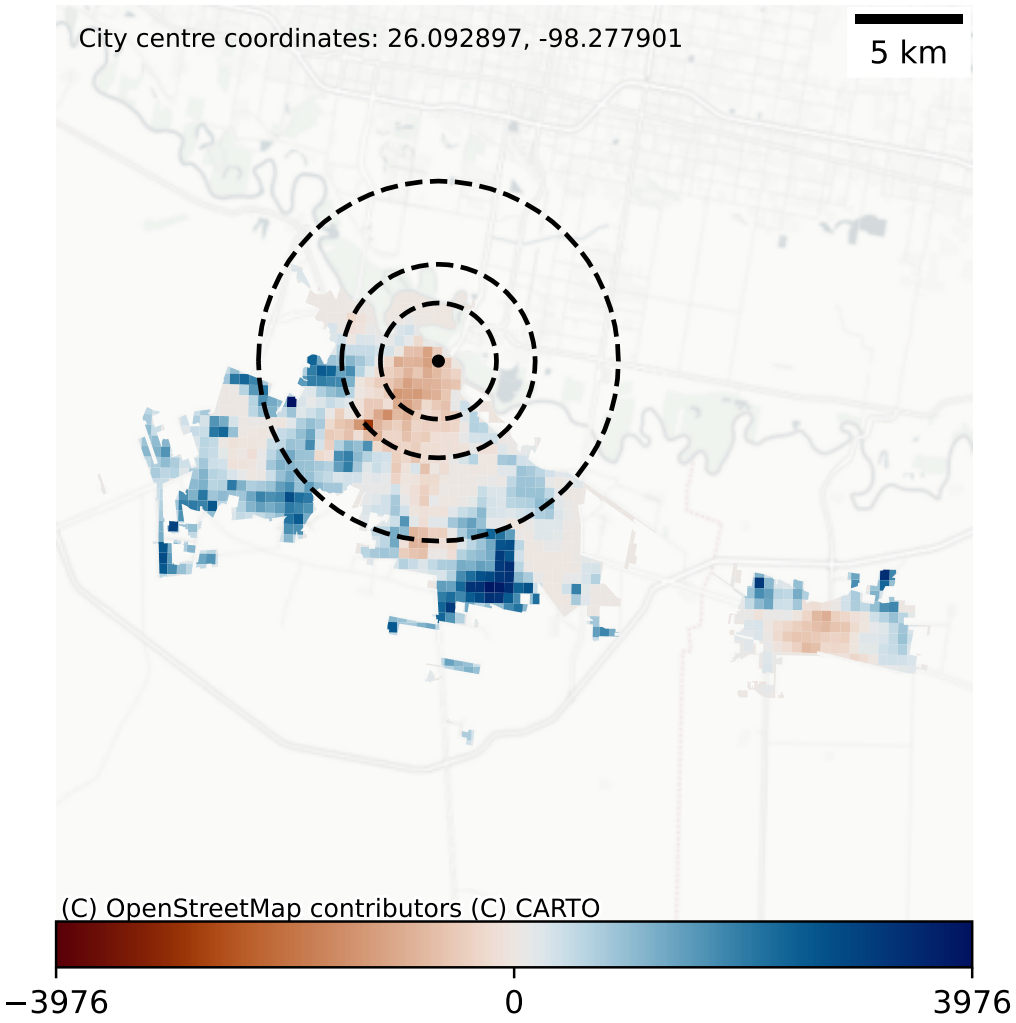

b)

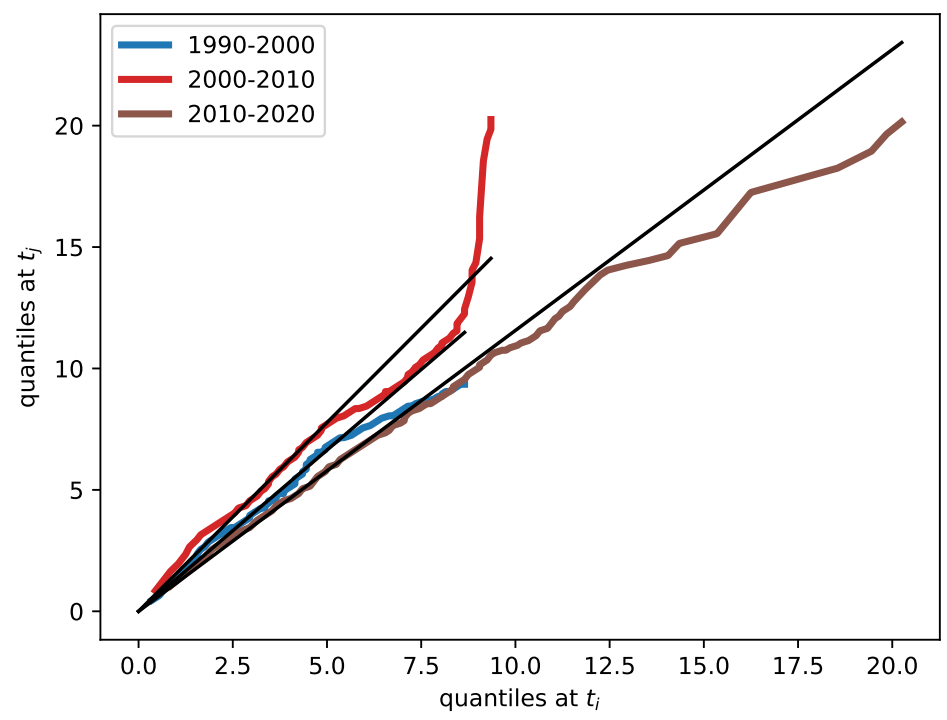

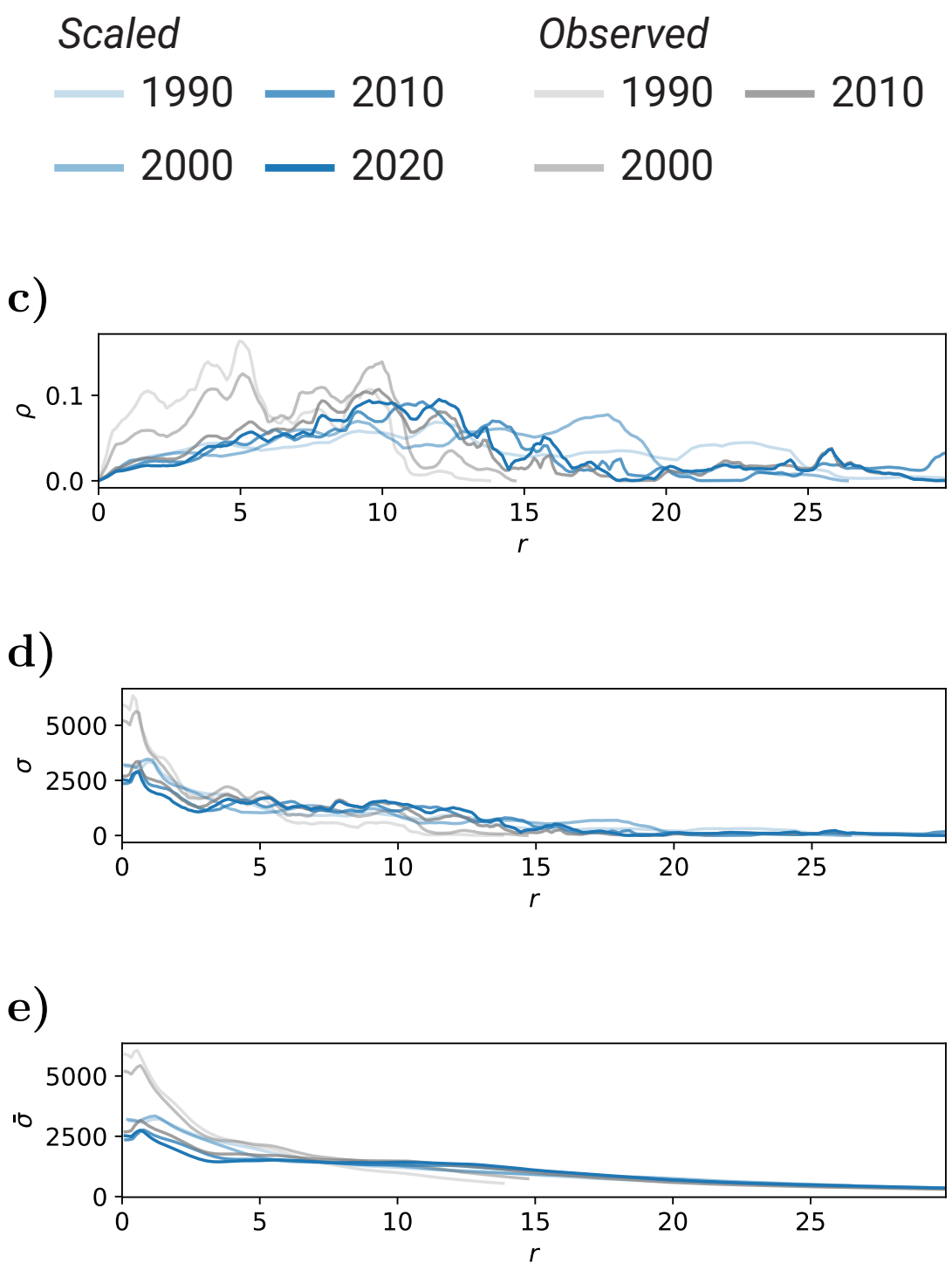

f)

Period (t_i-t_j)	$\frac{P(t_j)}{P(t_i)}$	Φ_{ij}	IQR
1990-2000	1.52	1.33	(1.30, 1.41)
2000-2010	1.70	1.55	(1.54, 1.72)
2010-2020	1.17	1.16	(1.14, 1.18)
1990-2010	2.58	2.17	(2.02, 2.44)
2000-2020	1.99	1.78	(1.75, 2.02)
1990-2020	3.02	2.54	(2.31, 2.85)

Supplementary Figure 60: Supplementary data for the metropolitan zone of Reynosa with code 28.1.01. Remoteness values are those of 2020. a) Population difference by grid cell (2020–1990); city centres are denoted as black dots. b) Quantile-quantile plots for the radial population distributions $\rho(s, t_i)$ and $\rho(s, t_j)$ (coloured curves); urban expansion factors Φ_{ij} from t_i to t_j are the estimated slopes (black lines). c) Radial population distribution $\rho(r)$ at remoteness distance r from the city centre. d) Radial population density $\sigma(r)$ at remoteness distance r from the city centre. e) Average population density $\bar{\sigma}(r)$ within disks of remoteness r with the same centre as the city. f) Urban expansion factors and their inter quartile range from the Theil-Sen estimation.

Tampico, 28.1.02

a)

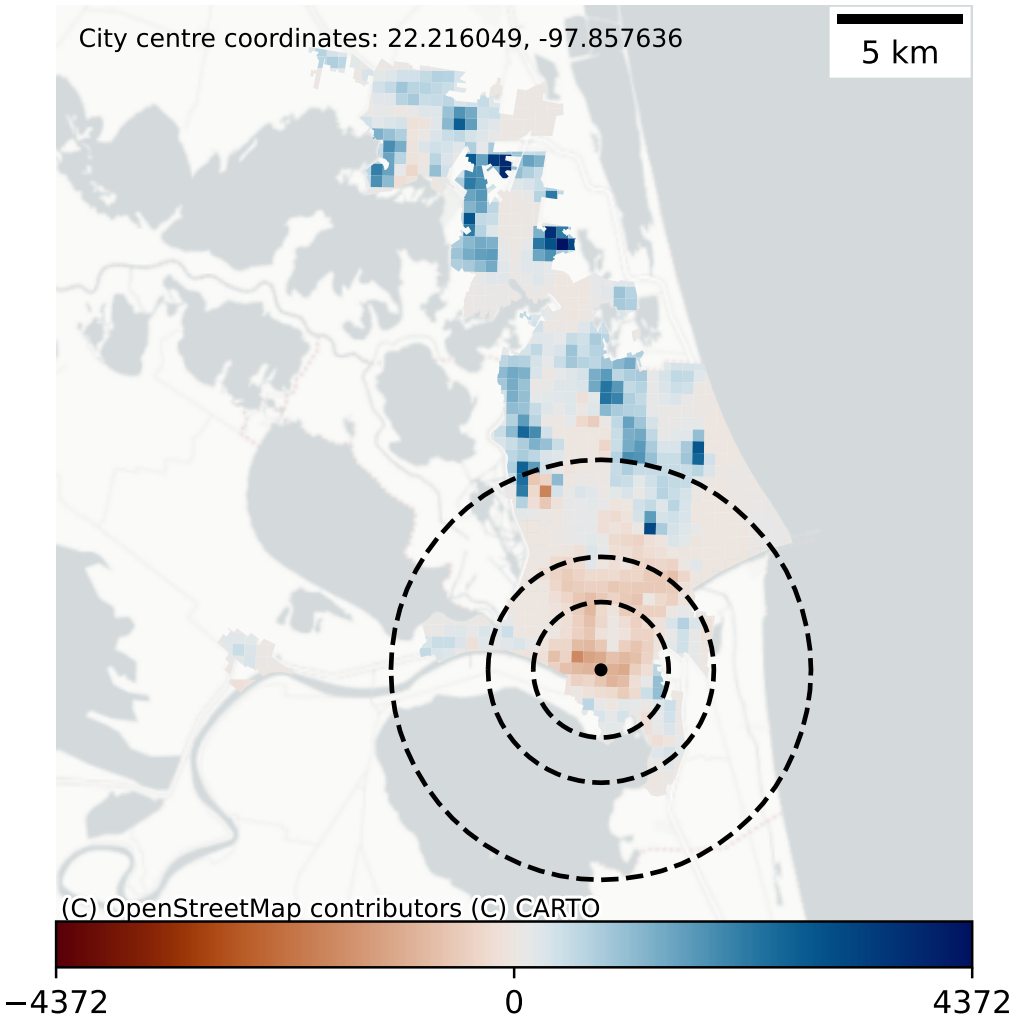

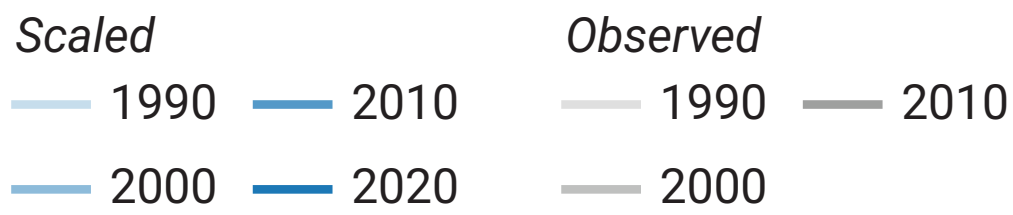

c)

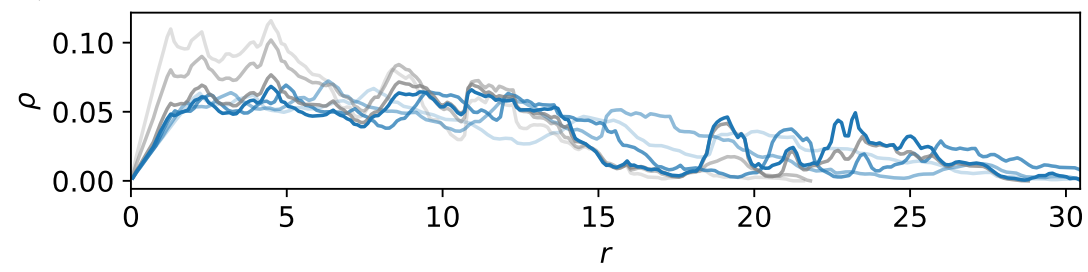

d)

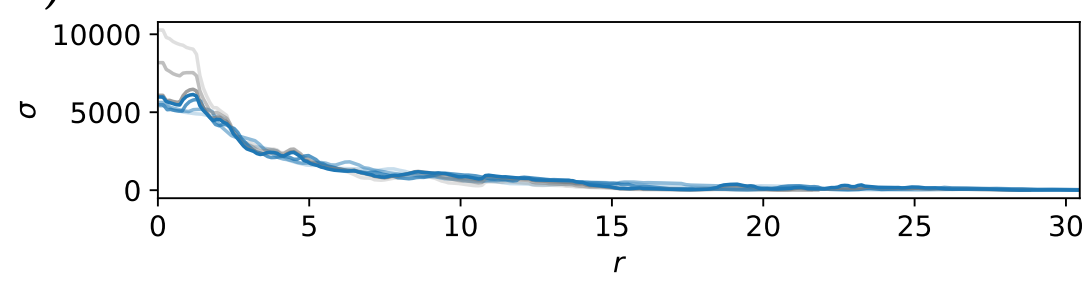

e)

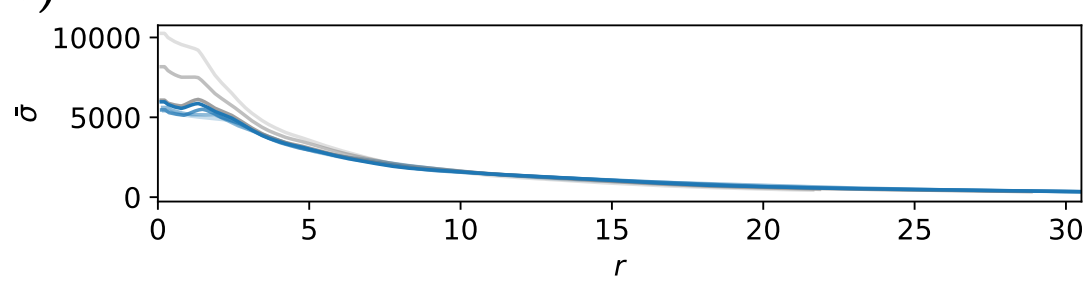

b)

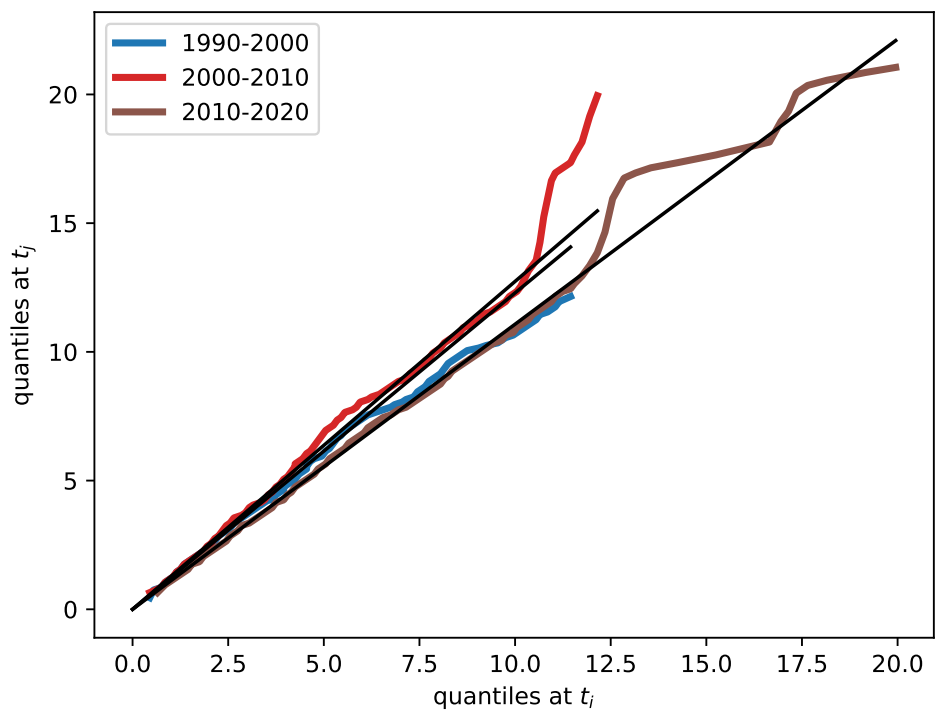

f)

Period (t_i-t_j)	$\frac{P(t_j)}{P(t_i)}$	Φ_{ij}	IQR
1990-2000	1.15	1.23	(1.20, 1.25)
2000-2010	1.24	1.27	(1.25, 1.30)
2010-2020	1.10	1.11	(1.09, 1.13)
1990-2010	1.43	1.56	(1.54, 1.58)
2000-2020	1.37	1.41	(1.38, 1.44)
1990-2020	1.58	1.74	(1.72, 1.76)

Supplementary Figure 61: Supplementary data for the metropolitan zone of Tampico with code 28.1.02. Remoteness values are those of 2020. a) Population difference by grid cell (2020–1990); city centres are denoted as black dots. b) Quantile-quantile plots for the radial population distributions $\rho(s, t_i)$ and $\rho(s, t_j)$ (coloured curves); urban expansion factors Φ_{ij} from t_i to t_j are the estimated slopes (black lines). c) Radial population distribution $\rho(r)$ at remoteness distance r from the city centre. d) Radial population density $\sigma(r)$ at remoteness distance r from the city centre. e) Average population density $\bar{\sigma}(r)$ within disks of remoteness r with the same centre as the city. f) Urban expansion factors and their inter quartile range from the Theil-Sen estimation.

Ciudad Victoria, 28.2.03

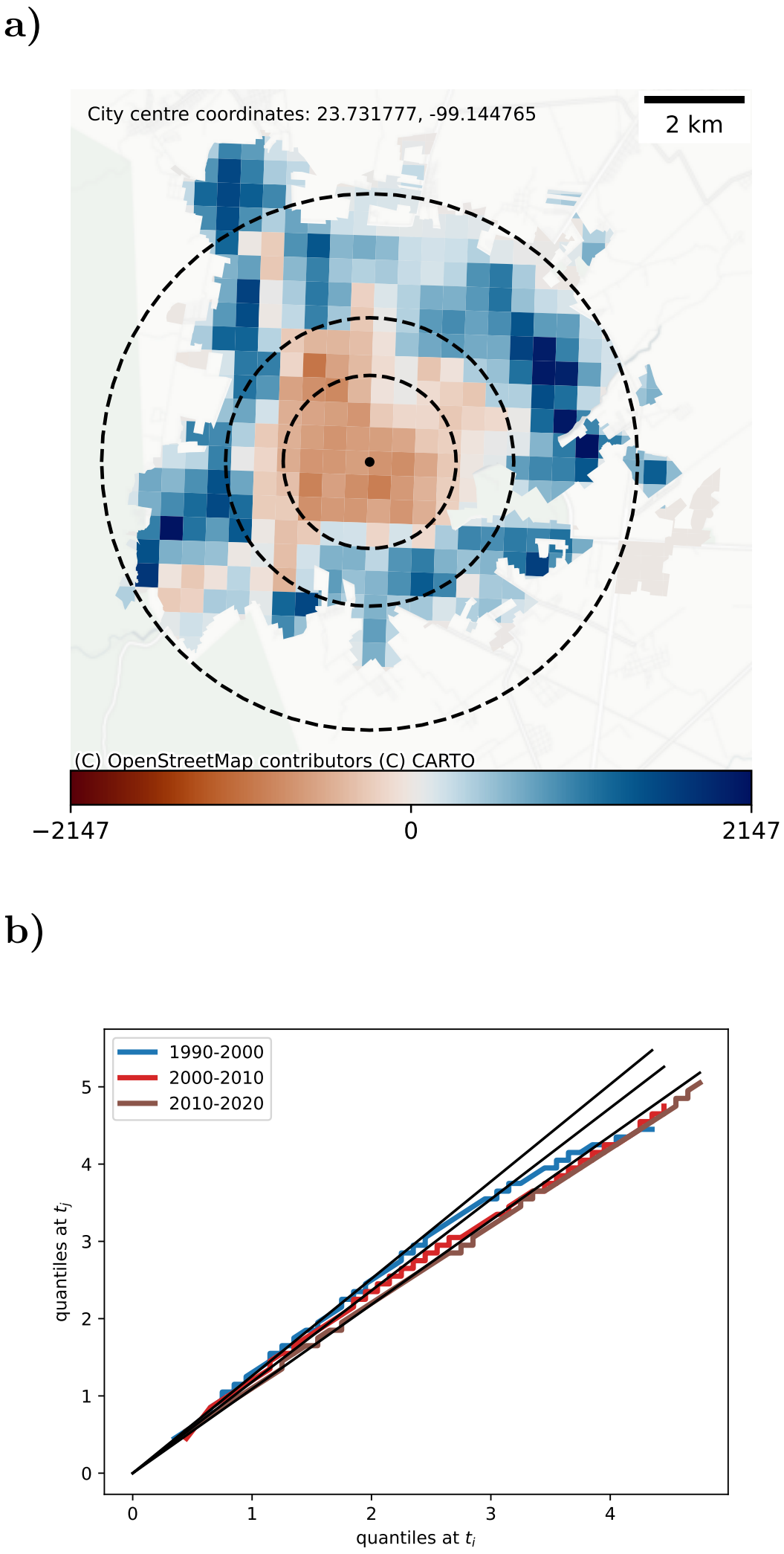

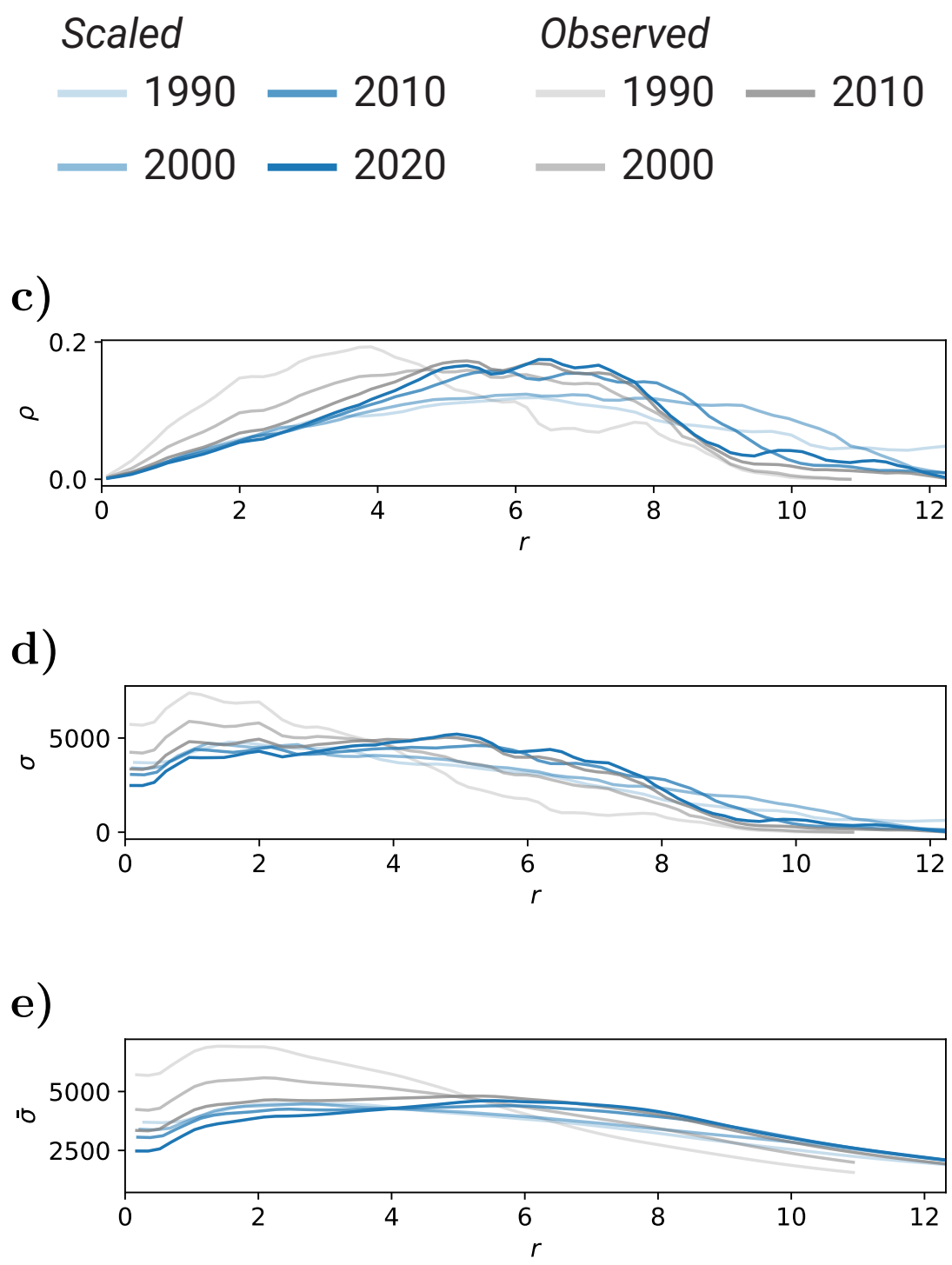

f)

Period (t_i-t_j)	$\frac{P(t_j)}{P(t_i)}$	Φ_{ij}	IQR
1990-2000	1.28	1.26	(1.23, 1.29)
2000-2010	1.23	1.18	(1.15, 1.21)
2010-2020	1.09	1.09	(1.08, 1.11)
1990-2010	1.56	1.48	(1.44, 1.53)
2000-2020	1.33	1.29	(1.25, 1.32)
1990-2020	1.70	1.62	(1.57, 1.70)

Supplementary Figure 62: Supplementary data for the metropolitan zone of Ciudad Victoria with code 28.2.03. Remoteness values are those of 2020. a) Population difference by grid cell (2020–1990); city centres are denoted as black dots. b) Quantile-quantile plots for the radial population distributions $\rho(s, t_i)$ and $\rho(s, t_j)$ (coloured curves); urban expansion factors Φ_{ij} from t_i to t_j are the estimated slopes (black lines). c) Radial population distribution $\rho(r)$ at remoteness distance r from the city centre. d) Radial population density $\sigma(r)$ at remoteness distance r from the city centre. e) Average population density $\bar{\sigma}(r)$ within disks of remoteness r with the same centre as the city. f) Urban expansion factors and their inter quartile range from the Theil-Sen estimation.

Matamoros, 28.2.04

a)

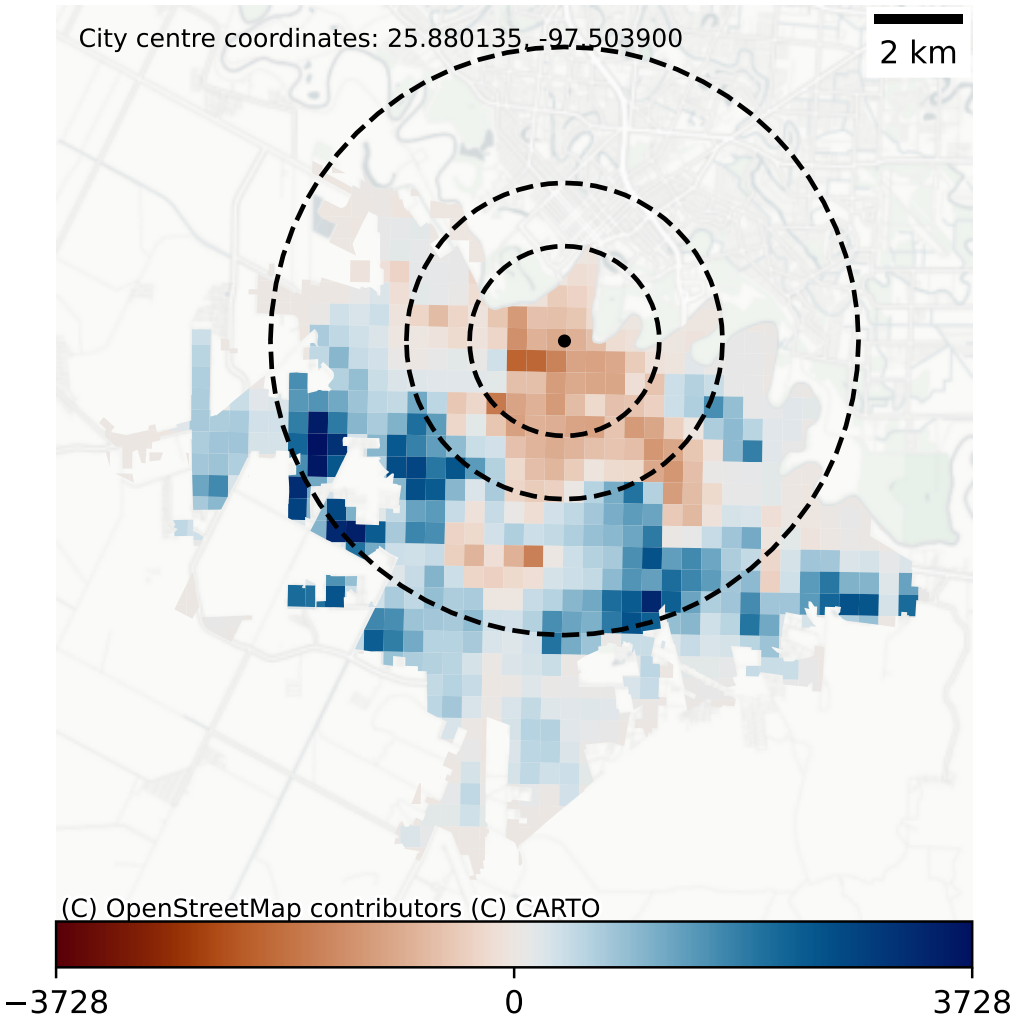

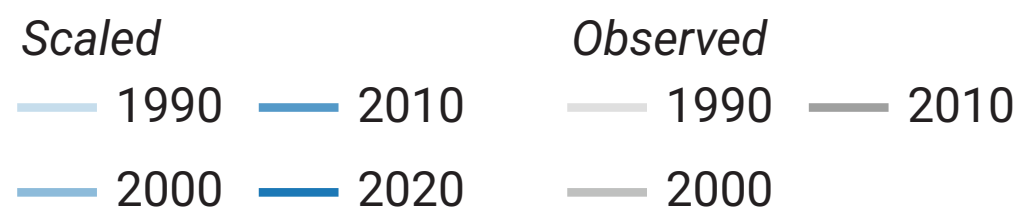

c)

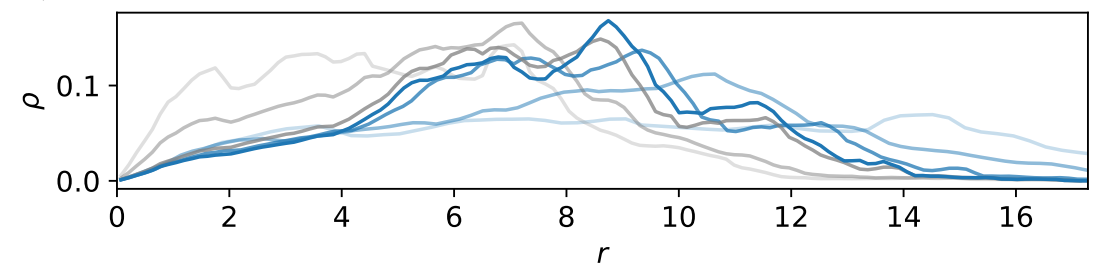

d)

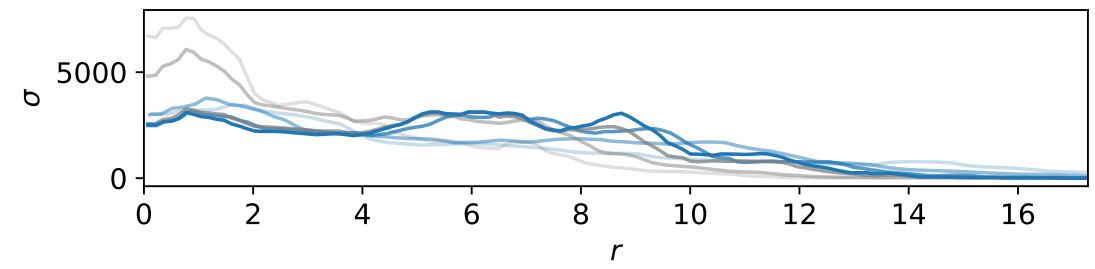

e)

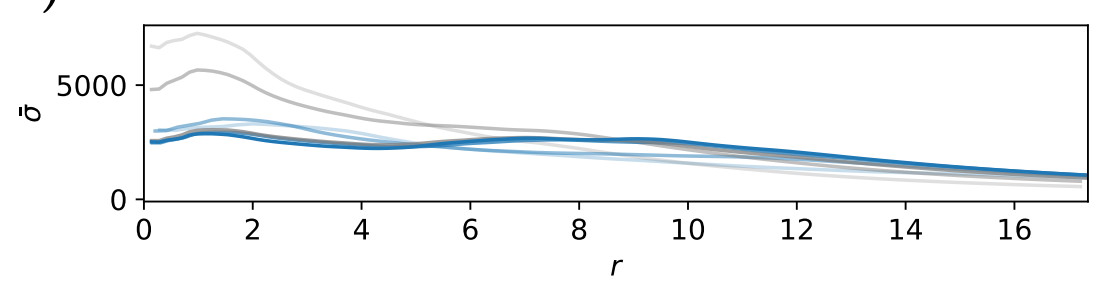

b)

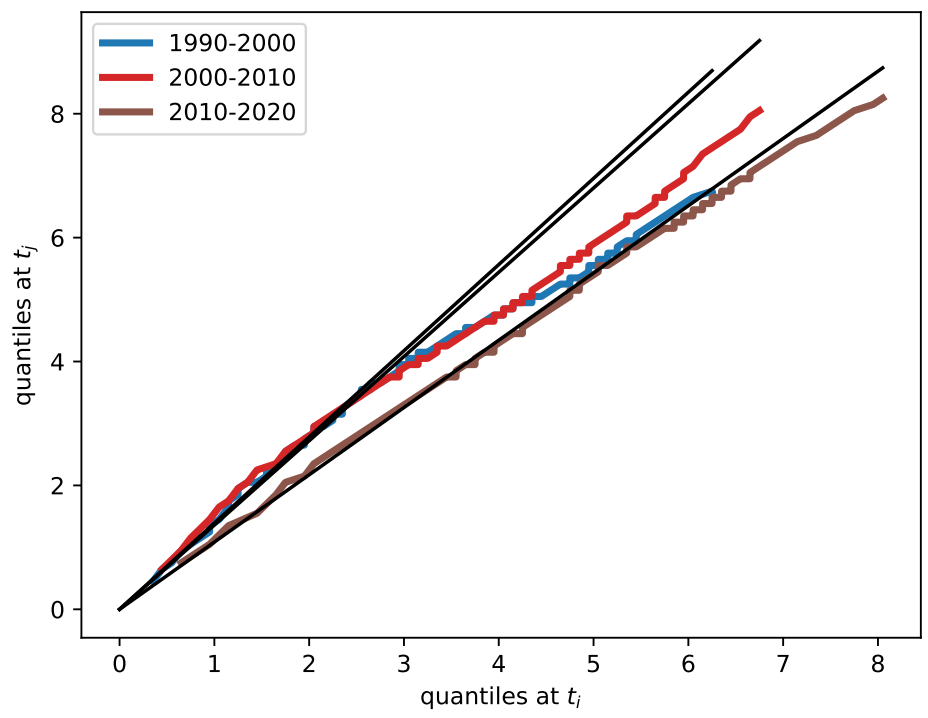

f)

Period (t_i-t_j)	$\frac{P(t_j)}{P(t_i)}$	Φ_{ij}	IQR
1990-2000	1.41	1.39	(1.36, 1.43)
2000-2010	1.20	1.36	(1.27, 1.44)
2010-2020	1.14	1.09	(1.07, 1.11)
1990-2010	1.69	1.92	(1.74, 2.08)
2000-2020	1.36	1.48	(1.35, 1.61)
1990-2020	1.92	2.11	(1.85, 2.33)

Supplementary Figure 63: Supplementary data for the metropolitan zone of Matamoros with code 28.2.04. Remoteness values are those of 2020. a) Population difference by grid cell (2020–1990); city centres are denoted as black dots. b) Quantile-quantile plots for the radial population distributions $\rho(s, t_i)$ and $\rho(s, t_j)$ (coloured curves); urban expansion factors Φ_{ij} from t_i to t_j are the estimated slopes (black lines). c) Radial population distribution $\rho(r)$ at remoteness distance r from the city centre. d) Radial population density $\sigma(r)$ at remoteness distance r from the city centre. e) Average population density $\bar{\sigma}(r)$ within disks of remoteness r with the same centre as the city. f) Urban expansion factors and their inter quartile range from the Theil-Sen estimation.

Nuevo Laredo, 28.2.05

a)

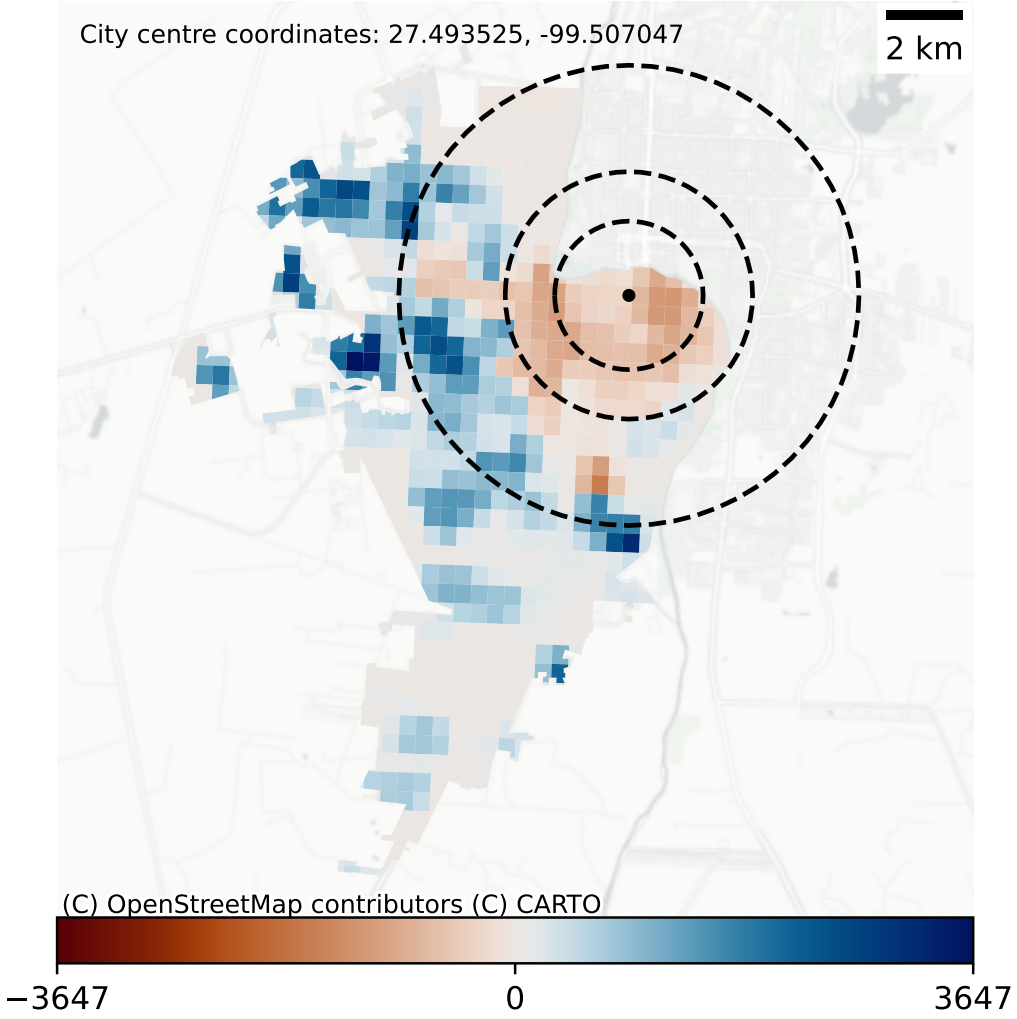

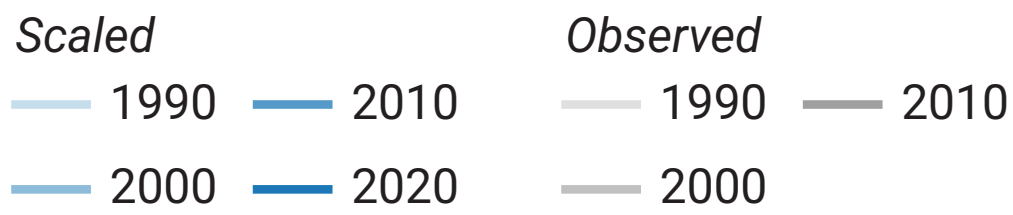

c)

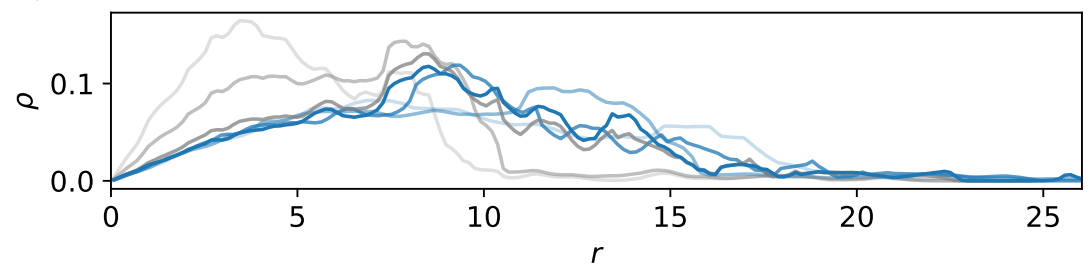

d)

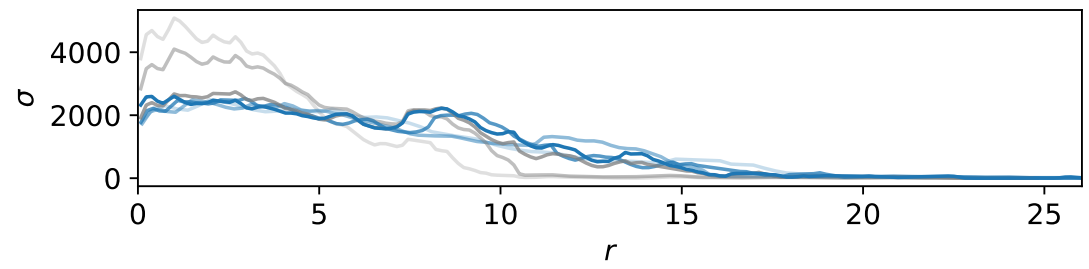

e)

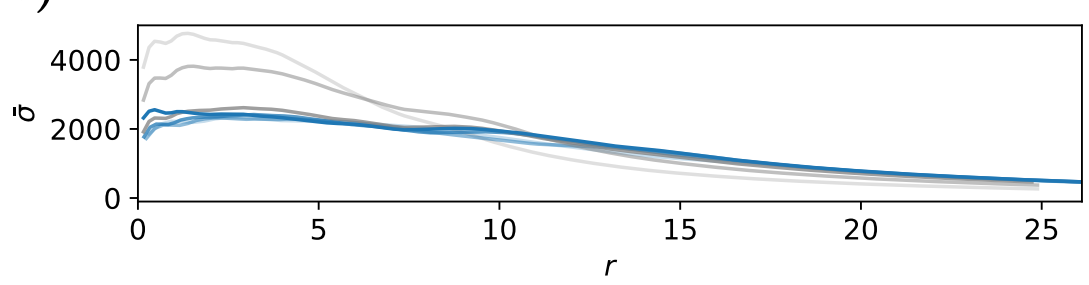

b)

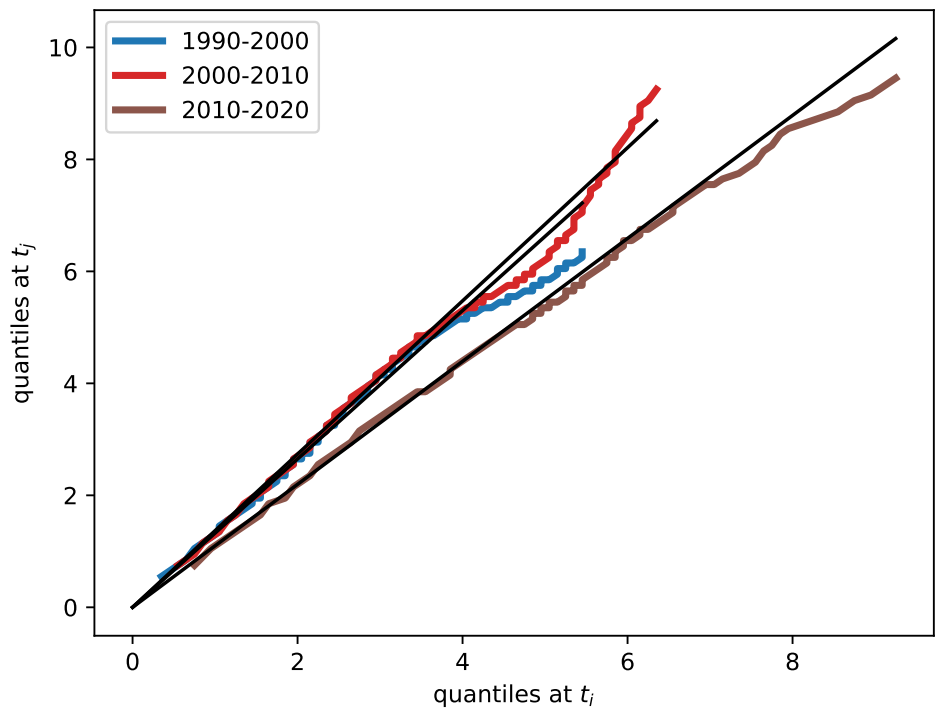

f)

Period (t_i-t_j)	$\frac{P(t_j)}{P(t_i)}$	Φ_{ij}	IQR
1990-2000	1.41	1.32	(1.30, 1.35)
2000-2010	1.23	1.37	(1.34, 1.39)
2010-2020	1.11	1.10	(1.09, 1.12)
1990-2010	1.74	1.81	(1.76, 1.86)
2000-2020	1.37	1.51	(1.47, 1.53)
1990-2020	1.94	2.00	(1.96, 2.03)

Supplementary Figure 64: Supplementary data for the metropolitan zone of Nuevo Laredo with code 28.2.05. Remoteness values are those of 2020. a) Population difference by grid cell (2020–1990); city centres are denoted as black dots. b) Quantile-quantile plots for the radial population distributions $\rho(s, t_i)$ and $\rho(s, t_j)$ (coloured curves); urban expansion factors Φ_{ij} from t_i to t_j are the estimated slopes (black lines). c) Radial population distribution $\rho(r)$ at remoteness distance r from the city centre. d) Radial population density $\sigma(r)$ at remoteness distance r from the city centre. e) Average population density $\bar{\sigma}(r)$ within disks of remoteness r with the same centre as the city. f) Urban expansion factors and their inter quartile range from the Theil-Sen estimation.

Tlaxcala-Apizaco, 29.1.01

a)

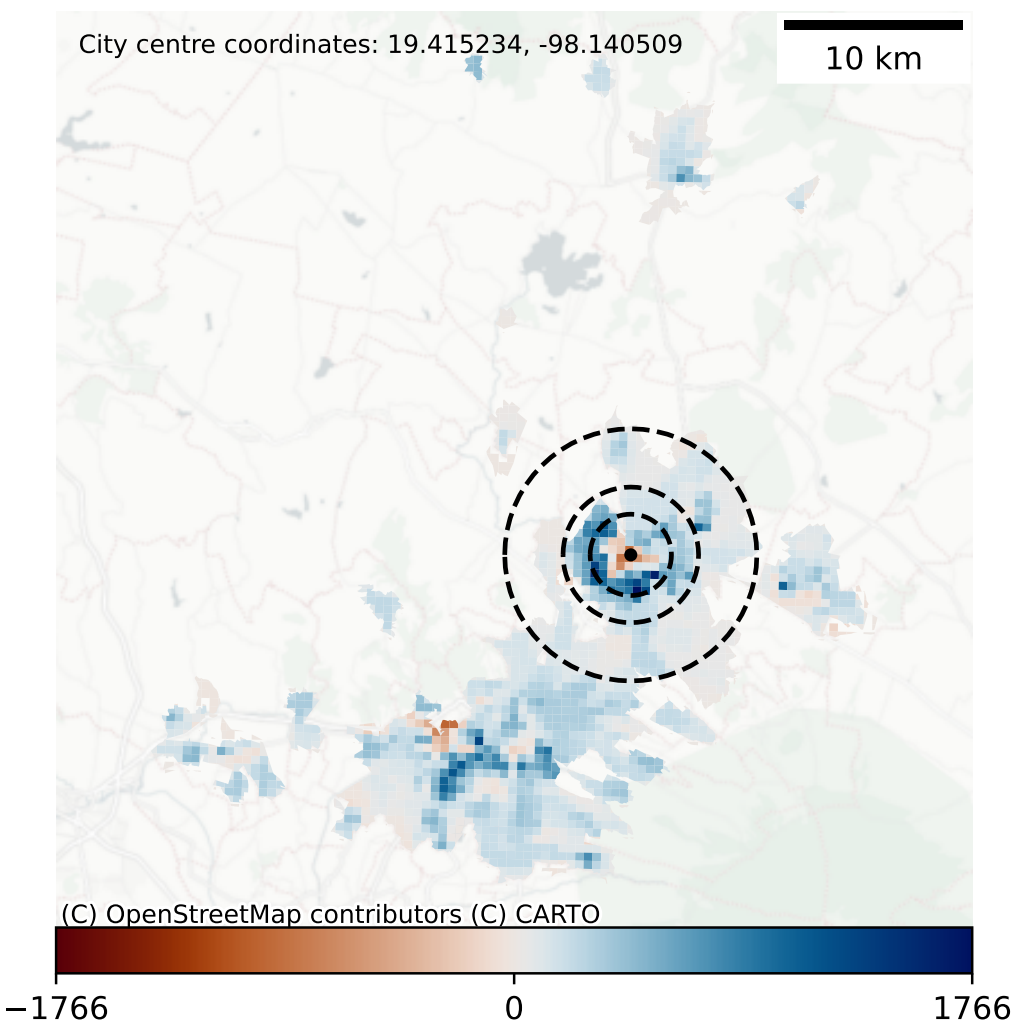

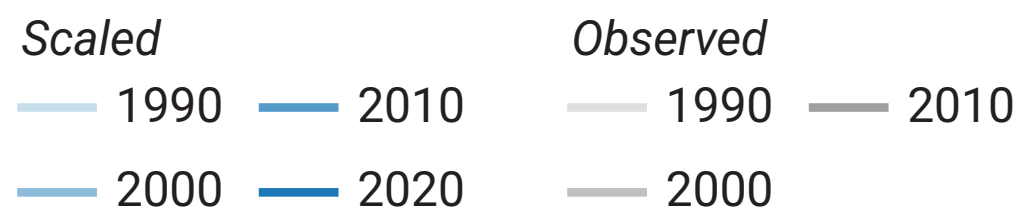

c)

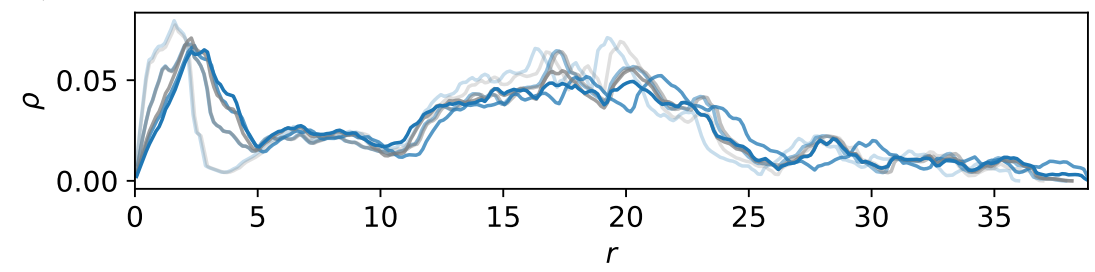

d)

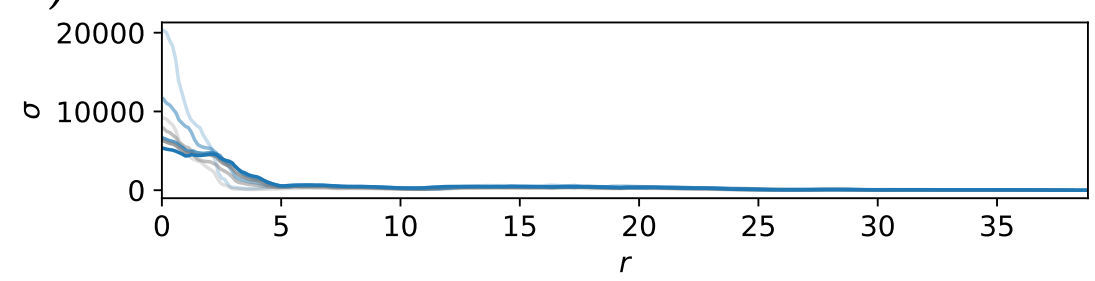

e)

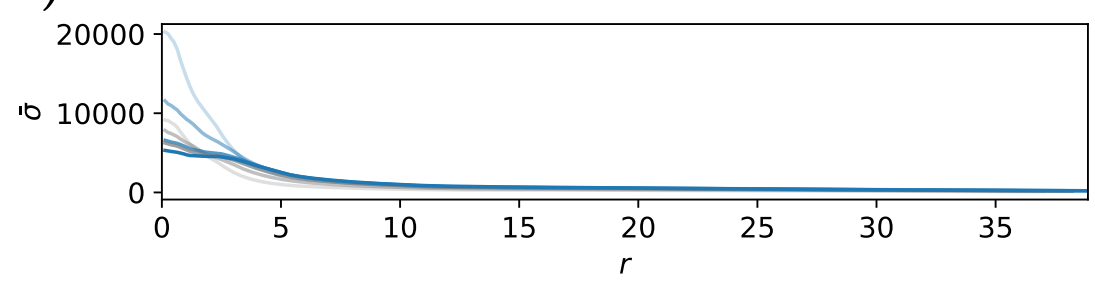

b)

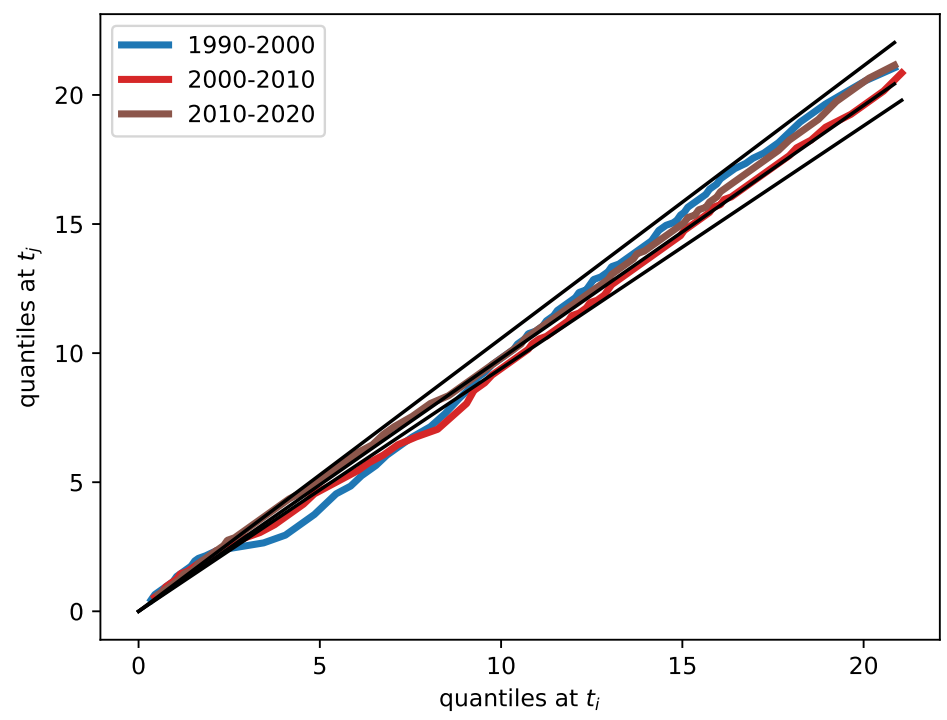

f)

Period (t_i-t_j)	$\frac{P(t_j)}{P(t_i)}$	Φ_{ij}	IQR
1990-2000	1.44	0.98	(0.88, 1.23)
2000-2010	1.24	0.94	(0.91, 1.12)
2010-2020	1.18	1.06	(1.01, 1.10)
1990-2010	1.78	0.91	(0.80, 1.37)
2000-2020	1.46	0.96	(0.92, 1.23)
1990-2020	2.10	0.89	(0.81, 1.51)

Supplementary Figure 65: Supplementary data for the metropolitan zone of Tlaxcala-Apizaco with code 29.1.01. Remoteness values are those of 2020. a) Population difference by grid cell (2020–1990); city centres are denoted as black dots. b) Quantile-quantile plots for the radial population distributions $\rho(s, t_i)$ and $\rho(s, t_j)$ (coloured curves); urban expansion factors Φ_{ij} from t_i to t_j are the estimated slopes (black lines). c) Radial population distribution $\rho(r)$ at remoteness distance r from the city centre. d) Radial population density $\sigma(r)$ at remoteness distance r from the city centre. e) Average population density $\bar{\sigma}(r)$ within disks of remoteness r with the same centre as the city. f) Urban expansion factors and their inter quartile range from the Theil-Sen estimation.

Coatzacoalcos, 30.1.01

a)

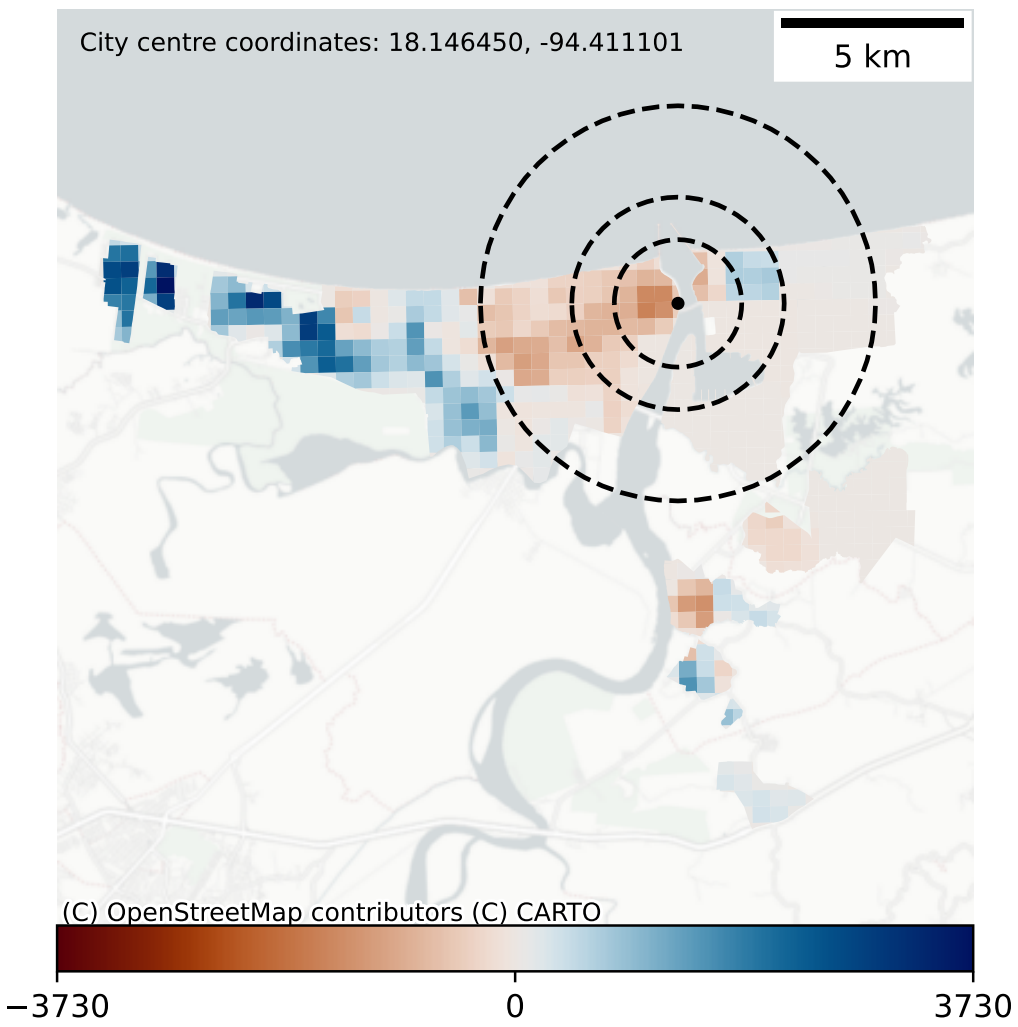

b)

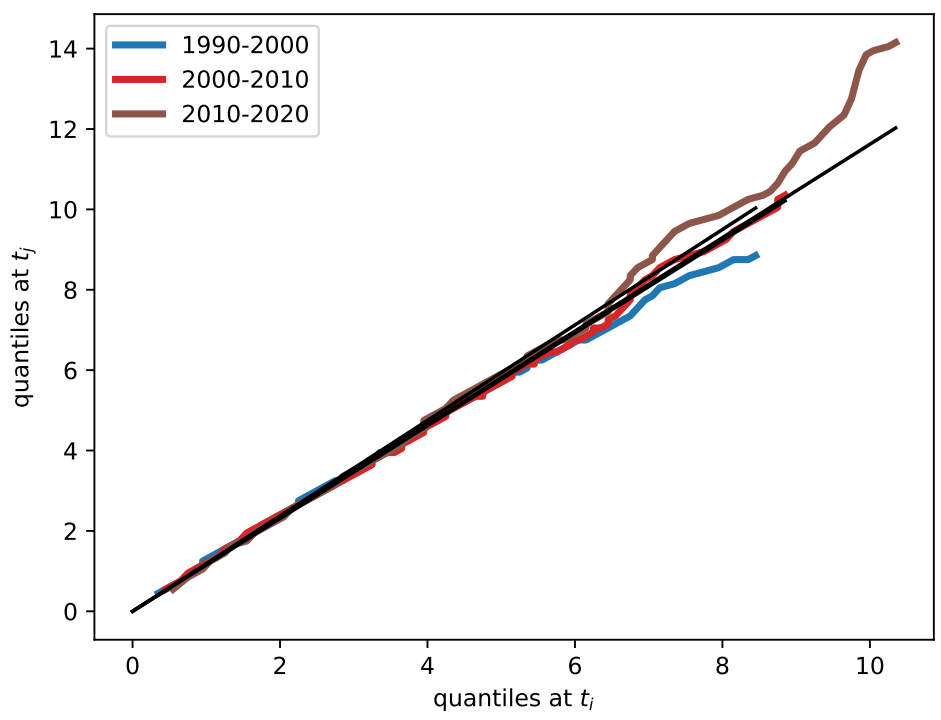

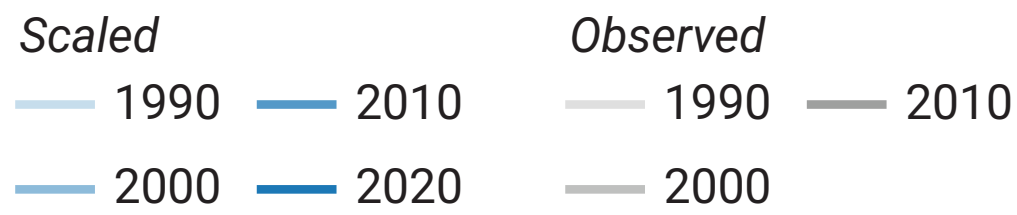

c)

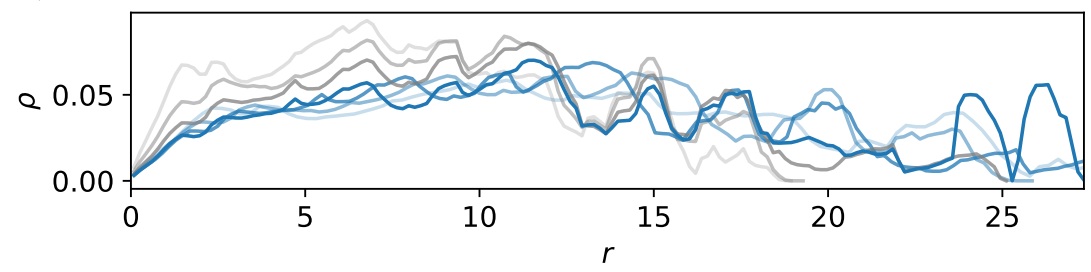

d)

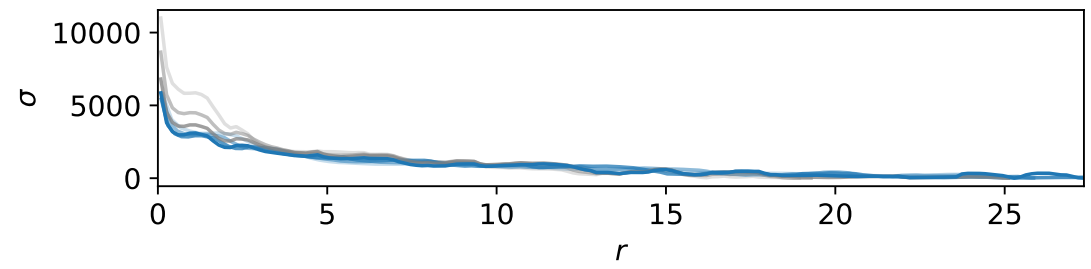

e)

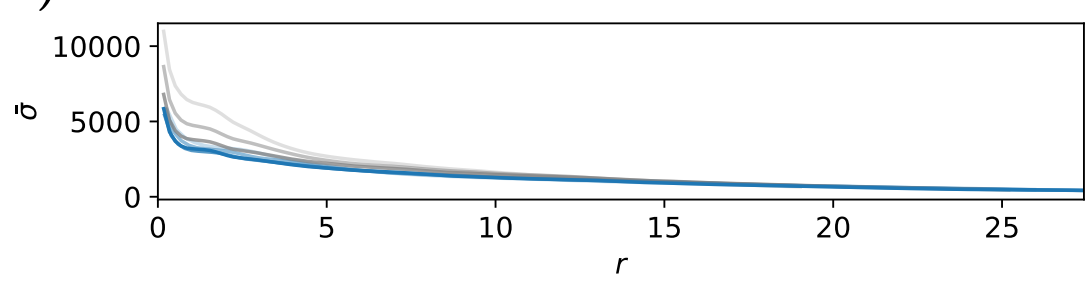

f)

Period (t_i-t_j)	$\frac{P(t_j)}{P(t_i)}$	Φ_{ij}	IQR
1990-2000	1.11	1.19	(1.17, 1.22)
2000-2010	1.12	1.15	(1.14, 1.21)
2010-2020	1.08	1.16	(1.15, 1.19)
1990-2010	1.24	1.38	(1.33, 1.46)
2000-2020	1.21	1.36	(1.33, 1.40)
1990-2020	1.34	1.60	(1.56, 1.68)

Supplementary Figure 66: Supplementary data for the metropolitan zone of Coatzacoalcos with code 30.1.01. Remoteness values are those of 2020. a) Population difference by grid cell (2020–1990); city centres are denoted as black dots. b) Quantile-quantile plots for the radial population distributions $\rho(s, t_i)$ and $\rho(s, t_j)$ (coloured curves); urban expansion factors Φ_{ij} from t_i to t_j are the estimated slopes (black lines). c) Radial population distribution $\rho(r)$ at remoteness distance r from the city centre. d) Radial population density $\sigma(r)$ at remoteness distance r from the city centre. e) Average population density $\bar{\sigma}(r)$ within disks of remoteness r with the same centre as the city. f) Urban expansion factors and their inter quartile range from the Theil-Sen estimation.

Córdoba, 30.1.02

a)

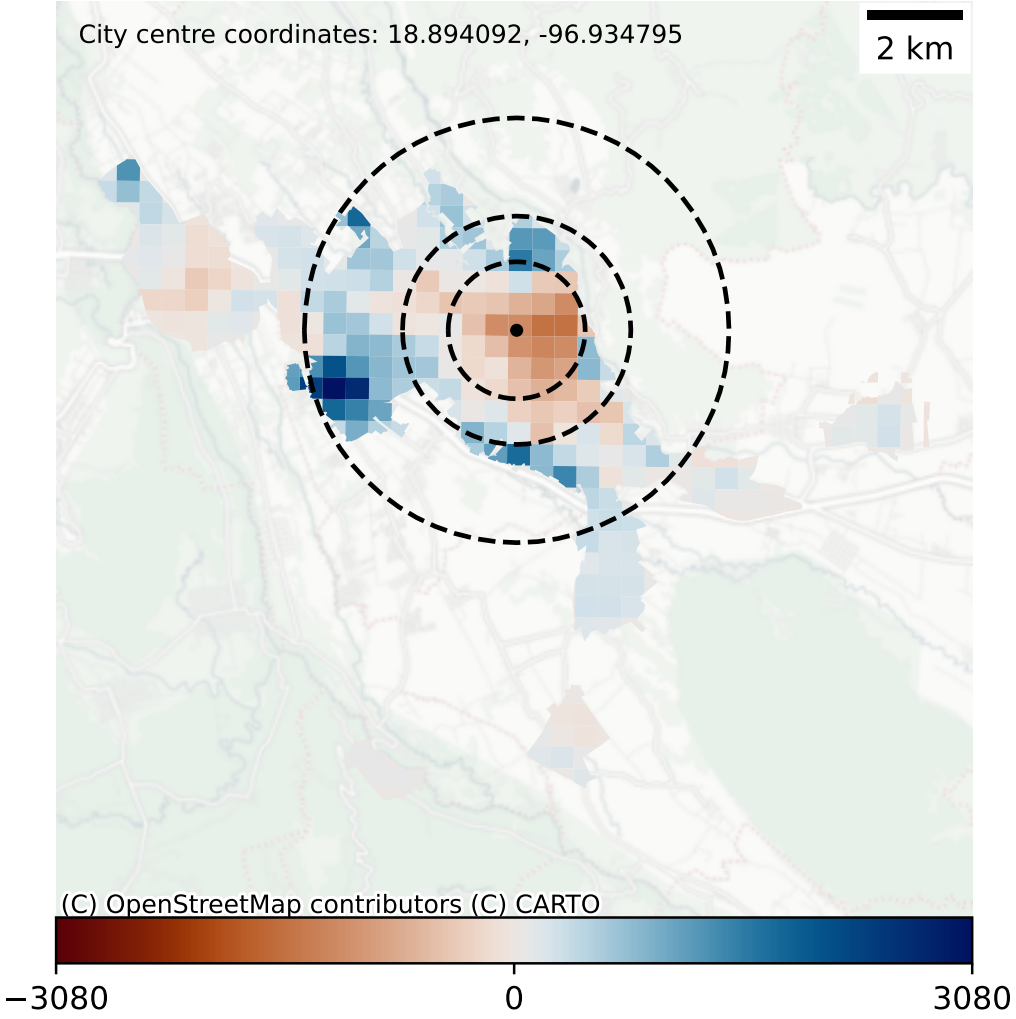

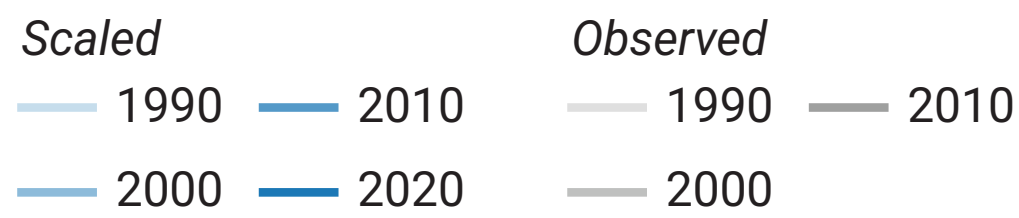

c)

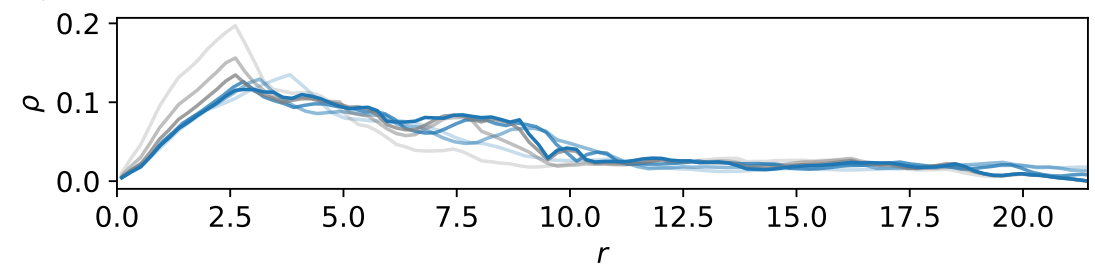

d)

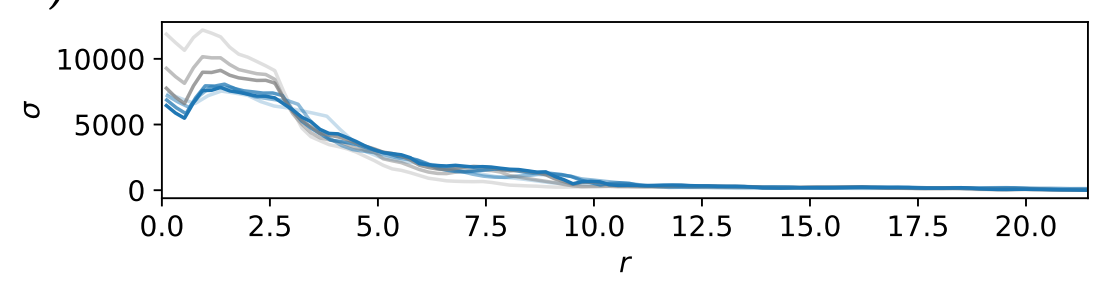

e)

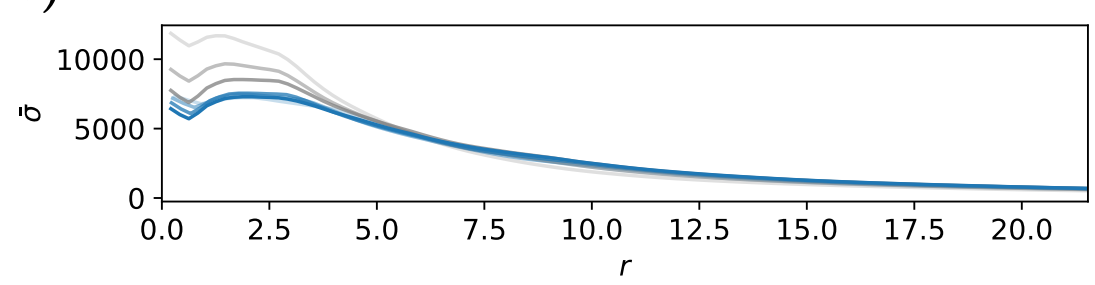

b)

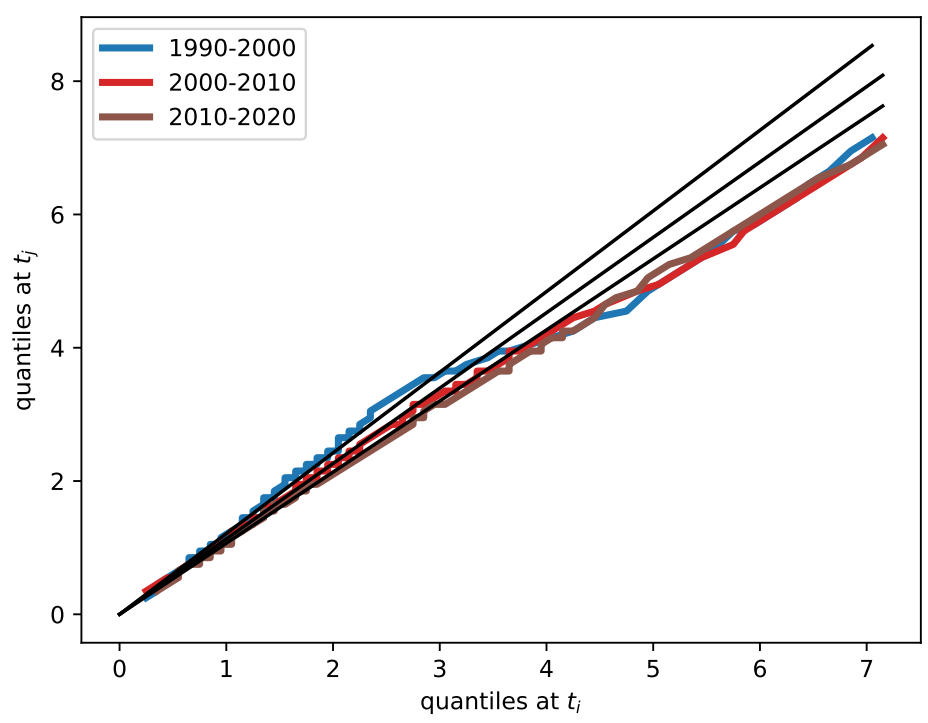

f)

Period (t_i-t_j)	$\frac{P(t_j)}{P(t_i)}$	Φ_{ij}	IQR
1990-2000	1.17	1.21	(1.17, 1.24)
2000-2010	1.12	1.13	(1.11, 1.16)
2010-2020	1.01	1.07	(1.05, 1.10)
1990-2010	1.31	1.36	(1.32, 1.41)
2000-2020	1.13	1.21	(1.18, 1.24)
1990-2020	1.32	1.46	(1.42, 1.51)

Supplementary Figure 67: Supplementary data for the metropolitan zone of Córdoba with code 30.1.02. Remoteness values are those of 2020. a) Population difference by grid cell (2020–1990); city centres are denoted as black dots. b) Quantile-quantile plots for the radial population distributions $\rho(s, t_i)$ and $\rho(s, t_j)$ (coloured curves); urban expansion factors Φ_{ij} from t_i to t_j are the estimated slopes (black lines). c) Radial population distribution $\rho(r)$ at remoteness distance r from the city centre. d) Radial population density $\sigma(r)$ at remoteness distance r from the city centre. e) Average population density $\bar{\sigma}(r)$ within disks of remoteness r with the same centre as the city. f) Urban expansion factors and their inter quartile range from the Theil-Sen estimation.

Minatitlán, 30.1.03

a)

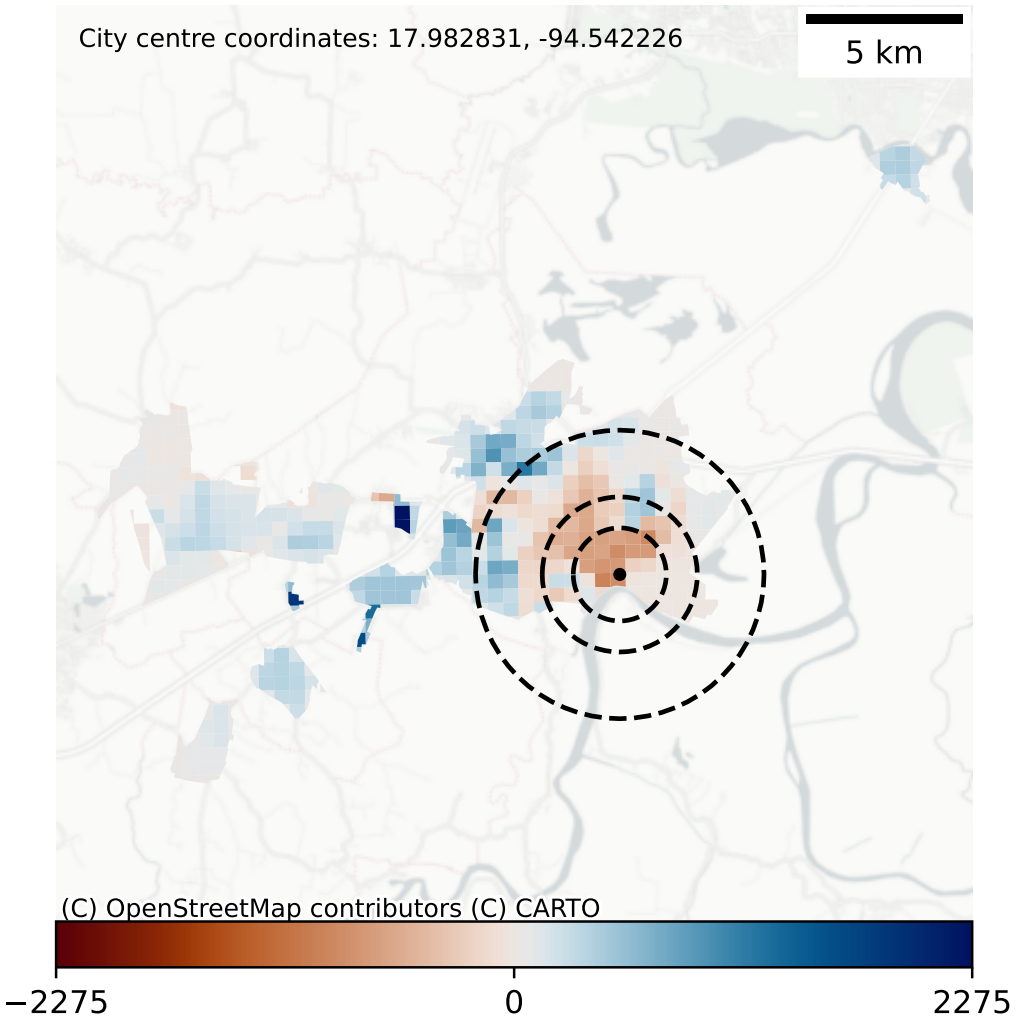

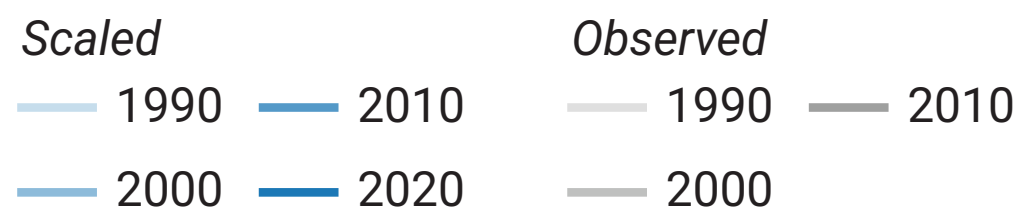

c)

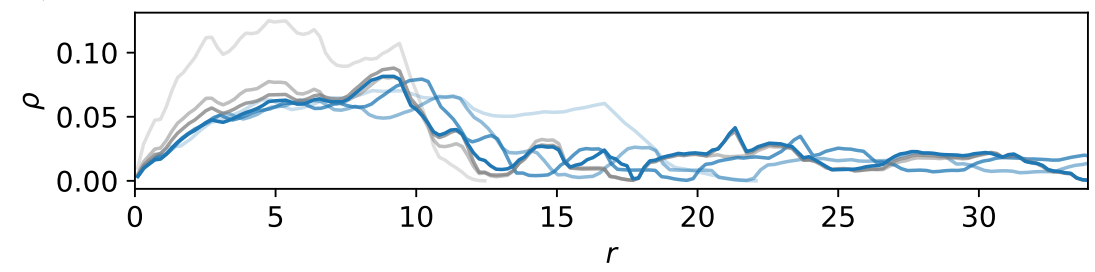

d)

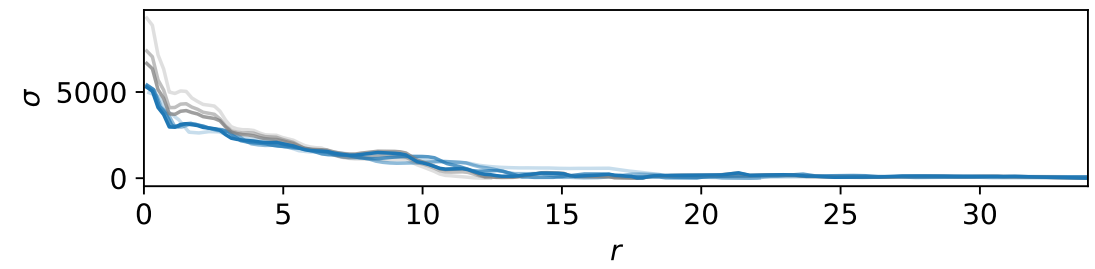

e)

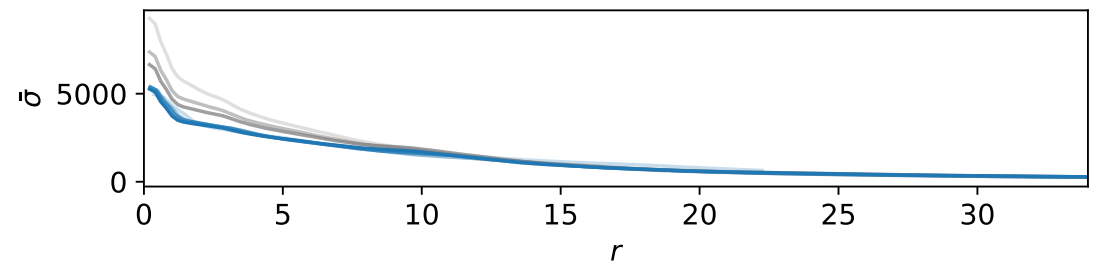

b)

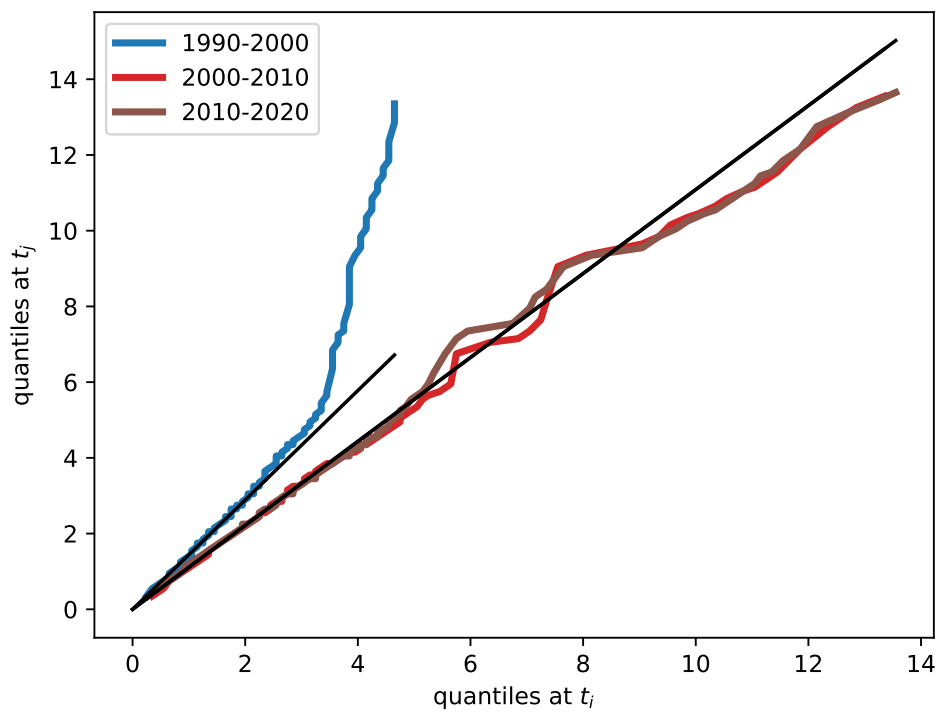

f)

Period (t_i-t_j)	$\frac{P(t_j)}{P(t_i)}$	Φ_{ij}	IQR
1990-2000	1.54	1.44	(1.41, 1.48)
2000-2010	1.10	1.11	(1.09, 1.13)
2010-2020	1.00	1.11	(1.09, 1.13)
1990-2010	1.69	1.61	(1.57, 1.64)
2000-2020	1.09	1.23	(1.21, 1.26)
1990-2020	1.68	1.78	(1.75, 1.81)

Supplementary Figure 68: Supplementary data for the metropolitan zone of Minatitlán with code 30.1.03. Remoteness values are those of 2020. a) Population difference by grid cell (2020–1990); city centres are denoted as black dots. b) Quantile-quantile plots for the radial population distributions $\rho(s, t_i)$ and $\rho(s, t_j)$ (coloured curves); urban expansion factors Φ_{ij} from t_i to t_j are the estimated slopes (black lines). c) Radial population distribution $\rho(r)$ at remoteness distance r from the city centre. d) Radial population density $\sigma(r)$ at remoteness distance r from the city centre. e) Average population density $\bar{\sigma}(r)$ within disks of remoteness r with the same centre as the city. f) Urban expansion factors and their inter quartile range from the Theil-Sen estimation.

Orizaba, 30.1.04

a)

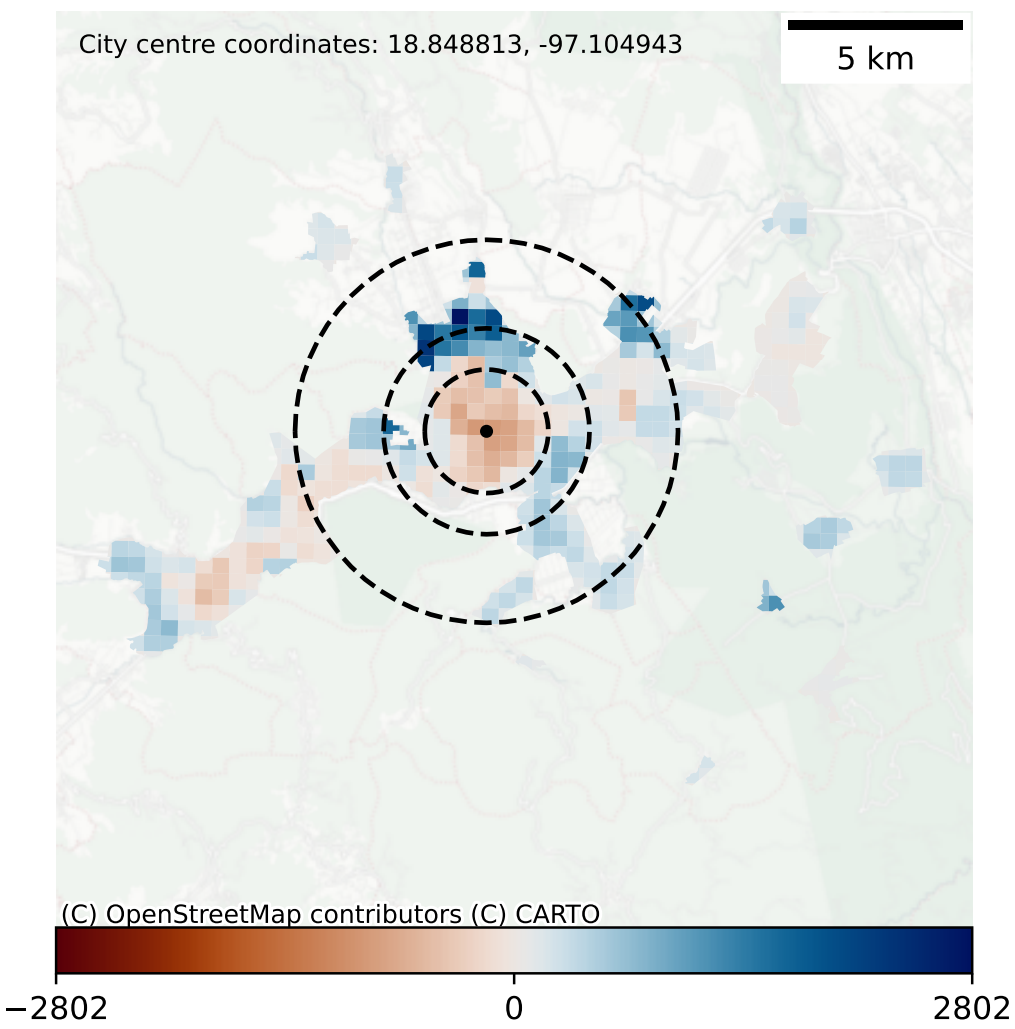

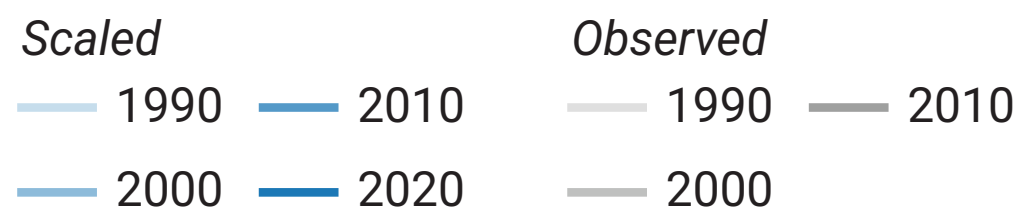

c)

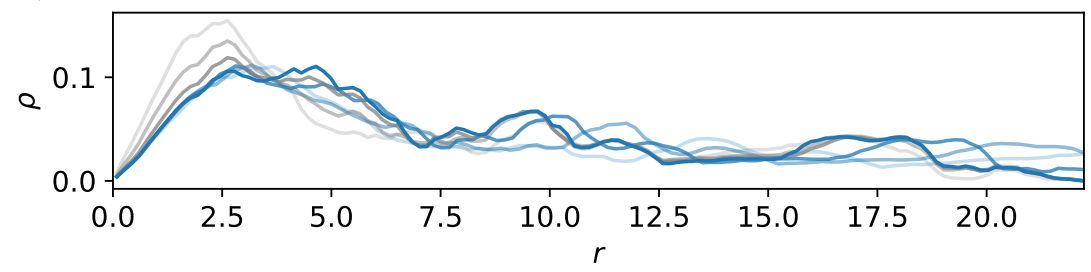

d)

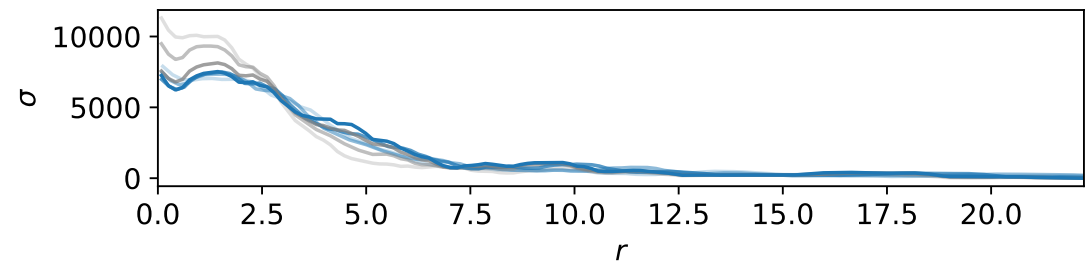

e)

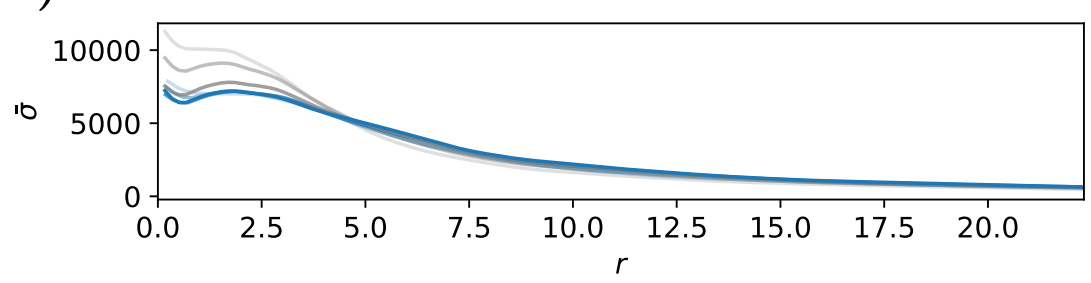

b)

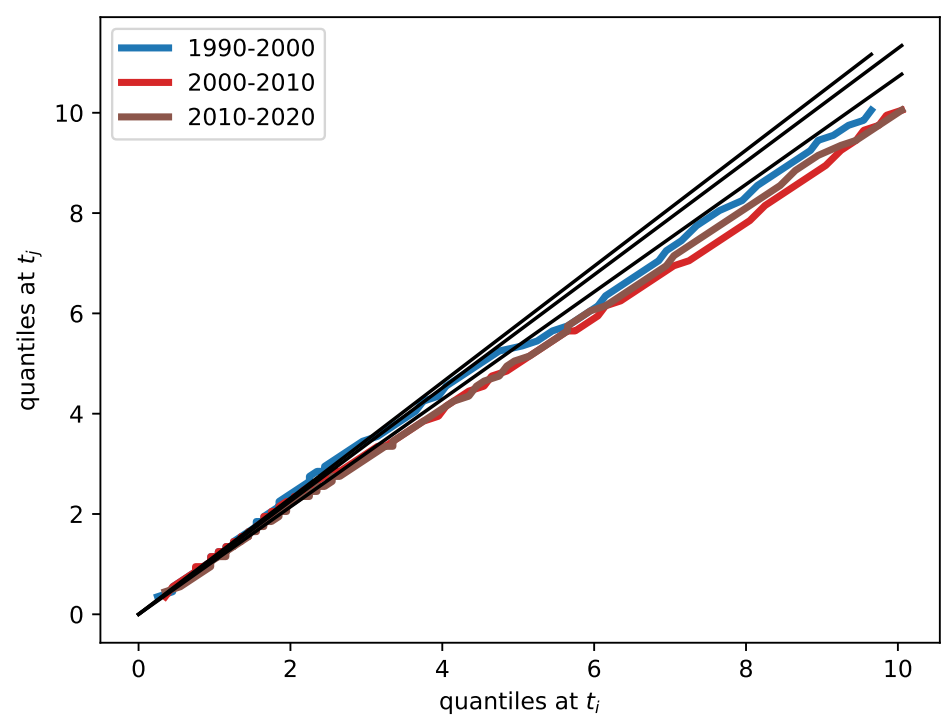

f)

Period (t_i-t_j)	$\frac{P(t_j)}{P(t_i)}$	Φ_{ij}	IQR
1990-2000	1.19	1.16	(1.13, 1.18)
2000-2010	1.09	1.13	(1.10, 1.16)
2010-2020	1.06	1.07	(1.04, 1.09)
1990-2010	1.29	1.31	(1.27, 1.34)
2000-2020	1.15	1.21	(1.18, 1.23)
1990-2020	1.37	1.40	(1.37, 1.42)

Supplementary Figure 69: Supplementary data for the metropolitan zone of Orizaba with code 30.1.04. Remoteness values are those of 2020. a) Population difference by grid cell (2020–1990); city centres are denoted as black dots. b) Quantile-quantile plots for the radial population distributions $\rho(s, t_i)$ and $\rho(s, t_j)$ (coloured curves); urban expansion factors Φ_{ij} from t_i to t_j are the estimated slopes (black lines). c) Radial population distribution $\rho(r)$ at remoteness distance r from the city centre. d) Radial population density $\sigma(r)$ at remoteness distance r from the city centre. e) Average population density $\bar{\sigma}(r)$ within disks of remoteness r with the same centre as the city. f) Urban expansion factors and their inter quartile range from the Theil-Sen estimation.

Poza Rica, 30.1.05

a)

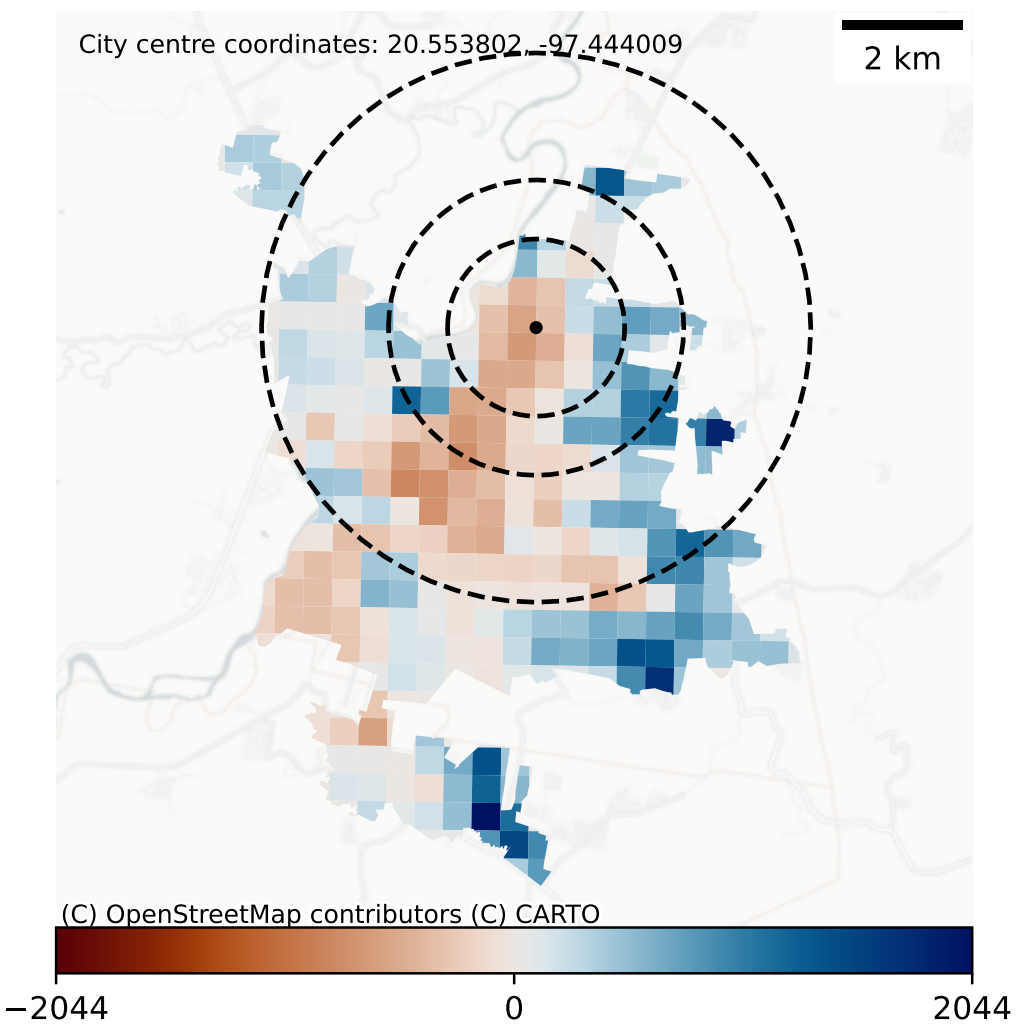

b)

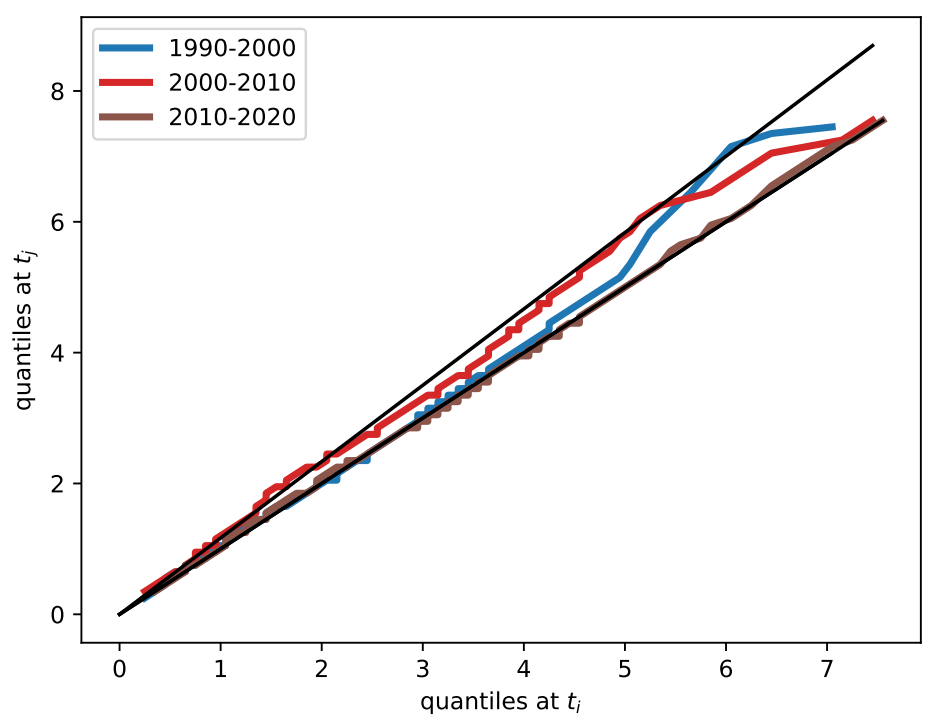

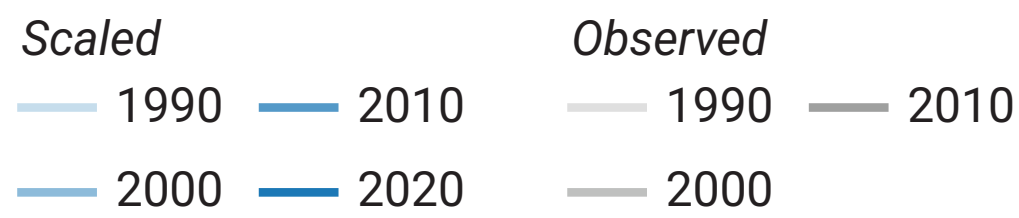

c)

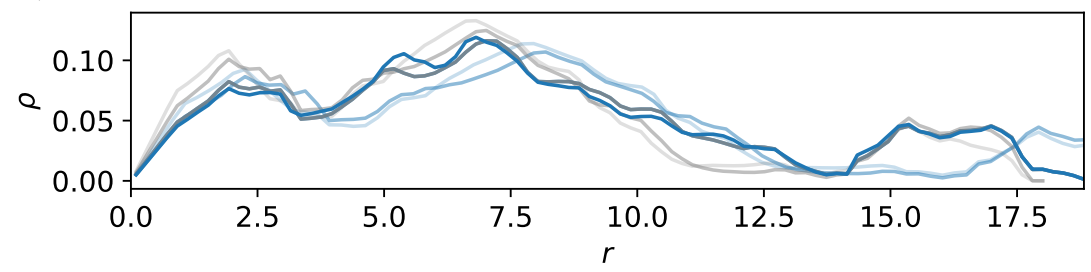

d)

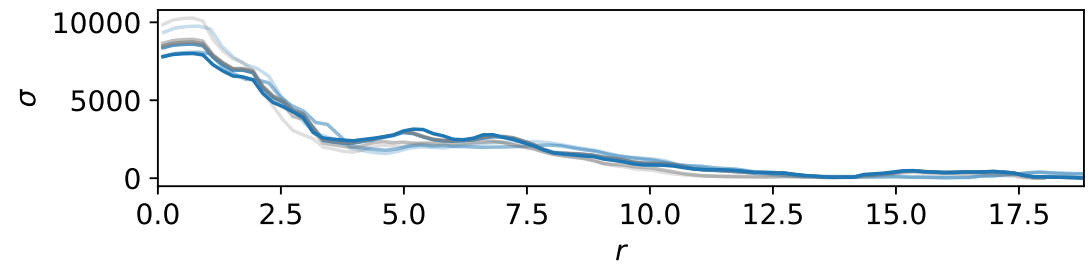

e)

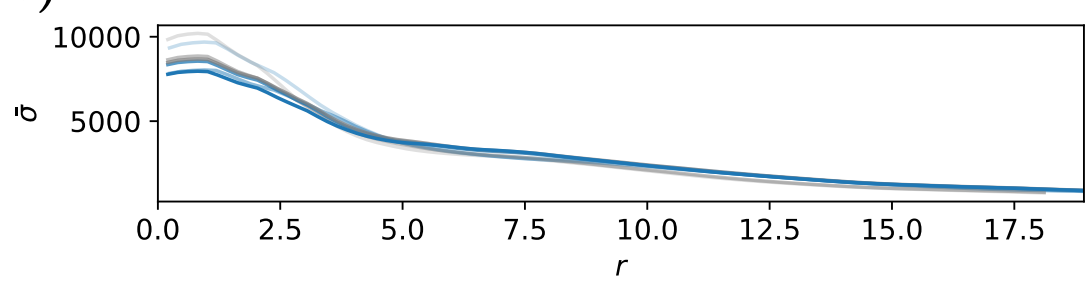

f)

Period (t_i-t_j)	$\frac{P(t_j)}{P(t_i)}$	Φ_{ij}	IQR
1990-2000	1.05	1.00	(1.00, 1.08)
2000-2010	1.25	1.17	(1.13, 1.22)
2010-2020	0.99	1.00	(1.00, 1.05)
1990-2010	1.31	1.23	(1.14, 1.27)
2000-2020	1.23	1.21	(1.13, 1.27)
1990-2020	1.29	1.27	(1.14, 1.32)

Supplementary Figure 70: Supplementary data for the metropolitan zone of Poza Rica with code 30.1.05. Remoteness values are those of 2020. a) Population difference by grid cell (2020–1990); city centres are denoted as black dots. b) Quantile-quantile plots for the radial population distributions $\rho(s, t_i)$ and $\rho(s, t_j)$ (coloured curves); urban expansion factors Φ_{ij} from t_i to t_j are the estimated slopes (black lines). c) Radial population distribution $\rho(r)$ at remoteness distance r from the city centre. d) Radial population density $\sigma(r)$ at remoteness distance r from the city centre. e) Average population density $\bar{\sigma}(r)$ within disks of remoteness r with the same centre as the city. f) Urban expansion factors and their inter quartile range from the Theil-Sen estimation.

Veracruz, 30.1.06

a)

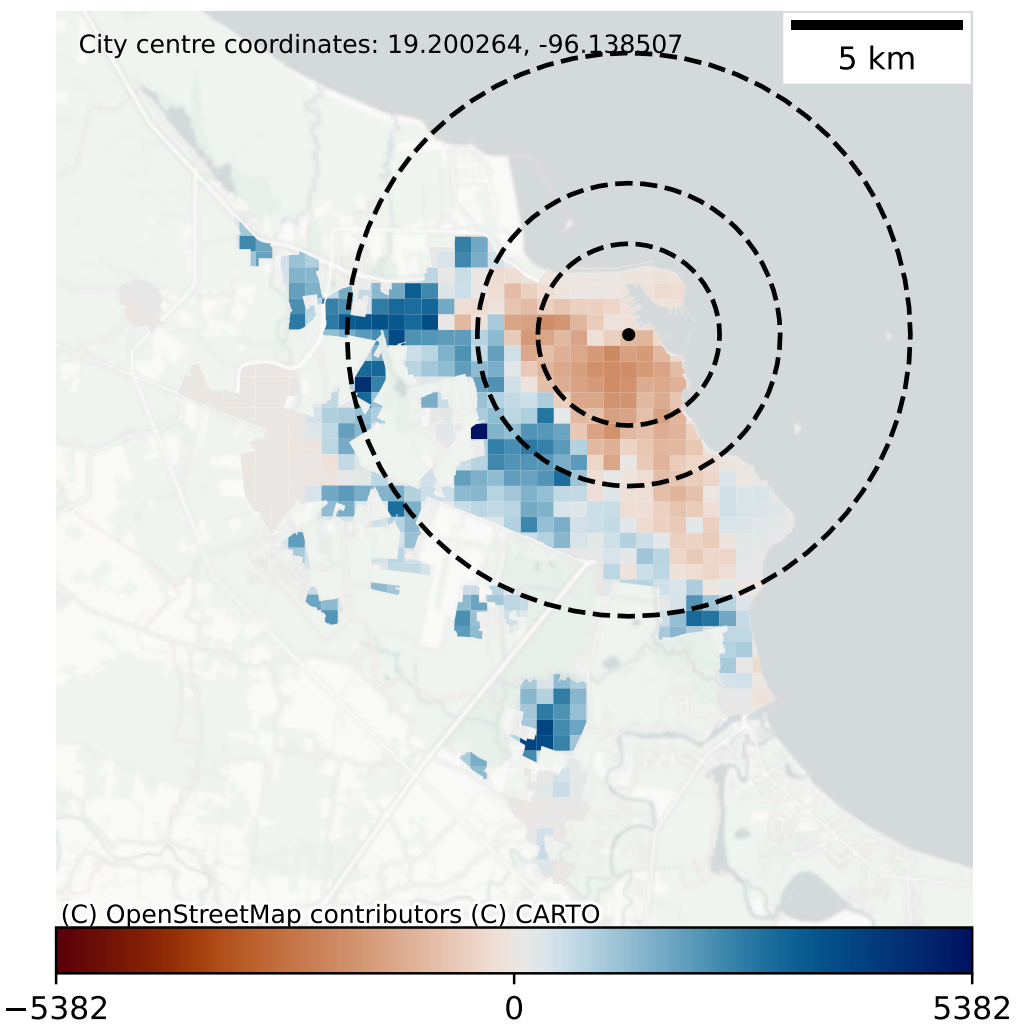

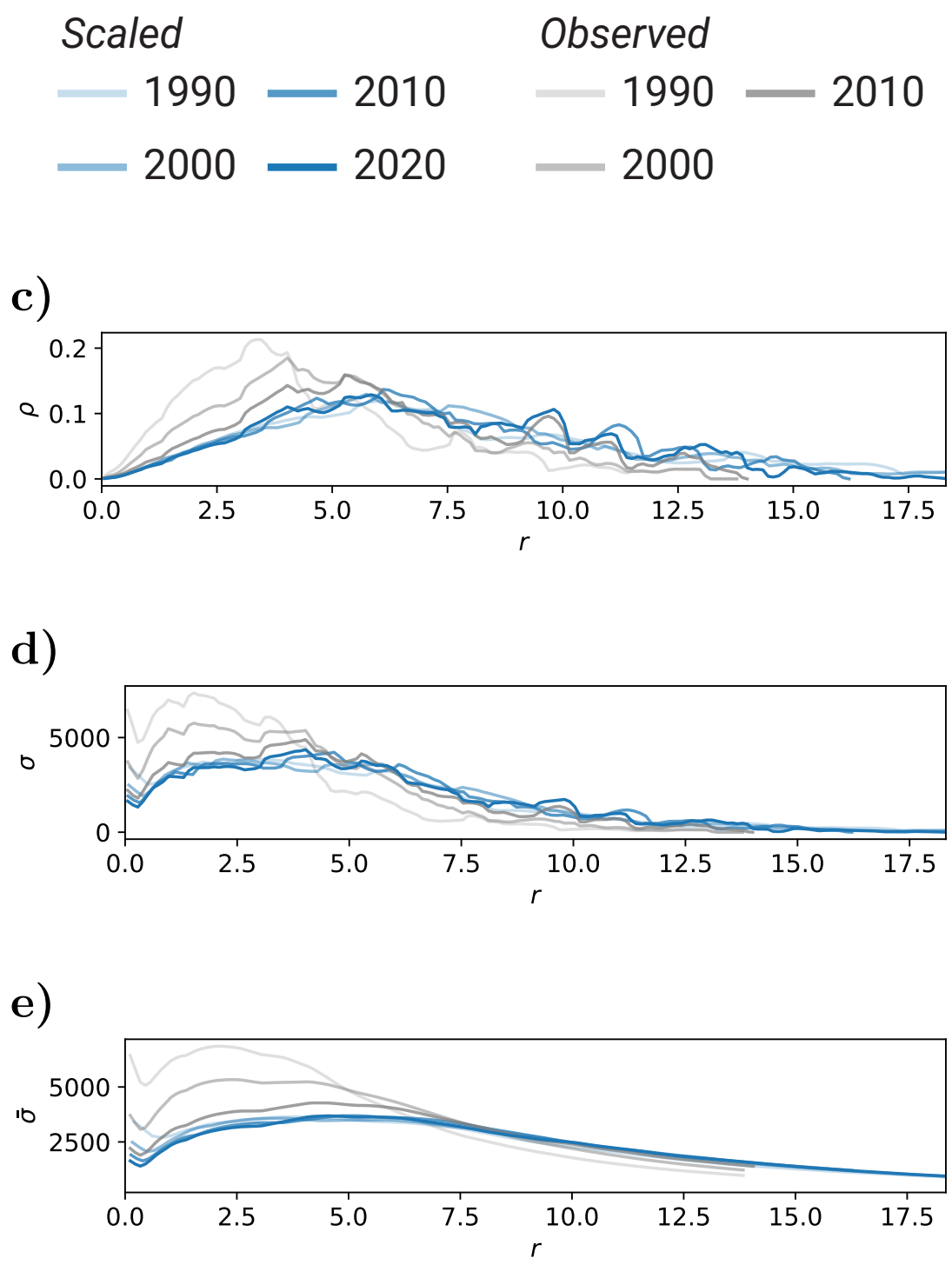

b)

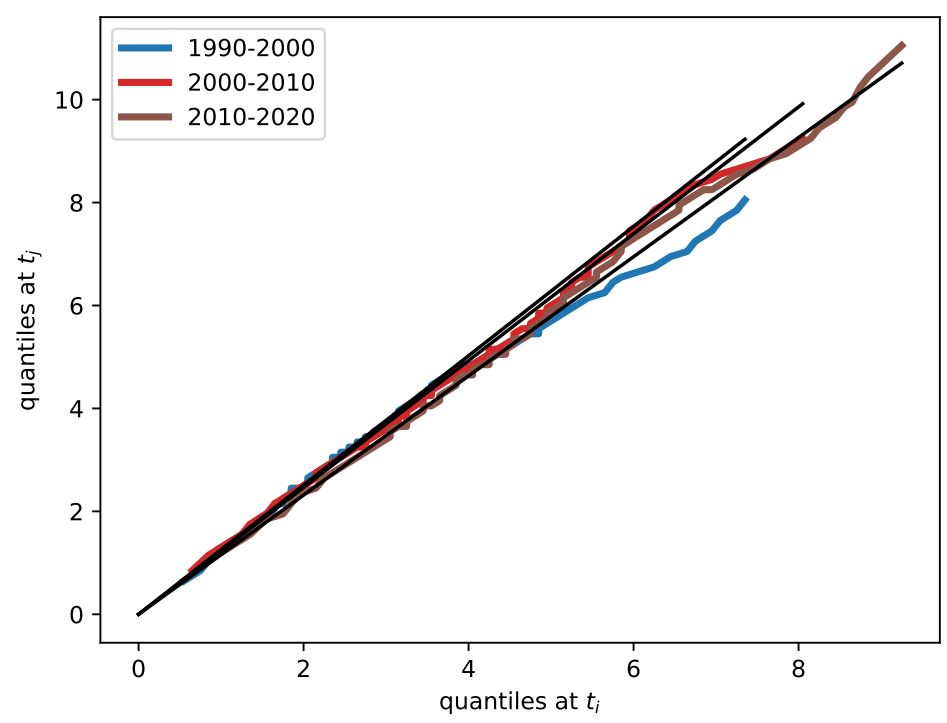

f)

Period (t_i-t_j)	$\frac{P(t_j)}{P(t_i)}$	Φ_{ij}	IQR
1990-2000	1.25	1.26	(1.24, 1.28)
2000-2010	1.18	1.23	(1.21, 1.26)
2010-2020	1.16	1.16	(1.14, 1.17)
1990-2010	1.47	1.55	(1.51, 1.59)
2000-2020	1.36	1.42	(1.40, 1.46)
1990-2020	1.71	1.79	(1.75, 1.84)

Supplementary Figure 71: Supplementary data for the metropolitan zone of Veracruz with code 30.1.06. Remoteness values are those of 2020. a) Population difference by grid cell (2020–1990); city centres are denoted as black dots. b) Quantile-quantile plots for the radial population distributions $\rho(s, t_i)$ and $\rho(s, t_j)$ (coloured curves); urban expansion factors Φ_{ij} from t_i to t_j are the estimated slopes (black lines). c) Radial population distribution $\rho(r)$ at remoteness distance r from the city centre. d) Radial population density $\sigma(r)$ at remoteness distance r from the city centre. e) Average population density $\bar{\sigma}(r)$ within disks of remoteness r with the same centre as the city. f) Urban expansion factors and their inter quartile range from the Theil-Sen estimation.

Xalapa, 30.1.07

a)

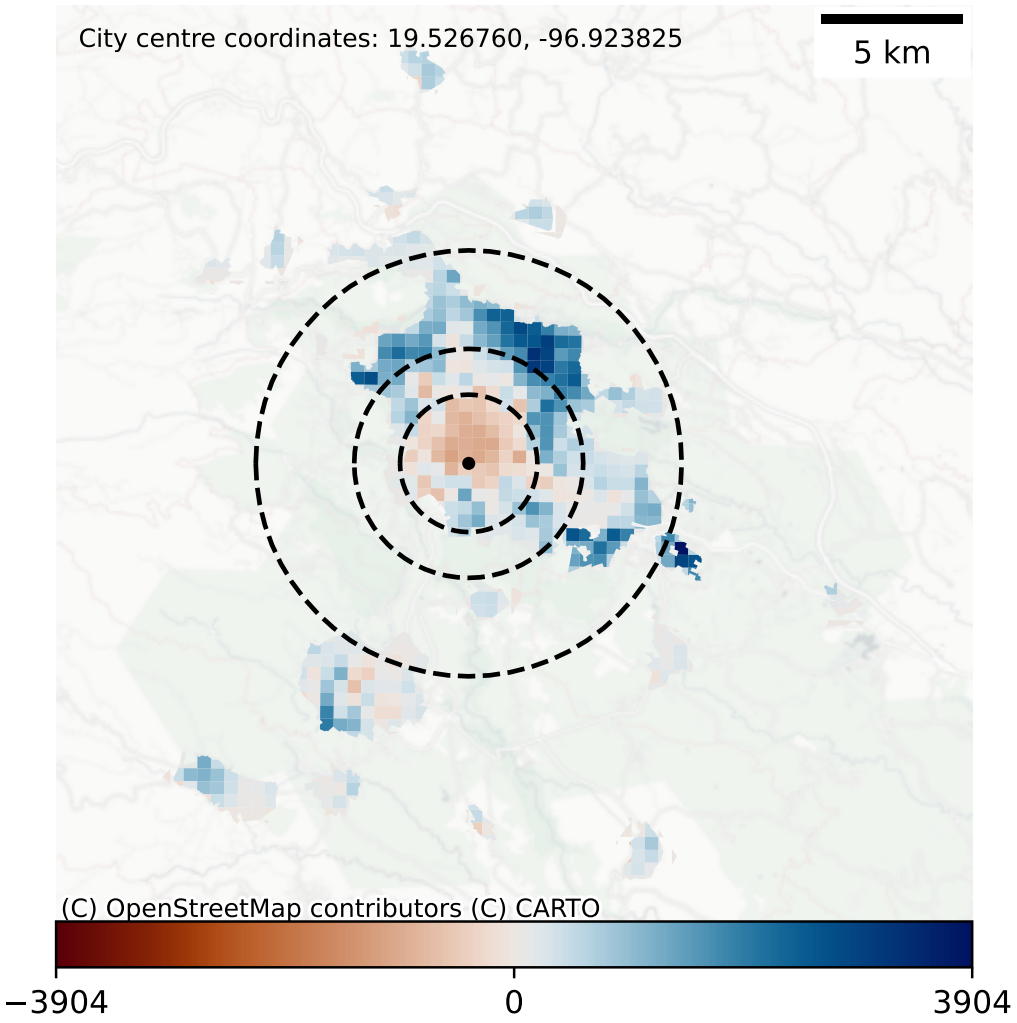

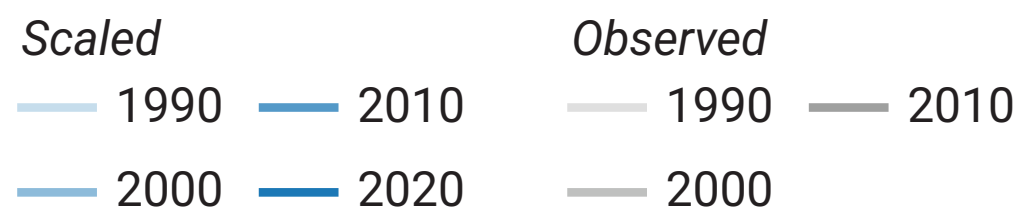

c)

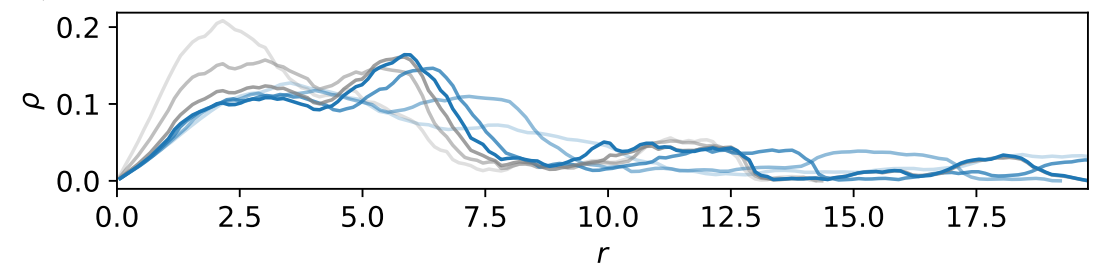

d)

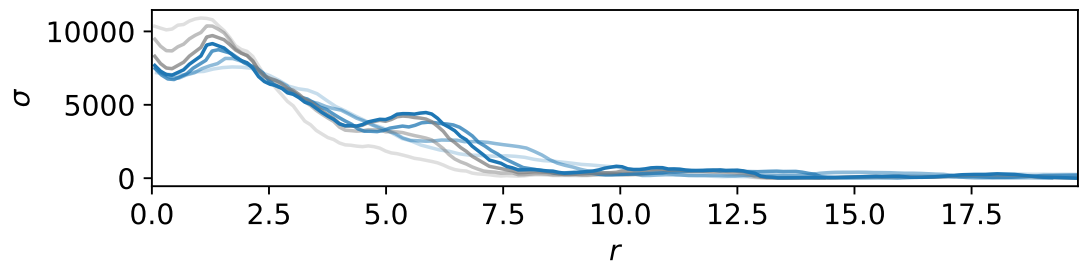

e)

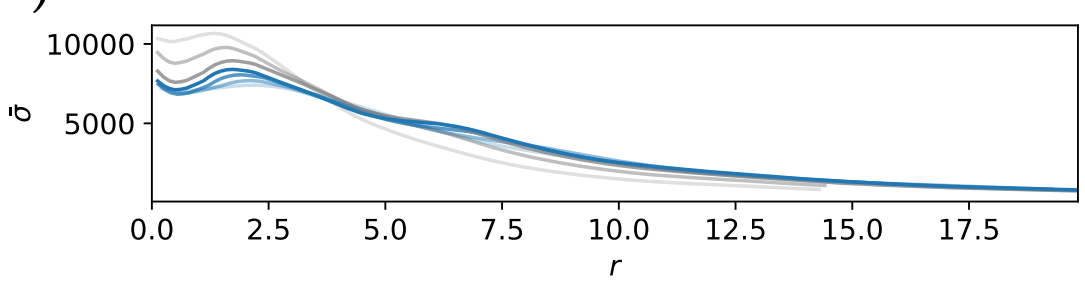

b)

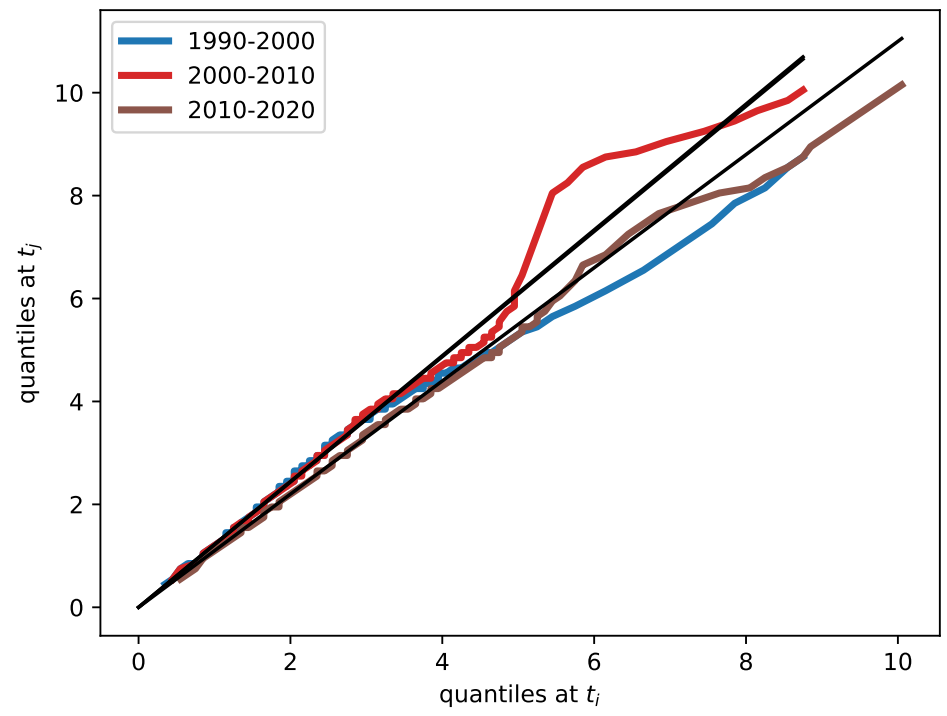

f)

Period (t_i-t_j)	$\frac{P(t_j)}{P(t_i)}$	Φ_{ij}	IQR
1990-2000	1.32	1.22	(1.20, 1.24)
2000-2010	1.29	1.22	(1.19, 1.23)
2010-2020	1.09	1.10	(1.08, 1.12)
1990-2010	1.71	1.48	(1.44, 1.52)
2000-2020	1.41	1.34	(1.30, 1.36)
1990-2020	1.86	1.62	(1.57, 1.68)

Supplementary Figure 72: Supplementary data for the metropolitan zone of Xalapa with code 30.1.07. Remoteness values are those of 2020. a) Population difference by grid cell (2020–1990); city centres are denoted as black dots. b) Quantile-quantile plots for the radial population distributions $\rho(s, t_i)$ and $\rho(s, t_j)$ (coloured curves); urban expansion factors Φ_{ij} from t_i to t_j are the estimated slopes (black lines). c) Radial population distribution $\rho(r)$ at remoteness distance r from the city centre. d) Radial population density $\sigma(r)$ at remoteness distance r from the city centre. e) Average population density $\bar{\sigma}(r)$ within disks of remoteness r with the same centre as the city. f) Urban expansion factors and their inter quartile range from the Theil-Sen estimation.

Mérida, 31.1.01

a)

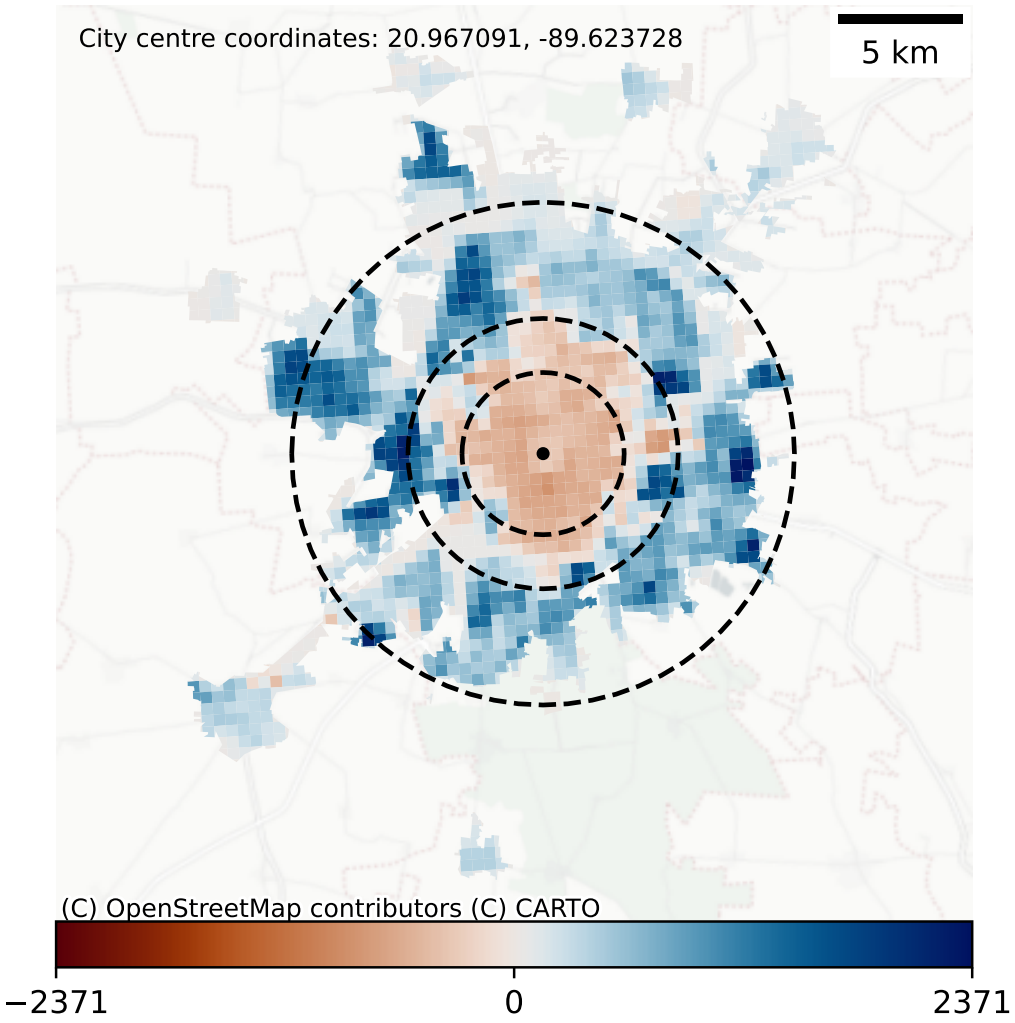

b)

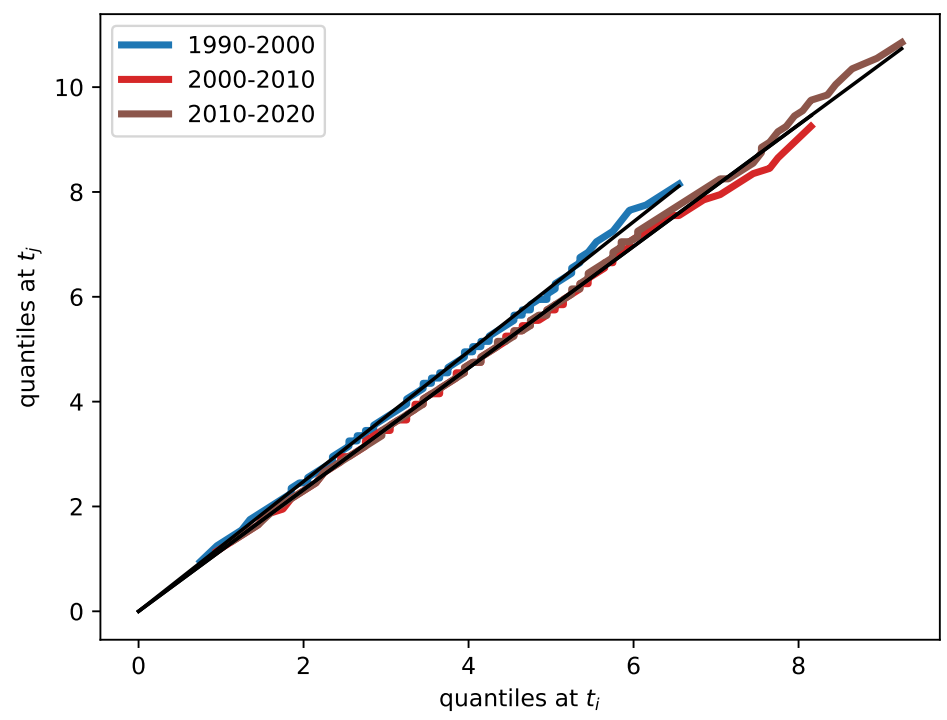

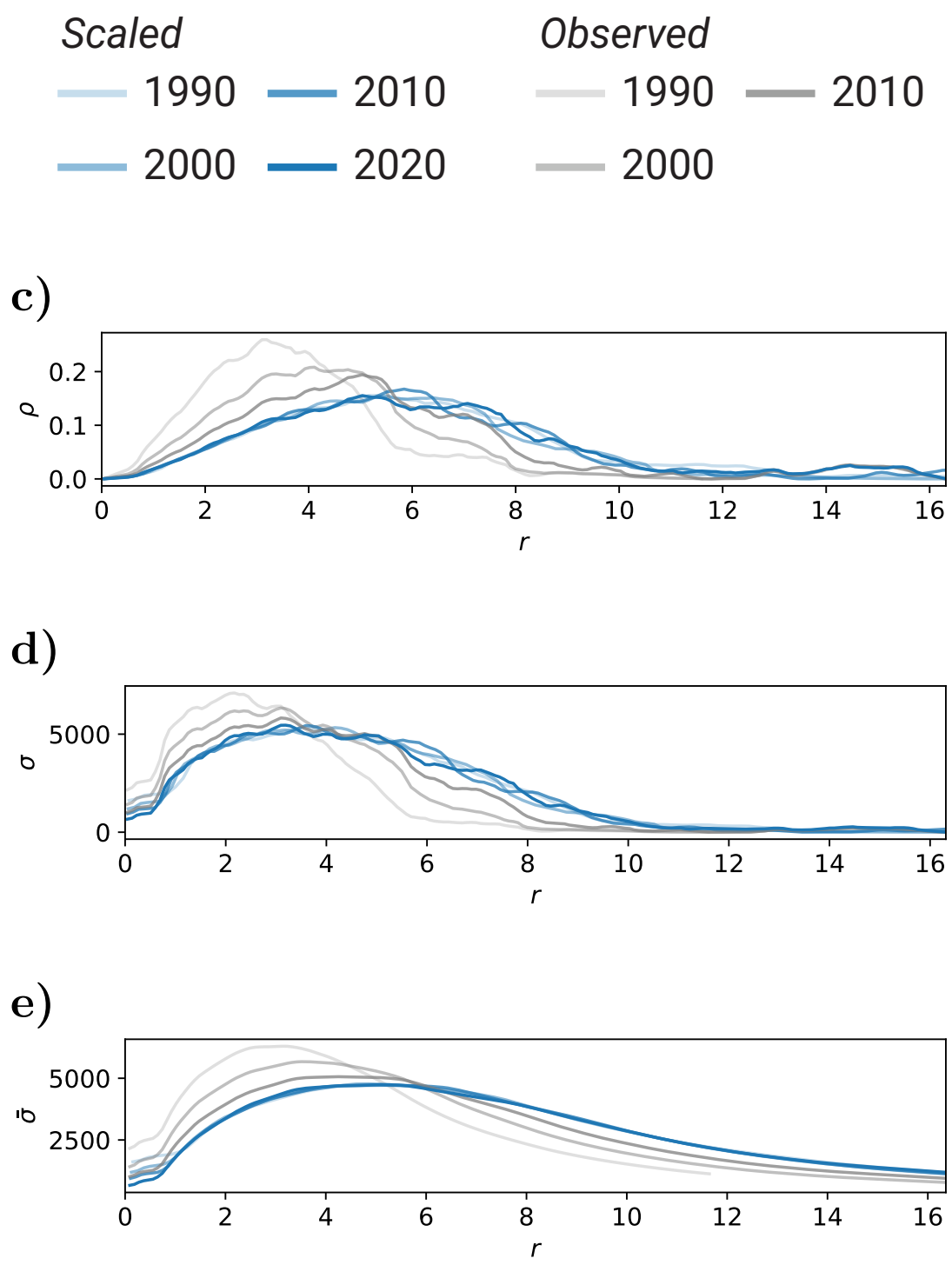

f)

Period (t_i-t_j)	$\frac{P(t_j)}{P(t_i)}$	Φ_{ij}	IQR
1990-2000	1.36	1.24	(1.22, 1.26)
2000-2010	1.21	1.16	(1.15, 1.17)
2010-2020	1.26	1.16	(1.15, 1.17)
1990-2010	1.65	1.44	(1.42, 1.45)
2000-2020	1.53	1.35	(1.33, 1.36)
1990-2020	2.08	1.67	(1.65, 1.69)

Supplementary Figure 73: Supplementary data for the metropolitan zone of Mérida with code 31.1.01. Remoteness values are those of 2020. a) Population difference by grid cell (2020–1990); city centres are denoted as black dots. b) Quantile-quantile plots for the radial population distributions $\rho(s, t_i)$ and $\rho(s, t_j)$ (coloured curves); urban expansion factors Φ_{ij} from t_i to t_j are the estimated slopes (black lines). c) Radial population distribution $\rho(r)$ at remoteness distance r from the city centre. d) Radial population density $\sigma(r)$ at remoteness distance r from the city centre. e) Average population density $\bar{\sigma}(r)$ within disks of remoteness r with the same centre as the city. f) Urban expansion factors and their inter quartile range from the Theil-Sen estimation.

Zacatecas-Guadalupe, 32.1.01

a)

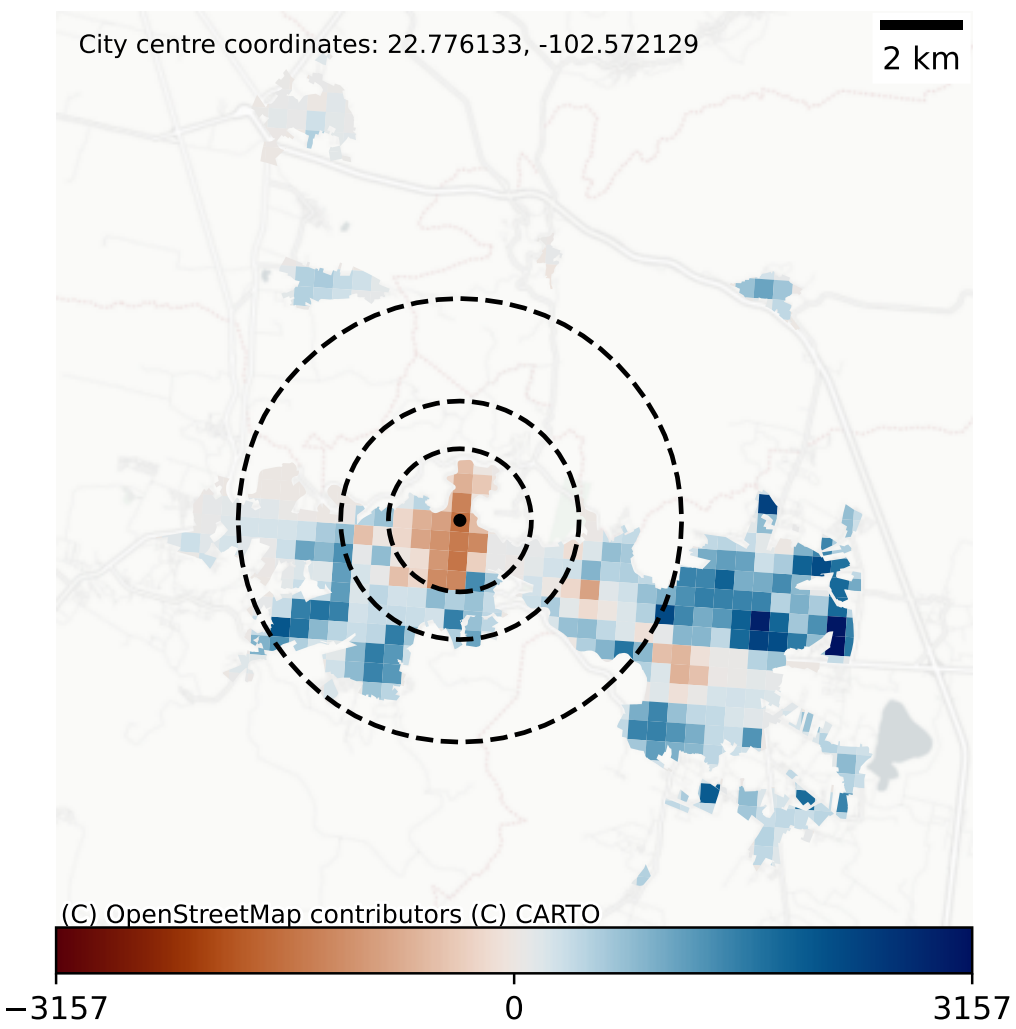

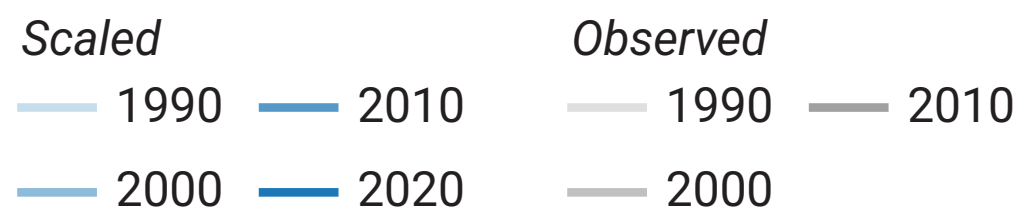

c)

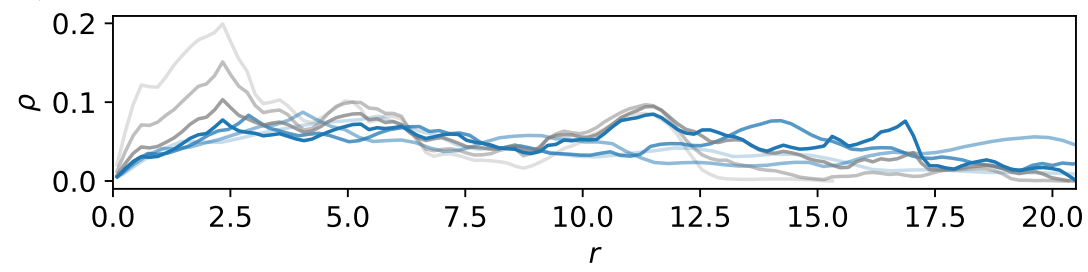

d)

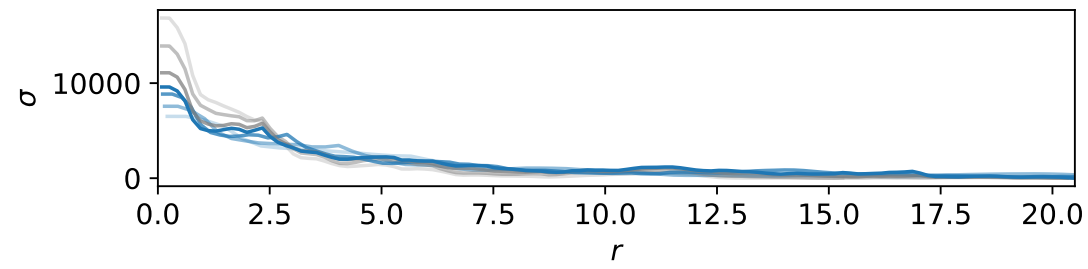

b)

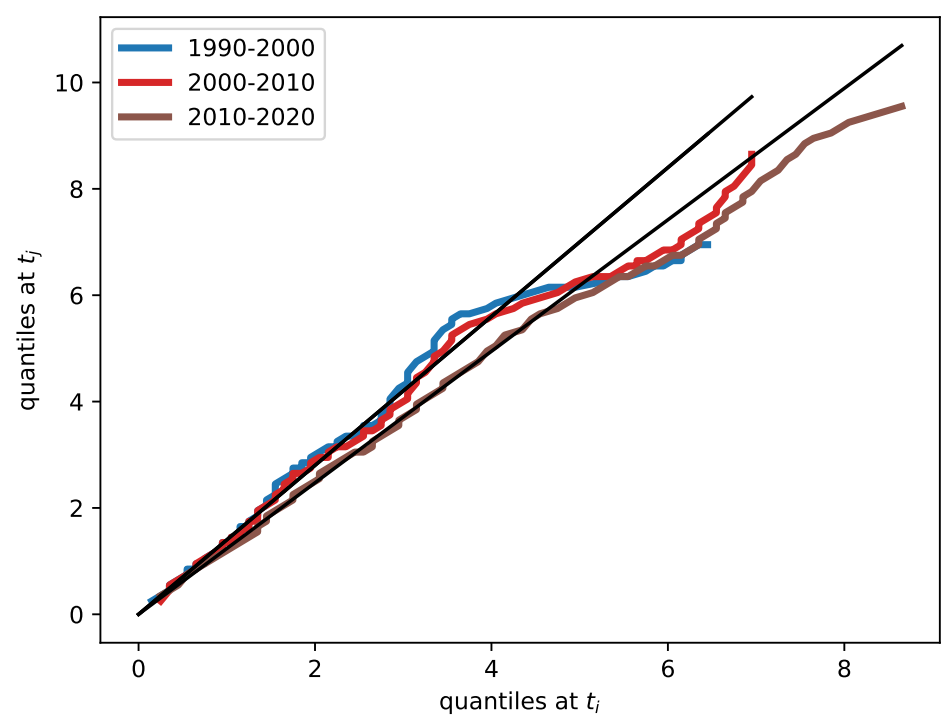

e)

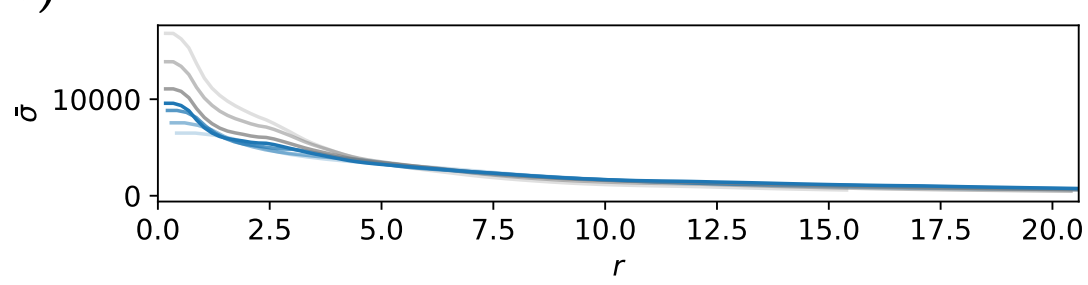

f)

Period (t_i-t_j)	$\frac{P(t_j)}{P(t_i)}$	Φ_{ij}	IQR
1990-2000	1.39	1.40	(1.35, 1.44)
2000-2010	1.34	1.40	(1.34, 1.44)
2010-2020	1.22	1.24	(1.21, 1.26)
1990-2010	1.86	1.94	(1.83, 2.04)
2000-2020	1.63	1.72	(1.64, 1.77)
1990-2020	2.27	2.42	(2.28, 2.51)

Supplementary Figure 74: Supplementary data for the metropolitan zone of Zacatecas-Guadalupe with code 32.1.01. Remoteness values are those of 2020. a) Population difference by grid cell (2020–1990); city centres are denoted as black dots. b) Quantile-quantile plots for the radial population distributions $\rho(s, t_i)$ and $\rho(s, t_j)$ (coloured curves); urban expansion factors Φ_{ij} from t_i to t_j are the estimated slopes (black lines). c) Radial population distribution $\rho(r)$ at remoteness distance r from the city centre. d) Radial population density $\sigma(r)$ at remoteness distance r from the city centre. e) Average population density $\bar{\sigma}(r)$ within disks of remoteness r with the same centre as the city. f) Urban expansion factors and their inter quartile range from the Theil-Sen estimation.

Supplementary References

1. Lemoy, R. & Caruso, G. Radial analysis and scaling of urban land use. *Scientific Reports* **11,** 22044 (2021).
2. Lemoy, R. & Caruso, G. Evidence for the homothetic scaling of urban forms. *Environment and Planning B: Urban Analytics and City Science* **47,** 870–888 (2020).
3. Prieto-Curiel, R., Luengas-Sierra, P. & Borja-Vega, C. Urban sprawl is associated with reduced access and increased costs of water and sanitation. *Nature Cities* **2,** 1148–1159 (2025).
4. Prieto-Curiel, R., Patino, J. E. & Anderson, B. Scaling of the morphology of African cities. *Proceedings of the National Academy of Sciences* **120,** e2214254120 (2023).
5. Bettencourt, L. M. A. & Lobo, J. Urban scaling in Europe. *Journal of The Royal Society Interface* **13.** Publisher: Royal Society, 20160005. `https://royalsocietypublishing.org/doi/10.1098/rsif.2016.0005` (2025) (Mar. 2016).
6. Bettencourt, L. M. The origins of scaling in cities. *Science* **340,** 1438–1441 (2013).
7. Lobo, J., Ortman, S. & Bettencourt, L. *Systematic Relationship Between Areal Extent and Population for Urban Areas in the USA* ISSN: 2693-5015. Dec. 2024. `https://www.researchsquare.com/article/rs-5308091/v1` (2025).
8. Laziou, G. & Lemoy, R. The three-dimensional structure of population density in world cities. *npj Urban Sustainability* **5,** 75 (2025).
9. Barreto, S. M. *et al.* Epidemiology in Latin America and the Caribbean: current situation and challenges. *International Journal of Epidemiology* **41,** 557–571 (2012).
10. Mitra, A. K. & Rodriguez-Fernandez, G. Latin America and the Caribbean: assessment of the advances in public health for the achievement of the Millennium Development Goals. *Int J Environ Res Public Health* **7,** 2238–2255 (2010).
11. Brea, J. *Population Dynamics in Latin America* **1** (Population Reference Bureau., 2003).
12. World Bank. *Fertility rate, total (births per woman) - Mexico* World Population Prospects, (UN). 2024. `https://data.worldbank.org/indicator/SP.DYN.TFRT.IN?locations=MX`.
13. Galindo, C. & Ordorica, M. Estimación de nacimientos ocurridos y registrados, México 1950-2000. *Papeles de población* **13,** 39–86 (2007).
14. World Bank. *Life expectancy at birth, total (years) - Mexico* World Population Prospects, (UN). 2024. `https://data.worldbank.org/indicator/SP.DYN.LE00.IN?locations=MX`.
15. World Bank. *World Development Indicators - GDP per capita, PPP (current international $)* World Development Indicators (WDI). 2024. `https://data.worldbank.org/indicator/NY.GDP.PCAP.PP.CD` (2024).
16. World Bank. *World Development Indicators - Gini Index* World Development Indicators (WDI). 2024. `https://data.worldbank.org/indicator/SI.POV.GINI` (2024).
17. Prieto-Curiel, R. *El inventario de la movilidad de México* 2023. `https://datos.nexos.com.mx/el-inventario-de-la-movilidad-de-mexico/`.
18. Portugali, J. in *Encyclopedia of complexity and systems science* 1–49 (Springer, 2017).
19. Schelling, T. C. Dynamic models of segregation. *Journal of Mathematical Sociology* **1,** 143–186 (1971).
20. Pieterse, E., Parnell, S. & Haysom, G. African dreams: locating urban infrastructure in the 2030 sustainable developmental agenda. *Area Development and Policy* **3,** 149–169 (2018).
21. Berrill, P. *et al.* Comparing urban form influences on travel distance, car ownership, and mode choice. *Transportation Research Part D: Transport and Environment* **128,** 104087 (2024).
22. Prieto-Curiel, R. & Barroso, F. The growing dominance of cars in suburban areas. *Transportation Research Interdisciplinary Perspectives* **32,** 101559 (2025).
23. Frolking, S., Mahtta, R., Milliman, T., Esch, T. & Seto, K. C. Global urban structural growth shows a profound shift from spreading out to building up. *Nature Cities* **1,** 555–566 (2024).
24. United Nations, P. D. *World Urbanization Prospects (2025 Revision)* 2025. `https://population.un.org/wup/`.

25. Cantor, D. J. The New Wave: forced Displacement Caused by Organized Crime in Central America and Mexico. *Refugee Survey Quarterly* **33,** 34–68 (2014).

26. Metzler, R., Jeon, J.-H., Cherstvy, A. G. & Barkai, E. Anomalous diffusion models and their properties: non-stationarity, non-ergodicity, and ageing at the centenary of single particle tracking. *Physical Chemistry Chemical Physics* **16,** 24128–24164 (2014).

27. Tatem, A. J. WorldPop, open data for spatial demography. *Scientific data* **4,** 1–4 (2017).

28. Carioli, A., Schiavina, M., MacManus, K. J. & Freire, S. *GHS-POP R2023A - GHS population grid multitemporal (1975-2030)* en. 2023. `http://data.europa.eu/89h/2ff68a52-5b5b-4a22-8f40-c41da8332cfe`.

29. Stevens, F. R., Gaughan, A. E., Linard, C. & Tatem, A. J. Disaggregating census data for population mapping using random forests with remotely-sensed and ancillary data. *PloS one* **10,** e0107042 (2015).

30. Pesaresi, M. & Politis, P. GHS-BUILT-V R2023A-GHS built-up volume grids derived from joint assessment of Sentinel2, Landsat, and global DEM data, multitemporal (1975-2030). *European Commission, Joint Research Centre (JRC)* (2023).

31. Kuffer, M., Owusu, M., Oliveira, L., Sliuzas, R. & van Rijn, F. The missing millions in maps: Exploring causes of uncertainties in global gridded population datasets. *ISPRS International Journal of Geo-Information* **11,** 403 (2022).

32. Cleveland, W. S. Robust locally weighted regression and smoothing scatterplots. *Journal of the American statistical association* **74,** 829–836 (1979).

33. Láng-Ritter, J., Keskinen, M. & Tenkanen, H. Global gridded population datasets systematically underrepresent rural population. *Nature Communications* **16,** 2170 (2025).
