# Supplementary material for: Scaling and Population Loss in Mexican Urban Centres
Source: Nat Commun. 2026 Jul 25;17:9039. doi: 10.1038/s41467-026-75780-5 (PMC13507054; doi:10.1038/s41467-026-75780-5)
Supplement: Supplementary file 2 [file 41467_2026_75780_MOESM2_ESM.pdf]

nature portfolio

Corresponding author(s): Eugen Resendiz

Last updated by author(s): Jun 15, 2026

Reporting Summary

Nature Portfolio wishes to improve the reproducibility of the work that we publish. This form provides structure for consistency and transparency in reporting. For further information on Nature Portfolio policies, see our Editorial Policies and the Editorial Policy Checklist.

Please do not complete any field with "not applicable" or n/a. Refer to the help text for what text to use if an item is not relevant to your study.
For final submission: please carefully check your responses for accuracy; you will not be able to make changes later.

Statistics

For all statistical analyses, confirm that the following items are present in the figure legend, table legend, main text, or Methods section.

n/a	Confirmed	
☐	☒	The exact sample size (n) for each experimental group/condition, given as a discrete number and unit of measurement
☐	☒	A statement on whether measurements were taken from distinct samples or whether the same sample was measured repeatedly
☐	☒	The statistical test(s) used AND whether they are one- or two-sided<br>*Only common tests should be described solely by name; describe more complex techniques in the Methods section.*
☐	☒	A description of all covariates tested
☐	☒	A description of any assumptions or corrections, such as tests of normality and adjustment for multiple comparisons
☐	☒	A full description of the statistical parameters including central tendency (e.g. means) or other basic estimates (e.g. regression coefficient) AND variation (e.g. standard deviation) or associated estimates of uncertainty (e.g. confidence intervals)
☐	☒	For null hypothesis testing, the test statistic (e.g. F, t, r) with confidence intervals, effect sizes, degrees of freedom and P value noted<br>*Give P values as exact values whenever suitable.*
☒	☐	For Bayesian analysis, information on the choice of priors and Markov chain Monte Carlo settings
☒	☐	For hierarchical and complex designs, identification of the appropriate level for tests and full reporting of outcomes
☒	☐	Estimates of effect sizes (e.g. Cohen's d, Pearson's r), indicating how they were calculated

Our web collection on statistics for biologists contains articles on many of the points above.

Software and code

Policy information about availability of computer code

Data collection	No software was used, data was downloaded from the official repositories from the National Institute of Statistics and Geography of Mexico
Data analysis	All code used to build the dataset and perform the analyses (developed in Python) is publicly available at https://github.com/CentroFuturoCiudades/scaling_depopulation_mex and archived on Zenodo at https://doi.org/10.5281/zenodo.20630381.

For manuscripts utilizing custom algorithms or software that are central to the research but not yet described in published literature, software must be made available to editors and reviewers. We strongly encourage code deposition in a community repository (e.g. GitHub). See the Nature Portfolio guidelines for submitting code & software for further information.

Data

Policy information about availability of data

All manuscripts must include a data availability statement. This statement should provide the following information, where applicable:

- Accession codes, unique identifiers, or web links for publicly available datasets
- A description of any restrictions on data availability
- For clinical datasets or third party data, please ensure that the statement adheres to our policy

The processed population data generated in this study---the high-resolution gridded population dataset for the 69 Mexican metropolitan areas across the 1990, 2000, 2010, and 2020 censuses, together with the derived radial density functions---have been deposited in the Zenodo repository\cite{depositionZenodo} under accession code \href{https://doi.org/10.5281/zenodo.20630381}{10.5281/zenodo.20630381}, and are also available at the project repository (\url{https://

github.com/CentroFuturoCiudades/scaling_depopulation_mex}).
The raw census data used in this study are third-party data owned by Mexico's National Institute of Statistics and Geography (INEGI). The 2000, 2010, and 2020 census population counts and AGEB geometries are available in the INEGI database (\url{https://www.inegi.org.mx/}). The 1990 AGEB geometries are available under restricted access, because INEGI distributes them under terms of use that do not permit redistribution by the authors; access can be obtained by requesting them directly from INEGI through its System for Census Information Consultation (SCINCE) (\url{https://www.inegi.org.mx/}). The processed 1990 population grid derived from these geometries is included in the deposited dataset above, so no restricted-access data are required to reproduce the results reported here.

Research involving human participants, their data, or biological material

Policy information about studies with human participants or human data. See also policy information about sex, gender (identity/presentation), and sexual orientation and race, ethnicity and racism.

Reporting on sex and gender	NA
Reporting on race, ethnicity, or other socially relevant groupings	NA
Population characteristics	NA
Recruitment	NA
Ethics oversight	NA

Note that full information on the approval of the study protocol must also be provided in the manuscript.

Field-specific reporting

Please select the one below that is the best fit for your research. If you are not sure, read the appropriate sections before making your selection.

☐ Life sciences ☐ Behavioural & social sciences ☒ Ecological, evolutionary & environmental sciences

Ecological, evolutionary & environmental sciences study design

All studies must disclose on these points even when the disclosure is negative.

Study description	This study analysed urban growth dynamics in 69 Mexican metropolitan areas from 1990 to 2020 using census data, developing a model of urban form change based on population size, density, and spatial configuration. We employ a radial probability density function and the urban expansion factor to create a framework for comparing urban expansion over time and across different regions.
Research sample	69 Mexican metropolitan areas
Sampling strategy	No sampling procedure was used; we used the whole population of Mexican metropolitan areas with available data for the study period (1990 to 2020).
Data collection	Data was downloaded from the official repositories from the National Institute of Statistics and Geography of Mexico.
Timing and spatial scale	Data was obtained at the census tract (AGEB) level for 69 Mexican metropolitan areas for the census periods of 1990, 2000, 2010 and 2020.
Data exclusions	Only the metropolitan area of Playa del Carmen, Mexico was excluded since no data was available for the 1990 census.
Reproducibility	NA
Randomization	NA
Blinding	NA

Did the study involve field work? ☐ Yes ☒ No

Reporting for specific materials, systems and methods

We require information from authors about some types of materials, experimental systems and methods used in many studies. Here, indicate whether each material, system or method listed is relevant to your study. If you are not sure if a list item applies to your research, read the appropriate section before selecting a response.

Materials & experimental systems

n/a	Involved in the study
☒	☐ Antibodies
☒	☐ Eukaryotic cell lines
☒	☐ Palaeontology and archaeology
☒	☐ Animals and other organisms
☒	☐ Clinical data
☒	☐ Dual use research of concern
☒	☐ Plants

Methods

n/a	Involved in the study
☒	☐ ChIP-seq
☒	☐ Flow cytometry
☒	☐ MRI-based neuroimaging

Plants

Seed stocks	NA
Novel plant genotypes	NA
Authentication	NA

This checklist template is licensed under a Creative Commons Attribution 4.0 International License, which permits use, sharing, adaptation, distribution and reproduction in any medium or format, as long as you give appropriate credit to the original author(s) and the source, provide a link to the Creative Commons license, and indicate if changes were made. The images or other third party material in this article are included in the article's Creative Commons license, unless indicated otherwise in a credit line to the material. If material is not included in the article's Creative Commons license and your intended use is not permitted by statutory regulation or exceeds the permitted use, you will need to obtain permission directly from the copyright holder. To view a copy of this license, visit http://creativecommons.org/licenses/by/4.0/
