# Supplementary material for: Scaling and Population Loss in Mexican Urban Centres
Source: Nat Commun. 2026 Jul 25;17:9039. doi: 10.1038/s41467-026-75780-5 (PMC13507054; doi:10.1038/s41467-026-75780-5)
Supplement: Supplementary file 3 [file 41467_2026_75780_MOESM3_ESM.pdf]

nature portfolio

Peer Review File

Scaling and Population Loss in Mexican Urban Centres

Corresponding Author: Dr Eugen Resendiz

This file contains all reviewer reports in order by version, followed by all author rebuttals in order by version.

Version 0:

Reviewer comments:

Reviewer #1

(Remarks to the Author)
The manuscript presents an ambitious and comprehensive study of population loss in Mexican urban centres. The use of radial probability density functions and the concept of the urban expansion factor provide a novel way to analyse metropolitan dynamics. The main finding, that city centres lost 2.5 million residents between 1990 and 2020 despite national urban growth, is significant and of interest for both research and policy.
The methodology is generally solid and carefully explained, especially the construction of the population grid and the idea of remoteness. However, some parts of the methods are very technical and could be simplified or moved to supplementary material for easier reading. The data sources are clear and reproducible, though I think the authors should discuss more about possible biases in INEGI data compared with alternatives such as WorldPop.
The results are strong and well illustrated, although some figures are crowded. For example, the map of 69 cities is difficult to read and may be better placed in the supplementary information. The typology of six regimes of density change is useful, but the policy meaning of each regime is not fully developed. The discussion provides valuable connections with diffusion concepts, but some terms may not be accessible for a broader audience. The link to international experiences is also limited, and expanding this would make the contribution stronger. Some conclusions, especially those connecting centre decline with housing reforms, feel speculative without stronger evidence or references.
In general the paper is well written, although some sentences are very long and dense. Careful editing for clarity would improve accessibility.Overall, this is an important contribution, but I recommend major revision before publication. The authors should simplify parts of the methodology, clarify data issues, make figures clearer, and provide a stronger discussion of causes and international comparisons.

(Remarks on code availability)
Yes, the code is easy to access.

Reviewer #3

(Remarks to the Author)
This paper investigates the counterintuitive coexistence of population growth and urban core depopulation in Mexican metropolitan areas from 1990 to 2020. Using census-based population grids and a scaling approach, the authors demonstrate that nearly all 69 Mexican metropolitan areas have expanded outward while losing population density in their central zones. The study is conceptually ambitious and methodologically sophisticated, providing a mathematical formalization of a process that urban geography has long recognized as suburbanization. However, several issues need to be addressed before the manuscript can be considered for publication. My specific comments are as follows:

1. My primary concern relates to the definition of location remoteness, which serves as the foundation of this study. The authors define it as the ratio of distance to the square root of urban population, aiming to ensure comparability across cities under the assumption that urban population density is similar. However, urban area actually scales nonlinearly with urban population—a phenomenon known as urban scaling, with an exponent of approximately 0.85. As a result, the distance-based remoteness index, adjusted only by the square root of population, remains incomparable across cities of different sizes. Would it not be more appropriate to replace population with urban area in the construction of the remoteness index?

2. The observed pattern, population decline in central areas accompanied by growth in peripheral zones, is a classic hallmark of suburbanization, extensively documented in North American and European urban studies. The manuscript

briefly mentions this link but does not situate its findings within this conceptual tradition. The authors should explicitly discuss how their scaling-based interpretation corresponds to, or extends, the conventional suburbanization framework. Doing so would help bridge the mathematical model with established urban geography literature.

3. Beyond the statistical and modeling dimensions, the paper should provide a clearer account of why suburbanization is occurring in Mexico. Are these trends driven by housing policies, infrastructure investment, economic restructuring, or demographic shifts? A short qualitative or quantitative discussion of the underlying mechanisms would enrich the interpretation of the scaling results.

4. While the decline of central populations is well demonstrated, the temporal dynamics are not discussed. When did this process begin, and was there an earlier phase of central population growth? In particular, why do central areas show pronounced decline before 2010? A brief temporal analysis or commentary on the inflection point of this trend would add valuable historical context.

5. Overall, this paper introduced multiple new constructs, such as the radial probability density function, urban expansion factor, remoteness scaling, and the decomposition into extensive and intensive processes, all in mathematical form without much intuitive explanation. The ideas are powerful but not reader-friendly.I strongly suggest that the authors include intuitive explanations, schematic illustrations, or worked examples to make the conceptual innovations accessible to a broader readership.

6. Figures 1 and 2 convey largely overlapping information. Consider moving Figure 2 to the Supplementary Information to streamline the main text.

7. In addition to the classical models cited (e.g., negative exponential functions), there are recent methodological developments that may be relevant to this work, like functions describing the gradients of urban land density or urban intensity.

(Remarks on code availability)

Version 1:

Reviewer comments:

Reviewer #1

(Remarks to the Author)
The authors have made substantial efforts to address the concerns raised in the first round of review. The revised manuscript is clearer, particularly in the methodological sections, where several technical explanations have been simplified and moved to the Supplementary Information. The authors also improved the connection of the study with the broader literature on suburbanization and urban spatial dynamics, which strengthens the conceptual framing of the paper. In addition, the discussion now provides more explanation of the possible mechanisms behind central population decline, including housing policies, infrastructure expansion, and demographic changes. The figures appear to have been simplified and are easier to interpret than in the previous version. Overall, most of the reviewer comments have been addressed in a satisfactory way and the manuscript has improved in clarity and contextualization. I believe the paper now represents a valuable contribution to the study of urban spatial dynamics, and I would support publication after minor editorial revisions.

(Remarks on code availability)
The shared code is well-organized.

Reviewer #3

(Remarks to the Author)
Thank you for the responses to all my comments.
Regarding one of my main concerns, the definition of "remoteness," I would like to add a further point. The author’s assumption that individuals in different cities occupy the same amount of space may not be rigorous. In fact, large cities tend to have higher population densities, which can be much greater than those in smaller cities. I suggest analyzing and elaborating on the uncertainty of the assumption and its potential impact on the results.

(Remarks on code availability)

Version 2:

Reviewer comments:

Reviewer #3

(Remarks to the Author)

Thank you for addressing all of my concerns. I believe the paper is now suitable for publication in this edition.

(Remarks on code availability)

Open Access This Peer Review File is licensed under a Creative Commons Attribution 4.0 International License, which permits use, sharing, adaptation, distribution and reproduction in any medium or format, as long as you give appropriate credit to the original author(s) and the source, provide a link to the Creative Commons license, and indicate if changes were made.
In cases where reviewers are anonymous, credit should be given to 'Anonymous Referee' and the source.
The images or other third party material in this Peer Review File are included in the article's Creative Commons license, unless indicated otherwise in a credit line to the material. If material is not included in the article's Creative Commons license and your intended use is not permitted by statutory regulation or exceeds the permitted use, you will need to obtain permission directly from the copyright holder.
To view a copy of this license, visit https://creativecommons.org/licenses/by/4.0/

Point by point answer

We are pleased to resubmit our manuscript titled "Scaling and Population Loss in Mexican Urban Centre" for consideration for publication in Nature Communications. We appreciate the reviewers for their positive assessment and recognition of the significance of our findings. After carefully reviewing all comments, we have addressed them as requested.

Reviewer 1

Reviewer (R1.1)*: The manuscript presents an ambitious and comprehensive study of population loss in Mexican urban centres. The use of radial probability density functions and the concept of the urban expansion factor provide a novel way to analyse metropolitan dynamics. The main finding, that city centres lost 2.5 million residents between 1990 and 2020 despite national urban growth, is significant and of interest for both research and policy. The methodology is generally solid and carefully explained, especially the construction of the population grid and the idea of remoteness. However, some parts of the methods are very technical and could be simplified or moved to supplementary material for easier reading.*

We thank the reviewer for the positive assessment and recognition of the relevance of our findings. We clarified that we simplified the technical sections where possible and moved detailed methodological steps to the supplementary material to improve readability.

Reviewer (R1.2)*: The data sources are clear and reproducible, though I think the authors should discuss more about possible biases in INEGI data compared with alternatives such as WorldPop.*

We added a short discussion of potential biases and differences between official INEGI census data and global population grids from WorldPop and Global Human Settlement Layer (GHS-POP). Although using census data has many limitations (such as the size of some units of observation or AGEBS), the added value is that we can base our study on the same data (and method for obtaining it) since 1990. Other sources, such as GHS-POP, present discrepancies with official national data.

We present a more detailed comparison of these data products in Manuscript section 5.1 Urban population mesh and in Supplementary Note 7 Error estimation for global population grids. In brief, we present evidence that global population grids exhibit spatial biases.

Reviewer (R1.3)*: The results are strong and well illustrated, although some figures are crowded.*

For example, the map of 69 cities is difficult to read and may be better placed in the supplementary information.

We acknowledge that some figures appear crowded. We have redesigned and adjusted most figures to improve readability. We have removed the figure showing the 69 cities and generated a new, reduced figure with 19 selected cities. Additionally, we generated an interactive version of the figure that includes all cities, which is available at: CitiesMoving.com/UrbanDispersion. The full set of maps are also available in Supplementary Note 8.

Reviewer (R1.4)*: The typology of six regimes of density change is useful, but the policy meaning of each regime is not fully developed.*

We expanded the description of the policy implications of each density change regime and clarified how the regimes could inform planning priorities and monitoring in the results section. We also added a brief description and examples of the scenario in Supplementary Notes Section 5: Different scenarios and implications at the city level.

Reviewer (R1.5)*: The discussion provides valuable connections with diffusion concepts, but some terms may not be accessible for a broader audience.*

We revised the discussion and methods to simplify terminology and moved all references to diffusion to Supplementary Note 6: Dynamical regimes of the scaling model.

Reviewer (R1.6)*: The link to international experiences is also limited, and expanding this would make the contribution stronger.*

We expanded the link to international experiences, in the Introduction and Results sections, by adding examples of trajectories in other regions: Berlin after the fall of the wall [1], car dependency in the US, Canada, some cities in India and Africa and its impact on urban forms [2, 3, 4, 5, 6]. We aim to situate the case of Mexican cities as an early stage of the trajectory followed by US cities, but ahead, at least from a demographic point of view, of African cities. We also clarified how our findings related to broader urbanisation patterns.

Reviewer (R1.7)*: Some conclusions, especially those connecting centre decline with housing reforms, feel speculative without stronger evidence or references.*

We removed some of our conclusions and strengthened the findings by adding supporting evidence and references. In detail, we added two paragraphs in the Discussion section explaining how demographic, urban growth, economic, and land-use changes have impacted outward migration, urban sprawl, peripheral housing development, and reinforced the depopulation of central areas. These were complemented by Supplementary Note 3: A country in evolution. There, we provide a more detailed account of the demographic and economic changes that have contributed to the observed phenomena. Also, in Supplementary Note 5, Different scenarios and implications at the city level, we provide a more detailed example for Acapulco.

Reviewer (R1.8)*: In general, the paper is well written, although some sentences are very long and dense. Careful editing for clarity would improve accessibility.*

We carefully revised the text and edited long, dense sentences for clarity and improved overall readability throughout the manuscript. Additionally, we rewrote the mathematical derivations to improve clarity.

Reviewer (R1.9)*: Overall, this is an important contribution, but I recommend major revision before publication. The authors should simplify parts of the methodology, clarify data issues, make figures clearer, and provide a stronger discussion of causes and international comparisons.*

We addressed the concerns raised by simplifying parts of the methodology by moving some elements to the supplementary information. Additionally, we have addressed data issues, improved figure clarity, and strengthened the discussion of causes and international comparisons, as detailed in the point-by-point replies.

Thank you for the time invested in our manuscript and the constructive criticism.

Reviewer 2

***Reviewer (R2.1)**: This paper investigates the counterintuitive coexistence of population growth and urban core depopulation in Mexican metropolitan areas from 1990 to 2020. Using census-based population grids and a scaling approach, the authors demonstrate that nearly all 69 Mexican metropolitan areas have expanded outward while losing population density in their central zones. The study is conceptually ambitious and methodologically sophisticated, providing a mathematical formalization of a process that urban geography has long recognized as suburbanization. However, several issues need to be addressed before the manuscript can be considered for publication.*

Thank you for this comment. We acknowledge the need to clarify the conceptual and methodological contributions relative to established suburbanisation literature. We added a note in the introduction and discussion to situate our approach within existing urban geography research and to emphasise how the scaling framework and population grids extended prior work.

***Reviewer (R2.2)**: My primary concern relates to the definition of location remoteness, which serves as the foundation of this study. The authors define it as the ratio of distance to the square root of urban population, aiming to ensure comparability across cities under the assumption that urban population density is similar. However, urban area actually scales nonlinearly with urban population—a phenomenon known as urban scaling, with an exponent of approximately 0.85. As a result, the distance-based remoteness index, adjusted only by the square root of population, remains incomparable across cities of different sizes. Would it not be more appropriate to replace population with urban area in the construction of the remoteness index?*

The rationale behind using remoteness is that it allows for rescaling cities to a similar size, without distorting their population density values. We use remoteness to compare population densities transversally across cities, so this property is of particularly importance to our results. Moreover, the derivation of the urban scaling factor and the observation of temporal scaling of the radial distribution do not use remoteness and are both independent of the particular transversal scaling exponent for area. We have highlighted this differences in Results and Supplementary Note 2: Distance to the city centre, remoteness, and alternative scaling exponents.

Remoteness is defined as the distance divided by the square root of the population (instead of taking the area directly) as follows. First, imagine a city with P inhabitants. If each person occupies a fixed surface in that city (say μ m^2), then the whole urban area occupies a surface of μP m^2. If the population is arranged in a circular and compact manner (like a honeycomb), then they would be placed in a circle with radius $r = \sqrt{\mu P/\pi} = \alpha\sqrt{P}$, for some value of $\alpha = \sqrt{\mu/\pi}$, which does not depend on the population. In that idealised city, we can express its radius as a function of the square root of the urban population. This idealised city is not necessarily observed, and larger cities could

reduce their radius if their coefficient α shrinks as they grow. However, the opposite could also be true, meaning that larger cities could expand with larger values of α. The idealised city enables us to compare more urban indicators, for example, the distance between two randomly selected points within each city [7]. In that idealised (round and compact) city, the expected distance between two randomly-selected points is $d = 128\alpha/45\pi\sqrt{P} = \kappa\sqrt{P}$, for some value of $\kappa = 128\alpha/45\pi$. Thus, in those simplified cities, the distance between any two randomly-selected points is proportional to the square root of the population. The same applies to the distance to the centre, which scales with the square root of the population. We applied the same principle of using the square root of the population to scale distances (or the radius of a city) across more than 100 cities in Africa, Asia, and Latin America to detect the access to different services, such as piped water and sewage [8].

Additionally, for the urban area scaling coefficient, we could use 0.85 as suggested. However, although it has been assumed that city surfaces scale sublinearly, this has not been observed across all geographies. Even within neighbouring regions, the urbanised area was found to be sublinear in France and the UK (with $\beta < 1$), but (statistically speaking) linear in Spain, Italy, and Germany (with β inside an interval that contains the value of 1) [9]. Outside Europe, there is also evidence of the scaling area of cities. For example, by looking at nearly 6,000 urban areas in Africa, we detected that the surface of the city is superlinear, and the distance between two random locations is proportional to $\kappa' = P^{0.532}$, so even longer distances than the model with round and compact cities [7]. Thus, using scaling coefficients obtained for a set of cities and applying them elsewhere is not straightforward.

A similar process applies to other scaling coefficients. As with many scaling coefficients, it remains unclear whether they are, in fact, universal and can be used blindly across different geographies. For example, it was claimed that larger cities tend to exhibit superlinear scaling of violence and crime, but recently, we found that, across nearly 50 studies, there is no universal pattern in such coefficients [10]. For some types of crime, it scales superlinearly, but for others, it is sublinear, or even city size has a minor effect on a per capita basis.

Now, if, instead of the linear expansion of the surface, we take it as a function of size (as suggested by the coefficient 0.85), we would obtain the following. The surface would have a scaling coefficient of $\beta = 0.85$ or linear with $\beta = 1$, resulting in the scaling of the distance to the centre varying with a coefficient in the $0.85/2 = 0.425$ and 0.5 intervals. The distinction in both extremes of the interval is not huge. Consider, for example, Mexico City (with 21 million people) and Merida (with 1 million people). Using $\beta = 1$ for the surface results in a coefficient of 0.5 when comparing the distance to the centre. A remoteness of $R = 10$ is 47km away from the city centre in Mexico City and only 10km away from the centre of Merida. However, if we used a different coefficient (say, 0.85 for the surface and therefore, 0.425 for the radius), then the same distance (47km) would have a 'remoteness' of 35 units in Mexico City, but in Merida (only 10km) would have a 'remoteness' of 28 units. Therefore, using different scaling values for the surface coefficient would only slightly compress the cumulative curves but would not alter the urban population loss near the centre of all cities in Mexico.

We have added this explanation to the Supplementary Note 2: Distance to the city centre, remoteness, and alternative scaling exponents, along with a couple of illustrations to highlight the relevance of using population rather than surface area as the unit of comparison across cities. We included as well

a comparison with alternative scaling exponents, 5/6 from Urban Scaling Theory and 2/3 (homothetic scaling [11]), where they are discussed within the scaling framework proposed in this study.

Reviewer (R2.3)*: The observed pattern, population decline in central areas accompanied by growth in peripheral zones, is a classic hallmark of suburbanization, extensively documented in North American and European urban studies. The manuscript briefly mentions this link but does not situate its findings within this conceptual tradition. The authors should explicitly discuss how their scaling-based interpretation corresponds to, or extends, the conventional suburbanization framework. Doing so would help bridge the mathematical model with established urban geography literature.*

We expanded the discussion to explicitly relate our findings to the literature on suburbanisation. We added some references, particularly Kenneth T. Jackson's book on the suburbanisation of the US [3]. We also clarified how the scaling-based interpretation complemented and extended conventional frameworks. We added references to classic and contemporary studies and explained how our approach quantified patterns that had previously been described qualitatively. We refined the introduction and discussion to situate the contribution more clearly within this tradition.

Additionally, we expanded the link to international experiences, in the Introduction and Results sections, by adding examples of trajectories in other regions: Berlin after the fall of the wall [1], car dependency in the US, Canada, some cities in India and Africa and its impact on urban forms [2, 3, 4, 5, 6]. We aim to situate the case of Mexican cities as an early stage of the trajectory followed by US cities, but ahead, at least from a demographic point of view, of African cities. We also clarified how our findings related to broader urbanisation patterns.

Reviewer (R2.4)*: Beyond the statistical and modelling dimensions, the paper should provide a clearer account of why suburbanization is occurring in Mexico. Are these trends driven by housing policies, infrastructure investment, economic restructuring, or demographic shifts? A short qualitative or quantitative discussion of the underlying mechanisms would enrich the interpretation of the scaling results.*

We added concise discussion paragraphs of the main mechanisms that contributed to suburbanisation in Mexico and drew on existing empirical studies to ground the explanation. We clarified that the process results from the complex interactions among those mechanisms, rather than from a single cause (see answer to R1.7 for additional details). There is also a crucial link between housing policy changes, infrastructure expansion, labour market restructuring, and demographic shifts. We expanded on these topics in the Supplementary Notes 3: A country in evolution, indicating how these factors align with the spatial patterns we documented. We kept this section focused to avoid overinterpretation while strengthening the link between mechanisms and observed outcomes.

Reviewer (R2.5)*: While the decline of central populations is well demonstrated, the temporal dynamics are not discussed. When did this process begin, and was there an earlier phase of central population growth? In particular, why do central areas show a pronounced decline before 2010? A brief*

temporal analysis or commentary on the inflection point of this trend would add valuable historical context.

We explicitly link the pronounced decline before 2010 with population growth rates in the Results. We complemented our Discussion section by expanding on population growth rates as well as on the changes in the country's economy and housing regulations that increased housing buying power, car ownership, and enabled private developers to build affordable housing in peripheral areas (see response to comment R1.7 and Supplementary Note 3: A country in evolution).

Reviewer (R2.6)*: Overall, this paper introduced multiple new constructs, such as the radial probability density function, urban expansion factor, remoteness scaling, and the decomposition into extensive and intensive processes, all in mathematical form without much intuitive explanation. The ideas are powerful but not reader-friendly. I strongly suggest that the authors include intuitive explanations, schematic illustrations, or worked examples to make the conceptual innovations accessible to a broader readership.*

We designed two new figures to provide intuitive illustrations, complemented by short explanations. Figure 1 illustrates the overall methodology of our approach, including examples of radial functions and remoteness scaling. Figure 4 illustrates the temporal scaling of radial functions and how the urban factor is estimated using the city of Colima as an example. Furthermore, we streamlined the mathematical presentation and complemented it with brief conceptual summaries to improve accessibility without reducing analytical precision.

Reviewer (R2.7)*: Figures 1 and 2 convey largely overlapping information. Consider moving Figure 2 to the Supplementary Information to streamline the main text.*

We adjusted most of our Figures to avoid redundancy and improve readability. In detail, we removed the figure showing 69 cities (previously Figure 1) and generated a new, simplified figure with 19 selected cities. Additionally, we generated an interactive version of the figure that includes all cities, available at CitiesMoving.com/UrbanDispersion, making the entire set easy to explore. The full set of maps is also available in Supplementary Note 8.

Reviewer (R2.8)*: In addition to the classical models cited (e.g., negative exponential functions), there are recent methodological developments that may be relevant to this work, like functions describing the gradients of urban land density or urban intensity.*

We incorporated recent methodological developments on urban land density and urban intensity gradients and added citations to position our approach relative to these models [12, 13, 14, 15, 11]. We clarified the complementarities and noted where our framework offered additional insights. We added a more detailed comparison with recent work on Urban Scaling Theory [16, 17] and homothetic scaling [11] in Supplementary Note 2.

References

[1] Gabriel M Ahlfeldt, Stephen J Redding, Daniel M Sturm, and Nikolaus Wolf. The economics of density: Evidence from the Berlin Wall. *Econometrica*, 83(6):2127–2189, 2015.

[2] Peter Berrill, Florian Nachtigall, Aneeque Javaid, Nikola Milojevic-Dupont, Felix Wagner, and Felix Creutzig. Comparing urban form influences on travel distance, car ownership, and mode choice. *Transportation Research Part D: Transport and Environment*, 128:104087, 2024.

[3] Kenneth T Jackson. *Crabgrass frontier: The suburbanization of the United States*. Oxford University Press, Oxford, UK, 1987.

[4] Kala Seetharam Sridhar. Density gradients and their determinants: Evidence from India. *Regional Science and Urban Economics*, 37(3):314–344, May 2007.

[5] Luís MA Bettencourt and Nicholas Marchio. Infrastructure deficits and informal settlements in sub-Saharan Africa. *Nature*, pages 1–8, 2025.

[6] Mariaflavia Harari. Cities in bad shape: Urban geometry in India. *American Economic Review*, 110(8):2377–2421, 2020.

[7] Rafael Prieto-Curiel, Jorge E Patino, and Brilé Anderson. Scaling of the morphology of African cities. *Proceedings of the National Academy of Sciences*, 120(9):e2214254120, 2023.

[8] Rafael Prieto-Curiel, Pavel Luengas-Sierra, and Christian Borja-Vega. Urban sprawl is associated with reduced access and increased costs of water and sanitation. *Nature Cities*, pages 1–12, 2025.

[9] Luís M. A. Bettencourt and José Lobo. Urban scaling in Europe. *Journal of The Royal Society Interface*, 13(116):20160005, March 2016. Publisher: Royal Society.

[10] Rafael Prieto-Curiel and Ronaldo Menezes. Violence, city size and geographical isolation in African cities. *Nature Communications*, 16(1):10049, 2025.

[11] Gaëtan Laziou and Rémi Lemoy. The three-dimensional structure of population density in world cities. *npj Urban Sustainability*, 5(1):75, 2025.

[12] Limin Jiao. Urban land density function: A new method to characterize urban expansion. *Landscape and Urban Planning*, 139:26–39, 2015.

[13] Rémi Lemoy and Geoffrey Caruso. Radial analysis and scaling of urban land use. *Scientific Reports*, 11(1):22044, 2021.

[14] Huiyuan Lu, Zhengyong Shang, Yanling Ruan, and Linlin Jiang. Study on urban expansion and population density changes based on the inverse s-shaped function. *Sustainability*, 15(13):10464, 2023.

[15] Muchen Zheng, Wenli Huang, Gang Xu, Xi Li, and Limin Jiao. Spatial gradients of urban land density and nighttime light intensity in 30 global megacities. *Humanities and Social Sciences Communications*, 10(1):1–11, 2023.

[16] Luís MA Bettencourt. The origins of scaling in cities. *Science*, 340(6139):1438–1441, 2013.

[17] Jose Lobo, Scott Ortman, and Luis Bettencourt. Systematic Relationship Between Areal Extent and Population for Urban Areas in the USA, December 2024. ISSN: 2693-5015.

Point by point answer

We are pleased to resubmit our manuscript titled "Scaling and Population Loss in Mexican Urban Centres" for consideration for publication in Nature Communications. We are grateful to our reviewers for their continued engagement with our work. After carefully reviewing all comments, we have addressed them as requested. Our responses and changes are provided below; at the end of this document, we provide a version of the manuscript with tracked changes.

Reviewer 1

Reviewer (R1.1): *The authors have made substantial efforts to address the concerns raised in the first round of review. The revised manuscript is clearer, particularly in the methodological sections, where several technical explanations have been simplified and moved to the Supplementary Information. The authors also improved the connection of the study with the broader literature on suburbanization and urban spatial dynamics, which strengthens the conceptual framing of the paper. In addition, the discussion now provides more explanation of the possible mechanisms behind central population decline, including housing policies, infrastructure expansion, and demographic changes. The figures appear to have been simplified and are easier to interpret than in the previous version. Overall, most of the reviewer comments have been addressed in a satisfactory way and the manuscript has improved in clarity and contextualization. I believe the paper now represents a valuable contribution to the study of urban spatial dynamics, and I would support publication after minor editorial revisions.*

We thank Reviewer 1 for the thorough and positive assessment and for the recommendation to publish. We have carefully proofread the entire manuscript and corrected several editorial issues. The full list of corrections is provided below.

- Missing spaces added throughout the text.
- Spelling made consistent with British English used throughout.
- Spurious words removed and typos corrected.
- **Results, Figure 2 caption.** Zone name corrected to match the nomenclature defined in the text (central / intermediate / *distant* / peri-urban): "*suburban*" $\rightarrow$ "*distant*".
- **Discussion, paragraph 3.** NAFTA is a free trade agreement, not a trade union; the term was corrected and the acronym introduced on first use.

- **Supplementary Note 3, Economic changes.** Same NAFTA terminology correction applied: "economic trade union between Mexico, the USA, and Canada (NAFTA) in 1994" → "North American Free Trade Agreement (NAFTA) between Mexico, the USA, and Canada in 1994".

Reviewer (R1.2— Remarks on code availability)*: The shared code is well-organized.*
We thank the reviewer for examining and commenting positively on the shared code.

Reviewer 3

Reviewer (R3.1): *Thank you for the responses to all my comments. Regarding one of my main concerns, the definition of "remoteness," I would like to add a further point. The author's assumption that individuals in different cities occupy the same amount of space may not be rigorous. In fact, large cities tend to have higher population densities, which can be much greater than those in smaller cities. I suggest analyzing and elaborating on the uncertainty of the assumption and its potential impact on the results.*

We thank the reviewer for this additional point and for the continued interest in the robustness of the remoteness definition. We have carefully considered this concern and wish to clarify two points. **One is that remoteness is defined to preserve population density values, not to match empirical city geometry.** The remoteness of a location in city i is defined as $r = 1000\, s/\sqrt{P_i}$, corresponding to a scaling exponent $\beta = 0.5$ in the general formula $r \propto s/P^{\beta}$. The constant per-capita space assumption ($\mu = \text{const}$) provides one geometric motivation for this choice: if each person occupied the same surface area μ in every city, the expected city radius would scale as $\sqrt{P}$, making $\sqrt{P}$ a natural unit for distances. However, this idealised description need not hold exactly; large cities may be somewhat denser. Yet, this increase in density does not alter much what is considered the centre of the city. We have included the following test in the Supplementary Materials.

We have used the square root of the population to scale distances in cities, but we could also consider an alternative scaling model based on area. Consider the area of the city as $A = \alpha P^{\gamma}$, for some value of γ. If large cities had a higher density, then $\gamma < 1$, but if large cities are equally dense, then we would observe that $\gamma \approx 1$. Then, instead of scaling distances by the square root of the population, we could consider distances divided by P^{β} with $\beta = \gamma/2$. For example, if we used a value of $\gamma = 5/6$, as predicted by Urban Scaling Theory [1] and observed in France [2] and the US [3], we would obtain the following. Consider, for example, the cases of Mexico City (with approximately 22 million people) and Merida (with nearly 1 million people). A remoteness of $r = 3$, identifying the central part of the city, is 14 km away from the city centre in Mexico City and only 3 km away from the centre of Merida. Those locations are considered equally remote based on the square root of their populations. If we used $\gamma = 5/6$, then the central part of Mexico City would correspond to a disc with a radius of 3.8 km in Merida (Figure 1). Therefore, using this value for the scaling surface coefficient would only slightly compress the cumulative curves.

While it would not alter the loss of urban population near the centre of all cities in Mexico, density values would scale as $1 - 2\beta = 1/6$ (equation (2)), distorting our comparison of population densities and making them less interpretable. Moreover, this exponent was obtained for the scaling of the actual

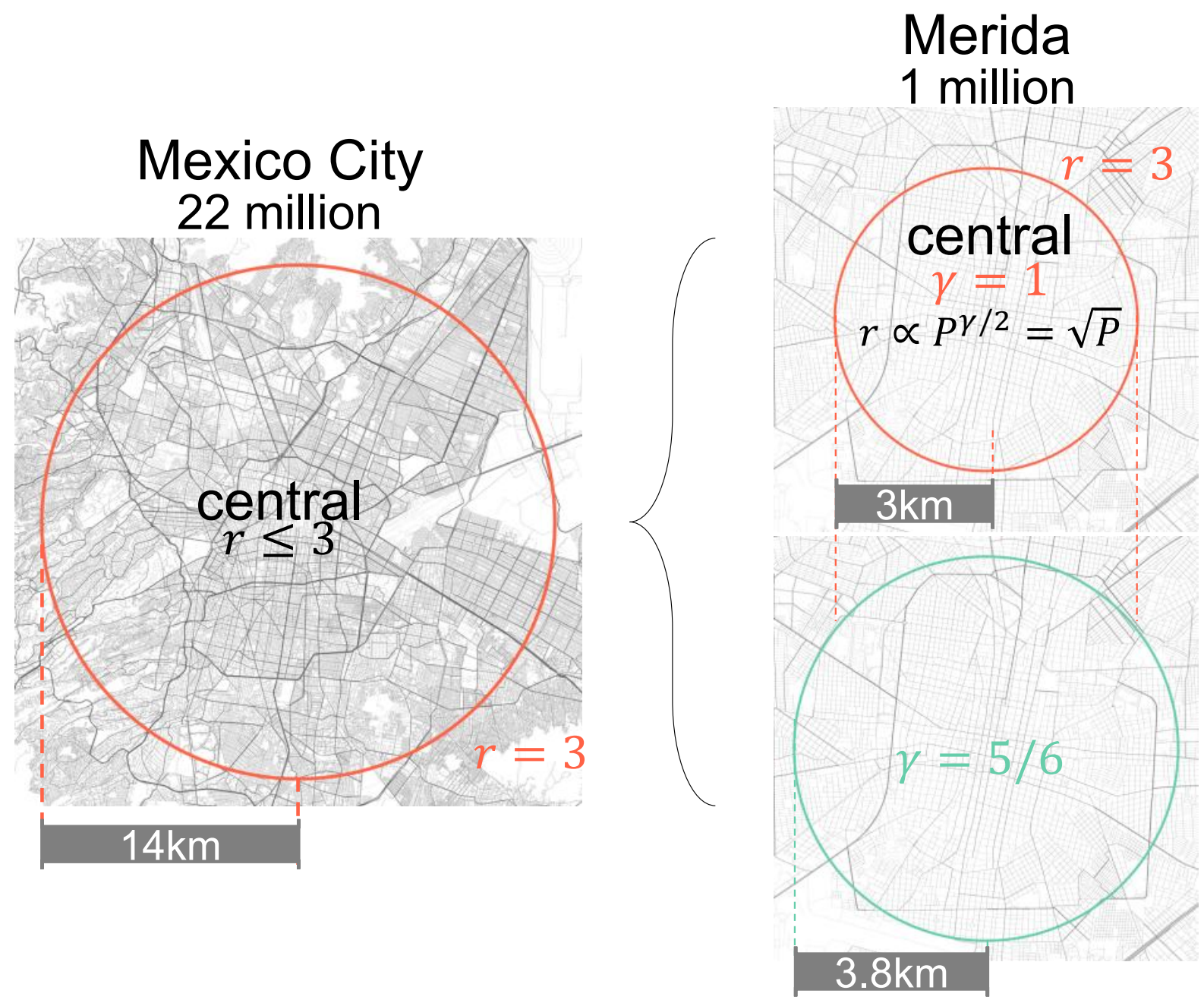

Figure 1: The central part of Mexico City, identified as the region with a remoteness smaller than three units (left), corresponds to a smaller disc in Merida (right). The core difference between the two cities is that Mexico City has over 22 million inhabitants, while Merida has roughly 1 million. Applying a different definition of remoteness (based on area, with the corresponding pre-factor to obtain the same region in Mexico City) has a minor impact on the definition of Merida's central part.

urbanised area within metropolitan areas, not the total extent of the city, taken as the disk that inscribes the city, used when performing radial analysis. Additionally, the temporal analysis of urban expansion (meaning the estimation of the urban expansion factor Φ_{ij} and finding that all cities expanded their radial distribution while losing central density) is conducted independently for each city using actual physical distances s. Remoteness is used only for cross-city comparisons of density profiles and for defining zone boundaries. The observation that $\Delta\sigma < 0$ near the centre of every city follows directly from each city's raw radial data and is entirely independent of the cross-sectional scaling assumption.

It is possible to utilise remoteness and alternative scaling exponents within our scaling framework. By dropping time dependence, we can apply the scaling described by equations (2) and (3) of the main text to a transversal comparison of cities. These results in the following equations for the urban expansion factor

$$\Phi_{io} = \left(\frac{P_o}{P_i}\right)^{\beta}, \tag{1}$$

and population density

$$\sigma_o(s) = \left(\frac{P_o}{P_i}\right)^{1-2\beta} \sigma_i\left(\frac{s}{\Phi_{io}}\right), \tag{2}$$

where o refers to the scaled version of city i, with population P_o. Remoteness is obtained by setting $\beta = 0.5$ and $P_o = 1 \times 10^6$. Note that for $\beta = 0.5$, the factor $\left(\frac{P_o}{P_i}\right)^{1-2\beta} = 1$, and population density values are preserved after scaling. By using remoteness, we scale urban areas to a similar city with a

population of 1 million and an equivalent population density. Since population density is preserved, we can compare population densities of the rescaled cities (as we do in Section 2.1 of the main text). Recently, a scaling exponent of $\beta = 1/3$ was proposed in a study using radial analysis on a global sample of cities [4]. There, a special case of equation (2) was suggested, deemed *homothetic* scaling, of the form

$$\sigma_o(s) = \left(\frac{P_o}{P_i}\right)^{\beta} \sigma_i \left(\left(\frac{P_i}{P_o}\right)^{\beta} s\right), \tag{3}$$

where the exponent that best fitted the sample of cities was $\beta = 1/3$. A possible explanation for this result is that the obtained $\beta = 1/3$ is the only value for β that is compatible with equation (2) (a solution to $1 - 2\beta = \beta$). Any other exponent would result in a transformation that is not a scaling, as radial probability densities ρ of transformed cities would not be normalised (see Methods in main text). Therefore, it is unclear if using the more general model (2) would yield a different exponent. However, using $\beta = 1/3$ would also alter population density values and impede comparisons between cities.

0.1 Population redistribution under alternative scaling exponents

While remoteness is adequate for density comparisons under scaling, it is informative to explore how the observed redistribution changes under the alternative scaling exponents. We perform a sensitivity analysis re-classifying each city's population into the four remoteness zones under two alternative values of β: the Urban Scaling Theory prediction $\beta = 0.425$ ($\gamma = 5/6$) [1, 3], and the homothetic scaling exponent $\beta = 1/3$ ($\gamma = 2/3$) [4]. For each alternative value, we recompute the total population in the central zone ($r < 3$) and the aggregate loss between 1990 and 2020 (Supplementary Figure 2).

Across all tested exponents, Mexican metropolitan zones continue to show a net population decline in their central zone, confirming that the finding is robust to the choice of parameter values (Supplementary Table 1).

Table 1: Sensitivity of central population loss to the choice of scaling exponent β. "Central loss" is the aggregate population decline in zones classified as central ($r < 3$) between 1990 and 2020 across all 69 metropolitan areas.

Scaling	β	Central loss (millions)
Standard remoteness	0.5	2.5
Urban Scaling Theory	0.425	2.4
Homothetic	1/3	2.2

The aggregate loss estimate changes because a smaller β compresses zone boundaries inward for larger cities, shrinking the physical extent of what is classified as "central". Yet, even in those different scenarios, the central parts of cities in Mexico lost, at least, 2.2 million inhabitants.

The deeper methodological reason for choosing $\beta = 0.5$ is different, and is independent of whether μ is truly constant. Remoteness is used in this paper to compare population density profiles *across* cities

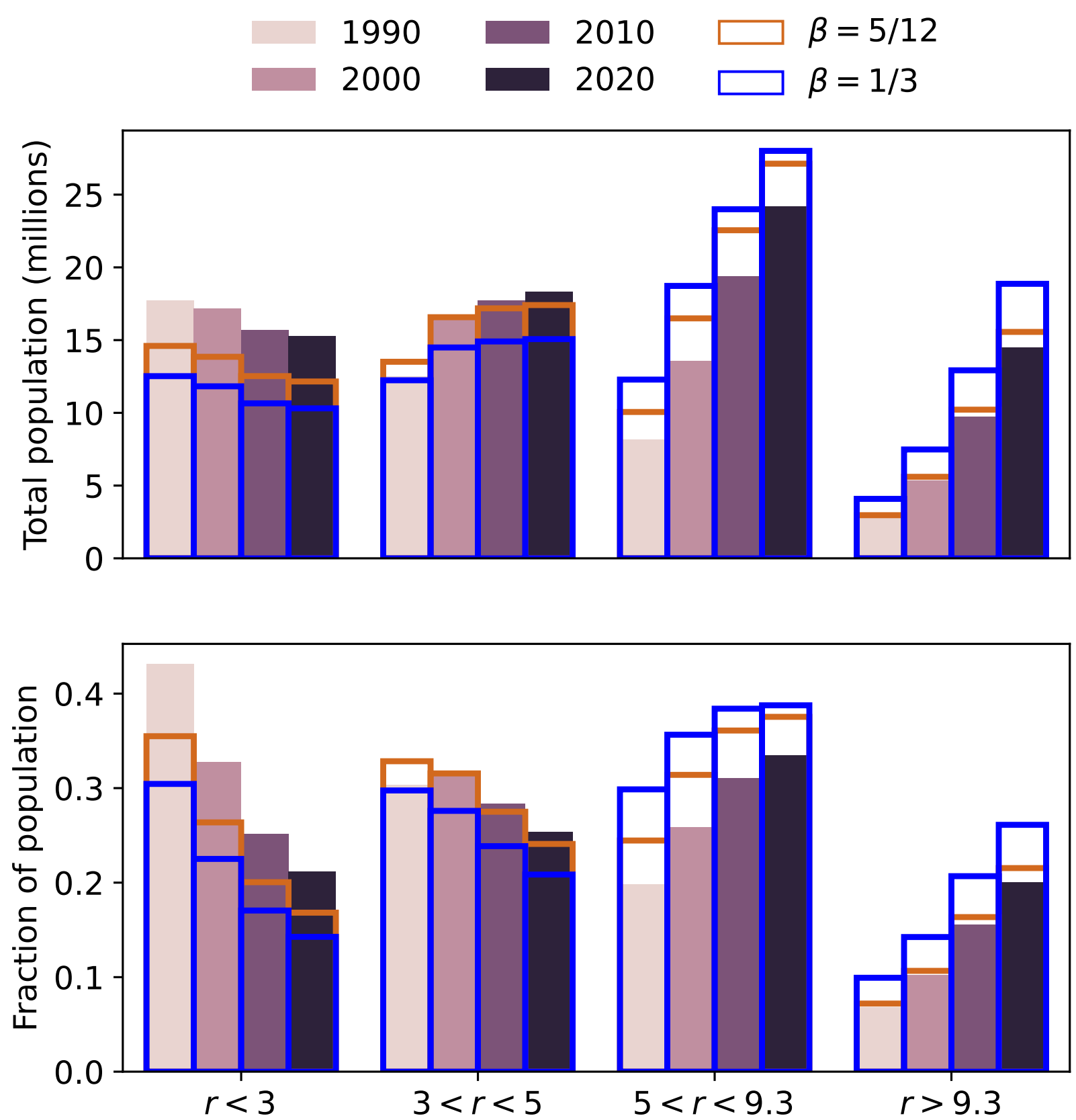

Figure 2: Population and population fraction aggregated over all metropolises at different remoteness brackets for all census years. Coloured bars correspond to remoteness scaling ($\beta = 0.5$), hollowed bars to UST ($\beta = 5/12$, brown) and homothetic scaling ($\beta = 1/3$, blue).

of different sizes. For this cross-sectional comparison to be meaningful, rescaling must not distort the density values themselves. Under the general rescaling (Supplementary Note 2, equation 3):

$$\sigma_o(s) = \left(\frac{P_o}{P_i}\right)^{1-2\beta} \sigma_i\left(\frac{s}{\Phi_{io}}\right), \tag{4}$$

the prefactor $(P_o/P_i)^{1-2\beta}$ equals one *if and only if* $\beta = 0.5$. For any other value of β, rescaling modifies population density values in proportion to the population ratio, making cities with very different populations incomparable in density terms. In other words, $\beta = 0.5$ is not merely one plausible assumption among others; it is the *unique* choice that allows direct, unbiased comparison of population densities across cities. The fact that some large Mexican cities may be somewhat denser than smaller ones does not change this: using a different β to "correct" for the density–size relationship would, by the same token, introduce a size-dependent distortion into the densities being compared, defeating the purpose of the cross-city comparison.

We have added a sentence to the relevant paragraph in Supplementary Note 2 making this reasoning

explicit.

The temporal analysis is entirely independent of the cross-sectional scaling assumption. The urban expansion factor Φ_{ij} and the temporal scaling of radial distributions are estimated for each city individually in terms of the actual physical distance s from the city centre. Remoteness does not enter this analysis at all. The observation that population density declined near the centre of every city—captured in Figure 3 of the manuscript as $\Delta\sigma < 0$ for small distances from the centre—follows directly from each city's raw radial data. It is independent of any assumption about how city radius scales with population.

Remoteness enters the paper in two auxiliary roles: (i) to enable the cross-city overlay of density profiles (Figures 3 and 5), for which $\beta = 0.5$ is uniquely appropriate as argued above, and (ii) to define the zone boundaries ($r < 3$ for central, etc.) used to aggregate populations across cities (Figure 2b–c and the 2.5 million figure). The zone boundaries were originally calibrated from data on urban land use and walkability [5], and the aggregate figure of 2.5 million is conditional on those boundaries. Both of these uses concern the *cross-sectional* comparison of cities; neither affects the temporal finding of universal central population loss established city by city.

We have added a clarifying sentence to Supplementary Note 2 to make the independence of the temporal analysis explicit, and we have updated the surrounding text to distinguish more clearly between the cross-sectional and temporal roles of remoteness.

Thank you again for the constructive engagement with our work.

References

[1] Luís MA Bettencourt. The origins of scaling in cities. *Science*, 340(6139):1438–1441, 2013.

[2] Luís M. A. Bettencourt and José Lobo. Urban scaling in Europe. *Journal of The Royal Society Interface*, 13(116):20160005, March 2016. Publisher: Royal Society.

[3] Jose Lobo, Scott Ortman, and Luis Bettencourt. Systematic Relationship Between Areal Extent and Population for Urban Areas in the USA, December 2024. ISSN: 2693-5015.

[4] Gaëtan Laziou and Rémi Lemoy. The three-dimensional structure of population density in world cities. *npj Urban Sustainability*, 5(1):75, 2025.

[5] Rafael Prieto-Curiel, Pavel Luengas-Sierra, and Christian Borja-Vega. Urban sprawl is associated with reduced access and increased costs of water and sanitation. *Nature Cities*, pages 1–12, 2025.
